# Phylogenetic characterization of bacterial endophytes from four *Pinus* species and their nematicidal activity against the pine wood nematode

**DOI:** 10.1038/s41598-019-48745-6

**Published:** 2019-08-28

**Authors:** Lakshmi Narayanan Ponpandian, Soon Ok Rim, Gnanendra Shanmugam, Junhyun Jeon, Young-Hwan Park, Sun-Keun Lee, Hanhong Bae

**Affiliations:** 1 Department of Biotechnology, Yeungnam University, Gyeongsan, Gyeongbuk 38541 Republic of Korea; 2 Nakdonggang National Institute of Biological Resources, Sangju, 37242 Republic of Korea; 3 Division of Forest Insect Pests and Diseases, National Institute of Forest Science, Seoul, 02455 Republic of Korea

**Keywords:** Symbiosis, Bacteriology

## Abstract

Recently, bacterial endophytes (BEs) have gained importance in the agricultural sector for their use as biocontrol agents to manage plant pathogens. Outbreak of the pine wilt disease (PWD) in Korea has led researchers to test the feasibility of BEs in controlling the pine wood nematode (PWN) *Bursaphelenchus xylophilus*. In this study, we have reported the diversity and biocontrol activity of BEs against the PWN. By employing a culture-dependent approach, 1,622 BEs were isolated from the needle, stem, and root tissues of *P*. *densiflora*, *P*. *rigida*, *P*. *thunbergii*, and *P*. *koraiensis* across 18 sampling sites in Korea. We classified 389 members based on 16S rDNA analysis and taxonomic binning, of which, 215 operational taxonomic units (OTUs) were determined. Using Shannon’s indices, diversity across the *Pinus* species and tissues was estimated to reveal the composition of BEs and their tissue-specific preferences. When their ethyl acetate crude extracts were analysed for biocontrol activity, 44 candidates with nematicidal activity were obtained. Among these, *Stenotrophomonas* and *Bacillus* sp. exhibited significant inhibitory activity against PWN during their developmental stages. Altogether, our study furnishes a basic comprehension of bacterial communities found in the *Pinus* species and highlights the potential of BEs as biocontrol agents to combat PWD.

## Introduction

Due to the economic and ecological significance of pine trees, the outbreak of pine wilt disease (PWD) in Korea, which is causing inevitable damage to its ecosystem and economy, has become a reason for serious concern^1,2^. PWD, caused by *Bursaphelenchus xylophilus* and commonly referred to as the pine wood nematode (PWN), affects various species of pine trees and induces aberrations in the vascular vessels of the hosts, eventually advancing into the development of wilting symptoms^3^. Following initial reports on PWN observed in the USA, Canada, China, Japan, and other south-eastern countries, the first incidence of PWD in Korea was documented in 1988, in which the PWN destroyed several hectares of the pine forest area^4,5^. Unlike the three pine species (*P*. *densiflora*, *P*. *koraiensis*, and *P*. *thunbergii*), *P*. *rigida* is native to North America and found to be resistant to PWD^6^.

Until now, methods employed for the preventive management and control of PWN from infected trees included clear-cutting, burning wilt-woods, and the application of nematicidal compounds^7^. Although injecting commercially existing nematicides such as emamectin benzoate, abamectin, and oxolinic acid into the pine trunk^8,9^ often yields positive results, its hazardous impact on human health and other environmental ecosystems often becomes a major issue. To circumvent and address these challenges, currently, developing a safer and cost-effective alternative is one of the prime areas of research. More recently, studies focusing on the use of BEs as biocontrol agents have shown a promising glimpse of their remedial application in the agriculture sector^10^. In general, BEs colonizing host plants have the tendency to demonstrate antagonistic activity against their own or different plant pathogens^11,12^. These beneficial activities are due to the production of secondary metabolites by BEs, many of which are used for various applications^13^. For instance, bacterial endophytes of *Pseudomonas*, *Bacillus*, and *Methylobacterium* isolated from the banana, chili, papaya, paddy, and coffee plants proved to be effective against the root-knot nematode *Meloidogyne incognita*^14,15^. Thus, due to the proven potential of BEs as biocontrol agents and the economic importance of pine trees, we hereby intend to propose a hypothesis on the basis of the following studies and objectives: (1) screening and isolation of BEs from four *Pinus* species; (2) investigating the selected isolates for biocontrol activity against different developmental stages of the PWN *in vitro*.

## Results

### Isolation and identification of bacterial endophytes

**Figure 1:**
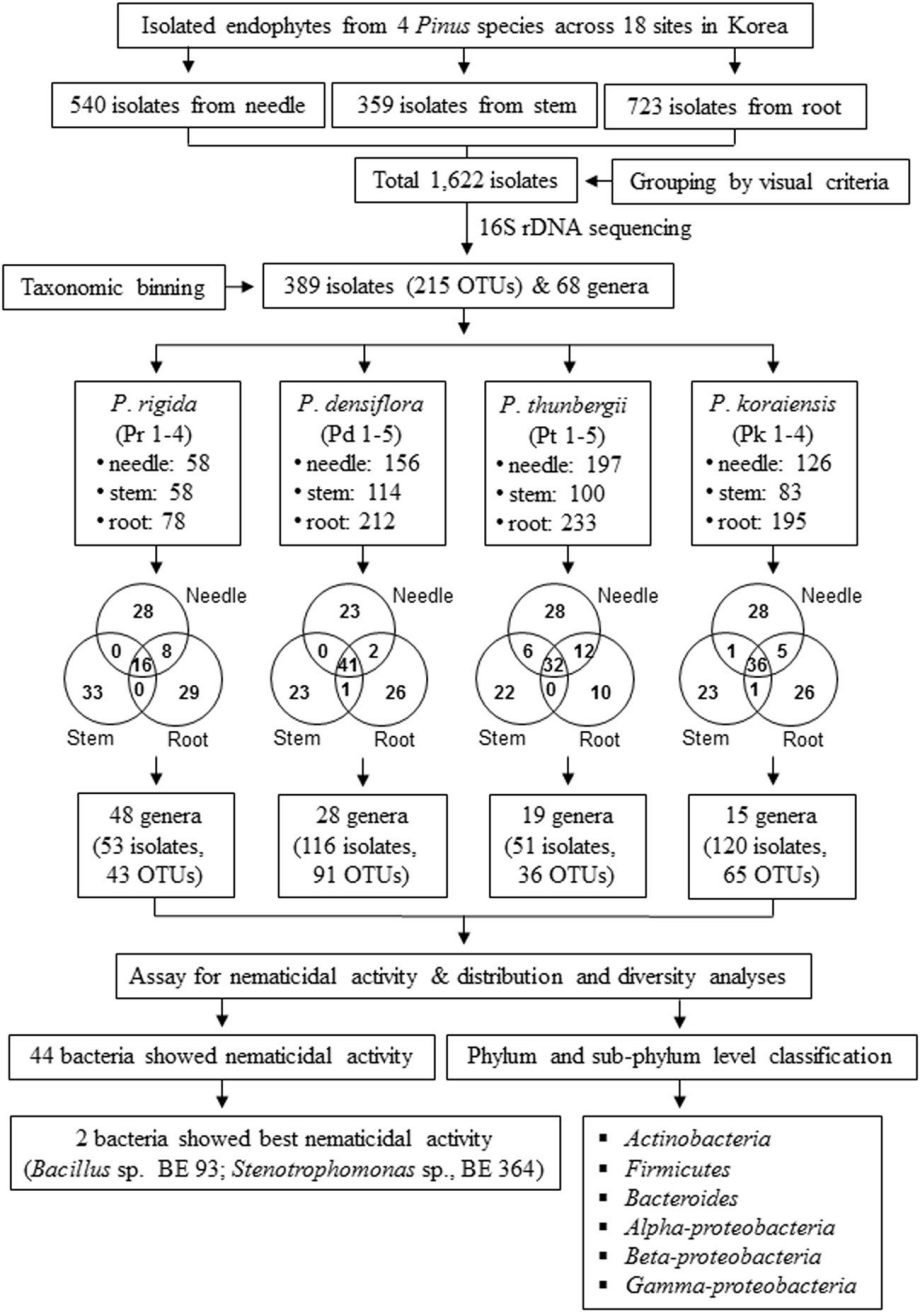
Schematic representation of workflow: isolation and identification of culturable bacterial endophytes from four *Pinus* species across 18 sampling sites in Korea.

In total, 1,622 culturable BEs were isolated from three different tissues (needles, stem, and root) of the four *Pinus* species across 18 sampling sites in Korea (Table 1). Based on the results of 16S rDNA sequencing, they were classified into 389 distinct bacterial groups (Note S1). Upon taxonomic binning of these 389 isolates, 215 operational taxonomic units (OTUs) encompassing 68 different genera were identified. Majority of the identified OTUs were endophytes, which contribute to the growth-promotion and development in plants and demonstrated more than 99% similarity with the reference strains (Fig. 1: Supplementary Table S1).

**Table 1:** Sampling overview of bacterial endophytes.

Pine species	Sampling site (symbol)	Estimated tree age	Number of isolates		
			Tissue		
			Needle	Stem	Root
*Pinus rigida*	Anseong (Pr 1)	45	12	15	21
	Seosan (Pr 2)	45	14	10	13
	Jungeup (Pr 3)	45	16	14	21
	Yungyang (Pr 4)	43	18	19	23
*Pinus densiflora*	Jungeup (Pd 1)	50	25	30	40
	Jeju island (Pd 2)	50	28	29	45
	Hoengseong (Pd 3)	45	40	25	43
	Bonghoa (Pd 4)	35	30	18	46
	Yangpung (Pd 5)	50	33	14	43
*Pinus thunbergii*	Pohang (Pt 1)	40	36	17	46
	Jeju island (Pt 2)	45	40	21	57
	Seosan (Pt 3)	50	46	22	41
	Gangneung (Pt 4)	35	47	24	40
	Yeosu (Pt 5)	45	29	18	49
*Pinus koraiensis*	Hongchun (Pk 1)	30	30	22	51
	Jecheon (Pk 2)	30	33	21	43
	Chuncheon (Pk 3)	35	31	18	56
	Pocheon (Pk 4)	50	32	22	45
Total			540	359	723

The table illustrates the number of isolates obtained from the needles, stems, and roots of four *Pinus* species from 18 sampling sites across Korea. Six trees were sampled from each sampling site.

**Figure 2:**
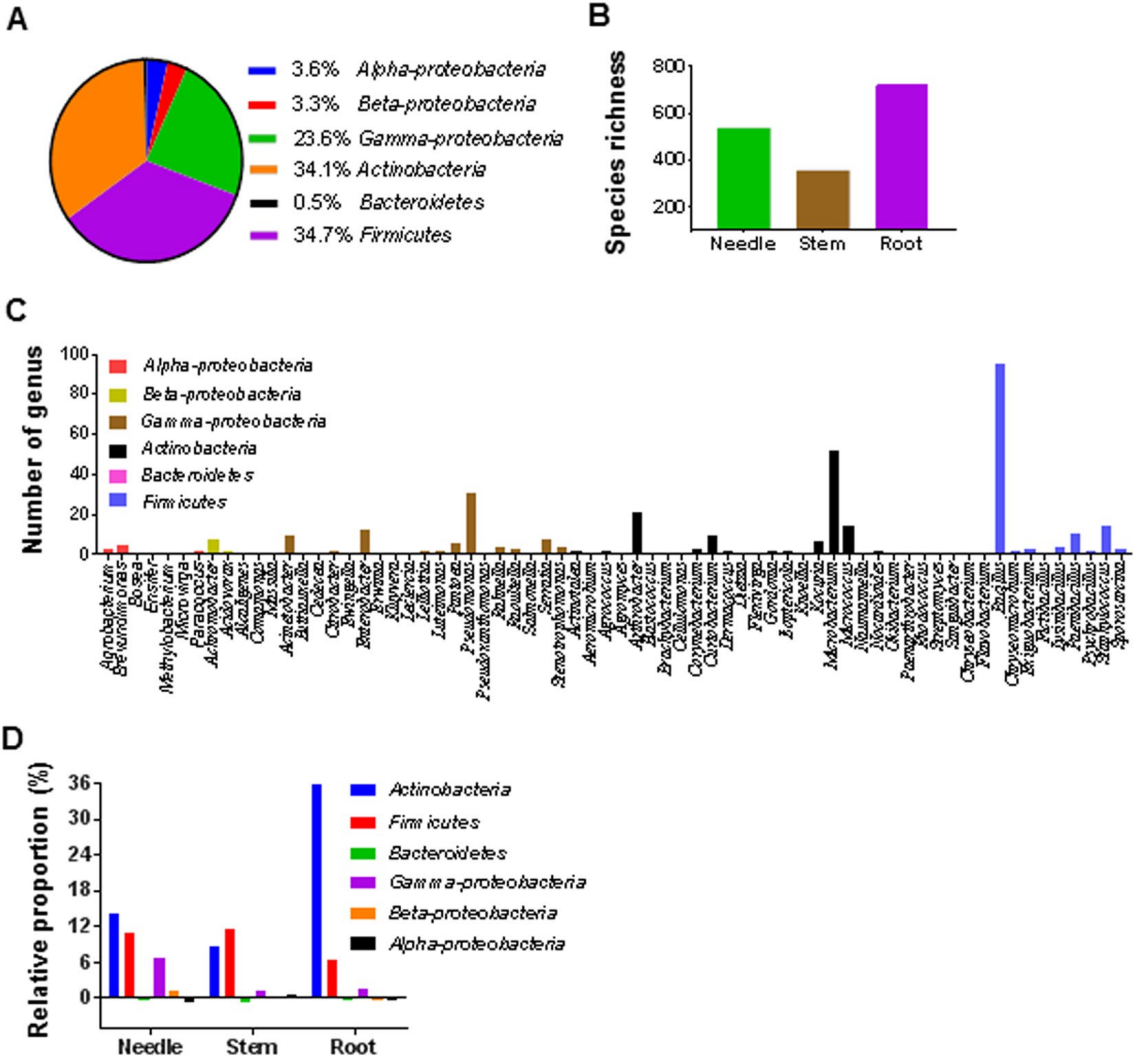
Distribution of culturable bacterial endophytes in the needles, stems, and roots of four *Pinus* species across 18 sampling sites. (**A**) Pie chart representing the percentage of bacterial endophytes shared by different phyla. (**B**) Bar graph showing the number of bacterial endophytes isolated from the needle, stem and root tissues. (**C**) Distribution of bacterial endophytes at the genus level (68 genera in total). Diverse colours indicate the phyla to which each genus belongs. (**D**) Relative proportion of the bacterial endophytes in different tissues at the phylum and sub-phylum level.

Examining the phylogenetic relationship among the 389 isolates showed a confined range of taxonomical distribution. Accordingly, all the isolates were grouped into 4 phyla as follows: Actinobacteria, Bacteroidetes, Firmicutes, and Proteobacteria (alpha, beta, and gamma). It was observed that majority of the OTUs predominantly belonged to the phyla Firmicutes followed by Actinobacteria and comprised 34.7% and 34.1% of the total members, respectively. Approximately, 23.6% of the total isolates were gamma-proteobacteria whereas those constituting the class of alpha- and beta-proteobacteria were less than 5% (Fig. 2A).

To explore the diversity and distribution of BEs in different tissues, the 1,622 isolates collected from four *Pinus* species were analysed. Their results revealed that the isolate count was the highest in the roots (723), followed by needles (540), and the lowest in stems (359) (Fig. 2B). In both *P*. *densiflora* and *P*. *koraiensis*, only members of a specific phylum were found, and their occurrence was restricted to a single type of tissue in the plant. For example, in *P*. *koraiensis*, gamma-proteobacteria belonging only to the genus *Pseudoxanthomonas* were found exclusively in the needles. Similarly, in *P*. *densiflora*, Actinobacteria belonging exclusively to the genus *Kocuria* were present in its root tissues. Thus, in the aforementioned tree species, we observed that no endophyte from the phyla Actinobacteria or Firmicutes were distributed in any other tissues. Contrarily, a relatively broader diversity and distribution was observed in members of *P*. *rigida* and *P*. *thunbergii*. For instance, the four phyla were found to be distributed within specific tissues of *P*. *rigida*. BEs of 12 genera related to the Gamma-proteobacteria were found to be heavily distributed in the needle tissues. However, in *P*. *thunbergii*, it was observed that 2 genera in the stem (alpha-proteobacteria) and 1 in the root (Actinobacteria) were distributed. Contrarily, no specific genus was found in the needles. Taken together, these results conveyed that the diversity and distribution of BEs among different tissues tends to be species- and tissue-specific (Supplementary Table S2).

**Figure 3:**
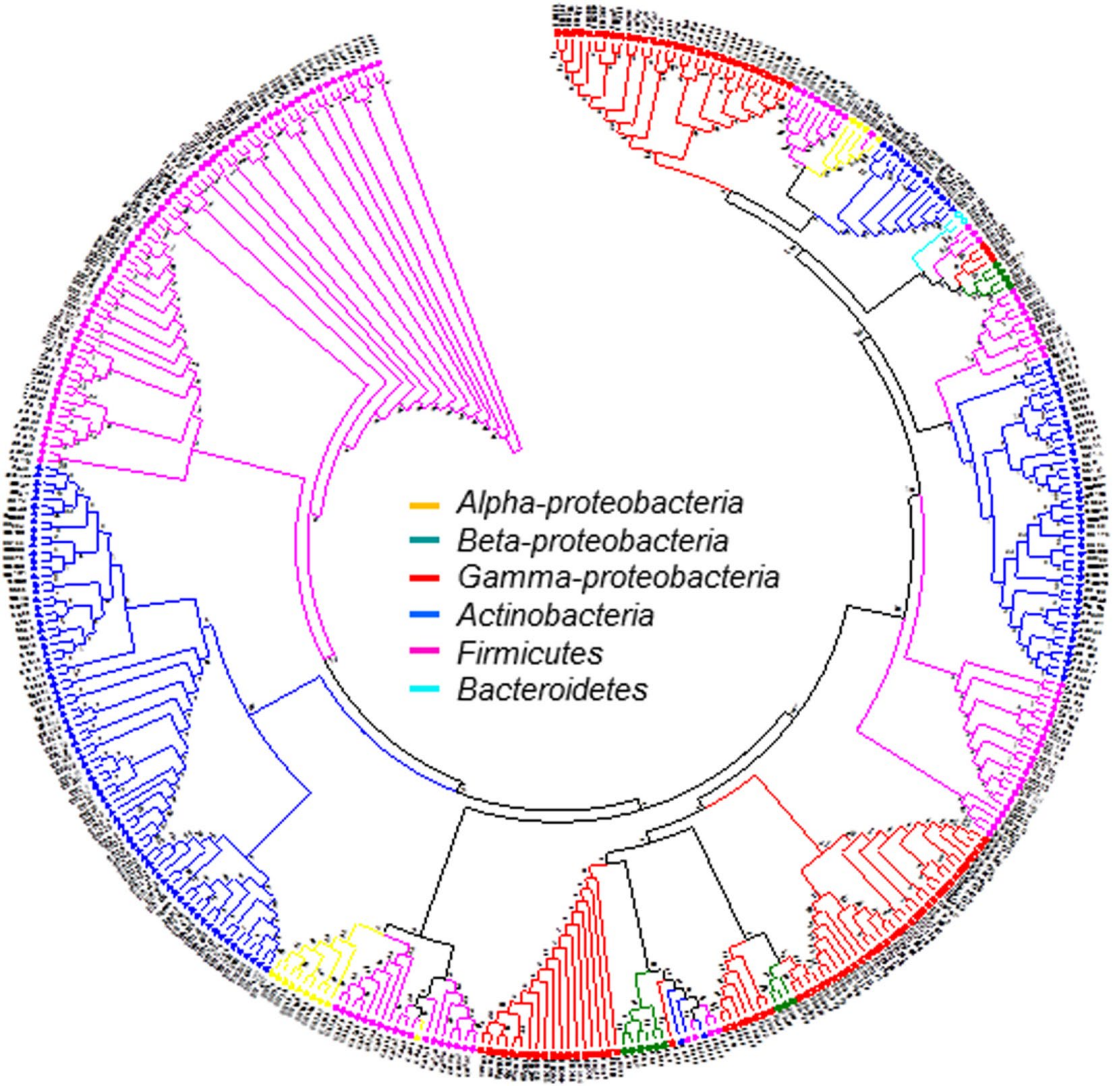
The phylogenetic tree was created using neighbour-joining methods with 16S rDNA sequence isolates from various tissues of four *Pinus* species. Bootstrap values expressed as the percentage of 1,000 replications are indicated at the nodes.

Among all the genera, the presence of *Bacillus* belonging to the phylum Firmicutes was predominant followed by that of *Microbacterium* of phylum Actinobacteria, present in 95% of Firmicutes, and 52% of Actinobacteria, respectively. *Pseudomonas* was the only genus of Proteobacteria that demonstrated a frequency of occurrence as high as 31% (Fig. 2C). The relative proportions of the different phyla and sub-phyla found in all sampling sites were analyzed. *Actinobacteria* were present most abundantly in the needle and stem tissues, while the highest number of Firmicutes members was found in the stems. In the needles, the counts of alpha-proteobacteria and gamma-proteobacteria were the maximum and minimum, respectively, while no beta-proteobacteria were found in the stems. Compared to all other phyla, the population of Bacteroidetes was the least in all the three tissues (Fig. 2D). The phylogenetic tree showed the relationship between the different isolates of BEs and the range of their taxonomical distribution. All the representative isolates were clustered into the following 4 phyla: Actinobacteria, Bacteroidetes, Firmicutes and Proteobacteria. The phylum and sub-phylum occurring most frequently was Actinobacteria, which included 113 isolates, followed by Firmicutes constituting 135 isolates. These results demonstrated the diversity among bacterial endophytes collected from the three different tissue types of four *Pinus* species located at different sites (Fig. 3).

### Diversity of bacterial endophytes among the pinus species

**Figure 4:**
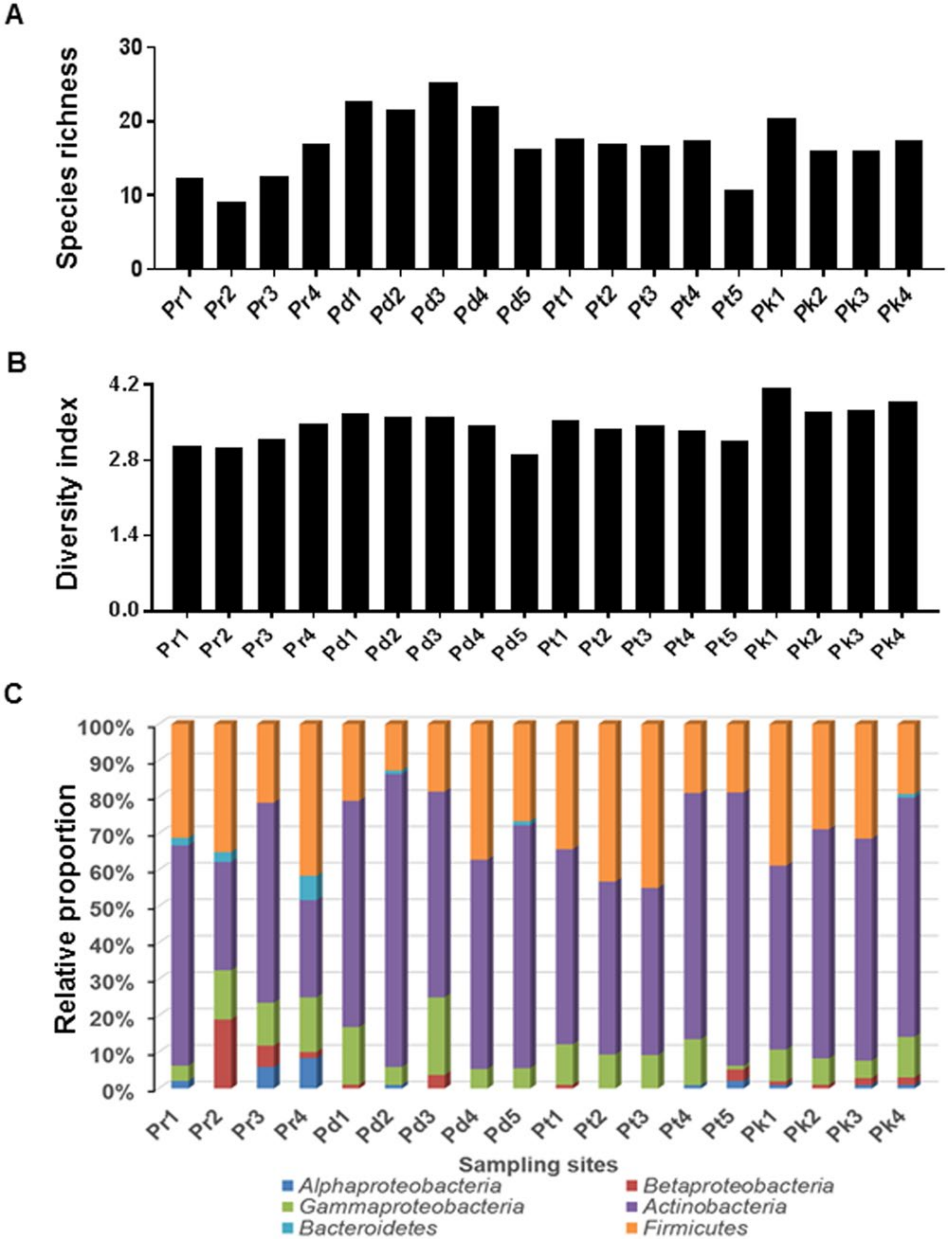
Distribution and diversity of the bacterial endophytes in four *Pinus* species. (**A**) Species richness across the sampling sites. (**B**) Shannon’s index (H′) across the sampling sites. (**C**) Relative proportion of bacterial endophytes at the phylum level across the sampling sites.

Differences in the diversity of BEs were observed in all samples collected from the four *Pinus* species. Significant to negligible variations in the diversity of BEs were noted within the same species collected from different locations. The species richness (S) showed that *P*. *densiflora* harboured the maximum number of BEs (average 21 species), followed by *P*. *koraiensis* (average 17 isolates) and *P*. *thunbergii* (average 15 species) (Fig. 4A). The least species richness was observed in *P*. *rigida* (average 12 species). Similar values of the Shannon index (H′ = 3 to 4) indicated that no significant differences were noted in the diversity of BEs among all the tested *Pinus* species (Fig. 4B). All the 389 isolates were classified at the phylum level and their relative abundance in all sampling sites was calculated. Members of Actinobacteria, Firmicutes and gamma-proteobacteria were detected in samples collected from all the sampling sites. The distribution of the members of alpha- and beta-proteobacteria was limited in a few sampling sites while that of Bacteroides was the least. (Fig. 4C).

Furthermore, in order to explain the role of edaphic factors in determining the differences in the distribution and composition of BEs in the *Pinus* species, factors such as soil pH, phosphate content, altitude, precipitation, and mean annual temperature were assessed. The results demonstrated that no comparable or significant changes in the diversity and species richness that were influenced either by climate or any other edaphic factors were noticed. (Supplementary Tables S2 and S3; Supplementary Figs S1 and S2).

### Identification of bacterial endophytes with potential nematicidal activity

**Figure 5:**
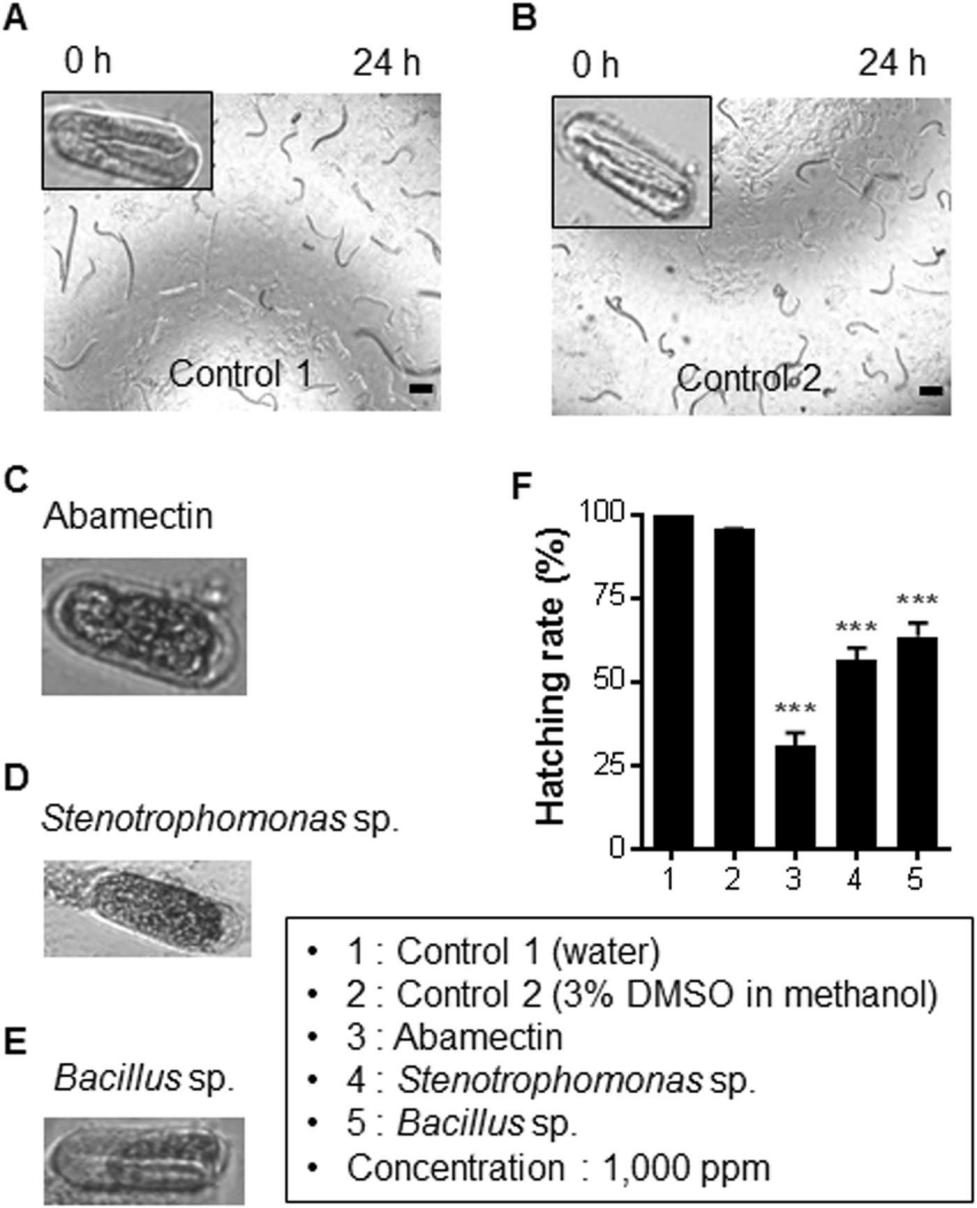
Effects of ethyl acetate extracts of two potential bacterial endophytes on egg hatchability of *Bursaphelenchus xylophilus*. The eggs and nematodes were monitored at different time points (0, 6, 12, 24, 48, and 72 h) and all pictures were taken at 24 h at a concentration of 1,000 ppm of ethyl acetate extracts. (**A**) Control 1: double distilled water. (**B**) Control 2: 3% DMSO in 100% methanol. (**C**) Control 3: abamectin. (**D**) *Stenotrophomonas* sp. (**E**) *Bacillus* sp. (**F**) Percentage of inhibition of egg hatching. Data are represented as the means ± standard error of the average of three trials with eighteen replications. ***P < 0.001 vs. control 2. Scale bars = 100 μm.

**Figure 6:**
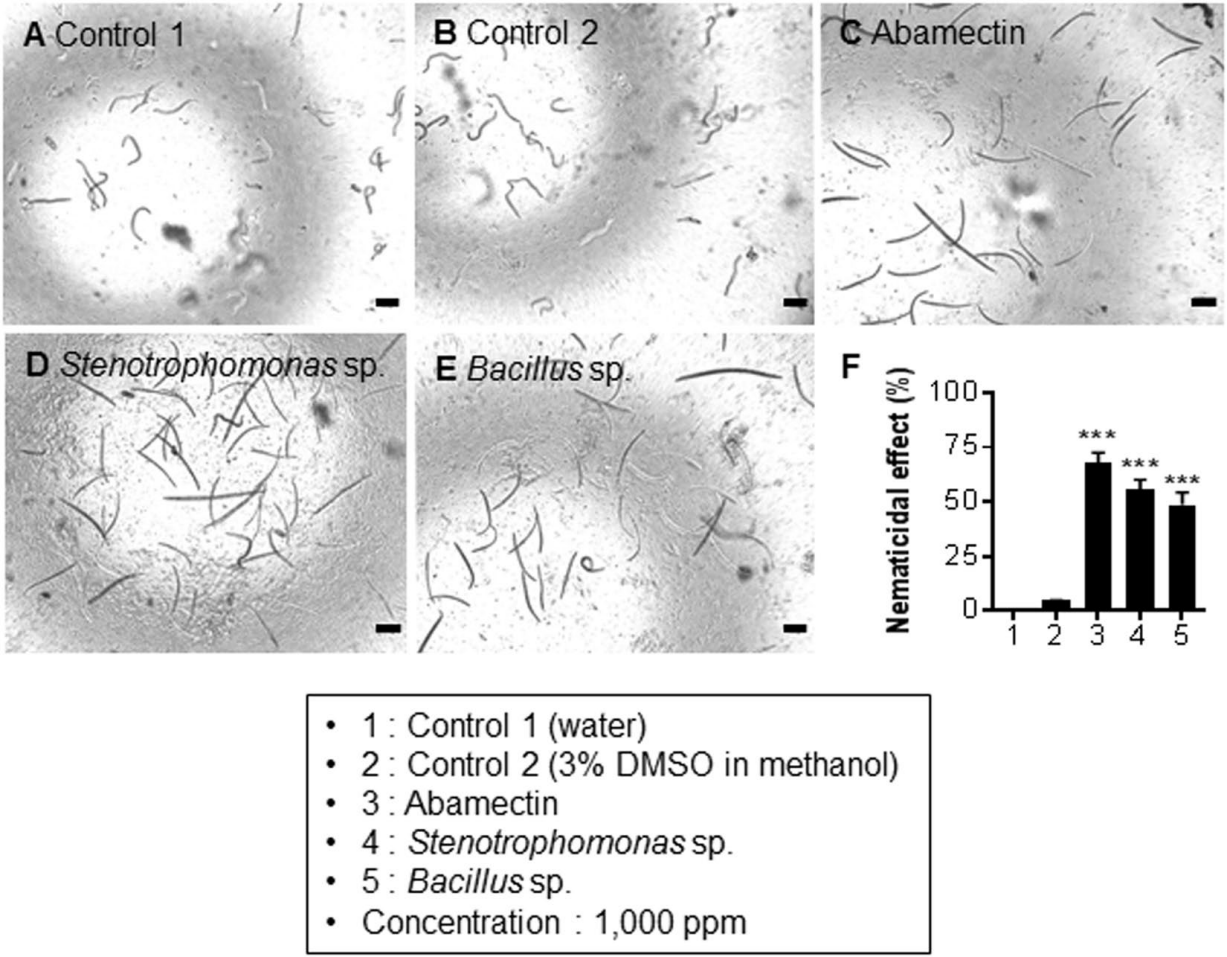
Nematicidal activity of the ethyl acetate extracts of two potential bacterial endophytes on the L3/L4 stages of *Bursaphelenchus xylophilus*. The nematodes were monitored at different time points (0, 12, 24, 48, and 72 h) and all pictures were taken at 24 h at a concentration of 1,000 ppm. (**A**) Control 1: double distilled water. (**B**) Control 2: 3% DMSO in 100% methanol. (**C**) Control 3: abamectin. (**D**) *Stenotrophomonas* sp. (**E**) *Bacillus* sp. (F) Percentage of dead nematodes (L3/L4 stages). Data are represented as the means ± standard error of the average of three trials with eighteen replications. Average size of the L3/L4 nematodes is 208 µm. ***P < 0.001 vs. control 2 (3% DMSO in methanol). Scale bars = 100 μm.

Nematicidal activities of the cellular extracts (CEs) of the 389 BEs at a concentration of 1,000 ppm were screened. Mortality rates of the PWNs were confirmed based on microscopic visualization (Logos Biosystems, iRiS^TM^ digital cell imaging system, Anyang, Korea) of the dead and live nematodes. Primarily, 44 isolates exhibited nematicidal activity on the L3/L4 and adult stages of the nematodes after treatment for 24 h (Supplementary Fig. S3). Moreover, among the 44 isolates, *Stenotrophomonas* (EB 394) and *Bacillus* sp. (EB 93) consistently exhibited high nematicidal activity against the PWNs (Figs 5 and 6).

The inhibitory effects of CEs on egg hatching of *B*. *xylophilus* were evaluated by exposing the eggs to the 44 nematicidal CEs after 24 h treatment. The cumulative number of eggs that hatched between 12 h and 24 h after treatment was considered for determining the egg hatching percentage. We observed that the CEs from two isolates had strong inhibitory effect on the egg hatchability in a dose/concentration-dependent manner, whereas no inhibitory effect was observed in control 1 (Fig. 5A; Movie S1). In control 2 (3% DMSO in methanol) and the positive control (abamectin), the rate of egg hatching was 96% (Fig. 5B; Movie S2) and 30% (Fig. 5C; Movie S3), respectively. Interestingly, the hatching rates of our target CEs isolated from *Stenotrophomonas* sp. and *Bacillus* sp. were up to 56%. (Fig. 5D,F; Movie S4) and 63%, respectively (Fig. 6E,F; Movie S5). Additionally, the hatching rates of the remaining 42 CEs ranged from 73% to 82% (Fig. S3A).

From the results obtained in the egg hatchability experiment, we intended to evaluate the efficacies of the CEs on different developmental stages of the PWN. Significant nematicidal effect of the *Stenotrophomonas* sp. and *Bacillus* sp. was observed on two stages of nematode life cycle (L3/L4 and adult). The relative toxicity of the CEs on the nematodes in the L3/L4 stages was demonstrated by their microscopic visualization. Although no toxic effects were seen in control 1 (Fig. 6A; Movie S6) and control 2 (Fig. 6B; Movie S7), a mortality rate of approximately 67% was observed upon treatment with abamectin (Fig. 6C,F; Movie S8). Mortality rates of 55% and 48% were seen in *Stenotrophomonas* sp. (Fig. 6D,F; Movie S9) and *Bacillus* sp. (Fig. 6E,F; Movie S10), respectively.

**Figure 7:**
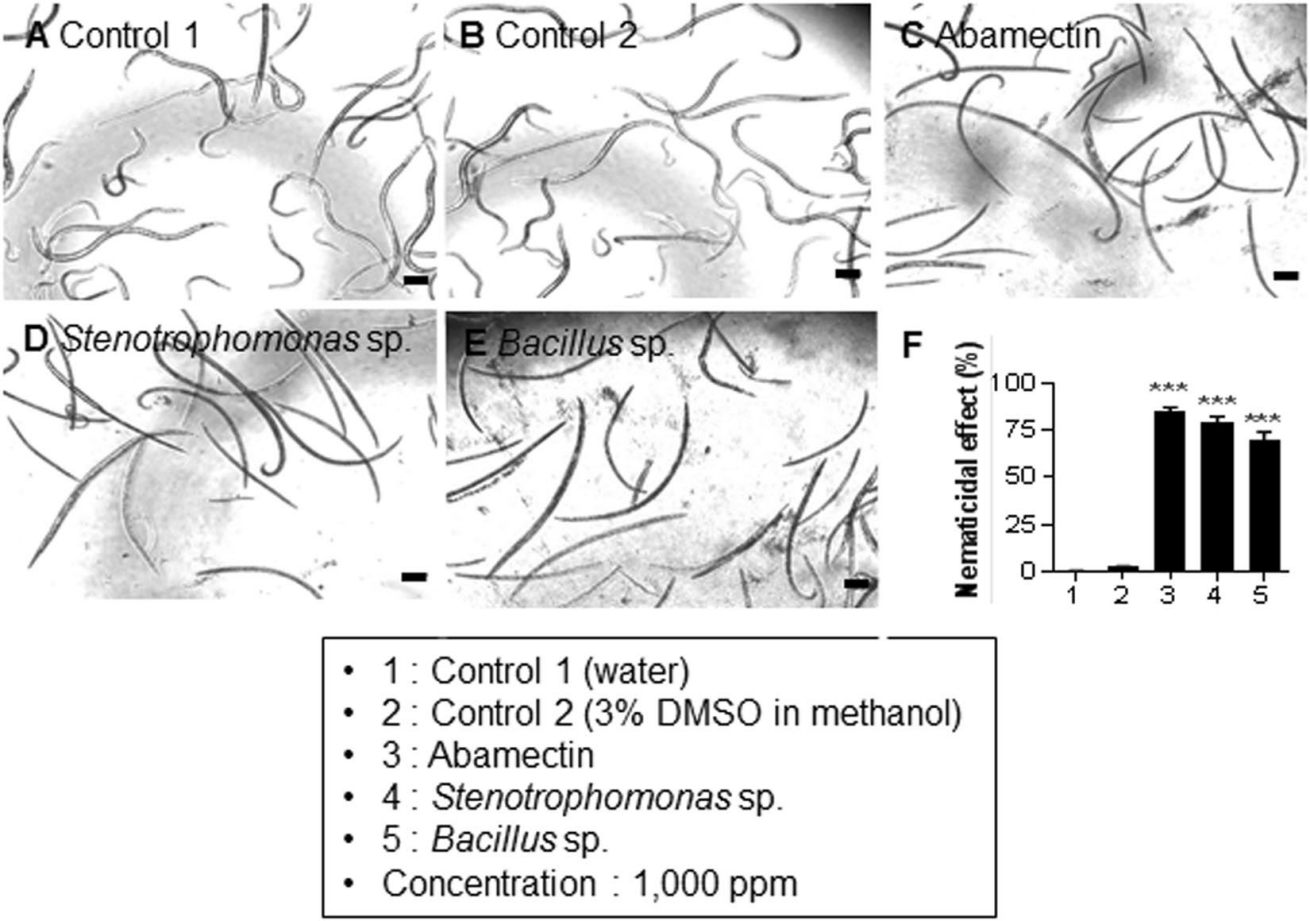
Nematicidal activity of ethyl acetate extracts of two potential bacteria on the adult stage of *Bursaphelenchus xylophilus*. The nematodes were monitored at different time points (0, 12, 24, 48, and 72 h) and all pictures were taken at 24 h at a specific concentration of 1,000 ppm. (**A**) Control 1: double distilled water. (**B**) Control 2: 3% DMSO in 100% methanol. (**C**) Control 3: abamectin. (**D**) *Stenotrophomonas* sp. (**E**) *Bacillus* sp. (**F**) Percentage of dead nematodes. Data are represented as means ± standard error of the averages of three trials with eighteen replications. Average size of the adult stage nematode is 773 µm. ***P < 0.001 vs. control 2. Scale bars = 100 μm.

Microscopic visualization of the adult stages showed live nematodes in control 1 (Fig. 7A; Movie S11) and control 2 (Fig. 7B; Movie S12). After treatment, the percentages of dead adult nematodes were as follows: 86% for abamectin (Fig. 7C,F; Movie S13), 79% for *Stenotrophomonas* sp. (Fig. 7D,F; Movie S14), and 70% for *Bacillus* sp. (Fig. 7E,F; Movie S15). Dead nematodes were paralyzed and straightened when transferred into clean water. Additionally, the mortality rates of the remaining 42 CEs were recorded to be in the range of 20% to 30% for the L3/L4 stages (Fig. S3B) and 30% to 35% for the adult stage (Fig. S3C). In summary, the results observed over a period of 12 h to 72 h demonstrated that mortality was induced after 24 h in response to abamectin and the two aforementioned CEs, under the defined concentrations and conditions.

The mortality rate was slightly higher for abamectin compared to that of the CEs. Unsurprisingly, mortality rates of 3% to 5% were observed in the water and solvent controls, and lower mortality rates were elicited by the remaining 42 CEs.

## Discussion

In recent decades, studies on the use of biological organisms as control agents for agricultural and biotechnological applications have increased considerably. One of the key factors influencing the rapid progress in this field is to identify and understand the ecology of novel BEs that are generally associated with the host plants^10^. Numerous reports suggest that various BEs existing within the tissues of living plants demonstrate antagonistic activity towards the pathogens, nematodes, and insects that attack the host plants^16^. Owing to the aforementioned beneficial aspects of BEs and current focus on the PWN, we hereby attempted to explore and identify the association of the BE community from four *Pinus* species and their nematicidal activity towards PWN.

The existence of diverse bacterial communities in the form of endophytes within the tissues of tree samples was illustrated using 16S rDNA analysis. The taxonomic binning of these samples presented 215 OTU belonging to 68 genera in total. It was found that the BEs from four different phyla, namely Actinobacteria, Firmicutes, Bacteroides, and Proteobacteria (alpha-, beta-, and gamma-) were distributed in needles, stems, and roots of all the sampled *Pinus* species. Interestingly, from the aforementioned BE communities, members of both Actinobacteria and Firmicutes constituted around 34% of the total population while Bacteroides, alpha-proteobacteria, and beta-proteobacteria accounted for less than 5%. In addition to the identification of the BE communities, investigating the species richness of the plants localized in different regions was a key factor since it indirectly helped in understanding their ecological relationships. In this regard, we observed that, in the entire population of *Pinus* species that was tested, the roots had harboured the maximum number of endophytes, followed by the stems and needles. This may be because the roots, being present in the soil rhizosphere, serve as a principal gateway for entry of the BEs, which can eventually migrate to other parts of the plants. For instance, per previous findings, it has been reported that *Exiguobacterium mexicanum* is a well-known endophyte that can migrate from the soil to the root and then to other parts of the plants^17^. Similarly, we found *Exiguobacterium aurantiacum* in three tissues of *P*. *rigida* indicating that this organism might have dispersed from the soil to the root and migrated further to its stems and needles.

Furthermore, in order to depict and explain the closest relationship between the BEs, a phylogenetic tree that clearly distinguished the phyla was constructed. Few species of *Microbacterium*, *Pseudomonas*, and *Bacillus*, being abundant in all our sampling sites, were documented as well-known endophytes in other hosts such as sweet corn, tomato, watermelon, bell pepper, and *Dendrobium candidum*^18,19^. Although most of the organisms of the genera mentioned this work have been recognized as common endophytes, through our work, species from the genera *Paenarthrobacter*, *Okibacterium*, *Flexivirga*, and *Naumannella* have been identified and reported for the first time as endophytes associated with pine trees. It is also noteworthy that *Cedecea neteri*, a human pathogen^20^, *Buttiauxella agrestis*, and *Ewingella Americana*, which are symbionts associated with *B*. *xylophilus*^21^, were present in all our samples. Taken together, our findings and their comprehensive analysis showed the diversity of BE communities and the level of the richness in species that were distributed among different parts of the pine trees.

Generally, secondary metabolites synthesized by the BEs help the host plants fight and protect against the pathogens^22^ or from any abiotic stress^23^. Several antimicrobial^13^, antifungal^11^ and antinematicidal^24^ activities of such secondary metabolites have been well documented^25^. Due to existing evidence of these inhibitory abilities, we investigated the antinematicidal activity of diverse BEs isolated from the four *Pinus* species. The 389 isolates belonging to different genera were tested *in vitro* to determine which isolates conferred antagonistic effects against the PWN. Our results showed that the CEs from 44 BEs could exert the nematicidal activity. The toxicity of the CEs towards the PWN was elucidated in three different phases. Egg hatchability being the critical factor in regulating population growth, we examined this factor initially. The results revealed that majority of the strains of the genera *Stenotrophomonas* and *Bacillus* showed marked toxicity on the egg hatching. Unlike abamectin, a commercial nematicide with 70% rate of inhibition, two potential BEs from our group inhibited the egg hatching at rates of 44% and 37%. Although CEs of the aforementioned BEs did not demonstrate significant activity like the synthetic compound abamectin, it is proven that the metabolites of these BEs are highly promising in killing the nematode. Additionally, considering the ill effects of the changes induced due to PWN development during PWD infection, we tried to explore the feasibility of using the CEs for inhibiting the nematode development. Interestingly, we found that 55% and 48% of mortality was achieved in the larval stage and 79% and 70% in adult stage of the PWN when treated with the CEs of *Stenotrophomonas* sp. (EB 394) and *Bacillus* sp. (EB 93), respectively. Furthermore, findings from previous studies revealed that *Stenotrophomonas* sp. and *Bacillus* sp., which exhibited nematicidal activity in our study, were found to be associated with the PWN community that causes PWD^26,27^. Additionally, bacteria attached to the PWN can be endophytes and may help both the plant and the nematode^28^. We similarly assume that our BEs and nematode-associated bacteria can claim anti-nematode activity. Significant mortality rates were observed in adults compared to the L3/L4 developmental stages. These changes might be due to differences between their levels of resistance and/or mechanisms of adult and juvenile nematodes to combat stress.

To summarize, our work highlights the presence and diversity of BEs in four *Pinus* species located across Korea. The strains belonging to the genera *Bacillus* and *Stenotrophomonas* demonstrated potential activity as biocontrol agents against the PWN. The initial results obtained in this study can be viewed as highly promising. Thus, with the results obtained from this groundwork, further studies involving the identification and characterization of compounds from the CEs might open a new window for exploring and developing agents that could be viable environmentally friendly alternatives to the chemicals used against the PWN.

## Methods

### Collection of tissue samples from four Pinus species

Culturable BEs were isolated from the disease-free members of four *Pinus* species (*P*. *densiflora*, *P*. *koraiensis*, *P*. *rigida*, and *P*. *thunbergii*). The samples were collected from three tissues (needles, stems, and roots). Five sampling sites were selected for *P*. *densiflora* and *P*. *thunbergii* while four sites were selected for *P*. *koraiensis* and *P*. *rigida*. Six trees (replicates) were sampled per site. In total, 18 sites were sampled across Korea during the summer of 2016 (June – August) (Table 1). Young needle samples (2-year old) were collected using sterilized blades. The stem samples were collected from a height of 1 m above the ground level using a sterilized increment borer. Without uprooting the tree, tertiary root samples were collected from a depth of 10 to 25 cm below the soil line. All three tissue samples were collected from the same tree, from its two opposite sides. The samples were stored in clean zip bags, brought immediately to the laboratory, and preserved at 4 °C. The BEs were isolated within 24 h of sampling.

### Isolation of bacterial endophytes

Tissue samples (1 g) were dissected, washed thrice in sterilized reverse osmosis (RO)-treated water, and sonicated for 20 s to extricate the loam and organic substances^29^. Each sample was rinsed in sterile 0.1% Tween 20 for 5 min. Subsequently, the samples were washed with 70% ethanol for 5 min and then rinsed in freshly prepared 4% sodium hypochlorite (NaOCl) solution for 5 min. Finally, the samples were washed ten times with RO water to remove the NaOCl. The samples were then soaked in sterile 10% (w/v) sodium bicarbonate (NaHCO_3_) for 10 min to arrest the growth of fungal endophytes, followed by ten times washing with RO water and drying on a sterilized paper. The surface-sterilized samples were rolled on separate petri dishes containing the media tryptic soy agar (TSA) and nutrient agar (NA) in order to confirm the success of surface sterilization (Thermo Fisher Scientific, Waltham, MA, USA). The petri dishes were incubated at 28 °C and monitored up to 15 d. Each surface-sterilized sample was ground using a sterile motor and pestle and suspended in 10 ml of sterile phosphate buffer (10 mM, pH 7.2). The ground sample suspension was incubated at 28 °C under conditions of shaking at 110 rpm for 30 min to discharge the BEs from the samples into the phosphate buffer. Then, 1 ml of phosphate buffer containing the BEs was serially diluted up to the 10^−5^ dilution using 9 ml of sterile H_2_O. Each dilution (100 µl) was plated in duplicates on sterile TSA and NA supplemented with 0.01% cycloheximide. All the BEs were grown on TSA and NA at 28 °C and observed up to 15 d. The number of bacteria were determined based on the number of colony forming units (CFUs). Each morphologically different bacterial colony was subcultured by streaking on a fresh plate of TSA and NA for further confirmation of the purity of the colonies. Several isolations were carried out by streaking on fresh media. A pure colony per bacterial isolate was differentiated and selected based on its size, form, shape, texture, opaqueness, height, and the morphology of its edges and surface on the respective growth medium. Individual colonies were subcultured for the preparation of glycerol stocks.

### Molecular identification of the bacterial endophytes

Universal primers for the region of 16S rDNA (forward primer 27F: 5′-AGAGTTGATCMTGGCTCAG-3′/reverse primer 1492R: 5′-GGTTACCTTGTTACGACTT-3′) were used for the amplification (Bionics, Seoul, Korea)^30^. The sequences were analyzed using the Geneiuos version v 10.1.3 software (Biomatters, Auckland, New Zealand)^31^. All the sequences were identified using the NCBI BLAST search (https://blast.ncbi.nlm.nih.gov/). A phylogenetic tree was built using the neighbour-joining method and evaluated by bootstrap analysis using the MEGA v 7.0^32^ with 1,000 replications to assess the comparative stability of the branches. All the 16S rDNA sequences of 389 isolates were submitted to NCBI for identification.

### Preparation of ethyl acetate crude extracts

Total metabolites were extracted from the cultures of 44 BEs suspended in tryptic soy broth (TSB) including those of the *Bacillus* sp. (EB 93) and *Stenotrophomonas* sp. (EB 394) using EtOAc^33^. The liquid cultures of *Stenotrophomonas* sp. and *Bacillus* sp. suspended in TSB [12 h growth, 500 µl, optical density (OD) 0.8 at 600 nm] were inoculated into 250 ml TSB in 1,000-ml Erlenmeyer flasks. The cultures were incubated for 6 d at 30 °C and 150 rpm until the two bacterial suspensions reached the stationary phase of growth (OD_600_ = 2.4). An equal volume of EtOAc (250 ml) was added to the broth culture and sonicated for 30 min. The EtOAc mixture was incubated by shaking at 120 rpm overnight and allowing to stand for 2 h. The top clear phase was separated using a separatory funnel. The EtOAc CEs were concentrated in a rotary evaporator at 50 °C. The CEs were dissolved in 3% dimethyl sulfoxide (DMSO) solution prepared in 100% methanol.

### Isolation of eggs, L3/L4 juvenile and adult stages of the nematodes

The PWNs were procured from the Korea Forest Research Institute (Seoul, Korea), fed on the fungal mycelia of *Botrytis cinerea*, grown for 8 d at 25 °C in the dark^34^ and collected using the Baermann funnel technique^35^ (Fig. S4).

The eight-day-old adult female nematodes were collected in 96-well polystyrene plates and petri dishes. All the female nematodes were observed up to 12 h until many quality eggs were laid at room temperature under the dark. Eggs (*c*. 100) were washed and counted and their quality was confirmed by evaluating the movement of the egg embryo. Subsequently, 10 µl of 0.1 mg CE was added to 90 µl of water holding 100 eggs. The final concentration of the CE was 1,000 ppm. The eggs were treated with CEs of the 44 BEs and monitored using water control (control 1), solvent control (control 2: 3% DMSO in methanol), and positive control (control 3: abamectin; Sigma-Aldrich, Saint-Louis, MO, USA). All the readings were taken after 24 h. The hatching rate (%) was calculated as follows:

$${\rm{Hatching}}\,{\rm{rate}}( \% )=[{\rm{juveniles}}/({\rm{eggs}}+{\rm{juveniles}})]\times 100$$

The PWNs were fed with *B*. *cinerea* and grown up to 5 d (L3/L4 stages) and 9 d (adult stage). Subsequently, they were collected and washed in sterile water (Supplementary Fig. S4). The length of the PWN at different stages was measured using the ImageJ software (https://imagej.nih.gov/ij/), based on which, the two stages were differentiated. The average length was 208 and 773 µm for L3/L4 and adult stage nematodes, respectively. Nematodes at both the stages were counted and separated in 96-well polystyrene plates. The experiment was carried out by adding 10 µl of 0.1 mg CE to 90 µl of water containing 100 nematodes, resulting in a final concentration of 1,000 ppm. The nematodes were monitored at 12, 24, 48 and 72 h after treatment. Mortality of the nematodes was calculated according to the Schneider-Orelli formula as follows^36^:

$$\begin{array}{ccc}{\rm{Corrected}}\,{\rm{mortality}}\,( \% ) & = & [{\rm{mortality}}\,{\rm{in}}\,{\rm{treatment}}\,( \% )-{\rm{mortality}}\,{\rm{in}}\,{\rm{negative}}\,{\rm{control}}\\  &  & ( \% )]/(1-{\rm{mortality}}\,{\rm{in}}\,{\rm{negative}}\,{\rm{control}})\end{array}$$

### Data analysis

The relative proportion (percentage) was calculated as the number of isolates belonging to a species or phylum, divided by the total isolates recovered from all the tissues. Species richness (S) among the BEs was estimated based on the specific sampling site. S is the number of species recovered from a specific region or tree sample. The Shannon’s diversity index (H) was analyzed using the following equation: H = −Σ (P*i* × In P*i*), where P*i* is the relative proportion of species in a specific sampling site or particular tissue (*i*)^37^. After performing the one-way analysis of variance (ANOVA) followed by the Tukey’s test to calculate the significant differences among data at any level (***P < 0.001) using the software GraphPad Prism 7 (La Jolla, CA, USA).

## Supplementary information

Supplementary Information

Supplementary Movie S1

Supplementary Movie S2

Supplementary Movie S3

Supplementary Movie S4

Supplementary Movie S5

Supplementary Movie S6

Supplementary Movie S7

Supplementary Movie S8

Supplementary Movie S9

Supplementary Movie S10

Supplementary Movie S11

Supplementary Movie S12

Supplementary Movie S13

Supplementary Movie S14

Supplementary Movie S15
