# Supplementary material for: Phylogenetic characterization of bacterial endophytes from four Pinus species and their nematicidal activity against the pine wood nematode
Source: Sci Rep. 2019 Aug 28;9:12457. doi: 10.1038/s41598-019-48745-6 (PMC6713757; doi:10.1038/s41598-019-48745-6)
Supplement: Supplementary file 1 — Supplementary Information [file 41598_2019_48745_MOESM1_ESM.pdf]

Supplementary information

Phylogenetic characterization of bacterial endophytes from four *Pinus* species and their nematicidal activity against the pine wood nematode

Lakshmi Narayanan Ponpandian[1], Soon Ok Rim[1], Gnanendra Shanmugam[1], Junhyun Jeon[1], Young-Hwan Park[2], Sun-Keun Lee[3], Hanhong Bae[1*]

[1] Department of Biotechnology, Yeungnam University, Gyeongsan, Gyeongbuk 38541, Republic of Korea

[2] Nakdonggang National Institute of Biological Resources, Sangju 37242, Republic of Korea

[3] Division of Forest Insect Pests and Diseases, National Institute of Forest Science, Seoul 02455, Republic of Korea

[*]**Correspondence:** Hanhong Bae

Email: hanhongbae@ynu.ac.kr

Phone: 8253-810-3031 (office), Fax: 8253-810-4769,

Running title: Pine bacterial endophyte and nematicidal activity

Supplementary Table S1. List of the bacterial endophytes isolated from four *Pinus* species across 18 sampling sites in Korea. Bacterial endophytes were identified based on the 16S rDNA sequence.

(EB) Endophytic bacteria number	Isolates	Identity	Accession no.
1	*Achromobacter marplatensis*	99%	MH119942
2	*Achromobacter pestifer*	99%	MH119943
3	*Achromobacter piechaudii*	99%	MH130290
4	*Achromobacter* sp.	99%	MH130291
5	*Achromobacter* sp.	99%	MH130292
6	*Achromobacter* sp.	99%	MH130293
7	*Achromobacter Spanius*	99%	MH130294
8	*Achromobacter xylosoxidans*	99%	MH130295
9	*Acidovorax* sp.	99%	MH130296
10	*Acidovorax* sp.	99%	MH130297
11	*Acinetobacter calcoaceticus*	100%	MH130298
12	*Acinetobacter lwoffii*	99%	MH130299
13	*Acinetobacter* sp.	100%	MH130300
14	*Acinetobacter radioresistens*	99%	MH130301
15	*Acinetobacter* sp.	98%	MH130302
16	*Paenibacillus* sp.	99%	MH130303
17	*Chryseomicrobium imtechense*	99%	MH130304
18	*Streptomyces werraensis*	99%	MH130305
19	*Acinetobacter* sp.	99%	MH130306
20	*Acinetobacter* sp.	100%	MH130307
21	*Actinotalea fermentans*	99%	MH130308
22	*Actinotalea* sp.	99%	MH130309
23	*Agrobacterium* sp.	99%	MH130310
24	*Agrobacterium* sp.	99%	MH130311
25	*Microbacterium* sp.	100%	MH130312
26	*Agrococcus citreus*	99%	MH130313
27	*Aeromicrobium* sp.	98%	MH130314
28	*Agrococcus* sp.	99%	MH130315
29	*Arthrobacter scleromae*	99%	MH130316
30	*Serratia rubidaea*	91%	MH142603
31	*Nocardioides exalbidus*	99%	MH130317
32	*Agromyces* sp.	99%	MH130318
33	*Alcaligenes* sp.	99%	MH130319
34	*Brevundimonas* sp.	100%	MH130320
35	*Bacillus cibi*	99%	MH130321
36	*Arthrobacter arilaitensis*	100%	MH130322

37	*Arthrobacter oryzae*	99%	MH130323
38	*Arthrobacter oxydans*	100%	MH130324
39	*Arthrobacter pascens*	100%	MH130325
40	*Arthrobacter phenanthrenivorans*	99%	MH130326
41	*Arthrobacter* sp.	99%	MH130327
42	*Arthrobacter* sp.	99%	MH130328
43	*Arthrobacter* sp.	99%	MH130329
44	*Bacillus mycoides*	99%	MH130330
45	*Arthrobacter* sp.	99%	MH130331
46	*Arthrobacter* sp.	99%	MH130332
47	*Arthrobacter* sp.	100%	MH130333
48	*Arthrobacter* sp.	100%	MH130334
49	*Arthrobacter* sp.	99%	MH130335
50	*Acinetobacter* sp.	99%	MH130336
51	*Arthrobacter* sp.	99%	MH130337
52	*Arthrobacter* sp.	99%	MH130338
53	*Arthrobacter* sp.	100%	MH130339
54	*Arthrobacter* sp.	99%	MH142604
55	*Bacillus aerophilus*	99%	MH130340
56	*Bacillus altitudinis*	100%	MH130341
57	*Bacillus amyloliquefacien*	100%	MH130342
58	*Paenarthrobacter* sp.	98%	MH142597
59	*Bacillus aquimaris*	100%	MH130343
60	*Bacillus aryabhattai*	99%	MH130344
61	*Brevundimonas vesicularis*	99%	MH130345
62	*Bacillus cereus*	100%	MH130346
63	*Bacillus circulans*	99%	MH130347
64	*Bacillus ginsengisoli*	99%	MH130348
65	*Arthrobacter* sp.	99%	MH141437
66	*Bacillus huizhouensis*	100%	MH130349
67	*Bacillus idriensis*	99%	MH130350
68	*Bacillus infantis*	99%	MH130351
69	*Arthrobacter* sp.	99%	MH130352
70	*Bacillus korlensis*	98%	MH130353
71	*Bacillus megaterium*	99%	MH130354
72	*Bacillus methylotrophicus*	99%	MH130355
73	*Microbacterium* sp.	99%	MH130356
74	*Arthrobacter* sp.	99%	MH130357
75	*Bacillus* sp.	99%	MH130358
76	*Bacillus oryzaecorticis*	99%	MH130359
77	*Bacillus pumilus*	100%	MH130360
78	*Bacillus simplex*	100%	MH130361
79	*Bacillus* sp.	99%	MH130362

80	*Microbacterium aoyamense*	99%	MH130363
81	*Bacillus acidiceler*	99%	MH130364
82	*Sanguibacter keddieii*	100%	MH130365
83	*Bacillus* sp.	99%	MH130366
84	*Bacillus* sp.	99%	MH130367
85	*Bacillus* sp.	100%	MH130368
86	*Bacillus arbutinivorans*	99%	MH130369
87	*Bacillus velezensis*	99%	MH130370
88	*Bacillus stratoSphericus*	99%	MH130371
89	*Bacillus arbutinivorans*	99%	MH130372
90	*Bacillus* sp.	99%	MH130373
91	*Agrobacterium tumefaciens*	100%	MH130374
92	*Bacillus* sp.	99%	MH130375
93	*Bacillus* sp.	100%	MH107110
94	*Bacillus* sp.	100%	MH130376
95	*Bacillus firmus*	99%	MH130377
96	*Bacillus thuringiensis*	99%	MH130378
97	*Bacillus toyonensis*	99%	MH130379
98	*Bacillus velezensis*	99%	MH130380
99	*Bacillus indicus*	99%	MH141438
100	*Bacillus weihenstephanensis*	99%	MH130381
101	*Microbacterium* sp.	99%	MH127540
102	*Blastococcus* sp.	99%	MH127541
103	*Micrococcus luteus*	99%	MH142592
104	*Brachybacterium paraconglomeratum*	99%	MH127542
105	*Bacillus licheniformis*	99%	MH127543
106	*Microbacterium testaceum*	99%	MH142601
107	*Brevundimonas bullata*	99%	MH127544
108	*Brevundimonas kwangchunensis*	99%	MH127545
109	*Brevundimonas staleyi*	99%	MH127546
110	*Bacillus marisflavi*	99%	MH127547
111	*Bacillus marisflavi*	99%	MH142593
112	*Buttiauxella agrestis*	99%	MH127548
113	*Cedecea neteri*	99%	MH127549
114	*Cellulomonas fimi*	99%	MH127550
115	*Bacillus marisflavi*	99%	MH127551
116	*Bacillus megaterium*	92%	MH142599
117	*Chryseobacterium* sp.	99%	MH127552
118	*Bacillus megaterium*	97%	MH142594
119	*Chryseomicrobium* sp.	99%	MH127553
120	*Citrobacter braakii*	96%	MH142600
121	*Citrobacter freundii*	99%	MH127554
122	*Micrococcus yunnanensis*	99%	MH127555
123	*Comamonas testosteroni*	99%	MH127556

124	*Corynebacterium aquaticum*	99%	MH127557
125	*Curtobacterium* sp.	99%	MH127558
126	*Curtobacterium* sp.	100%	MH127559
127	*Bacillus megaterium*	98%	MH127560
128	*Dermacoccus nishinomiyaensis*	99%	MH127561
129	*Dermacoccus* sp.	100%	MH127562
130	*Microbacterium oxydans*	99%	MH142595
131	*Dietzia maris*	99%	MH127563
132	*Bacillus megaterium*	99%	MH127564
133	*Bacillus megaterium*	99%	MH127565
134	*Ensifer adhaerens*	99%	MH127566
135	*Enterobacter aerogenes*	99%	MH127567
136	*Enterobacter amnigenus*	99%	MH127568
137	*Enterobacter asburiae*	99%	MH127569
138	*Enterobacter ludwigii*	99%	MH127570
139	*Enterobacter* sp.	99%	MH127571
140	*Enterobacter* sp.	98%	MH127572
141	*Enterobacter* sp.	99%	MH127573
142	*Enterobacter* sp.	99%	MH127574
143	*Enterobacter* sp.	99%	MH127575
144	*Enterobacter* sp.	99%	MH127576
145	*Enterobacter* sp.	99%	MH127577
146	*Bacillus megaterium*	99%	MH127578
147	*Enterobacter* sp.	99%	MH127579
148	*Pantoea agglomerans*	99%	MH127580
149	*Erwinia billingiae*	99%	MH127581
150	*Ewingella americana*	99%	MH127582
151	*Exiguobacterium acetylicum*	99%	MH127583
152	*Exiguobacterium aurantiacum*	99%	MH127584
153	*Exiguobacterium mexicanum*	99%	MH127585
154	*Bacillus megaterium*	99%	MH127586
155	*Microbacterium* sp.	99%	MH127587
156	*Bacillus megaterium*	99%	MH127588
157	*Bacillus megaterium*	99%	MH127589
158	*Flavobacterium ummariense*	97%	MH127590
159	*Flexivirga* sp.	99%	MH127591
160	*Staphylococcus hominis*	99%	MH127592
161	*Gordonia alkanivorans*	99%	MH127593
162	*Gordonia terrae*	99%	MH127594
163	*Bosea* sp.	99%	MH127595
164	*Isoptericola nanjingensis*	99%	MH127596
165	*Microbacterium hominis*	99%	MH127597
166	*Bacillus megaterium*	99%	MH127598

167	*Kluyvera* sp.	99%	MH127599
168	*Knoellia flava*	99%	MH127600
169	*Micrococcus* sp.	97%	MH142596
170	*Kocuria* sp.	100%	MH127601
171	*Kocuria carniphila*	99%	MH127602
172	*Kocuria rhizophila*	99%	MH127603
173	*Kocuria palustris*	99%	MH127604
174	*Bacillus subtilis*	99%	MH127605
175	*Bacillus huizhouensis*	99%	MH127606
176	*Kocuria rosea*	99%	MH127607
177	*Kocuria* sp.	100%	MH127608
178	*Kocuria* sp.	98%	MH127609
179	*Leclercia adecarboxylata*	99%	MH127610
180	*Lelliottia amnigena*	99%	MH127611
181	*Lelliottia* sp.	98%	MH127612
182	*Bacillus megaterium*	99%	MH127613
183	*Lysinibacillus macroides*	99%	MH127614
184	*Lysinibacillus massiliensis*	99%	MH127615
185	*Lysinibacillus Sphaericus*	99%	MH127616
186	*Lysinibacillus varians*	99%	MH127617
187	*Bacillus megaterium*	100%	MH127618
188	*Bacillus megaterium*	100%	MH127619
189	*Methylobacterium brachiatum*	98%	MH127620
190	*Bacillus meqaterium*	99%	MH127621
191	*Bacillus meqaterium*	99%	MH127622
192	*Bacillus meqaterium*	99%	MH127623
193	*Microbacterium* sp.	99%	MH127624
194	*Microbacterium foliorum*	99%	MH127625
195	*Bacillus subtilis*	99%	MH127626
196	*Microbacterium hydrocarbonoxydans*	99%	MH127627
197	*Microbacterium hydrothermale*	99%	MH127628
198	*Microbacterium imperiale*	99%	MH127629
199	*Bacillus simplex*	99%	MH127630
200	*Bacillus pumilus*	100%	MH127631
201	*Bacillus pseudomycoides*	99%	MH127661
202	*Bacillus pumilus*	99%	MH142609
203	*Microbacterium oxydans*	99%	MH127662
204	*Microbacterium paraoxydans*	99%	MH127663
205	*Microbacterium phylloSphaerae*	99%	MH127664
206	*Microbacterium radiodurans*	99%	MH127665
207	*Microbacterium saccharophilum*	99%	MH127666
208	*Microbacterium* sp.	99%	MH127667
209	*Microbacterium* sp.	100%	MH127668

210	*Microbacterium* sp.	99%	MH127669
211	*Microbacterium* sp.	99%	MH127670
212	*Microbacterium* sp.	99%	MH127671
213	*Microbacterium* sp.	99%	MH127672
214	*Microbacterium* sp.	99%	MH127673
215	*Microbacterium* sp.	99%	MH127674
216	*Microbacterium* sp.	100%	MH127675
217	*Bacillus pumilus*	99%	MH127676
218	*Microbacterium* sp.	100%	MH127677
219	*Microbacterium* sp.	99%	MH127678
220	*Microbacterium* sp.	99%	MH127679
221	*Microbacterium* sp.	99%	MH127680
222	*Microbacterium* sp.	99%	MH127681
223	*Microbacterium* sp.	99%	MH127682
224	*Raoultella ornithinolytica*	99%	MH127683
225	*Microbacterium* sp.	99%	MH127684
226	*Bacillus idriensis*	99%	MH127685
227	*Microbacterium* sp.	100%	MH127686
228	*Bacillus* sp.	96%	MH142610
229	*Microbacterium* sp.	99%	MH127687
230	*Microbacterium testaceum*	99%	MH127688
231	*Micrococcus aloeverae*	99%	MH127689
232	*Bacillus* sp.	98%	MH127690
233	*Micrococcus* sp.	98%	MH127691
234	*Micrococcus* sp.	99%	MH127692
235	*Micrococcus* sp.	99%	MH127693
236	*Micrococcus* sp.	99%	MH127694
237	*Micrococcus* sp.	99%	MH127695
238	*Micrococcus* sp.	100%	MH127696
239	*Micrococcus* sp.	100%	MH127697
240	*Micrococcus* sp.	99%	MH127698
241	*Micrococcus yunnanensis*	99%	MH127699
242	*Microvirga* sp.	99%	MH127700
243	*Bacillus* sp.	99%	MH127701
244	*Staphylococcus hominis*	98%	MH142598
245	*Naumannella* sp.	99%	MH127702
246	*Bacillus* sp.	99%	MH127703
247	*Nocardioides* sp.	99%	MH127704
248	*Bacillus* sp.	99%	MH127705
249	*Bacillus circulans*	97%	MH127706
250	*Bacillus* sp.	99%	MH127707
251	*Paenibacillus amylolyticus*	100%	MH127708
252	*Bacillus* sp.	99%	MH127709

253	*Bacillus* sp.	99%	MH127710
254	*Paenibacillus cineris*	99%	MH127711
255	*Bacillus cereus*	100%	MH127712
256	*Paenibacillus polymyxa*	99%	MH127713
257	*Bacillus cereus*	99%	MH127714
258	*Bacillus* sp.	99%	MH127715
259	*Paenibacillus* sp.	99%	MH127716
260	*Paenibacillus* sp.	99%	MH127717
261	*Paenibacillus Sputi*	98%	MH127718
262	*Pantoea agglomerans*	99%	MH127719
263	*Pantoea* sp.	99%	MH127720
264	*Pantoea* sp.	99%	MH127721
265	*Bacillus aryabhattai*	99%	MH127722
266	*Pantoea vagans*	99%	MH127723
267	*Paracoccus* sp.	99%	MH127724
268	*Paracoccus* sp.	99%	MH127725
269	*Bacillus aryabhattai*	98%	MH127726
270	*Sporosarcina luteola*	99%	MH127727
271	*Bacillus amyloliquefaciens*	99%	MH127728
272	*Bacillus amyloliquefaciens*	99%	MH127729
273	*Bacillus* sp.	99%	MH127730
274	*Pseudomonas alcaliphila*	100%	MH127731
275	*Pseudomonas extremorientalis*	99%	MH127732
276	*Pseudomonas fluorescens*	100%	MH127733
277	*Pseudomonas fulva*	100%	MH127734
278	*Sporosarcina koreensis*	99%	MH142611
279	*Pseudomonas japonica*	99%	MH127735
280	*Pseudomonas koreensis*	99%	MH127736
281	*Pseudomonas lurida*	99%	MH127737
282	*Pseudomonas mendocina*	99%	MH127738
283	*Bacillus* sp.	99%	MH127739
284	*Pseudomonas moraviensis*	99%	MH142602
285	*Pseudomonas protegens*	100%	MH127740
286	*Pseudomonas putida*	99%	MH127741
287	*Bacillus amyloliquefaciens*	99%	MH127742
288	*Acinetobacter lwoffii*	99%	MH127743
289	*Pseudomonas* sp.	99%	MH127744
290	*Pseudomonas* sp.	99%	MH127745
291	*Pseudomonas* sp.	99%	MH127746
292	*Bacillus* sp.	99%	MH127747
293	*Pseudomonas* sp.	99%	MH127748
294	*Pseudomonas* sp.	99%	MH127749
295	*Bacillus Sporothermodurans*	99%	MH127750

296	*Bacillus subterraneus*	99%	MH127751
297	*Acinetobacter lwoffii*	99%	MH127752
298	*Pseudomonas* sp.	99%	MH127753
299	*Bacillus taiwanensis*	99%	MH127754
300	*Pseudomonas* sp.	99%	MH127755
301	*Pseudomonas* sp.	99%	MH127756
302	*Bacillus thioparans*	99%	MH127757
303	*Microbacterium testaceum*	99%	MH127758
304	*Pseudomonas* sp.	100%	MH127759
305	*Pseudomonas* sp.	99%	MH127760
306	*Pseudomonas* sp.	99%	MH127761
307	*Pseudomonas* sp.	99%	MH127762
308	*Pseudomonas* sp.	99%	MH127763
309	*Pseudomonas* sp.	100%	MH127764
310	*Bacillus thuringiensis*	97%	MH142605
311	*Pseudomonas vancouverensis*	99%	MH127765
312	*Pseudoxanthomonas japonensis*	100%	MH127766
313	*Psychrobacillus psychrodurans*	99%	MH127767
314	*Psychrobacillus* sp.	99%	MH127768
315	*Rahnella aquatilis*	99%	MH127769
316	*Microbacterium oleivorans*	99%	MH127770
317	*Rahnella* sp.	99%	MH127771
318	*Rahnella* sp.	99%	MH127772
319	*Raoultella ornithinolytica*	100%	MH127773
320	*Raoultella planticola*	99%	MH127774
321	*Bacillus aerophilus*	100%	MH127775
322	*Staphylococcus pettenkoferi*	99%	MH142606
323	*Staphylococcus pettenkoferi*	99%	MH127776
324	*Bacillus aerophilus*	99%	MH127777
325	*Okibacterium fritillariae*	99%	MH127778
326	*Rahnella* sp.	95%	MH142607
327	*Rhodococcus ruber*	99%	MH127779
328	*Corynebacterium simulans*	99%	MH127780
329	*Salmonella* sp.	99%	MH127781
330	*Corynebacterium singulare*	99%	MH127782
331	*Serratia fonticola*	100%	MH127783
332	*Staphylococcus hominis*	99%	MH127784
333	*Serratia marcescens*	99%	MH127785
334	*Serratia plymuthica*	100%	MH127786
335	*Staphylococcus pettenkoferi*	100%	MH127787
336	*Pseudomonas* sp.	99%	MH127788
337	*Curtobacterium flaccumfaciens*	97%	MH156217
338	*Serratia* sp.	99%	MH127789

339	*Serratia* sp.	99%	MH127790
340	*Serratia* sp.	100%	MH127791
341	*Serratia* sp.	99%	MH127792
342	*Microbacterium* sp.	99%	MH127793
343	*Curtobacterium flaccumfaciens*	98%	MH127794
344	*Isoptericola variabilis*	99%	MH127795
345	*Curtobacterium flaccumfaciens*	99%	MH127796
346	*Curtobacterium flaccumfaciens*	99%	MH127797
347	*Pseudomonas* sp.	95%	MH142608
348	*Curtobacterium herbarum*	98%	MH156218
349	*Sporosarcina aquimarina*	99%	MH127798
350	*Pseudomonas psychrotolerans*	99%	MH127799
351	*Curtobacterium herbarum*	99%	MH127800
352	*Staphylococcus capitis*	100%	MH127801
353	*Staphylococcus cohnii*	99%	MH127802
354	*Staphylococcus devriesei*	99%	MH127803
355	*Curtobacterium* sp.	98%	MH156219
356	*Staphylococcus hominis*	99%	MH127804
357	*Microbacterium trichothecenolyticum*	99%	MH156220
358	*Micrococcus* sp.	99%	MH127805
359	*Staphylococcus* sp.	100%	MH127806
360	*Staphylococcus* sp.	100%	MH127807
361	*Stenotrophomonas maltophilia*	99%	MH127808
362	*Stenotrophomonas rhizophila*	100%	MH127809
363	*Stenotrophomonas* sp.	99%	MH127810
364	*Stenotrophomonas* sp.	99%	MH107111
365	*Curtobacterium* sp.	99%	MH127811
366	*Staphylococcus saprophyticus*	99%	MH127812
367	*Pseudomonas libanensis*	99%	MH127813
368	*Pseudomonas azotoformans*	99%	MH127814
369	*Fictibacillus nanhaiensis*	99%	MH127815
370	*Pantoea ananatis*	99%	MH127816
371	*Paenibacillus shenyangensis*	99%	MH127817
372	*Paenibacillus polymyxa*	99%	MH127818
373	*Paenibacillus massiliensis*	99%	MH127819
374	*Staphylococcus xylosus*	99%	MH127820
375	*Luteimonas aestuarii*	99%	MH127821
376	*Luteimonas aestuarii*	99%	MH127822
377	*Massilia timonae*	99%	MH127823
378	*Microbacterium dextranolyticum*	98%	MH156221
379	*Microbacterium dextranolyticum*	97%	MH156222
380	*Microbacterium flavescens*	98%	MH156223
381	*Microbacterium hatanonis*	99%	MH127824

382	*Microbacterium laevaniformans*	99%	MH127825
383	*Microbacterium laevaniformans*	99%	MH127826
384	*Microbacterium laevaniformans*	99%	MH127827
385	*Microbacterium paludicola*	99%	MH127828
386	*Microbacterium* sp.	96%	MH156224
387	*Microbacterium* sp.	98%	MH127829
388	*Microbacterium* sp.	99%	MH127830
389	*Paenibacillus durus*	93%	MH127831

Supplementary Table S2. Number of genera found in different tissues and *Pinus* species.

		Number of genera in specific tissue			
	Genus	Needle	Stem	Root	*Pinus* species
Alpha-proteobacteria	*Agrobacterium*			4	*P. rigida*
	Bosea		1		*P. rigida*
	Ensifer		1		*P. rigida*
	Microvirga			1	*P. rigida*
	Paracoccus		2		*P. thunbergii*
Beta-proteobacteria	*Alcaligenes*	1			*P. rigida*
	Comamonas	2			*P. rigida*
	Massilia	1			*P. rigida*
Gamma-proteobacteria	*Buttiauxella*	1			*P. rigida*
	Cedecea	1			*P. rigida*
	Citrobacter	2			*P. rigida*
	Erwinia	2			*P. rigida*
	Lelliottia	2			*P. rigida*
	Luteimonas	2			*P. rigida*
	Pseudoxanthomonas	1			*P. koraiensis*
	Salmonella	1			*P. rigida*
Actinobacteria	*Aeromicrobium*			1	*P. thunbergii*
	Knoellia			2	*P. rigida*
	Kocuria			27	*P. densiflora*
	Naumannella			1	*P. rigida*
Firmicutes	*Chryseomicrobium*		2		*P. rigida*
	Fictibacillus		2		*P. rigida*

Supplementary Table S3. Bacterial endophyte sampling overview: the geographic location and meta-data of 18 sampling sites across Korea.

Sampling sites	Latitude	Longitude	Altitude (m)	Mean rainfall (mm)	Mean temperature (℃)	pH
Pr 1	37.0744580	127.1119200	70	284	25.5	5.9
Pr 2	36.8906971	126.4491716	60	295.6	25.2	6.1
Pr 3	35.5521138	127.0191565	240	205.1	27.1	5.3
Pr 4	36.6061179	129.0885334	250	323.9	23	6.1
Pd 1	35.5565492	126.9866204	310	205.1	27.1	5.3
Pd 2	33.3737599	126.4716048	1030	98.6	27.4	5.3
Pd 3	37.5098629	128.1603840	540	360.1	25.7	5.7
Pd 4	37.0054026	128.9415950	700	416.5	25.9	6.1
Pd 5	37.6118298	127.4436801	300	385	25.4	6.0
Pt 1	36.1205372	129.4117678	1010	113.2	25.9	5.6
Pt 2	33.4729632	126.5005679	30	98.6	27.4	5.3
Pt 3	36.8766539	126.3867902	40	295.6	25.2	6.1
Pt 4	37.9165560	128.8103605	10	424.2	24.1	5.6
Pt 5	34.7087622	127.7600016	30	157.8	25.3	6.1
Pk 1	37.8073347	127.9294259	470	429.4	25.3	5.8
Pk 2	37.1883303	127.9838292	320	419.7	24.1	5.8
Pk 3	37.9058734	127.9312447	250	617.1	25.2	6.2
Pk 4	37.9654135	127.1123213	120	366.3	25.1	5.7

Supplementary Table S4. Bacterial endophyte sampling overview: the 18 sampling sites across Korea and their meta-data.

Sampling sites	Organic matter (g / kg)	Available phosphate (mg / kg)	K	Ca	Mg	Electrical conductivity	Available silicic acid (mg / kg)
			($cmol^+$ / kg)				
Pr 1	20.8	252.4	0.7	4.2	1.7	0.4	123.2
Pr 2	22.3	336.6	1.1	6.6	2.4	1.1	75.9
Pr 3	30.4	892.7	1.0	5.8	1.9	0.2	7.9
Pr 4	21.4	251.2	0.8	7.4	2.8	0.1	96.2
Pd 1	30.4	892.7	1.0	5.8	1.9	0.2	7.9
Pd 2	50.6	591.7	1.2	4.6	1.8	1.7	0.0
Pd 3	30.2	547.2	0.8	3.7	1.1	0.8	48.9
Pd 4	27.0	571.7	0.9	5.5	1.5	0.4	11.2
Pd 5	24.4	299.6	0.5	5.1	1.1	0.6	248.8
Pt 1	22.1	153.3	0.5	6.9	2.5	0.5	116.3
Pt 2	55.7	464.0	11.7	5.5	2.0	38.7	0.1
Pt 3	22.3	336.6	1.1	6.6	2.4	1.1	75.9
Pt 4	25.3	667.1	0.6	3.3	1.2	0.3	49.6
Pt 5	31.9	526.2	0.8	6.4	1.6	0.8	81.0
Pk 1	21.7	217.9	0.4	4.2	1.1	0.4	138.4
Pk 2	25.5	320.5	0.7	5.6	1.8	0.2	70.8
Pk 3	30.2	302.6	0.6	4.1	1.6	0.6	30.1
Pk 4	21.3	237.3	0.9	3.9	0.9	0.4	72.4

Supplementary Fig. S1 Scatter plots describing the relationships between the meta-data and Shannon's index of the sampling regions.

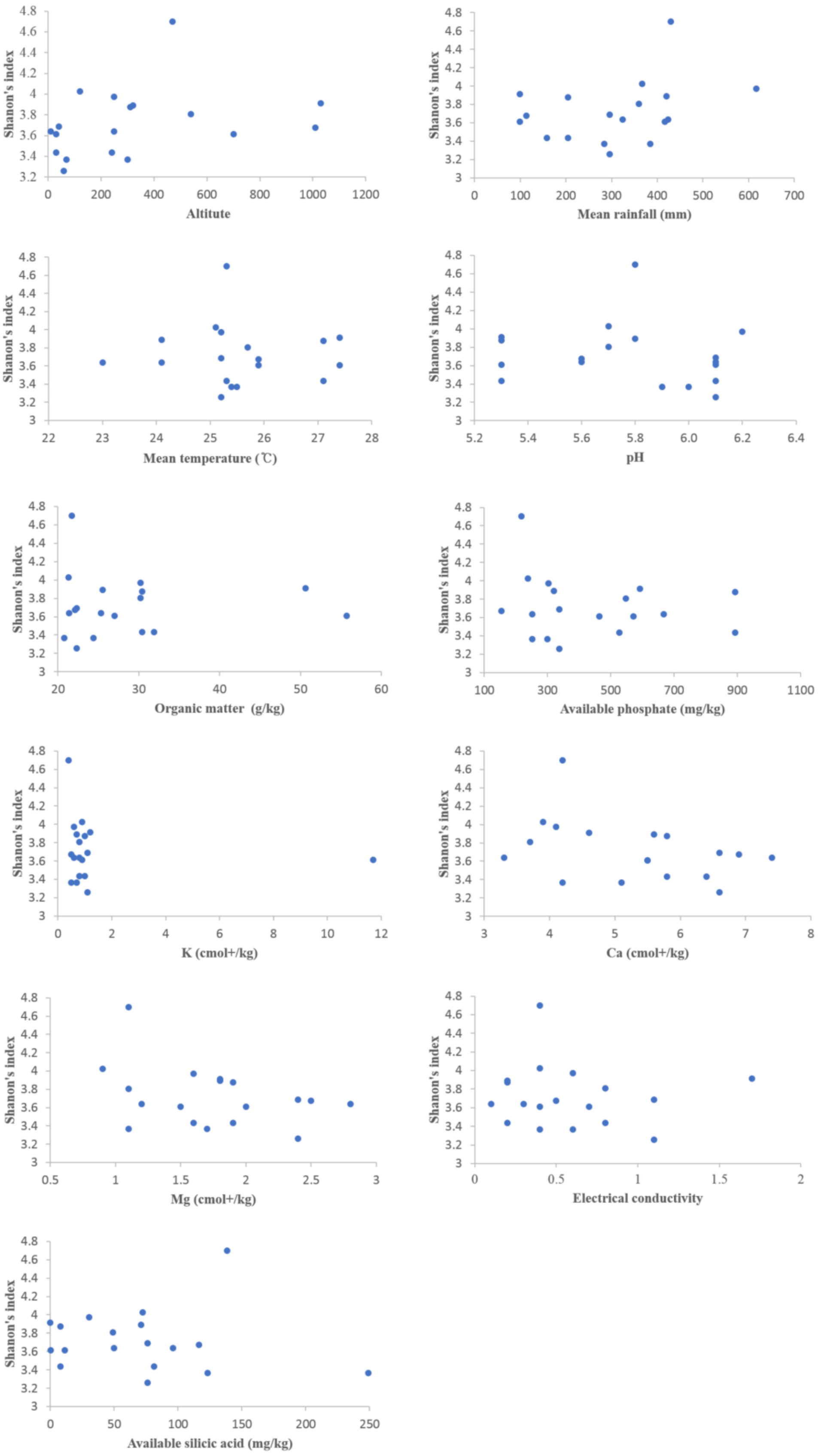

Supplementary Fig. S2. Scatter plots describing the relationships between meta-data and species richness of the sampling regions.

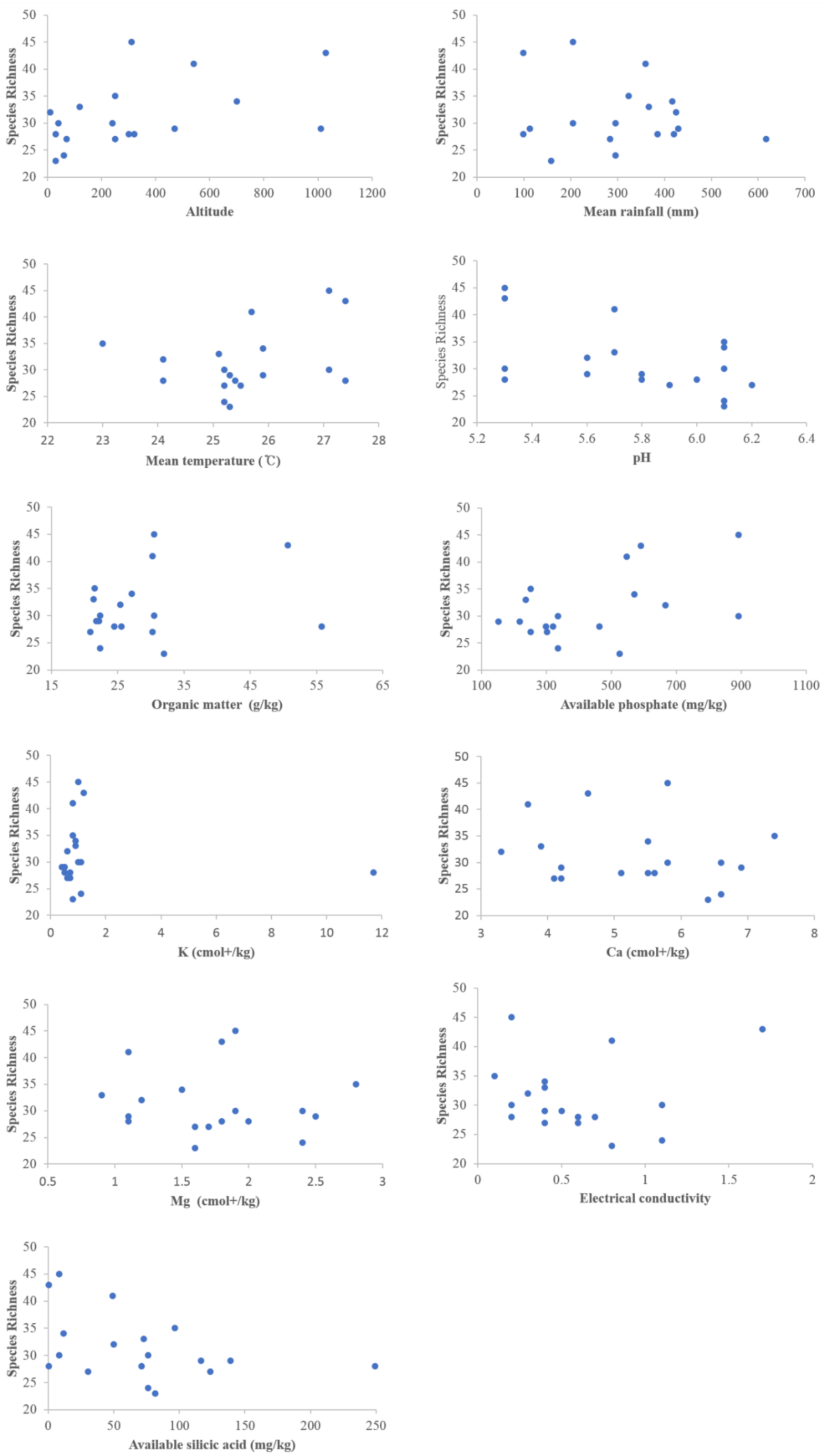

Supplementary Fig. S3. Treatment of ethyl acetate extracts of the 42 bacterial endophytes at 1,000 ppm concentration on the eggs, and L3/L4 and adult stages of *Bursaphelenchus xylophilus*. The nematodes and eggs were monitored at different time intervals (0 h, 12 h, 24 h, 48 h, and 72 h). All graphs were plotted based on the 24 h result. (A) Percentage of egg hatching rate. (B) Percentage of nematicidal effect on the L3/ L4 stage of nematodes. (C) Percentage of nematicidal effect on the adult stage of nematodes. Data are represented as the means ± standard error of means of three trials with eighteen replications. ***$P < 0.001$ *vs*. control 2. Average size of the L3/L4 nematodes is 208 µm and that of the adult stage nematode is 773 µm. Scale bars = 100 µm.

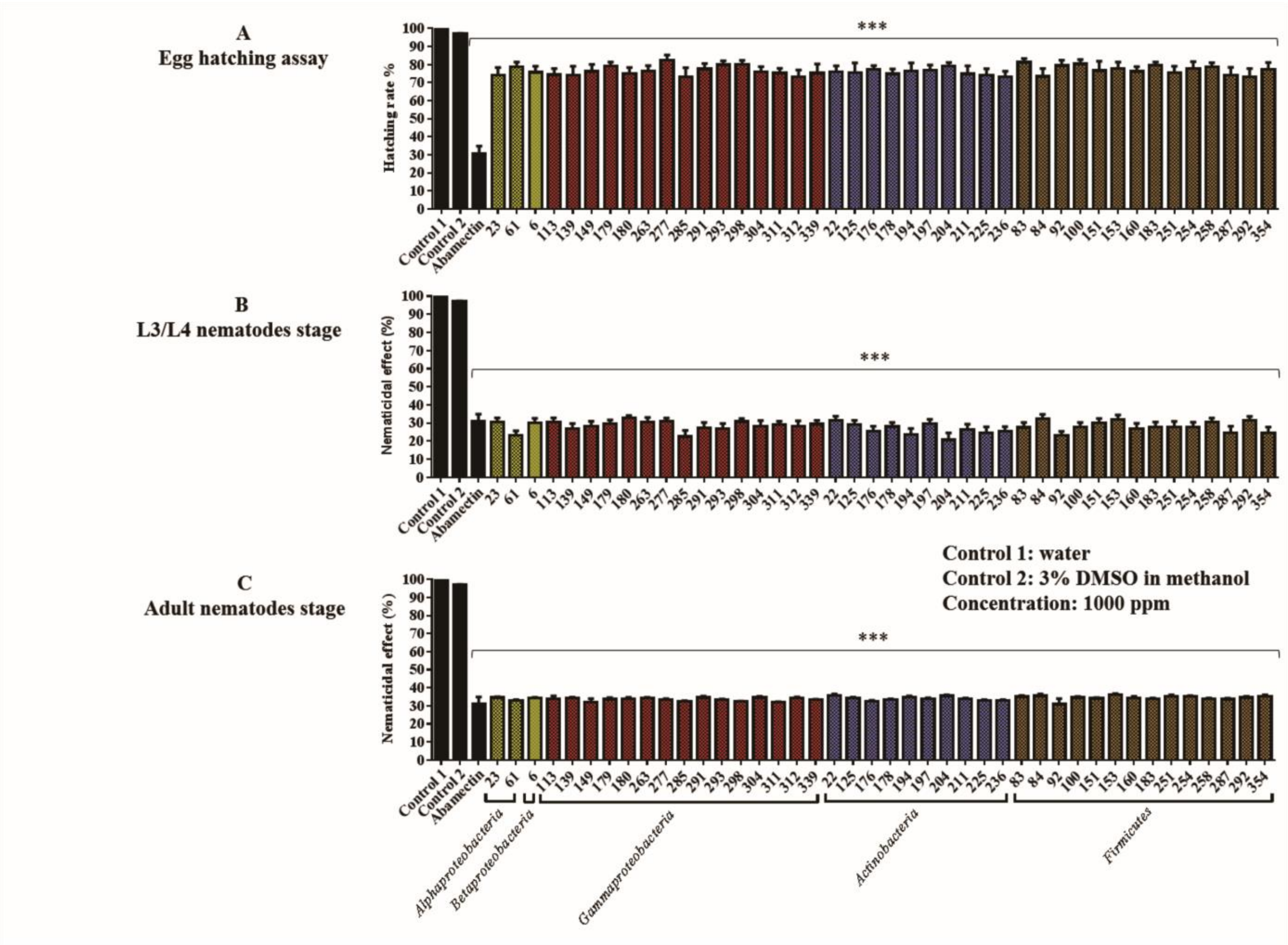

Supplementary Fig. S4. Isolation of eggs and different developmental stages of the nematodes.

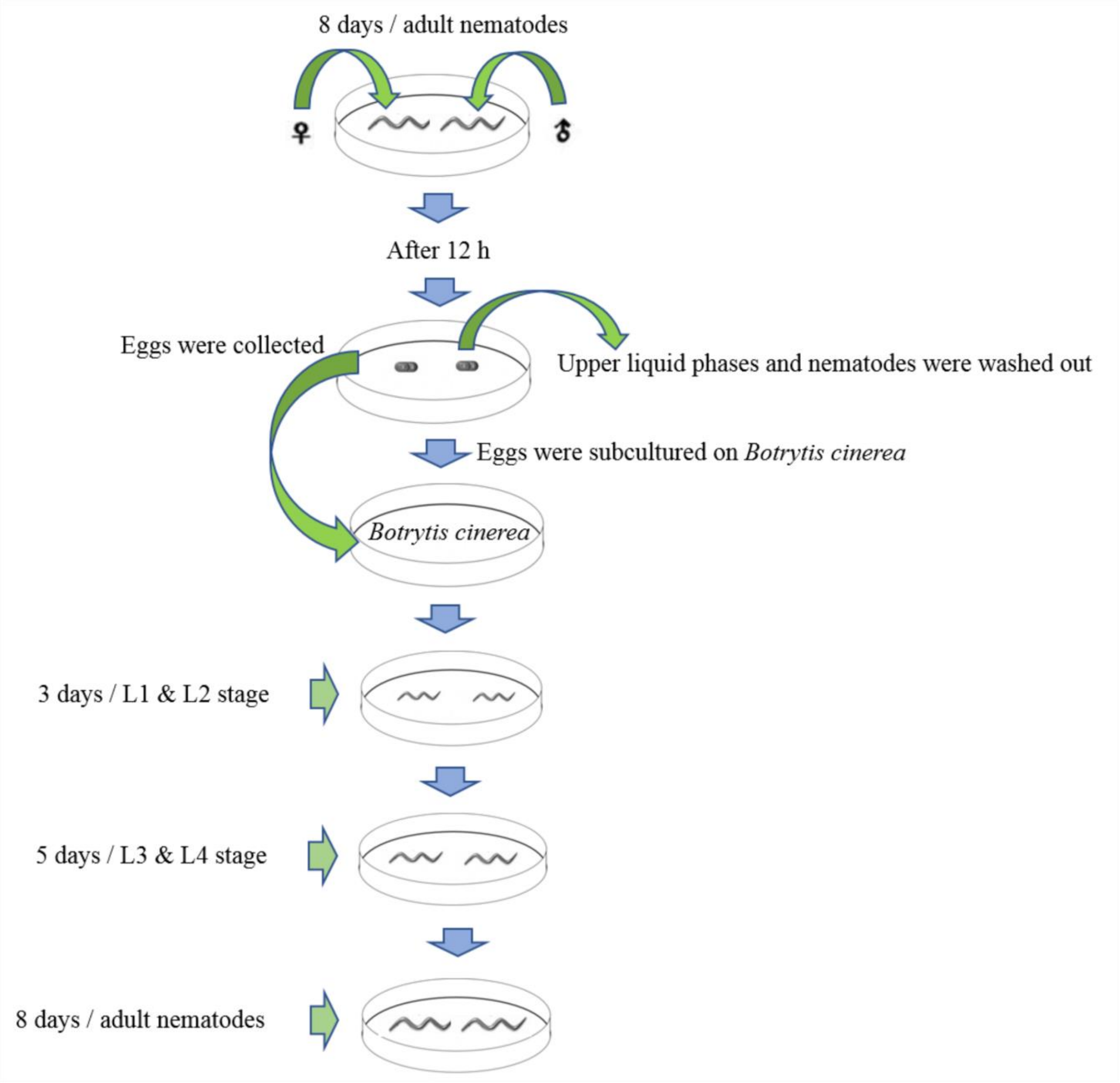

Movie S1. Control 1: Treatment of water on egg. After 24 h, 100% of egg was hatched to a L1/L2 juvenile stage of nematodes.

Movie S2. Control 2: Treatment of 3% DMSO in methanol on egg. After 24 h, 95% of egg was hatched to al1 L1/L2 juvenile stage of nematodes.

Movie S3. Positive control: Treatment of abamectin at 1,000 ppm. After 24 h, egg embryo was arrested, and the least hatching rate of egg was 31%.

Movie S4. Treatment of crude extract of *Stenotrophomonas* sp. at 1,000 ppm. After 24 h, movement of active egg embryo was arrested, and the least hatching rate of egg was 57%.

Movie S5. Treatment of crude extract of *Bacillus* sp. at 1,000 ppm. After 24 h, movement of active egg embryo was arrested, and the least hatching rate of egg was 64%.

Movie S6. Control 1: Treatment of water on L3/L4 juvenile stage. After 24 h, 100% of L3/L4 juvenile stage of nematodes was alive.

Movie S7. Control 2: Treatment of 3% DMSO in methanol on L3/L4 juvenile stage. After 24 h, 95% of L3/L4 juvenile stage of nematodes was alive.

Movie S8. Positive control: Treatment of abamectin at 1,000 ppm. After 24 h, 80% of L3/L4 juvenile stage nematodes was confirmed as dead and paralyzed.

Movie S9. Treatment of crude extract of *Stenotrophomonas* sp. at1,000 ppm. After 24 h, 64% of L3/L4 juvenile stage nematodes was confirmed as dead and paralyzed.

Movie S10. Treatment of crude extract of *Bacillus* sp. at 1,000 ppm. After 24 h, 61% of L3/L4 juvenile stage nematodes was confirmed as dead and paralyzed.

Movie S11. Control 1: Treatment of water on adult stage. After 24 h, 100% of L3/L4 adult stage of nematodes was alive.

Movie S12. Control 2: Treatment of 3% DMSO in methanol on adult stage. After 24 h, 98% of adult stage of nematodes was alive.

Movie S13. Positive control: Treatment of abamectin at 1,000 ppm. After 24 h, 86% of adult stage nematodes was confirmed as dead and paralyzed.

Movie S14. Treatment of crude extract of *Stenotrophomonas* sp. at 1,000 ppm. After 24 h, 79% of adult stage nematodes was confirmed as dead and paralyzed.

Movie S15. Treatment of crude extract of *Bacillus* sp. at 1,000 ppm. After 24 h, 70% of adult stage nematodes was confirmed as dead and paralyzed.

Note S1. 16S rDNA sequences of 389 bacterial endophytes.

```
>EB1
TGCAAGTCGAACGGCAGCACGGACTTCGGTCTGGTGGCGAGTGGCGAACGGGTGAGTAATGTATCGGAACGTGCCTAGTAGCGGGGGATAACTACGCGAA
AGCGTAGCTAATACCGCATACGCCCTTCGGGGGAAAGCAGGGGATCGCAAGACCTTGCACTATTAGAGCGGCCGATATCGGATTAGCTAGTTGGTGGGGT
AACGGCTCACCAAGGCGACGATCCGTAGCTGGTTTGAGAGGACGACCAGCCACACTGGGACTGAGACACGGCCCAGACTCCTACGGGAGGCAGCAGTGGG
GAATTTTGGACAATGGGGGAAACCCTGATCCAGCCATCCCGCGTGTGCGATGAAGGCCTTCGGGTTGTAAAGCACTTTTGGCAGGAAAGAAACGTCATGG
GGCTAATACCCCCGTGAAACTGACGGTACCTGCAGAATAAGCACCGGCTAACTACGTGCCAGCAGCCGCGGTAATACGTAGGGTGCAAGCGTTAATCGGA
ATTACTGGGCGTAAAGCGTGCGCAGGCGGTTCGGAAAGAAAGATGTGAAATCCCAGAGCTTAACTTTGGAACTGCATTTTTAACTACCGAGCTAGAGTGT
GTCAGAGGGAGGTGGAATTCCGCGTGTAGCAGTGAAATGCGTAGATATGCGGAGGAACACCGATGGCGAAGGCAGCCTCCTGGGATAACACTGACGCTCA
TGCACGAAAGCGTGGGGAGCAAACAGGATTAGATACCCTGGTAGTCCACGCCCTAAACGATGTCAACTAGCTGTTGGGGCCTTCGGGCCTTGGTAGCGCA
GCTAACGCGTGAAGTTGACCGCCTGGGGAGTACGGTCGCAAGATTAAAACTCAAAGGAATTGACGGGGACCCGCACAAGCGGTGGATGATGTGGATTAAT
TCGATGCAACGCGAAAAACCTTACCTACCCTTGACATGTCTGGAATGCCGAAGAGATTTGGCAGTGCTCGCAAGAGAACCGGAACACAGGTGCTGCATGG
CTGTCGTCAGCTCGTGTCGTGAGATGTTGGGTTAAGTCCCGCAACGAGCGCAACCCTTGTCATTAGTTGCTACGAAAGGGCACTCTAATGAGACTGCCGG
TGACAAACCGGAGGAAGGTGGGGATGACGTCAAGTCCTCATGGCCCTTATGGGTAGGGCTTCACACGTCATACAATGGTCGGGACAGAGGGTCGCCAACC
CGCGAGGGGGAGCCAATCCCAGAAACCCGATCGTAGTCCGGATCGCAGTCTGCAACTCGACTGCGTGAAGTCGGAATCGCTAGTAATCGCGGATCAGCAT
GTCGCGGTGAATACGTTCCCGGGTCTTGTACACACCGCCCGTCACACCATGGGAGTGGGTTTTACCAGAAGTAGTTAGCCTAACCGCAAGGAGGGCGATA
CCA

>EB2
CGGCAGCACGGACTTCGGTCTGGTGGCGAGTGGCGAACGGGTGAGTAATGTATCGGAACGTGCCTAGTAGCGGGGGATAACTACGCGAAAGCGTAGCTAA
TACCGCATACGCCCTACGGGGGAAAGCAGGGGATCGCAAGACCTTGCACTATTAGAGCGGCCGATATCGGATTAGCTAGTTGGTGGGGTAACGGCTCACC
AAGGCGACGATCCGTAGCTGGTTTGAGAGGACGACCAGCCACACTGGGACTGAGACACGGCCCAGACTCCTACGGGAGGCAGCAGTGGGGAATTTTGGAC
AATGGGGGAAACCCTGATCCAGCCATCCCGCGTGTGCGATGAAGGCCTTCGGGTTGTAAAGCACTTTTGGCAGGAAAGAAACGTCATGGGTTAATACCCC
GTGAAACTGACGGTACCTGCAGAATAAGCACCGGCTAACTACGTGCCAGCAGCCGCGGTAATACGTAGGGTGCAAGCGTTAATCGGAATTACTGGGCGTA
AAGCGTGCGCAGGCGGTTCGGAAAGAAAGATGTGAAATCCCAGAGCTTAACTTTGGAACTGCATTTTTAACTACCGAGCTAGAGTGTGTCAGAGGGAGGT
GGAATTCCGCGTGTAGCAGTGAAATGCGTAGATATGCGGAGGAACACCGATGGCGAAGGCAGCCTCCTGGGATAACACTGACGCTCATGCACGAAAGCGT
GGGGAGCAAACAGGATTAGATACCCTGGTAGTCCACGCCCTAAACGATGTCAACTAGCTGTTGGGGCCTTCGGGCCTTGGTAGCGCAGCTAACGCGTGAA
GTTGACCGCCTGGGGAGTACGGTCGCAAGATTAAAACTCAAAGGAATTGACGGGGACCCGCACAAGCGGTGGATGATGTGGATTAATTCGATGCAACGCG
AAAAACCTTACCTACCCTTGACATGTCTAGAAGGCCGAAGAGATTTGGCTGTGCTCGCAAGAGAACTGGAACACAGGTGCTGCATGGCTGTCGTCAGCTC
GTGTCGTGAGATGTTGGGTTAAGTCCCGCAACGAGCGCAACCCTTGTCATTAGTTGCTACGAAAGGGCACTCTAATGAGACTGCCGGTGACAAACCGGAG
GAAGGTGGGGATGACGTCAAGTCCTCATGGCCCTTATGGGTAGGGCTTCACACGTCATACAATGGTCGGGACAGAGGGTCGCCAACCCGCGAGGGGGAGC
CAATCCCAGAAACCCGATCGTAGTCCGGATCGCAGTCTGCAACTCGACTGCGTGAAGTCGGAATCGCTAGTAATCGCGGATCAGCATGTCGCGGTGAATA
CGTTCCCGGGTCTTGTACACACCGCCCGTCACACCATGGGAGTGGGTTTTACCAGAAGTAGTTAGCCTAACCGTAAGGGGGGCGAT

>EB3
TGCAGTCGAACGGCAGCACGGACTTCGGTCTGGTGGCGAGTGGCGAACGGGTGAGTAATGTATCGGAACGTGCCTAGTAGCGGGGGATAACTACGCGAAA
GCGTAGCTAATACCGCATACGCCCTTCGGGGGAAAGCAGGGGATCGCAAGACCTTGCACTATTAGAGCGGCCGATATCGGATTAGCTAGTTGGTGGGGTA
ACGGCTCACCAAGGCGACGATCCGTAGCTGGTTTGAGAGGACGACCAGCCACACTGGGACTGAGACACGGCCCAGACTCCTACGGGAGGCAGCAGTGGGG
AATTTTGGACAATGGGGGAAACCCTGATCCAGCCATCCCGCGTGTGCGATGAAGGCCTTCGGGTTGTAAAGCACTTTTGGCAGGAAAGAAACGTCATGGG
CTAATACCCCCGTGAAACTGACGGTACCTGCAGAATAAGCACCGGCTAACTACGTGCCAGCAGCCGCGGTAATACGTAGGGTGCAAGCGTTAATCGGAAT
TACTGGGCGTAAAGCGTGCGCAGGCGGTTCGGAAAGAAAGATGTGAAATCCCAGAGCTTAACTTTGGAACTGCATTTTTAACTACCGAGCTAGAGTGTGT
CAGAGGGAGGTGGAATTCCGCGTGTAGCAGTGAAATGCGTAGATATGCGGAGGAACACCGATGGCGAAGGCAGCCTCCTGGGATAACACTGACGCTCATG
CACGAAAGCGTGGGGAGCAAACAGGATTAGATACCCTGGTAGTCCACGCCCTAAACGATGTCAACTAGCTGTTGGGGCCTTCGGGCCTTGGTAGCGCAGC
TAACGCGTGAAGTTGACCGCCTGGGGAGTACGGTCGCAAGATTAAAACTCAAAGGAATTGACGGGGACCCGCACAAGCGGTGGATGATGTGGATTAATTC
GATGCAACGCGAAAAACCTTACCTACCCTTGACATGTCTGGAATGCCGAAGAGATTTGGCAGTGCTCGCAAGAGAACCGGAACACAGGTGCTGCATGGCT
GTCGTCAGCTCGTGTCGTGAGATGTTGGGTTAAGTCCCGCAACGAGCGCAACCCTTGTCATTAGTTGCTACGAAAGGGCACTCTAATGAGACTGCCGGTG
ACAAACCGGAGGAAGGTGGGGATGACGTCAAGTCCTCATGGCCCTTATGGGTAGGGCTTCACACGTCATACAATGGTCGGGACAGAGGGTCGCCAACCCG
CGAGGGGGAGCCAATCCCAGAAACCCGATCGTAGTCCGGATCGCAGTCTGCAACTCGACTGCGTGAAGTCGGAATCGCTAGTAATCGCGGATCAGCATGT
CGCGGTGAATACGTTCCCGGGTCTTGTACACACCGCCCGTCACACCATGGGAGTGGGTTTTACCAGAAGTAGTTAGCCTAACCGCAAGGAGGGCGATACC
A

>EB4
TGCAGTCGAACGGCAGCACGGACTTCGGTCTGGTGGCGAGTGGCGAACGGGTGAGTAATGTATCGGAACGTGCCTAGTAGCGGGGGATAACTACGCGAAA
GCGTAGCTAATACCGCATACGCCCTACGGGGGAAAGCAGGGGATCGCAAGACCTTGCACTATTAGAGCGGCCGATATCGGATTAGCTAGTTGGTGGGGTA
ATGGCTCACCAAGGCGACAATCCGTACCGGGTTTGAGAGGACGACCAGCCACACTGGGACTGAGACACGGCCCAGACTCCTACGGGAGGCAGCAGTGGGG
AATTTTGGACAATGGGGGAAACCCTGATCCAGCCATCCCGCGTGTGCGATGAAGGCCTTCGGGTTGTAAAGCACTTTTGGCAGGAAAGAAACGTCATGGG
CTAATACCCCGTGAAACTGACGGTACCTGCAGAATAAGCACCGGCTAACTACGTGCCAGCAGCCGCGGTAATACGTAGGGTGCAAGCGTTAATCGGAATT
ACTGGGCGTAAAGCGTGCGCAGGCGGTTCGGAAAGAAAGATGTGAAATCCCAGAGCTTAACTTTGGAACTGCATTTTTAACTACCGGGCTAGAGTGTGTC
AGAGGGAGGTGGAATTCCGCGTGTAGCAGTGAAATGCGTAGATATGCGGAGGAACACCGATGGCGAAGGCAGCCTCCTGGGATAACACTGACGCTCATGC
ACGAAAGCGTGGGGGAGCAAACAGGATTAGATACCCTGGTAGTCCACGCCCTAAACGATGTCAACTAGCTGTTGGGGCCTTCGGGCCTTAGTAGCGCAGC
TAACGCGTGAAGTTGACCGCCTGGGGAGTACGGTCGCAAGATTAAAACTCAAAGGAATTGACGGGGACCCGCACAAGCGGTGGATGATGTGGATTAATTC
```

GATGCAACGCGAAAAACCTTACCTACCCTTGACATGTCTGGAATTCCGAAGAGATTTGGAAGTGCTCGCAAGAGAACCGGAACACAGGTGCTGCATGGCT
GTCGTCAGCTCGTGTCGTGAGATGTTGGGTTAAGTCCCGCAACGAGCGCAACCCTTGTCATTAGTTGCTACGAAAGGGCACTCTAATGAGACTGCCGGTG
ACAAACCGGAGGAAGGTGGGGATGACGTCAAGTCCTCATGGCCCTTATGGGTAGGGCTTCACACGTCATACAATGGTCGGGACAGAGGGTCGCCAACCCG
CGAGGGGGAGCCAATCCCAGAAACCCGATCGTAGTCCGGATCGCAGTCTGCAACTCGACTGCGTGAAGTCGGAATCGCTAGTAATCGCGGATCAGCATGT
CGCGGTGAATACGTTCCCGGGTCTTGTACACACCGCCCGTCACACCATGGGAGTGGGTTTTACCAGAAGTAGTTAGCCTAACCGCAAGGGGGGCGAT

>EB5
TGCAAGTCGAACGGCAGCACGGACTTCGGTCTGGTGGCGAGTGGCGAACGGGTGAGTAATGTATCGGAACGTGCCTAGTAGCGGGGGATAACTACGCGAA
AGCGTAGCTAATACCGCATACGCCCTACGGGGGAAAGCAGGGGATCGCAAGACCTTGCACTATTAGAGCGGCCGATATCGGATTAGCTAGTTGGTGGGGT
AACGGCTCACCAAGGCGACGATCCGTAGCTGGTTTGAGAGGACGACCAGCCACACTGGGACTGAGACACGGCCCAGACTCCTACGGGAGGCAGCAGTGGG
GAATTTTGGACAATGGGGGAAACCCTGATCCAGCCATCCCGCGTGTGCGATGAAGGCCTTCGGGTTGTAAAGCACTTTTGGCAGGAAAGAAACGTCATGG
GTTAATACCCCGTGAAACTGACGGTACCTGCAGAATAAGCACCGGCTAACTACGTGCCAGCAGCCGCGGTAATACGTAGGGTGCAAGCGTTAATCGGAAT
TACTGGGCGTAAAGCGTGCGCAGGCGGTTCGGAAAGAAAGATGTGAAATCCCAGAGCTTAACTTTGGAACTGCATTTTTAACTACCGGGCTAGAGTGTGT
CAGAGGGAGGTGGAATTCCGCGTGTAGCAGTGAAATGCGTAGATATGCGGAGGAACACCGATGGCGAAGGCAGCCTCCTGGGATAACACTGACGCTCATG
CACGAAAGCGTGGGGAGCAAACAGGATTAGATACCCTGGTAGTCCACGCCCTAAACGATGTCAACTAGCTGTTGGGGCCTTCGGCCTTAGTAGCGCAGC
TAACGCGTGAAGTTGACCGCCTGGGGAGTACGGTCGCAAGATTAAAACTCAAAGGAATTGACGGGGACCCGCACAAGCGGTGGATGATGTGGATTAATTC
GATGCAACGCGAAAAACCTTACCTACCCCTTGACATGTCTGGAATTCCGAAGAGATTTGGAAGTGCTCGCAAGAGAACCGGAACACAGGTGCTGCATGGC
TGTCGTCAGCTCGTGTCGTGAGATGTTGGGTTAAGTCCCGCAACGAGCGCAACCCTTGTCATTAGTTGCTACGAAAGGGCACTCTAATGAGACTGCCGGT
GACAAACCGGAGGAAGGTGGGGATGACGTCAAGTCCTCATGGCCCTTATGGGTAGGGCTTCACACGTCATACAATGGTCGGGACAGAGGGTCGCCAACCC
GCGAGGGGGAGCCAATCCCAGAAACCCGATCGTAGTCCGGATCGCAGTCTGCAACTCGACTGCGTGAAGTCGGAATCGCTAGTAATCGCGGATCAGCATG
TCGCGGTGAATACGTTCCCGGGTCTTGTACACACCGCCCGTCACACCATGGGAGTGGGTTTTACCAGAAGTAGTTAGCCTAACCGTAAGGGGGGCGAT

>EB6
TTGCGGTTAGGCTAACTACTTCTGGTAAAACCCACTCCCATGGTGTGACGGGCGGTGTGTACAAGACCCGGGAACGTATTCACCGCGACATGCTGATCCG
CGATTACTAGCGATTCCGACTTCACGCAGTCGAGTTGCAGACTGCGATCCGGACTACGATCGGGTTTCTGGGATTGGCTCCCCCTCGCGGGTTGGCGACC
CTCTGTCCCGACCATTGTATGACGTGTGAAGCCCTACCCATAAGGGCCATGAGGACTTGACGTCATCCCCACCTTCCTCCGGTTTGTCACCGGCAGTCTC
ATTAGAGTGCCCTTTCGTAGCAACTAATGACAAGGGTTGCGCTCGTTGCGGGACTTAACCCAACATCTCACGACACGAGCTGACGACAGCCATGCAGCAC
CTGTGTTCCGGTTCTCTTGCGAGCACTTCCAAATCTCTTCGGAATTCCAGACATGTCAAGGGGTAGGTAAGGTTTTTCGCGTTGCATCGAATTAATCCAC
ATCATCCACCGCTTGTGCGGGTCCCCGTCAATTCCTTTGAGTTTTAATCTTGCGACCGTACTCCCCAGGCGGTCAACTTCACGCGTTAGCTGCGCTACT
AAGGCCCGAAGGCCCCAACAGCTAGTTGACATCGTTTAGGGCGTGGACTACCAGGGTATCTAATCCTGTTTGCTCCCCACGCTTTCGTGCATGAGCGTCA
GTGTTATCCCAGGAGGCTGCCTTCGCCATCGGTGTTCCTCCGCATATCTACGCATTTCACTGCTACACGCGGAATTCCACCTCCCTCTGACACACTCTAG
CCCGGTAGTTAAAAATGCAGTTCCAAAGTTAAGCTCTGGGATTTCACATCTTTCTTTCCGAACCGCCTGCGCACGCTTTACGCCCAGTAATTCCGATTAA
CGCTTGCACCCTACGTATTACCGCGGCTGCTGGCACGTAGTTAGCCGGTGCTTATTCTGCAGGTACCGTCAGTTTCACGGGGTATTAGCCCATGACGTTT
CTTTCCTGCCAAAAGTGCTTTACAACCCGAAGGCCTTCATCGCACACGCGGGATGGCTGGATCAGGGTTTCCCCCATTGTCCAAAATTCCCCACTGCTGC
CTCCCGTAGGAGTCTGGGCCGTGTCTCAGTCCCAGTGTGGCTGGTCGTCCTCTCAAACCAGCTACGGATCGTCGCCTTGGTGAGCCATTACCCCACCAAC
TAGCTAATCCGATATCGGCCGCTCTAATAGTGCAAGGTCTTGCGATCCCCTGCTTTCCCCCGTAGGGCGTATGCGGTATTAGCTACGCTTTCGCGTAGTT
ATCCCCCGCTACTAGGCACGTTCCGATACATTACTCACCCGTTCGCCACTCGCCACCAGACCGAAGTCCGTGCTGCCGTTCGACTGCA

>EB7
GCCCCCCTTACGGTTAGGCTAACTACTTCTGGTAAAACCCACTCCCATGGTGTGACGGGCGGTGTGTACAAGACCCGGGAACGTATTCACCGCGACATGC
TGATCCGCGATTACTAGCGATTCCGACTTCACGCAGTCGAGTTGCAGACTGCGATCCGGACTACGATCGGGTTTCTGGGATTGGCTCCCCCTCGCGGGTT
GGCGACCCTCTGTCCCGACCATTGTATGACGTGTGAAGCCCTACCCATAAGGGCCATGAGGACTTGACGTCATCCCCACCTTCCTCCGGTTTGTCACCGG
CAGTCTCATTAGAGTGCCCTTTCGTAGCAACTAATGACAAGGGTTGCGCTCGTTGCGGGACTTAACCCAACATCTCACGACACGAGCTGACGACAGCCAT
GCAGCACCTGTGTTCCGGTTCTCTTGCGAGCACTTCCAAATCTCTTCGGACTTCTAGACATGTCAAGGGTAGGTAAGGTTTTTCGCGTTGCATCGAATTA
ATCCACATCATCCACCGCTTGTGCGGGTCCCCGTCAATTCCTTTGAGTTTTAATCTTGCGACCGTACTCCCCAGGCGGTCAACTTCACGCGTTAGCTGCG
CTACCAAGGCCCGAAGGCCCCAACAGCTAGTTGACATCGTTTAGGGCGTGGACTACCAGGGTATCTAATCCTGTTTGCTCCCCACGCTTTCGTGCATGAG
CGTCAGTGTTATCCCAGGAGGCTGCCTTCGCCATCGGTGTTCCTCCGCATATCTACGCATTTCACTGCTACACGCGGAATTCCACCTCCCTCTGACACAC
TCTAGCTCGGTAGTTAAAAATGCAGTTCCAAAGTTAAGCTCTGGGATTTCACATCTTTCTTTCCGAACCGCCTGCGCACGCTTTACGCCCAGTAATTCCG
ATTAACGCTTGCACCCTACGTATTACCGCGGCTGCTGGCACGTAGTTAGCCGGTGCTTATTCTGCAGGTACCGTCAGTTTCACGGGGTATTAACCCATGA
CGTTTCTTTCCTGCCAAAAGTGCTTTACAACCCGAAGGCCTTCATCGCACACGCGGGATGGCTGGATCAGGGTTTCCCCCATTGTCCAAAATTCCCCACT
GCTGCCTCCCGTAGGAGTCTGGGCCGTGTCTCAGTCCCAGTGTGGCTGGTCGTCCTCTCAAACCAGCTACGGATCGTCGCCTTGGTGAGCCGTTACCCCA
CCAACTAGCTAATCCGATATCGGCCGCTCTAATAGTGCAAGGTCTTGCGATCCCCTGCTTTCCCCCGTAGGGCGTATGCGGTATTAGCTACGCTTTCGCG
TAGTTATCCCCCGCTACTAGGCACGTTCCGATACATTACTCACCCGTTCGCCACTCGCCACCAGACCGAAGTCCGTGCTGCCGTTCGACTGCA

>EB8
TCGCCCCCCTTGCGGTTAGGCTAACTACTTCTGGTAAAACCCACTCCCATGGTGTGACGGGCGGTGTGTACAAGACCCGGGAACGTATTCACCGCGACAT
GCTGATCCGCGATTACTAGCGATTCCGACTTCACGCAGTCGAGTTGCAGACTGCGATCCGGACTACGATCGGGTTTCTGGGATTGGCTCCCCCTCGCGGG
TTGGCGACCCTCTGTCCCGACCATTGTATGACGTGTGAAGCCCTACCCATAAGGGCCATGAGGACTTGACGTCATCCCCACCTTCCTCCGGTTTGTCACC
GGCAGTCTCATTAGAGTGCCCTTTCGTAGCAACTAATGACAAGGGTTGCGCTCGTTGCGGGACTTAACCCAACATCTCACGACACGAGCTGACGACAGCC
ATGCAGCACCTGTGTTCCGGTTCTCTTGCGAGCACTTCCGAATCTCTTCAGAATTCCAGACATGTCAAGGGTAGGTAAGGTTTTTCGCGTTGCATCGAAT
TAATCCACATCATCCACCGCTTGTGCGGGTCCCCGTCAATTCCTTTGAGTTTTAATCTTGCGACCGTACTCCCCAGGCGGTCAACTTCACGCGTTAGCTG
CGCTACCAAGGCCCGAAGGCCCCAACAGCTAGTTGACATCGTTTAGGGCGTGGACTACCAGGGTATCTAATCCTGTTTGCTCCCCACGCTTTCGTGCATG
AGCGTCAGTGTTATCCCAGGAGGCTGCCTTCGCCATCGGTGTTCCTCCGCATATCTACGCATTTCACTGCTACACGCGGAATTCCACCTCCCTCTGACAC
ACTCTAGCTCGGTAGTTAAAAATGCAGTTCCAAAGTTAAGCTCTGGGATTTCACATCTTTCTTTCCGAACCGCCTGCGCACGCTTTACGCCCAGTAATTC
CGATTAACGCTTGCACCCTACGTATTACCGCGGCTGCTGGCACGTAGTTAGCCGGTGCTTATTCTGCAGGTACCGTCAGTTTCACGGGGTATTAACCCAT
GACGTTTCTTTCCTGCCAAAAGTGCTTTACAACCCGAAGGCCTTCATCGCACACGCGGGATGGCTGGATCAGGGTTTCCCCCATTGTCCAAAATTCCCCA
CTGCTGCCTCCCGTAGGAGTCTGGGCCGTGTCTCAGTCCCAGTGTGGCTGGTCGTCCTCTCAAACCAGCTACGGATCGTCGCCTTGGTGAGCCGTTACCC
CACCAACTAGCTAATCCGATATCGGCCGCTCTAATAGTGCAAGGTCTTGCGATCCCCTGCTTTCCCCCGTAGGGCGTATGCGGTATTAGCTACGCTTTCG
CGTAGTTATCCCCCGCTACTAGGCACGTTCCGATACATTACTCACCCGTTCGCCACTCGCCACCAGACCGAAGTCCGTGCTGCCGTTCGA

>EB9
GCAGTCGAACGGTAACAGGTCTTCGGATGCTGACGAGTGGCGAACGGGTGAGTAATACATCGGAACGTGCCCGAGAGTGGGGGATAACGAAGCGAAAGCT
TTGCTAATACCGCATACGATCTCAGGATGAAAGCAGGGGACCGCAAGGCCTTGCGCTCACGGAGCGGCCGATGGCAGATTAGGTAGTTGGTGGGATAAAA
GCTTACCAAGCCGACGATCTGTAGCTGGTCTGAGAGGACGACCAGCCACACTGGGACTGAGACACGGCCCAGACTCCTACGGGAGGCAGCAGTGGGGAAT
TTTGGACAATGGGCGCAAGCCTGATCCAGCCATGCCGCGTGCAGGATGAAGGCCTTCGGGTTGTAAACTGCTTTTGTACGGAACGAAAAGACTCTGGTTA
ATACCTGGGGTCCATGACGGTACCGTAAGAATAAGCACCGGCTAACTACGTGCCAGCAGCCGCGGTAATACGTAGGGTGCGAGCGTTAATCGGAATTACT
GGGCGTAAAGCGTGCGCAGGCGGTTATATAAGACAGATGTGAAATCCCCGGGCTCAACCTGGGAACTGCATTTGTGACTGTATAGCTAGAGTACGGCAGA
GGGGGATGGAATTCCGCGTGTAGCAGTGAAATGCGTAGATATGCGGAGGAACACCGATGGCGAAGGCAATCCCCTGGGCCTGTACTGACGCTCATGCACG

AAAGCGTGGGGAGCAAACAGGATTAGATACCCTGGTAGTCCACGCCCTAAACGATGTCAACTGGTTGTTGGGTCTTCACTGACTCAGTAACGAAGCTAAC
GCGTGAAGTTGACCGCCTGGGGAGTACGGCCGCAAGGTTGAAACTCAAAGGAATTGACGGGGACCCGCACAAGCGGTGGATGATGTGGTTTAATTCGATG
CAACGCGAAAAACCTTACCCACCTTTGACATGTACGGAATCCTTTAGAGATAGAGGAGTGCTCGAAAGAGAACCGTAACACAGGTGCTGCATGGCTGTCG
TCAGCTCGTGTCGTGAGATGTTGGGTTAAGTCCCGCAACGNAGCGCAACCCTTGTCATTAGTTGCTACATTCAGTTGGGCACTCTAATGAGACTGCCGGT
GACAAACCGGAGGAAGGTGGGGATGACGTCAAGTCCTCATGGCCCTTATAGGTGGGGCTACACACGTCATACAATGGCTGGTACAGAGGGTTGCCAACCC
GCGAGGGGGAGCCAATCCCATAAAGCCAGTCGTAGTCCGGATCGCAGTCTGCAACTCGACTGCGTGAAGTCGGAATCGCTAGTAATCGCGGATCAGAATG
TCGCGGTGAATACGTTCCCGGGTCTTGTACACACCGCCCGTCACACCATGGGAGCGGGTTCTGCCAGAAGTAGTTAGCCTAACCGCAAGGAGGGCGAT

>EB10
TGCAAGTCGAACGGTAACAGGTCTTCGGATGCTGACGAGTGGCGAACGGGTGAGTAATACATCGGAACGTGCCCGATCGTGGGGGATAACGGAGCGAAAG
CTTTGCTAATACCGCATACGATCTACGGATGAAAGCAGGGGACCGCAAGGCCTTGCGCGGACGGAGCGGCCGATGGCAGATTAGGTAGTTGGTGGGATAA
AAGCTTACCAAGCCGACGATCTGTAGCTGGTCTGAGAGGACGACCAGCCACACTGGGACTGAGACACGGCCCAGACTCCTACGGGAGGCAGCAGTGGGGA
ATTTTGGACAATGGGCGCAAGCCTGATCCAGCCATGCCGCGTGCAGGATGAAGGCCTTCGGGTTGTAAACTGCTTTTGTACGGAACGAAAAGACTTCTTC
TAATACAGGAGGTCCATGACGGTACCGTAAGAATAAGCACCGGCTAACTACGTGCCAGCAGCCGCGGTAATACGTAGGGTGCGAGCGTTAATCGGAATTA
CTGGGCGTAAAGCGTGCGCAGGCGGTTATATAAGACAGATGTGAAATCCCCGGGCTCAACCTGGGAACTGCATTTGTGACTGTATAGCTAGAGTACGGCA
GAGGGGGATGGAATTCCGCGTGTAGCAGTGAAATGCGTAGATATGCGGAGGAACACCGATGGCGAAGGCAATCCCCTGGGCCTGTACTGACGCTCATGCA
CGAAAGCGTGGGGAGCAAACAGGATTAGATACCCTGGTAGTCCACGCCCTAAACGATGTCAACTGGTTGTTGGGTCTTCACTGACTCAGTAACGAAGCTA
ACGCGTGAAGTTGACCGCCTGGGGAGTACGGCCGCAAGGTTGAAAACTCAAAGGAATTGACGGGGACCCGCACAAGCGGTGGATGATGTGGTTTAATTCG
ATGCAACGCGAAAAACCTTACCCACCTTTGACATGTACGGAATCCTTTAGAGATAGAGGAGTGCTCGAAAGAGAGCCGTAACACAGGTGCTGCATGGCTG
TCGTCAGCTCGTGTCGTGAGATGTTGGGTTAAGTCCCGCAACGAGCGCAACCCTTGTCATTAGTTGCTACATTCAGTTGGGCACTCTAATGAGACTGCCG
GTGACAAACCGGAGGAAGGTGGGGATGACGTCAAGTCCTCATGGCCCTTATAGGTGGGGCTACACACGTCATACAATGGCTGGTACAGAGGGTTGCCAAC
CCGCGAGGGGGAGCCAATCCCATAAAGCCAGTCGTAGTCCGGATCGCAGTCTGCAACTCGACTGCGTGAAGTCGGAATCGCTAGTAATCGCGGATCAGAA
TGTCGCGGTGAATACGTTCCCGGGTCTTGTACACACCGCCCGTCACACCATGGGAGCGGGTTCTGCCAGAAGTAGGTAGCCTAACCGTAAGGAGGGCGCT

>EB11
AGCGTCCTCCTTGCGGTTAGACTACCTACTTCTGGTGCAACAAACTCCCATGGTGTGACGGGCGGTGTGTACAAGGCCCGGGAACGTATTCACCGCGGCA
TTCTGATCCGCGATTACTAGCGATTCCGACTTCATGGAGTCGAGTTGCAGACTCCAATCCGGACTACGATCGGCTTTTTGAGATTAGCATCCTATCGCTA
GGTAGCAACCCTTTGTACCGACCATTGTAGCACGTGTGTAGCCCTGGCCGTAAGGGCCATGATGACTTGACGTCGTCCCCGCCTTCCTCCAGTTTGTCAC
TGGCAGTATCCTTAAAGTTCCCGACATTACTCGCTGGCAAATAAGGAAAAGGGTTGCGCTCGTTGCGGGACTTAACCCAACATCTCACGACACGAGCTGA
CGACAGCCATGCAGCACCTGTATGTAAGTTCCCGAAGGCACCAATCCATCTCTGGAAAGTTCTTACTATGTCAAGGCCAGGTAAGGTTCTTCGCGTTGCA
TCGAATTAAACCACATGCTCCACCGCTTGTGCGGGCCCCCGTCAATTCATTTGAGTTTTAGTCTTGCGACCGTACTCCCCAGGCGGTCTACTTATCGCGT
TAGCTGCGCCACTAAAGCCTCAAAGGCCCCAACGGCTAGTAGACATCGTTTACGGCATGGACTACCAGGGTATCTAATCCTGTTTGCTCCCCATGCTTTC
GCACCTCAGCGTCAGTGTTAGGCCAGATGGCTGCCTTCGCCATCGGTATTCCTCCAGATCTCTACGCATTTCACCGCTACACCTGGAATTCTACCATCCT
CTCCCACACTCTAGCTAACCAGTATCGAATGCAATTCCCAAGTTAAGCTCGGGGATTTCACATTTGACTTAATTAGCCGCCTACGCGCGCTTTACGCCCA
GTAAATCCGATTAACGCTTGCACCCTCTGTATTACCGCGGCTGCTGGCACAGAGTTAGCCGGTGCTTATTCTGCGAGTAACGTCCACTATCTGAAGGTAT
TAACTTCAGTAGCCTCCTCCTCGCTTAAAGTGCTTTACAACCATAAGGCCTTCTTCACACACGCGGCATGGCTGGATCAGGCTTGCGCCCATTGTCCAAT
ATTCCCCACTGCTGCCTCCCGTAGGAGTCTGGGCCGTGTCTCAGTCCCAGTGTGGCGGATCATCCTCTCAGACCCGCTACAGATCGTCGCCTTGGTAGGC
CTTTACCCCACCAACTAGCTAATCCGACTTAGGCTCATCTATTAGCGCAAGGTCCGAAGATCCCCTGCTTTCTCCCGTAGGACGTATGCGGTATTAGCAT
TCCTTTCGAAATGTTGTCCCCCACTAATAGGCAGATTCCTAAGCATTACTCACCCGTCCGCCGCTAAGTGATAGTGCAAGCACCATCACTCCGCTCGACT
TGCATG

>EB12
TGCAGTCGAGCGGGGAAGAGTAGCTTGCTACTTGACCTAGCGGCGGACGGGTGAGTAATGCTTAGGAATCTGCCTATTAGTGGGGGACAACATCTCGAAA
GGGATGCTAATACCGCATACGTCCTACGGGAGAAAGCAGGGGACCTTCGGGCCTTGCGCTAATAGATGAGCCTAAGTCGGATTAGCTAGTTGGTGGGGTA
AAGGCCTACCAAGGCGACGATCTGTAGCGGGTCTGAGAGGATGATCCGCCACACTGGGACTGAGACACGGCCCAGACTCCTACGGGAGGCAGCAGTGGGG
AATATTGGACAATGGGGGGAACCCTGATCCAGCCATGCCGCGTGTGTGAAGAAGGCCTTTTGGTTGTAAAGCACTTTAAGCGAGGAGGAGGCTACCGAGA
TTAATACTCTTGGATAGTGGACGTTACTCGCAGAATAAGCACCGGCTAACTCTGTGCCAGCAGCCGCGGTAATACAGAGGGTGCAAGCGTTAATCGGATT
TACTGGGCGTAAAGCGCGCGTAGGTGGCCAATTAAGTCAAATGTGAAATCCCCGAGCTTAACTTGGGAATTGCATTCGATACTGGTTGGCTAGAGTATGG
GAGAGGATGGTAGAATTCCAGGTGTAGCGGTGAAATGCGTAGAGATCTGGAGGAATACCGATGGCGAAGGCAGCCATCTGGCCTAATACTGACACTGAGG
TGCGAAAGCATGGGGAGCAAACAGGATTAGATACCCTGGTAGTCCATGCCGTAAACGATGTCTACTAGCCGTTGGGGCCTTTGAGGCTTTAGTGGCGCAG
CTAACGCGATAAGTAGACCGCCTGGGGAGTACGGTCGCAAGACTAAAACTCAAATGAATTGACGGGGGCCCGCACAAGCGGTGGAGCATGTGGTTTAATT
CGATGCAACGCGAAGAACCTTACCTGGTCTTGACATAGTAAGAACTTTCCAGAGATGGATTGGTGCCTTCGGGAACTTACATACAGGTGCTGCATGGCTG
TCGTCAGCTCGTGTCGTGAGATGTTGGGTTAAGTCCCGCAACGAGCGCAACCCTTTTCCTTATTTGCCAGCGGGTTAAGCCGGGAACTTTAAGGATACTG
CCAGTGACAAACTGGAGGAAGGCGGGGACGACGTCAAGTCATCATGGCCCTTACGACCAGGGCTACACACGTGCTACAATGGTCGGTACAAAGGGTTGCT
ACCACGCGAGTGGATGCTAATCTCAAAAAGCCGATCGTAGTCCGGATTGGAGTCTGCAACTCGACTCCATGAAGTCGGAATCGCTAGTAATCGCGGATCA
GAATGCCGCGGTGAATACGTTCCCGGGCCTTGTACACACCGCCCGTCACACCATGGGAGTTTGTTGCACCAGAAGTAGGTAGTCTAACCTTAGGGGGGAC
GCT

>EB13
TGCAAGTCGAGCGGAGAGAGGTAGCTTGCTACTGATCTTAGCGGCGGACGGGTGAGTAATGCTTAGGAATCTGCCTATTAGTGGGGGACAACATTTCGAA
AGGAATGCTAATACCGCATACGTCCTACGGGAGAAAGCAGGGGATCTTCGGACCTTGCGCTAATAGATGAGCCTAAGTCGGATTAGCTAGTTGGTGGGGT
AAAGGCCTACCAAGGCGACGATCTGTAGCGGGTCTGAGAGGATGATCCGCCACACTGGGACTGAGACACGGCCCAGACTCCTACGGGAGGCAGCAGTGGG
GAATATTGGACAATGGGCGGAAGCCTGATCCAGCCATGCCGCGTGTGTGAAGAAGGCCTTATGGTTGTAAAGCACTTTAAGCGAGGAGGAGGCTACTTTA
GTTAATACCTAGAGATAGTGGACGTTACTCGCAGAATAAGCACCGGCTAACTCTGTGCCAGCAGCCGCGGTAATACAGAGGGTGCAAGCGTTAATCGGAT
TTACTGGGCGTAAAGCGCGCGTAGGCGGCTAATTAAGTCAAATGTGAAATCCCCGAGCTTAACTTGGGAATTGCATTCGATACTGGTTAGCTAGAGTGTG
GGAGAGGATGGTAGAATTCCAGGTGTAGCGGTGAAATGCGTAGAGATCTGGAGGAATACCGATGGCGAAGGCAGCCATCTGGCCTAACACTGACGCTGAG
GTGCGAAAGCATGGGGAGCAAACAGGATTAGATACCCTGGTAGTCCATGCCGTAAACGATGTCTACTAGCCGTTGGGGCCTTTGAGGCTTTAGTGGCGCA
GCTAACGCGATAAGTAGACCGCCTGGGGAGTACGGTCGCAAGACTAAAACTCAAATGAATTGACGGGGGCCCGCACAAGCGGTGGAGCATGTGGTTTAAT
TCGATGCAACGCGAAGAACCTTACCTGGCCTTGACATAGTAAGAACTTTCCAGAGATGGATTGGTGCCTTCGGGAACTTACATACAGGTGCTGCATGGCT
GTCGTCAGCTCGTGTCGTGAGATGTTGGGTTAAGTCCCGCAACGAGCGCAACCCTTTTCCTTATTTGCCAGCGAGTAATGTCGGGAACTTTAAGGATACT
GCCAGTGACAAACTGGAGGAAGGCGGGGACGACGTCAAGTCATCATGGCCCTTACGGCCAGGGCTACACACGTGCTACAATGGTCGGTACAAAGGGTTGC
TACCTAGCGATAGGATGCTAATCTCAAAAAGCCGATCGTAGTCCGGATTGGAGTCTGCAACTCGACTCCATGAAGTCGGAATCGCTAGTAATCGCGGATC
AGAATGCCGCGGTGAATACGTTCCCGGGCCTTGTACACACCGCCCGTCACACCATGGGAGTTTGTTGCACCAGAAGTAGCTAGCCTAACTGCAAAGAGGG
C

>EB14
TGCAAGTCGAGCGGATGAAGGTAGCTTGCTACTGGATTCAGCGGCGGACGGGTGAGTAATGCTTAGGAATCTGCCTATTAGTGGGGGACAACGTTCCGAA
AGGAGCGCTAATACCGCATACGTCCTACGGGAGAAAGCAGGGGACCTTTGGGCCTTGCGCTAATAGATGAGCCTAAGTCGGATTAGCTAGTTGGTAGGGT

AAAGGCCTACCAAGGCGACGATCTGTAGCGGGTCTGAGAGGATGATCCGCCACACTGGGACTGAGACACGGCCCAGACTCCTACGGGAGGCAGCAGTGGG
GAATATTGGACAATGGGGGGAACCCTGATCCAGCCATGCCGCGTGTGTGAAGAAGGCCTTTTGGTTGTAAAGCACTTTAAGCGAGGAGGAGGCTACCTAG
ATTAATACTTTAGGATAGTGGACGTTACTCGCAGAATAAGCACCGGCTAACTCTGTGCCAGCAGCCGCGGTAATACAGAGGGTGCGAGCGTTAATCGGAT
TTACTGGGCGTAAAGCGTGCGTAGGCGGCCAATTAAGTCAAATGTGAAATCCCCGAGCTTAACTTGGGAATTGCATTCGATACTGGTTGGCTAGAGTATG
GGAGAGGATGGTAGAATTCCAGGTGTAGCGGTGAAATGCGTAGAGATCTGGAGGAATACCGATGGCGAAGGCAGCCATCTGGCCTAATACTGACGCTGAG
GTACGAAAGCATGGGGAGCAAACAGGATTAGATACCCTGGTAGTCCATGCCGTAAACGATGTCTACTAGCCGTTGGGGCCCTTGAGGCTTTAGTGGCGCA
GCTAACGCGATAAGTAGACCGCCTGGGGAGTACGGTCGCAAGACTAAAACTCAAATGAATTGACGGGGGCCCGCACAAGCGGTGGAGCATGTGGTTTAA
TTCGATGCAACGCGAAGAACCTTACCTGGCCTTGACATACAGAGAACTTTCCAGAGATGGATTGGTGCCTTCGGGAACTCTGATACAGGTGCTGCATGGC
TGTCGTCAGCTCGTGTCGTGAGATGTTGGGTTAAGTCCCGCAACGAGCGCAACCCTTTTCCTTATTTGCCAGCACTTCGGGTGGGAACTTTAAGGATACT
GCCAGTGACAAACTGGAGGAAGGCGGGGACGACGTCAAGTCATCATGGCCCTTACGGCCAGGGCTACACACGTGCTACAATGGTCGGTACAAAGGGTTGC
TACACAGCGATGTGATGCTAATCTCAAAAAGCCGATCGTAGTCCGGATTGGAGTCTGCAACTCGACTCCATGAAGTCGGAATCGCTAGTAATCGCGGATC
AGAATGCCGCGGTGAATACGTTCCCGGGCCTTGTACACACCGCCCGTCACACCATGGGAGTTTGTTGCACCAGAAGTAGGTAGTCTAACCGCAAGGAGGA
CGC

>EB15
CGTTGGTAGCGTCCTCCCTAAGGTTAGACTACCTACTTCTGGTGCAACAAACTCCCATGGTGTGACGGGCGGTGTGTACCAGGCCCGGGAACGTATTCAC
CGCGGCATTCTGATCCGCGATTACTAGCGATTCCGACTTCATGGAGTCGAGTTGCAGACTCCAATCCGGACTACGATCGGCTTTTTGAGATTAGCATCCT
CTCGCGAGGTAGCAACCCTTTGTACCGACCATTGTAGCACGTGTGTAGCCCTGGTCATAAGGGGCATGATGACTTGACGTCGTCCCCGCCTTCCTCCAGT
TTGTCACTGGCAGTATCCTTAAAGTTCCCGGCTTAACCCGCTGGGAAATAAGGAAAAGGGTTGCGCTCGTTGCGGGACTTAACCCCAACATCTCACGACA
CGAGCTGACGACAGCCCATGCAGCACCTGTATGTAAGTTCCCGAAGGCACCAATCCATCTCTGGAAAGTTCTTACTATGTCAAGACCAGGTAAGGGTCTT
CGCGTTGCATCGAATTAAACCACATGGTACCACTGCTTGTGCGGGCCCCCGTCAATTCATTTGAGTTTTAGTCTTGCGACCGTACTCCCCAGGCGGTCTA
CTTATCGCGTTAGCTGCGCCACTAAAGCCTCAAAGGCCCCAACGGCTAGTAGACATCGTTTACGGCATGGACTACCAGGGTATCTAATCCTGTTTGCTCC
CCATGCTTTCGCACCTCAGTGTCAGTATTATGCCAGATGGATGCCTTCCCCATCGGTATTCCTCCAGATCTCTACGCATTTCACCGCTACACCTGGAATT
CTACCATCCTCTCCCATACTCTAGCCAACCAGTATCGAATGCAATTCCCAACTTAAGCTCGGGGATTTCACATTTGGACTTAATTGGACCACCTACGCGC
GATTTTACGTCCCAGTAAATCCCGATTAACGCTTGCACCCTCTGTATTACCGCGGCTGCTGGCACAGAGTTAGCCGGTGCTTATTCTGCGAGTAACGTCC
ACTATCCAAGAGTATTAATCTCGGTAGCCTCCTCCTCGCTTAAAGTGCTTTACAACCAAAAGGCCTTCTTCACACACGCGGCATGGCTGGATCAGGGTTC
CCCCCATTGTCCAATATTCCCCACTGCTGCCTCCCGTAGGAGTCTGGGCCGTGTCTCAGTCCCAGTGTGGCGGATCATCCTCTCAGACCCGCTACAGATC
GTCGCCTTGGTAGGCCTTTACCCCACCAACTAGGTAATCCGACTTAGGCTCATCTATTAGCGCAAGGCCCGAAGGTCCCCTGCTTTCTCCCGTAGGACGT
ATGCGGTATTAGCATCCCTTTCGAGATGTTGTCCCCCCCTAATAGGCAGATTCCTAAGCATTACTCACCCGTCCGCCGCTAGGTAAGTAGCAAGTTACAT
TTCCCCGCTCGACTGCA

>EB16
GGCGGCTGGCTCCTTGCGGTTACCCCACCGACTTCGGGTGTTATAAACTCTCGTGGTGTGACGGGCGGTGTGTACAAGACCCGGGAACGTATTCACCGCG
GCATGCTGATCCGCGATTACTAGCAATTCCGACTTCATGCAGGCGAGTTGCAGCCTGCAATCCGAACTGAGACCGGCTTTTTAGGATTCGTTCCACCTCG
CGGCTTCACAGCCCGTTGTACCGGCCATTGTAGTACGTGTGTAGCCCAGGTCATAAGGGGCATGATGATTTGACGTCATCCCCACCNTTCCTCCGGTTTG
TCACCGGCAGTCACCTTAGAGTGCCCACCCGAAGTGCTGGCAACTAAGATCAAGGGTTGCGCTCGTTGCGGGACTTAACCCAACATCTCACGACACGAGC
TGACGACAACCATGCACCACCTGTCTCCTCTGTCCCGAAGGAAAGCCCTATCTCTAGGACGGTCAGAGGGATGTCAAGACCTGGTAAGGTTCTTCGCGTT
GCTTCGAATTAAACCACATACTCCACTGCTTGTGCGGGTCCCCGTCAATTCCTTTGAGTTTCAGTCTTGCGACCGTACTCCCCAGGCGGAATGCTTAATG
TGTTAACTTCGGCACCAAGGGTATCGAAACCCCTAACACCTAGCATTCATCGTTTACGGCGTGGACTACCAGGGTATCTAATCCTGTTTGCTCCCCACGC
TTTCGCGCCTCAGCGTCAGTTACAGCCCAGAGAGTCGCCTTCGCCACTGGTGTTCCTCCACATCTCTACGCATTTCACCGCTACACGTGGAATTCCACTC
TCCTCTTCTGCACTCAAGTCATCCAGTTTCCAGTGCGATCCGGGGTTGAGCCCCGGGATTAAACACCAGACTTAAATGACCGCCTGCGCGCGCTTTACGC
CCAATAATTCCGGACAACGCTTGCCCCCTACGTATTACCGCGGCTGCTGGCACGTAGTTAGCCGGGGCTTTCTTCTCAGGTACCGTCACTCCTTGAGCAG
TTACTCTCAAGGACGTTCTTCCCTGGCAACAGAGCTTTACGATCCGAAAACCTTCATCACTCACGCGGCATTGCTCCGTCAGGCTTTCGCCCATTGCGGA
AGATTCCCTACTGCTGCCTCCCGTAGGAGTCTGGGCCGTGTCTCAGTCCCAGTGTGGCCGATCACCCTCTCAGGTCGGCTACGCATCGTCGCCTTGGTGA
GCCGTTACCTCACCAACTAGCTAATGCGCCGCAGGCCCATCCTCAAGTGACAGATTGCTCCGTCTTTCCAGCTTCCTTCAGGCGAAGAAAGCAAGTATTC
GGTATTAGCTACCGTTTCCGGTAGTTGTCCCAAGCTTGAGGGCAGGTTGCCTACGTGTTACTCACCCGTCCGCCGCTAACCATCAGAGAAGCAACGCTTG
CTCTATGCAAGTCCGCTCGA

>EB17
CGGCTGGCTCCATAAAGGTTACCCCACCGACTTCGGGTGTTACAAACTCTCGTGGTGTGACGGGCGGTGTGTACAAGGCCCGGGAACGTATTCACCGTGG
CATGCTGATCCACGATTACTAGCGATTCCAGCTTCATGTAGGCGAGTTGCAGCCTACAATCCGAACTGAGAATGGTTTTATGGGATTGGCTCCCCCTCGC
GGGTTCGCAGCCCTTTGTACCATCCATTGTAGCACGTGTGTAGCCCAGGTCATAAGGGGCATGATGATTTGACGTCATCCCCACCTTCCTCCGGTTTGTC
ACCGGCAGTCACCTTAGAGTGCCCAACTGAATGCTGGCAACTAAGATCAAGGGTTGCGCTCGTTGCGGGACTTAACCCAACATCTCACGACACGAGCTGA
CGACAACCATGCACCACCTGTCACCGCCGTCCCCGAAGGGAAGGCCTAGTCTCCTAGGCGGTCGGCGGGATGTCAAGACCTGGTAAGGTTCTTCGCGTTG
CTTCGAATTAAACCACATGCTCCCACCGCTTGTGCGGGCCCCCGTCAATTCCTTTGAGTTTCAGTCTTGCGACCGTACTCCCCAGGCGGAGTGCTTAATG
CGTTAGCTGCAGCACTAAGGGGCGGAAACCCCCTAACACTTAGCACTCATCGTTTACGGCGTGGACTACCAGGGTATCTAATCCTGTTTGCTCCCCACGC
TTTCGCGCCTCAGCGTCAGTTACAGACCAGAAAGCCGCCTTCGCCACTGGTGTTCCTCCAAATCTCTACGCATTTCACCGCTACACTTGGAATTCCGCTT
TCCTCTTCTGCACTCAAGTCCCCCAGTTTCCAATGACCCTCCACGGTTGAGCCGTGGGCTTTCACATCAGACTTAAGGGACCGCCTGCGCGCGCTTTACG
CCCAATGATTCCGGACAACGCTTGCCACCTACGTATTACCGCGGCTGCTGGCACGTAGTTAGCCGTGGCTTTCTAATGAGGTACCGTCAAGGTACCGGCA
GTTACTCCGGTACTTGTTCTTCCCTCACAACAGAGTTTTACGATCCGAGAACCTTCTTCACTCACGCGGCGTTGCTCCATCAGACTTTCGTCCATTGTGG
AAGATTCCCTACTGCTGCCTCCCGTAGGAGTCTGGGCCGTGTCTCAGTCCCAGTGTGGCCGATCACCCTCTCAGGTCGGCTACGCATCGTCGCCTTGGTG
GGCCGTTACCCCACCAACTAGCTAATGCGCCGCGGGCCCATCCTGTAGTGACAGCCGAAGCCGCCTTTCAACACCGGACCATGTGGTCTGGTGGATTATT
CGGTATTAGCCCCGGTTTCCCGGAGTTATCCCAATCTACAGGGCAGGTTGCCCACGTGTTACTCACCCGTCCGCCGCTGAATCATGGAGGAGCAAGCTCC
TCCTTCATCCGCTCGACTGCATGT

>EB18
TCTGCCCTGCACTCTGGGACAAGCCCTGGAAACGGGGTCTAATACCGGATACTGATCATCTTGGGCATCCTTGGTGATCGAAAGCTCCGGCGGTGCAGGA
TGAGCCCGCGGCCTATCAGCTTGTTGGTGAGGTAATGGCTCACCAAGGCGACGACGGGTAGCCGGCCTGAGAGGGCGACCGGCCACACTGGGACTGAGAC
ACGGCCCAGACTCCTACGGGAGGCAGCAGTGGGGAATATTGCACAATGGGCGAAAGCCTGATGCAGCGACGCCGCGTGAGGGATGACGGCCTTCGGGTTG
TAAACCTCTTTCAGCAGGGAAGAAGCGAAAGTGACGGTACCTGCAGAAGAAGCGCCGGCTAACTACGTGCCAGCAGCCGCGGTAATACGTAGGGCGCGAG
CGTTGTCCGGAATTATTGGGCGTAAAGAGCTCGTAGGCGGCTTGTCGCGTCGGTTGTGAAAGCCCGGGGCTTAACCCCGGGTCTGCAGTCGATACGGGCA
GGCTAGAGTTCGGTAGGGGAGATCGGAATTCCTGGTGTAGCGGTGAAATGCGCAGATATCAGGAGGAACACCGGTGGCGAAGGCGGATCTCTGGGCCGAT
ACTGACGCTGAGGAGCGAAAGCGTGGGGAGCGAACAGGATTAGATACCCTGGTAGTCCACGCCGTAAACGGTGGGCACTAGGTGTGGGCGACATTCCACG
TCGTCCGTGCCGCAGCTAACGCATTAAGTGCCCCGCCTGGGGAGTACGGCCGCAAGGCTAAAACTCAAAGGAATTGACGGGGGCCCGCACAAGCGGCGGA
GCATGTGGCTTAATTCGACGCAACGCGAAGAACCTTACCAAGGCTTGACATACACCGGAAAACCCTGGAGACAGGGTCCCCCTTGTGGTCGGTGTACAGG
TGGTGCATGGCTGTCGTCAGCTCGTGTCGTGAGATGTTGGGTTAAGTCCCGCAACGAGCGCAACCCTTGTCCCGTGTTGCCAGCAGGCCCTTGTGGTGCT
GGGGACTCACGGGAGACCGCCGGGGTCAACTCGGAGGAAGGTGGGGACGACGTCAAGTCATCATGCCCCTTATGTCTTGGGCTGCACACGTGCTACAATG

GCCGGTACAATGAGCTGCGATACCGCGAGGTGGAGCGAATCTCAAAAAGCCGGTCTCAGTTCGGATTGGGGTCTGCAACTCGACCCCATGAAGTCGGAGT
CGCTAGTAATCGCAGATCAGCATTGATGCGGTGAATACGTTCCCGGGCTTGGTACACACCGCCCGTCA

>EB19
TGCAGTCGAGCGGATGAAGGTAGCTTGCTACTGGATTCAGCGGCGGACGGGTGAGTAATGCTTAGGAATCTGCCTATTAGTGGGGGACAACGTTCCGAAA
GGAGCGCTAATACCGCATACGTCCTACGGGAGAAAGCAGGGGACCTTTGGGCCTTGCGCTAATAGATGAGCCTAAGTCGGATTAGCTAGTTGGTAGGGTA
AAGGCCTACCAAGGCGACGATCTGTAGCGGGTCTGAGAGGATGATCCGCCACACTGGGACTGAGACACGGCCCAGACTCCTACGGGAGGCAGCAGTGGGG
AATATTGGACAATGGGGGGAACCCTGATCCAGCCATGCCGCGTGTGTGAAGAAGGCCTTTTGGTTGTAAAGCACTTTAAGCGAGGAGGAGGCTACCTAGA
TTAATACTTTAGGATAGTGGACGTTACTCGCAGAAATAAGCACCGGCTAACTCTGTGCCAGCAGCCGCGGTAATACAGAGGGTGCGAGCGTTAATCGGAT
TTACTGGGCGTAAAGCGTGCGTAGGCGGCCAATTAAGTCAAATGTGAAATCCCCGAGCTTAACTTGGGAATTGCATTCGATACTGGTTGGCTAGAGTATG
GGAGAGGATGGTAGAATTCCAGGTGTAGCGGTGAAATGCGTAGAGATCTGGAGGAATACCGATGGCGAAGGCAGCCATCTGGCCTAATACTGACGCTGAG
GTACGAAAGCATGGGGAGCAAACAGGATTAGATACCCTGGTAGTCCATGCCGTAAACGATGTCTACTAGCCGTTGGGGCCCTTGAGGCTTTAGTGGCGCA
GCTAACGCGATAAGTAGACCGCCTGGGGAGTACGGTCGCAAGACTAAAACTCAAATGAATTGACGGGGGCCCGCACAAGCGGTGGAGCATGTGGTTTAAT
TCGATGCAACGCGAAGAACCTTACCTGGCCTTGACATACAGAGAACTTTCCAGAGATGGATTGGTGCCTTCGGGAACTCTGATACAGGTGCTGCATGGCT
GTCGTCAGCTCGTGTCGTGAGATGTTGGGTTAAGTCCCGCAACGAGCGCAACCCTTTTCCTTATTTGCCAGCACTTCGGGTGGGAACTTTAAGGATACTG
CCAGTGACAAACTGGAGGAAGGCGGGGACGACGTCAAGTCATCATGGCCCTTACGGCCAGGGCTACACACGTGCTACAATGGTCGGTACAAAGGGTTGCT
ACACAGCGATGTGATGCTAATCTCAAAAAGCCGATCGTAGTCCGGATTGGAGTCTGCAACTCGACTCCATGAAGTCGGAATCGCTAGTAATCGCGGATCA
GAATGCCGCGGTGAATACGTTCCCGGGCCTTGTACACACCGCCCGTCACACCATGGGAGTTTGTTGCACCAGAAGTAGGTAGTCTAACCGCAAGGAGGAC
GC

>EB20
GTCGAGCGGAGAGAGGTAGCTTGCTACTGATCTTAGCGGCGGACGGGTGAGTAATGCTTAGGAATCTGCCTATTAGTGGGGGACAACATTTCGAAAGGAA
TGCTAATACCGCATACGTCCTACGGGAGAAAGCAGGGGATCTTCGGACCTTGCGCTAATAGATGAGCCTAAGTCGGATTAGCTAGTTGGTGGGGTAAAGG
CCTACCAAGGCGACGATCTGTAGCGGGTCTGAGAGGATGATCCGCCACACTGGGACTGAGACACGGCCCAGACTCCTACGGGAGGCAGCAGTGGGGAATA
TTGGACAATGGGCGGAAGCCTGATCCAGCCATGCCGCGTGTGTGAAGAAGGCCTTATGGTTGTAAAGCACTTTAAGCGAGGAGGAGGCTACTTTAGTTAA
TACCTAGAGATAGTGGACGTTACTCGCAGAATAAGCACCGGCTAACTCTGTGCCAGCAGCCGCGGTAATACAGAGGGTGCAAGCGTTAATCGGATTTACT
GGGCGTAAAGCGCGCGTAGGCGGCTAATTAAGTCAAATGTGAAATCCCCGAGCTTAACTTGGGAATTGCATTCGATACTGGTTAGCTAGAGTGTGGGAGA
GGATGGTAGAATTCCAGGTGTAGCGGTGAAATGCGTAGAGATCTGGAGGAATACCGATGGCGAAGGCAGCCATCTGGCCTAACACTGACGCTGAGGTGCG
AAAGCATGGGGAGCAAACAGGATTAGATACCCTGGTAGTCCATGCCGTAAACGATGTCTACTAGCCGTTGGGGCCTTTGAGGCTTTAGTGGCGCAGCTAA
CGCGATAAGTAGACCGCCTGGGGAGTACGGTCGCAAGACTAAAACTCAAATGAATTGACGGGGGCCCGCACAAGCGGTGGAGCATGTGGTTTAATTCGAT
GCAACGCGAAGAACCTTACCTGGCCTTGACATAGTAAGAACTTTCCAGAGATGGATTGGTGCCTTCGGGAACTTACATACAGGTGCTGCATGGCTGTCGT
CAGCTCGTGTCGTGAGATGTTGGGTTAAGTCCCGCAACGAGCGCAACCCTTTTCCTTATTTGCCAGCGAGTAATGTCGGGAACTTTAAGGATACTGCCAG
TGACAAACTGGAGGAAGGCGGGGACGACGTCAAGTCATCATGGCCCTTACGGCCAGGGCTACACACGTGCTACAATGGTCGGTACAAAGGGTTGCTACCT
AGCGATAGGATGCTAATCTCAAAAAGCCGATCGTAGTCCGGATTGGAGTCTGCAACTCGACTCCATGAAGTCGGAATCGCTAGTAATCGCGGATCAGAAT
GCCGCGGTGAATACGTTCCCGGGCCTTGTACACACCGCCCGTCACACCATGGGAGTTTGTTGCACCAGAAGTAGCTAGCCTAACTGCAAAGAGGGCGG

>EB21
TGCAAGTCGAACGGTGACCCGGTGCTTGCACCGGTGATCAGTGGCGAACGGGTGAGTAACACGTGAGTAACCTGCCCCAGACTCTGGGATAACCCCGAGA
AATCGGAGCTAATACCGGATACGAGACGCACAGGCATCTGCAGCGTCTGGAAAGAATTTCGGTCTGGGATGGACTCGCGGCCTATCAGCTAGTTGGTGAG
GTAACGGCCCACCAAGGCGACGACGGGTAGCCGGCCTGAGAGGGCGACCGGCCACACTGGGACTGAGACACGGCCCAGACTCCTACGGGAGGCAGCAGTG
GGGAATATTGCACAATGGGCGCAAGCCTGATGCAGCGACGCCGCGTGGGGGATGAAGGCCTTCGGGTTGTAAACCCCTTTCAGCAGGGAAGAAGCGAAAG
TGACGGTACCTGCAGAAGAAGCGCCGGCTAACTACGTGCCAGCAGCCGCGGTAATACGTAGGGCGCAAGCGTTGTCCGGAATTATTGGGCGTAAAGAGCT
CGTAGGCGGTTTGTCGCGTCTGCTGTGAAAACCCGAGGCTCAACCTCGGGCCTGCAGTGGGTACGGGCAGACTAGAGTGCGGTAGGGGAGACTGGAATTC
CTGGTGTAGCGGTGGAATGCGCAGATATCAGGAGGAACACCGATGGCGAAGGCAGGTCTCTGGGCCGCAACTGACGCTGAGGAGCGAAAGCATGGGGAGC
GAACAGGATTAGATACCCTGGTAGTCCATGCCGTAAACGTTGGGCACTAGGTGTGGGGCTCATTCCACGAGTTCCGTGCCGCAGCAAACGCATTAAGTGC
CCCGCCTGGGGAGTACGGCCGCAAGGCTAAAACTCAAAGGAATTGACGGGGGCCCGCACAAGCGGCGGAGCATGCGGATTAATTCGATGCAACGCGAAGA
ACCTTACCAAGGCTTGACATATACCGAAAACTCATGGAGACATGGGGTCCGCAAGGGCGGTATACAGGTGGTGCATGGTTGTCGTCAGCTCGTGTCGTGA
GATGTTGGGTTAAGTCCCGCAACGAGCGCAACCCTCGTCCTATGTTGCCAGCACATCATGGTGGGGACTCATAGGAGACTGCCGGGGTCAACTCGGAGGA
AGGTGGGGATGACGTCAAATCATCATGCCCCTTATGTCTTGGGCTTCACGCATGCTACAATGGCCGGTACAAAGGGCTGCGATACCGCAAGGTGGAGCGA
ATCCCAAAAAGCCGGTCTCAGTTCGGATTGGGGTCTGCAACTCGACCCCATGAAGTCGGAGTCGCTAGTAATCGCAGATCAGCAACGCTGCGGTGAATAC
GTTCCCGGGCCTTGTACACACCGCCCGTCAAGTCACGAAAGTCGGTAACACCCGAAGCCGGTGGCCCAACCCTTGGGAGGGAGCCGTCG

>EB22
CGGTGATCTCGGTGCTTGCACCGGGTGATCAGTGGCGAACGGGTGAGTAACACGTGAGTAACCTGCCCCCGACTCTGGGATAACCCCGGGAAATCGGAGC
TAATACCGGATACGAGACGCACAGGCATCTGTAGCGTCTGGAAAGAATTTCGGTCGGGGATGGACTCGCGGCCTATCAGCTTGTTGGTGGGGTAACGGCC
CACCAAGGCGACGACGGGTAGCCGGCCTGAGAGGGCGACCGGCCACACTGGGACTGAGACACGGCCCAGACTCCTACGGGAGGCAGCAGTGGGGAATATT
GCACAATGGGCGCAAGCCTGATGCAGCGACGCCGCGTGGGGGATGAAGGCCTTCGGGTTGTAAACCCCTTTCAGCAGGGAAGAAGCGCAAGTGACGGTAC
CTGCAGAAGAAGCGCCGGCTAACTACGTGCCAGCAGCCGCGGTAATACGTAGGGCGCAAGCGTTGTCCGGAATTATTGGGCGTAAAGAGCTCGTAGGCGG
TTTGTCGCGTCTGCTGTGAAAACCCGAGGCTCAACCTCGGGCCTGCAGTGGGTACGGGCAGACTAGAGTGCGGTAGGGGAGACTGGAATTCCTGGTGTAG
CGGTGGAATGCGCAGATATCAGGAGGAACACCGATGGCGAAGGCAGGTCTCTGGGCCGCAACTGACGCTGAGGAGCGAAAGCATGGGGAGCGAACAGGAT
TAGATACCCTGGTAGTCCATGCCGTAAACGTTGGGCACTAGGTGTGGGGCTCATTCCACGAGTTCCGTGCCGCAGCAAACGCATTAAGTGCCCCGCCTGG
GGAGTACGGCCGCAAGGCTAAAACTCAAAGGAATTGACGGGGGGCCCGCACAAGCGGCGGAGCATGCGGATTAATTCGATGCAACGCGAAGAACCTTACC
AAGGCTTGACATACACCGGTAACGGCCAGAGATGGTCGCCCCGCAAGGTCGGTGTACAGGTGGTGCATGGTTGTCGTCAGCTCGTGTCGTGAGATGTTGG
GTTAAGTCCCGCAACGAGCGCAACCCTCGTCCCATGTTGCCAGCGGGTTATGCCGGGGACTCATGGGAGACTGCCGGGGTCAACTCGGAGGAAGGTGGGG
ATGACGTCAAATCATCATGCCCCTTATGTCTTGGGCTTCACGCATGCTACAATGGCCGGTACAAAGGGCTGCGATACCGCGAGGTGGAGCGAATCCCAAA
AAGCCGGTCTCAGTTCGGATTGGGGTCTGCAACTCGACCCCATGAAGTCGGAGTCGCTAGTAATCGCAGATCAGCAACGCTGCGGTGAATACGTTCCCGG
GCCTTGTACACACCGCCCGTCAAGTCACGAAAGTCGGTAACACCCGAAGCCAGTGGCCCAACCCTTGTGGGGGGAG

>EB23
TGCAGTCGAACGCCCCGCAAGGGGAGTGGCAGACGGGTGAGTAACGCGTGGGAACATACCCTTTTCTACGGAATAGCTCTGGGAAACTGGAATTAATACC
GTATACGCCCTACGGGGGAAAGATTTATCGGGGAAGGATTGGCCCGCGTTGGATTAGCTAGTTGGTGGGGTAAAGGCCTACCAAGGCGACGATCCATAGC
TGGTCTGAGAGGATGATCAGCCACATTGGGACTGAGACACGGCCCAAACTCCTACGGGAGGCAGCAGTGGGGAATATTGGACAATGGGCGCAAGCCTGAT
CCAGCCATGCCGCGTGAGTGATGAAGGCCTTAGGGTTGTAAAGCTCTTTCACCGATGAAGATAATGACGGTAGTCGGAGAAGAAGCCCCGGCTAACTTCG
TGCCAGCAGCCGCGGTAATACGAAGGGGGGCTAGCGTTGTTCGGAATTACTGGGCGTAAAGCGCACGTAGGCGGATATTTAAGTCAGGGGTGAAATCCCA
GAGCTCAACTCTGGAACTGCCTTTGATACTGGGTATCTTGAGTATGGAAGAGGTAAGTGGAATTGCGAGTGTAGAGGTGAAATTCGTAGATATTCGCAGG
AACACCAGTGGCGAAGGCGGCTTACTGGTCCATTACTGACGCTGAGGTGCGAAAGCGTGGGGAGCAAACAGGATTAGATACCCTGGTAGTCCACGCCGTA
AACGATGAATGTTAGCCGTCGGGCAGTATACTGTTCGGTGGCGCAGCTAACGCATTAAACATTCCGCCTGGGGAGTACGGTCGCAAGATTAAAACTCAAA
GGAATTGACGGGGGCCCGCACAAGCGGTGGAGCATGTGGTTTAATTCGAAGCAACGCGCAGAACCTTACCAGCTCTTGACATTCGGGGTATGGTCATTGG

AGACGATGACCTTCAGTTCGGCTGGCCCTAGAACAGGTGCTGCATGGCTGTCGTCAGCTCGTGTCGTGAGATGTTGGGTTAAGTCCCGCAACGAGCGCAA
CCCTCGCCCTTAGTTGCCAGCATTGAGTTGGGCACTCTAAGGGGACTGCCGGTGATAAGCCGAGAGGAAGGTGGGGATGACGTCAAGTCCTCATGGCCCT
TACGGGCTGGGCTACACACGTGCTACAATGGTGGTGACAGTGGGCAGCGAGACAGCGATGTCGAGCTAATCTCCAAAAGCCATCTCAGTTCGGATTGCAC
TCTGCAACTCGAGTGCATGAAGTTGGAATCGCTAGTAATCGCAGATCAGCATGCTGCGGTGAATACGTTCCCGGGCCTTGTACACACCGCCCGTCACACC
ATGGGAGTTGGTTTTACCCGAAGGCGCTGCGCTAACCGCAAGGGGCAGGCGACC

>EB24
CGCCTGCCCCCTTGCGGTTAGCGCAGCGCCTTCGGGTAAAACCAACTCCCATGGTGTGACGGGCGGTGTGTACAAGGCCCGGGAACGTATTCACCGCAGC
ATGCTGATCTGCGATTACTAGCGATTCCAACTTCATGCACTCGAGTTGCAGAGTGCAATCCGAACTGAGATGGCTTTTGGAGATTAGCTCGACATCGCTG
TCTCGCTGCCCACTGTCACCACCATTGTAGCACGTGTGTAGCCCAGCCCGTAAGGGCCATGAGGACTTGACGTCATCCCCACCTTCCTCTCGGCTTATCA
CCGGCAGTCCCCTTAGAGTGCCCAACTAAAATGCTGGCAACTAAGGGCGAGGGTTGCGCTCGTTGCGGGACTTAACCCAACATCTCACGACACGAGCTGA
CGACAGCCCATGCAGCACCTGTTCTAGGGCCAGCCGAACTGAAGGTCATCGTCTCCAATGACCATACCCCGAATGTCAAGAGCTGGTAAGGTTCTGCGCG
TTGCTTCGAATTAAACCACATGCTCCACCGCTTGTGCGGGCCCCCGTCAATTCCTTTGAGTTTTAATCTTGCGACCGTACTCCCCAGGCGGAATGTTTAA
TGCGTTAGCTGCGCCACCGAACAGTATACTGCCCGACGGCTAACATTCATCGTTTACGGCGTGGACTACCAGGGTATCTAATCCTGTTTGCTCCCCACGC
TTTCGCACCTCAGCGTCAGTAATGGACCAGTAAGCCGCCTTCGCCACTGGTGTTCCTGCGAATATCTACGAATTTCACCTCTACACTCGCAATTCCACTT
ACCTCTTCCATACTCAAGATACCCAGTATCAAAGGCAGTTCCAGAGTTGAGCTCTGGGATTTCACCCCTGACTTAAATATCCGCCTACGTGCGCTTTACG
CCCAGTAATTCCGAACAACGCTAGCCCCCTTCGTATTACCGCGGCTGCTGGCACGAAGTTAGCCGGGGCTTCTTCTCCGACTACCGTCATTATCTTCATC
GGTGAAAGAGCTTTACAANCCCTAAGGCCTTCATCACTCACGCGGCATGGCTGGATCAGGCTTGCGCCCATTGTCCAATATTCCCCACTGCTGCCTCCCG
TAGGAGTTTGGGCCGTGTCTCAGTCCCAATGTGGCTGATCATCCTCTCAGACCAGCTATGGATCGTCGCCTTGGTAGGCCTTTACCCCACCAACTAGCTA
ATCCAACGCGGGCCAATCCTTCCCCGATAAATCTTTCCCCCGTAGGGCGTATACGGTATTAATTCCAGTTTCCCAGAGCTATTCCGTAGAAAAGGGTATG
TTCCCACGCGTTACTCACCCGTCTGCCACTCCCCTTGCGGGGCGTTCGACTGCA

>EB25
TTCGACGGCTCCTCCACAAGGGTTAGGCCACCGGCTTCAGGTGTTACCGACTTTCATGACTTGACGGGCGGTGTGTACAAGACCCGGGAACGTATTCACC
GCAGCGTTGCTGATCTGCGATTACTAGCGACTCCGACTTCATGAGGTCGAGTTGCAGACCTCAATCCGAACTGGGACCGGCTTTTTGGGATTCGCTCCAC
CTCACGGTATTGCAGCCCTTTGTACCGGCCATTGTAGCATGCGTGAAGCCCAAGACATAAGGGGCATGATGATTTGACGTCATCCCCACCTTCCTCCGAG
TTGACCCCGGCAGTATCCCATGAGTTCCCACCATTACGTGCTGGCAACATAGAACGAGGGTTGCGCTCGTTGCGGGACTTAACCCAACATCTCACGACAC
GAGCTGACGACAACCATGCACCACCTGTTTACGAGTGTCCAAAGAGTTGACCATTTCTGGCCCGTTCTCGTATATGTCAAGCCTTGGTAAGGTTCTTCGC
GTTGCATCGAATTAATCCGCATGCTCCGCCGCTTGTGCGGGTCCCCGTCAATTCCTTTGAGTTTTAGCCTTGCGGCCGTACTCCCCAGGCGGGGAACTTA
ATGCGTTAGCTGCGTCACGGAATCCGTGGAATGGACCCCACAACTAGTTCCCAACGTTTACGGGGTGGACTACCAGGGTATCTAAGCCTGTTTGCTCCCC
ACCCTTTCGCTCCTCAGCGTCAGTTACGGCCCAGAGATCTGCCTTCGCCATCGGTGTTCCTCCTGATATCTGCGCATTCCACCGCTACACCAGGAATTCC
AATCTCCCCTACCGCACTCTAGTCTGCCCGTACCCACTGCAGGCCCGAGGTTGAGCCTCGGGATTTCACAGCAGACGCGACAAACCGCCTACGAGCTCTT
TACGCCCAATAATTCCGGATAACGCTTGCGCCCTACGTATTACCGCGGCTGCTGGCACGTAGTTAGCCGGCGCTTTTTCTGCAGGTACCGTCACTTTCGC
TTCTTCCCTGCTAAAAGAGGTTTACAACCCGAAGGCCGTCATCCCTCACGCGGCGTTGCTGCATCAGGCTTTCGCCCATTGTGCAATATTCCCCACTGCT
GCCTCCCGTAGGAGTCTGGGCCGTGTCTCAGTCCCAGTGTGGCCGGTCACCCTCTCAGGCCGGCTACCCGTCGACGCCTTGGTGAGCCATTACCTCACCA
ACAAGCTGATAGGCCGCGAGCCCATCCCCAACCGAAAAATCTTTCCAAACGCAGACCATGCGGTCACGTCACATATCCAGTATTAGACGCCGTTTCCAGC
GCTTATCCCAGAGTCAGGGGCAGGTTGCTCACGTGTTACTCACCCGTTCGCCACTGATCCCACAGAGCAAGCTCCGTGTTCACCGTTCGACTGCA

>EB26
TCCGTGAGGTTAGGCCACTGGCTTCGGGTGTTACCGACTTTCATGACTTGACGGGCGGTGTGTACAAGGGCCGGGAACGTATTCACCGTAGCGTTGCTGA
TCTACGATTACTAGCGACTCCGACTTCATGAGGTCGAGTTGCAGACCTCAATCCGAACTGAGACCGGCTTTTTGGGATTCGCTCCACCTTACGGTATTGC
AGCCCATTGTACCGGCCATTGTAGCATGCGTGAAGCCCAAGACATAAGGGGCATGATGATTTGACCTCATCCCCACCTTCCTCCGAGTTGACCCCGGCAG
TATCCCATGAGTTCCCACCATTACGTGCTGGCAACATAGGACGAGGGTTGCGCTCGTTGCGGGACTTAACCCCAACATCTCACGACACGAGCTGACGACA
ACCATGCACCACCTGTATACGAGTGTCCAAAGAGTTCGCTGTCTCCAGCGCGTTCTCGTATATGTCAAGCCTTGGTAAGGTTCTTCGCGTTGCATCGAAT
TAATCCGCATGCTCCGCCGCTTGTGCGGGCCCCCGTCAATTCCTTTGAGTTTTAGCCTTGCGGCCGTACTCCCCAGGCGGGGAACTTAATGCGTTAGCTA
CGACACGGAGACCGTGGAATGGTCCCCACATCTAGTTCCCAACGTTTACGGCATGGACTACCAGGGTATCTAAGCCTGTTTGCTCCCCATGCTTTCGCTC
CTCAGCGTCAGTTACGGCCCAGAGATCTGCCTTCGCCATCGGTGTTCCTCCTGATATCTGCGCATTCCACCGCTACACCAGGAATTCCAATCTCCCCTAC
CGCACTCTAGTCTGCCCGTACCCACTGCAAGCCCGAGGTTGAGCCTCGGGTTTTCACAGCAGACGTGACAAACCGCCTACGAGCTCTTTACGCCCAATAA
TTCCGGACAACGCTTGCACCCTACGTATTACCGCGGCTGCTGGCACGTAGTTAGCCGGTGCTTTTTCTGCAGGTACCGTCACTTTCGCTTCTTCCCTGCT
AAAAGAGGTTTACAACCCGAAGGCCTTCGTCCCTCACGCGGCGTTGCTGCATCAGGCTTGCGCCCATTGTGCAATATTCCCCACTGCTGCCTCCCGTAGG
AGTCTGGGCCGTGTCTCAGTCCCAGTGTGGCCGGTCACCCTCTCAGGCCGGCTACCCGTCGTCGTCTTGGTGAGCCATTACCTCACCAACTAACTGATAG
GCCGTGAGCTGATCCTTGACCGAAGTTCTTTCCACGACCAGGAGATGCCTCCGATCGTCGTATCCGGTATTAGACGCCGTTTCCAGCGCTTATCCCAGAG
TCAAGGGCACATTGCTCACGTATTACTCACCCGTTCGCCACTAATCAGGAGGAGCAAGCTCCTCCGTCAT

>EB27
GATAATGGTTGGATACGACGGATAATGCCGAATATGAACTCCTTTCGCATGTTTGGAGGTGGAAAGCTCCGGCGGAGAAGGATGAGCTCGCGGCCTATCA
GCTTGTTGGTGAGGTAATGGCTCACCAAGGCATTGACGGGTAGCCGGCCTGAGAGGGTGACCGGCCACACTGGGACTGAGACACGGCCCAGACTCCTACG
GGAGGCAGCAGTGGGGAATATTGGACAATRGGCGAAANGCCKRAKCCAGCAACGCCGCGNTGAGGGATGACGGCCTTCGGGTTGTAAACCTCTTTCAGCA
GGGACGAAGCGAAAGTGACGGTACCTGCAGAAGAAGGACCGGCCAACTACGTGCCAGCAGCCGCGGTAATACGTAGGGTCCGAGCGTTGTCCGGAATTAT
TGGGCGTAAAGGGCTCGTAGGCGGTTTGTCGCGTCGGNAGTGAAAACTCAGGGCTCAACCCTGAGCGTGCTTCCGATACGGGCAGACTAGAGGTATGCAG
GGGAGAACGGAATTCCTGGTGTAGCGGTGGAATGCGCAGATATCAGGAGGAACACCGGTGGCGAAGGCGGTTCTCTGGGCATTACCTGACGCTGAGGAGC
GAAAGCATGGGGAGCGAACAGGATTAGATACCCTGGTAGTCCATGCCGTAAACGTTGGGCGCTAGGTGTGGGGACCTTCCACGGTCTCCGTGCCGCAGCT
AACGCATTAAGCGCCCCGCCTGGGGAGTACGGCCGCAAGGCTAAAACTCAAAGGAATTGACGGGGGCCCGCACAAGCGGCGGAGCATGCTGATTAATTCG
ATGCAACGCGAAGAACCTTACCTGGGTTTGACATATACCGGAAAGCTGCAGAGATGTAGCCCCCTTTTGGTCGGTATACAGGTGGTGCATGGCGTCGTCA
GCTCGTGTCGTGAGATGTTGGGTTAAGTCCCGCAACGAGCGCAACCCTCGTCCTATGTTGCCAGCACGTCATGGTGGGGACTCATAGGAGACTGCCGGGG
TCAACTCGGAGGAAGGTGGGGATGACGTCAAGTCTTCATGCCCCTTATGTCCAGGGCTTCAAGCATGCTACAATGGCCGGTACAAAGGGCTGCGAAACCG
CAAGGTGGAGCGAATCCCAAAAAGCCGGTCTCAGTTCGGATTGGGGTCTGCAACTCGACCCCATGAAGTCGGAGTCGCTGGTAATCGCAGATCAGCAACG
GTGCGGTGAATACGTTCCCGGGCCTATACACACCGCCCTCCATATCTTGAAACTCGGCAACACCAGAGT

>EB28
GGCTTCGGGTGTTACCGACTTTCATGACTTGACGGGCGGTGTGTACAAGGCCCGGGAACGTATTCACCGTAGCGTTGCTGATCTACGATTACTAGCGACT
CCGACTTCATGAGGTCGAGTTGCAGACCTCAATCCGAACTGAGACCGGCTTTTTGGGATTCGCTCCACCTTACGGTATTGCAGCCCATTGTACCGGCCAT
TGTAGCATGCGTGAAGCCCAAGACATAAGGGGCATGATGATTTGACCTCATCCCCACCTTCCTCCGAGTTGACCCCGGCAGTATCCCATGAGTTCCCACC
ATTACGTGCTGGCAACATAGGACGAGGGTTGCGCTCGTTGCGGGACTTAACCCAACATCTCACGACACGAGCTGACGACAACCATGCACCACCTGTATAC
GAGTGTCCAAAGAGTTTCACTGTCTCCAGTGCGTTCTCGTATATGTCAAGCCTTGGTAAGGTTCTTCGCGTTGCATCGAATTAATCCGCATGCTCCGCCG
CTTGTGCGGGCCCCCCGTCAATTCCTTTGAGTTTTAGCCTTGCGGCCGTACTCCCCAGGCGGGGAACTTAATGCGTTAGCTACGACACGGAGACCGTGGA
ATGGTCCCCACATCTAGTTCCCAACGTTTACGGCATGGACTACCAGGGTATCTAAGCCTGTTTGCTCCCCATGCTTTCGCTCCTCAGCGTCAGTTACGGC
CCAGAGATCTGCCTTCGCCATCGGTGTTCCTCCTGATATCTGCGCATTCCACCGCTACACCAGGAATTCCAATCTCCCCTACCGCACTCTAGTCTGCCCG

TACCCACTGCAAGCCCGAGGTTGAGCCTCGGGTTTTCACAGCAGACGTGACAAACCGCCTACGAGCTCTTTACGCCCAATAATTCCGGACAACGCTTGCA
CCCTACGTATTACCGCGGCTGCTGGCACGTAGTTAGCCGGTGCTTTTTCTGCAGGTACCGTCACTTTCGCTTCTTCCCTGCTAAAAGAGGTTTACAACCC
GAAGGCCTTCGTCCCTCACGCGGCGTTGCTGCATCAGGCTTGCGCCCATTGTGCAATATTCCCCACTGCTGCCTCCCGTAGGAGTCTGGGCCGTGTCTCA
GTCCCAGTGTGGCCGGTCACCCTCTCAGGCCGGCTACCCGTCGTCGTCTTGGTGAGCCATTACCTCACCAACTAACTGATAGGCCGTGAGCTGATCCTTG
ACCGAAGTTCTTTCCACGACCAGGAGATGCCTCCGATCGTCGTATCCGGTATTAGACGCCGTTTCCAGCGCTTATCCCAGAGTCAAGGGCACATTGCTCA
CGTATTACTCACCCGTTCGCCACTAATCAGGAGGAGCAAGCTCCTCCGTCATCGTTCGACTGCA

>EB29
TGCAAGTCGAACGATGATGGGAGCTTGCTCCTGGATTAGTGGCGAACGGGTGAGTAACACGTGAGTAACCTGCCCTTAACTCTGGGATAAGCCTGGGAAA
CTGGGTCTAATACCGGATATGACTCCTCATCGCATGGTGGGGGGTGGAAAGCTTTTTGTGGTTTTGGATGGACTCCCGGCCTATCAGCTTGTTGGTGAGG
TAATGGCTTACCAAGGCGACGACGGGTAGCCGGCCTGAGAGGGTGACCGGCCACACTGGGACTGAGACACGGCCCAGACTCCTACGGGAGGCAGCAGTGG
GGAATATTGCACAATGGGCGCAAGCCTGATGCAGCGACGCCGCGTGAGGGATGAAGGCCTTCGGGTTGTAAACCTCTTTCAGTAGGGAAGAAGCGAAAGT
GACGGTACCTGCAGAAGAAGCGCCGGCTAACTACGTGCCAGCAGCCGCGGTAATACGTAGGGCGCAAGCGTTATCCGGAATTATTGGGCGTAAAGAGCTC
GTAGGCGGTTTGTCGCGTCTGCCGTGAAAGTCCGGGGCTCAACTCCGGATCTGCGGTGGGTACGGGCAGACTAGAGTGATGTAGGGGAGACTGGAATTCC
TGGTGTAGCGGTGAAATGCGCAGATATCAGGAGGAACACCGATGGCGAAGGCAGGTCTCTGGGCATTAACTGACGCTGAGGAGCGAAAGCATGGGGAGCG
AACAGGATTAGATACCCTGGTAGTCCATGCCGTAAACGTTGGGCACTAGGTGTGGGGGACATTCCACGTTTTCCGCGCCGTAGCTAACGCATTAAGTGCC
CCGCCTGGGGAGTACGGCCGCAAGGCTAAAACTCAAAGGAATTGACGGGGGCCCGCACAAGCGGCGGAGCATGCGGATTAATTCGATGCAACGCGAAGAA
CCTTACCAAGGCTTGACATGAACCGGTAATACCTGGAGACAGGTGCCCCGCTTGCGGTCGGTTTACAGGTGGTGCATGGTTGTCGTCAGCTCGTGTCGTG
AGATGTTGGGTTAAGTCCCGCAACGAGCGCAACCCTCGTTCTATGTTGCCAGCACGTGATGGTGGGGACTCATAGGAGACTGCCGGGGTCAACTCGGAGG
AAGGTGGGGACGACGTCAAATCATCATGCCCCTTATGTCTTGGGCTTCACGCATGCTACAATGGCCGGTACAAAGGGTTGCGATACTGTGAGGTGGAGCT
AATCCCAAAAAGCCGGTCTCAGTTCGGATTGGGGTCTGCAACTCGACCCCATGAAGTCGGAGTCGCTAGTAATCGCAGATCAGCAACGCTGCGGTGAATA
CGTTCCCGGGCCTTGTACACACCGCCCGTCAAGTCACGAAAGTTGGTAACACCCGAAGCCGGTGGCCTAACCCCTTGTGGGA

>EB30
TGCAAGTCGAGCGGCAGCGGGAGGAAGCTTGCTTCCTCGCCGGCGAGCGGCGGACGGGTGAGTAATGTCTGGGGATCTGCCCGATGGAGGGGGATAACCA
CTGGAAACGGTGGCTAATACCGCATAACGTCGCAAGACCAAAGTGGGGGACCTTCGGGCCTCACACCATCGGATGAACCCAGATGGGATTAGCTAGTAGG
TGGGGTAATGGCTCACCTAGGCGACGATCCCTAGCTGGTCTGAGAGGATGACCAGCCACACTGGAACTGAGACACGGTCCAGACTCCTACGGGAGGCAGC
AGTGGGGAATATTGCACAATGGGCGCAAGCCTGATGCAGCCATGCCGCGTGTGTGAAGAAGGCCTTCGGGTTGTAAAGCACTTTCAGCGAGGAGGAAGGT
GGTGAGCTTAATACGTTCATCAATTGACGTTACTCGCAGAAGAAGCACCGGCTAACTCCGTGCCAGCAGCCGCGGTAATACGGAGGGTGCAAGCGTTAAT
CGGAATTACTGGGCGTAAAGCGCACGCAGGCGGTTTGTTAAGTCAGATGTGAAATCCCCGAGCTTAACTTGGGAACTGCATTTGAAACTGGCAAGCTAGA
GTCTCGTAGAGGGGGGTAGAATTCCAGGTGTAGCGGTGAAATGCGTAGAGATCTGGAGGAATACCGGTGGCGAAGGCGGCCCCCTGGACGAAGACTGACG
CTCAGGTGCGAAAGCGTGGGGAGCAAACAGGATTAGATACCCTGGTAGTCCACGCCGTAAACGATGTCGATTTGGAGGTTGTGCCCTTGAGGCGTGGCTT
CCGGAGCTAACGCGTTAAATCGACCGCCTGGGGAGTACGGCCGCAAGGTTAAAACTCAAATGAATTGACGGGGGCCCGCACAAGCGGTGGAGCATGTGGT
TTAATTCGATGCAACGCGAAGAACCTTACCTACTCTTGACATCCAGAGAACTTAGCAGAGATGGTTTGGTGCCTTCGGGAACTCTGAGACAGGTGCTGCA
TGGCTGTCGTCAGCTCGTGTTGTGAAATGTTGGGTTAAGTCCCGCAACGAGCGCAACCCTTATCCTTTGTTGCCAGCGGTTCGGCCGGGAACTCAAAGGA
GACTGCCAGTGATAAACTGGAGGAAGGTGGGGATGACGTCAAGTCATCATGGCCCTTACGAGTAGGGCTACACACGTGCTACAATGGCGTATACAAAGAG
AAGCGAACCTGCGAGGGCAAGCGGACCTCATAAAGTACGTCGTAGTCCGGATTGGAGTCTGCAACTCGACTCCATGAAGTCGGAATCGCTAGTAATCGTA
GATCAGAATGCTACGGTGAATACGTTCCCGGGCCTTGTACACACCGCCCGTCACACCATGGGAGTGGGTTGCAAAAGAAGTAGGTAGCTTAACCTTCGGG
AGGGCGCT

>EB31
CTCCCTCCACAAGGGTTGGGCCACCGGCTTCGGGTGTTGCCGACTTTCGTGACGTGACGGGCGGTGTGTACAAGGCCCGGGAACGTATTCACCGCAGCGT
TGCTGATCTGCGATTACTAGCGACTCCGACTTCATGGGGTCGAGTTGCAGACCCCAATCCGAACTGAGACCGGCTTTTTGGGATTCGCTCCCCCTTACGG
GATCGCAGCCCTTTGTACCGGCCATTGTAGCATGCGTGAAGCCCTGGACATAAGGGGCATGATGACTTGACGTCATCCCCACCTTCCTCCGAGTTGACCC
CGGCAGTCTCCTATGAGTCCCCACCATTACGTGCTGGCAACATAGAACGAGGGTTGCGCTCGTTGCGGGACTTAACCCAACATCTCACGACACGAGCTGA
CGACAGCCATGCACCACCTGTACACCCCCAAAAGAAGCCCCCATCTCTGGAGCGGCAGGGTGTATGTCAAACCCAGGTAAGGTTCTTCGCGTTGCATCGA
ATTAATCCGCATGCTCCGCCGCTTGTGCGGGCCCCCGTCAATTCCTTTGAGTTTTAGCCTTGCGGCCGTACTCCCCAGGCGGGGCGCTTAATGCGTTAGC
TGCGGCACGGAATCCGTGGAATGGACCCCACACCTAGCGCCCAACGTTTACGGTGTGGACTACCAGGGTATCTAATCCTGTTCGCTCCCCACACTTTCGC
TCCTCAGCGTCAGGTCATTCCCAGAGAACCGCCTTCGCCACCGGTGTTCCTCCTGATATCTGCGCATTTCACCGCTACACCAGGAATTCCGTTCTCCCCT
GAATACCTCTAGTCTGCCCGTATCGAAAGCAAGCCAGGTGTTAAGCACCTGGTTTTCACTCCCGACGCGACAAACCGCCTACGAGCCCTTTACGCCCAAT
AATTCCGGACAACGCTCGCACCCTACGTATTACCGCGGCTGCTGGCACGTAGTTGGCCGGTGCTTCTTTACCTGTTACCGTCACTTGCGCTTCGTCACAG
GCGAAAGAGGTTTACAACCCGAAGGCCGTCATCCCTCACGCGGCGTTGCTGGATCAGGCTTCCGCCCATTGTCCAATATTCCCCACTGCTGCCTCCCGTA
GGAGTCTGGGCCGTGTCTCAGTCCCAGTGTGGCCGGTCACCCTCTCAGGCCGGCTACCCGTCGAAGCCATGGTGAGCCATTACCTCACCATCAAGCTGAT
AGGCCGCGAGCACATCCCTGGCCGAAAAACTTTCCACCACCATCACATGCGCGAAGTGGTCATATCCGGTATTAATCACCGTTTCCGGTGGCTATCCCAA
AGCCAGGGGCAGATTACTCACGTGTTACTCACCCGTTCGCCGCTCGAGTACCACCGAAGTGGCCTTCCGCTCGACTGCA

>EB32
GCAGTCGAACGATGAAGCCCAGCTTGCTGGGTGGATTAGTGGCGAACGGGTGAGTAACACGTGAGTAACCTGCCCTGGACTCTGGGATAACCCCGAGAAA
TCGGAGCTAATACCGGATAGGACCTTTCCTCGCATGAGGTTTGGTGGAAAGTTTTTCGGTCTGGGATGGACTCGCGGCCTATCAGCTTGTTGGTGAGGTA
ATGGCTCACCAAGGCGTCGACGGGTAGCCGGCCTGAGAGGGTGACCGGCCACACTGGGACTGAGACACGGCCCAGACTCCTACGGGAGGCAGCAGTGGGG
AATATTGCACAATGGGCGCAAGCCTGATGCAGCAACGCCGCGTGCGGGATGACGGCCTTCGGGTTGTAAACCGCTTTTAGTAGGGAAGAAGGGCTTCGGC
TTGACGGTACCTGCAGAAAAAGGACCGGCTAACTACGTGCCAGCAGCCGCGGTAATACGTAGGGTCCGAGCGTTGTCCGGAATTATTGGGCGTAAAGAGC
TCGTAGGCGGTTTGTCGCGTCTGCTGTGAAAACTAGAGGCTCAACCTCTAGCCTGCAGTGGGTACGGGCAGACTTGAGTGGTGTAGGGGAGACTGGAATT
CCTGGTGTAGCGGTGGAATGCGCAGATATCAGGAGGAACACCGATGGCGAAGGCAGGTCTCTGGGCACTTACTGACGCTGAGGAGCGAAAGCGTGGGGAG
CGAACAGGATTAGATACCCTGGTAGTCCACGCCGTAAACGTTGGGCGCTAGATGTGGGGACCTTTCCACGGTTTCCGTGTCGTAGCTAACGCATTAAGCG
CCCCGCCTGGGGAGTACGGCCGCAAGGCTAAAACTCAAAGGAATTGACGGGGGCCCGCACAAGCGGCGGAGCATGCGGATTAATTCGATGCAACGCGAA
GAACCTTACCAAGGCTTGACATATACGAGAACGGGCCAGAAATGGTCAACTCTTTGGACACTCGTATACAGGTGGTGCATGGTTGTCGTCAGCTCGTGTC
GTGAGATGTTGGGTTAAGTCCCGCAACGAGCGCAACCCTCGTCGCATGTTGCCAGCACGTTATGGTGGGGACTCATGTGAGACTGCCGGGGTCAACTCGG
AGGAAGGTGGGGATGACGTCAAATCATCATGCCCCTTATGTCTTGGGCTTCACGCATGCTACAATGGCCGGTACAAAGGGCTGCGATGTCGTAAGGCGGA
GCGAATCCCAAAAAGCCGGTCTCAGTTCGGATTGAGGTCTGCAACTCGACCTCATGAAGTCGGAGTCGCTAGTAATCGCAGATCAGCAACGCTGCGGTGA
ATACGTTCCCGGGCCTTGTACACACCGCCCGTCAAGTCATGAAAGTCGGTAACACCCGAAGCCGGTGGCCTAACCCTTGTGGAGGGAGCCGT

>EB33
TGCAGTCGAACGGCAGCACGGACTTCGGTCTGGTGGCGAGTGGCGAACGGGTGAGTAATGTATCGGAACGTGCCTAGTAGCGGGGGATAACTACGCGAAA
GCGTAGCTAATACCGCATACGCCCTACGGGGGAAAGCAGGGGATCGCAAGACCTTGCACTATTAGAGCGGCCGATATCGGATTAGCTAGTTGGTGGGGTA
ACGGCTCACCAAGGCGACGATCCGTAGCTGGTTTGAGAGGACGACCAGCCACACTGGGACTGAGACACGGCCCAGACTCCTACGGGAGGCAGCAGTGGGG
AATTTTGGACAATGGGGGAAACCCTGATCCAGCCATCCCGCGTGTGCGATGAAGGCCTTCGGGTTGTAAAGCACTTTTGGCAGGAAAGAAACGTCATGGG
TTAATACCCCGTGAAAACTGACGGTACCTGCAGAATAAGCACCGGCTAACTACGTGCCAGCAGCCGCGGTAATACGTAGGGTGCAAGCGTTAATCGGAAT

TACTGGGCGTAAAGCGTGCGCAGGCGGTTCGGAAAGAAAGATGTGAAATCCCAGAGCTTAACTTTGGAACTGCATTTTTAACTACCGGGCTAGAGTGTGT
CAGAGGGAGGTGGAATTCCGCGTGTAGCAGTGAAATGCGTAGATATGCGGAGGAACACCGATGGCGAAGGCAGCCTCCTGGGATAACACTGACGCTCATG
CACGAAAGCGTGGGGAGCAAACAGGATTAGATACCCTGGTAGTCCACGCCCTAAACGATGTCAACTAGCTGTTGGGGCCTTCGGGCCTTAGTAGCGCAGC
TAACGCGTGAAGTTGACCGCCTGGGGAGTACGGTCGCAAGATTAAAACTCAAAGGAATTGACGGGGACCCGCACAAGCGGTGGATGATGTGGGATTAAT
TCGATGCAACGCGAAAAACCTTACCTACCCCTTGACATGTCTGGAATTCCGAAGAGATTCGGAAAGTGCTCGCAAGAGAACCGGAACACAGGTGCTGCAT
GGCTGTCGTCAGCTCGTGTCGTGAGATGTTGGGTTAAGTCCCGCAACGAGCGCAACCCTTGTCATTAGTTGCTACGAAAGGGCACTCTAATGAGACTGCC
GGTGACAAACCGGAGGAAGGTGGGGATGACGTCAAGTCCTCATGGCCCTTATGGGTAGGGCTTCACACGTCATACAATGGTCGGGACAGAGGGTCGCCAA
CCCGCGAGGGGGAGCCAATCCCAGAAACCCGATCGTAGTCCGGATCGCAGTCTGCAACTCGACTGCGTGAAGTCGGAATCGCTAGTAATCGCGGATCAGC
ATGTCGCGGTGAATACGTTCCCGGGTCTTGTACACACCGCCCGTCACACCATGGGAGTGGGTTTACCAGAAGTAGTTAGCCTAACCGTAAGGGGGGCGA
T

>EB34
TGCAAGTCGAACGGACCCTTCGGGGTTAGTGGCGGACGGGTGAGTAACACGTGGGAACGTGCCTTTAGGTTCGGAATAGCTCCTGGAAACGGGTGGTAAT
GCCGAATGTGCCCTTCGGGGGAAAGATTTATCGCCTTTAGAGCGGCCCGCGTCTGATTAGCTAGTTGGTGAGGTAATGGCTCACCAAGGCGACGATCAGT
AGCTGGTCTGAGAGGATGACCAGCCACATTGGGACTGAGACACGGCCCAAACTCCTACGGGAGGCAGCAGTGGGGAATCTTGCGCAATGGGCGAAAGCCT
GACGCAGCCATGCCGCGTGAATGATGAAGGTCTTAGGATTGTAAAATTCTTTCACCGGGGACGATAATGACGGTACCCGGAGAAGAAGCCCCGGCTAACT
TCGTGCCAGCAGCCGCGGTAATACGAAGGGGGCTAGCGTTGCTCGGAATTACTGGGCGTAAAGGGCGCGTAGGCGGACATTTAAGTCAGGGGTGAAATCC
CAGAGCTCAACTCTGGAACTGCCTTTGATACTGGGTGTCTTGAGTGTGAGAGAGGTATGTGGAACTCCGAGTGTAGAGGTGAAATTCGTAGATATTCGGA
AGAACACCAGTGGCGAAGGCGACATACTGGCTCATTACTGACGCTGAGGCGCGAAAGCGTGGGGAGCAAACAGGATTAGATACCCTGGTAGTCCACGCCG
TAAACGATGATTGCTAGTTGTCGGGCTGCATGCAGTTCGGTGACGCAGCTAACGCATTAAGCAATCCGCCTGGGGAGTACGGTCGCAAGATTAAAACTCA
AAGGAATTGACGGGGGCCCGCACAAGCGGTGGAGCATGTGGTTTAATTCGAAGCAACGCGCAGAACCTTACCACCTTTTGACATGCCTGGACCGCCAGAG
AGATCTGGCTTTCTCTTCGGAGACTAGGACACAGGTGCTGCATGGCTGTCGTCAGCTCGTGTCGTGAGATGTTGGGTTAAGTCCCGCAACGAGCGCAACC
CTCGCCATTAGTTGCCATCATTTAGTTGGGAACTCTAATGGGACTGCCGGTGCTAAGCCGGAGGAAGGTGGGGATGACGTCAAGTCCTCATGGCCCTTAC
AGGGTGGGCTACACACGTGCTACAATGGCGACTACAGAGGGTTAATCCTTAAAAGTCGTCTCAGTTCGGATTGTCCTCTGCAACTCGAGGGCATGAAGTT
GGAATCGCTAGTAATCGCGGATCAGCATGCCGCGGTGAATACGTTCCCGGGCCTTGTACACACCGCCCGTCACACCATGGGAGTTGGTTCTACCCGAAGG
CGATGCGCTAACCAGCAATGGAGGCAGTCG

>EB35
GCTCAGGACGAACGCTGGCGGCGTGCCTAATACATGCAAGTCGAGCGGACCTCTTCGGAGATCAGCGGCGGACGGGTGAGTAACACGTGGGCAACCTGCC
TGTAAGACTGGGATAACTCCGGGAAACCGGAGCTAATACCGGATACTATGTCAAACCGCATGGTTTGACATTCAAAGACGGTTTCGGCTGTCACTTACAG
ATGGGCCCGCGGCGCATTAGCTAGTTGGTGAGGTAATGGCTCACCAAGGCGACGATGCGTAGCCGACCTGAGAGGGTGATCGGCCACACTGGGACTGAGA
CACGGCCCAGACTCCTACGGGAGGCAGCAGTAGGGAATCTTCCGCAATGGACGAAAGTCTGACGGAGCAACGCCGCGTGAGTGATGAAGGTTTTCGGATC
GTAAAACTCTGTTGTCAGGGAAGAACAAGTGCCGGAGTAACTGCCGGCACCTTGACGGTACCTGACCAGAAAGCCACGGCTAACTACGTGCCAGCAGCCG
CGGTAATACGTAGGTGGCAAGCGTTGTCCGGAATTATTGGGCGTAAAGCGCGCGCAGGCGGTTTCTTAAGTCTGATGTGAAAGCCCCCGGCTCAACCGGG
GAGGGTCATTGGAAACTGGGAAACTTGAGTGCAGAAGAGGAGAGTGGAATTCCACGTGTAGCGGTGAAATGCGTAGAGATGTGGAGGAACACCAGTGGCG
AAGGCGACTCTCTGGTCTGTAACTGACGCTGAGGCGCGAAAGCGTGGGGAGCGAACAGGATTAGATACCCTGGTAGTCCACGCCGTAAACGATGAGTGCT
AAGTGTTAGAGGGTTTCCGCCCTTTAGTGCTGCAGCTAACGCATTAAGCACTCCGCCTGGGGAGTACGGTCGCAAGACTGAAACTCAAAGGAATTGACGG
GGGCCCGCACAAGCGGTGGAGCATGTGGTTTAATTCGAAGCAACGCGAAGAACCTTACCAGGTCTTGACATCCTTTGCCACTTCTAGAGATAGAAGGTTC
CCCTTCGGGGGACAAAGTGACAGGTGGTGCATGGTTGTCGTCAGCTCGTGTCGTGAGATGTTGGGTTAAGTCCCGCAACGAGCGCAACCCTTGATCTTAG
TTGCCAGCATTCAGTTGGGCACTCTAAGGTGACTGCCGGTGACAAACCGGAGGAAGGTGGGGATGACGTCAAATCATCATGCCCCTTATGACCTGGGCT
ACACACGTGCTACAATGGATGGTACAAAGGGCTGCGAGACCGCGAGGTTTAGCCAATCCCATAAAACCATTCTCAGTTCGGATTGCAGGCTGCAACTCGC
CTGCATGAAGCTGGAATCGCTAGTAATCGCGGATCAGCATGCCGCGGTGAATACGTTCCCGGGCCTTGTACACACCGCCCGTCACACCACGAGAGTTTGC
AACACCCGAAGTCGGTGGGGTAACCGCAAGGAGCCAGCCGCCTAAAGGTGGGGTAAATGAATTGGGG

>EB36
CCCCCCACACAAGGTGGTTAGGCCATCGGCTTCGGGTGTTACCAACTTTCGTGACTTGACGGGCGGTGTGTACAAGGCCCGGGAACGTATTCACCGCAGC
GTTGCTGATCTGCGATTACTAGCGACTCCGACTTCATGGGGTCGAGTTGCAGACCCCAATCCGAACTGAGACCGGCTTTTAGGGATTAGCTCCACCTCAC
AGTATCGCAACCCATTGTACCGGCCATTGTAGCATGCGTGAAGCCCAAGACATAAGGGGCATGATGATTTGACGTCATCCCCACCTTCCTCCGAGTTGAC
CCCGGCAGTCTCCCATGAGTCCCCACCACTACGTGCTGGCAACATGGAACGAGGGTTGCGCTCGTTGCGGGACTTAACCCAACATCTCACGACACGAGCT
GACGACAACCATGCACCACCTGTGAACCAGCCCCGAAGGGAAACCCCATCTCTGGAGCGGTCTGGCACATGTCAAGCCTTGGTAAGGTTCTTCGCGTTGC
ATCGAATTAATCCGCATGCTCCGCCGCTTGTGCGGGCCCCCGTCAATTCCTTTGAGTTTTAGCCTTGCGGCCGTACTCCCCAGGCGGGGCACTTAATGCG
TTAGCTACGGCGCGGAAAACGTGGAATGTCCCCCACACCTAGTGCCCAACGTTTACGGCATGGACTACCAGGGTATCTAATCCTGTTCGCTCCCCATGCT
TTCGCTCCTCAGCGTCAGTAAATGCCCAGAGACCTGCCTTCGCCATCGGTGTTCCTCCTGATATCTGCGCATTTCACCGCTACACCAGGAATTCCAGTCT
CCCCTACATCACTCTAGTCTGCCCGTACCCACCGCAGATCCGAGGTTGAGCCTCGGACTTTCACGGCAGACGCGACAAACCGCCTACGAGCTCTTTACGC
CCAATAAATCCGGATAACGCTTGCGCCCTACGTATTACCGCGGCTGCTGGCACGTAGTTAGCCGGCGCTTCTTCTGCAGGTACCGTCACTTTCGCTTCTT
CCCTACTGAAAGAGGTTTACAACCCGAAGGCCGTCATCCCTCACGCGGCGTCGCTGCATCAGGCTTCCGCCCATTGTGCAATATTCCCCACTGCTGCCTC
CCGTAGGAGTCTGGGCCGTGTCTCAGTCCCAGTGTGGCCGGTCACCCTCTCAGGCCGGCTACCCGTCGTCGCCTTGGTGAGCCATTACCTCACCAACAAG
CTGATAGGCCGCGAGTCCATCCCCCACCGATAAATCTTTCAACAACCCACCATGCGGCAGGAAGTCATATCCGGTATTAGACCCAGTTTCCCGGGCTTAT
CCCAAAGTCGGGGGCAGGTTACTCACGTGTTACTCACCCGTTCGCCACTAATCCACCCAGCAAGCTGGGCTTCATCGTTCGA

>EB37
TGCAGTCGAACGATGATCCCAGCTTGCTGGGGGATTAGTGGCGAACGGGTGAGTAACACGTGAGTAACCTGCCCTTAACTCTGGGATAAGCCTGGGAAAC
TGGGTCTAATACCGGATATGACTCCTCATCGCATGGTGGGGGGTGGAAAGCTTTATTGTGGGTTTTGGATGGACTCGCGGCCTATCAGCTTGTTGGTGAG
GTAATGGCTCACCAAGGCGACGACGGGTAGCCGGCCTGAGAGGGTGACCGGCCACACTGGGACTGAGACACGGCCCAGACTCCTACGGGAGGCAGCAGTG
GGGAATATTGCACAATGGGCGAAAGCCTGATGCAGCGACGCCGCGTGAGGGATGACGGCCTTCGGGTTGTAAACCTCTTTCAGTAGGGAAGAAGCGAAAG
TGACGGTACCTGCAGAAGAAGCGCCGGCTAACTACGTGCCAGCAGCCGCGGTAATACGTAGGGCGCAAGCGTTATCCGGAATTATTGGGCGTAAAGAGCT
CGTAGGCGGTTTGTCGCGTCTGCCGTGAAAGTCCGGGGCTCAACTCCGGATCTGCGGTGGGTACGGGCAGACTAGAGTGATGTAGGGGAGACTGGAATTC
CTGGTGTAGCGGTGAAATGCGCAGATATCAGGAGGAACACCGATGGCGAAGGCAGGTCTCTGGGCATTAACTGACGCTGAGGAGCGAAAGCATGGGGAGC
GAACAGGATTAGATACCCTGGTAGTCCATGCCGTAAACGTTGGGCACTAGGTGTGGGGGACATTCCACGTTTTCCGCGCCGTAGCTAACGCATTAAGTGC
CCCGCCTGGGGAGTACGGCCGCAAGGCTAAAACTCAAAGGAATTGACGGGGGCCCGCACAAGCGGCGGAGCATGCGGATTAATTCGATGCAACGCGAAGA
ACCTTACCAAGGCTTGACATGAACCGGAAACACCTGGAAACAGGTGCCCCGCTTGCGGTCGGTTTACAGGTGGTGCATGGTTGTCGTCAGCTCGTGTCGT
GAGATGTTGGGTTAAGTCCCGCAACGAGCGCAACCCTCGTTCTATGTTGCCAGCGCGTTATGGCGGGGACTCATAGGAGACTGCCGGGGTCAACTCGGAG
GAAGGTGGGGACGACGTCAAATCATCATGCCCCTTATGTCTTGGGCTTCACGCATGCTACAATGGCCGGTACAAAGGGTTGCGATACTGTGAGGTGGAGC
TAATCCCAAAAAGCCGGTCTCAGTTCGGATTGGGGTCTGCAACTCGACCCCATGAAGTCGGAGTCGCTAGTAATCGCAGATCAGCAACGCTGCGGTGAAT
ACGTTCCCGGGCCTTGTACACACCGCCCGTCAAGTCACGAAAGTTGGTAACACCCGAAGCCGGTGGCCTAACCCCTTG

>EB38

AAGTCGAACGATGATGGGAGCTTGCTCCTGGATTAGTGGCGAACGGGTGAGTAACACGTGAGTAACCTGCCCTTAACTCTGGGATAAGCCTGGGAAACTG
GGTCTAATACCGGATATGACTCCTCATCGCATGGTGGGGGGTGGAAAGCTTTATTGTGGTTTTGGATGGACTCGCGGCCTATCAGCTTGTTGGTGAGGTA
ATGGCTTACCAAGGCGACGACGGGTAGCCGGCCTGAGAGGGTGACCGGCCACACTGGGACTGAGACACGGCCCAGACTCCTACGGGAGGCAGCAGTGGGG
AATATTGCACAATGGGCGCAAGCCTGATGCAGCGACGCCGCGTGAGGGATGACGGCCTTCGGGTTGTAAACCTCTTTCAGTAGGGAAGAAGCGAAAGTGA
CGGTACCTGCAGAAGAAGCGCCGGCTAACTACGTGCCAGCAGCCGCGGTAATACGTAGGGCGCAAGCGTTATCCGGAATTATTGGGCGTAAAGAGCTCGT
AGGCGGTTTGTCGCGTCTGCCGTGAAAGTCCGGGGCTCAACTCCGGATCTGCGGTGGGTACGGGCAGACTAGAGTGATGTAGGGGAGACTGGAATTCCTG
GTGTAGCGGTGAAATGCGCAGATATCAGGAGGAACACCGATGGCGAAGGCAGGTCTCTGGGCATTAACTGACGCTGAGGAGCGAAAGCATGGGGAGCGAA
CAGGATTAGATACCCTGGTAGTCCATGCCGTAAACGTTGGGCACTAGGTGTGGGGGACATTCCACGTTTTCCGCGCCGTAGCTAACGCATTAAGTGCCCC
GCCTGGGGAGTACGGCCGCAAGGCTAAAACTCAAAGGAATTGACGGGGGCCCGCACAAGCGGCGGAGCATGCGGATTAATTCGATGCAACGCGAAGAACC
TTACCAAGGCTTGACATGAACCGGTAATACCTGGAGACAGGTGCCCCGCTTGCGGTCGGTTTACAGGTGGTGCATGGTTGTCGTCAGCTCGTGTCGTGAG
ATGTTGGGTTAAGTCCCGCAACGAGCGCAACCCTCGTTCTATGTTGCCAGCACGTGATGGTGGGGACTCATAGGAGACTGCCGGGGTCAACTCGGAGGAA
GGTGGGGACGACGTCAAATCATCATGCCCCTTATGTCTTGGGCTTCACGCATGCTACAATGGCCGGTACAAAGGGTTGCGATACTGTGAGGTGGAGCTAA
TCCCAAAAAGCCGGTCTCAGTTCGGATTGGGGTCTGCAACTCGACCCCATGAAGTCGGAGTCGCTAGTAATCGCAGATCAGCAACGCTGCGGTGAATACG
TTCCCGGGCCTTGTACACACCGCCCGTCAAGTCACGAAAGTTGGTAACACCCGAAGCCGGTGGCCTAACCCCTTGTGGGAGGGAG

>EB39
GCAGTCGAACGATGATCTCCAGCTTGCTGGGGGGATTAGTGGCGAACGGGTGAGTAACACGTGAGTAACCTGCCCTTAACTCTGGGATAAGCCTGGGAAA
CTGGGTCTAATACCGGATATGACTCCTCATCGCATGGTGGGGGGTGGAAAGCTTTTGTGGTTTTGGATGGACTCGCGGCCTATCAGCTTGTTGGTGAGGT
AATGGCTCACCAAGGCGACGACGGGTAGCCGGCCTGAGAGGGTGACCGGCCACACTGGGACTGAGACACGGCCCAGACTCCTACGGGAGGCAGCAGTGGG
GAATATTGCACAATGGGCGCAAGCCTGATGCAGCGACGCCGCGTGAGGGATGACGGCCTTCGGGTTGTAAACCTCTTTCAGTAGGGAAGAAGCGAAAGTG
ACGGTACCTGCAGAAGAAGCGCCGGCTAACTACGTGCCAGCAGCCGCGGTAATACGTAGGGCGCAAGCGTTATCCGGAATTATTGGGCGTAAAGAGCTCG
TAGGCGGTTTGTCGCGTCTGCCGTGAAAGTCCGGGGCTCAACTCCGGATCTGCGGTGGGTACGGGCAGACTAGAGTGATGTAGGGGAGACTGGAATTCCT
GGTGTAGCGGTGAAATGCGCAGATATCAGGAGGAACACCGATGGCGAAGGCAGGTCTCTGGGCATTAACTGACGCTGAGGAGCGAAAGCATGGGGAGCGA
ACAGGATTAGATACCCTGGTAGTCCATGCCGTAAACGTTGGGCACTAGGTGTGGGGGACATTCCACGTTTTCCGCGCCGTAGCTAACGCATTAAGTGCCC
CGCCTGGGGAGTACGGCCGCAAGGCTAAAACTCAAAGGAATTGACGGGGGCCCGCACAAGCGGCGGAGCATGCGGATTAATTCGATGCAACGCGAAGAAC
CTTACCAAGGCTTGACATGGACCGGACCGCCGCAGAAATGTGGTTTCCCCTTTGGGGCCGGTTCACAGGTGGTGCATGGTTGTCGTCAGCTCGTGTCGTG
AGATGTTGGGTTAAGTCCCGCAACGAGCGCAACCCTCGTTCCATGTTGCCAGCGCGTAATGGCGGGGACTCATGGGAGACTGCCGGGGTCAACTCGGAGG
AAGGTGGGGACGACGTCAAATCATCATGCCCCTTATGTCTTGGGCTTCACGCATGCTACAATGGCCGGTACAAAGGGTTGCGATACTGTGAGGTGGAGCT
AATCCCAAAAAGCCGGTCTCAGTTCGGATTGGGGTCTGCAACTCGACCCCATGAAGTCGGAGTCGCTAGTAATCGCAGATCAGCAACGCTGCGGTGAATA
CGTTCCCGGGCCTTGTACACACCGCCCGTCAAGTCACGAAAGTTGGTAACACCCGAAGCCGGTGGCCTAACCCCTTGTGGGAGGGAG

>EB40
GTGGCGAACGGGTGAGTAACACGTGAGTAACCTGCCCTTAACTCTGGGATAAGCCTGGGAAACTGGGTCTAATACCGGATATGACTCCTCATCGCATGGT
GGGGGGTGGAAAGCTTTTTGTGGTTTTGGATGGACTCGCGGCCTATCAGCTTGTTGGTGAGGTAATGGCTTACCAAGGSGACGACGGGTAGCCGGCCTGA
RAGGGTGACCGGCCACACTGGGACTGAGACACGGCCCAGACTCCTACGGGAGGCAGCAGTGGGGAATATTGCACAATGGGCGCAAGCCTGATGCAGSGAC
GCCGCGTGACGGATGACGGCCTTCGGGTTGTAAACCTCTTTCAGTAGGGAAGAAGCGAAAGTGACGGTACCTGCAGAAGAAGCGCCGGCTAACTACGTGC
CAGCAGCCGCGGTAATACGTAGGGCGCAAGCGTTATCCGGAATTATTGGGCGTAAAGAGCTCGTAGGCGGTTTGTCGCGTCTGCCGTGAAAGTCCGGGGC
TCAACTCCGGATCTGCGGTGGGTACGGGCAGACTAGAGTGATGTAGGGGAGACTGGAATTCCTGGTGTAGCGGTGAAATGCGCAGATATCAGGAGGAACA
CCGATGGCGAAGGCAGGTCTCTGGGCATTAACTGACGCTGAGGAGCGAAAGCATGGGGAGCGAACAGGATTAGATACCCTGGTAGTCCATGCCGTAAACG
TTGGGCACTAGGTGTGGGGGACATTCCACGTTTTCCGCGCCGTAGCTAACGCATTAAGTGCCCCGCCTGGGGAGTACGGCCGCAAGGCTAAAACTCAAAG
GAATTGACGGGGGCCCGCACAAGCGGCGGAGCATGCGGATTAATTCGATGCAACGCGAAGAACCTTACCAAGGCTTGACATGAACCGGTAACGCCTGGAG
ACAGGTGCCCCGCTTGCGGTCGGTTTACAGGTGGTGCATGGTTGTCGTCAGCTCGTGTCGTGAGATGTTGGGTTAAGTCCCGCAACGAGCGCAACCCTCG
TTCTATGTTGCCAGCACGTGATGGTGGGGACTCATAGGAGACTGCCGGGGTCAACTCGGAGGAAGGTGGGGACGACGTCAAATCATCATGCCCCTTATGT
CTTGGGCTTCACGCATGCTACAATGGCCGGTACAAAGGGTTGCGATACTGTGAGGTGGAGCTAATCCCAAAAAGCCGGTCTCAGTTCGGATTGGGGTCTG
CAACTCGACCCCATGAAGTCGGAGTCGCTAGTAATCGCAGATCAGCAACGCTGCGGTGAATACGTTCCCGGGCTTTGTACACACCGCCCGTCAAGTCACG
AAACTTGGTAACACCTGAAGCCGGTGGCGTATCACTAAGT

>EB41
TCAGGATGAACGCTGGCGGCGTGCTTAACACATGCAAGTCGAACGATGATCCCAGCTTGCTGGGGGATTAGTGGCGAACGGGTGAGTAACACGTGAGTAA
CCTGCCCTTGACTCTGGGATAAGCCTGGGAAACTGGGTCTAATACCGGATATGACTCCTCATCGCATGGTGGGGGGTGGAAAGCTTTTGTGGTTTTGGAT
GGACTCGCGGCCTATCAGCTTGTTGGTGGGGTAATGGCCTACCAAGGCGACGACGGGTAGCCGGCCTGAGAGGGTGACCGGCCACACTGGGACTGAGACA
CGGCCCAGACTCCTACGGGAGGCAGCAGTGGGGAATATTGCACAATGGGCGCAAGCCTGATGCAGCGACGCCGCGTGAGGGATGACGGCCTTCGGGTTGT
AAACCTCTTTCAGTAGGGAAGAAGCGTAAGTGACGGTACCTGCAGAAGAAGCGCCGGCTAACTACGTGCCAGCAGCCGCGGTAATACGTAGGGCGCAAGC
GTTATCCGGAATTATTGGGCGTAAAGAGCTCGTAGGCGGTTTGTCGCGTCTGCTGTGAAAGACCGGGGCTCAACTCCGGTTCTGCAGTGGGTACGGGCAG
ACTAGAGTGCAGTAGGGGAGACTGGAATTCCTGGTGTAGCGGTGAAATGCGCAGATATCAGGAGGAACACCGATGGCGAAGGCAGGTCTCTGGGCTGTAA
CTGACGCTGAGGAGCGAAAGCATGGGGAGCGAACAGGATTAGATACCCTGGTAGTCCATGCCGTAAACGTTGGGCACTAGGTGTGGGGGACATTCCACGT
TTTCCGCGCCGTAGCTAACGCATTAAGTGCCCCGCCTGGGGAGTACGGCCGCAAGGCTAAAACTCAAAGGAATTGACGGGGGCCCGCACAAGCGGCGGAG
CATGCGGATTAATTCGATGCAACGCGAAGAACCTTACCAAGGCTTGACATGAACCGGAAAGACCTGGAAACAGGTGCCCCGCTTGCGGTCGGTTTACAGG
TGGTGCATGGTTGTCGTCAGCTCGTGTCGTGAGATGTTGGGTTAAGTCCCGCAACGAGCGCAACCCTCGTTCTATGTTGCCAGCGGTTCGGCCGGGGACT
CATAGGAGACTGCCGGGGTCAACTCGGAGGAAGGTGGGGACGACGTCAAATCATCATGCCCCTTATGTCTTGGGCTTCACGCATGCTACAATGGCCGGTA
CAAAGGGTTGCGATACTGTGAGGTGGAGCTAATCCCAAAAAGCCGGTCTCAGTTCGGATTGGGGTCTGCAACTCGACCCCATGAAGTCGGAGTCGCTAGT
AATCGCAGATCAGCAACGCTGCGGTGAATACGTTCCCGGGCCTTGTACACACCGCCCGTCAAGTCACGAAAGTTGGTAACACCCGAAGCCGGTGGCCTAA
CCCTTGTGGGGGGGAGCCGTCCAAAGGGGGAACCGGCGATTGGGACTAATCGAAAAA

>EB42
GCAGTCGAACGATGATCCGGTGCTTGCACCGGGGATTAGTGGCGAACGGGTGAGTAACACGTGAGTAACCTGCCCTTGACTCTGGGATAAGCCTGGGAAA
CTGGGTCTAATACCGGATATGACCTTCCATCGCATGGTGGTTGGTGGAAAGCTTTTGTGGTTTTGGATGGACTCGCGGCCTATCAGCTTGTTGGTGAGGT
AATGGCTCACCAAGGCGACGACGGGTAGCCGGCCTGAGAGGGTGACCGGCCACACTGGGACTGAGACACGGCCCAGACTCCTACGGGAGGCAGCAGTGGG
GAATATTGCACAATGGGCGCAAGCCTGATGCAGCGACGCCGCGTGAGGGATGACGGCCTTCGGGTTGTAAACCTCTTTCAGTAGGGAAGAAGCGAAAGTG
ACGGTACCTGCAGAAGAAGCGCCGGCTAACTACGTGCCAGCAGCCGCGGTAATACGTAGGGCGCAAGCGTTATCCGGAATTATTGGGCGTAAAGAGCTCG
TAGGCGGTTTGTCGCGTCTGCCGTGAAAGTCCGGGGCTCAACTCCGGATCTGCGGTGGGTACGGGCAGACTAGAGTGATGTAGGGGAGACTGGAATTCCT
GGTGTAGCGGTGAAATGCGCAGATATCAGGAGGAACACCGATGGCGAAGGCAGGTCTCTGGGCATTAACTGACGCTGAGGAGCGAAAGCATGGGGAGCGA
ACAGGATTAGATACCCTGGTAGTCCATGCCGTAAACGTTGGGCACTAGGTGTGGGGGACATTCCACGTTTTCCGCGCCGTAGCTAACGCATTAAGTGCCC
CGCCTGGGGAGTACGGCCGCAAGGCTAAAACTCAAAGGAATTGACGGGGGCCCGCACAAGCGGCGGAGCATGCGGATTAATTCGATGCAACGCGAAGAAC
CTTACCAAGGCTTGACATGAACCGGAAAGACCTGGAAAACAGGTGCCCCGCTTGCGGTCGGTTTACAGGTGGTGCATGGTTGTCGTCAGCTCGTGTCGTG
AGATGTTGGGTTAAGTCCCGCAACGAGCGCAACCCTCGTTCTATGTTGCCAGCACGTGATGGTGGGGACTCATAGGAGACTGCCGGGGTCAACTCGGAGG
AAGGTGGGGACGACGTCAAATCATCATGCCCCTTATGTCTTGGGCTTCACGCATGCTACAATGGCCGGTACAAAGGGTTGCGATACTGTGAGGTGGAGCT

AATCCCAAAAAGCCGGTCTCAGTTCGGATTGGGGTCTGCAACTCGACCCCATGAAGTCGGAGTCGCTAGTAATCGCAGATCAGCAACGCTGCGGTGAATA
CGTTCCCGGGCCTTGTACACACCGCCCGTCAAGTCACGAAAGTTGGTAACACCCGAAGCCGGTGGCCTAACCCCTTGTGGGAGGGA

>EB43
TGCAGTCGAACGATGAACCTCACTTGTGGGGGGATTAGTGGCGAACGGGTGAGTAACACGTGAGTAACCTGCCCTTGACTCTGGGATAAGCCTGGGAAAC
TGGGTCTAATACCGGATATGACTCCTCATCGCATGGTGGGGGGTGGAAAGCTTTTTGTGGTTTTGGATGGACTCGCGGCCTATCAGCTTGTTGGTGAGGT
AATGGCTCACCAAGGCGACGACGGGTAGCCGGCCTGAGAGGGTGACCGGCCACACTGGGACTGAGACACGGCCCAGACTCCTACGGGAGGCAGCAGTGGG
GAATATTGCACAATGGGCGAAAGCCTGATGCAGCGACGCCGCGTGAGGGATGACGGCCTTCGGGTTGTAAACCTCTTTCAGTAGGGAAGAAGCGAAAGTG
ACGGTACCTGCAGAAGAAGCGCCGGCTAACTACGTGCCCAGCAGCCGCGGTAATACGTAGGGCGCAAGCGTTATCCGGAATTATTGGGCGTAAAGAGCTC
GTAGGCGGTTTGTCGCGTCTGCCGTGAAAGTCCGGGGCTCAACTCCGGATCTGCGGTGGGTACGGGCAGACTAGAGTGATGTAGGGGAGACTGGAATTCC
TGGTGTAGCGGTGAAATGCGCAGATATCAGGAGGAACACCGATGGCGAAGGCAGGTCTCTGGGCATTAACTGACGCTGAGGAGCGAAAGCATGGGGAGCG
AACAGGATTAGATACCCTGGTAGTCCATGCCGTAAACGTTGGGCACTAGGTGTGGGGGACATTCCACGTTTTCCGCGCCGTAGCTAACGCATTAAGTGCC
CCGCCTGGGGGAGTACGGCCGCAAGGCTAAAAACTCAAAGGAATTGACGGGGGCCCGCACAAGCGGCGGAGCATGCGGATTAATTCGATGCAACGCGAAG
AACCTTACCAAGGCTTGACATGGACTGGAAATACCTGGAAACAGGTGCCCCGCTTGCGGTCGGTTCACAGGTGGTGCATGGTTGTCGTCAGCTCGTGTCG
TGAGATGTTGGGTTAAGTCCCGCAACGAGCGCAACCCTCGTTCTATGTTGCCAGCGCGTTATGGCGGGGACTCATAGGAGACTGCCGGGGTCAACTCGGA
GGAAGGTGGGGACGACGTCAAATCATCATGCCCCTTATGTCTTGGGCTTCACGCATGCTACAATGGCCGGTACAAAGGGTTGCGATACTGTGAGGTGGAG
CTAATCCCAAAAAGCCGGTCTCAGTTCGGATTGGGGTCTGCAACTCGACCCCATGAAGTCGGAGTCGCTAGTAATCGCAGATCAGCAACGCTGCGGTGAA
TACGTTCCCGGGCCTTGTACACACCGCCCGTCAAGTCACGAAAGTTGGTAACACCCGAAGCCGGTGGCCTAACCCCTTGT

>EB44
GTCGAGCGAATGGATTAAGAGCTTGCTCTTATGAAGTTAGCGGCGGACGGGTGAGTAACACGTGGGTAACCTGCCCATAAGACTGGGATAACTCCGGGAA
ACCGGGGCTAATACCGGATAACATTTTGCACCGCATGGTGCGAAATTCAAAGGCGGCTTCGGCTGTCACTTATGGATGGACCCGCGTCGCATTAGCTAGT
TGGTGAGGTAACGGCTCACCAAGGCAACGATGCGTAGCCGACCTGAGAGGGTGATCGGCCACACTGGGACTGAGACACGGCCCAGACTCCTACGGGAGGC
AGCAGTAGGGAATCTTCCGCAATGGACGAAAGTCTGACGGAGCAACGCCGCGTGAGTGATGAAGGCTTTCGGGTCGTAAAACTCTGTTGTTAGGGAAGAA
CAAGTGCTAGTTGAATAAGCTGGCACCTTGACGGTACCTAACCAGAAAGCCACGGCTAACTACGTGCCAGCAGCCGCGGTAATACGTAGGTGGCAAGCGT
TATCCGGAATTATTGGGCGTAAAGCGCGCGCAGGTGGTTTCTTAAGTCTGATGTGAAAGCCCACGGCTCAACCGTGGAGGGTCATTGGAAACTGGGAGAC
TTGAGTGCAGAAGAGGAAAGTGGAATTCCATGTGTAGCGGTGAAATGCGTAGAGATATGGAGGAACACCAGTGGCGAAGGCGACTTTCTGGTCTGTAACT
GACACTGAGGCGCGAAAGCGTGGGGAGCAAACAGGATTAGATACCCTGGTAGTCCACGCCGTAAACGATGAGTGCTAAGTGTTAGAGGGTTTCCGCCCTT
TAGTGCTGAAGTTAACGCATTAAGCACTCCGCCTGGGGAGTACGGCCGCAAGGCTGAAACTCAAAGGAATTGACGGGGGCCCGCACAAGCGGTGGAGCA
TGTGGTTTAATTCGAAGCAACGCGAAGAACCTTACCAGGTCTTGACATCCTCTGACAACCCTAGAGATAGGGCTTCCCCTTCGGGGGCAGAGTGACAGGT
GGTGCATGGTTGTCGTCAGCTCGTGTCGTGAGATGTTGGGTTAAGTCCCGCAACGAGCGCAACCCTTGATCTTAGTTGCCATCATTAAGTTGGGCACTCT
AAGGTGACTGCCGGTGACAAACCGGAGGAAGGTGGGGATGACGTCAAATCATCATGCCCCTTATGACCTGGGCTACACACGTGCTACAATGGACGGTACA
AAGAGCTGCAAGACCGCGAGGTGGAGCTAATCTCATAAAACCGTTCTCAGTTCGGATTGTAGGCTGCAACTCGCCTACATGAAGCTGGAATCGCTAGTAA
TCGCGGATCAGCATGCCGCGGTGAATACGTTCCCGGGCCTTGTACACACCGCCCGTCACACCACGAGAGTTTGTAACACCCGAAGTCGGTGGGGTAACCT
TTTTGGAGCCAGCCGC

>EB45
TGCAGTCGAACGATGAACCTCACTTGTGGGGGGATTAGTGGCGAACGGGTGAGTAACACGTGAGTAACCTGCCCTTGACTCTGGGATAAGCCTGGGAAAC
TGGGTCTAATACCGGATATGACTCCTCATCGCATGGTGGGGGGTGGAAAGCTTTTTGTGGTTTTGGATGGACTCGCGGCCTATCAGCTTGTTGGTGAGGT
AATGGCTCACCAAGGCGACGACGGGTAGCCGGCCTGAGAGGGTGACCGGCCACACTGGGACTGAGACACGGCCCAGACTCCTACGGGAGGCAGCAGTGGG
GAATATTGCACAATGGGCGAAAGCCTGATGCAGCGACGCCGCGTGAGGGATGACGGCCTTCGGGTTGTAAACCTCTTTCAGTAGGGAAGAAGCGAAAGTG
ACGGTACCTGCAGAAGAAGCGCCGGCTAACTACGTGCCAGCAGCCGCGGTAATACGTAGGGCGCAAGCGTTATCCGGAATTATTGGGCGTAAAGAGCTCG
TAGGCGGTTTGTCGCGTCTGCCGTGAAAGTCCGGGGCTCAACTCCGGATCTGCGGTGGGTACGGGCAGACTAGAGTGATGTAGGGGAGACTGGAATTCCT
GGTGTAGCGGTGAAATGCGCAGATATCAGGAGGAACACCGATGGCGAAGGCAGGTCTCTGGGCATTAACTGACGCTGAGGAGCGAAAGCATGGGGAGCGA
ACAGGATTAGATACCCTGGTAGTCCATGCCGTAAACGTTGGGCACTAGGTGTGGGGGACATTCCACGTTTTCCGCGCCGTAGCTAACGCATTAAGTGCCC
CGCCTGGGGAGTACGGCCGCAAGGCTAAAACTCAAAGGAATTGACGGGGGCCCGCACAAGCGGCGGAGCATGCGGATTAATTCGATGCAACGCGAAGAAC
CTTACCAAGGCTTGACATGGACTGGAAATACCTGGAAACAGGTGCCCCGCTTGCGGTCGGTTCACAGGTGGTGCATGGTTGTCGTCAGCTCGTGTCGTGA
GATGTTGGGTTAAGTCCCGCAACGAGCGCAACCCTCGTTCTATGTTGCCAGCGCGTTATGGCGGGGACTCATAGGAGACTGCCGGGGTCAACTCGGAGGA
AGGTGGGGACGACGTCAAATCATCATGCCCCTTATGTCTTGGGCTTCACGCATGCTACAATGGCCGGTACAAAGGGTTGCGATACTGTGAGGTGGAGCTA
ATCCCAAAAAGCCGGTCTCAGTTCGGATTGGGGTCTGCAACTCGACCCCATGAAGTCGGAGTCGCTAGTAATCGCAGATCAGCAACGCTGCGGTGAATAC
GTTCCCGGGCCTTGTACACACCGCCCGTCAAGTCACGAAAGTTGGTAACACCTGAAGCCGGTGGCCTAACCCCTGT

>EB46
TTAGGCCACCGGCTTCGGGTGTTACCAACTTTCGTGACTTGACGGGCGGTGTGTACAAGGCCCGGGAACGTATTCACCGCAGCGTTGCTGATCTGCGATT
ACTAGCGACTCCGACTTCATGGGGTCGAGTTGCAGACCCCAATCCGAACTGAGACCGGCTTTTTGGGATTAGCTCCACCTCACAGTATCGCAACCCTTTG
TACCGGCCATTGTAGCATGCGTGAAGCCCAAGACATAAGGGGCATGATGATTTGACGTCGTCCCCACCTTCCTCCGAGTTGACCCCGGCAGTCTCCTATG
AGTCCCCACCATCACGTGCTGGCAACATAGAACGAGGGTTGCGCTCGTTGCGGGACTTAACCCAACATCTCACGACACGAGCTGACGACAACCATGCACC
ACCTGTAAACCGACCGCAAGCGGGGCACCTGTCTCCAGGTATTACCGGTTCATGTCAAGCCTTGGTAAGGTTCTTCGCGTTGCATCGAATTAATCCGCAT
GCTCCGCCGCTTGTGCGGGCCCCCGTCAATTCCTTTGAGTTTTAGCCTTGCGGCCGTACTCCCCAGGCGGGGCACTTAATGCGTTAGCTACGGCGCGGAA
AACGTGGAATGTCCCCCACACCTAGTGCCCAACGTTTACGGCATGGACTACCAGGGTATCTAATCCTGTTCGCTCCCCATGCTTTCGCTCCTCAGCGTCA
GTTAATGCCCAGAGACCTGCCTTCGCCATCGGCGTTCCTCCTGATATCTGCGCATTTCACCGCTACACCAGGAATTCCAGTCTCCCCTACATCACTCTAG
TCTGCCCGTACCCACCGCAGATCCGGAGTTGAGCCCCGGACTTTCACGGAAGACGCGACAAACCGCCTACGAGCTCTTTACGCCCAATAATTCCGGATAA
CGCTTGCGCCCTACGTATTACCGCGGATGCTGGTACGTAATTAGCCGGTGCTTCTTCTGCAGGAACCGTCACTTTCCGCTTCTTCCCTACTGAAAGAGGT
TTACAACCCGAATGGCCGCCATCC

>EB47
GCAAGTCGAACGATGATCCGGTGCTTGCACCGGGGATTAGTGGCGAACGGGTGAGTAACACGTGAGTAACCTGCCCTTGACTCTGGGATAAGCCTGGGAA
ACCGGGTCTAATACCGGATACGACCTTTCACCGCATGGTGGTTGGTGGAAAGATTTTTTGGTTTTGGATGGACTCGCGGCCTATCAGCTTGTTGGTGAGG
TAATGGCTCACCAAGGCGACGACGGGTAGCCGGCCTGAGAGGGTGACCGGCCACACTGGGACTGAGACACGGCCCAGACTCCTACGGGAGGCAGCAGTGG
GGAATATTGCACAATGGGCGCAAGCCTGATGCAGCGACGCCGCGTGAGGGATGACGGCCTTCGGGTTGTAAACCTCTTTCAGCAGGGAAGAAGCGAAAGT
GACGGTACCTGCAGAAGAAGCGCCGGCTAACTACGTGCCAGCAGCCGCGGTAATACGTAGGGCGCAAGCGTTGTCCGGAATTATTGGGCGTAAAGAGCTC
GTAGGCGGTTTGTCGCGTCTGCTGTGAAAGCCCGGGGCTCAACCCCGGGTCTGCAGTGGGTACGGGCAGACTAGAGTGCAGTAGGGGAGACTGGAATTCC
TGGTGTAGCGGTGAAATGCGCAGATATCAGGAGGAACACCGATGGCGAAGGCAGGTCTCTGGGCTGTAACTGACGCTGAGGAGCGAAAGCATGGGGAGCG
AACAGGATTAGATACCCTGGTAGTCCATGCCGTAAACGTTGGGCACTAGGTGTGGGGGACATTCCACGTTTTCCGCGCCGTAGCTAACGCATTAAGTGCC
CCGCCTGGGGAGTACGGCCGCAAGGCTAAAACTCAAAGGAATTGACGGGGGCCCGCACAAGCGGCGGAGCATGCGGATTAATTCGATGCAACGCGAAGAA
CCTTACCAAGGCTTGACATGGACCGGACCGCCGCAGAAATGCGGTTTCCCTTCGGGGCTGGTTTACAGGTGGTGCATGGTTGTCGTCAGCTCGTGTCGTG
AGATGTTGGGTTAAGTCCCGCAACGAGCGCAACCCTCGTTCTATGTTGCCAGCGCGTGATGGCGGGGACTCATAGGAGACTGCCGGGGTCAACTCGGAGG
AAGGTGGGGACGACGTCAAATCATCATGCCCCTTATGTCTTGGGCTTCACGCATGCTACAATGGCCGGTACAAAGGGTTGCGATACTGTGAGGTGGAGCT

AATCCCAAAAAGCCGGTCTCAGTTCGGATTGAGGTCTGCAACTCGACCTCATGAAGTCGGAGTCGCTAGTAATCGCAGATCAGCAACGCTGCGGTGAATA
CGTTCCCGGGCCTTGTACACACCGCCCGTCAAGTCACGAAAGTTGGTAACACCCGAAGCCGGTGGCCTAACCCCTTGTGGGAGGGAGCCGTCG

>EB48
TCGAACGATGATCCCAGCTTGCTGGGGGATTAGTGGCGAACGGGTGAGTAACACGTGAGTAACCTGCCCTTGACTCTGGGATAAGCCTGGGAAACTGGGT
CTAATACCGGATATGACCGTCTGACGCATGTCAGGTGGTGGAAAGCTTTTGTGGTTTTGGATGGACTCGCGGCCTATCAGCTTGTTGGTGGGGTAATGGC
CTACCAAGGCGACGACGGGTAGCCGGCCTGAGAGGGTGACCGGCCACACTGGGACTGAGACACGGCCCAGACTCCTACGGGAGGCAGCAGTGGGGAATAT
TGCACAATGGGCGCAAGCCTGATGCAGCGACGCCGCGTGAGGGATGACGGCCTTCGGGTTGTAAACCTCTTTCAGTAGGGAAGAAGCGAAAGTGACGGTA
CCTGCAGAAGAAGCGCCGGCTAACTACGTGCCAGCAGCCGCGGTAATACGTAGGGCGCAAGCGTTATCCGGAATTATTGGGCGTAAAGAGCTCGTAGGCG
GTTTGTCGCGTCTGCTGTGAAAGACCGGGGCTCAACTCCGGTTCTGCAGTGGGTACGGGCAGACTAGAGTGCAGTAGGGGAGACTGGAATTCCTGGTGTA
GCGGTGAAATGCGCAGATATCAGGAGGAACACCGATGGCGAAGGCAGGTCTCTGGGCTGTAACTGACGCTGAGGAGCGAAAGCATGGGGAGCGAACAGGA
TTAGATACCCTGGTAGTCCATGCCGTAAACGTTGGGCACTAGGTGTGGGGGACATTCCACGTTTTCCGCGCCGTAGCTAACGCATTAAGTGCCCCGCCTG
GGGAGTACGGCCGCAAGGCTAAAACTCAAAGGAATTGACGGGGGCCCGCACAAGCGGCGGAGCATGCGGATTAATTCGATGCAACGCGAAGAACCTTACC
AAGGCTTGACATGAACCGGTAATACCTGGAAACAGGTGCCCCGCTTGCGGTCGGTTTACAGGTGGTGCATGGTTGTCGTCAGCTCGTGTCGTGAGATGTT
GGGTTAAGTCCCGCAACGAGCGCAACCCTCGTTCTATGTTGCCAGCGCGTTATGGCGGGGACTCATAGGAGACTGCCGGGGTCAACTCGGAGGAAGGTGG
GGACGACGTCAAATCATCATGCCCCTTATGTCTTGGGCTTCACGCATGCTACAATGGCCGGTACAAAGGGTTGCGATACTGTGAGGTGGAGCTAATCCCA
AAAAGCCGGTCTCAGTTCGGATTGGGGTCTGCAACTCGACCCCATGAAGTCGGAGTCGCTAGTAATCGCAGATCAGCAACGCTGCGGTGAATACGTTCCC
GGGCCTTGTACACACCGCCCGTCAAGTCACGAAAGTTGGTAACACCCGAAGCCGGTGGCCTAACCCTTG

>EB49
TGCAGTCGAACGATGAAGACTCCTGCTTGCAGGGGTTGGATTAGTGGCGAACGGGTGAGTATCACGTGAGTAACCTTCCCTTGACTCTGGGATAAGCCCG
GGAAACTGGGTCTAATACTGGATATGACCTTCCATCGCATGGTGGGGGGTGGAAAGATTTATCGGTCTTGGATGGACTCGCGGCCTATCAGCTTGTTGGT
GAGGTAATGGCTCACCAAGGCGACGACGGGTAGCCGGCCTGAGAGGGTGACCGGCCACACTGGGACTGAGACACGGCCCAGACTCCTACGGGAGGCAGCA
GTGGGGAATATTGCACAATGGGCGCAAGCCTGATGCAGCGACGCCGCGTGAGGGATGACGGCCTTCGGGTTGTAAACCTCTTTCAGTAGGGAAGAAGCCT
TCGGGTGACGGTACCTGCAGAAGAAGCGCCGGCTAACTACGTGCCAGCAGCCGCGGTAATACGTAGGGCGCGAGCGTTATCCGGAATTATTGGGCGTAAA
GAGCTCGTAGGCGGTTTGTCGCGTCTGCTGTGAAAGCCCGGGGCTTAACTCCGGGTGTGCAGTGGGTACGGGCAGACTAGAGTGCAGTAGGGGAGACTGG
AATTCCTGGTGTAGCGGTGAAATGCGCAGATATCAGGAGGAACACCGATGGCGAAGGCAGGTCTCTGGGCTGTTACTGACGCTGAGGAGCGAAAGCATGG
GGAGCGAACAGGATTAGATACCCTGGTAGTCCATGCCGTAAACGTTGGGCACTAGGTGTGGGGGACATTCCACGTTTTCCGCGCCGTAGCTAACGCATTA
AGTGCCCCGCCTGGGGAGTACGGCCGCAAGGCTAAAACTCAAAGGAATTGACGGGGGCCCGCACAAGCGGCGGAGCATGCGGATTAATTCGATGCAACGC
GAAGAACCTTACCAAGGGCTTGACATGTGCCAGACCGCCATAGAGATATGGTTTCCCTTTTTGGGGCTGGTTCACAGGTGGTGCATGGTTGTCGTCAGCT
CGTGTCGTGAGATGTTGGGTTAAGTCCCGCAACGAGCGCAACCCTCGTTCCATGTTGCCAGCGGGTTATGCCGGGGACTCATGGGAGACTGCCGGGGTCA
ACTCGGAGGAAGGTGGGGACGACGTCAAATCATCATGCCCCTTATGTCTTGGGCTTCACGCATGCTACAATGGCCGGTACAATGGGTTGCGATACTGTGA
GGTTGAGCTAATCCCAAAAAGCCGGTCTCAGTTCGGATTGGGGTCTGCAACTCGACCCCATGAAGTCGGAGTCGCTAGTAATCGCAGATCAGCAACGCTG
CGGTGAATACGTTCCCGGGCCTTGTACACACCGCCCGTCAAGTCACGAAAGTTGGTAACACCCGAAGCCGGTGGCCTAACCCTTGTGGGGGGAGCCGT

>EB50
GGTTAGACTACCTACTTCTGGTGCAACAAACTCCCATGGTGTGACGGGCGGTGTGTACAAGGCCCGGGAACGTATTCACCGCGGCATTCTGATCCGCGAT
TACTAGCGATTCCGACTTCATGGAGTCGAGTTGCAGACTCCAATCCGGACTACGATCGGCTTTTTGAGATTAGCATCCTCTCGCGAGGTAGCAACCCTTT
GTACCGACCATTGTAGCACGTGTGTAGCCCTGGTCGTAAGGGCCATGATGACTTGACGTCGTCCCCGCCTTCCTCCAGTTTGTCACTGGCAGTATCCTTA
AAGTTCCCGGCTTAACCCGCTGGCAAATAAGGAAAAGGGTTGCGCTCGTTGCGGGACTTAACCCAACATCTCACGACACGAGCTGACGACAGCCATGCAG
CACCTGTATGTAAGTTCCCGAAGGCACCAATCCATCTCTGGAAAGTTCTTACTATGTCAAGACCAGGTAAGGTTCTTCGCGTTGCATCGAATTAAACCAC
ATGCTCCACCGCTTGTGCGGGCCCCCGTCAATTCATTTGAGTTTTAGTCTTGCGACCGTACTCCCCAGGCGGTCTACTTATCGCGTTAGCTGCGCCACTA
AAGCCTCAAAGGCCCCAACGGCTAGTAGACATCGTTTACGGCATGGACTACCAGGGTATCTAATCCTGTTTGCTCCCCATGCTTTCGCACCTCAGTGTCA
GTATTAGGCCAGATGGCTGCCTTCGCCATCGGTATTCCTCCAGATCTCTACGCATTTCACCGCTACACCTGGAATTCTACCATCCTCTCCCATACTCTAG
CCAACCAGTATCGAATGCAATTCCCAAGTTAAGCTCGGGGATTTCACATTTGACTTAATTGGCCACCTACGCGCGCTTTACGCCCAGTAAATCCGATTAA
CGCTTGCACCCTCTGTATTACCGCGGCTGCTGGCACAGAGTTAGCCGGTGCTTATTCTGCGAGTAACGTCCACTATCCAAGAGTATTAATCTCGGTAGCC
TCCTCCTCGCTTAAAGTGCTTTACAACCAAAAGGCCTTCTTCACACACGCGGCATGGCTGGATCAGGGTTCCCCCCATTGTCCAATATTCCCCACTGCTG
CCTCCCGTAGGAGTCTGGGCCGTGTCTCAGTCCCAGTGTGGCGGATCATCCTCTCAGACCCGCTACAGATCGTCGCCTTGGTAGGCCTTTACCCCACCAA
CTAGCTAATCCGACTTAGGCTCATCTATTAGCGCAAGGCCCGAAGGTCCCCTGCTTTCTCCCGTAGGACGTATGCGGTATTAGCATCCCTTTCGAGATGT
TGTCCCCCACTAATAGGCAGATTCCTAAGCATTACTCACCCGTCCGCCGCTAGGTAATGTAGCAAGCTACATTTCCCCGCTCGACTTGCA

>EB51
TGCAGTCGAACGATGATGGGAGCTTGCTCCTGGATTAGTGGCGAACGGGTGAGTAACACGTGAGTAACCTGCCCTTAACTCTGGGATAAGCCTGGGAAAC
TGGGTCTAATACCGGATATGACTCCTCATCGCATGGTGGGGGGTGGAAAGCTTTATTGTGGTTTTGGATGGACTCGCGGCCTATCAGCTTGTTGGTGAGG
TAATGGCTTACCAAGGCGACGACGGGTAGCCGGCCTGAGAGGGTGACCGGCCACACTGGGACTGAGACACGGCCCAGACTCCTACGGGAGGCAGCAGTGG
GGAATATTGCACAATGGGCGCAAGCCTGATGCAGCGACGCCGCGTGAGGGATGACGGCCTTCGGGTTGTAAACCTCTTTCAGTAGGGAAGAAGCGAAAGT
GACGGTACCTGCAGAAGAAGCGCCGGCTAACTACGTGCCCAGCAGCCGCGGTAATACGTAGGGCGCAAGCGTTATCCGGAATTATTGGGCGTAAAGAGCT
CGTAGGCGGTTTGTCGCGTCTGCCGTGAAAGTCCGGGGCTCAACTCCGGATCTGCGGTGGGTACGGGCAGACTAGAGTGATGTAGGGGAGACTGGAATTC
CTGGTGTAGCGGTGAAATGCGCAGATATCAGGAGGAACACCGATGGCGAAGGCAGGTCTCTGGGCATTAACTGACGCTGAGGAGCGAAAGCATGGGGAGC
GAACAGGATTAGATACCCTGGTAGTCCATGCCGTAAACGTTGGGCACTAGGTGTGGGGGACATTCCACGTTTTCCGCGCCGTAGCTAACGCATTAAGTGC
CCCGCCTGGGGAGTACGGCCGCAAGGCTAAAACTCAAAGGAATTGACGGGGGGCCCGCACAAGCGGCGGAGCATGCGGATTAATTCGATGCAACGCGAAG
AACCTTACCAAGGCTTGACATGAACCGGTAATACCTGGAGACAGGTGCCCCGCTTGCGGTCGGTTTACAGGTGGTGCATGGTTGTCGTCAGCTCGTGTCG
TGAGATGTTGGGTTAAGTCCCGCAACGAGCGCAACCCTCGTTCTATGTTGCCAGCACGTGATGGTGGGGACTCATAGGAGACTGCCGGGGTCAACTCGGA
GGAAGGTGGGGACGACGTCAAATCATCATGCCCCTTATGTCTTGGGCTTCACGCATGCTACAATGGCCGGTACAAAGGGTTGCGATACTGTGAGGTGGAG
CTAATCCCAAAAAGCCGGTCTCAGTTCGGATTGGGGTCTGCAACTCGACCCCATGAAGTCGGAGTCGCTAGTAATCGCAGATCAGCAACGCTGCGGTGAA
TACGTTCCCGGGCCTTGTACACACCGCCCGTCAAGTCACGAAAGTTGGTAACACCCGAAGCCGGTGGCCTAACCCCTTGTGGGAGGGAGC

>EB52
CGATGATGGGAGCTTGCTCCTGGATTAGTGGCGAACGGGTGAGTAACACGTGAGTAACCTGCCCTTAACTCTGGGATAAGCCTGGGAAACTGGGTCTAAT
ACCGGATATGACTCCTCATCGCATGGTGGGGGGTGGAAAGCTTTTTGTGGTTTTGGATGGACTCGCGGCCTATCAGCTTGTTGGTGAGGTAATGGCTTAC
CAAGGCGACGACGGGTAGCCGGCCTGAGAGGGTGACCGGCCACACTGGGACTGAGACACGGCCCAGACTCCTACGGGAGGCAGCAGTGGGGAATATTGCA
CAATGGGCGCAAGCCTGATGCAGCGACGCCGCGTGAGGGATGACGGCCTTCGGGTTGTAAACCTCTTTCAGTAGGGAAGAAGCGAAAGTGACGGTACCTG
CAGAAGAAGCGCCGGCTAACTACGTGCCAGCAGCCGCGGTAATACGTAGGGCGCAAGCGTTATCCGGAATTATTGGGCGTAAAGAGCTCGTAGGCGGTTT
GTCGCGTCTGCCGTGAAAGTCCGGGGCTCAACTCCGGATCTGCGGTGGGTACGGGCAGACTAGAGTGATGTAGGGGAGACTGGAATTCCTGGTGTAGCGG
TGAAATGCGCAGATATCAGGAGGAACACCGATGGCGAAGGCAGGTCTCTGGGCATTAACTGACGCTGAGGAGCGAAAGCATGGGGAGCGAACAGGATTAG
ATACCCCTGGTAGTCCATGCCGTAAACGTTGGGCACTAGGTGTGGGGGACATTCCACGTTTTCCGCGCCGTAGCTAACGCATTAAGTGCCCCGCCTGGGG
AGTACGGCCGCAAGGCTAAAACTCAAAGGAATTGACGGGGGCCCGCACAAGCGGCGGAGCATGCGGATTAATTCGATGCAACGCGAAGAACCTTACCAAG
GCTTGACATGAACCGGTAATACCTGGAGACAGGTGCCCCGCTTGCGGTCGGTTTACAGGTGGTGCATGGTTGTCGTCAGCTCGTGTCGTGAGATGTTGGG

TTAAGTCCCGCAACGAGCGCAACCCTCGTTCTATGTTGCCAGCACGTGATGGTGGGGACTCATAGGAGACTGCCGGGGTCAACTCGGAGGAAGGTGGGGA
CGACGTCAAATCATCATGCCCCTTATGTCTTGGGCTTCACGCATGCTACAATGGCCGGTACAAAGGGTTGCGATACTGTGAGGTGGAGCTAATCCCAAAA
AGCCGGTCTCAGTTCGGATTGGGGTCTGCAACTCGACCCCATGAAGTCGGAGTCGCTAGTAATCGCAGATCAGCAACGCTGCGGTGAATACGTTCCCGGG
CCTTGTACACACCGCCCGTCAAGTCACGAAAGTTGGTAACACCCGAAGCCGGTGGCCTAACCCCTTG

>EB53
GACTCCCCCCACACAAGGTGGTTAGGCCATCGGCTTCGGGTGTTACCAACTTTCGTGACTTGACGGGCGGTGTGTACAAGGCCCGGGAACGTATTCACCG
CAGCGTTGCTGATCTGCGATTACTAGCGACTCCGACTTCATGGGGTCGAGTTGCAGACCCCAATCCGAACTGAGACCGGCTTTTAGGGATTAGCTCCACC
TCACAGTATCGCAACCCATTGTACCGGCCATTGTAGCATGCGTGAAGCCCAAGACATAAGGGGCATGATGATTTGACGTCATCCCCACCTTCCTCCGAGT
TGACCCCGGCAGTCTCCCATGAGTCCCCACCACTACGTGCTGGCAACATGGAACGAGGGTTGCGCTCGTTGCGGGACTTAACCCAACATCTCACGACACG
AGCTGACGACAACCATGCACCACCTGTGAACCAGCCCCGAAGGGAAACCCCATCTCTGGAGCGGTCTGGCACATGTCAAGCCTTGGTAAGGTTCTTCGCG
TTGCATCGAATTAATCCGCATGCTCCGCCGCTTGTGCGGGCCCCCGTCAATTCCTTTGAGTTTTAGCCTTGCGGCCGTACTCCCCAGGCGGGGCACTTAA
TGCGTTAGCTACGGCGCGGAAAACGTGGAATGTCCCCCACACCTAGTGCCCAACGTTTACGGCATGGACTACCAGGGTATCTAATCCTGTTCGCTCCCCA
TGCTTTCGCTCCTCAGCGTCAGTAAATGCCCAGAGACCTGCCTTCGCCATCGGTGTTCCTCCTGATATCTGCGCATTTCACCGCTACACCAGGAATTCCA
GTCTCCCCTACATCACTCTAGTCTGCCCGTACCCACCGCAGATCCGAGGTTGAGCCTCGGACTTTCACGGCAGACGCGACAAACCGCCTACGAGCTCTTT
ACGCCCAATAAATCCGGATAACGCTTGCGCCCTACGTATTACCGCGGCTGCTGGCACGTAGTTAGCCGGCGCTTCTTCTGCAGGTACCGTCACTTTCGCT
TCTTCCCTACTGAAAGAGGTTTACAACCCGAAGGCCGTCATCCCTCACGCGGCGTCGCTGCATCAGGCTTCCGCCCATTGTGCAATATTCCCCACTGCTG
CCTCCCGTAGGAGTCTGGGCCGTGTCTCAGTCCCAGTGTGGCCGGTCACCCTCTCAGGCCGGCTACCCGTCGTCGCCTTGGTGAGCCATTACCTCACCAA
CAAGCTGATAGGCCGCGAGTCCATCCCCCACCGATAAATCTTTCAACAACCCACCATGCGGCAGGAAGTCATATCCGGTATTAGACCCAGTTTCCCGGGC
TTATCCCAAAGTCGGGGGCAGGTTACTCACGTGTTACTCACCCGTTCGCCACTAATCCACCCAGCAAGCTGGGCTTCATCGTTCGACTG

>EB54
GGGGGGGCGGCGCCTCTATCCTGCAAGTCGAACGATGATGGGAGCTTGCTCCTGGATTAGTGGCGAACGGGTGAGTAACACGTGAGTAACCTGCCCTTAA
CTCTGGGATAAGCCTGGGAAACTGGGTCTAATACCGGATATGACTCCTCATCGCATGGTGGGGGGTGGAAAGCTTTATTGTGGTTTTGGATGGACTCGCG
GCCTATCAGCTTGTTGGTGAGGTAATGGCTTACCAAGGCGACGACGGGTAGCCGGCCTGAGAGGGTGACCGGCCACACTGGGACTGAGACACGGCCCAGA
CTCCTACGGGAGGCAGCAGTGGGGAATATTGCACAATGGGCGCAAGCCTGATGCAGCGACGCCGCGTGAGGGATGACGGCCTTCGGGTTGTAAACCTCTT
TCAGTAGGGAAGAAGCGAAAGTGACGGTACCTGCAGAAGAAGCGCCGGCTAACTACGTGCCAGCAGCCGCGGTAATACGTAGGGCGCAAGCGTTATCCGG
AATTATTGGGCGTAAAGAGCTCGTAGGCGGTTTGTCGCGTCTGCCGTGAAAGTCCGGGGCTCAACTCCGGATCTGCGGTGGGTACGGGCAGACTAGAGTG
ATGTAGGGGAGACTGGAATTCCTGGTGTAGCGGTGAAATGCGCAGATATCAGGAGGAACACCGATGGCGAAGGCAGGTCTCTGGGCATTAACTGACGCTG
AGGAGCGAAAGCATGGGGAGCGAACAGGATTAGATACCCTGGTAGTCCATGCCGTAAACGTTGGGCACTAGGTGTGGGGGACATTCCACGTTTTCCGCGC
CGTAGCTAACGCATTAAGTGCCCCGCCTGGGGAGTACGGCCGCAAGGCTAAAACTCAAAGGAATTGACGGGGGCCCGCACAAGCGGCGGAGCATGCGGAT
TAATTCGATGCAACGCGAAGAACCTTACCAAGGCTTGACATGAACCGGTAATACCTGGAGACAGGTGCCCCGCTTGCGGTCGGTTTACAGGTGGTGCATG
GTTGTCGTCAGCTCGTGTCGTGAGATGTTGGGTTAAGTCCCGCAACGAGCGCAACCCTCGTTCTATGTTGCCAGCACGTGATGGTGGGGACTCATAGGAG
ACTGCCGGGGTCAACTCGGAGGAAGGTGGGGACGACGTCAAATCATCATGCCCCTTATGTCTTGGGCTTCACGCATGCTACAATGGCCGGTACAAAGGGT
TGCGATACTGTGAGGTGGAGCTAATCCCAAAAAGCCGGTCTCAGTTCGGATTGGGGTCTGCAACTCGACCCCATGAAGTCGGAGTCGCTAGTAATCGCAG
ATCAGCAACGCTGCGGTGAATACGTTCCCGGGCCTTGTACACACCGCCCGTCAAGTCACGAAAGTTGGTAACACCCGAAGCCGGTGGCCTAACCCCTTGT
GGGAGGGAGCTGTCGAAGGTGGGACTGGCGATGGGACTAAGTCGCGGCGATGTGGCGGCCCGGCC

>EB55
ACATGCAAGTCGAGCGGACAGAAGGGAGCTTGCTCCCGGATGTTAGCGGCGGACGGGTGAGTAACACGTGGGTAACCTGCCTGTAAGACTGGGATAACTC
CGGGAAACCGGAGCTAATACCGGATAGTTCCTTGAACCGCATGGTTCAAGGATGAAAGACGGTTTCGGCTGTCACTTACAGATGGACCCGCGGCGCATTA
GCTAGTTGGTGAGGTAACGGCTCACCAAGGCGACGATGCGTAGCCGACCTGAGAGGGTGATCGGCCACACTGGGACTGAGACACGGCCCAGACTCCTACG
GGAGGCAGCAGTAGGGAATCTTCCGCAATGGACGAAAGTCTGACGGAGCAACGCCGCGTGAGTGATGAAGGTTTTCGGATCGTAAAGCTCTGTTGTTAGG
GAAGAACAAGTGCAAGAGTAACTGCTTGCACCTTGACGGTACCTAACCAGAAAGCCACGGCTAACTACGTGCCAGCAGCCGCGGTAATACGTAGGTGGCA
AGCGTTGTCCGGAATTATTGGGCGTAAAGGGCTCGCAGGCGGTTTCTTAAGTCTGATGTGAAAGCCCCCGGCTCAACCGGGGAGGGTCATTGGAAACTGG
GAAACTTGAGTGCAGAAGAGGAGAGTGGAATTCCACGTGTAGCGGTGAAATGCGTAGAGATGTGGAGGAACACCAGTGGCGAAGGCGACTCTCTGGTCTG
TAACTGACGCTGAGGAGCGAAAGCGTGGGGAGCGAACAGGATTAGATACCCTGGTAGTCCACGCCGTAAACGATGAGTGCTAAGTGTTAGGGGGTTTCCG
CCCCTTAGTGCTGCAGCTAACGCATTAAGCACTCCGCCTGGGGAGTACGGTCGCAAGACTGAAACTCAAAGGAATTGACGGGGGCCCGCACAAGCGGTGG
AGCATGTGGGTTTAATTCGAAGCAACGCGAAGAACCTTACCAGGTCTTGACATTCTCTGACAACCCTAGAGATAGGGCTTTCCCTTCGGGGACAGAGTG
ACAGGTGGTGCATGGTTGTCGTCAGCTCGTGTCGTGAGATGTTGGGTTAAGTCCCGCAACGAGCGCAACCCTTGATCTTAGTTGCCAGCATTCAGTTGGG
CACTCTAAGGTGACTGCCGGTGACAAACCGGAGGAAGGTGGGGATGACGTCAAATCATCATGCCCCTTATGACCTGGGCTACACACGTGCTACAATGGAC
AGAACAAAGGGCTGCGAGACCGCAAGGTTTAGCCAATCCCACAAATCTGTTCTCAGTTCGGATCGCAGTCTGCAACTCGACTGCGTGAAGCTGGAATCGC
TAGTAATCGCGGATCAGCATGCCGCGGTGAATACGTTCCCGGGCCTTGTACACACCGCCCGTCACACCACGAGAGTTTGCAACACCCGAAGTCGGTGAGG
TAACCTTTATGGAGCCAGCCG

>EB56
GTCGAGCGGACAGAAGGGAGCTTGCTCCCGGATGTTAGCGGCGGACGGGTGAGTAACACGTGGGTAACCTGCCTGTAAGACTGGGATAACTCCGGGAAAC
CGGAGCTAATACCGGATAGTTCCTTGAACCGCATGGTTCAAGGATGAAAGACGGTTTCGGCTGTCACTTACAGATGGACCCGCGGCGCATTAGCTAGTTG
GTGAGGTAACGGCTCACCAAGGCGACGATGCGTAGCCGACCTGAGAGGGTGATCGGCCACACTGGGACTGAGACACGGCCCAGACTCCTACGGGAGGCAG
CAGTAGGGAATCTTCCGCAATGGACGAAAGTCTGACGGAGCAACGCCGCGTGAGTGATGAAGGTTTTCGGATCGTAAAGCTCTGTTGTTAGGGAAGAACA
AGTGCAAGAGTAACTGCTTGCACCTTGACGGTACCTAACCAGAAAGCCACGGCTAACTACGTGCCAGCAGCCGCGGTAATACGTAGGTGGCAAGCGTTGT
CCGGAATTATTGGGCGTAAAGGGCTCGCAGGCGGTTTCTTAAGTCTGATGTGAAAGCCCCCGGCTCAACCGGGGAGGGTCATTGGAAACTGGGAAACTTG
AGTGCAGAAGAGGAGAGTGGAATTCCACGTGTAGCGGTGAAATGCGTAGAGATGTGGAGGAACACCAGTGGCGAAGGCGACTCTCTGGTCTGTAACTGAC
GCTGAGGAGCGAAAGCGTGGGGAGCGAACAGGATTAGATACCCTGGTAGTCCACGCCGTAAACGATGAGTGCTAAGTGTTAGGGGGTTTCCGCCCCTTAG
TGCTGCAGCTAACGCATTAAGCACTCCGCCTGGGGAGTACGGTCGCAAGACTGAAACTCAAAGGAATTGACGGGGGCCCGCACAAGCGGTGGAGCATGTG
GTTTAATTCGAAGCAACGCGAAGAACCTTACCAGGTCTTGACATCCTCTGACAACCCTAGAGATAGGGCTTTCCCTTCGGGGACAGAGTGACAGGTGGTG
CATGGTTGTCGTCAGCTCGTGTCGTGAGATGTTGGGTTAAGTCCCGCAACGAGCGCAACCCTTGATCTTAGTTGCCAGCATTCAGTTGGGCACTCTAAGG
TGACTGCCGGTGACAAACCGGAGGAAGGTGGGGATGACGTCAAATCATCATGCCCCTTATGACCTGGGCTACACACGTGCTACAATGGACAGAACAAAGG
GCTGCGAGACCGCAAGGTTTAGCCAATCCCACAAATCTGTTCTCAGTTCGGATCGCAGTCTGCAACTCGACTGCGTGAAGCTGGAATCGCTAGTAATCGC
GGATCAGCATGCCGCGGTGAATACGTTCCCGGGCCTTGTACACACCGCCCGTCACACCACGAGAGTTTGCAACACCCGAAGTCGGTGAGGTAACCTTTAT
GGAGCCAGCCG

>EB57
TGCAAGTCGAGCGGACAGATGGGAGCTTGCTCCCTGATGTTAGCGGCGGACGGGTGAGTAACACGTGGGTAACCTGCCTGTAAGACTGGGATAACTCCGG
GAAACCGGGGCTAATACCGGATGGTTGTCTGAACCGCATGGTTCAGACATAAAAGGTGGCTTCGGCTACCACTTACAGATGGACCCGCGGCGCATTAGCT
AGTTGGTGAGGTAACGGCTCACCAAGGCGACGATGCGTAGCCGACCTGAGAGGGTGATCGGCCACACTGGGACTGAGACACGGCCCAGACTCCTACGGGA
GGCAGCAGTAGGGAATCTTCCGCAATGGACGAAAGTCTGACGGAGCAACGCCGCGTGAGTGATGAAGGTTTTCGGATCGTAAAGCTCTGTTGTTAGGGAA
GAACAAGTGCCGTTCAAATAGGGCGGCACCTTGACGGTACCTAACCAGAAAGCCACGGCTAACTACGTGCCAGCAGCCGCGGTAATACGTAGGTGGCAAG

CGTTGTCCGGAATTATTGGGCGTAAAGGGCTCGCAGGCGGTTTCTTAAGTCTGATGTGAAAGCCCCCGGCTCAACCGGGGAGGGTCATTGGAAACTGGGG
AACTTGAGTGCAGAAGAGGAGAGTGGAATTCCACGTGTAGCGGTGAAATGCGTAGAGATGTGGAGGAACACCAGTGGCGAAGGCGACTCTCTGGTCTGTA
ACTGACGCTGAGGAGCGAAAGCGTGGGGAGCGAACAGGATTAGATACCCTGGTAGTCCACGCCGTAAACGATGAGTGCTAAGTGTTAGGGGGTTTCCGCC
CCTTAGTGCTGCAGCTAACGCATTAAGCACTCCGCCTGGGGAGTACGGTCGCAAGACTGAAACTCAAAGGAATTGACGGGGGCCCGCACAAGCGGTGGAG
CATGTGGTTTAATTCGAAGCAACGCGAAGAACCTTACCAGGTCTTGACATCCTCTGACAATCCTAGAGATAGGACGTCCCCTTCGGGGGCAGAGTGACAG
GTGGTGCATGGTTGTCGTCAGCTCGTGTCGTGAGATGTTGGGTTAAGTCCCGCAACGAGCGCAACCCTTGATCTTAGTTGCCAGCATTCAGTTGGGCACT
CTAAGGTGACTGCCGGTGACAAACCGGAGGAAGGTGGGGATGACGTCAAATCATCATGCCCCTTATGACCTGGGCTACACACGTGCTACAATGGACAGAA
CAAAGGGCAGCGAAACCGCGAGGTTAAGCCAATCCCACAAATCTGTTCTCAGTTCGGATCGCAGTCTGCAACTCGACTGCGTGAAGCTGGAATCGCTAGT
AATCGCGGATCAGCATGCCGCGGTGAATACGTTCCCGGGCCTTGTACACACCGCCCGTCACACCACGAGAGTTTGTAACACCCGAAGTCGGTGAGGTAAC
CTTTATGGAGCCAG

>EB58
TGCTCAGGATGAACGCTGGCGGCGTGCTTAACACATGCAAGTCGAACGATGATCCCAGCTTGCTGGGGGATTAGTGGCGAACGGGTGAGTAACACGTGAG
TAACCTGCCCTTGACTCTGGGATAAGCCTGGGAAACTGGGTCTAATACCGGATACGACCATTCCACGCATGTGGTGGTGGTGGAAAGCTTTTGTGGTTTT
GGATGGACTCGCGGCCTATCAGCTTGTTGGTGGGGTAATGGCCTACCAAGGCGACGACGGGTAGCCGGCCTGAGAGGGTGACCGGCCACACTGGGACTGA
GACACGGCCCAGACTCCTACGGGAGGCAGCAGTGGGGAATATTGCACAATGGGCGCAAGCCTGATGCAGCGACGCCGCGTGAGGGATGACGGCCTTCGGG
TTGTAAACCTCTTTCAGTAGGGAAGAAGCGTAAGTGACGGTACCTGCAGAAGAAGCGCCGGCTAACTACGTGCCAGCAGCCGCGGTAATACGTAGGGCGC
AAGCGTTATCCGGAATTATTGGGCGTAAAGAGCTCGTAGGCGGTTTGTCGCGTCTGCTGTGAAAGACCGGGGCTCAACTCCGGTTCTGCAGTGGGTACGG
GCAGACTAGAGTGCAGTAGGGGAGACTGGAATTCCTGGTGTAGCGGTGAAATGCGCAGATATCAGGAGGAACACCGATGGCGAAGGCAGGTCTCTGGGCT
GTAACTGACGCTGAGGAGCGAAAGCATGGGGAGCGAACAGGATTAGATACCCTGGTAGTCCATGCCGTAAACGTTGGGCACTAGGTGTGGGGGACATTCC
ACGTTTTCCGCGCCGTAGCTAACGCATTAAGTGCCCCGCCTGGGGAGTACGGCCGCAAGGCTAAAACTCAAAGGAATTGACGGGGGCCCGCACAAGCGGC
GGAGCATGCGGATTAATTCGATGCAACGCGAAGAACCTTACCAAGGCTTGACATGAACCGGAAAGACCTGGAAACAGGTGCCCCGCTTGCGGTCGGTTTA
CAGGTGGTGCATGGTTGTCGTCAGCTCGTGTCGTGAGATGTTGGGTTAAGTCCCGCAACGAGCGCAACCCTCGTTCTATGTTGCCAGCGCGTGATGGCGG
GGACTCATAGGAGACTGCCGGGGTCAACTCGGAGGAAGGTGGGGACGACGTCAAATCATCATGCCCCTTATGTCTTGGGCTTCACGCATGCTACAATGGC
CGGTACAAAGGGTTGCGATACTGTGAGGTGGAGCTAATCCCAAAAAGCCGGTCTCAGTTCGGATTGGGGTCTGCAACTCGACCCCATGAAGTCGGAGTCG
CTAGTAATCGCAGATCAGCAACGCTGCGGTGAATACGTTCCCGGGCCTTGTACACACCGCCCGTCAAGTCACGAAAGTTGGTAACACCCGAAGCCGGTGG
CCTAACCCTTGTGGGGGGAGCCGTCGAAGGTGGGACCGGCGATTGGGACTAAGTCTACAAGGGG

>EB59
TGCAGTCGAGCGGATCGATGGGAGCTTGCTCCCTGAGATCAGCGGCGGACGGGTGAGTAACACGTGGGTAACCTGCCTGTAAGACTGGGATAACTCCGGG
AAACCGGGGCTAATACCGGATAACACCTACCCCCGCATGGGGGAAGGTTGAAAGGTGGCTTCGGCTATCACTTACAGATGGACCCGCGGCGCATTAGCTA
GTTGGTGAGGTAATGGCTCACCAAGGCGACGATGCGTAGCCGACCTGAGAGGGTGATCGGCCACACTGGGACTGAGACACGGCCCAGACTCCTACGGGAG
GCAGCAGTAGGGAATCTTCCGCAATGGACGAAAGTCTGACGGAGCAACGCCGCGTGAGTGAAGAAGGTTTTCGGATCGTAAAACTCTGTTGTTAGGGAAG
AACAAGTGCCGTTCGAATAGGGCGGCGCCTTGACGGTACCTAACCAGAAAGCCACGGCTAACTACGTGCCAGCAGCCGCGGTAATACGTAGGTGGCAAGC
GTTGTCCGGAATTATTGGGCGTAAAGCGCGCGCAGGTGGTTTCTTAAGTCTGATGTGAAAGCCCACGGCTCAACCGTGGAGGGTCATTGGAAACTGGGGA
ACTTGAGTGCAGAAGAGGAAAGTGGAATTCCAAGTGTAGCGGTGAAATGCGTAGATATTTGGAGGAACACCAGTGGCGAAGGCGACTTTCTGGTCTGTAA
CTGACACTGAGGCGCGAAAGCGTGGGGAGCAAACAGGATTAGATACCCTGGTAGTCCACGCCGTAAACGATGAGTGCTAAGTGTTAGAGGGTTTCCGCCC
TTTAGTGCTGCAGCTAACGCATTAAGCACTCCGCCTGGGGAGTACGGTCGCAAGACTGAAACTCAAAGGAATTGACGGGGGCCCGCACAAGCGGTGGAGC
ATGTGGTTTAATTCGAAGCAACGCGAAGAACCTTACCAGGTCTTGACATCCTCTGACAACCCTAGAGATAGGGCTTTCCCCTTCGGGGGACAGAGTGACA
GGTGGTGCATGGTTGTCGTCAGCTCGTGTCGTGAGATGTTGGGTTAAGTCCCGCAACGAGCGCAACCCTTGATCTTAGTTGCCAGCATTCAGTTGGGCAC
TCTAAGATGACTGCCGGTGACAAACCGGAGGAAGGTGGGGATGACGTCAAATCATCATGCCCCTTATGACCTGGGCTACACACGTGCTACAATGGACGGT
ACAAAGGGCTGCAAGACCGCGAGGTTTAGCCAATCCCATAAAACCGTTCTCAGTTCGGATTGTAGGCTGCAACTCGCCTACATGAAGCTGGAATCGCTAG
TAATCGCGGATCAGCATGCCGCGGTGAATACGTTCCCGGGCCTTGTACACACCGCCCGTCACACCACGAGAGTTTGTAACACCCGAAGTCGGTGAGGTAA
CCTTTGGAGCCAGCCGCCT

>EB60
AGCGCAGCGCCTTCCTATAGAACCAACTCCCATGGTGTGACGGGCGGTGTGTACAAGGCCCGGGAACGTATTCACCGCGGCATGCTGATCCGCGATTACT
AGCGATTCCAACTTCATGCCCTCGAGTTGCAGAGGACAATCCGAACTGAGACGACTTTTAAGGATTAACCCTCTGTAGTCGCCATTGTAGCACGTGTGTA
GCCCACCCTGTAAGGGCCATGAGGACTTGACGTCATCCCCACCTTNCCTCCGGCTTAGCACCGGCAGTCCCATTAGAGTTCCCAACTAAATGATGGCAAC
TAATGGCGAGGGTTGCGCTCGTTGCGGGACTTAACCCAACATCTCACGACACGAGCTGACGACAGCCATGCAGCACCTGTGTCCTAGTCCCCGAAGGGA
AAGCCACGTCTCCGTGGCGGTCCAGGCATGTCAAAAGGTGGTAAGGTTCTGCGCGTTGCTTCGAATTAAACCACATGCTCCACCGCTTGTGCGGGCCCCC
GTCAATTCCTTTGAGTTTTAATCTTGCGACCGTACTCCCCAGGCGGATTGCTTAATGCGTTAGCTGCGTCACCGAAATGCATGCATCCCGACAACTAGCA
ATCATCGTTTACGGCGTGGACTACCAGGGTATCTAATCCTGTTTGCTCCCCACGCTTTCGAGCCTCAGCGTCAGTAATGAGCCAGTGTGTCGCCTTCGCC
ACTGGTGTTCTTCCGAATATCTACGAATTTCACCTCTACACTCGGAGTTCCACACACCTCTCTCATACTCAAGACACCCAGTATCAAAGGCAATTCCGAG
GTTGAGCCCCGGGATTTCACCCCTGACTTAAATGTCCGCCTACGCTCCCTTTACGCCCAGTAATTCCGAGCAACGCTAGCCCCCTTCGTATTACCGCGGC
TGCTGGCACGAAGTTAGCCGGGGCTTCTTCTCCGGGTACCGTCATTATCGTCCCCGGTGAAAGAATTTTACAATCCTAAGACCTTCATCATTCACGCGGC
ATGGCTGCGTCAGGCTTTCGCCCATTGCGCAAGATTCCCCACTGCTGCCTCCCGTAGGAGTTTGGGCCGTGTCTCAGTCCCAATGTGGCTGATCATCCTC
TCAGACCAGCTACTGATCGTCGCCTTGGTGAGCCTTTACCTCACCAACTAGCTAATCAGACGCGGGCCGCTCTAAAGGCGATAAATCTTTCCCCCGAAGG
GCACATTCGGTATTAGCACAAGTTTCCCTGAGTTATTCCGAACCTAAAGGCACGTTCCCACGTGTTACTCACCCGTCCGCCACTAACTCCGAAGAGTTCG

>EB61
TGCAAGTCGAGCGAATCATTGGGAGCTTGCTCCCTTTGGTTAGCGGCGGACGGGTGAGTAACACGTGGGCAACCTGCCTGTAAGACTGGGATAACTTCGG
GAAACCGGAGCTAATACCGGATAATCTTTTTCCTCTCATGAGGAAAAACTGAAAGACGGTTTCGGCTGTCACTTACAGATGGGCCCGCGGCGCATTAGCT
AGTTGGTGAGGTAACGGCTCACCAAGGCGACGATGCGTAGCCGACCTGAGAGGGTGATCGGCCACACTGGGACTGAGACACGGCCCAGACTCCTACGGGA
GGCAGCAGTAGGGAATCTTCCACAATGGACGAAAGTCTGATGGAGCAACGCCGCGTGAGCGATGAAGGCCTTCGGGTCGTAAAGCTCTGTTGTTAGGGAA
GAACAAGTATCGGAGTAACTGCCGGTACCTTGACGGTACCTAACCAGAAAGCCACGGCTAACTACGTGCCAGCAGCCGCGGTAATACGTAGGTGGCAAGC
GTTGTCCGGAATTATTGGGCGTAAAGCGCGCGCAGGCGGTCCTTTAAGTCTGATGTGAAAGCCCACGGCTCAACCGTGGAGGGTCATTGGAAACTGGGGG
ACTTGAGTGCAGAAGAGGAAAGCGGAATTCCACGTGTAGCGGTGAAATGCGTAGAGATGTGGAGGAACACCAGTGGCGAAGGCGGCTTTCTGGTCTGTAA
CTGACGCTGAGGCGCGAAAGCGTGGGGAGCAAACAGGATTAGATACCCTGGTAGTCCACGCCGTAAACGATGAGTGCTAAGTGTTAGGGGGTTTCCGCCC
CTTAGTGCTGCAGCTAACGCATTAAGCACTCCGCCTGGGGAGTACGGCCGCAAGGCTGAAACTCAAAGGAATTGACGGGGGCCCGCACAAGCGGTGGAGC
ATGTGGTTTAATTCGAAGCAACGCGAAGAACCTTACCAGGTCTTGACATCCTCTGACACTCCTAGAGATAGGACGTTCCCCTTCGGGGGACAGAGTGACA
GGTGGTGCATGGTTGTCGTCAGCTCGTGTCGTGAGATGTTGGGTTAAGTCCCGCAACGAGCGCAACCCTTGATCTTAGTTGCCAGCATTCAGTTGGGCAC
TCTAAGGTGACTGCCGGTGACAAACCGGAGGAAGGTGGGGATGACGTCAAATCATCATGCCCCTTATGACCTGGGCTACACACGTGCTACAATGGATGGT
ACAAAGGGCTGCAAGACCGCGAGGTTTAGCCAATCCCATAAAACCATTCTCAGTTCGGATTGTAGGCTGCAACTCGCCTACATGAAGCCGGAATCGCTAG
TAATCGCGGATCAGCATGCCGCGGTGAATACGTTCCCGGGCCTTGTACACACCGCCCGTCACACCACGAGAGTTTGTAACACCCGAAGTCGGTGGGGTAA
CCGTAAGGAGCCAGCCGCCT

>EB62

CGGCTGGCTCCATAAAGGTTACCCCACCGACTTCGGGTGTTACAAACTCTCGTGGTGTGACGGGCGGTGTGTACAAGGCCCGGGAACGTATTCACCGCGG
CATGCTGATCCGCGATTACTAGCGATTCCAGCTTCATGTAGGCGAGTTGCAGCCTACAATCCGAACTGAGAACGGTTTTATGAGATTAGCTCCACCTCGC
GGTCTTGCAGCTCTTTGTACCGTCCATTGTAGCACGTGTGTAGCCCAGGTCATAAGGGGCATGATGATTTGACGTCATCCCCACCTTCCTCCGGTTTGTC
ACCGGCAGTCACCTTAGAGTGCCCAACTTAATGATGGCAACTAAGATCAAGGGTTGCGCTCGTTGCGGGACTTAACCCAACATCTCACGACACGAGCTGA
CGACAACCATGCACCACCTGTCACTCTGCTCCCGAAGGAGAAGCCCTATCTCTAGGGTTTTCAGAGGATGTCAAGACCTGGTAAGGTTCTTCGCGTTGCT
TCGAATTAAACCACATGCTCCACCGCTTGTGCGGGCCCCCGTCAATTCCTTTGAGTTTCAGCCTTGCGGCCGTACTCCCCAGGCGGAGTGCTTAATGCGT
TAACTTCAGCACTAAAGGGCGGAAACCCTCTAACACTTAGCACTCATCGTTTACGGCGTGGACTACCAGGGTATCTAATCCTGTTTGCTCCCCACGCTTT
CGCGCCTCAGTGTCAGTTACAGACCAGAAAGTCGCCTTCGCCACTGGTGTTCCTCCATATCTCTACGCATTTCACCGCTACACATGGAATTCCACTTTCC
TCTTCTGCACTCAAGTCTCCCAGTTTCCAATGACCCTCCACGGTTGAGCCGTGGGCTTTCACATCAGACTTAAGAAACCACCTGCGCGCGCTTTACGCCC
AATAATTCCGGATAACGCTTGCCACCTACGTATTACCGCGGCTGCTGGCACGTAGTTAGCCGTGGCTTTCTGGTTAGGTACCGTCAAGGTGCCAGCTTAT
TCAACTAGCACTTGTTCTTCCCTAACAACAGAGTTTTACGACCCGAAAGCCTTCATCACTCACGCGGCGTTGCTCCGTCAGACTTTCGTCCATTGCGGAA
GATTCCCTACTGCTGCCTCCCGTAGGAGTCTGGGCCGTGTCTCAGTCCCAGTGTGGCCGATCACCCTCTCAGGTCGGCTACGCATCGTTGCCTTGGTGAG
CCGTTACCTCACCAACTAGCTAATGCGACGCGGGTCCATCCATAAGTGACAGCCGAAGCCGCCTTTCAATTTCGAACCATGCAGTTCAAAATATTATCCG
GTATTAGCCCCGGTTTCCCGGAGTTATCCCAGTCTTATGGGCAGGTTACCCACGTGTTACTCACCCGTCCGCCGCTAACTTCTTGAGAGCAAGCTCTCAA
TCCATTCGCTCGACTGCA

>EB63
TGCAGTCGAGCGGACTTTAAAAGCTTGCTTTTAAAGTTAGCGGCGGACGGGTGAGTAACACGTGGGCAACCTGCCTGTAAGACTGGGATAACTTCGGGAA
ACCGGAGCTAATACCGGATAATCCTTTTCCTCTCATGAGGAAAAGCTGAAAGACGGTTTACGCTGTCACTTACAGATGGGCCCGCGGCGCATTAGCTAGT
TGGTGAGGTAACGGCTCACCAAGGCGACGATGCGTAGCCGACCTGAGAGGGTGATCGGCCACACTGGGACTGAGACACGGCCCAGACTCCTACGGGAGGC
AGCAGTAGGGAATCTTCCGCAATGGACGAAAGTCTGACGGAGCAACGCCGCGTGAGTGATGAAGGTTTTCGGATCGTAAAACTCTGTTGTTAGGGAAGAA
CAAGTACAAGAGTAACTGCTTGTACCTTGACGGTACCTAACCAGAAAGCCACGGCTAACTACGTGCCAGCAGCCGCGGTAATACGTAGGTGGCAAGCGTT
GTCCGGAATTATTGGGCGTAAAGCGCGCGCAGGCGGTCCTTTAAGTCTGATGTGAAAGCCCACGGCTCAACCGTGGAGGGTCATTGGAAACTGGGGGACT
TGAGTGCAGAAGAGAAGAGTGGAATTCCACGTGTAGCGGTGAAATGCGTAGAGATGTGGAGGAACACCAGTGGCGAAGGCGACTCTTTGGTCTGTAACTG
ACGCTGAGGCGCGAAAGCGTGGGGAGCAAACAGGATTAGATACCCTGGTAGTCCACGCCGTAAACGATGAGTGCTAAGTGTTAGAGGGTTTCCGCCCTTT
AGTGCTGCAGCAAACGCATTAAGCACTCCGCCTGGGGAGTACGGCCGCAAGGCTGAAACTCAAAGGAATTGACGGGGGCCCGCACAAGCGGTGGAGCATG
TGGTTTAATTCGAAGCAACGCGAAGAACCTTACCAGGTCTTGACATCCTCTGACACTCCTAGAGATAGGACGTTCCCCTTCGGGGGACAGAGTGACAGG
TGGTGCATGGTTGTCGTCAGCTCGTGTCGTGAGATGTTGGGTTAAGTCCCGCAACGAGCGCAACCCTTGATCTTAGTTGCCAGCATTCAGTTGGGCACTC
TAAGGTGACTGCCGGTGACAAACCGGAGGAAGGTGGGGATGACGTCAAATCATCATGCCCCTTATGACCTGGGCTACACACGTGCTACAATGGATGGTAC
AAAGGGCAGCAAAACCGCGAGGTCGAGCAAATCCCATAAAACCATTCTCAGTTCGGATTGTAGGCTGCAACTCGCCTACATGAAGCTGGAATCGCTAGTA
ATCGCGGATCAGCATGCCGCGGTGAATACGTTCCCGGGCCTTGTACACACCGCCCGTCACACCACGAGAGTTTGTAACACCCGAAGTCGGTGGGGTAACC
TTTTGGAGCCAGCCG

>EB64
TGCAGTCGAGCGAACTGATTAGAAGCTTGCTTCTATGACGTTAGCGGCGGACGGGTGAGTAACACGTGGGCAACCTGCCTGTAAGACTGGGATAACTTCG
GGAAACCGAAGCTAATACCGGATAGGATCTTCTCCTTCATGGGAGATGATTGAAAGATGGTTTCGGCTATCACTTACAGATGGGCCCGCGGTGCATTAGC
TAGTTGGTGAGGTAACGGCTCACCAAGGCAACGATGCATAGCCGACCTGAGAGGGTGATCGGCCACACTGGGACTGAGACACGGCCCAGACTCCTACGGG
AGGCAGCAGTAGGGAATCTTCCGCAATGGACGAAAGTCTGACGGAGCAACGCCGCGTGAGTGATGAAGGCTTTCGGGTCGTAAAACTCTGTTGTTAGGGA
AGAACAAGTACGAGAGTAACTGCTCGTACCTTGACGGTACCTAACCAGAAAGCCACGGCTAACTACGTGCCAGCAGCCGCGGTAATACGTAGGTGGCAAG
CGTTATCCGGAATTATTGGGCGTAAAGCGCGCGCAGGCGGTTTCTTAAGTCTGATGTGAAAGCCCACGGCTCAACCGTGGAGGGTCATTGGAAACTGGGG
AACTTGAGTGCAGAAGAGAAAAGCGGAATTCCACGTGTAGCGGTGAAATGCGTAGAGATGTGGAGGAACACCAGTGGCGAAGGCGGCTTTTTGGTCTGTA
ACTGACGCTGAGGCGCGAAAGCGTGGGGAGCAAACAGGATTAGATACCCTGGTAGTCCACGCCGTAAACGATGAGTGCTAAGTGTTAGAGGGTTTCCGCC
CTTTAGTGCTGCAGCTAACGCATTAAGCACTCCGCCTGGGGAGTACGGTCGCAAGACTGAAACTCAAAGGAATTGACGGGGGCCCGCACAAGCGGTGGAG
CATGTGGTTTTAATTCGAAGCAACGCGAAGAACCTTACCAGGTCTTGACATCCTCTGACAACTCTAGAGATAGAGCGTTCCCCTTCGGGGGACAGAGTGA
CAGGTGGTGCATGGTTGTCGTCAGCTCGTGTCGTGAGATGTTGGGTTAAGTCCCGCAACGAGCGCAACCCTTGATCTTAGTTGCCAGCATTTAGTTGGGC
ACTCTAAGGTGACTGCCGGTGACAAACCGGAGGAAGGTGGGGATGACGTCAAATCATCATGCCCCTTATGACCTGGGCTACACACGTGCTACAATGGATG
GTACAAAGGGCTGCAAGACCGCGAGGTCAAGCCAATCCCATAAAACCATTCTCAGTTCGGATTGTAGGCTGCAACTCGCCTACATGAAGCTGGAATCGCT
AGTAATCGCGGATCAGCATGCCGCGGTGAATACGTTCCCGGGCCTTGTACACACCGCCCGTCACACCACGAGAGTTTGTAACACCCGAAGTCGGTGGAGT
AACCGTAAGGAGCTAGCCGCCTAA

>EB65
GATTAGTGGCGAACGGGTGAGTAACACGTGAGTAACCTGCCCTTAACTCTGGGATAAGCCTGGGAAACTGGGTCTAATACCGGATATGACTCCTCATCGC
ATGGTGGGGGGTGGAAAGCTTTATTGTGGTTTTGGATGGACTCGCGGCCTATCAGCTTGTTGGTGAGGTAATGGCTCACCAAGGCGACGACGGGTAGCCG
GCCTGAGAGGGTGACCGGCCACACTGGGACTGAGACACGGCCCAGACTCCTACGGGAGGCAGCAGTGGGGAATATTGCACAATGGGCGCAAGCCTGATGC
AGCGACGCCGCGTGAGGGATGACGGCCTTCGGGTTGTAAACCTCTTTCAGTAGGGAAGAAGCGAAAGTGACGGTACCTGCAGAAGAAGCGCCGGCTAACT
ACGTGCCAGCAGCCGCGGTAATACGTAGGGCGCAAGCGTTATCCGGAATTATTGGGCGTAAAGAGCTCGTAGGCGGTTTGTCGCGTCTGCCGTGAAAGTC
CGGGGCTCAACTCCGGATCTGCGGTGGGTACGGGCAGACTAGAGTGATGTAGGGGAGACTGGAATTCCTGGTGTAGCGGTGAAATGCGCAGATATCAGGA
GGAACACCGATGGCGAAGGCAGGTCTCTGGGCATTAACTGACGCTGAGGAGCGAAAGCATGGGGAGCGAACAGGATTAGATACCCTGGTAGTCCATGCCG
TAAACGTTGGGCACTAGGTGTGGGGGACATTCCACGTTTTCCGCGCCGTAGCTAACGCATTAAGTGCCCCGCCTGGGGAGTACGGCCGCAAGGCTAAAAC
TCAAAGGAATTGACGGGGGCCCGCACAAGCGGCGGAGCATGCGGATTAATTCGATGCAACGCGAAGAACCTTACCAAGGCTTGACATGGGCCGGACCGGG
CTGGAAACAGTCCTTCCCCTTTGGGGCCGGTTCACAGGTGGTGCATGGTTGTCGTCAGCTCGTGTCGTGAGATGTTGGGTTAAGTCCCGCAACGAGCGCA
ACCCTCGTTCCATGTTGCCAGCGCGTAATGGCGGGGACTCATGGGAGACTGCCGGGGTCAACTCGGAGGAAGGTGGGGACGACGTCAAATCATCATGCCC
CTTATGTCTTGGGCTTCACGCATGCTACAATGGCCGGTACAAAGGGTTGCGATACTGTGAGGTGGAGCTAATCCCAAAAAGCCGGTCTCAGTTCGGATTG
GGGTCTGCAACTCGACCCCATGAAGTCGGAGTCGCTAGTAATCGCAGATCAGCAACGCTGCGGTGAATACGTTCCCGGGCCTTGTACACACCGCCCGTCA
AGTCACGAAAGTTGGTAACACCCGAAGCCGGTGGCCTAACCCCTTGTGGGAGGGAG

>EB66
TGGCTCCATGAAGGTTACCTCACCGACTTCGGGTGTTACAAACTCTCGTGGTGTGACGGGCGGTGTGTACAAGGCCCGGGAACGTATTCACCGCGGCATG
CTGATCCGCGATTACTAGCGATTCCGGCTTCATGCAGGCGAGTTGCAGCCTGCAATCCGAACTGAGAATGGCTTTATGGGATTCGCTTACCTTCGCAGGT
TTGCAGCCCTTTGTACCATCCATTGTAGCACGTGTGTAGCCCAGGTCATAAGGGGCATGATGATTTGACGTCATCCCCACCTTCCTCCGGTTTGTCACCG
GCAGTCACCTTAGAGTGCCCAACTGAATGCTGGCAACTAAGATCAAGGGTTGCGCTCGTTGCGGGACTTAACCCAACATCTCACGACACGAGCTGACGAC
AACCATGCACCACCTGTCACTCTGTCCCCCGAAGGGGAAAGCCCTATCTCTAGGGTTGTCAGAGGATGTCAAGACCTGGTAAGGTTCTTCGCGTTGCTTC
GAATTAAACCACATGCTCCACCGCTTGTGCGGGCCCCCGTCAATTCCTTTGAGTTTCAGCCTTGCGGCCGTACTCCCCAGGCGGAGTGCTTAATGCGTTA
GCTGCAGCACTAAAGGGCGGAAACCCTCTAACACTTAGCACTCATCGTTTACGGCGTGGACTACCAGGGTATCTAATCCTGTTTGCTCCCCACGCTTTCG
CGCCTCAGTGTCAGTTACAGACCAGAAAGTCGCCTTCGCCACTGGTGTTCCTCCAAATCTCTACGCATTTCACCGCTACACTTGGAATTCCACTTTCCTC
TTCTGCACTCAAGTCCCCCAGTTTCCAATGACCCTCCACGGTTGAGCCGTGGGCTTTCACATCAGACTTAAGGAACCACCTGCGCGCGCTTTACGCCCAA
TAATTCCGGACAACGCTTGCCACCTACGTATTACCGCGGCTGCTGGCACGTAGTTAGCCGTGGCTTTCTGGTTAGGTACCGTCAAGGTACCAGCAGTTAC
TCTGGTACTTGTTCTTCCCTAACAACAGAACTTTACGACCCGAAGGCCTTCTTCGTTCACGCGGCGTTGCTCCGTCAGACTTTCGTCCATTGCGGAAGAT

TCCCTACTGCTGCCTCCCGTAGGAGTCTGGGCCGTGTCTCAGTCCCAGTGTGGCCGATCACCCTCTCAGGTCGGCTACGCATCGTCGCCTTGGTGAGCCA
TTACCTCACCAACTAGCTAATGCGCCGCGGGCCCATCTATAAGTGACAGCGTAAACCGTCTTTCCATCTTCTCTCATGCGAGAAAAGAACGTATCCGGTA
TTAGCTCCGGTTTCCCGAAGTTATCCCAGTCTTATAGGCAGGTTGCCCACGTGTTACTCACCCGTCCGCCGCTAATCTCAGGGAGCAAGCTCCCGTCGAT
TCGCTCGACTGC

>EB67
TGGCTCCTTGCGGTTACCCCACCGACTTCGGGTGTTGCAAACTCTCGTGGTGTGACGGGCGGTGTGTACAAGGCCCGGGAACGTATTCACCGCGGCATGC
TGATCCGCGATTACTAGCGATTCCAGCTTCATGCAGGCGAGTTGCAGCCTGCAATCCGAACTGAGAATGGTTTTATGGGATTGGCTAAACCTCGCGGTCT
CGCAGCCCTTTGTACCATCCATTGTAGCACGTGTGTAGCCCAGGTCATAAGGGGCATGATGATTTGACGTCATCCCCACCTTCCTCCGGTTTGTCACCGG
CAGTCACCTTAGAGTGCCCAACTGAATGCTGGCAACTAAGATCAAGGGTTGCGCTCGTTGCGGGACTTAACCCAACATCTCACGACACGAGCTGACGACA
ACCCATGCACCACCTGTCACTTTGTCCCCCGAAGGGGAACCTTCTATCTCTAGAAGTGGCAAAGGATGTCAAGACCTGGTAAGGTTCTTCGCGTTGCTTC
GAATTAAACCACATGCTCCACCGCTTGTGCGGGCCCCCGTCAATTCCTTTGAGTTTCAGTCTTGCGACCGTACTCCCCAGGCGGAGTGCTTAATGCGTTA
GCTGCAGCACTAAAGGGCGGAAACCCTCTAACACTTAGCACTCATCGTTTACGGCGTGGACTACCAGGGTATCTAATCCTGTTCGCTCCCCACGCTTTCG
CGCCTCAGCGTCAGTTACAGACCAGAGAGTCGCCTTCGCCACTGGTGTTCCTCCACATCTCTACGCATTTCACCGCTACACGTGGAATTCCACTCTCCTC
TTCTGCACTCAAGTTTCCCAGTTTCCAATGACCCTCCCCGGTTGAGCCGGGGGCTTTCACATCAGACTTAAGAAACCGCCTGCGCGCGCTTTACGCCCAA
TAATTCCGGACAACGCTTGCCACCTACGTATTACCGCGGCTGCTGGCACGTAGTTAGCCGTGGCTTTCTGGTTAGGTACCGTCAAGGTGCGAGCAGTTAC
TCTCGCACTTGTTCTTCCCTAACAACAGAGTTTTACGATCCGAAAACCTTCATCACTCACGCGGCGTTGCTCCGTCAGACTTTCGTCCATTGCGGAAGAT
TCCCTACTGCTGCCTCCCGTAGGAGTCTGGGCCGTGTCTCAGTCCCAGTGTGGCCGATCACCCTCTCAGGTCGGCTACGCATCGTTGCCTTGGTGAGCCA
TTACCTCACCAACTAGCTAATGCGCCGCGGGCCCATCTGTAAGTGACAGCCGAAACCGTCTTTCCAACTTGAACCATGCGGTTCAAGATACTATCCGGTA
TTAGCTCCGGTTTCCCGGAGTTATCCCAGTCTTACAGGCAGGTTGCCCACGTGTTACTCACCCGTCCGCCGCTAACCTGCTGGAGCAAGCTCCAGCAAGT
CCGCTCGACTGCA

>EB68
TGCAAGTCGAGCGGACGGATGGGAGCTTGCTCCCTGAAGTCAGCGGCGGACGGGTGAGTAACACGTGGGCAACCTGCCTGTAAGACTGGGATAACTTCGG
GAAACCGGAGCTAATACCGGATAATGCACAGCCTCTCATGAGGCTATGCTGAAAGATGGTTTCGGCTATCACTTACAGATGGGCCCGCGGCGCATTAGCT
AGTTGGTGAGGTAACGGCTCACCAAGGCAACGATGCGTAGCCGACCTGAGAGGGTGATCGGCCACACTGGGACTGAGACACGGCCCAGACTCCTACGGGA
GGCAGCAGTAGGGAATCTTCCGCAATGGACGAAAGTCTGACGGAGCAACGCCGCGTGAGTGATGAAGGTTTTCGGATCGTAAAACTCTGTTGTCAGGGAA
GAACAAGTGCCGGAGTAACTGCCGGCACCTTGACGGTACCTGACCAGAAAGCCACGGCTAACTACGTGCCAGCAGCCGCGGTAATACGTAGGTGGCAAGC
GTTGTCCGGAATTATTGGGCGTAAAGCGCGCGCAGGCGGTCTCTTAAGTCTGATGTGAAAGCCCACGGCTCAACCGTGGAGGGTCATTGGAAACTGGGG
ACTTGAGTGCAGAAGAGGAAAGTGGAATTCCACGTGTAGCGGTGAAATGCGTAGAGATGTGGAGGAACACCAGTGGCGAAGGCGACTTTCTGGTCTGTAA
CTGACGCTGAGGCGCGAAAGCGTGGGGAGCAAACAGGATTAGATACCCTGGTAGTCCACGCCGTAAACGATGAGTGCTAAGTGTTAGAGGGTTTCCGCCC
TTTAGTGCTGCAGCAAACGCATTAAGCACTCCGCCTGGGGAGTACGGCCGCAAGGCTGAAACTCAAAGGAATTGACGGGGGCCCGCACAAGCGGTGGAGC
ATGTGGTTTAATTCGAAGCAACGCGAAGAACCTTACCAGGTCTTGACATCTCCTGACAACCCTAGAGATAGGGCGTTCCCCTTCGGGGGACAGGATGACA
GGTGGTGCATGGTTGTCGTCAGCTCGTGTCGTGAGATGTTGGGTTAAGTCCCGCAACGAGCGCAACCCTTGATCTTAGTTGCCAGCATTCGGTTGGGCAC
TCTAAGGTGACTGCCGGTGACAAACCGGAGGAAGGTGGGGATGACGTCAAATCATCATGCCCCTTATGACCTGGGCTACACACGTGCTACAATGGATGGT
ACAAAGGGCTGCAAGACCGCGAGGTTAAGCGAATCCCATAAAACCATTCTCAGTTCGGATTGCAGGCTGCAACTCGCCTGCATGAAGCTGGAATCGCTAG
TAATCGCGGATCAGCATGCCGCGGTGAATACGTTCCCGGGCCTTGTACACACCGCCCGTCACACCACGAGAGTTTGTAACACCCGAAGTCGGTGGGGTAA
CCTTTGGAGCCAGCCG

>EB69
ATGCTCAGGATGAACGCTGGCGGCGTGCTTAACACATGCAAGTCGAACGATGATCCGCAGCTTGCTGCGGGGATTAGTGGCGAACGGGTGAGTAACACGT
GAGTAACCTGCCCTTAACTCTGGGATAAGCCTGGGAAACTGGGTCTAATACCGGATATGACTCCTCATCGCATGGTGGGGGGTGGAAAGCTTTTTGTGGT
TTTGGATGGACTCGCGGCCTATCAGCTTGTTGGTGAGGTAATGGCTCACCAAGGCGACGACGGGTAGCCGGCCTGAGAGGGTGACCGGCCACACTGGGAC
TGAGACACGGCCCAGACTCCTACGGGAGGCAGCAGTGGGGAATATTGCACAATGGGCGCAAGCCTGATGCAGCGACGCCGCGTGAGGGATGACGGCCTTC
GGGTTGTAAACCTCTTTCAGTAGGGAAGAAGCGAAAGTGACGGTACCTGCAGAAGAAGCGCCGGCTAACTACGTGCCAGCAGCCGCGGTAATACGTAGGG
CGCAAGCGTTATCCGGAATTATTGGGCGTAAAGAGCTCGTAGGCGGTTTGTCGCGTCTGCCGTGAAAGTCCGGGGCTCAACTCCGGATCTGCGGTGGGTA
CGGGCAGACTAGAGTGATGTAGGGGAGACTGGAATTCCTGGTGTAGCGGTGAAATGCGCAGATATCAGGAGGAACACCGATGGCGAAGGCAGGTCTCTGG
GCATTAACTGACGCTGAGGAGCGAAAGCATGGGGAGCGAACAGGATTAGATACCCTGGTAGTCCATGCCGTAAACGTTGGGCACTAGGTGTGGGGGACAT
TCCACGTTTTCCGCGCCGTAGCTAACGCATTAAGTGCCCCGCCTGGGGAGTACGGCCGCAAGGCTAAAACTCAAAGGAATTGACGGGGGCCCGCACAAGC
GGCGGAGCATGCGGATTAATTCGATGCAACGCGAAGAACCTTACCAAGGCTTGACATGGACCGGGCTGGAAACAGTCCTTCCCCTTTGGGGCCGG
TTCACAGGTGGTGCATGGTTGTCGTCAGCTCGTGTCGTGAGATGTTGGGTTAAGTCCCGCAACGAGCGCAACCCTCGTTCCATGTTGCCAGCGCGTAATG
GCGGGGACTCATGGGAGACTGCCGGGGTCAACTCGGAGGAAGGTGGGGACGACGTCAAATCATCATGCCCCTTATGTCTTGGGCTTCACGCATGCTACAA
TGGCCGGTACAAAGGGTTGCGATACTGTGAGGTGGAGCTAATCCCAAAAAGCCGGTCTCAGTTCGGATTGGGGTCTGCAACTCGACCCCATGAAGTCGGA
GTCGCTAGTAATCGCAGATCAGCAACGCTGCGGTGAATACGTTCCCGGGCCTTGTACACACCGCCCGTCAAGTCACGAAAGTTGGTAACACCCGAAGCCG
GTGGCCTAACCCCTTGTGGGAAAGGAACTGTCCAAAGGTGGGACTGGCGATTGGGACTAGTCGAAA

>EB70
GCCAACTAAGATTCAAGGGGTTGCGCTTCGTTGCGGGGACTTAACCCCCAACATTTTCAGGGACACGAGGTGGACGACAACCCATGCCACCACCTGTCAC
TTTGTCCCCCGAAGGGGGAACGCCCTATCTCTAGGGTTGTCAGAGGATGTCAAGACCTGGTAAGGTTCTTTCGCGTTGCTTCGAATTAAACCACATGCT
CCCACCGCTTGTGCGGGCCCCCCGTCAATTCCTTTGAGTTTCAGCCTTGCGGCCGTACTCCCCAGGCGGAGTGCTTAATGCGTTTGCTGCAGCACTAAAG
GGCGGAAACCCTCTAACACTTAGCACTCATCGTTTACGGCGTGGACTACCAGGGTATCTAATCCTGTTTGCTCCCCACGCTTTCGCGCCTCAGTGTCAGT
TACAGGCCAAAGAGTCGCCTTCGCCACTGGTGTTCCTCCACATCTCTACGCATTTCACCGCTACACGTGGAATTCCACTCTTCTCTCCTGCACTCAAGTC
TCCCAGTTTCCAATGACCCTCCCCGGTTGAGCCGGGGGCTTTCACATCAGACTTAAGAGACCACCTGCGCGCGCTTTACGCCCAATAATTCCGGACAACG
CTTGCCACCTACGTATTACCGCGGCTGCTGGCACGTAGTTAGCCGTGGCTTTCTGGTTAGGTACCGTCAAGGTACCGGCAGTTACTCCGGTACTTGTTCT
TCCCTAACAACAGAGTTTTACGATCCGAAAACCTTCATCACTCACGCGGCGTTGCTCCGTCAGACTTTCGTCCATTGCGGAAGATTCCCTACTGCTGCCT
CCCGTAGGAGTCTGGGCCGTGTCTCAGTCCCAGTGTGGCCGATCACCCTCTCAGGTCGGCTACGCATCGTCGCCTTGGTGAGCCGTTACCTCACCAACTA
GCTAATGCGCCGCGGGCCCATCTATGAGTGATAGCCGAAACCATCTTTCAACTTCTCTACATGAGTAGAAAAGAATTATCCGGTATTAGCCCCGGTTTCC
CGGAGTTATCCCAGTCTCATAGGCAGGTTGCCCACGTGTTACTCACCCGTCCGCCGCT

>EB71
TCGAGCGAACTGATTAGAAGCTTGCTTCTATGACGTTAGCGGCGGACGGGTGAGTAACACGTGGGCAACCTGCCTGTAAGACTGGGATAACTTCGGGAAA
CCGAAGCTAATACCGGATAGGATCTTCTCCTTCATGGGAGATGATTGAAAGATGGTTTCGGCTATCACTTACAGATGGGCCCGCGGTGCATTAGCTAGTT
GGTGAGGTAACGGCTCACCAAGGCAACGATGCATAGCCGACCTGAGAGGGTGATCGGCCACACTGGGACTGAGACACGGCCCAGACTCCTACGGGAGGCA
GCAGTAGGGAATCTTCCGCAATGGACGAAAGTCTGACGGAGCAACGCCGCGTGAGTGATGAAGGCTTTCGGGTCGTAAAACTCTGTTGTTAGGGAAGAAC
AAGTACAAGAGTAACTGCTTGTACCTTGACGGTACCTAACCAGAAAGCCCACGGCTAACTACGTGCCAGCAGCCGCGGTAATACGTAGGTGGCAAGCG
TTATCCGGAATTATTGGGCGTAAAGCGCGCGCAGGCGGTTTCTTAAGTCTGATGTGAAAGCCCACGGCTCAACCGTGGAGGGTCATTGGAAACTGGGGA
ACTTGAGTGCAGAAGAGAAAAGCGGAATTCCACGTGTAGCGGTGAAATGCGTAGAGATGTGGAGGAACACCAGTGGCGAAGGCGGCTTTTTGGTCTGTAA
CTGACGCTGAGGCGCGAAAGCGTGGGGAGCAAACAGGATTAGATACCCTGGTAGTCCACGCCGTAAACGATGAGTGCTAAGTGTTAGAGGGTTTCCGCCC

TTTAGTGCTGCAGCTAACGCATTAAGCACTCCGCCTGGGGAGTACGGTCGCAAGACTGAAACTCAAAGGGAATTGACGGGGGCCCGCACAAGCGGTGGAG
CATGTGGTTTAATTCGAAGCAACGCGAAGAACCTTACCAGGTCTTGACATCCTCTGACAACTCTAGAGATAGAGCGTTCCCCTTCGGGGGACAGAGTGAC
AGGTGGTGCATGGTTGTCGTCAGCTCGTGTCGTGAGATGTTGGGTTAAGTCCCGCAACGAGCGCAACCCTTGATCTTAGTTGCCAGCATTTAGTTGGGCA
CTCTAAGGTGACTGCCGGTGACAAACCGGAGGAAGGTGGGGATGACGTCAAATCATCATGCCCCTTATGACCTGGGCTACACACGTGCTACAATGGATGG
TACAAAGGGCTGCAAGACCGCGAGGTCAAGCCAATCCCATAAAACCATTCTCAGTTCGGATTGTAGGCTGCAACTCGCCTACATGAAGCTGGAATCGCTA
GTAATCGCGGATCAGCATGCCGCGGTGAATACGTTCCCGGGCCTTGTACACACCGCCCGTCACACCACGAGAGTTTGTAACACCCGAAGTCGGTGGAGTA
ACCGTAAGGAGCTAGCCGCCT

>EB72
TGCAAGTCGAGCGGACAGATGGGAGCTTGCTCCCTGATGTTAGCGGCGGACGGGTGAGTAACACGTGGGTAACCTGCCTGTAAGACTGGGATAACTCCGG
GAAACCGGGGCTAATACCGGATGGTTGTCTGAACCGCATGGTTCAGACATAAAAGGTGGCTTCGGCTACCACTTACAGATGGACCCGCGGCGCATTAGCT
AGTTGGTGAGGTAACGGCTCACCAAGGCGACGATGCGTAGCCGACCTGAGAGGGTGATCGGCCACACTGGGACTGAGACACGGCCCAGACTCCTACGGGA
GGCAGCAGTAGGGAATCTTCCGCAATGGACGAAAGTCTGACGGAGCAACGCCGCGTGAGTGATGAAGGTTTTCGGATCGTAAAGCTCTGTTGTTAGGGAA
GAACAAGTGCCGTTCAAATAGGGCGGCACCTTGACGGTACCTAACCCAGAAAGCCACGGCTAACTACGTGCCAGCAGCCGCGGTAATACGTAGGTGGCAA
GCGTTGTCCGGAATTATTGGGCGTAAAGGGCTCGCAGGCGGTTTTCTTAAGTCTGATGTGAAAGCCCCCGGCTCAACCGGGGAGGGTCATTGGAAACTGG
GGAACTTGAGTGCAGAAGAGGAGAGTGGAATTCCACGTGTAGCGGTGAAATGCGTAGAGATGTGGAGGAACACCAGTGGCGAAGGCGACTCTCTGGTCTG
TAACTGACGCTGAGGAGCGAAAGCGTGGGGAGCGAACAGGATTAGATACCCTGGTAGTCCACGCCGTAAACGATGAGTGCTAAGTGTTAGGGGGTTTCCG
CCCCTTAGTGCTGCAGCTAACGCATTAAGCACTCCGCCTGGGGAGTACGGTCGCAAGACTGAAACTCAAAGGAATTGACGGGGGCCCGCACAAGCGGTGG
AGCATGTGGTTTAATTCGAAGCAACGCGAAGAACCTTACCAGGTCTTGACATCCTCTGACAATCCTAGAGATAGGACGTCCCCTTCGGGGGCAGAGTGAC
AGGTGGTGCATGGTTGTCGTCAGCTCGTGTCGTGAGATGTTGGGTTAAGTCCCGCAACGAGCGCAACCCTTGATCTTAGTTGCCAGCATTCAGTTGGGCA
CTCTAAGGTGACTGCCGGTGACAAACCGGAGGAAGGTGGGGATGACGTCAAATCATCATGCCCCTTATGACCTGGGCTACACACGTGCTACAATGGACAG
AACAAAGGGCAGCGAAACCGCGAGGTTAAGCCAATCCCACAAATCTGTTCTCAGTTCGGATCGCAGTCTGCAACTCGACTGCGTGAAGCTGGAATCGCTA
GTAATCGCGGATCAGCATGCCGCGGTGAATACGTTCCCGGGCCTTGTACACACCGCCCGTCACACCACGAGAGTTTGTAACACCCGAAGTCGGTGAGGTA
ACCTTTAGGAGCCAGCCG

>EB73
GATCATGGCTCAGTAAGTCGTAACAAGGTAACCGTAGAGTTTGATTATGGCTCAGGAAGTCGCAACGAGGTATCTGCCCCTGACTCTGGGATAAGCGCTG
GAAACGGCGTCTAATACTGGATACGAGCTGCGAAGGCATCTTCAGCAGCTGGAAAGAATTTCGGTCAGGGATGAGCTCGCGGCCTATCAGCTTGTTGGTG
AGGTAATGGCTCACCAAGGCGTCGACGGGTAGCCGGCCTGAGAGGGTGACCGGCCACACTGGGACTGAGACACGGCCCAGACTCCTACGGGAGGCAGCAG
TGGGGAATATTGCACAATGGGCGAAAGCCTGATGCAGCAACGCCGCGTGAGGGATGACGGCCTTCGGGTTGTAAACCTCTTTTAGCAGGGAAGAAGCGAA
AGTGACGGTACCTGCAGAAAAAGCGCCGGCTAACTACGTGCCAGCAGCCGCGGTAATACGTAGGGCGCAAGCGTTATCCGGAATTATTGGGCGTAAAGAG
CTCGTAGGCGGTTTGTCGCGTCTGCTGTGAAATCCCGAGGCTCAACCTCGGGCCTGCAGTGGGTACGGGCAGACTAGAGTGCGGTAGAGGAGATTGGAAT
TCCTGGTGTAGCGGTGGAATGCGCAGATATCAGGAGGAACACCGATGGCGAAGGCAGATCTCTGGGCCGTAACTGACGCTGAGGAGCGAAAGGGTGGGGA
GCAAACAGGCTTAGATACCCTGGTAGTCCACCCCGTAAACGTTGGGAACTAGTTGTGGGGTCCATTCCACGGATTCCGTGACGCAGCTAACGCATTAAGT
TCCCCGCCTGGGGAGTACGGCCGCAAGGCTAAAACTCAAAGGAATTGACGGGGACCCGCACAAGCGGCGGAGCATGCGGATTAATTCGATGCAACGCGAA
GAACCTTACCAAGGCTTGACATATACGAGAACGGGCCAGAAATGGTCAACTCTTTGGACACTCGTAAACAGGTGGTGCATGGTTGTCGTCAGCTCGTGTC
GTGAGATGTTGGGTTAAGTCCCGCAACGAGCGCAACCCTCGTTCTATGTTGCCAGCACGTCATGGTGGGAACTCATGGAATACTGCCGGGGTCAACTCGG
AGGAAGGTGAGGATGACGTCAAATCATCATGCCCCTTATGTCTTGGGCTTCACGCATGCTACAATGGCCGGTACAATGGGCTGCAATACCGCAAGGTGGA
GCGAATCCCAAAAAGCCGGTCCCAGTTCTGATTGAGGTCTGCAACTCGACCTCATGAAGTCGGAGTCGCTTGTAATCGCAGATCAGCACCGTTGCGGGGA
ACACGTTCCCGGTAGAGTTTGACCACGGCTCACTAAGTCGTAACAAGGTAACCGTAGAGTTGATCAGGCTCAGT

>EB74
CTCAGGATGAACGCTGGCGGCGTGCTTAACACATGCAAGTCGAACGATGATCCCAGCTTGCTGGGGGATTAGTGGCGAACGGGTGAGTAACACGTGAGTA
ACCTGCCCTTGACTCTGGGATAAGCCTGGGAAACTGGGTCTAATACCGGATACGACCATTCCACGCATGTGGTGGTGGTGGAAAGCTTTTGTGGTTTTGG
ATGGACTCGCGGCCTATCAGCTTGTTGGTGGGGTAATGGCCTACCAAGGCGACGACGGGTAGCCGGCCTGAGAGGGTGACCGGCCACACTGGGACTGAGA
CACGGCCCAGACTCCTACGGGAGGCAGCAGTGGGGAATATTGCACAATGGGCGCAAGCCTGATGCAGCGACGCCGCGTGAGGGATGACGGCCTTCGGGTT
GTAAACCTCTTTCAGTAGGGAAGAAGCGTAAGTGACGGTACCTGCAGAAGAAGCGCCGGCTAACTACGTGCCAGCAGCCGCGGTAATACGTAGGGCGCAA
GCGTTATCCGGAATTATTGGGCGTAAAGAGCTCGTAGGCGGTTTGTCGCGTCTGCTGTGAAAGACCGGGGCTCAACTCCGGTTCTGCAGTGGGTACGGGC
AGACTAGAGTGCAGTAGGGGAGACTGGAATTCCTGGTGTAGCGGTGAAATGCGCAGATATCAGGAGGAACACCGATGGCGAAGGCAGGTCTCTGGGCTGT
AACTGACGCTGAGGAGCGAAAGCATGGGGAGCGAACAGGATTAGATACCCTGGTAGTCCATGCCGTAAACGTTGGGCACTAGGTGTGGGGGACATTCCAC
GTTTTCCGCGCCGTAGCTAACGCATTAAGTGCCCCGCCTGGGGAGTACGGCCGCAAGGCTAAAACTCAAAGGAATTGACGGGGGCCCGCACAAGCGGCGG
AGCATGCGGATTAATTCGATGCAACGCGAAGAACCTTACCAAGGCTTGACATGAACCGGAAAGACCTGGAAACAGGTGCCCCGCTTGCGGTCGGTTTACA
GGTGGTGCATGGTTGTCGTCAGCTCGTGTCGTGAGATGTTGGGTTAAGTCCCGCAACGAGCGCAACCCTCGTTCTATGTTGCCAGCGCGTGATGGCGGGG
ACTCATAGGAGACTGCCGGGGTCAACTCGGAGGAAGGTGGGGACGACGTCAAATCATCATGCCCCTTATGTCTTGGGCTTCACGCATGCTACAATGGCCG
GTACAAAGGGTTGCGATACTGTGAGGTGGAGCTAATCCCAAAAAGCCGGTCTCAGTTCGGATTGGGGTCTGCAACTCGACCCCATGAAGTCGGAGTCGCT
AGTAATCGCAGATCAGCAACGCTGCGGTGAATACGTTCCCGGGCCTTGTACACACCGCCCGTCAAGTCACGAAAGTTGGTAACACCCGAAGCCGGTGGCC
TAACCCTTGTGGGGGGAGCCGTCCAAGGTGGGACCGGCGATTGGGACTAGTCGAAAAAAGGGGGA

>EB75
TGCAAGTCGAGCGGACCTCTTCGGAGGTTAGCGGCGGACGGGTGAGTAACACGTGGGCAACCTGCCTGTAAGACTGGGATAACTCCGGGAAACCGGAGCT
AATACCGGATAGTATCTTGAACCGCATGGTTCAAGCTGGAAAGACGGTTTCGGCTGTCACTTACAGATGGGCCCGCGGCGCATTAGCTAGTTGGTGAGGT
AATGGCTCACCAAGGCAACGATGCGTAGCCGACCTGAGAGGGTGATCGGCCACACTGGGACTGAGACACGGCCCAGACTCCTACGGGAGGCAGCAGTAGG
GAATCTTCCGCAATGGACGAAAGTCTGACGGAGCAACGCCGCGTGAGTGATGAAGGTTTTCGGATCGTAAAACTCTGTTGTTAGGGAAGAACAAGTGCGA
GAGTAACTGCTCGCACCTTGACGGTACCTAACCAGAAAGCCACGGCTAACTACGTGCCAGCAGCCGCGGTAATACGTAGGTGGCAAGCGTTGTCCGGAAT
TATTGGGCGTAAAGCGCGCGCAGGCGGTTTCTTAAGTCTGATGTGAAAGCCCCCGGCTCAACCGGGGAGGGTCATTGGAAACTGGGAAACTTGAGTGCAG
AAGAGGAGAGTGGAATTCCACGTGTAGCGGTGAAATGCGTAGAGATGTGGAGGAACACCAGTGGCGAAGGCGACTCTCTGGTCTGTAACTGACGCTGAGG
CGCGAAAGCGTGGGGAGCGAACAGGATTAGATACCCTGGTAGTCCACGCCGTAAACGATGAGTGCTAAGTGTTAGAGGGTTTCCGCCCTTTAGTGCTGCA
GCTAACGCATTAAGCACTCCGCCTGGGGAGTACGGTCGCAAGACTGAAACTCAAAGGAATTGACGGGGGCCCGCACAAGCGGTGGAGCATGTGGTTTAAT
TCGAAGCAACGCGAAGAACCTTACCAGGTCTTGACATCCTTTGCCACTTCTAGAGATAGAAGGTTCCCCTTCGGGGGACAAAGTGACAGGTGGTGCATGG
TTGTCGTCAGCTCGTGTCGTGAGATGTTGGGTTAAGTCCCGCAACGAGCGCAACCCTTGATCTTAGTTGCCAGCATTCAGTTGGGCACTCTAAGGTGACT
GCCGGTGACAAACCGGAGGAAGGTGGGGATGACGTCAAATCATCATGCCCCTTATGACCTGGGCTACACACGTGCTACAATGGATGGTACAAAGGGCTGC
GAGACCGCGAGGTTTAGCCAATCCCATAAAACCATTCTCAGTTCGGATTGCAGGCTGCAACTCGCCTGCATGAAGCTGGAATCGCTAGTAATCGCGGATC
AGCATGCCGCGGTGAATACGTTCCCGGGCCTTGTACACACCGCCCGTCACACCACGAGAGTTTGCAACACCCGAAGTCGGTGGGGTAACCGCAAGGAGCC
AGCCGCCT

>EB76

GTCGAGCGAATCGATGGGAGCTTGCTCCCTGAGATTAGCGGCGGACGGGTGAGTAACACGTGGGCAACCTGCCTATAAGACTGGGATAACTTCGGGAAAC
CGGAGCTAATACCGGATACGTTCTTTTCTCGCATGAGAGAAGATGGAAAGACGGTTTACGCTGTCACTTATAGATGGGCCCGCGGCGCATTAGCTAGTTG
GTGAGGTAATGGCTCACCAAGGCGACGATGCGTAGCCGACCTGAGAGGGTGATCGGCCACACTGGGACTGAGACACGGCCCAGACTCCTACGGGAGGCAG
CAGTAGGGAATCTTCCGCAATGGACGAAAGTCTGACGGAGCAACGCCGCGTGAACGAAGAAGGCCTTCGGGTCGTAAAGTTCTGTTGTTAGGGAAGAACA
AGTACCAGAGTAACTGCTGGTACCTTGACGGTACCTAACCAGAAAGCCACGGCTAACTACGTGNCCAGCAGCCGCGGTAATACGTAGGTGGCAAGCGTTG
TCCGGAATTATTGGGCGTAAAGCGCGCGCAGGTGGTTCCTTAAGTCTGATGTGAAAGCCCACGGCTCAACCGTGGAGGGTCATTGGAAACTGGGGAACTT
GAGTGCAGAAGAGGAAAGTGGAATTCCAAGTGTAGCGGTGAAATGCGTAGAGATTTGGAGGAACACCAGTGGCGAAGGCGACTTTCTGGTCTGTAACTGA
CACTGAGGCGCGAAAGCGTGGGGAGCAAACAGGATTAGATACCCTGGTAGTCCACGCCGTAAACGATGAGTGCTAAGTGTTAGAGGGTTTCCGCCCTTTA
GTGCTGCAGCTAACGCATTAAGCACTCCGCCTGGGGAGTACGGCCGCAAGGCTGAAACTCAAAGGAATTGACGGGGGCCCGCACAAGCGGTGGAGCATGT
GGTTTAATTCGAAGCAACGCGAAGAACCTTACCAGGTCTTGACATCCTCTGACAACCCTAGAGATAGGGCTTTCCCCTTCGGGGGACAGAGTGACAGGTG
GTGCATGGTTGTCGTCAGCTCGTGTCGTGAGATGTTGGGTTAAGTCCCGCAACGAGCGCAACCCTTGATCTTAGTTGCCAGCATTCAGTTGGGCACTCTA
AGGTGACTGCCGGTGACAAACCGGAGGAAGGTGGGGATGACGTCAAATCATCATGCCCCTTATGACCTGGGCTACACACGTGCTACAATGGATGGTACAA
AGGGCTGCAAACCTGCGAAGGTAAGCGAATCCCATAAAGCCATTCTCAGTTCGGATTGCAGGCTGCAACTCGCCTGCATGAAGCCGGAATCGCTAGTAAT
CGCGGATCAGCATGCCGCGGTGAATACGTTCCCGGGCCTTGTACACACCGCCCGTCACACCACGAGAGTTTGTAACACCCGAAGTCGGTGAGGTAACCTT
CATGGAGCCAGCCGCCT

>EB77
TCGAGCGGACAGAAGGGAGCTTGCTCCCGGATGTTAGCGGCGGACGGGTGAGTAACACGTGGGTAACCTGCCTGTAAGACTGGGATAACTCCGGGAAACC
GGAGCTAATACCGGATAGTTCCTTGAACCGCATGGTTCAAGGATGAAAGACGGTTTCGGCTGTCACTTACAGATGGACCCGCGGCGCATTAGCTAGTTGG
TGAGGTAACGGCTCACCAAGGCGACGATGCGTAGCCGACCTGAGAGGGTGATCGGCCACACTGGGACTGAGACACGGCCCAGACTCCTACGGGAGGCAGC
AGTAGGGAATCTTCCGCAATGGACGAAAGTCTGACGGAGCAACGCCGCGTGAGTGATGAAGGTTTTCGGATCGTAAAGCTCTGTTGTTAGGGAAGAACAA
GTGCAAGAGTAACTGCTTGCACCTTGACGGTACCTAACCAGAAAGCCACGGCTAACTACGTGCCAGCAGCCGCGGTAATACGTAGGTGGCAAGCGTTGTC
CGGAATTATTGGGCGTAAAGGGCTCGCAGGCGGTTTCTTAAGTCTGATGTGAAAGCCCCCGGCTCAACCGGGGAGGGTCATTGGAAACTGGGAAACTTGA
GTGCAGAAGAGGAGAGTGGAATTCCACGTGTAGCGGTGAAATGCGTAGAGATGTGGAGGAACACCAGTGGCGAAGGCGACTCTCTGGTCTGTAACTGACG
CTGAGGAGCGAAAGCGTGGGGAGCGAACAGGATTAGATACCCTGGTAGTCCACGCCGTAAACGATGAGTGCTAAGTGTTAGGGGGTTTCCGCCCCTTAGT
GCTGCAGCTAACGCATTAAGCACTCCGCCTGGGGAGTACGGTCGCAAGACTGAAACTCAAAGGAATTGACGGGGGCCCGCACAAGCGGTGGAGCATGTGG
TTTAATTCGAAGCAACGCGAAGAACCTTACCAGGTCTTGACATCCTCTGACAACCCTAGAGATAGGGCTTTCCCTTCGGGGACAGAGTGACAGGTGGTGC
ATGGTTGTCGTCAGCTCGTGTCGTGAGATGTTGGGTTAAGTCCCGCAACGAGCGCAACCCTTGATCTTAGTTGCCAGCATTCAGTTGGGCACTCTAAGGT
GACTGCCGGTGACAAACCGGAGGAAGGTGGGGATGACGTCAAATCATCATGCCCCTTATGACCTGGGCTACACACGTGCTACAATGGACAGAACAAAGGG
CTGCGAGACCGCAAGGTTTAGCCAATCCCACAAATCTGTTCTCAGTTCGGATCGCAGTCTGCAACTCGACTGCGTGAAGCTGGAATCGCTAGTAATCGCG
GATCAGCATGCCGCGGTGAATACGTTCCCGGGCCTTGTACACACCGCCCGTCACACCACGAGAGTTTGCAACACCCGAAGTCGGTGAGGTAACCTTTATG
GAGCCAG

>EB78
GTCGAGCGAATCGATGGGAGCTTGCTCCCTGAGATTAGCGGCGGACGGGTGAGTAACACGTGGGCAACCTGCCTATAAGACTGGGATAACTTCGGGAAAC
CGGAGCTAATACCGGATACGTTCTTTTCTCGCATGAGAGAAGATGGAAAGACGGTTTACGCTGTCACTTATAGATGGGCCCGCGGCGCATTAGCTAGTTG
GTGAGGTAATGGCTCACCAAGGCGACGATGCGTAGCCGACCTGAGAGGGTGATCGGCCACACTGGGACTGAGACACGGCCCAGACTCCTACGGGAGGCAG
CAGTAGGGAATCTTCCGCAATGGACGAAAGTCTGACGGAGCAACGCCGCGTGAACGAAGAAGGCCTTCGGGTCGTAAAGTTCTGTTGTTAGGGAAGAACA
AGTACCAGAGTAACTGCTGGTACCTTGACGGTACCTAACCAGAAAGCCACGGCTAACTACGTGCCAGCAGCCGCGGTAATACGTAGGTGGCAAGCGTTGT
CCGGAATTATTGGGCGTAAAGCGCGCGCAGGTGGTTCCTTAAGTCTGATGTGAAAGCCCACGGCTCAACCGTGGAGGGTCATTGGAAACTGGGGAACTTG
AGTGCAGAAGAGGAAAGTGGAATTCCAAGTGTAGCGGTGAAATGCGTAGAGATTTGGAGGAACACCAGTGGCGAAGGCGACTTTCTGGTCTGTAACTGAC
ACTGAGGCGCGAAAGCGTGGGGAGCAAACAGGATTAGATACCCTGGTAGTCCACGCCGTAAACGATGAGTGCTAAGTGTTAGAGGGTTTCCGCCCTTTAG
TGCTGCAGCTAACGCATTAAGCACTCCGCCTGGGGAGTACGGCCGCAAGGCTGAAACTCAAAGGAATTGACGGGGGCCCGCACAAGCGGTGGAGCATGTG
GTTTAATTCGAAGCAACGCGAAGAACCTTACCAGGTCTTGACATCCTCTGACAACCCTAGAGATAGGGCTTTCCCCTTCGGGGGACAGAGTGACAGGTGG
TGCATGGTTGTCGTCAGCTCGTGTCGTGAGATGTTGGGTTAAGTCCCGCAACGAGCGCAACCCTTGATCTTAGTTGCCAGCATTCAGTTGGGCACTCTAA
GGTGACTGCCGGTGACAAACCGGAGGAAGGTGGGGATGACGTCAAATCATCATGCCCCTTATGACCTGGGCTACACACGTGCTACAATGGATGGTACAAA
GGGCTGCAAACCTGCGAAGGTAAGCGAATCCCATAAAGCCATTCTCAGTTCGGATTGCAGGCTGCAACTCGCCTGCATGAAGCCGGAATCGCTAGTAATC
GCGGATCAGCATGCCGCGGTGAATACGTTCCCGGGCCTTGTACACACCGCCCGTCACACCACGAGAGTTTGTAACACCCGAAGTCGGTGAGGTAACCTTC
ATGGAGCCAGCCG

>EB79
GCAAGTCGAGCGAATCTGAGGGAGCTTGCTCCCAAAGATTAGCGGCGGACGGGTGAGTAACACGTGGGTAACCTGCCTGTAAGATTGGGATAACTCCGGG
AAACCGGAGCTAATACCGGATAACATTTCGAACCGCATGGTTCGAAATTGAAAGATGGTTTCGGCTATCACTTACAGATGGACCCGCGGCGCATTAGCTA
GTTGGTGAGGTAACGGCTCACCAAGGCAACGATGCGTAGCCGACCTGAGAGGGTGATCGGCCACACTGGGACTGAGACACGGCCCAGACTCCTACGGGAG
GCAGCAGTAGGGAATCTTCCGCAATGGACGAAAGTCTGACGGAGCAACGCCGCGTGAACGATGAAGGCCTTTCGGGTCGTAAAGTTCTGTTGTTAGGGAA
GAACAAGTACCAGAGTAACTGCTGGTACCTTGACGGTACCTAACCAGAAAGCCACGGCTAACTACGTGCCAGCAGCCGCGGTAATACGTAGGTGGCAAGC
GTTGTCCGGAATTATTGGGCGTAAAGCGCGCGCAGGCGGTTTCTTAAGTCTGATGTGAAAGCCCACGGCTCAACCGTGGAGGGTCATTGGAAACTGGGGA
ACTTGAGTGCAGAAGAGGAGAGTGGAATTCCACGTGTAGCGGTGAAATGCGTAGAGATGTGGAGGAACACCAGTGGCGAAGGCGACTCTCTGGTCTGTAA
CTGACGCTGAGGCGCGAAAGCGTGGGGAGCGAACAGGATTAGATACCCTGGTAGTCCACGCCGTAAACGATGAGTGCTAAGTGTTAGAGGGTTTCCGCCC
TTTAGTGCTGCAGCAAACGCATTAAGCACTCCGCCTGGGGAGTACGGTCGCAAGACTGAAACTCAAAGGAATTGACGGGGGCCCGCACAAGCGGTGGAGC
ATGTGGTTTAATTCGAAGCAACGCGAAGAACCTTACCAGGTCTTGACATCCTTCGCTACTTCTAGAGATAGAAGGTTCCCCTTCGGGGGACGAAGTGACA
GGTGGTGCATGGTTGTCGTCAGCTCGTGTCGTGAGATGTTGGGTTAAGTCCCGCAACGAGCGCAACCCTTGATCTTAGTTGCCAGCATTCAGTTGGGCAC
TCTAAGGTGACTGCCGGTGACAAACCGGAGGAAGGTGGGGATGACGTCAAATCATCATGCCCCTTATGACCTGGGCTACACACGTGCTACAATGGATGGT
ACAAAGGGCTGCAAGACTGCGAAGTCAAGCGAATCCCATAAAACCATTCTCAGTTCGGATTGCAGGCTGCAACTCGCCTGCATGAAGCCGGAATCGCTAG
TAATCGCGGATCAGCATGCCGCGGTGAATACGTTCCCGGCCCTTGTACACACCCCCCGTCACACCACGAGAGTTTGTAACACCCGAAGTCGGTGGGGTAA
CCGTAAGGAGCCAGCCGCCTAA

>EB80
CCACAAGGGTTGGGCCACCGGCTTCAGGTGTTACCGACTTTCATGACTTGACGGGCGGTGTGTACAAGACCCGGGAACGTATTCACCGCAGCGTTGCTGA
TCTGCGATTACTAGCGACTCCGACTTCATGAGGTCGAGTTGCAGACCTCAATCCGAACTGGGACCGGCTTTTTGGGATTCGCTCCACCTTACGGTATTGC
AGCCCTTTGTACCGGCCATTGTAGCATGCGTGAAGCCCAAGACATAAGGGGCATGATGATTTGACGTCATCCCCACCTTCCTCCGAGTTGACCCCGGCAG
TATCCCATGAGTTCCCACCATTACGTGCTGGCAACATAGAACGAGGGTTGCGCTCGTTGCGGGACTTAACCCAACATCTCACGACACGAGCTGACGACAA
CCATGCACCACCTGTTTACGAGTGTCCAAAGAGTTGACCATTTCTGGCCCGTTCTCGTATATGTCAAGCCTTGGTAAGGTTCTTCGCGTTGCATCGAATT
AATCCGCATGCTCCGCCGCTTGTGCGGGTCCCCGTCAATTCCTTTGAGTTTTAGCCTTGCGGCCGTACTCCCCAGGCGGGGAACTTAATGCGTTAGCTGC
GTCACGGAGACCGTGGAATGGTCCCCACAACTAGTTCCCAACGTTTACGGGGTGGACTACCAGGGTATCTAAGCCTGTTTGCTCCCCACCCTTTCGCTCC
TCAGCGTCAGTTACGGCCCAGAGATCTGCCTTCGCCATCGGTGTTCCTCCTGATATCTGCGCATTCCACCGCTACACCAGGAATTCCAATCTCCCCTACC
GCACTCTAGTCTGCCCGTACCCACTGCAGGCCCGAGGTTGAGCCTCGGGTTTTCACAGCAGACGCGACAAACCGCCTACGAGCTCTTTACGCCCAATAAT
TCCGGATAACGCTTGCGCCCTACGTATTACCGCGGCTGCTGGCACGTAGTTAGCCGGCGCTTTTTCTGCAGGTACCGTCACTTTTCGCTTCTTCCCTGCT

AAAAGAGGTTTACAACCCGAAGGCCGTCGTCCCTCACGCGGCGTTGCTGCATCAGGCTTTCGCCCATTGTGCAATATTCCCCACTGCTGCCTCCCGTAGG
AGTCTGGGCCGTGTCTCAGTCCCAGTGTGGCCGGTCACCCTCTCAGGCCGGCTACCCGTCGACGCCTTGGTGAGCCATTACCTCACCAACAAGCTGATAG
GCCGCGAGCTCATCCCTGACCGAAGTTCTTTCCAGCTGCTGAAGATGCCTTCGCAGCTCGTATCCGGTATTAGACGCCGTTTCCAGCGCTTATCCCAGAG
TCAGGGGCAGATTGCTCACGTGTTACTCACCCGTTCGCCACTGATCCAGCAGAGCAAGCTCCGCTTTCACCG

>EB81
CTCAGGACGAACGCTGGCGGCGTGCCTAATACATGCAAGTCGAGCGGACTAATGGGAGCTTGCTCCCGTTAGTTAGCGGCGGACGGGTGAGTAACACGTG
GGCAACCTACCTGTAAGACTGGGATAACTTCGGGAAACCGGAGCTAATACCGGATGACATAAAGGAACTCCTGTTCCTTTATTGAAAGATGGCTTCGGCT
ATCACTTACAGATGGGCCCGCGGCGCATTAGCTAGTTGGTGAGGTAACGGCTCACCAAGGCGACGATGCGTAGCCGACCTGAGAGGGTGATCGGCCACAC
TGGGACTGAGACACGGCCCAGACTCCTACGGGAGGCAGCAGTAGGGAATCTTCCGCAATGGACGAAAGTCTGACGGAGCAACGCCGCGTGAGCGATGAAG
GCCTTCGGGTCGTAAAGCTCTGTTGTTAGGGAAGAATAAGTGCTAGTTGAATAAGCTGGCACCTTGACGGTACCTAACCAGAAAGCCACGGCTAACTACG
TGCCAGCAGCCGCGGTAATACGTAGGTGGCAAGCGTTGTCCGGAATTATTGGGCGTAAAGCGCGCGCAGGCGGTTTCTTAAGTCTGATGTGAAAGCCCCC
GGCTCAACCGGGGAGGGTCATTGGAAACTGGGAAACTTGAGTGCAGAAGAGGAAAGTGGAATTCCAAGTGTAGCGGTGAAATGCGTAGAGATTTGGAGGA
ACACCAGTGGCGAAGGCGACTTTCTGGTCTGTAACTGACGCTGAGGCGCGAAAGCGTGGGGAGCAAACAGGATTAGATACCCTGGTAGTCCACGCTGTAA
ACGATGAGTGCTAAGTGTTAGAGGGTTTCCGCCCTTTAGTGCTGAAGTTAACGCATTAAGCACTCCGCCTGGGGAGTACGGTCGCAAGACTGAAACTCAA
AGGAATTGACGGGGGCCCGCACAAGTGGTGGAGCATGTGGTTTAATTCGAAGCAACGCGAAGAACCTTACCAGGTCTTGACATCCTCTGACAACCCTAGA
GATAGGGCTTTCCCTTCGGGGACAGAGTGACAGGTGGTGCATGGTTGTCGTCAGCTCGTGTCGTGAGATGTTGGGTTAAGTCCCGCAACGAGCGCAACCC
TTGATCTTAGTTGCCAGCATTTAGTTGGGCACTCTAAGGTGACTGCCGGTGACAAACCGGAGGAAGGTGGGGATGACGTCAAATCATCATGCCCCTTAT
GACCTGGGCTACACACGTGCTACAATGGATAGTACAAAGGGTTGCAAGACCGCGAGGTGGAGCTAATCCCATAAAACTATTCTCAGTTCGGATTGTAGGC
TGCAACTCGCCTACATGAAGCCGGAATCACTAGTAATCGCGGATCAGCATGCCGCGGTGAATACGTTCCCGGGCCTTGTACACACCGCCCGTCACACCAC
GAGAGTTTGTAACACCCGAAGTCGGTGGGGTAACCTTTTAGGGGCCAGCCGCCTAAAGTGGGACAGATAATTGGGGTGAATCTACAAA

>EB82
CAAGGGGTTAGGCCACCGGCTTCGGGTGTTACCGACTTTCGTGACTTGACGGGCGGTGTGTACAAGGCCCGGGAACGTATTCACCGCAGCGTTGCTGATC
TGCGATTACTAGCGACTCCGACTTCATGGGGTCGAGTTGCAGACCCCAATCCGAACTGAGACCGGCTTTTTGGGATTCGCTCCACCTCGCGGTATCGCAG
CCCTTTGTACCGGCCATTGTAGCATGCGTGAAGCCCAAGACATAAGGGGCATGATGATTTGACGTCATCCCCACCTTCCTCCGAGTTGACCCCGGCAGTC
TCCTATGAGTCCCCACCATGACGTGCTGGCAACATAGGACGAGGGTTGCGCTCGTTGCGGGACTTAACCCAACATCTCACGACACGAGCTGACGACAACC
ATGCACCACCTGTACACCAGTCCGAAGACGCGACCATCTCTGGCCGATTCCGGTGTATGTCAAGCCTTGGTAAGGTTCTTCGCGTTGCATCGAATTAATC
CGCATGCTCCGCCGCTTGTGCGGGCCCCCGTCAATTCCTTTGAGTTTTAGCCTTGCGGCCGTACTCCCCAGGCGGGGCACTTAATGCGTTAGCTGCGGCG
CGGAACTCGTGGAATGAGCCCCACACCTAGTGCCCAACGTTTACGGCATGGACTACCAGGGTATCTAATCCTGTTCGCTCCCCATGCTTTCGCTCCTCAG
CGTCAGTAGTGGCCCAGTGACCTGCCTTCGCCATCGGTGTTCCTCCTGATATCTGCGCATTCCACCGCTACACCAGGAATTCCAGTCACCCCTACCACAC
TCTAGTCTGCCCGTACCCGATGCAAGCTCAAGGTTGAGCCTTGAGTTTTCACACCAGACGCGACAAACCGCCTACGAGCTCTTTACGCCCAATAATTCCG
GACAACGCTTGCGCCCTACGTATTACCGCGGCTGCTGGCACGTAGTTAGCCGGCGCTTCTTCTGCAGGTACCGTCACTTTCGCTTCTTCCCTACTGAAAG
AGGTTTACAACCCGAAGGCCTTCATCCCTCACGCGGCGTCGCTGCATCAGGCTTTCGCCCATTGTGCAATATTCCCCACTGCTGCCTCCCGTAGGAGTCT
GGGCCGTGTCTCAGTCCCAGTGTGGCCGGTCGCCCTCTCAGGCCGGCTACCCGTCGACGCCTTGGTGAGCCGTTACCTCACCAACTAGCTGATAGGCCGC
GAGTCCATCCTTGACCGAAAAACTTTCCAGACGCCGACCATGCGGTCGCGTCTCGTATCCGGTATTAGCCCCGGTTTCCCGGAGTTATCCCAGAGTCAAG
GGCAGGTTACTCACGTGTTACTCACCCGTTCGCCACTGATCAACCAGAGCAAGCTCTAGCGTCACCGTTCGACTTGCA

>EB83
TGCAGTCGAGCGAACGATGGGAGCTTGCTCCCTGAGATTAGCGGCGGACGGGCGAGTAACACGTGGGCAAGCTGCCTATAGGACTGGGATAACTTCGGGA
AACCGGAGCTAATACCGGATACGTTCTTTTCTCGCATGAGAAAAGATGGAAAGACGGTTTACGCTGTCACTTATAGATGGGCCCGCGGCGCATTAGCTAG
TTGGTGAGGTAATGGATCACCTATGCGACGATGCATAGCAGACCTGAGAGGGTGATCAGCCACACTGGGACTGAAACACTGCCCAAACTCCTACGGGAGG
CAGCAGTACGGAATCTTCCGCAATGGACGAAAGTCTGACGGAGCAACCCCCGCGTGAACGAAGAAGGCCTTCGGGTCGTAAAGTTCTGTTGTTAGGGAAG
AACAAGTACCAGAGTAACTGCTGGTACCTTGACGGTACCTAACCAGAAAGCCACGGCTAACTACGTGCCAGCAGCCGCGGTAATACGTAGGTGGCAAGCG
TTGTCCGGAATTATTGGGCGTAAAGCGCGCGCAGGTGGTTCCTTAAGTCTGATGTGAAAGCCCACGGCTCAACCGTGGAGGGTCATTGGAAACTGGGGAA
CTTGAGTGCAGAAGAGGAAAGTGGAATTCCAAGTGTAGCGGTGAAATGCGTAGAGATTTGGAGGAACACCAGTGGCGAAGGCGACTTTCTGGTCTGTAAC
TGACACTGAGGCGCGAAAGCGTGGGGAGCAAACAGGATTAGATACCCTGGTAGTCCACGCCGTAAACGATGAGTGCTAAGTGTTAGAGGGTTTCCGCCCT
TTAGTGCTGCAGCTAACGCATTAAGCACTCCGCCTGGGGAGTACGGCCGCAAGGCTGAAACTCAAAGGAATTGACGGGGGCCCGCACAAGCGGTGGAGCA
TGTGGTTTAATTCGAAGCAACGCGAAGAACCTTACCAGGTCTTGACATCCTCTGACAACCCTAGAGATAGGGCTTTCCCCTTCGGGGGACAGAGTGACAG
GTGGTGCATGGTTGTCGTCAGCTCGTGTCGTGAGATGTTGGGTTAAGTCCCGCAACGAGCGCAACCCTTGATCTTAGTTGCCAGCATTCAGTTGGGCACT
CTAAGGTGACTGCCGGTGACAAACCGGAGGAAGGTGGGGATGACGTCAAATCATCATGCCCCTTATGACCTGGGCTACACACGTGCTACAATGGATGGTA
CAAAGGGCTGCAAACCTGCGAAGGTAAGCGAATCCCATAAAGCCATTCTCAGTTCGGATTGCAGGCTGCAACTCGCCTGCATGAAGCCGGAATCGCTAGT
AATCGCGGATCAGCATGCCGCGGTGAATACGTTCCCGGGCCTTGTACACACCGCCCGTCACACCACGAGAGTTTGTAACACCCGAAGTCGGTGAGGTAAC
CTTCATGGAGCC

>EB84
TGCAGTCGAGCGGACGTTTTTGAAGCTTGCTTCAAAAACGTTAGCGGCGGACGGGTGAGTAACACGTGGGCAACCTACCTTATCGACTGGGATAACTCCG
GGAAACCGGGGCTAATACCGGATAACATCTAGCACCTCCTGGTGCCGGATTAAAAGAGGGCTTCTTGCTCTCACGATGAGATGGGCCCGCGGCGCATTAG
CTAGTTGGAGAGGTAACGGCTCCCCAAGGCGACGATGCGTAGCCGACCTGAGAGGGTGATCGGCCACACTGGGACTGAGACACGGCCCAGACTCCTACGG
GAGGCAGCAGTAGGGAATCTTCCGCAATGGACGAAAGTCTGACGGAGCAACGCCGCGTGAGTGATGAAGGGTTTCGGCTCGTAAAGCTCTGTTATGAGGG
AAGAACACGTACCGTTCGAATAGGGCGGTACCTTGACGGTACCTCATCAGAAAGCCACGGCTAACTACGTGCCAGCAGCCGCGGTAATACGTAGGTGGCA
AGCGTTGTCCGGAATTATTGGGCGTAAAGCGCGCGCAGGCGGCCTTTTAAGTCTGATGTGAAATCTTGCGGCTCAACCGCAAGCGGCCATTGGAAACTGG
GAGGCTTGAGTACAGAAGAGGAGAGTGGAATTCCACGTGTAGCGGTGAAATGCGTAGATATGTGGAGGAACACCAGTGGCGAAGGCGACTCTCTGGTCTG
TAACTGACGCTGAGGCGCGAAAGCGTGGGGAGCAAACAGGATTAGATACCCTGGTAGTCCACGCCGTAAACGATGAGTGCTAGGTGTTAGGGGNTTTCGA
TGCCCNGTAGTGCCGAANGTTAACACATTAAGCNACTCCGCCTGGGGNAGTACGGCCGCAAGGCTGAAANCTCAAAGGAATTGACGGGGGCCCGCACAAG
CAGTGGAGCATGTGGTTTAATTCGAAGCAACGCGAAGAACCTTACCAGGNTCTTGACATCCTTTGACCACTCTGGAGACAGAGCTTCCCCTTCGGGGGNC
AAAGTGACAGGTGGTGCATGGTTGTCGTCAGCTCGTGTCGTGAGATGTTGGGTTAAGTCCCGCAACGAGCGCAACCCTTGACCTTAGTTGCCAGCATTTA
GTTGGGCACTCTAAGGTGACTGCCGGTGACAAACCGGAGGAAGGTGGGGATGACGTCAAATCATCATGCCCCTTATGACCTGGGCTACACACGTGCTACA
ATGGATGGTACAAAGGGTTGCGAAGCCGCGAGGTGAAGCCAATCCCATAAAGCCATTCTCAGTTCGGATTGTAGGCTGCAACTCGCCTGCATGAAGCTGG
AATTGCTAGTAATCGCGGATCAGCATGCCGCGGTGAATACGTTCCCGGGCCTTGTACACACCGCCCGTCACACCACGAGAGTTTGTAACACCCGAAGTCG
GTGAGGTAACC

>EB85
TGCAGTCGAGCGAATGGATTAAGAGCTTGCTCTTATGAAGTTAGCGGCGGACGGGTGAGTAACACGTGGGTAACCTGCCCATAAGACTGGGATAACTCCG
GGAAACCGGGGCTAATACCGGATAACATTTTGCACCGCATGGTGCGAAATTCAAAGGCGGCTTCGGCTGTCACTTATGGATGGACCCGCGTCGCATTAGC
TAGTTGGTGAGGTAACGGCTCACCAAGGCAACGATGCGTAGCCGACCTGAGAGGGTGATCGGCCACACTGGGACTGAGACACGGCCCAGACTCCTACGGG
AGGCAGCAGTAGGGAATCTTCCGCAATGGACGAAAGTCTGACGGAGCAACGCCGCGTGAGTGATGAAGGCTTTCGGGTCGTAAAACTCTGTTGTTAGGGA
AGAACAAGTGCTAGTTGAATAAGCTGGCACCTTGACGGTACCTAACCAGAAAGCCACGGCTAACTACGTGCCAGCAGCCGCGGTAATACGTAGGTGGCAA

GCGTTATCCGGAATTATTGGGCGTAAAGCGCGCGCAGGTGGTTTCTTAAGTCTGATGTGAAAGCCCACGGCTCAACCGTGGAGGGTCATTGGAAACTGGG
AGACTTGAGTGCAGAAGAGGAAAGTGGAATTCCATGTGTAGCGGTGAAATGCGTAGAGATATGGAGGAACACCAGTGGCGAAGGCGACTTTCTGGTCTGT
AACTGACACTGAGGCGCGAAAGCGTGGGGAGCAAACAGGATTAGATACCCTGGTAGTCCACGCCGTAAACGATGAGTGCTAAGTGTTAGAGGGTTTCCGC
CCTTTAGTGCTGAAGTTAACGCATTAAGCACTCCGCCTGGGGAGTACGGCCGCAAGGCTGAAACTCAAAGGAATTGACGGGGGCCCGCACAAGCGGTGGA
GCATGTGGTTTAATTCGAAGCAACGCGAAGAACCTTACCAGGTCTTGACATCCTCTGACAACCCTAGAGATAGGGCTTCCCCTTCGGGGGCAGAGTGACA
GGTGGTGCATGGTTGTCGTCAGCTCGTGTCGTGAGATGTTGGGTTAAGTCCCGCAACGAGCGCAACCCTTGATCTTAGTTGCCATCATTAAGTTGGGCAC
TCTAAGGTGACTGCCGGTGACAAACCGGAGGAAGGTGGGGATGACGTCAAATCATCATGCCCCTTATGACCTGGGCTACACACGTGCTACAATGGACGGT
ACAAAGAGCTGCAAGACCGCGAGGTGGAGCTAATCTCATAAAACCGTTCTCAGTTCGGATTGTAGGCTGCAACTCGCCTACATGAAGCTGGAATCGCTAG
TAATCGCGGATCAGCATGCCGCGGTGAATACGTTCCCGGGCCTTGTACACACCGCCCGTCACACCACGAGAGTTTGTAACACCCGAAGTCGGTGGGGTAA
CCTTTTTGGAGCCAGCCGCCT

>EB86
CGGCTGGCTCCTTACGGTTACCCCACCGACTTCGGGTGTTACAAACTCTCGTGGTGTGACGGGCGGTGTGTACAAGGCCCGGGAACGTATTCACCGCGGC
ATGCTGATCCGCGATTACTAGCGATTCCGGCTTCATGCAGGCGAGTTGCAGCCTGCAATCCGAACTGAGAATGGTTTTATGGGATTGGCTAAACCTCGCG
GTCTTGCAGCCCTTTGTACCATCCATTGTAGCACGTGTGTAGCCCAGGTCATAAGGGGCATGATGATTTGACGTCATCCCCACCTNTCCTCCGGTTTGTC
ACCGGCAGTCACCTTAGAGTGCCCAACTAAATGCTGGCAACTAAGATCAAGGGTTGCGCTCGTTGCGGGACTTAACCCAACATCTCACGACACGAGCTGA
CGACAACCATGCACCACCTGTCACTCTGTCCCCCGAAGGGGAACGTCCTATCTCTAGGAGTGTCAGAGGATGTCAAGACCTGGTAAGGTTCTTCGCGTTG
CTTCGAATTAAACCACATGCTCCACCGCTTGTGCGGGCCCCCGTCAATTCCTTTGAGTTTCAGCCTTGCGGCCGTACTCCCCAGGCGGAGTGCTTAATGC
GTTAGCTGCAGCACTAAAGGGCGGAAACCCTCTAACACTTAGCACTCATCGTTTACGGCGTGGACTACCAGGGTATCTAATCCTGTTTGCTCCCCACGCT
TTCGCGCCTCAGCGTCAGTTACAGACCAGAAAGCCGCCTTCGCCACTGGTGTTCCTCCACATCTCTACGCATTTCACCGCTACACGTGGAATTCCGCTTT
CCTCTTCTGCACTCAAGTCCCCCAGTTTCCAATGACCCTCCACGGTTGAGCCGTGGGCTTTCACATCAGACTTAAAGGACCGCCTGCGCGCGCTTTACGC
CCAATAATTCCGGACAACGCTTGCCACCTACGTATTACCGCGGCTGCTGGCACGTAGTTAGCCGTGGCTTTCTGGTTAGGTACCGTCAAGGTACCGGCAG
TTACTCCGGTACTTGTTCTTCCCTAACAACAGAGCTTTACGACCCGAAGGCCTTCATCGCTCACGCGGCGTTGCTCCATCAGACTTTCGTCCATTGTGGA
AGATTCCCTACTGCTGCCTCCCGTAGGAGTCTGGGCCGTGTCTCAGTCCCAGTGTGGCCGATCACCCTCTCAGGTCGGCTACGCATCGTCGCCTTGGTGA
GCCGTTACCTCACCAACTAGCTAATGCGCCGCGGGCCCATCTGTAAGTGTCAGCCGAAACCGTCTTTCAGCTTTTCCTCATGAGAGGAAAAGGATTATCC
GGTATTAGCACCGGTTTCCCGGTGTTATCCCAGTCTTACAGGCAGGTTGCCCACGTGTTACTCACCCGTCCGCCGCTACGACTCTCGAAGAGTCTCGCTC
GA

>EB87
CGAGCGGACAGATGGGAGCTTGCTCCCTGATGTTAGCGGCGGACGGGCGAGTAACACGTGGGTAACCTGCCTGTAAGACTGGGATAACTCCGGGAAACCG
GGGCTAATACCGGATGGTTGTCTGAACCGCATGGTTCAGACATAAAAGGTGGCTTCGGCTACCACTTACAGATGGACCCGCGGCGCATTAGCTAGTTGGT
GAGGTAACGGCTCACCAAGGCGACGATGCGTAGCCGACCTGAGAGGGTGATCGGCCACACTGGGACTGAGACACGGCCCAGACTCCTACGGGAGGCAGCA
GTAGGGAATCTTCCGCAATGGACGAAAGTCTGACGGAGCAACGCCGCGTGAGTGATGAAGGTTTTCGGATCGTAAAGCTCTGTTGTTAGGGAAGAACAAG
TGCCGTTCAAATAGGGGCGGCACCTTGACGGTACCTAACCAGAAAGCCACGGCTAACTACGTGCCAGCAGCCGCGGTAATACGTAGGTGGCAAGCGTT
GTCCGGAATTATTGGGGCGTAAAAGGGCTCGCAGGCGGTTTCTTAAGTCTGATGTGAAAGCCCCCGGCTCAACCGGGGAGGGTCATTGGAAACTGGGGAA
CTTGAGTGCAGAAGAGGAGAGTGGAATTCCACGTGTAGCGGTGAAATGCGTAGAGATGTGGAGGAACACCAGTGGCGAAGGCGACTCTCTGGTCTGTAAC
TGACGCTGAGGAGCGAAAGCGTGGGGAGCGAACAGGATTAGATACCCTGGTAGTCCACGCCGTAAACGATGAGTGCTAAGTGTTAGGGGGTTTCCGCCCC
TTAGTGCTGCAGCTAACGCATTAAGCACTCCGCCTGGGGAGTACGGTCGCAAGACTGAAACTCAAAGGAATTGACGGGGGCCCGCACAAGCGGTGGAGCA
TGTGGTTTAATTCGAAGCAACGCGAAGAACCTTACCAGGTCTTGACATCCTTCTGACAATCCTAGAGATAGGACGTCCCCTTCGGGGGCAGAGTGACAGG
TGGTGCATGGTTGTCGTCAGCTCGTGTCGTGAGATGTTGGGTTAAGTCCCGCAACGAGCGCAACCCTTGATCTTAGTTGCCAGCATTCAGTTGGGCACTC
TAAGGTGACTGCCGGTGACAAACCGGAGGAAGGTGGGGATGACGTCAAATCATCATGCCCCTTATGACCTGGGCTACACACGTGCTACAATGGACAGAAC
AAAGGGCAGCGAAACCGCGAGGTTAAGCCAATCCCACAAATCTGTTCTCAGTTCGGATCGCAGTCTGCAACTCGACTGCGTGAAGCTGGAATCGCTAGTA
ATCGCGGATCAGCATGCCGCGGTGAATACGTTCCCGGGCCTTGTACACACCGCCCGTCACACCACGAGAGTTTGTAACACCCGAAGTCGGTGAGGTAACC
TTTATGGAGCCAGCCG

>EB88
GGCTGGCTCCATAAAGGTTACCTCACCGACTTCGGGTGTTGCAAACTCTCGTGGTGTGACGGGCGGTGTGTACAAGGCCCGGGAACGTATTCACCGCGGC
ATGCTGATCCGCGATTACTAGCGATTCCAGCTTCACGCAGTCGAGTTGCAGACTGCGATCCGAACTGAGAACAGATTTGTGGGATTGGCTAAACCTTGCG
GTCTCGCAGCCCTTTGTTCTGTCCATTGTAGCACGTGTGTAGCCCAGGTCATAAGGGGCATGATGATTTGACGTCATCCCCACCTTCCTCCGGTTTGTCA
CCGGCAGTCACCTTAGAGTGCCCAACTGAATGCTGGCAACTAAGATCAAGGGTTGCGCTCGTTGCGGGACTTAACCCAACATCTCACGACACGAGCTGAC
GACAACCATGCACCACCTGTCACTCTGTCCCCGAAGGGAAAGCCCTATCTCTAGGGTTGTCAGAGGATGTCAAGACCTGGTAAGGTTCTTCGCGTTGCTT
CGAATTAAACCACATGCTCCACCGCTTGTGCGGGCCCCCGTCAATTCCTTTGAGTTTCAGTCTTGCGACCGTACTCCCCAGGCGGAGTGCTTAATGCGTT
AGCTGCAGCACTAAGGGGCGGAAACCCCCTAACACTTAGCACTCATCGTTTACGGCGTGGACTACCAGGGTATCTAATCCTGTTCGCTCCCCACGCTTTC
GCTCCTCAGCGTCAGTTACAGACCAGAGAGTCGCCTTCGCCACTGGTGTTCCTCCACATCTCTACGCATTTCACCGCTACACGTGGAATTCCACTCTCCT
CTTCTGCACTCAAGTTTCCCAGTTTCCAATGACCCTCCCCGGTTGAGCCGGGGGCTTTCACATCAGACTTAAGAAACCGCCTGCGAGCCCTTTACGCCCA
ATAATTCCGGACAACGCTTGCCACCTACGTATTACCGCGGCTGCTGGCACGTAGTTAGCCGTGGCTTTCTGGTTAGGTACCGTCAAGGTGCAAGCAGTT
ACTCTTGCACTTGTTCTTCCCTAACAACAGAGCTTTACGATCCGAAAACCTTCATCACTCACGCGGCGTTGCTCCGTCAGACTTTCGTCCATTGCGGAAG
ATTCCCTACTGCTGCCTCCCGTAGGAGTCTGGGCCGTGTCTCAGTCCCAGTGTGGCCGATCACCCTCTCAGGTCGGCTACGCATCGTCGCCTTGGTGAGC
CGTTACCTCACCAACTAGCTAATGCGCCGCGGGTCCATCTGTAAGTGACAGCCGAAACCGTCTTTCATCCTTGAACCATGCGGTTCAAGGAACTATCCGG
TATTAGCTCCGGTTTCCCGGAGTTATCCCAGTCTTACAGGCAGGTTACCCACGTGTTACTCACCCGTCCGCCGCTAACATCCGGGAGCAAGCTCCCTTCT
GTCCGCTCGACTTGCA

>EB89
AGCGGCGGACGGGTGAGTAACACGTGGGCAACCTGCCTGTAAGACTGGGATAACTCCGGGAAACCGGAGCTAATACCGGATACTATGTCAAACCGCATGG
TTTGACATTCAAAGACGGTTTCGGCTGTCACTTACAGATGGGCCCGCGGCGCATTAGCTAGTTGGTGAGGTAATGGCTCACCAAGGCGACGATGCGTAGC
CGACCTGAGAGGGTGATCGGCCACACTGGGACTGAGACACGGCCCAGACTCCTACGGGAGGCAGCAGTAGGGAATCTTCCGCAATGGACGAAAGTCTGAC
GGAGCAACGCCGCGTGAGTGATGAAGGTTTTCGGATCGTAAAACTCTGTTGTCAGGGAAGAACAAGTGCCGGAGTAACTGCCGGCACCTTGACGGTACCT
GACCAGAAAGCCACGGCTAACTACGTGCCAGCAGCCGCGGTAATACGTAGGTGGCAAGCGTTGTCCGGAATTATTGGGCGTAAAGCGCGCGCAGGCGGTT
TCTTAAGTCTGATGTGAAAGCCCCCGGCTCAACCGGGGAGGGTCATTGGAAACTGGGAAACTTGAGTGCAGAAGAGGAGAGTGGAATTCCACGTGTAGCG
GTGAAATGCGTAGAGATGTGGAGGAACACCAGTGGCGAAGGCGACTCTCTGGTCTGTAACTGACGCTGAGGCGCGAAAGCGTGGGGAGCGAACAGGATTA
GATACCCTGGTAGTCCACGCCGTAAACGATGAGTGCTAAGTGTTAGAGGGTTTCCGCCCTTTAGTGCTGCAGCTAACGCATTAAGCACTCCGCCTGGGGA
GTACGGTCGCAAGACTGAAACTCAAAGGAATTGACGGGGGCCCGCACAAGCGGTGGAGCATGTGGTTTAATTCGAAGCAACGCGAAGAACCTTACCAGGT
CTTGACATCCTTTGCCACTTCTAGAGATAGAAGGTTCCCCTTCGGGGGACAAAGTGACAGGTGGTGCATGGTTGTCGTCAGCTCGTGTCGTGAGATGTTG
GGTTAAGTCCCGCAACGAGCGCAACCCTTGATCTTAGTTGCCAGCATTCAGTTGGGCACTCTAAGGTGACTGCCGGTGACAAACCGGAGGAAGGTGGGGA
TGACGTCAAATCATCATGCCCCTTATGACCTGGGCTACACACGTGCTACAATGGATGGTACAAAGGGCTGCGAGACCGCGAGGTTTAGCCAATCCCATAA
AACCATTCTCAGTTCGGATTGCAGGCTGCAACTCGCCTGCATGAAGCTGGAATCGCTAGTAATCGCGGATCAGCATGCCGCGGTGAATACGTTCCCGGGC
CTTGTACACACCGCCCGTCACACCACGAGAGTTTGCAACACCCGAAGTCGGTGGGGTAACCGCAAGGAGCCAGCCGCCTAAGGTGGGGTAGATGATTGGG
G

>EB90
GCGAACAGAGAAGGAGCTTGCTCCTTTGACGTTAGCGGCGGACGGGTGAGTAACACGTGGGCAACCTACCCTATAGTTTGGGATAACTCCGGGAAACCGG
GGCTAATACCGAATAATCTAGTTCACCTCATGGTGAACTATTAAAAGACGGTTTCGGCTGTCGCTATAGGATGGGCCCGCGGCGCATTAGCTAGTTGGTG
AGGTAATGGCTCACCAAGGCGACGATGCGTAGCCGACCTGAGAGGGTGATCGGCCACACTGGGACTGAGACACGGCCCAGACTCCTACGGGAGGCAGCAG
TAGGGAATCTTCCACAATGGGCGAAAGCCTGATGGAGCAACGCCGCGTGAGTGAAGAAGGATTTCGGTTCGTAAAACTCTGTTGTAAGGGAAGAACAAGT
ACAGTAGTAACTGGCTGTACCTTGACGGTACCTTATTAGAAAGCCACGGCTAACTACGTGCCAGCAGCCGCGGTAATACGTAGGTGGCAAGCGTTGTCCG
GAATTATTGGGCGTAAAGCGCGCGCAGGTGGTTTCTTAAGTCTGATGTGAAAGCCCACGGCTCAACCGTGGAGGGTCATTGGAAACTGGGAGACTTGAG
TGCAGAAGAGGATAGTGGAATTCCAAGTGTAGCGGTGAAATGCGTAGAGATTTGGAGGAACACCAGTGGCGAAGGCGACTATCTGGTCTGTAACTGACAC
TGAGGCGCGAAAGCGTGGGGAGCAAACAGGATTAGATACCCTGGTAGTCCACGCCGTAAACGATGAGTGCTAAGTGTTAGGGGGTTTCCGCCCCTTAGTG
CTGCAGCTAACGCATTAAGCACTCCGCCTGGGGAGTACGGTCGCAAGACTGAAACTCAAAGGAATTGACGGGGGCCCGCACAAGCGGTGGAGCATGTGGT
TTAATTCGAAGCAACGCGAAGAACCTTACCAGGTCTTGACATCCCGTTGACCACTGTAGAGATATAGTTTCCCCTTCGGGGGCAACGGTGACAGGTGGTG
CATGGTTGTCGTCAGCTCGTGTCGTGAGATGTTGGGTTAAGTCCCGCAACGAGCGCAACCCTTGATCTTAGTTGCCATCATTTAGTTGGGCACTCTAAGG
TGACTGCCGGTGACAAACCGGAGGAAGGTGGGGATGACGTCAAATCATCATGCCCCTTATGACCTGGGCTACACACGTGCTACAATGGACGATACAAACG
GTTGCCAACTCGCGAGAGGGAGCTAATCCGATAAAGTCGTTCTCAGTTCGGATTGTAGGCTGCAACTCGCCTACATGAAGCCGGAATCGCTAGTAATCGC
GGATCAGCATGCCGCGGTGAATACGTTCCCGGGCCTTGTACACACCGCCCGTCACACCACGAGAGTTTGTAACACCCGAAGTCGGTGAGGTAACCTTTGG
AGCCAGCCGCC

>EB91
GTCGAGCGCCCCGCAAGGGGAGCGGCAGACGGGTGAGTAACGCGTGGGAATCTACCGTGCCCTACGGAATAGCTCCGGGAAACTGGAATTAATACCGTAT
ACGCCCTACGGGGGAAAGATTTATCGGGGTATGATGAGCCCGCGTTGGATTAGCTAGTTGGTGGGGTAAAGGCCTACCAAGGCGACGATCCATAGCTGGT
CTGAGAGGATGATCAGCCACATTGGGACTGAGACACGGCCCAAACTCCTACGGGAGGCAGCAGTGGGGAATATTGGACAATGGGCGCAAGCCTGATCCAG
CCATGCCGCGTGAGTGATGAAGGTCTTAGGATTGTAAAGCTCTTTCACCGGTGAAGATAATGACGGTAACCGGAGAAGAAGCCCCGGCTAACTTCGTGCC
AGCAGCCGCGGTAATACGAAGGGGGCTAGCGTTGTTCGGAATTACTGGGCGTAAAGCGCACGTAGGCGGATATTTAAGTCAGGGGTGAAATCCCAGAGCT
CAACTCTGGAACTGCCTTTGATACTGGGTATCTTGAGTATGGAAGAGGTGAGTGGAATTCCGAGTGTAGAGGTGAAATTCGTAGATATTCGGAGGAACAC
CAGTGGCGAAGGCGGCTCACTGGTCCATTACTGACGCTGAGGTGCGAAAGCGTGGGGAGCAAACAGGATTAGATACCCTGGTAGTCCACGCCGTAAACGA
TGAATGTTAGCCGTCGGGCAGTATACTGTTCGGTGGCGCAGCTAACGCATTAAACATTCCGCCTGGGGAGTACGGTCGCAAGATTAAAACTCAAAGGAAT
TGACGGGGGCCCGCACAAGCGGTGGAGCATGTGGTTTAATTCGAAGCAACGCGCAGAACCTTACCAGCTCTTGACATCCGGGTCGCGGACAGTGGAGACA
TTGTCCTTCAGTTAGGCTGGACCCAGGACAGGTGCTGCATGGCTGTCGTCAGCTCGTGTCGTGAGATGTTGGGTTAAGTCCCGCAACGAGCGCAACCCTC
GCCCTTAGTTGCCAGCATTCAGTTGGGCACTCTAAGGGGACTGCCGGTGATAAGCCGAGAGGAAGGTGGGGATGACGTCAAGTCCTCATGGCCCTTACGG
GCTGGGCTACACACGTGCTACAATGGTGGTGACAGTGGGCAGCGAGACAGCGATGTCGAGCTAATCTCCAAAAGCCATCTCAGTTCGGATTGCACTCTGC
AACTCGAGTGCATGAAGTTGGAATCGCTAGTAATCGCGGATCAGCATGCCGCGGTGAATACGTTCCCGGGCCTTGTACACACCGCCCGTCACACCATGGG
AGTTGGTTTTACCCGAAGGTAGTGCGCTAACCCGCAAGGGAGGCAG

>EB92
TCGAGCGAACTGATTAGAAGCTTGCTTCTATGACGTTAGCGGCGGACGGGTGAGTAACACGTGGGCAACCTGCCTGTAAGACTGGGATAACTTCGGGAAA
CCGAAGCTAATACCGGATAGGATCTTCTCCTTCATGGGAGATGATTGAAAGATGGTTTCGGCTATCACTTACAGATGGGCCCGCGGTGCATTAGCTAGTT
GGTGAGGTAACGGCTCACCAAGGCAACGATGCATAGCCGACCTGAGAGGGTGATCGGCCACACTGGGACTGAGACACGGCCCAGACTCCTACGGGAGGCA
GCAGTAGGGAATCTTCCGCAATGGACGAAAGTCTGACGGAGCAACGCCGCGTGAGTGATGAAGGCTTTCGGGTCGTAAAACTCTGTTGTTAGGGAAGAAC
AAGTACGAGAGTAACTGCTCGTACCTTGACGGTACCTAACCAGAAAGCCACGGCTAACTACGTGCCAGCAGCCGCGGTAATACGTAGGTGGCAAGCGTTA
TCCGGAATTATTGGGCGTAAAGCGCGCGCAGGCGGTTTCTTAAGTCTGATGTGAAAGCCCACGGCTCAACCGTGGAGGGTCATTGGAAACTGGGGAACTT
GAGTGCAGAAGAGAAAAGCGGAATTCCACGTGTAGCGGTGAAATGCGTAGAGATGTGGAGGAACACCAGTGGCGAAGGCGGCTTTTTGGTCTGTAACTGA
CGCTGAGGCGCGAAAGCGTGGGGAGCAAACAGGATTAGATACCCTGGTAGTCCACGCCGTAAACGATGAGTGCTAAGTGTTAGAGGGTTTCCGCCCTTTA
GTGCTGCAGCTAACGCATTAAGCACTCCGCCTGGGGAGTACGGTCGCAAGACTGAAACTCAAAGGAATTGACGGGGGCCCGCACAAGCGGTGGAGCATGT
GGTTTAATTCGAAGCAACGCGAAGAACCTTACCAGGTCTTGACATCCTCTGACAACTCTAGAGATAGAGCGTTCCCCTTCGGGGGACAGAGTGACAGGTG
GTGCATGGGTTGTCGTCAGCTCGTGTCGTGAGATGTTGGGGTTAAGTCCCGCAACGAGCGCAACCCTTGATCTTAGTTGCCAGCATTTAGTTGGGCACTC
TAAGGTGACTGCCGGTGACAAACCGGAGGAAGGTGGGGATGACGTCAAATCATCATGCCCCTTATGACCTGGGCTACACACGTGCTACAATGGATGGTAC
AAAGGGCTGCAAGACCGCGAGGTCAAGCCAATCCCATAAAACCATTCTCAGTTCGGATTGTAGGCTGCAACTCGCCTACATGAAGCTGGAATCGCTAGTA
ATCGCGGATCAGCATGCCGCGGTGAATACGTTCCCGGGCCTTGTACACACCGCCCGTCACACCACGAGAGTTTGTAACACCCGAAGTCGGTGGAGTAACC
GTAAGGAGCTAGCCG

>EB93
CGGCTGGCTCCATGAAGGTTACCTCACCGACTTCGGGTGTTACAAACTCTCGTGGTGTGACGGGCGGTGTGTACAAGGCCCGGGAACGTATTCACCGCGG
CATGCTGATCCGCGATTACTAGCGATTCCGGCTTCATGCAGGCGAGTTGCAGCCTGCAATCCGAACTGAGAATGGCTTTATGGGATTCGCTTACCTTCGC
AGGTTTGCAGCCCTTTGTACCATCCATTGTAGCACGTGTGTAGCCCAGGTCATAAGGGGCATGATGATTTGACGTCATCCCCACCTTCCTCCGGTTTGTC
ACCGGCAGTCACCTTAGAGTGCCCAACTGAATGCTGGCAACTAAGATCAAGGGTTGCGCTCGTTGCGNGGACTTAACNCCAACATCTCACGACACGAGCT
GACGACAACCATGCACCACCTGTCACTCTGTCCCCCGAAGGGGAAAGCCCTATCTCTAGGGTTGTCAGAGGATGTCAAGACCTGGTAAGGTTCTTCGCGT
TGCTTCGAATTAAACCACATGCTCCACCGCTTGTGCGGGCCCCCGTCAATTCCTTTGAGTTTCAGCCTTGCGGCCGTACTCCCCAGGCGGAGTGCTTAAT
GCGTTAGCTGCAGCACTAAAGGGCGGAAACCCTCTAACACTTAGCACTCATCGTTTACGGCGTGGACTACCAGGGTATCTAATCCTGTTTGCTCCCCACG
CTTTCGCGCCTCAGTGTCAGTTACAGACCAGAAAGTCGCCTTCGCCACTGGTGTTCCTCCAAATCTCTACGCATTTCACCGCTACACTTGGAATTCCACT
TTCCTCTTCTGCACTCAAGTTCCCCAGTTTCCAATGACCCTCCACGGTTGAGCCGTGGGCTTTCACATCAGACTTAAGGAACCACCTGCGCGCGCTTTAC
GCCCAATAATTCCGGACAACGCTTGCCACCTACGTATTACCGCGGCTGCTGGCACGTAGTTAGCCGTGGCTTTCTGGTTAGGTACCGTCAAGGTACCAGC
AGTTACTCTGGTACTTGTTCTTCCCTAACAACAGAACTTTACGACCCGAAGGCCTTCTTCGTTCACGCGGCGTTGCTCCGTCAGACTTTCGTCCATTGCG
GAAGATTCCCTACTGCTGCCTCCCGTAGGAGTCTGGGCCGTGTCTCAGTCCCAGTGTGGCCGATCACCCTCTCAGGTCGGCTACGCATCGTCGCCTTGGT
GAGCCATTACCTCACCAACTAGCTAATGCGCCGCGGGCCCATCTATAAGTGACAGCGTAAACCGTCTTTCCATCTTCTCTCATGCGAGAGAAGAACGTAT
CCGGTATTAGCTCCGGTTTCCCGAAGTTATCCCAGTCTTATAGGCAGGTTGCCCACGTGTTACTCACCCGTCCGCCGCTAATCTCAGGGAGCAAGCTCCC
GTCGATTCGCTCGACTTGCA

>EB94
CGAGCGAACCGATTAAGAGCTTGCTCTTAAGAAGTTAGCGGCGGACGGGTGAGTAACACGTAGGTAACCTGCCTATAAGACTGGGATAACTCCGGGAAAC
CGGGGCTAATACCGGATAACATTTTGCACCGCATGGTGCGAAATTGAAAGGCGGCTTCGGCTGTCACTTATAGATGGACCTGCGGCGCATTAGCTAGTTG
GTGAGGTAACGGCTCACCAAGGCGACGATGCGTAGCCGACCTGAGAGGGTGATCGGCCACACTGGGACTGAGACACGGCCCAGACTCCTACGGGAGGCAG
CAGTAGGGAATCTTCCGCAATGGACGAAAGTCTGACGGAGCAACGCCGCGTGAGCGATGAAGGCCTTCGGGTCGTAAAGCTCTGTTGTTAGGGAAGAACA
AGTGCTAGTTGAATAAGCTGGCACCTTGACGGTACCTAACCAGAAAGCCACGGCTAACTACGTGCCAGCAGCCGCGGTAATACGTAGGTGGCAAGCGTTA
TCCGGAATTATTGGGCGTAAAGCGCGCGCAGGTGGTTTCTTAAGTCTGATGTGAAAGCCCACGGCTCAACCGTGGAGGGTCATTGGAAACTGGGAGACTT
GAGTGCAGAAGAGGAAAGTGGAATTCCATGTGTAGCGGTGAAATGCGTAGAGATATGGAGGAACACCAGTGGCGAAGGCGACTTTCTGGTCTGCAACTGA
CACTGAGGCGCGAAAGCGTGGGGAGCAAACAGGATTAGATACCCTGGTAGTCCACGCCGTAAACGATGAGTGCTAAGTGTTAGAGGGTTTCCGCCCTTTA
GTGCTGAAGTTAACGCATTAAGCACTCCGCCTGGGGAGTACGGCCGCAAGGCTGAAACTCAAAGGAATTGACGGGGGCCCGCACAAGCGGTGGAGCATGT

GGTTTAATTCGAAGCAACGCGAAGAACCTTACCAGGTCTTGACATCCTCTGACAACCCTAGAGATAGGGCTTCCCCTTCGGGGGCAGAGTGACAGGTGGT
GCATGGTTGTCGTCAGCTCGTGTCGTGAGATGTTGGGTTAAGTCCCGCAACGAGCGCAACCCTTGATCTTAGTTGCCATCATTAAGTTGGGCACTCTAAG
GTGACTGCCGGTGACAAACCGGAGGAAGGTGGGGATGACGTCAAATCATCATGCCCCTTATGACCTGGGCTACACACGTGCTACAATGGACGGTACAAAG
AGTCGCAAGACCGCGAGGTGGAGCTAATCTCATAAAACCGTTCTCAGTTCGGATTGTAGGCTGCAACTCGCCTACATGAAGCTGGAATCGCTAGTAATCG
CGGATCAGCATGCCGCGGTGAATACGTTCCCGGGCCTTGTACACACCGCCCGTCACACCACGAGAGTTTGTAACACCCGAAGTCGGTGGGGTAACCTTTG
GAGCCA

>EB95
AGCTTGCTCCCTGAAGTCAGCGGCGGACGGGTGAGTAACACGTGGGCAACCTGCCTGTAAGACTGGGATAACTCCGGGAAACCGGGGCTAATACCGGATA
ACTCTTTTCCTCACATGAGGAAAAGCTGAAAGATGGTTTCGGCTATCACTTACAGATGGGCCCGCGGCGCATTAGCTAGTTGGTGAGGTAACGGCTCACC
AAGGCCACGATGCGTAGCCGACCTGAGAGGGTGATCGGCCACACTGGGACTGAGACACGGCCCAGACTCCTACGGGAGGCAGCAGTAGGGAATCTTCCGC
AATGGACGAAAGTCTGACGGAGCAACGCCGCGTGAGTGATGAAGGTTTTCGGATCGTAAAACTCTGTTGTCAGGGAAGAACAAGTACCGGAGTAACTGCC
GGTACCTTGACGGTACCTGACCAGAAAGCCACGGCTAACTACGTGCCAGCAGCCGCGGTAATACGTAGGTGGCAAGCGTTGTCCGGAATTATTGGGCGTA
AAGCGCGCGCAGGCGGTTCCTTAAGTCTGATGTGAAAGCCCCCGGCTCAACCGGGGAGGGTCATTGGAAACTGGGGAACTTGAGTGCAGAAGAGAAGAGT
GGAATTCCACGTGTAGCGGTGAAATGCGTAGAGATGTGGAGGAACACCAGTGGCGAAGGCGACTCTTTGGTCTGTAACTGACGCTGAGGCGCGAAAGCGT
GGGGAGCAAACAGGATTAGATACCCTGGTAGTCCACGCCGTAAACGATGAGTGCTAAGTGTTAGAGGGTTTCCGCCCTTTAGTGCTGCAGCAAACGCATT
AAGCACTCCGCCTGGGGAGTACGGCCGCAAGGCTGAAACTCAAAGGAATTGACGGGGGCCCGCACAAGCGGTGGAGCATGTGGTTTAATTCGAAGCAACG
CGAAGAACCTTACCAGGTCTTGACATCTCCTGACAACCCTAGAGATAGGGCGTTCCCCTTCGGGGGACAGGATGACAGGTGGTGCATGGTTGTCGTCAGC
TCGTGTCGTGAGATGTTGGGTTAAGTCCCGCAACGAGCGCAACCCTTGATCTTAGTTGCCAGCATTCAGTTGGGCACTCTAAGGTGACTGCCGGTGACAA
ACCGGAGGAAGGTGGGGATGACGTCAAATCATCATGCCCCTTATGACCTGGGCTACACACGTGCTACAATGGATGGTACAAAGGGCTGCAAGACCGCGAG
GTTAAGCGAATCCCATAAAACCATTCTCAGTTCGGATTGCAGGCTGCAACTCGCCTGCATGAAGCCGGAATCGCTAGTAATCGCGGATCAGCATGCCGCG
GTGAATACGTTCCCGGGCCTTGTACACACCGCCCGTCACACCACGAGAGTTTGTAACACCCGAAGTCGGTGGGGTAACCTTTGGAGCCA

>EB96
GTCGAGCGAATGGATTGAGAGCTTGCTCTCAAGAAGTTAGCGGCGGACGGGTGAGTAACACGTGGGTAACCTGCCCATAAGACTGGGATAACTCCGGGAA
ACCGGGGCTAATACCGGATAACATTTTGAACTGCATGGTTCGAAATTGAAAGGCGGCTTCGGCTGTCACTTATGGATGGACCCGCGTCGCATTAGCTAGT
TGGTGAGGTAACGGCTCACCAAGGCAACGATGCGTAGCCGACCTGAGAGGGTGATCGGCCACACTGGGACTGAGACACGGCCCAGACTCCTACGGGAGGC
AGCAGTAGGGAATCTTCCGCAATGGACGAAAGTCTGACGGAGCAACGCCGCGTGAGTGATGAAGGCTTTCGGGTCGTAAAACTCTGTTGTTAGGGAAGAA
CAAGTGCTAGTTGAATAAGCTGGCACCTTGACGGTACCTAACCAGAAAGCCACGGCTAACTACGTGCCAGCAGCCGCGGTAATACGTAGGTGGCAAGCGT
TATCCGGAATTATTGGGCGTAAAGCGCGCGCAGGTGGTTTCTTAAGTCTGATGTGAAAGCCCACGGCTCAACCGTGGAGGGTCATTGGAAACTGGGAGAC
TTGAGTGCAGAAGAGGAAAGTGGAATTCCATGTGTAGCGGTGAAATGCGTAGAGATATGGAGGAACACCAGTGGCGAAGGCGACTTTCTGGTCTGTAACT
GACACTGAGGCGCGAAAGCGTGGGGAGCAAACAGGATTAGATACCCTGGTAGTCCACGCCGTAAACGATGAGTGCTAAGTGTTAGAGGGTTTCCGCCCTT
TAGTGCTGAAGTTAACGCATTAAGCACTCCGCCTGGGGAGTACGGCCGCAAGGCTGAAACTCAAAGGAATTGACGGGGGCCCGCACAAGCGGTGGAGCA
TGTGGTTTAATTCGAAGCAACGCGAAGAACCTTACCAGGTCTTGACATCCTCTGAAAACCCTAGAGATAGGGCTTCTCCTTCGGGAGCAGAGTGACAGGT
GGTGCATGGTTGTCGTCAGCTCGTGTCGTGAGATGTTGGGTTAAGTCCCGCAACGAGCGCAACCCTTGATCTTAGTTGCCATCATTAAGTTGGGCACTCT
AAGGTGACTGCCGGTGACAAACCGGAGGAAGGTGGGGATGACGTCAAATCATCATGCCCCTTATGACCTGGGCTACACACGTGCTACAATGGACGGTACA
AAGAGCTGCAAGACCGCGAGGTGGAGCTAATCTCATAAAACCGTTCTCAGTTCGGATTGTAGGCTGCAACTCGCCTACATGAAGCTGGAATCGCTAGTAA
TCGCGGATCAGCATGCCGCGGTGAATACGTTCCCGGGCCTTGTACACACCGCCCGTCACACCACGAGAGTTTGTAACACCCGAAGTCGGTGGGGTAACCT
TTTTGGAGCCAGCCG

>EB97
TGCAAGTCGAGCGAATGGATTGAGAGCTTGCTCTCAAGAAGTTAGCGGCGGACGGGTGAGTAACACGTGGGTAACCTGCCCATAAGACTGGGATAACTCC
GGGAAACCGGGGCTAATACCGGATAACATTTTGAACTGCATGGTTCGAAATTGAAAGGCGGCTTCGGCTGTCACTTATGGATGGACCCGCGTCGCATTAG
CTAGTTGGTGAGGTAACGGCTCACCAAGGCAACGATGCGTAGCCGACCTGAGAGGGTGATCGGCCACACTGGGACTGAGACACGGCCCAGACTCCTACGG
GAGGCAGCAGTAGGGAATCTTCCGCAATGGACGAAAGTCTGACGGAGCAACGCCGCGTGAGTGATGAAGGCTTTCGGGTCGTAAAACTCTGTTGTTAGGG
AAGAACAAGTGCTAGTTGAATAAGCTGGCACCTTGACGGTACCTAACCAGAAAGCCACGGCTAACTACGTGCCAGCAGCCGCGGTAATACGTAGGTGGCA
AGCGTTATCCGGAATTATTGGGCGTAAAGCGCGCGCAGGTGGTTTCTTAAGTCTGATGTGAAAGCCCACGGCTCAACCGTGGAGGGTCATTGGAAACTGG
GAGACTTGAGTGCAGAAGAGGAAAGTGGAATTCCATGTGTAGCGGTGAAATGCGTAGAGATATGGAGGAACACCAGTGGCGAAGGCGACTTTCTGGTCTG
TAACTGACACTGAGGCGCGAAAGCGTGGGGAGCAAACAGGATTAGATACCCTGGTAGTCCACGCCGTAAACGATGAGTGCTAAGTGTTAGAGGGTTTCCG
CCCTTTAGTGCTGAAGTTAACGCATTAAGCACTCCGCCTGGGGAGTACGGCCGCAAGGCTGAAACTCAAAGGAATTGACGGGGGCCCGCACAAGCGGTGG
AGCATGTGGTTTAATTCGAAGCAACGCGAAGAACCTTACCAGGTCTTGACATCCTCTGAAAACCCTAGAGATAGGGCTTCTCCTTCGGGAGCAGAGTGAC
AGGTGGTGCATGGTTGTCGTCAGCTCGTGTCGTGAGATGTTGGGTTAAGTCCCGCAACGAGCGCAACCCTTGATCTTAGTTGCCATCATTAAGTTGGGCA
CTCTAAGGTGACTGCCGGTGACAAACCGGAGGAAGGTGGGGATGACGTCAAATCATCATGCCCCTTATGACCTGGGCTACACACGTGCTACAATGGACGG
TACAAAGAGCTGCAAGACCGCGAGGTGGAGCTAATCTCATAAAACCGTTCTCAGTTCGGATTGTAGGCTGCAACTCGCCTACATGAAGCTGGAATCGCTA
GTAATCGCGGATCAGCATGCCGCGGTGAATACGTTCCCGGGCCTTGTACACACCGCCCGTCACACCACGAGAGTTTGTAACACCCGAAGTCGGTGGGGTA
ACCTTTATGGAGCCAGCCGCCT

>EB98
TGCAAGTCGAGCGGACAGATGGGAGCTTGCTCCCTGATGTTAGCGGCGGACGGGTGAGTAACACGTGGGTAACCTGCCTGTAAGACTGGGATAACTCCGG
GAAACCGGGGCTAATACCGGATGGTTGTCTGAACCGCATGGTTCAGACATAAAAGGTGGCTTCGGCTACCACTTACAGATGGACCCGCGGCGCATTAGCT
AGTTGGTGAGGTAACGGCTCACCAAGGCGACGATGCGTAGCCGACCTGAGAGGGTGATCGGCCACACTGGGACTGAGACACGGCCCAGACTCCTACGGGA
GGCAGCAGTAGGGAATCTTCCGCAATGGACGAAAGTCTGACGGAGCAACGCCGCGTGAGTGATGAAGGTTTTCGGATCGTAAAGCTCTGTTGTTAGGGAA
GAACAAGTGCCGTTCAAATAGGGCGGCACCTTGACGGTACCTAACCAGAAAGCCACGGCTAACTACGTGCCAGCAGCCGCGGTAATACGTAGGTGGCAAG
CGTTGTCCGGAATTATTGGGCGTAAAGGGCTCGCAGGCGGTTTCTTAAGTCTGATGTGAAAGCCCCCGGCTCAACCGGGGAGGGTCATTGGAAACTGGGG
AACTTGAGTGCAGAAGAGGAGAGTGGAATTCCACGTGTAGCGGTGAAATGCGTAGAGATGTGGAGGAACACCAGTGGCGAAGGCGACTCTCTGGTCTGTA
ACTGACGCTGAGGAGCGAAAGCGTGGGGAGCGAACAGGATTAGATACCCTGGTAGTCCACGCCGTAAACGATGAGTGCTAAGTGTTAGGGGGTTTCCGC
CCCTTAGTGCTGCAGCTAACGCATTAAGCACTCCGCCTGGGGAGTACGGTCGCAAGACTGAAACTCAAAGGAATTGACGGGGGCCCGCACAAGCGGTGGA
GCATGTGGTTTAATTCGAAGCAACGCGAAGAACCTTACCAGGTCTTGACATCCTCTGACAATCCTAGAGATAGGACGTCCCCTTCGGGGGCAGAGTGACA
GGTGGTGCATGGTTGTCGTCAGCTCGTGTCGTGAGATGTTGGGTTAAGTCCCGCAACGAGCGCAACCCTTGATCTTAGTTGCCAGCATTCAGTTGGGCAC
TCTAAGGTGACTGCCGGTGACAAACCGGAGGAAGGTGGGGATGACGTCAAATCATCATGCCCCTTATGACCTGGGCTACACACGTGCTACAATGGACAGA
ACAAAGGGCAGCGAAACCGCGAGGTTAAGCCAATCCCACAAATCTGTTCTCAGTTCGGATCGCAGTCTGCAACTCGACTGCGTGAAGCTGGAATCGCTAG
TAATCGCGGATCAGCATGCCGCGGTGAATACGTTCCCGGGCCTTGTACACACCGCCCGTCACACCACGAGAGTTTGTAACACCCGAAGTCGGTGAGGTAA
CCTTTAGGAGCCAGCC

>EB99
GAGGTCTAGCCAATCCCATAAAACCATTCTCAGTTCGGATTGCAGGCTGCAACTCGCATGCATGAAACAGGAATCGTAGATAAAGATGGAGGATCAGTAA
GTGGTAACAAAGTATCCGTAGAGTTTGATCATGGCTCAGTAAGTCGTAACAAGGTAMCCGTAGAGTTTGATCATGGCTCAGTAAGTCGTAACAGGGTAAC
CGTAAAGTTCCGGGAAACCGGAGCTAATACCGGATACTATGTCAAACCGCATGGTTTGACATTCAAAGACGGTTTCGGCTGTCACTTACAGATGGGCCCG

CGGCGCATTAGCTAGTTGGTGAGGTAATGGCTCACCAAGGCGACGATGCGTAGCCGACCTGAGAGGGTGATCGGCCACACTGGGACTGAGACACGGCCCA
GACTCCTACGGGAGGCAGCAGTAGGGAATCTTCCGCAATGGACGAAAGTCTGACGGAGCAACGCCGCGTGAGTGATGAAGGTTTTCGGATCGTAAAACTC
TGTTGTCAGGGAAGAACAAGTGCCGGAGTAACTGCCGGCACCTTGACGGTACCTGACCAGAAAGCCACGGCTAACTACGTGCCAGCAGCCGCGGTAATAC
GTAGGTGGCAAGCGTTGTCCGGAATTATTGGGCGTAAAGCGCGCGCAGGCGGTTTCTTAAGTCTGATGTGAAAGCCCCCGGCTCAACCGGGGAGGGTCAT
TGGAAACTGGGAAACTTGAGTGCAGGAGAGGAGAGTGGAATTCCACGTGTAGCGGTGAAATGCGTAGAGATGTGGAGGAACACCAGTGGCGAAGGCGACT
CTCTGGTCTGTAACTGACGCTGAGGCGCGAAAGCGTGCGGAGCGAACA

>EB100
AGGCGGCTGGCTCCATAAAGGTTACCCCACCGACTTCGGGTGTTACAAACTCTCGTGGTGTGACGGGCGGTGTGTACAAGGCCCGGGAACGTATTCACCG
CGGCATGCTGATCCGCGATTACTAGCGATTCCAGCTTCATGTAGGCGAGTTGCAGCCTACAATCCGAACTGAGAACGGTTTTATGAGATTAGCTCCACCT
CGCGGTCTTGCAGCTCTTTGTACCGTCCATTGTAGCACGTGTGTAGCCCAGGTCATAAGGGGCATGATGATTTGACGTCATCCCCACCTTCCTCCGGTTT
GTCACCGGCAGTCACCTTAGAGTGCCCAACTTAATGATGGCAACTAAGATCAAGGGTTGCGCTCGTTGCGGGACTTAACCCAACATCTCACGACACGAGC
TGACGACAACCATGCACCACCTGTCACTCTGCTCCCGAAGGAGAAGCTCTATCTCTAGAGTTTTCAGAGGATGTCAAGACCTGGTAAGGTTCTTCGCGTT
GCTTCGAATTAAACCACATGCTCCACCGCTTGTGCGGGCCCCCGTCAATTCCTTTGAGTTTCAGCCTTGCGGCCGTACTCCCCAGGCGGAGTGCTTAATG
CGTTAACTTCAGCACTAAAGGGGCGGAAACCCTCTAACACTTAGCACTCATCGTTTACGGCGTGGACTACCAGGGTATCTAATCCTGTTTGCTCCCCACG
CTTTCGCGCCTCAGTGTCAGTTACAGACCAGAAAGTCGCCTTCGCCACTGGTGTTCCTCCATATCTCTACGCATTTCACCGCTACACATGGAATTCCACT
TTCCTCTTCTGCACTCAAGTCTCCCAGTTTCCAATGACCCTCCACGGTTGAGCCGTGGGCTTTCACATCAGACTTAAGAAACCACCTGCGCGCGCTTTAC
GCCCAATAATTCCGGATAACGCTTGCCACCTACGTATTACCGCGGCTGCTGGCACGTAGTTAGCCGTGGCTTTCTGGTTAGGTACCGTCAAGGTGCCAGC
TTATTCAACTAGCACTTGTTCTTCCCTAACAACAGAGTTTTACGACCCGAAAGCCTTCATCACTCACGCGGCGTTGCTCCGTCAGACTTTCGTCCATTGC
GGAAGATTCCCTACTGCTGCCTCCCGTAGGAGTCTGGGCCGTGTCTCAGTCCCAGTGTGGCCGATCACCCTCTCAGGTCGGCTACGCATCGTCGCCTTGG
TGAGCCGTTACCTCACCAACTAGCTAATGCGACGCGGGTCCATCCATAAGTGACAGCCGAAGCCGCCTTTCAATTTCGAACTATGCAGTTCAAAATATTA
TCCGGTATTAGCCCCGGTTTCCCGGAGTTATCCCAGTCTTATGGGTAGGTTACCCACGTGTTACTCACCCGTCCGCCGCTAACTTCATAAGAGCAAGCTC
TTAATCCATTCGCTCGACTTGC

>EB101
ATTGGCGAACGGGTGAGTAACACGTGAGCAACCTGCCCTGGACTCTGGGATAAGCGCTGGAAACGGTGTCTAATACTGGATATGAGCCTCTATCGCATGG
TGGGGGTTGGAAAGATTTTTCGGTCTGGGATGGGCTCGCGGCCTATCAGCTTGTTGGTGAGGTAATGGCTTACCAAGGCGTCGACGGGTAGCCGGCCTGA
GAGGGTGACCGGCCACACTGGGACTGAGACACGGCCCAGACTCCTACGGGAGGCAGCAGTGGGGAATATTGCACAATGGGCGGAAGCCTGATGCAGCAAC
GCCGCGTGAGGGATGACGGCCTTCGGGTTGTAAACCTCTTTTAGCAAGGAAGAAGCGTGAGTGACGGTACTTGCAGAAAAAGCGCCGGCTAACTACGTGC
CAGCAGCCGCGGTAATACGTAGGGCGCAAGCGTTATCCGGAATTATTGGGCGTAAAGAGCTCGTAGGCGGTTTGTCGCGTCTGCTGTGAAATCCCGAGGC
TCAACCTCGGGTCTGCAGTGGGTACGGGCAGACTAGAGTGCGGTAGGGGAGATTGGAATTCCTGGTGTAGCGGTGGAATGCGCAGATATCAGGAGGAACA
CCGATGGCGAAGGCAGATCTCTGGGCCGTAACTGACGCTGAGGAGCGAAAGGGTGGGGAGCAAACAGGCTTAGATACCCTGGTAGTCCACCCCGTAAACG
TTGGGAACTAGTTGTGGGGTCCTTTCCACGGATTCCGTGACGCAGCTAACGCATTAAGTTCCCCGCCTGGGGAGTACGGCCGCAAGGCTAAAACTCAAAG
GAATTGACGGGGACCCGCACAAGCGGCGGAGCATGCGGATTAATTCGATGCAACGCGAAGAACCTTACCAAGGCTTGACATACACCAGAACACCCCAGAA
ATGGGGGACTCTTTGGACACTGGTGAACAGGTGGTGCATGGTTGTCGTCAGCTCGTGTCGTGAGATGTTGGGTTAAGTCCCGCAACGAGCGCAACCCTCG
TTCTATGTTGCCAGCACGTTATGGTGGGAACTCATGGGATACTGCCGGGGTCAACTCGGAGGAAGGTGGGGATGACGTCAAATCATCATGCCCCTTATGT
CTTGGGCTTCACGCATGCTACAATGGCCGGTACAAAGGGCTGCAATACCGTGAGGTGGAGCGAATCCCAAAAAGCCGGTCCCAGTTCGGATTGAGGTCTG
CAACTCGACCTCATGAAGTCGGAGTCGCTAGTAATCGCAGATCAGCAACGCTGCGGTGAATACGTTCCCGGTTCTTGTACACACCGCCCGTCAAGTCATG
AAAGTCGGTA

>EB102
GGCTCCCTCCACAAGGGTTGGGCCACCGGCTTCGGGCGTTACCGACTTTCGTGACGTGACGGGCGGTGTGTACAAGGCCCGGGAACGTATTCACCGCAGC
GTTGCTGATCTGCGATTACTAGCGACTCCAACTTCATGGGGTCGAGTTGCAGACCCCAATCCGAACTGAGACCGGCTTTTTGGGATTCGCTCCACCTCGC
GGTATCGCAGCCCTTTGTACCGGCCATTGTAGCATGTTTGCAGCCCTAGACATAAGGGGCATGATGATTTGACGTCATCCCCACCTTCCTCCGAGTTGAC
CCCGGCAGTCTCCCATGAGTCCCCACCATTACGTGCTGGCAACATGGAACGAGGGTTGCGCTCGTTGCGGGACTTAACCCAACATCTCACGACACGAGCT
GACGACAACCATGCACCACCTGTGCACGGCCCTTACGGACCCACCATCTCTGGAGGATTTCCGTGCATGTCAAGCCTAGGTAAGGTTCTTCGCGTTGCAT
CGAATTAAGCAACATGCTCCGCCGCTTGTGCGGGCCCCCGTCAATTCCTTTGAGTTTTAGCCTTGCGGCCGTACTCCCCAGGCGGGGCGCTTAATGCGTT
AGCTGCGGCACGGAACTCGTGGAATGAGCCCCACACCTAGCGCCCAACGTTTACGGCGTGGACTACCAGGGTATCTAATCCTGTTCGCTCCCCACGCTTT
CGCTCCTCAGCGTCAGTTGTTGCCCAGAGACCCGCCTTCGCCACCGGTGTTCCTCCTGATATCTGCGCATTTCACCGCTACACCAGGAATTCCAGTCTCC
CCTGCAACACTCTAGTCTGCTCGTATCGACTGCAGGCCTGAGGTTGAGCCTCAGGTTTTCACAGCCGACGCAACAAACCGCCTACGAGCTCTTTACGCCC
AATAATTCCGGACAACGCTTGCACCCTACGTATTACCGCGGCTGCTGGCACGTAGTTGGCCGGTGCTTCTTCTGCAGGTACCGTCACTTTTCGCTTCGTC
CCTGCTGAAAGAGGTTTACAACCCGAAGGCCGTCATCCCCCACGCGGCGTCGCTGCGTCAGGCTTTCGCCCATTGCGCAATATTCCCCACTGCTGCCTCC
CGTAGGAGTCTGGGCCGTGTCTCAGTCCCAGTGTGGCCGGTCACCCTCTCAGGCCGGCTACCCGTCGTCGCCTTGGTAGGCCACTACCCCACCAACAAGC
TGATAGGCCGCGGGCCCATCCTCAGCCGATAAATCTTTCCACCACCAGACCATGCGGTCAGCAGTCATATCCAGTATTAGCCCCAATTTCTTGGAGTTAT
CCCAGAGCTGAGGGCAGGTTGCCCACGTGTTACTCACCCGTTCGCCACTGATCCCCCACCGAAGCGGGTTCACCGTTCGACTGCA

>EB103
CACATGCAGTCGAACGATGAAGCCCAGCTTGCTGGGTGGATTAGTGGCGAACGGGTGAGTAACACGTGAGTAACCTGCCCTTAACTCTGGGATAAGCCTG
GGAAACTGGGTCTAATACCGGATAGGAGCGTCCACCGCATGGTGGGTGTTGGAAAGATTTATCGGTTTTGGATGGACTCGCGGCCTATCAGCTTGTTGGT
GAGGTAATGGCTCACCAAGGCGACGACGGGTAGCCGGCCTGAGAGGGTGACCGGCCACACTGGGACTGAGACACGGCCCAGACTCCTACGGGAGGCAGCA
GTGGGGAATATTGCACAATGGGCGCAAGCCTGATGCAGCGACGCCGCGTGAGGGATGACGGCCTTCGGGTTGTAAACCTCTTTCAGTAGGGAAGAAGCGA
AAGTGACGGTACCTGCAGAAGAAGCACCGGCTAACTACGTGCCAGCAGCCGCGGTAATACGTAGGGTGCGAGCGTTATCCGGAATTATTGGGCGTAAAGA
GCTCGTAGGCGGTTTGTCGCGTCTGTCGTGAAAGTCCGGGGCTTAACCCCGGATCTGCGGTGGGTACGGGCAGACTAGAGTGCAGTAGGGGAGACTGGAA
TTCCTGGTGTAGCGGTGGAATGCGCAGATATCAGGAGGAACACCGATGGCGAAGGCAGGTCTCTGGGCTGTAACTGACGCTGAGGAGCGAAAGCATGGGG
AGCGAACAGGATTAGATACCCTGGTAGTCCATGCCGTAAACGTTGGGCACTAGGTGTGGGGACCATTCCACGGTTTCCGCGCCGCAGCTAACGCATTAAG
TGCCCCGCCTGGGGAGTACGGCCGCAAGGCTAAAACTCAAAGGAATTGACGGGGGCCCGCACAAGCGGCGGAGCATGCGGATTAATTCGATGCAACGCGA
AGAACCTTACCAAGGCTTGACATGTTCTCGATCGCCGTAGAGATACGGTTTCCCCTTTGGGGCGGGTTCACAGGTGGTGCATGGTTGTCGTCAGCTCGTG
TCGTGAGATGTTGGGTTAAGTCCCGCAACGAGCGCAACCCTCGTTCCATGTTGCCAGCACGTAGTGGTGGGGACTCATGGGAGACTGCCGGGGTCAACTC
GGAGGAAGGTGAGGACGACGTCAAATCATCATGCCCCTTATGTCTTGGGCTTCACGCATGCTACAATGGCCGGTACAATGGGTTGCGATACTGTGAGGTG
GAGCTAATCCCAAAAAGCCGGTCTCAGTTCGGATTGGGGTCTGCAACTCGACCCCATGAAGTCGGAGTCGCTAGTAATCGCAGATCAGCAACGCTGCGGT
GAATACGTTCCCGGGCCTTGTACACACCGCCCGTCAAGTCACGAAAGTCGGTAACACCCGAAGCCGGTGGCCTAACCCTTGTGGGGGGAGCCG

>EB104
TGCAAGTCGAACGATGACGGTGGTGCTTGCACCGCCTGATTAGTGGCGAACGGGTGAGTAACACGTGAGCAACCTGCCCTCCACTTCGGGATAACCTCGG
GAAATCGAGGCTAATACCGGATATGAGCACTCATCGCATGGTGGGTGCTGGAAAGATTTATCGGTGGGGGATGGACTCGCGGCCTATCAGTTTGTTGGTG
AGGTGATGGCTTACCAAGACGATGACGGGTAGCCGGCCTGAGAGGGCGACCGGCCACACTGGGACTGAGACACGGCCCAGACTCCTACGGGAGGCAGCAG
TGGGGAATATTGCACAATGGGCGAAAGCCTGATGCAGCGACGCCGCGTGGGGGATGACGGCCTTCGGGTTGTAAACCCCTTTCAGCAGGGAAGAAGCGAG
AGTGACGGTACCTGCAGAAGAAGCGCCGGCTAACTACGTGCCAGCAGCCGCGGTAATACGTAGGGCGCAAGCGTTGTCCGGAATTATTGGGCGTAAAGAG

CTTGTAGGTGGCTTGTCGCGTCTGCCGTGAAAACCCGAGGCTCAACCTCGGGCGTGCGGTGGGTACGGGCAGGCTAGAGTGTGGTAGGGGAGACTGGAAC
TCCTGGTGTAGCGGTGAAATGCGCAGATATCAGGAAGAACACCGATGGCGAAGGCAGGTCTCTGGGCCATTACTGACACTGAGAAGCGAAAGCATGGGTA
GCGAACAGGATTAGATACCCTGGTAGTCCATGCCGTAAACGTTGGGCACTAGGTGTGGGGGACATTCCACGTTTTCCGCGCCGTAGCTAACGCATTAAGT
GCCCCGCCTGGGGAGTACGGCCGCAAGGCTAAAACTCAAAGGAATTGACGGGGGCCCGCACAAGCGGCGGAGCATGCTGATTAATTCGATGCAACGCGAA
GAACCTTACCAAGGCTTGACATGCACTGGACGGCTGCAGAGATGTGGCTTTCTTTGGACTGGTGCACAGGTGGTGCATGGTTGTCGTCAGCTCGTGTCGT
GAGATGTTGGGTTAAGTCCCGCAACGAGCGCAACCCTTGTTCTATGTTGCCAGCACGTGATGGTGGGGACTCATAGGAGACTGCCGGGGTCAACTCGGAG
GAAGGTGGGGACGACGTCAAATCATCATGCCCCTTATGTCTTGGGCTTCAAGCATGCTACAATGGTCGGTACAATGGGTTGCGAAACTGTGAGGTGGAGC
GAATCCCAAAAAGCCGGCCTCAGTTCGGATTGGGGTCTGCAACTCGACCCCATGAAGTCGGAGTCGCTAGTAATCGCAGATCAGCAACGCTGCGGTGAAT
ACGTTCCCGGGCCTTGTACACACCGCCCGTCAAGTCACGAAAGTCGGTAACACCCGAAGCCAGTGGCCCATCCTCGTGAGGGAG

>EB105
CAAGTCGAGCGGACAGATGGGAGCTTGCTCCCTGATGTCAGCGCCGGACGCGTGAGTAACACGTGGGTAACCTGCCTGTAAGACTGGGATAACTCCGGGA
AACCGGGGCTAATACCGGATGCTTGATTGAACCGCATGGTTCAATTATAAAAGGTGGCTTTTAGCTACCACTTACAGATGGACCCGCGGCGCATTAGCTA
GTTGGTGAGGTAACGGCTCACCAAGGCAACGATGCGTAGCCGACCTGAGAGGGTGATCGGCCACACTGGGACTGAGACACGGCCCAGACTCCTACGGGAG
GCAGCAGTAGGGAATCTTCCGCAATGGACGAAAGTCTGACGGAGCAACGCCGCGTGAGTGATGAAGGTTTTCGGATCGTAAAACTCTGTTGTTAGGGAAG
AACAAGTACCGTTCGAATAGGGCGGTACCTTGACGGTACCTAACCAGAAAGCCACGGCTAACTACGTGCCAGCAGCCGCGGTAATACGTAGGTGGCAAGC
GTTGTCCGGAATTATTGGGCGTAAAGCGCGCGCAGGCGGTTTCTTAAGTCTGATGTGAAAGCCCCCGGCTCAACCGGGGAGGGTCATTGGAAACTGGGGA
ACTTGAGTGCAGAAGAGGAGAGTGGAATTCCACGTGTAGCGGTGAAATGCGTAGAGATGTGGAGGAACACCAGTGGCGAAGGCGACTCTCTGGTCTGTAA
CTGACGCTGAGGCGCGAAAGCGTGGGGAGCGAACAGGATTAGATACCCTGGTAGTCCACGCCGTAAACGATGAGTGCTAAGTGTTAGAGGGTTTCCGCCC
TTTAGTGCTGCAGCAAACGCATTAAGCACTCCGCCTGGGGAGTACGGTCGCAAGACTGAAACTCAAAGGAATTGACGGGGGCCCGCACAAGCGGTGGAGC
ATGTGGTTTAATTCGAAGCAACGCGAAGAACCTTACCAGGTCTTGACATCCTCTGACAACCCTAGAGATAGGGCTTCCCCTTCGGGGGCAGAGTGACAGG
TGGTGCATGGTTGTCGTCAGCTCGTGTCGTGAGATGTTGGGTTAAGTCCCGCAACGAGCGCAACCCTTGATCTTAGTTGCCAGCATTCAGTTGGGCACTC
TAAGGTGACTGCCGGTGACAAACCGGAGGAAGGTGGGGATGACGTCAAATCATCATGCCCCTTATGACCTGGGCTACACACGTGCTACAATGGGCAGAA
CAAAGGGCAGCGAAGCCGCGAGGCTAAGCCAATCCCACAAATCTGTTCTCAGTTCGGATCGCAGTCTGCAACTCGACTGCGTGAAGCTGGAATCGCTAGT
AATCGCGGATCAGCATGCCGCGGTGAATACGTTCCCGGGCCTTGTACACACCGCCCGTCACACCACGAGAGTTTGTAACACCCGAAATTCGGTGAGGTA
ACCTTTTGGAGCCAGCCCCCCAAAGTGGGACAGATGATTGGGGTGAAT

>EB106
GGTGGATCAGTGGCGAACGGGTGAGTAACACGTGAGCAACCTGCCCTGGACTCTGGGATAAGCGCTGGAAACGGCGTCTAATACTGGATATGAGACGTGA
TCGCATGGTCGTGTTTGGAAAGATTTTTCGGTCTGGGATGGGCTCGCGGCCTATCAGCTTGTTGGTGAGGTAATGGCTCACCAAGGCGTCGACGGGTAGC
CGGCCTGAGAGGGTGACCGGCCACACTGGGACTGAGACACGGCCCAGACTCCTACGGGAGGCAGCAGTGGGGAATATTGCACAATGGGCGAAAGCCTGAT
GCAGCAACGCCGCGTGAGGGATGACGGCCTTCGGGTTGTAAACCTCTTTTAGCAGGGAAGAAGCGAAAGTGACGGTACCTGCAGAAAAAGCGCCGGCTAA
CTACGTGCCAGCAGCCGCGGTAATACGTAGGGCGCAAGCGTTATCCGGAATTATTGGGCGTAAAGAGCTCGTAGGCGGTTTGTCGCGTCTGCTGTGAAAT
CCCGAGGCTCAACCTCGGGCCTGCAGTGGGTACGGGCAGACTAGAGTGCGGTAGGGGAGATTGGAATTCCTGGTGTAGCGGTGGAATGCGCAGATATCAG
GAGGAACACCGATGGCGAAGGCAGATCTCTGGGCCGTAACTGACGCTGAGGAGCGAAAGGGTGGGGAGCAAACAGGCTTAGATACCCTGGTAGTCCACCC
CGTAAACGTTGGGAACTAGTTGTGGGGACCATTCCACGGTTTCCGTGACGCAGCTAACGCATTAAGTTCCCCGCCTGGGGAGTACGGCCGCAAGGCTAAA
ACTCAAAGGAATTGACGGGGACCCGCACAAGCGGCGGAGCATGCGGATTAATTCGATGCAACGCGAAGAACCTTACCAAGGCTTGACATATACGAGAACG
GGCCAGAAATGGTCAACTCTTTGGACACTCGTAAACAGGTGGTGCATGGTTGTCGTCAGCTCGTGTCGTGAGATGTTGGGTTAAGTCCCGCAACGAGCGC
AACCCTCGTTCTATGTTGCCAGCACGTAATGGTGGGAACTCATGGGATACTGCCGGGGTCAACTCGGAGGAAGGTGGGGATGACGTCAAATCATCATGCC
CCTTATGTCTTGGGCTTCACGCATGCTACAATGGCCGGTACAAAGGGCTGCAATACCGTGAGGTGGAGCGAATCCCAAAAAGCCGGTCCCAGTTCGGATT
GAGGTCTGCAACTCGACCTCATGAAGTCGGAGTCGCTAGTAATC

>EB107
TCGACGGACCCTTCGGGGTTAGTGGCGGACGGGTGAGTAACACGTGGGAACGTGCCTTTAGGTTCGGAATAGCTCCTGGAAACGGGTGGTAATGCCGAAT
GTGCCCTTCGGGGGAAAGATTTATCGCCTTTAGAGCGGCCCGCGTCTGATTAGCTAGTTGGTGAGGTAATGGCTCACCAAGGCGACGATCAGTAGCTGGT
CTGAGAGGATGACCAGCCACATTGGGACTGAGACACGGCCCAAACTCCTACGGGAGGCAGCAGTGGGGAATCTTGCGCAATGGGCGAAAGCCTGACGCAG
CCATGCCGCGTGAATGATGAAGGTCTTAGGATTGTAAAATTCTTTCACCGGGGACGATAATGACGGTACCCGGAGAAGAAGCCCCGGCTAACTTCGTGCC
AGCAGCCGCGGTAATACGAAGGGGGCTAGCGTTGCTCGGAATTACTGGGCGTAAAGGGCGCGTAGGCGGACATTTAAGTCAGGGGTGAAATCCCAGAGCT
CAACTCTGGAACTGCCTTTGATACTGGGTGTCTTGAGTGTGAGAGAGGTATGTGGAACTCCGAGTGTAGAGGTGAAATTCGTAGATATTCGGAAGAACAC
CAGTGGCGAAGGCGACATACTGGCTCATTACTGACGCTGAGGCGCGAAAGCGTGGGGAGCAAACAGGATTAGATACCCTGGTAGTCCACGCCGTAAACGA
TGATTGCTAGTTGTCGGGCTGCATGCAGTTCCGTGACGCAGCTAACGCATTAAGCAATCCGCCTGGGGAGTACGGTCGCAAGATTAAAACTCAAAGGAAT
TGACGGGGGGCCCGCACAAGCGGTGGAGCATGTGGTTTAATTCGAAGCAACGCGCAGAACCTTACCACCTTTTGACATGCCTGGGACCGCCAGAGAGATC
TGGCTTTCTCTTCGGAGACTAGGACACAGGTGCTGCATGGCTGTCGTCAGCTCGTGTCGTGAGATGTTGGGTTAAGTCCCCGCAACGAGCGCAACCCTCG
CCATTAGTTGGCCATCATTTAGTTGGGAACTCTAATGGGACAGCCGGTGCTAAGCCGGAGGAAGGTGGGGATGACGTCAAGTCCTCATGGCCCTTACAGG
GTGGGCTACACACGTGCTACAATGGCGACTACAGAGGGTTAATCCTTAAAAGTCGTCTCAGTTCGGATTGTCCTCTGCAACTCGAGGGCATGAAGTGGGA
ATCGCTAGTAATCGCGGATCAGCATGCCGCGGTGAATACGTTCCCGGGCCTTGTACACACCGCCCGTCACACCATGGGAGTTGGTTCTACCCGAAGGCGA
TGCGCTAACCCGCAAGGGAGGCA

>EB108
GTCGAACGAAGTCTTCGGACTTAGTGGCGGACGGGTGAGTAACACGTGGGAACGTGCCTTTAGGTTCGGAATAACTCAGGGAAACTTGTGCTAATACCGA
ATGTGCCCTTCGGGGGAAAGATTTATCGCCTTTAGAGCGGCCCGCGTCTGATTAGCTAGTTGGTGAGGTAAAGGCTCACCAAGGCTACGATCAGTAGCTG
GTCTGAGAGGATGACCAGCCACATTGGGACTGAGACACGGCCCAAACTCCTACGGGAGGCAGCAGTGGGGAATCTTGCGCAATGGGCGAAAGCCTGACGC
AGCCATGCCGCGTGGATGATGAAGGTCTTAGGATTGTAAAATCCTTTCACCGGTGAAGATAATGACTGTAGCCGGAGAAGAAGCCCCGGCTAACTTCGTG
CCAGCAGCCGCGGTAATACGAAGGGGGCTAGCGTTGCTCGGAATTACTGGGCGTAAAGGGAGCGTAGGCGGACATTTAAGTCAGGGGTGAAATCCCGAGG
CTCAACCTCGGAACTGCCTTTGATACTGGGTGTCTTGAGTGTGAGAGAGGTATGTGGAACTCCGAGTGTAGAGGTGAAATTCGTAGATATTCGGAAGAAC
ACCAGTGGCGAAGGCGACATACTGGCTCATTACTGACGCTGAGGCTCGAAAGCGTGGGGAGCAAACAGGATTAGATACCCTGGTAGTCCACGCCGTAAAC
GATGATTGCTAGTTGTCGGGAAGCTTGCTTTTCGGTGACGCAGCTAACGCATTAAGCAATCCGCCTGGGGAGTACGGTCGCAAGATTAAAACTCAAAGGA
ATTGACGGGGGCCCGCACAAGCGGTGGAGCATGTGGTTTAATTCGAAGCAACGCGCAGAACCTTACCACCTTTTGACATGCCCGGACCGCCACAGAGATG
TGGCTTTCCCTTCGGGGACTGGGACACAGGTGCTGCATGGCTGTCGTCAGCTCGTGTCGTGAGATGTTGGGTTAAGTCCCGCAACGAGCGCAACCCTCGC
CATTAGTTGCCATCATTCAGTTGGGAACTCTAATGGGACTGCCGGTGCTAAGCCGGAGGAAGGTGGGGATGACGTCAAGTCCTCATGGCCCTTACAGGGT
GGGCTACACACGTGCTACAATGGCGACTACAGAGGGTTAATCCTTAAAAGTCGTCTCAGTTCGGATTGTCCTCTGCAACTCGAGGGCATGAAGTTGGAAT
CGCTAGTAATCGCGGATCAGCATGCCGCGGTGAATACGTTCCCGGGCCTTGTACACACCGCCCGTCACACCATGGGAGTTGGTTCTACCCGAAGGTGGTG
CGCTAACCGCAAGGAGGCAGCCAA

>EB109
CGACTGCCTCCTTACGGTTAGCGCATCGCCTTCGGGTAGAACCAACTCCCATGGTGTGACGGGCGGTGTGTACAAGGCCCGGGAACGTATTCACCGCGGC
ATGCTGATCCGCGATTACTAGCGATTCCAACTTCATGCCCTCGAGTTGCAGAGGACAATCCGAACTGAGACGACTTTTAAGGATTAACCCTCTGTAGTCG
CCATTGTAGCACGTGTGTAGCCCACCCTGTAAGGGCCATGAGGACTTGACGTCATCCCCACCTTCCTCCGGCTTAGCACCGGCAGTCCCATTAGAGTTCC

CAACTAAATGATGGCAACTAATGGCGAGGGTTGCGCTCGTTGCGGGACTTAACCCAACATCTCACGACACGAGCTGACGACAGCCATGCAGCACCTGTGT
CCTAGTCTCCGAAGAGAAAGCCACATCTCTGTGGCGGTCCAGGCATGTCAAAAAGGTGGTAAGGTTCTGCGCGTTGCTTCGAATTAAACCACATGCTCCA
CCGCTTGTGCGGGCCCCCGTCAATTCCTTTGAGTTTTAATCTTGCGACCGTACTCCCCAGGCGGATTGCTTAATGCGTTAGCTGCGTCACCGAGAGGTAA
ACCCCCCAACAACTAGCAATCATCGTTTACGGCGTGGACTACCAGGGTATCTAATCCTGTTTGCTCCCCACGCTTTCGAGCCTCAGCGTCAGTAATGATC
CAGTATGTCGCCTTCGCCACTGGTGTTCTTCCGAATATCTACGAATTTCACCTCTACACTCGGAGTTCCACATACCTCTATCATACTCAAGACACCCAGT
ATCAAAGGCAGTTCCAGAGTTGAGCTCTGGGATTTCACCCCTGACTTAAATGTCCGCCTACGCTCCCTTTACGCCCAGTAATTCCGAGCAACGCTAGCCC
CCCTTCGTATTACCGCGGCTGCTGGCACGAAGTTAGCCGGGGCTTCTTCTCCGGGTACCGTCATTATCGTCCCCGGTGAAAGAATTTTACAATCCTAAGA
CCTTCATCATTCACGCGGCATGGCTGCGTCAGGCTTTCGCCCATTGCGCAAGATTCCCCACTGCTGCCTCCCGTAGGAGTTTGGGCCGTGTCTCAGTCCC
AATGTGGCTGGTCATCCTCTCAGACCAGCTACTGATCGTAGCCTTGGTGAGCCTTTACCTCACCAACTAGCTAATCAGACGCGGGCCGCTCTAAAGGCGA
TAAATCTTTCCCCCGAAGGGCACATTCGGCATTACCACCCGTTTCCAGGAGCTATTCCGAACCTAAAGGCACGTTCCCACGTGTTACTCACCCGTCCGCC
ACTAACCCCGAAGGGTCCGTTCGACTGCA

>EB110
GACGTGATGGGCGGTGTGTACAATGTACGGGAACGTATTCACCGCGGCATGCTGATCCGCGATTACTAACGATTCCAGCTTCATGTAGGCGAGTTGCAGC
CTACAATCCGAACTGAGAACGGTTTTATGGGATTGGCTAAACCTCGCGGTCTTGCAGCCCTTTGTACCGTCCATTGTAGCACGTGTGTAGCCCAGGTCAT
AAGGGGCATGATGATTTGACGTCATCCCCACCTTCCTCCGGTTTGTCACCGGCAGTCATCTTAGAGTGCCCAACTGAATGCTGGCAACTAAGATCAAGGG
TTGCGCTCGTTGCGGGACTTAACCCAACATCTCACGACACGAGCTGACGACAACCATGCACCACCTGTCACTCTGTCCCCCGAAGGGGAAAGCCCTATCT
CTAGGGTTGTCAGAGGATGTCAAGACCTGGTAAGGTTCTTCGCGTTGCTTCGAATTAAACCACATGCTCCACCGCTTGTGCGGGCCCCCGTCAATTCCTT
TGAGTTTCAGTCTTGCGACCGTACTCCCCAGGCGGAGTGCTTAATGCGTTAGCTGCAGCACTAAAGGGCGGAAACCCTCTAACACTTAGCACTCATCGTT
TACGGCGTGGACTACCAGGGTATCTAATCCTGTTTGCTCCCCACGCTTTCGCGCCTCAGTGTCAGTTACAGACCAGAAAGTCGCCTTCGCCACTGGTGTT
CCTCCAAATATCTACGCATTTCACCGCTACACTTGGAATTCCACTTTCCTCTTCTGCACTCAAGTTCCCCAGTTTCCAATGACCCTCCACGGTTGAGCCG
TGGGCTTTCACATCAGACTTAAGAAACCACCTGCGCGCGCTTTACGCCCAATAATTCCGGACAACGCTTGCCACCTACGTATTACCGCGGCTGCTGGCAC
GTAGTTAGCCGTGGCTTTCTGGTTAGGTACCGTCAAGGTGCCGCCCTATTCGAACGGCACTTGTTCTTCCCTAACAACAGAGTTTTACGATCCGAAAACC
TTCTTCACTCACGCGGCGTTGCTCCGTCAGACTTTCGTCCATTGCGGAAGANTTCCCTACTGCTGCCTCCCGTAGGAGTCTGGGCCGTGTCTCAGTCCCA
GTGTGGCCGATCACCCTCTCAGGTCGGCTACGCATCGTCGCCTTGGTGAGCCGTTACCTCACCAACTAGCTAATGCGCCGCGGGTCCATCTGTAAGTGAT
AGCCGAAGCCACCTTTCAACCTTCCCCCATGCGGGGGTAGGTGTTATCCGGTATTAGCCCCGGTTTCCCGGAGTTATCCCAGTCTTACAGGCAGGTTACC
CACGTGTACTCACCCGTCC

>EB111
CAGTCGAGCGGATCGATGGGAGCTTGCTCCCTGAGATCAGCGGCGGACGGGTGAGTAACACGTGGGTAACCTGCCTGTAAGACTGGGATAACTCCGGGAA
ACCGGGGCTAATACCGGATAACACCTACCCCCGCATGGGGGAAGGTTGAAAGGTGGCTTCGGCTATCACTTACAGATGGACCCGCGGCGCATTAGCTAGT
TGGTGAGGTAACGGCTCACCAAGGCGACGATGCGTAGCCGACCTGAGAGGGTGATCGGCCACACTGGGACTGAGACACGGCCCAGACTCCTACGGGAGGC
AGCAGTAGGGAATCTTCCGCAATGGACGAAAGTCTGACGGAGCAACGCCGCGTGAGTGAAGAAGGTTTTCGGATCGTAAAACTCTGTTGTTAGGGAAGAA
CAAGTGCCGTTCGAATAGGGCGGCACCTTGACGGTACCTAACCAGAAAGCCACGGCTAACTACGTGCCAGCAGCCGCGGTAATACGTAGGTGGCAAGCGT
TGTCCGGAATTATTGGGCGTAAAGCGCGCGCAGGTGGTTTCTTAAGTCTGATGTGAAAGCCCACGGCTCAACCGTGGAGGGTCATTGGAAACTGGGGAAC
TTGAGTGCAGAAGAGGAAAGTGGAATTCCAAGTGTAGCGGTGAAATGCGTAGATATTTGGAGGAACACCAGTGGCGAAGGCGACTTTCTGGTCTGTAACT
GACACTGAGGCGCGAAAGCGTGGGGAGCAAACAGGATTAGATACCCTGGTAGTCCACGCCGTAAACGATGAGTGCTAAGTGTTAGAGGGTTTCCGCCCTT
TAGTGCTGCAGCTAACGCATTAAGCACTCCGCCTGGGGAGTACGGTCGCAAGACTGAAACTCAAAGGAATTGACGGGGGCCCGCACAAGCGGTGGAGCAT
GTGGTTTAATTCGAAGCAACGCGAAGAACCTTACCAGGTCTTGACATCCTCTGACAACCCTAGAGATAGGGCTTTCCCCTTCGGGGGACAGAGTGACAGG
TGGTGCATGGTTGTCGTCAGCTCGTGTCGTGAGATGTTGGGTTAAGTCCCGCAACGAGCGCAACCCTTGATCTTAGTTGCCAGCATTCAGTTGGGCACTC
TAAGATGACTGCCGGTGACAAACCGGAGGAAGGTGGGGATGACGTCAAATCATCATGCCCCTTATGACCTGGGCTACACACGTGCTACAATGGACGGTAC
AAAGGGCTGCAAGACCGCGAGGTTTAGCCAATCCCATAAAACCGTTCTCAGTTCGGATTGTAGGCTGCAACTCGCCTACATGAAGCTGGAATCGCTAGTA
ATCGCGGATCAGCATGCCGCGGTGAATACGTTCCCGGGCCTTGTACACACCGCCCGTCACACCACGAGAGTTTGTAACACCCGAAGTCGGTGAGGTAACC
T

>EB112
AGCGCCCTCCCGAAGGTTAAGCTACCTACTTCTTTTGCAACCCACTCCCATGGTGTGACGGGCGGTGTGTACAAGGCCCGGGAACGTATTCACCGTAGCA
TTCTGATCTACGATTACTAGCGATTCCGACTTCACGGAGTCGAGTTGCAGACTCCGATCCGGACTACGACGCACTTTATGAGGTCCGCTTGCTCTCGCGA
GGTCGCTTCTCTTTGTATGCGCCATTGTAGCACGTGTGTAGCCCTACTCGTAAGGGCCATGATGACTTGACGTCATCCCCACCTTCCTCCAGTTTATCAC
TGGCAGTCTCCTTTGAGTTCCCGGCCGAACCGCTGGCAACAAAGGATAAGGGTTGCGCTCGTTGCGGGACTTAACCCAACATTTCACAACACGAGCTGAC
GACAGCCATGCAGCACCTGTCTCAGAGTTCCCGAAGGCACTAAGCTATCTCTGGCGAATTCTCTGGATGTCAAGAGTAGGTAAGGTTCTTCGCGTTGCAT
CGAATTAAACCACATGCTCCACCGCTTGTGCGGGCCCCCGTCAATTCATTTGAGTTTTAACCTTGCGGCCGTACTCCCCAGGCGGTCGACTTAACGCGTT
AGCTCCGGAAGCCACTCCTCAAGGGAACAACCTCCAAGTCGACATCGTTTACGGCGTGGACTACCAGGGTATCTAATCCTGTTTGCTCCCCACGCTTTCG
CACCTGAGCGTCAGTCTTTGTCCAGGGGGCCGCCTTCGCCACCGGTATTCCTCCAGATCTCTACGCATTTCACCGCTACACCTGGAATTCTACCCCCCTC
TACAAGACTCTAGCCTGCCAGTTTCGAATGCAGTTCCCAGGTTGAGCCCGGGGATTTCACATCCGACTTGACAGACCGCCTGCGTGCGCTTTACGCCCAG
TAATTCCGATTAACGCTTGCACCCTCCGTATTACCGCGGCTGCTGGCACGGAGTTAGCCGGTGCTTCTTCTGCGAGTAACGTCAATCACTGCGGTTATTA
ACCACAATGCCTTCCTCCTCGCTGAAAGTACTTTACAACCCGAAGGCCTTCTTCATACACGCGGCATGGCTGCATCAGGCTTGCGCCCATTGTGCAATAT
TCCCCACTGCTGCCTCCCGTAGGAGTCTGGACCGTGTCTCAGTTCCAGTGTGGCTGGTCATCCTCTCAGACCAGCTAGGGATCGTCGCCTAGGTGAGCCA
TTACCTCACCTACTAGCTAATCCCATCTGGGCACATCTGATGGCAAGAGGCCCGAAGGTCCCCCTCTTTGGTCCGAAGACGTTATGCGGTATTAGCTACC
GTTTCCAGTAGTTATCCCCCTCCATCAGGCAGTTTCCCAGACATTACTCACCCGTCCGCCGCTCGTCACCCAGGAGCAAGCTCCCTGTGCTACCGCTCGA
CTTGCA

>EB113
TAGCGCCCTCCCGAAGGTTAAGCTACCTACTTCTTTTGCAACCCACTCCCATGGTGTGACGGGCGGTGTGTACAAGGCCCGGGAACGTATTCACCGTAGC
ATTCTGATCTACGATTACTAGCGATTCCGACTTCATGGAGTCGAGTTGCAGACTCCAATCCGGACTACGACGCACTTTATGAGGTCCGCTTGCTCTCGCG
AGGTCGCTTCTCTTTGTATGCGCCATTGTAGCACGTGTGTAGCCCTACTCGTAAGGGCCATGATGACTTGACGTCATCCCCACCTTCCTCCAGTTTATCA
CTGGCAGTCTCCTTTGAGTTCCCGGCCGAACCGCTGGCAACAAAGGATAAGGGTTGCGCTCGTTGCGGGACTTAACCCAACATTTCACAACACGAGCTGA
CGACAGCCATGCAGCACCTGTCTCAGAGTTCCCGAAGGCACCAATCCATCTCTGGAAAGTTCTCTGGATGTCAAGAGTAGGTAAGGTTCTTCGCGTTGCA
TCGAATTAAACCACATGCTCCACCGCTTGTGCGGGCCCCCGTCAATTCATTTGAGTTTTAACCTTGCGGCCGTACTCCCCAGGCGGTCGACTTAACGCGT
TAGCTCCGGAAGCCACGCCTCAAGGGCACAACCTCCAAGTCGACATCGTTTACGGCGTGGACTACCAGGGTATCTAATCCTGTTTGCTCCCCACGCTTTC
GCACCTGAGCGTCAGTCTTTGTCCAGGGGGCCGCCTTCGCCACCGGTATTCCTCCAGATCTCTACGCATTTCACCGCTACACCTGGAATTCTACCCCCCT
CTACAAGACTCAAGCTTGCCAGTTTCGAATGCAGTTCCCAGGTTGAGCCCGGGGATTTCACATCCCGACTTAACAAACCGCCTGCGTGCGCTTTACGCCC
AGTAATTCCGATTAACGCTTGCACCCTCCGTATTACCGCGGCTGCTGGCACGGAGTTAGCCGGTGCTTCTTCTGCGAGTAACGTCAATCGCCAAGGTTAT
TAACCTTAACGCCTTCCTCCTCGCTGAAAGTACTTTACAACCCGAAGGCCTTCTTCATACACGCGGCATGGCTGCATCAGGCTTGCGCCCATTGTGCAAT
ATTCCCCACTGCTGCCTCCCGTAGGAGTCTGGACCGTGTCTCAGTTCCAGTGTGGCTGGTCATCCTCTCAGACCAGCTAGGGATCGTCGCCTAGGTGAGC
CATTACCCCACCTACTAGCTAATCCCATCTGGGTTCATCTGATGGCAAGAGGCCCGAAGGTCCCCCTCTTTGGTCTTGCGACGTTATGCGGTATTAGCTA
CCGTTTCCAGTAGTTATCCCCCTCCATCAGGCAGATCCCCAGACATTACTCACCCGTCCGCCGCTCGTCACCCAGAGAGCAAGCTCTCCCGTGCTACCGC
TCGACTTGCA

>EB114
AAGGGTTGGGCCACCGGCTTCGGGTGTTACCGACTTTCGTGACTTGACGGGCGGTGTGTACAAGGCCCGGGAACGTATTCACCGCAGCGTTGCTGATCTG
CGATTACTAGCGACTCCGACTTCATGGGGTCGAGTTGCAGACCCCAATCCGAACTGAGACCGGCTTTTTGGGATTCGCTCCACCTCGCGGTATCGCAGCC
CTTTGTACCGGCCATTGTAGCATGCGTGAAGCCCAAGACATAAGGGGCATGATGATTTGACGTCATCCCCACCTTCCTCCGAGTTGACCCCGGCAGTCTC
CCATGAGTCCCCGGCATAACCCGCTGGCAACATGGACGAGGGTTGCGCTCGTTGCGGGACTTAACCCAACATCTCACGACACGAGCTGACGACAACCAT
GCACCACCTGTACACCGACCTTACGGGGAGCACATCTCTGCACTTTTCCGGTGTATGTCAAGCCTTGGTAAGGTTCTTCGCGTTGCATCGAATTAATCCG
CATGCTCCGCCGCTTGTGCGGGCCCCCGTCAATTTCTTTGAGTTTTAGCCTTGCGGCCGTACTCCCCAGGCGGGGCACTTAATGCGTTTGCTGCGGCACA
GAGCTCGTGGAATGAGCCCCACACCTAGTGCCCAACGTTTACGGCATGGACTACCAGGGTATCTAATCCTGTTCGCTCCCCATGCTTTCGCTCCTCAGCG
TCAGTTGCGGCCCAGTGACCTGCCTTCGCCATCGGTGTTCCTCCTGATATCTGCGCATTCCACCGCTACACCAGGAATTCCAGTCACCCCTACCGCACTC
TAGTCTGCCCGTACCCACTGCAAGCCCCAGGTTGAGCCTGAGGTTTTCACAGCAGACGCGACAAACCGCCTACGAGCTCTTTACGCCCAATAATTCCGGA
CAACGCTTGCGCCCTACGTATTACCGCGGCTGCTGGCACGTAGTTAGCCGGCGCTTCTTCTGCAGGTACCGTCACTTGCGCTTCTTCCCTGCTGAAAGAG
GTTTACAACCCGAAGGCCGTCATCCCTCACGCGGCGTCGCTGCATCAGGCTTGCGCCCATTGTGCAATATTCCCCACTGCTGCCTCCCGTAGGAGTCTGG
GCCGTGTCTCAGTCCCAGTGTGGCCGGTCGCCCTCTCAGGCCGGCTACCCGTCGTCGCCTTGGTAGGCCGTTACCCCACCAACAAGCTGATAGGCCGCGA
GCCCATCCCTGACCGAAAAACTTTCCAGACGCAACAGATGCCTGTACGTCTCATATCCGGTATTAGCGACCGTTTCCGGTCGTTATCCCAGAGTCAGGGG
CAGGTTGCTCACGTGTTACTCACCCGTTCGCCACTGATCAGACCAGCAAGCTGGTCGTCACCGTTCGA

>EB115
GATGTGATGGGCGGTGTGTACAATGCCCGGGAACGTAGTCACCGCGGCATGCTGATCCGCGATTACTAACGATTCCAGCTTCATGTAGGCGAGTTGCAGC
CTACAATCCGAACTGAGAACGGTTTTATGGGATTGGCTAAACCTCGCGGTCTTGCAGCCCTTTGTACCGTCCATTGTAGCACGTGTGTAGCCCAGGTCAT
AAGGGGCATGATGATTTGACGTCATCCCCACCTTCCTCCGGTTTGTCACCGGCAGTCATCTTAGAGTGCCCAACTGAATGCTGGCAACTAAGATCAAGGG
TTGCGCTCGTTGCGGGACTTAACCCAACATCTCACGACACGAGCTGACGACAACCATGCACCACCTGTCACTCTGTCCCCCGAAGGGGAAAGCCCTATCT
CTAGGGTTGTCAGAGGATGTCAAGACCTGGTAAGGTTCTTCGCGTTGCTTCGAATTAAACCACATGCTCCACCGCTTGTGCGGGCCCCCGTCAATTCCTT
TGAGTTTCAGTCTTGCGACCGTACTCCCCAGGCGGAGTGCTTAATGCGTTAGCTGCAGCACTAAAGGGCGGAAACCCTCTAACACTTAGCACTCATCGTT
TACGGCGTGGACTACCAGGGTATCTAATCCTGTTTGCTCCCCACGCTTTCGCGCCTCAGTGTCAGTTACAGACCAGAAAGTCGCCTTCGCCACTGGTGTT
CCTCCAAATATCTACGCATTTCACCGCTACACTTGGAATTCCACTTTCCTCTTCTGCACTCAAGTTCCCCAGTTTCCAATGACCCTCCACGGTTGAGCCG
TGGGCTTTCACATCAGACTTAAGAAACCACCTGCGCGCGCTTTACGCCCAATAATTCCGGACAACGCTTGCCACCTACGTATTACCGCGGCTGCTGGCAC
GTAGTTAGCCGTGGCTTTCTGGTTAGGTACCGTCAAGGCGCCGCCCTATTCGAACGGCACTTGTTCTTCCCTAACAACAGAGTTTTACGATCCGAAAACC
TTCTTCACTCACGCGGCGTTGCTCCGTCAGACTTTCGTCCATTGCGGAAGATTCCCTACTGCTGCCTCCCGTAGGAGTCTGGGCCGTGTCTCAGTCCCAG
TGTGGCCGATCACCCTCTCAGGTCGGCTACGCATCGTCGCCTTGGTGAGCCATTACCTCACCAACTAGCTAATTGCGCCGCGGGTCCATCTGTAAGTGAT
AGCCGAAGCCACCTTTCAACCTTCCCCCATGCGGGGGTAGGTGTTATCCGGTATTAGCCCCGGTTTCCCGGAGTTATCCCAGTAATACAGGCAGGTTCCC
CACGTGAATTCATGAGTCATAATCAAACTTTACGGATACCGTGTTACGACTTACGAGCCATAATCAAACT

>EB116
CCTCTTTCAGGGGGAGATGGATAGAAAAATGTTTTTGGGTTTTCAATTAAAAGATGGGCCCCGCGGTGCATTAAGTTAGTTGGGGGAGGAAAGGGTTCCA
CAAAGGCAAAGGATGCATAGCCGACCTGAAGAGGGTGATCGGCCCCAATGGGGATTGAAACACGGCCCAAGATTCCTACGGGAGGCAGCAGTAGGGAATC
TTCCGCAATGGACGAAAGTCTGACGGAGCAACGCCGCGTGAGTGATGAAGGCTTTCGGGTCGTAAAACTCTGTTGTTAGGGAAGAACAAGTACGAGAGTA
ACTGCTCGTACCTTGACGGTACCTAACCAGAAAGCCACGGCTAACTACGTGCCAGCAGCCGCGGTAATACGTAGGTGGCAAGCGTTATCCGGAATTATTG
GGCGTAAAGCGCGCGCAGGCGGTTTCTTAAGTCTGATGTGAAAGCCCACGGCTCAACCGTGGAGGGTCATTGGAAACTGGGGAACTTGAGTGCAGAAGAG
AAAAGCGGAATTCCACGTGTAGCGGTGAAATGCGTAGAGATGTGGAGGAACACCAGTGGCGAAGGCGGCTTTTTGGTCTGTAACTGACGCTGAGGCGCGA
AAGCGTGGGGAGCAAACAGGATTAGATACCCTGGTAGTCCACGCCGTAAACGATGAGTGCTAAGTGTTAGAGGGTTTCCGCCCTTTAGTGCTGCAGCTAA
CGCATTAAGCACTCCGCCTGGGGAGTACGGTCGCAAGACTGAAACTCAAAGGAATTGACGGGGGCCCGCACAAGCGGTGGAGCATGTGGTTTAATTCGAA
GCAACGCGAAGAACCTTACCAGGTCTTGACATCCTCTGACAACTCTAGAGATAGAGCGTTCCCCTTCGGGGGACAGAGTGACAGGTGGTGCATGGTTGTC
GTCAGCTCGTGTCGTGAGATGTTGGGTTAAGTCCCGCAACGAGCGCAACCCTTGATCTTAGTTGCCAGCATTTAGTTGGGCACTCTAAGGTGACTGCCGG
TGACAAACCGGAGGAAGGTGGGGATGACGTCAAATCATCATGCCCCTTATGACCTGGGCTACACACGTGCTACAATGGATGGTACAAAGGGCTGCAAGAC
CGCGAGGTCAAGCCAATCCCATAAAACCATTCTCAGTTCGGATTGTAGGCTGCAACTGGCATACATGAAGCTGGAATCGTTGGTAATGGTCGATCAGCAG
GACGCGGTGACTACGTTGCCGTACTTTGTGCTCCTCGCTCGTTTCGTCGTGACACTTTACTCTCGCGTTTGTCCCTGTCGCTCGGTCTCGTCCCGCCGTC
ACCGTGCTCTGCGCCGCTAAGATATTAGTTCCGCCCCC

>EB117
GGCAGCTCCTGTTACGGTCACCGACTTCAGGTACCCCAAACTTCCATGGCTTGACGGGCGGTGTGTACAAGGCCCGGGAACGTATTCACCGCATCATGGC
TGATATGCGATTACTAGCGATTCCAGCTTCATAGAGTCGAGTTGCAGACTCCAATCCGAACTGAGACCGGCTTTCGAGATTCGCATCCTATCGCTAGGTA
GCTGCCCTCTGTACCGGCCATTGTATTACGTGTGTGGCCCAAGGCGTAAGGGCCGTGATGATTTGACGTCATCCCCACCTTCCTCTCTACTTGCGTAGGC
AGTCTCACTAGAGTCCCCAACTGAATGATGGCAACTAGTGACAGGGGTTGCGCTCGTTGCAGGACTTAACCTAACACCTCACGGCACGAGCTGACGACAA
CCATGCAGCACCTTGAAAATTGTCCGAAGAAAAGTCTATTTCTAAACCTGTCAATTCCCATTTAAGCCTTGGTAAGGTTCCTCGCGTATCATCGAATTAA
ACCACATAATCCACCGCTTGTGCGGGCCCCCGTCAATTCCTTTGAGTTTCATTCTTGCGAACGTACTCCCCAGGTGGCTAACTTATCACTTTCGCTTAGT
CTCTGAACCCTAAAGCCCAAAAACGAGTTAGCATCGTTTACGGCGTGGACTACCAGGGTATCTAATCCTGTTCGCTCCCCACGCTTTCGTCCATCAGCGT
CAGTTAAAACATAGTGACCTGCCTTCGCAATTGGTGTTCTAAGTAATATCTATGCATTTCACCGCTACACTACTTATTCCAGCCACTTCTACTTTACTCA
AGACCTGCAGTATCAATGGCAGTTTCATAGTTAAGCTATGAGATTTCACCACTGACTTACAGATCCGCCTACGGACCCTTTAAACCCAATAAATCCGGAT
AACGCTTGCACCCTCCGTATTACCGCGGCTGCTGGCACGGAGTTAGCCGGTGCTTATTCGTATAGTACCTTCAGCTTTCCACACGTGGAAAGGTTTATCC
CTATACAAAAGAAGTTTACAACCCATAGGGCCGTCGTCCTTCACGCGGGATGGCTGGATCAGGCTCTCACCCATTGTCCAATATTCCTCACTGCTGCCTC
CCGTAGGAGTCTGGTCCGTGTCTCAGTACCAGTGTGGGGGATCACCCTCTCAGGCCCCCTAAAGATCACTGACTTGGTAGGCCGTTACCCTACCAACTAT
CTAATCTTGCGCGTGCCCATCTCTATCCACCGGAGTTTTCAATATCAAATGATGCCATTCAATATATTATGGGGTATTAATCTTCCTTTCGAAAGGCTAT
CCCCCTGATAAAGGCAGGTTGCACACGTGTTCCGCACCCGTACGCCGCTCTCAAGATCCCGAAAGATCTCTACCGCTCGGCTTGCA

>EB118
GTTAGCGGCGGACGGGTGAGTAACACGTGGGCAACCTGCCTGTAAGACTGGGATAACTTCGGGAAACCGAAGCTAATACCGGATAGGATCTTCTCCTTCA
TGGGAGATGATTGAAAGATGGTTTCGGCTATCACTTACAGATGGGCCCGCGGTGCATTAGCTAGTTGGTGAGGTAACGGCTCACCAAGGCAACGATGCAT
AGCCGACCTGAGAGGGTGATCGGCCACACTGGGACTGAGACACGGCCCAGACTCCTACGGGAGGCAGCAGTAGGGAATCTTCCGCAATGGACGAAAGTCT
GACGGAGCAACGCCGCGTGAGTGATGAAGGCTTTCGGGTCGTAAAACTCTGTTGTTAGGGAAGAACAAGTACGAGAGTAACTGCTCGTACCTTGACGGTA
CCTAACCAGAAAGCCACGGCTAACTACGTGCCAGCAGCCGCGGTAATACGTAGGTGGCAAGCGTTATCCGGAATTATTGGGCGTAAAGCGCGCGCAGGCG
GTTTCTTAAGTCTGATGTGAAAGCCCACGGCTCAACCGTGGAGGGTCATTGGAAACTGGGGAACTTGAGTGCAGAAGAGAAAAGCGGAATTCCACGTGTA
GCGGTGAAATGCGTAGAGATGTGGAGGAACACCAGTGGCGAAGGCGGCTTTTTGGTCTGTAACTGACGCTGAGGCGCGAAAGCGTGGGGAGCAAACAGGA
TTAGATACCCTGGTAGTCCACGCCGTAAACGATGAGTGCTAAGTGTTAGAGGGTTTCCGCCCTTTAGTGCTGCAGCTAACGCATTAAGCACTCCGCCTGG
GGAGTACGGTCGCAAGACTGAAACTCAAAGGAATTGACGGGGGCCCGCACAAGCGGTGGAGCATGTGGTTTAATTCGAAGCAACGCGAAGAACCTTACCA
GGTCTTGACATCCTCTGACAACTCTAGAGATAGAGCGTTCCCCTTCGGGGGACAGAGTGACAGGTGGTGCATGGTTGTCGTCAGCTCGTGTCGTGAGATG
TTGGGTTAAGTCCCGCAACGAGCGCAACCCTTGATCTTAGTTGCCAGCATTTAGTTGGGCACTCTAAGGTGACTGCCGGTGACAAACCGGAGGAAGGTGG
GGATGACGTCAAATCATCATGCCCCTTATGACCTGGGCTACACACGTGCTACAATGGATGGTACAAAGGGCTGCAAGACCGCGAGGTCAAGCCAATCCCA

TAAAACCATTCTCAGTTCGGATTGTAGGCTGCAACTCGCCTACATGAAGCTGGAATCGCTAGTAATCGCGGATCAGCATGCCGCGGTGAATACGTTCCCG
TACAGTGTGCTCACGGCTCATGACGTCGTAACAAGGTATCCGTAGAGTTTGATCCTGGCTCAGGAAGTCGTAAAAGGAGCCTTGATTGACGTCGGA

>EB119
CTTCGGCGGCTGGCCCCATAAAGGTTACCCCACCGACTTCGGGTGTTACAAACTCTCGTGGTGTGACGGGCGGTGTGTACAAGGCCCGGGAACGTATTCA
CCGTGGCATGCTGATCCACGATTACTAGCGATTCCAGCTTCATGTAGGCGAGTTGCAGCCTACAATCCGAACTGAGAATGGTTTTATGGGATTGGCTCCC
CCTCGCGGGTTCGCAGCCCTTTGTACCATCCATTGTAGCACGTGTGTAGCCCAGGTCATAAGGGGCATGATGATTTGACGTCATCCCCACCTTCCTCCGG
TTTGTCACCGGCAGTCACCTTAGAGTGCCCAACTGAATGCTGGCAACTAAGATCAAGGGTTGCGCTCGTTGCGGGACTTAACCCAACATCTCACGACACG
AGCTGACGACAACCATGCACCACCTGTCACCGCCGTCCCCGAAGGGAAGGCCTAGTCTCCTAGGCGGTCGGCGGGATGTCAAGACCTGGTAAGGTTCTTC
GCGTTGCTTCGAATTAAACCACATGCTCCACCGCTTGTGCGGGCCCCCGTCAATTCCTTTGAGTTTTCAGTCTTGCGACCGTACTCCCCAGGCGGAGTGC
TTAATGCGTTAGCTGCAGCACTAAGGGGCGGAAACCCCCTAACACTTAGCACTCATCGTTTACGGCGTGGACTACCAGGGTATCTAATCCTGTTTGCTCC
CCACGCTTTCGCGCCTCAGCGTCAGTTACAGACCAGAAAGCCGCCTTCGCCACTGGTGTTCCTCCAAATCTCTACGCATTTCACCGCTACACTTGGAATT
CCGCTTTCCTCTTCTGCACTCAAGTCCCCCAGTTTCCAATGACCCTCCACGGTTGAGCCGTGGGCTTTCACATCAGACTTAAGGGACCGCCTGCGCGCGC
TTTACGCCCAATGATTCCGGACAACGCTTGCCACCTACGTATTACCGCGGCTGCTGGCACGTAGTTAGCCGTGGCTTTCTAATGAGGTACCGTCAAGGTA
CCGGCAGTTACTCCGGTACTTGTTCTTCCCTCACAACAGAGTTTTACGATCCGAGAACCTTCTTCACTCACGCGGCGTTGCTCCATCAGACTTTCGTCCA
TTGTGGAAGATTCCCTACTGCTGCCTCCCGTAGGAGTCTGGGCCGTGTCTCAGTCCCAGTGTGGCCGATCACCCTCTCAGGTCGGCTACGCATCGTCGCC
TTGGTGGGCCGTTACCCCACCAACTAGCTAATGCGCCGCGGGCCCATCCTGTAGTGACAGCCGAGGCCGCCTTTCAACACCGGACCATGTGGTCTGGTGG
ATTATTCGGTATTAGCCCCGGTTTCCCGGAGTTATCCCAATCTACAGGGCAGGTTGCCCACGTGTTACTCACCCGTCCGCCGCTGAATCTGGAGGAGCAA
GCTCCTCCTTCATCCGCTCGACTTGCATGT

>EB120
CACATGCAAGTCGAACGGTAGCACAGAGGAGCTTGCTCCTTGGGTGACGAGTGGCGGACGGGTGAGTAATGTCTGGGAAACTGCCCGATGGAGGGGGATA
ACTACTGGAAACGGTAGCTAATACCGCATAACGTCGCAAGACCAAAGAGGGGGACCTTCGGGCCTCTTGCCATCGGATGTGCCCAGATGGGATTAGCTAG
TAGGTGGGGTAACGGCTCACCTAGGCGACGATCCCTAGCTGGTCTGAGAGGATGACCAGCCACACTGGAACTGAGACACGGTCCAGACTCCTACGGGAGG
CAGCAGTGGGGAATATTGCACAATGGGCGCAAGCCTGATGCAGCCATGCCGCGTGTATGAAGAAGGCCTTCGGGTTGTAAAGTACTTTCAGCGAGGAGGA
AGGTGTTGTGGTTAATAACCGCAGCGATTGACGTTACTCGCAGAAGAAGCACCGGCTAACTCCGTGCCAGCAGCCGCGGTAATACGGAGGGTGCAAGCGT
TAATCGGAATTACTGGGCGTAAAGCGCACGCAGGCGGTCTGTCAAGTCGGATGTGAAATCCCCGGGCTCAACCTGGGAACTGCATCCGAAACTGGCAGGC
TAGAGTCTTGTAGAGGGGGGTAGAATTCCAGGTGTAGCGGTGAAATGCGTAGAGATCTGGAGGAATACCGGTGGCGAAGGCGGCCCCCTGGACAAAGACT
GACGCTCAGGTGCGAAAGCGTGGGGAGCAAACAGGATTAGATACCCTGGTAGTCCACGCCGTAAACGATGTCGACTTGGAGGTTGTGCCCTTGAGGCGTG
GCTTCCGGAGCTAACGCGTTAAGTCGACCGCCTGGGGAGTACGGCCGCAAGGTTAAAACTCAAATGAATTGACGGGGGCCCGCACAAGCGGTGGAGCATG
TGGTTTAATTCGATGCAACGCGAAGAACCTTACCTACTCTTGACATCCAGAGAACTTAGCAGAGATGCTTTGGTGCCTTCGGGAACTCTGAGACAGGTGC
TGCATGGCTGTCGTCAGCTCGTGTTGTGAAATGTTGGGTTAAGTCCCGCAACGAGCGCAACCCTTATCCTTTGTTGCCAGCGGTTAGGCCGGGAACTCAA
AGGAGACTGCCAGTGATAAACTGGAGGAAGGTGGGGATGACGTCAAGTCATCATGGCCCTTACGAGTAGGGCTACACACGTGCTACAATGGCATATACAA
AGAGAAGCGACCTCGCGAGAGCAAGCGGACCTCATAAAGTATGTCGTAGTCCGGATTGGAGTCTGCAACTCGACTCCATGAAGTCGGAATCGCTAGTAAT
CGTGGATCAGAATGCCACGGTGAATACGTTCCCGGGCCTTGTACACACCGCCCGTCACACCATGGGAGTGGGTTGCAAAAGAAGTAGGTAGCTTAACCTT

>EB121
TGCAGTCGAACGGTAGCACAGAGGAGCTTGCTCCTTGGGTGACGAGTGGCGGACGGGTGAGTAATGTCTGGGAAACTGCCCGATGGAGGGGGATAACTAC
TGGAAACGGTAGCTAATACCGCATAACGTCGCAAGACCAAAGAGGGGGACCTTCGGGCCTCTTGCCATCGGATGTGCCCAGATGGGATTAGCTAGTAGGT
GGGGTAACGGCTCACCTAGGCGACGATCCCTAGCTGGTCTGAGAGGATGACCAGCCACACTGGAACTGAGACACGGTCCAGACTCCTACGGGAGGCAGCA
GTGGGGAATATTGCACAATGGGCGCAAGCCTGATGCAGCCATGCCGCGTGTATGAAGAAGGCCTTCGGGTTGTAAAGTACTTTCAGCGAGGAGGAAGGTG
TTGTGGTTAATAACCGCAGCCATTGACGTTACTCGCAGAAGAAGCACCGGCTAACTCCGTGCCAGCAGCCGCGGTAATACGGAGGGTGCAAGCGTTAATC
GGAATTACTGGGCGTAAAGCGCACGCAGGCGGTCTGTCAAGTCGGATGTGAAATCCCCGGGCTCAACCTGGGAACTGCATCCGAAACTGGCAGGCTAGAG
TCTTGTAGAGGGGGGTAGAATTCCAGGTGTAGCGGTGAAATGCGTAGAGATCTGGAGGAATACCGGTGGCGAAGGCGGCCCCCTGGACAAAGACTGACGC
TCAGGTGCGAAAGCGTGGGGAGCAAACAGGATTAGATACCCTGGTAGTCCACGCCGTAAACGATGTCGACTTGGAGGTTGTGCCCTTGAGGCGTGGCTTC
CGGAGCTAACGCGTTAAGTCGACCGCCTGGGGGAGTACGGCCGCAAGGTTAAAACTCAAATGAATTGACGGGGGCCCGCACAAGCGGTGGAGCATGTGGT
TTAATTCGATGCAACGCGAAGAACCTTACCTACTCTTGACATCCAGAGAACTTAGCAGAGATGCTTTGGTGCCTTCGGGAACTCTGAGACAGGTGCTGCA
TGGCTGTCGTCAGCTCGTGTTGTGAAATGTTGGGTTAAGTCCCGCAACGAGCGCAACCCTTATCCTTTGTTGCCAGCGGTTAGGCCGGGAACTCAAAGGA
GACTGCCAGTGATAAACTGGAGGAAGGTGGGGATGACGTCAAGTCATCATGGCCCTTACGAGTAGGGCTACACACGTGCTACAATGGCATATACAAAGAG
AAGCGACCTCGCGAGAGCAAGCGGACCTCATAAAGTATGTCGTAGTCCGGATTGGAGTCTGCAACTCGACTCCATGAAGTCGGAATCGCTAGTAATCGTG
GATCAGAATGCCACGGTGAATACGTTCCCGGGCCTTGTACACACCGCCCGTCACACCATGGGAGTGGGTTGCAAAAGAAGTAGGTAGCTTAACCTTCGGG
AGGGCGC

>EB122
TTGGCGAACGGGTGAGTAACACGTGAGTAACCTGCCCTTAACTCTGGGATAAGCCTGGGAAACTGGGTCTAATACCGGATAGGAGCGTCCACCGCATGGT
GGGTGTTGGAAAGATTTATCGGTTTTGGATGGACTCGCGGCCTATCAGCTTGTTGGTGAGGTAATGGCTCACCAAGGCGACGACGGGTAGCCGGCCTGAG
AGGGTGACCGGCCACACTGGGACTGAGACACGGCCCAGACTCCTACGGGAGGCAGCAGTGGGGAATATTGCACAATGGGCGCAAGCCTGATGCAGCGACG
CCGCGTGAGGGATGACGGCCTTCGGGTTGTAAACCTCTTTCAGTAGGGAAGAAGCGAAAGTGACGGTACCTGCAGAAGAAGCACCGGCTAACTACGTGCC
AGCAGCCGCGGTAATACGTAGGGTGCGAGCGTTATCCGGAATTATTGGGCGTAAAGAGCTCGTAGGCGGTTTGTCGCGTCTGTCGTGAAAGTCCGGGGCT
TAACCCCGGATCTGCGGTGGGTACGGGCAGACTAGAGTGCAGTAGGGGAGACTGGAATTCCTGGTGTAGCGGTGGAATGCGCAGATATCAGGAGGAACAC
CGATGGCGAAGGCAGGTCTCTGGGCTGTAACTGACGCTGAGGAGCGAAAGCATGGGGAGCGAACAGGATTAGATACCCTGGTAGTCCATGCCGTAAACGT
TGGGCACTAGGTGTGGGGACCATTCCACGGTTTCCGCGCCGCAGCTAACGCATTAAGTGCCCCGCCTGGGGAGTACGGCCGCAAGGCTAAAACTCAAAGG
AATTGACGGGGGCCCGCACAAGCGGCGGAGCATGCGGATTAATTCGATGCAACGCGAAGAACCTTACCAAGGCTTGACATGTTCTCGATCGCCGTAGAGA
TACGGTTTCCCCTTTGGGGCGGGTTCACAGGTGGTGCATGGTTGTCGTCAGCTCGTGTCGTGAGATGTTGGGTTAAGTCCCGCAACGAGCGCAACCCTCG
TTCCATGTTGCCAGCACGTAATGGTGGGGACTCATGGGAGACTGCCGGGGTCAACTCGGAGGAAGGTGAGGACGACGTCAAATCATCATGCCCCTTATGT
CTTGGGCTTCACGCATGCTACAATGGCCGGTACAATGGGTTGCGATACTGTGAGGTGGAGCTAATCCCAAAAAGCCGGTCTCAGTTCGGATTGGGGTCTG
CAACTCGACCCCATGAAGTCGGAGTCGCTAGTAATCGCAGATCAGCAACGCTGCGGTGAATACGTTCCCGGTCCTTGTACACACCGCCCCTCAAGTTAGG

>EB123
TGCAGTCGAACGGTAACAGGTCTTCGGATGCTGACGAGTGGCGAACGGGTGAGTAATACATCGGAACGTGCCTAGTAGTGGGGGATAACTACTCGAAAGA
GTAGCTAATACCGCATGAGATCTACGGATGAAAGCAGGGGACCTTCGGGCCTTGTGCTACTAGAGCGGCTGATGGCAGATTAGGTAGTTGGTGGGGTAAA
GGCTTACCAAGCCTGCGATCTGTAGCTGGTCTGAGAGGACGACCAGCCACACTGGGACTGAGACACGGCCCAGACTCCTACGGGAGGCAGCAGTGGGGAA
TTTTGGACAATGGGCGAAAGCCTGATCCAGCAATGCCGCGTGCAGGATGAAGGCCCTCGGGTTGTAAACTGCTTTTGTACGGAACGAAAAGCCTGGGGCT
AATATCCCCGGGTCATGACGGTACCGTAAGAATAAGCACCGGCTAACTACGTGCCAGCAGCCGCGGTAATACGTAGGGTGCAAGCGTTAATCGGAATTAC
TGGGCGTAAAGCGTGCGCAGGCGGTTTTGTAAGACAGTGGTGAAATCCCCGGGCTCAACCTGGGAACTGCCATTGTGACTGCAAGGCTAGAGTGCGGCA
GAGGGGGATGGAATTCCGCGTGTAGCAGTGAAATGCGTAGATATGCGGAGGAACACCGATGGCGAAGGCAATCCCCTGGGCCTGCACTGACGCTCATGCA
CGAAAGCGTGGGGAGCAAACAGGATTAGATACCCTGGTAGTCCACGCCCTAAACGATGTCAACTGGTTGTTGGGTCTTAACTGACTCAGTAACGAAGCTA
ACGCGTGAAGTTGACCGCCTGGGGAGTACGGCCGCAAGGTTGAAACTCAAAGGAATTGACGGGGGACCCGCACAAGCGGTGGATGATGTGGTTTAATTCG

ATGCAACGCGAAAAACCTTACCCACCTTTGACATGGCAGGAACTTACCAGAGATGGTTTGGTGCTCGAAAGAGAACCTGCACACAGGTGCTGCATGGCTG
TCGTCAGCTCGTGTCGTGAGATGTTGGGTTAAGTCCCGCAACGAGCGCAACCCTTGCCATTAGTTGCTACATTCAGTTGAGCACTCTAATGGGACTGCCG
GTGACAAACCGGAGGAAGGTGGGGATGACGTCAAGTCCTCATGGCCCTTATAGGTGGGGCTACACACGTCATACAATGGCTGGTACAAAGGGTTGCCAAC
CCGCGAGGGGGAGCTAATCCCATAAAGCCAGTCGTAGTCCGGATCGCAGTCTGCAACTCGACTGCGTGAAGTCGGAATCGCTAGTAATCGTGGATCAGAA
TGTCACGGTGAATACGTTCCCGGGTCTTGTACACACCGCCCGTCACACCATGGGAGCGGGTCTCGCCAGAAGTAGGTAGCCTAACCGCAAGGAGGGCGCT

>EB124
GCTCCCTCCAAAGGTTAGGCCACTGGCTTCGGGTGTTACCGACTTTCATGACTTGACGGGCGGTGTGTACAAGGCCCGGGAACGTATTCACCGTAGCGTT
GCTGATCTACGATTACTAGCGACTCCGACTTCATGAGGTCGAGTTGCAGACCTCAATCCGAACTGAGACCGGCTTTTTGGGATTCGCTCCACCTTACGGT
ATCGCAGCCCATTGTACCGGCCATTGTAGCATGCGTGAAGCCCAAGACATAAGGGGCATGATGATTTGACCTCATCCCCACCTTCCTCCGAGTTGACCCC
GGCAGTATCCCATGAGTTCCCACCATTACGTGCTGGCAACATAGGACGAGGGTTGCGCTCGTTGCGGGACTTAACCCAACATCTCACGACACGAGCTGAC
GACAACCATGCACCACCTGTATACGAGTGTCCAAAGAGTTCACTGTCTCCAGTGCGTTCTCGTATATGTCAAGCCTTGGTAAGGTTCTTCGCGTTGCATC
GAATTAATCCGCATGCTCCGCCGCTTGTGCGGGCCCCCGTCAATTCCTTTGAGTTTTAGCCTTGCGGCCGTACTCCCCAGGCGGGGAACTTAATGCGTTA
GCTACGACACGGAGACCGTGGAATGGTCCCCACATCTAGTTCCCAACGTTTACGGCATGGACTACCAGGGTATCTAAGCCTGTTTGCTCCCCATGCTTTC
GCTCCTCAGCGTCAGTTACGGCCCAGAGATCTGCCTTCGCCATCGGTGTTCCTCCTGATATCTGCGCATTCCACCGCTACACCAGGAATTCCAATCTCCC
CTACCGCACTCTAGTCTGCCCGTACCCACTGCAAGCCCGAGGTTGAGCCTCGGGTTTTCACAGCAGACGTGACAAACCGCCTACGAGCTCTTTACGCCCA
ATAATTCCGGACAACGCTTGCACCCTACGTATTACCGCGGCTGCTGGCACGTAGTTAGCCGGTGCTTTTTCTGCAGGTACCGTCACTCTCGCTTCTTCCC
TGCTAAAAGAGGTTTACAACCCGAAGGCCTTCGTCCCTCACGCGGCGTTGCTGCATCAGGCTTGCGCCCATTGTGCAATATTCCCCACTGCTGCCTCCCG
TAGGAGTCTGGGCCGTGTCTCAGTCCCAGTGTGGCCGGTCACCCTCTCAGGCCGGCTACCCGTCGTCGCCTTGGTGAGCCATTACCTCACCAACTAGCTG
ATAGGCCGTGAGCTGATCCTTGACCGAAGTTCTTTCCACCACCAGGAGATGCCTCCGGTGGTCGTATCCGGTATTAGACGCCGTTTCCAGCGCTTATCCC
AGAGTCAAGGGCACATTGCTCACGTATTACTCACCCGTTCGCCACTAATCCGGAGGAGCAAGCTCCTCCTTCATCGTTCGACTTGCA

>EB125
TGCAGTCGAACGATGATCAGGAGCTTGCTCCTGTGATTAGTGGCGAACGGGTGAGTAACACGTGAGTAACCTGCCCCTGACTCTGGGATAAGCGTTGGAA
ACGACGTCTAATACTGGATATGATCACTGGCCGCATGGTCTGGTGGTGGAAAGATTTTTCGGTTGGGGATGGACTCGCGGCCTATCAGCTTGTTGGTGAG
GTAATGGCTCACCAAGGCGACGACGGGTAGCCGGCCTGAGAGGGTGACCGGCCACACTGGGACTGAGACACGGCCCAGACTCCTACGGGAGGCAGCAGTG
GGGAATATTGCACAATGGGCGAAAGCCTGATGCAGCAACGCCGCGTGAGGGATGACGGCCTTCGGGTTGTAAACCTCTTTTAGTAGGGAAGAAGCGAAAG
TGACGGTACCTGCAGAAAAAGCACCGGCTAACTACGTGCCAGCAGCCGCGGTAATACGTAGGGTGCAAGCGTTGTCCGGAATTATTGGGCGTAAAGAGCT
CGTAGGCGGTTTGTCGCGTCTGCTGTGAAATCCCGAGGCTCAACCTCGGGCTTGCAGTGGGTACGGGCAGACTAGAGTGCGGTAGGGGAGATTGGAATTC
CTGGTGTAGCGGTGGAATGCGCAGATATCAGGAGGAACACCGATGGCGAAGGCAGATCTCTGGGCCGTAACTGACGCTGAGGAGCGAAAGCGTGGGGAGC
GAACAGGATTAGATACCCTGGTAGTCCACGCCGTAAACGTTGGGCGCTAGATGTAGGGACCTTTCCACGGTTTCTGTGTCGTAGCTAACGCATTAAGCGC
CCCGCCTGGGGAGTACGGCCGCAAGGCTAAAACTCAAAGGAATTGACGGGGGCCCGCACAAGCGGCGGAGCATGCGGATTAATTCGATGCAACGCGAAGA
ACCTTACCAAGGCTTGACATACGCCGGAAACGGCCAGAGATGGTCGCCCCCTTGTGGTCGGTGTACAGGTGGTGCATGGTTGTCGTCAGCTCGTGTCGTG
AGATGTTGGGTTAAGTCCCGCAACGAGCGCAACCCTCGTTCTATGTTGCCAGCGCGTTATGGCGGGGACTCATAGGAGACTGCCGGGGTCAACTCGGAGG
AAGGTGGGGATGACGTCAAATCATCATGCCCCTTATGTCTTGGGCTTCACGCATGCTACAATGGCCGGTACAAAGGGCTGCGATACCGTAAGGTGGAGCG
AATCCCAAAAAGCCGGTCTCAGTTCGGATTGAGGTCTGCAACTCGACCTCATGAAGTCGGAGTCGCTAGTAATCGCAGATCAGCAACGCTGCGGTGAATA
CGTTCCCGGGCCTTGTACACACCGCCCGTCAAGTCATGAAAGTCGGTAACACCCGAAGCGCGGTGGCCTAACCC

>EB126
TTCCACAAGGGTTAGGCCACCGGCTTCGGGTGTTACCGACTTTCATGACTTGACGGGCGGTGTGTACAAGGCCCGGGAACGTATTCACCGCAGCGTTGCT
GATCTGCGATTACTAGCGACTCCGACTTCATGAGGTCGAGTTGCAGACCTCAATCCGAACTGAGACCGGCTTTTTGGGATTCGCTCCACCTTACGGTATC
GCAGCCCTTTGTACCGGCCATTGTAGCATGCGTGAAGCCCAAGACATAAGGGGCATGATGATTTGACGTCATCCCCACCTTCCTCCGAGTTGACCCCGGC
AGTCTCCTATGAGTCCCCGGCATAACCCGCTGGCAACATAGAACGAGGGTTGCGCTCGTTGCGGGACTTAACCCAACATCTCACGACACGAGCTGACGAC
AACCATGCACCACCTGTACACCGACCACAAGGGGGCGACCATCTCTGGCCGTTTCCGGTGTATGTCAAGCCTTGGTAAGGTTCTTCGCGTTGCATCGAAT
TAATCCGCATGCTCCGCCGCTTGTGCGGGCCCCCGTCAATTCCTTTGAGTTTTAGCCTTGCGGCCGTACTCCCCAGGCGGGGCGCTTAATGCGTTAGCTA
CGACACAGAAACCGTGGAAAGGTCCCTACATCTAGCGCCCAACGTTTACGGCATGGACTACCAGGGTATCTAATCCTGTTCGCTCCCCATGCTTTCGCTC
CTCAGCGTCAGTTACGGCCCAGAGATCTGCCTTCGCCATCGGTGTTCCTCCTGATATCTGCGCATTCCACCGCTACACCAGGAATTCCAATCTCCCCTAC
CGCACTCTAGTCTGCCCGTACCCACTGCAAGCCCGAGGTTGAGCCTCGGGATTTCACAGCAGACGCGACAAACCGCCTACGAGCTCTTTACGCCCAATAA
TTCCGGACAACGCTTGCACCCTACGTATTACCGCGGCTGCTGGCACGTAGTTAGCCGGTGCTTTTTCTGCAGGTACCGTCACTTTCGCTTCTTCCCTACT
AAAAGAGGTTTACAACCCGAAGGCCGTCATCCCTCACGCGGCGTTGCTGCATCAGGCTTTCGCCCATTGTGCAATATTCCCCACTGCTGCCTCCCGTAGG
AGTCTGGGCCGTGTCTCAGTCCCAGTGTGGCCGGTCACCCTCTCAGGCCGGCTACCCGTCGTCGCCTTGGTGAGCCATTACCTCACCAACAAGCTGATAG
GCCGCGAGTCCATCCCCAACCAAAAAATCTTTCCACCACCAGACCATGCGGCCGGCGATCATATCCAGTATTAGACGTCGTTTCCAACGCTTATCCCAGA
GTCAGGGGCAGGTTACTCACGTGTTACTCACCCGTTCGCCACTAATCCACCCAGCAAGCTGGGCATCATCGTTCGACTTGCA

>EB127
TAAGTCGTAACAAGGTAACCGTAGAGTTTGATTATGGCTCAGTAAGTCGTAACAAGGTAACCGTGCAGTTTGATCATGGCTCATTAAGGCAAAACAAGGT
AACCGTGGAGTTTGATTATCTCCTTCATGGGAGAAGATTGAAAGATGGTTTCGGCTATCATTTACTGATGGGCCCGCGGTGCATTAGCTAGTTGGTGAGG
TAACGGCTCACCAAGGCAACGATGCATAGCCGACCTGAGAGGGTGATCGGCCACACTGGGACTGAGACACGGCCCAGACTCCTACGGGAGGCAGCAGTAG
GGAATCTTCCGCAATGGACGAAAGTCTGACGGAGCAACGCCGCGTGAGTGATGAAGGCTTTCGGGTCGTAAAACTCTGTTGTTAGGGAAGAACAAGTACG
AGAGTAACTGCTCGTACCTTGACGGTACCTAACCAGAAGCCACGGCTAACTACGTGCCAGCAGCCGCGGTAATACGTAGGTGGCAAGCGTTATCCGGAAT
TATTGGGCGTAAAGCGCGCGCAGGCGGTTTCTTAAGTCTGATGTGAAAGCCCACGGCTCAACCGTGGAGGGTCATTGGAAACTGGGGAACTTGAGTGCAG
AAGAGAAAAGCGGAATYCACGTGTAGCGGTGAAATGCGTAGAGATGTGGAGGAACACCAGTGGCGAAGGCGGCTTTTTGGTCTGTAACTGACGCTGAGGC
GCGAAAGCGTGGGGAGCAAACAGGATTAGATACCCTGGTAGTCCACGCCGTAAACGATGAGTGCTAAGTGTTAGAGGGTTTCCGCCCTTTAGTGCTGCAG
CTAACGCATTAAGCACTCCGCCTGGGGAGTACGGTCGCAAGACTGAAACTCAAAGGAATTGACGGGGGCCCGCACAAGCGGTGGAGCATGTGGTTTAATT
CGAAGCAACGCGAAGAACCTTACCAGGTCTTGACATCCTCTGACAACTCTAGAGATAGAGCGTTCCCCTTCGGGGGACAGAGTGACAGGTGGTGCATGGT
TGTCGTCAGCTCGTGTCGTGAGATGTTGGGTTAAGTCCCGCAACGAGCGCAACCCTTGATCTTAGTTGCCAGCATTCAGTTGGGCACTCTAAGGTGACTG
CCGGTGACAAACCGGAGGAAGGTGGGGATGACGTCAAATCATCATGCCCCTTATGACCTGGGCTACACACGTGCTACAATGGATGGTACAAAGGGCTGCA
AGACCGCGAGGTCAAGCCAGTCCCATAAAACCATTTTCAGTTCGGGTTGTAGGCTGCAACTGGCATTCATGACGCGGGAACCGTTGAGTCAACGGATCAG
CATGCCGCGGTGA

>EB128
CAAGGGTTAGGCCACCGGCTTCGGGTGTTACCGACTTTCGTGACTTGACGGGCGGTGTGTACAAGGCCCGGGAACGTATTCACCGCAGCGTTGCTGATCT
GCGATTACTAGCGACTCCGACTTCATGGGGTCGAGTTGCAGACCCCAATCCGAACTGAGACCGGTTTTTTGGGATTAGCTCCACCTCACGGTTTCGCAAC
CCTCTGTACCGGCCATTGTAGCATGCGTGAAGCCCAAGACATAAGGGGCATGATGATTTGACGTCATCCCCACCTTCCTCCGAGTTGACCCCGGCAGTCT
CCCATGAGTCCCCACCTTGACGTGCTGGCAACATGGAACGAGGGTTGCGCTCGTTGCGGGACTTAACCCAACATCTCACGACACGAGCTGACGACAACCA
TGCACCACCTGTACACCAGTCCGAAGACGCACACATCTCTGCATGATTCCGGTGTATGTCAAGCCTTGGTAAGGTTCTTCGCGTTGCATCGAATTAATCC
GCATGCTCCGCCGCTTGTGCGGGCCCCCGTCAATTCCTTTGAGTTTTAGCCTTGCGGCCGTACTCCCCAGGCGGGGCGCTTAATGCGTTAGCTGCGGCAC
GGAACTCGTGGAATGAGTCCCACACCTAGCGCCCAACGTTTACGGCATGGACTACCAGGGTATCTAATCCTGTTCGCTCCCCATGCTTTCGCTTCTCAGC

GTCAGTAATGGCCCAGAGACCTGCCTTCGCCATCGGTGTTCCTCCTGATATCTGCGCATTTCACCGCTACACCAGGAATTCCAGTCTCCCCTACCACACT
CTAGTCTGCCCGTACCCACTGCAGAACCGGAGTTAAGCCCCGGTCTTTCACAGCAGACGCGACAAAACCGCCTACAAGCTCTTTACGCCCAATAATTCCG
GACAACGCTCGCACCCTACGTATTACCGCGGCTGCTGGCACGTAGTTAGCCGGTGCTTCTTCTCCAGGTACCGTCACGTTAGCTTCGTCCCTGGTGAAAG
AGGTTTACAACCCGAAGGCCGTCATCCCTCACGCGGCGTCGCTGCATCAGGCTTTCGCCCATTGTGCAATATTCCCCACTGCTGCCTCCCGTAGGAGTCT
GGGCCGTGTCTCAGTCCCAGTGTGGCCGGTCGCCCTCTCAGGCCGGCTACCCGTCGTCGCCTTGGTAGGCCATTACCCCACCAACAAGCTGATAGGCCGC
GAGTCCATCCCCCACCACAAAAACTTTCCACCAACACTCCATGCGGAGATCGGTCGTATCCAGTATTAGACCCAGTTTCCCAGGCTTATCCCAGAGTGAG
GGGCAGGTTACTCACGTGTTACTCACCCGTTCGCCACTAATCCACACCAGCAAGCTGGTGCTTCATCGTTCGACTGCA

>EB129
CGGCTCCCCCCACAAGGGTTAGGCCACCGGCTTCGGGTGTTACCAGACTTTCGTGACTTGACGGGCGGTGTGTACAAGGCCCGGGAACGTATTCACCGCA
GCGTTGCTGATCTGCGATTACTAGCGACTCCGACTTCATGGGGTCGAGTTGCAGACCCCAATCCGAACTGAGACCGGCTTTTTGGGATTAGCTCCACCTC
ACAGTATCGCAACCCATTGTACCGGCCATTGTAGCATGCGTGAAGCCCAAGACATAAGGGGCATGATGATTTGACGTCGTCCTCACCTTCCTCCGAGTTG
ACCCCGGCAGTCTCCCATGAGTCCCCACCACTACGTGCTGGCAACATGGAACGAGGGTTGCGCTCGTTGCGGGACTTAACCCAACATCTCACGACACGAG
CTGACGACAACCATGCACCACCTGTGAACCCGCCCCAAAGGGGAAACCGTATCTCTACGGCGATCGAGAACATGTCAAGCCTTGGTAAGGTTCTTCGCGT
TGCATCGAATTAATCCGCATGCTCCGCCGCTTGTGCGGGCCCCCGTCAATTCCTTTGAGTTTTAGCCTTGCGGCCGTACTCCCCAGGCGGGGCACTTAA
TGCGTTAGCTGCGGCGCGGAAACCGTGGAATGGTCCCCACACCTAGTGCCCAACGTTTACGGCATGGACTACCAGGGTATCTAATCCTGTTCGCTCCCCA
TGCTTTCGCTCCTCAGCGTCAGTTACAGCCCAGAGACCTGCCTTCGCCATCGGTGTTCCTCCTGATATCTGCGCATTCCACCGCTACACCAGGAATTCCA
GTCTCCCCTACTGCACTCTAGTCTGCCCGTACCCACCGCAGATCCGGGGTTAAGCCCCGGACTTTCACGACAGACGCGACAAACCGCCTACGAGCTCTTT
ACGCCCAATAATTCCGGATAACGCTCGCACCCTACGTATTACCGCGGCTGCTGGCACGTAGTTAGCCGGTGCTTCTTCTGCAGGTACCGGTCACTTTCGC
TTCTTCCCTACTGAAAGAGGTTTACAACCCGAAGGCCGTCATCCCTCACGCGGCGTCGCTGCATCAGGCTTGCGCCCATTGTGCAATATTCCCCACTGCT
GCCTCCCGTAGGAGTCTGGGCCGTGTCTCAGTCCCAGTGTGGCCGGTCACCCTCTCAGGCCGGCTACCCGTCGTCGCCTTGGTGAGCCATTACCTCACCA
ACAAGCTGATAGGCCGCGAGTCCATCCAAAACCGATAAATCTTTCCAACACCCACCATGCGGTGGACGCTCCTATCCGGTATTAGACCCAGTTTCCCAGG
CTTATCCCAGAGTTAAGGGCAGGTTACTCACGTGTTACTCACCCGTTCGCCACTAATCCACCCAGCAAGCTGGGCTTCATCGTTCGACTGCA

>EB130
CACGGAGCTTGCTCTGTGGGATCAGTGGCGAACGGGTGAGTAACACGTGAGCAACCTGCCCCTGACTCTGGGATAAGCGCTGGAAACGGCGTCTAATACT
GGATATGTGACGTGATCGCATGGTCTGCGTCTGGAAAGAATTTCGGTTGGGGATGGGCTCGCGGCCTATCAGCTTGTTGGTGAGGTAATGGCTCACCAAG
GCGTCGACGGGTAGCCGGCCTGAGAGGGTGACCGGCCACACTGGGACTGAGACACGGCCCAGACTCCTACGGGAGGCAGCAGTGGGGAATATTGCACAAT
GGGCGCAAGCCTGATGCAGCAACGCCGCGTGAGGGACGACGGCCTTCGGGTTGTAAACCTCTTTTAGCAGGGAAGAAGCGAAAGTGACGGTACCTGCAGA
AAAAGCGCCGGCTAACTACGTGCCAGCAGCCGCGGTAATACGTAGGGCGCAAGCGTTATCCGGAATTATTGGGCGTAAAGAGCTCGTAGGCGGTTTGTCG
CGTCTGCTGTGAAATCCGGAGGCTCAACCTCCGGCCTGCAGTGGGTACGGGCAGACTAGAGTGCGGTAGGGGAGATTGGAATTCCTGGTGTAGCGGTGGA
ATGCGCAGATATCAGGAGGAACACCGATGGCGAAGGCAGATCTCTGGGCCGTAACTGACGCTGAGGAGCGAAAGGGTGGGGAGCAAACAGGCTTAGATAC
CCTGGTAGTCCACCCCGTAAACGTTGGGAACTAGTTGTGGGGTCCATTCCACGGATTCCGTGACGCAGCTAACGCATTAAGTTCCCCGCCTGGGGAGTAC
GGCCGCAAGGCTAAAACTCAAAGGAATTGACGGGGACCCGCACAAGCGGCGGAGCATGCGGATTAATTCGATGCAACGCGAAGAACCTTACCAAGGCTTG
ACATATACGAGAACGGGCCAGAAATGGTCAACTCTTTGGACACTCGTAAACAGGTGGTGCATGGTTGTCGTCAGCTCGTGTCGTGAGATGTTGGGTTAAG
TCCCGCAACGAGCGCAACCCTCGTTCTATGTTGCCAGCACGTAATGGTGGGAACTCATGGGATACTGCCGGGGTCAACTCGGAGGAAGGTGGGGATGACG
TCAAATCATCATGCCCCTTATGTCTTGGGCTTCACGCATGCTACAATGGCCGGTACAAAGGGCTGCAATACCGTGAGGTGGAGCGAATCCCAAAAAGCCG
GTCCCAGTTCGGATTGAGGTCTGCAACTCGACCTCATGAAGTCGGAGTCGCTAGTAATCGCAGATCAGCAACGCTGCGGTGAATACGTTCCCGGGTCTTG
TACACACCGCCCGTCAAGTCATGAAAGTCGGTAACACCTGAAGCCGGTGGCCTAACCC

>EB131
AAGTCGACGGTAAGGCCCCTTCGGGGGTACACGAGTGGCGAACGGGTGAGTAACACGTGGGTAATCTGCCCTGCACTTCGGGATAAGCCTGGGAAACCGG
GTCTAATACCGGATATGAACTCCTGCCGCATGGTGGGGGTTGGAAAGTTTTTCGGTGCAGGATGAGTCCGCGGCCTATCAGCTTGTTGGTGGGGTAATGG
CCTACCAAGGCGACGACGGGTAGCCGGCCTGAGAGGGTGATCGGCCACACTGGGACTGAGACACGGCCCAGACTCCTACGGGAGGCAGCAGTGGGGAATA
TTGCACAATGGGCGAAAGCCTGATGCAGCGACGCCGCGTGGGGGATGACGGTCTTCGGATTGTAAACCCCTTTCAGTAGGGACGAAGCGCAAGTGACGGT
ACCTGCAGAAGAAGCACCGGCTAACTACGTGCCAGCAGCCGCGGTAATACGTAGGGTGCGAGCGTTGTCCGGAATTACTGGGCGTAAAGAGCTCGTAGGC
GGTTTGTCACGTCGTCTGTGAAATCCCTCGGCTTAACCGGGGGCGTGCAGGCGATACGGGCAGACTTGAGTACTACAGGGGAGACTGGAATTCCTGGTGT
AGCGGTGAAATGCGCAGATATCAGGAGGAACACCGGTGGCGAAGGCGGGTCTCTGGGTAGTAACTGACGCTGAGGAGCGAAAGCATGGGTAGCGAACAGG
ATTAGATACCCTGGTAGTCCATGCCGTAAACGGTGGGCGCTAGGTGTGGGGTCCTTCCACGGACTCCGTGCCGTAGCTAACGCATTAAGCGCCCCGCCTG
GGGAGTACGGCCGCAAGGCTAAAACTCAAAGGAATTGACGGGGGCCCGCACAAGCGGCGGAGCATGTGGATTAATTCGATGCAACGCGAAGAACCTTACC
TAGGCTTGACATATACAGGACGACGGCAGAGATGTCGTTTCCCTTGTGGCTTGTATACAGGTGGTGCATGGTTGTCGTCAGCTCGTGTCGTGAGATGTTG
GGTTAAGTCCCGCAACGAGCGCAACCCCTGTCTCATGTTGCCAGCACGTAATGGTGGGGACTCGTGAGAGACTGCCGGGGTCAACTCGGAGGAAGGTGGG
GATGACGTCAAATCATCATGCCCCTTATGTCTAGGGCTTCACACATGCTACAATGGCTAGTACAGAGGGCTGCGATACCGCGAGGTGGAGCGAATCCCTT
AAAGCTGGTCTCAGTTCGGATTGGGGTCTGCAACTCGACCCCATGAAGTCGGAGTCGCTAGTAATCGCAGATCAGCAACGCTGCGGTGAATACGTTCCCG
GGCCTTGTACACACCGCCCGTCACGTCATGAAAGTCGGTAACACCCGAAGCCGGTGGCCTAACCCTTGTGGA

>EB132
GCTCAGGATGAACGCTGGCGGCGTGCCTAATACATGCAAGTCGAGCGAACTGATTAGAAGCTTGCTTCTATGACGTTAGCGGCGGACGGGTGAGTAACAC
GTGGGCAACCTGCCTGTAAGACTGGGATAACTTCGGGAAACCGAAGCTAATACCGGATAGGATCTTCTCCTTCATGGGAGATGATTGAAAGATGGTTTCG
GCTATCACTTACAGATGGGCCCGCGGTGCATTAGCTAGTTGGTGAGGTAACGGCTCACCAAGGCAACGATGCATAGCCGACCTGAGAGGGTGATCGGCCA
CACTGGGACTGAGACACGGCCCAGACTCCTACGGGAGGCAGCAGTAGGGAATCTTCCGCAATGGACGAAAGTCTGACGGAGCAACGCCGCGTGAGTGATG
AAGGCTTTCGGGTCGTAAAACTCTGTTGTTAGGGAAGAACAAGTACGAGAGTAACTGCTCGTACCTTGACGGTACCTAACCAGAAAGCCACGGCTAACTA
CGTGCCAGCAGCCGCGGTAATACGTAGGTGGCAAGCGTTATCCGGAATTATTGGGCGTAAAGCGCGCGCAGGCGGTTTCTTAAGTCTGATGTGAAAGCCC
ACGGCTCAACCGTGGAGGGTCATTGGAAACTGGGGAACTTGAGTGCAGAAGAGAAAAGCGGAATTCCACGTGTAGCGGTGAAATGCGTAGAGATGTGGAG
GAACACCAGTGGCGAAGGCGGCTTTTTGGTCTGTAACTGACGCTGAGGCGCGAAAGCGTGGGGAGCAAACAGGATTAGATACCCTGGTAGTCCACGCCGT
AAACGATGAGTGCTAAGTGTTAGAGGGTTTCCGCCCTTTAGTGCTGCAGCTAACGCATTAAGCACTCCGCCTGGGGAGTACGGTCGCAAGACTGAAACTC
AAAGGAATTGACGGGGGCCCGCACAAGCGGTGGAGCATGTGGTTTAATTCGAAGCAACGCGAAGAACCTTACCAGGTCTTGACATCCTCTGACAACTCTA
GAGATAGAGCGTTCCCCTTCGGGGGACAGAGTGACAGGTGGTGCATGGTTGTCGTCAGCTCGTGTCGTGAGATGTTGGGTTAAGTCCCGCAACGAGCGCA
ACCCTTGATCTTAGTTGCCAGCATTTAGTTGGGCACTCTAAGGTGACTGCCGGTGACAAACCGGAGGAAGGTGGGGATGACGTCAAATCATCATGCCCC
TTATGACCTGGGCTACACACGTGCTACAATGGATGGTACAAAGGGCTGCAAGACCGCGAGGTCAAGCCAATCCCATAAAACCATTCTCAGTTCGGATTGT
AGGCTGCAACTCGCCTACATGAAGCTGGAATCGCTAGTAATCGCGGATCAGCATGCCGCGGTGAATACGTTCCCGGGCCTTGTACACACCGCCCGTCACA
CCACGAGAGTTTGTAACACCCGAAGTCGGTGGAGTAACCGTAAGGAGCTAGCCGCCTAAGGTGGGACAAATGATTGGGGTGAAT

>EB133
GATGTGACGAGCGGTGTGTACAATGTACGGGAACGTATTCACCGCGGCATGCTGATCCGCGATTACTAACGATTCCAGCTTCATGTAGGCGAGTTGCAGC
CTACAATCCGAACTGAGAATGGTTTTATGGGATTGGCTTGACCTCGCGGTCTTGCAGCCCTTTGTACCATCCATTGTAGCACGTGTGTAGCCCAGGTCAT
AAGGGGCATGATGATTTGACGTCATCCCCACCTTCCTCCGGTTTGTCACCGGCAGTCACCTTAGAGTGCCCAACTAAATGCTGGCAACTAAGATCAAGGG
TTGCGCTCGTTGCGGGACTTAACNCCAACATCTCNACGACACGAGCTGACGACAANCCATGCACCACCTGTCACTCTGTCCCCCGAAGGGGAACGCTCTA

TCTCTAGAGTTGTCAGAGGATGTCAAGACCTGGTAAGGTTCTTCGCGTTGCTTCGAATTAAACCACATGCTCCACCGCTTGTGCGGGCCCCCGTCAATTC
CTTTGAGTTTCAGTCTTGCGACCGTACTCCCCAGGCGGAGTGCTTAATGCGTTAGCTGCAGCACTAAAGGGCGGAAACCCTCTAACACTTAGCACTCATC
GTTTACGGCGTGGACTACCAGGGTATCTAATCCTGTTTGCTCCCCACGCTTTCGCGCCTCAGCGTCAGTTACAGACCAAAAAGCCGCCTTCGCCACTGGT
GTTCCTCCACATCTCTACGCATTTCACCGCTACACGTGGAATTCCGCTTTTCTCTTCTGCACTCAAGTTCCCCAGTTTCCAATGACCCTCCACGGTTGAG
CCGTGGGCTTTCACATCAGACTTAAGAAACCGCCTGCGCGCGCTTTACGCCCAATAATTCCGGATAACGCTTGCCACCTACGTATTACCGCGGCTGCTGG
CACGTAGTTAGCCGTGGCTTTCTGGTTAGGTACCGTCAAGGTACAAGCAGTTACTCTCGTACTTGTTCTTCCCTAACAACAGAGTTTTACGACCCGAAAG
CCTTCATCACTCACGCGGCGTTGCTCCGTCAGACTTTCGTCCATTGCGGAAGATTCCCTACTGCTGCCTCCCGTAGGAGTCTGGGCCGTGTCTCAGTCCC
AGTGTGGCCGATCACCCTCTCAGGTCGGCTATGCATCGTTGCCTTGGTGAGCCGTTACCTCACCAACTAGCTAATGCACCGCGGGCCCATCTGTAAGTGA
TAGCCGAAACCATCTTTCAATCATCTCCCATGAAGGAGAAGATCCTATCCGGTATTAGCTTCGGTTTCCCGAAGTTATCCCAGTCTTACAGGCAGGTTGC
CCACGTGTTAATCACCCGTCCGCCGTCAAACT

>EB134
CGCCCCGCAAGGGGAGCGGCAGACGGGTGAGTAACGCGTGGGAATCTACCCTTTTCTACGGAATAACGCAGGGAAACTTGTGCTAATACCGTATACGCCC
TTCGGGGGAAAGATTTATCGGGAAAGGATGAGCCCGCGTTGGATTAGCTAGTTGGTGGGGTAAAGGCCTACCAAGGCGACGATCCATAGCTGGTCTGAGA
GGATGATCAGCCACATTGGGACTGAGACACGGCCCAAACTCCTACGGGAGGCAGCAGTGGGGAATATTGGACAATGGGCGCAAGCCTGATCCCAGCCATG
CCGCGTGAGTGATGAAGGCCCTAGGGTTGTAAAGCTCTTTCACCGGTGAAGATAATGACGGTAACCGGAGAAGAAGCCCCGGCTAACTTCGTGCCAGCAG
CCGCGGTAATACGAAGGGGGCTAGCGTTGTTCGGAATTACTGGGCGTAAAGCGCACGTAGGCGGACATTTAAGTCAGGGGTGAAATCCCGGGGCTCAACC
CCGGAACTGCCTTTGATACTGGGTGTCTAGAGTATGGAAGAGGTGAGTGGAATTCCGAGTGTAGAGGTGAAATTCGTAGATATTCGGAGGAACACCAGTG
GCGAAGGCGGCTCACTGGTCCATTACTGACGCTGAGGTGCGAAAGCGTGGGGAGCAAACAGGATTAGATACCCTGGTAGTCCACGCCGTAAACGATGAAT
GTTAGCCGTCGGGCAGTTTACTGTTCGGTGGCGCAGCTAACGCATTAAACATTCCGCCTGGGGAGTACGGTCGCAAGATTAAAACTCAAAGGAATTGACG
GGGGCCCGCACAAGCGGTGGAGCATGTGGTTTAATTCGAAGCAACGCGCAGAACCTTACCAGCCCTTGACATCCCGATCGCGGATTACGGAGACGTTTTC
CTTCAGTTCGGCTGGATCGGAGACAGGTGCTGCATGGCTGTCGTCAGCTCGTGTCGTGAGATGTTGGGTTAAGTCCCGCAACGAGCGCAACCCTCGCCCT
TAGTTGCCAGCATTTAGTTGGGCACTCTAAGGGGACTGCCGGTGATAAGCCGAGAGGAAGGTGGGGATGACGTCAAGTCCTCATGGCCCTTACGGGCTGG
GCTACACACGTGCTACAATGGTGGTGACAGTGGGCAGCGAGACCGCGAGGTCGAGCTAATCTCCAAAAGCCATCTCAGTTCGGATTGCACTCTGCAACTC
GAGTGCATGAAGTTGGAATCGCTAGTAATCGCAGATCAGCATGCTGCGGTGAATACGTTCCCGGGCCTTGTACACACCGCCCGTCACACCATGGGAGTTG
GTTCTACCCGAAGGTAGTGCGCTAACCGCAAGGAGGCAGCT

>EB135
TGCAAGTCGAGCGGTAGCACAAGAGAGCTTGCTCTCGGGTGACGAGCGGCGGACGGGTGAGTAATGTCTGGGAAACTGCCTGATGGAGGGGGATAACTAC
TGGAAACGGTAGCTAATACCGCATAACGTCGCAAGACCAAAGTGGGGGACCTTCGGGCCTCATGCCATCAGATGTGCCCAGATGGGATTAGCTAGTAGGT
GGGGTAATGGCTCACCTAGGCGACGATCCCTAGCTGGTCTGAGAGGATGACCAGCCACACTGGAACTGAGACACGGTCCAGACTCCTACGGGAGGCAGCA
GTGGGGAATATTGCACAATGGGCGCAAGCCTGATGCAGCCATGCCGCGTGTATGAAGAAGGCCTTCGGGTTGTAAAGTACTTTCAGCGAGGAGGAAGGCG
TTAAGGTTAATAACCTTGGCGATTGACGTTACTCGCAGAAGAAGCACCGGCTAACTCCGTGCCAGCAGCCGCGGTAATACGGAGGGTGCAAGCGTTAATC
GGAATTACTGGGCGTAAAGCGCACGCAGGCGGTCTGTCAAGTCGGATGTGAAATCCCCGGGCTCAACCTGGGAACTGCATTCGAAACTGGCAGGCTAGAG
TCTTGTAGAGGGGGGTAGAATTCCAGGTGTAGCGGTGAAATGCGTAGAGATCTGGAGGAATACCGGTGGCGAAGGCGGCCCCCTGGACAAAGACTGACGC
TCAGGTGCGAAAGCGTGGGGAGCAAACAGGATTAGATACCCTGGTAGTCCACGCCGTAAACGATGTCGACTTGGAGGTTGTGCCCTTGAGGCGTGGCTTC
CGGAGCTAACGCGTTAAGTCGACCGCCTGGGGAGTACGGCCGCAAGGTTAAAACTCAAATGAATTGACGGGGGCCCGCACAAGCGGTGGAGCATGTGGTT
TAATTCGATGCAACGCGAAGAACCTTACCTACTCTTGACATCCAGAGAACTTAGCAGAGATGCTTTGGTGCCTTCGGGAACTCTGAGACAGGTGCTGCAT
GGCTGTCGTCAGCTCGTGTTGTGAAATGTTGGGTTAAGTCCCGCAACGAGCGCAACCCTTATCCTTTGTTGCCAGCGGTCCGGCCGGGAACTCAAAGGAG
ACTGCCAGTGATAAACTGGAGGAAGGTGGGGATGACGTCAAGTCATCATGGCCCTTACGAGTAGGGCTACACACGTGCTACAATGGCATATACAAAGAGA
AGCGACCTCGCGAGAGCAAGCGGACCTCATAAAGTATGTCGTAGTCCGGATTGGAGTCTGCAACTCGACTCCATGAAGTCGGAATCGCTAGTAATCGTAG
ATCAGAATGCTACGGTGAATACGTTCCCGGGCCTTGTACACACCGCCCGTCACACCATGGGAGTGGGTTGCAAAAGAAGTAGGTAGCTTAACCTTC

>EB136
TGCAAGTCGAGCGGTAGCACAGAGAGCTTGCTCTCGGGTGACGAGCGGCGGACGGGTGAGTAATGTCTGGGAAACTGCCTGATGGAGGGGGATAACTACT
GGAAACGGTAGCTAATACCGCATAACGTCGCAAGACCAAAGAGGGGGACCTTCGGGCCTCTTGCCATCAGATGTGCCCAGATGGGATTAGCTAGTAGGTG
GGGTAACGGCTCACCTAGGCGACGATCCCTAGCTGGTCTGAGAGGATGACCAGCCACACTGGAACTGAGACACGGTCCAGACTCCTACGGGAGGCAGCAG
TGGGGAATATTGCACAATGGGCGCAAGCCTGATGCAGCCATGCCGCGTGTATGAAGAAGGCCTTCGGGTTGTAAAGTACTTTCAGCGAGGAGGAAGGCAT
TAAGGTTAATAACCTTAGTGATTGACGTTACTCGCAGAAGAAGCACCGGCTAACTCCGTGCCAGCAGCCGCGGTAATACGGAGGGTGCAAGCGTTAATCG
GAATTACTGGGCGTAAAGCGCACGCAGGCGGTCTGTCAAGTCGGATGTGAAATCCCCGGGCTCAACCTGGGAACTGCATTCGAAACTGGCAGGCTAGAGT
CTTGTAGAGGGGGGTAGAATTCCAGGTGTAGCGGTGAAATGCGTAGAGATCTGGAGGAATACCGGTGGCGAAGGCGGCCCCCTGGACAAAGACTGACGCT
CAGGTGCGAAAGCGTGGGGAGCAAACAGGATTAGATACCCTGGTAGTCCACGCCGTAAACGATGTCGACTTGGAGGTTGTTCCCTTGAGGAGTGGCTTCC
GGAGCTAACGCGTTAAGTCGACCGCCTGGGGAGTACGGCCGCAAGGTTAAAACTCAAATGAATTGACGGGGGCCCGCACAAGCGGTGGAGCATGTGGTTT
AATTCGATGCAACGCGAAGAACCTTACCTACTCTTGACATCCACGGAATTTAGCAGAGATGCTTTAGTGCCTTCGGGAAACCGTGAGACAGGTGCTGCAT
GGCTGTCGTCAGCTCGTGTTGTGAAATGTTGGGTTAAGTCCCGCAACGAGCGCAACCCTTATCCTTTGTTGCCAGCGGTTCGGCCGGGAACTCAAAGGAG
ACTGCCAGTGATAAACTGGAGGAAGGTGGGGATGACGTCAAGTCATCATGGCCCTTACGAGTAGGGCTACACACGTGCTACAATGGCGCATACAAAGAGA
AGCGACCTCGCGAGAGCAAGCGGACCTCATAAAGTGCGTCGTAGTCCGGATTGGAGTCTGCAACTCGACTCCATGAAGTCGGAATCGCTAGTAATCGTAG
ATCAGAATGCTACGGTGAATACGTTCCCGGGCCTTGTACACACCGCCCGTCACACCATGGGAGTGGGTTGCAAAAGAAGTAGGTAGCTTAACCTTCGGGA
GGGCGC

>EB137
TGCAGTCGAGCGGTAGCACAGGGAGCTTGCTCCTGGGTGACGAGCGGCGGACGGGTGAGTAATGTCTGGGAAACTGCCTGATGGAGGGGGATAACTACTG
GAAACGGTAGCTAATACCGCATAACGTCGCAAGACCAAAGAGGGGGACCTTCGGGCCTCTTGCCATCAGATGTGCCCAGATGGGATTAGCTAGTAGGTGG
GGTAACGGCTCACCTAGGCGACGATCCCTAGCTGGTCTGAGAGGATGACCAGCCACACTGGAACTGAGACACGGTCCAGACTCCTACGGGAGGCAGCAGT
GGGGAATATTGCACAATGGGCGCAAGCCTGATGCAGCCATGCCGCGTGTATGAAGAAGGCCTTCGGGTTGTAAAGTACTTTCAGCGGGGAGGAAGGCGAT
AAGGTTAATAACCTCGTCGATTGACGTTACCCGCAGAAGAAGCACCGGCTAACTCCGTGCCAGCAGCCGCGGTAATACGGAGGGTGCAAGCGTTAATCGG
AATTACTGGGCGTAAAGCGCACGCAGGCGGTCTGTCAAGTCGGATGTGAAATCCCCGGGCTCAACCTGGGAACTGCATTCGAAACTGGCAGGCTAGAGTC
TTGTAGAGGGGGGTAGAATTCCAGGTGTAGCGGTGAAATGCGTAGAGATCTGGAGGAATACCGGTGGCGAAGGCGGCCCCCTGGACAAAGACTGACGCTC
AGGTGCGAAAGCGTGGGGAGCAAACAGGATTAGATACCCTGGTAGTCCACGCCGTAAACGATGTCGACTTGGAGGTTGTTCCCTTGAGGAGTGGCTTCCG
GAGCTAACGCGTTAAGTCGACCGCCTGGGGAGTACGGCCGCAAGGTTAAAACTCAAATGAATTGACGGGGGCCCGCACAAGCGGTGGAGCATGTGGTTTA
ATTCGATGCAACGCGAAGAACCTTACCTACTCTTGACATCCAGAGAACTTTCCAGAGATGGATTGGTGCCTTCGGGAACTCTGAGACAGGTGCTGCATGG
CTGTCGTCAGCTCGTGTTGTGAAATGTTGGGTTAAGTCCCGCAACGAGCGCAACCCTTATCCTTTGTTGCCAGCGGTTCGGCCGGGAACTCAAAGGAGAC
TGCCAGTGATAAACTGGAGGAAGGTGGGGATGACGTCAAGTCATCATGGCCCTTACGAGTAGGGCTACACACGTGCTACAATGGCGCATACAAAGAGAAG
CGACCTCGCGAGAGCAAGCGGACCTCATAAAGTGCGTCGTAGTCCGGATTGGAGTCTGCAACTCGACTCCATGAAGTCGGAATCGCTAGTAATCGTAGAT
CAGAATGCTACGGTGAATACGTTCCCGGGCCTTGTACACACCGCCCGTCACACCATGGGAGTGGGTTGCAAAAGAAGTAGGTAGCTTAACCTTCGGGAGG
GCGCTTACCA

>EB138

AGCGCCCTCCCGAAGGTTAAGCTACCTACTTCTTTTGCAACCCACTCCCATGGTGTGACGGGCGGTGTGTACAAGGCCCGGGAACGTATTCACCGTAGCA
TTCTGATCTACGATTACTAGCGATTCCGACTTCATGGAGTCGAGTTGCAGACTCCAATCCGGACTACGACGCACTTTATGAGGTCCGCTTGCTCTCGCGA
GGTCGCTTCTCTTTGTATGCGCCATTGTAGCACGTGTGTAGCCCTACTCGTAAGGGCCATGATGACTTGACGTCATCCCCACCTTCCTCCAGTTTATCAC
TGGCAGTCTCCTTTGAGTTCCCGGCCGAACCGCTGGCAACAAAGGATAAGGGTTGCGCTCGTTGCGGGACTTAACCCAACATTTCACAACACGAGCTGAC
GACAGCCATGCAGCACCTGTCTCAGAGTTCCCGAAGGCACCAAAGCATCTCTGCTAAGTTCTCTGGATGTCAAGAGTAGGTAAGGTTCTTCGCGTTGCAT
CGAATTAAACCACATGCTCCACCGCTTGTGCGGGCCCCCGTCAATTCATTTGAGTTTTAACCTTGCGGCCGTACTCCCCAGGCGGTCGACTTAACGCGTT
AGCTCCGGAAGCCACGCCTCAAGGGCACAACCTCCAAGTCGACATCGTTTACGGCGTGGACTACCAGGGTATCTAATCCTGTTTGCTCCCCACGCTTTCG
CACCTGAGCGTCAGTCTTTGTCCAGGGGGCCGCCTTCGCCACCGGTATTCCTCCAGATCTCTACGCATTTCACCGCTACACCTGGAATTCTACCCCCCTC
TACAAGACTCTAGCCTGCCAGTTTCGAATGCAGTTCCCAGGTTGAGCCCGGGGATTTCACATCCGACTTGACAGACCGCCTGCGTGCGCTTTACGCCCCA
GTAATTCCGATTAACGCTTGCACCCTCCGTATTACCGCGGCTGCTGGCACGGAGTTAGCCGGTGCTTCTTCTGCGAGTAACGTCAATCGCTGCGGTTATT
AACCACAACGCCTTCCTCCTCGCTGAAAGTACTTTACAACCCGAAGGCCTTCTTCATACACGCGGCATGGCTGCATCAGGCTTGCGCCCATTGTGCAATA
TTCCCCACTGCTGCCTCCCGTAGGAGTCTGGACCGTGTCTCAGTTCCAGTGTGGCTGGTCATCCTCTCAGACCAGCTAGGGATCGTCGCCTAGGTGAGCC
ATTACCCCACCTACTAGCTAATCCCATCTGGGCACATCCGATGGCAAGAGGCCCGAAGGTCCCCCTCTTTGGTCCGAAGACGTTATGCGGTATTAGCTAC
CGTTTCCAGTAGTTATCCCCCTCCATCGGGCAGTTTCCCAGACATTACTCACCCGTCCGCCGCTCGTCACCCGGAGAGCAAGCTCTCCTGTGCTACCGCT
CGAC

>EB139
CATGCAAGTCGAGCGGTAGCACAGAGAGCTTGCTCTCGGGTGACGAGCGGCGGACGGGTGAGTAATGTCTGGGAAACTGCCTGATGGAGGGGGATAACTA
CTGGAAACGGTAGCTAATACCGCATAACGTCGCAAGACCAAAGAGGGGGACCTTCGGGCCTCTTGCCATCAGATGTGCCCAGATGGGATTAGCTAGTAGG
TGGGGTAACGGCTCACCTAGGCGACGATCCCTAGCTGGTCTGAGAGGATGACCAGCCACACTGGAACTGAGACACGGTCCAGACTCCTACGGGAGGCAGC
AGTGGGGAATATTGCACAATGGGCGCAAGCCTGATGCAGCCATGCCGCGTGTATGAAGAAGGCCTTCGGGTTGTAAAGTACTTTCAGCGAGGAGGAAGGC
ATTGTGGTTAATAACCCCAGTGATTGACGTTACTCGCAGAAGAAGCACCGGCTAACTCCGTGCCAGCAGCCGCGGTAATACGGAGGGTGCAAGCGTTAAT
CGGAATTACTGGGCGTAAAGCGCACGCAGGCGGTCTGTCAAGTCGGATGTGAAATCCCCGGGCTCAACCTGGGAACTGCATTCGAAACTGGCAGGCTAGA
GTCTTGTAGAGGGGGGTAGAATTCCAGGTGTAGCGGTGAAATGCGTAGAGATCTGGAGGAATACCGGTGGCGAAGGCGGCCCCCTGGACAAAGACTGACG
CTCAGGTGCGAAAGCGTGGGGAGCAAACAGGATTAGATACCCTGGTAGTCCACGCCGTAAACGATGTCGACTTGGAGGTTGTTCCCTTGAGGAGTGGCTT
CCGGAGCTAACGCGTTAAGTCGACCGCCTGGGGAGTACGGCCGCAAGGTTAAAACTCAAATGAATTGACGGGGGGCCCGCACAAGCGGTGGAGCATGTGG
TTTAATTCGATGCAACGCGAAGAACCTTACCTACTCTTGACATCCACGGAATTTAGCAGAGATGCTTTAGTGCCTTCGGGAACCGTGAGACAGGTGCTGC
ATGGGCTGTCGTCAGCTCGTGTTGTGAAATGTTGGGTTAAGTCCCGCAACGAGCGCAACCCTTATCCTTTGTTGCCAGCGGTTCGGCCGGGAACTCAAAG
GAGACTGCCAGTGATAAACTGGAGGAAGGTGGGGATGACGTCAAGTCATCATGGCCCTTACGAGTAGGGCTACACACGTGCTACAATGGCGTATACAAAG
AGAAGCGACCTCGCGAGAGCAAGCGGACCTCATAAAGTACGTCGTAGTCCGGATTGGAGTCTGCAACTCGACTCCATGAAGTCGGAATCGCTAGTAATCG
TAGATCAGAATGCTACGGTGAATACGTTCCCGGGCCTTGTACACACCGCCCGTCACACCATGGGAGTGGGTTGCAAAAGAAGTAGGTAGCTTAACCTTCG
GGAGGGCGC

>EB140
GTCGAGCGGTAGCCACATAGCTTGCTCTCGGGTGACCAGCGGCGGACGGGTGAGTAATGTCTGGGAAACTGCCTGATGGAGGGGGATAACTACTGGAAAC
GGTACCTAATACCGCATAACGTCGCAAGACCAAAAAGGGGGACCTTCGGGCCTCTTGCCATCAGATGTGCCCAGATGGGATTAGCTAGTAGGTGGGGTAA
CGGCTCACCTAGGCGACGATCCCTAGCTGGTCTGAGAGGATGACCAGCCACACTGGAACTGAGACACGGTCCAGACTCCTACGGGAGGCAGCAGTGGGGA
ATATTGCACAATGGGCGCAAGCCTGATGCAGCCATGCCGCGTGTATGAAGAAGGCCTTCGGGTTGTAAAGTACTTTCAGCGAGGAGGAAGGCATTGTGGT
TAATAACCACAGTGATTGACGTTACTCGCAGAAGAAGCACCGGCTAACTCCGTGCCAGCAGCCGCGGTAATACGGAGGGTGCAAGCGTTAATCGGAATTA
CTGGGCGTAAAGCGCACGCATGCGGTCTGTCAAGTCGGATGTGAAATCCCCGGGCTCAACCTGGGAACTGCATTCGAAACTGGCAGGCTAGAGTCTTGTA
GAGGGGGGTAGAATTCCAGGTGTAGCGGTGAAATGCGTAGAGATCTGGAGGAATACCGGTGGCGAAGGCGGCCCCCTGGACAAAGACTGACGCTCAGGTG
CGAAAGCGTGGGGAGCAAACAGGATGAGATACCCTGGTAGTCCACACCGTAAACGATGTCTACTTGGAGGTTGTTCCCTTGAGGAGTGGCTTCCGGAGCT
AACGCGTTAAGTCGACCGCCTGGGGAGTACGGCCGCAAGGTTAAAACTCAAATGAATTGACCGGGGCCCGCACAAGCGGTGGAGCATGAGGTTTAATTCA
ATGCAACGCGAAGAACCTTACCTACTCTTGACATCCAAAGAAATTAGCAGAGATGCTTTGGTGCCTTCGGGAACTGTGAGACAGGTGCTGCATGGCTGCC
GTCAGCTCGTGTTGTGAAATGTTGGGTTAAGTCCCGCAACGAGCGCAACCCTTATCCTCTGTTGCCAGCGGTTCGGCCGGGAACTCAAAGGAGACTGCCA
GTGATAACCTGGAGGAAGGTGGGGATGACGTCAAGTCATCATGGCCCTTACGAGTAGGGCTACACACGTGCTACAATGGCGCATACAAAGAGAAGCGACC
TCGCGAGAGCAAGCGGACGCTCATAAAGTGCGTCGTAGTCCGGATTGGAGTCTGCAACTCGACTCCATGAAGTCGGAATCGCTAGTAATCGTAGATCAGA
ATGCTACGGTGAATACGTTCCCGGGCCTTGTACACACCGCCCGTCACACCATGGGAGTGGGTGCAAAAGAAGTAGGTAGCTTAACCTTCGGGAGGG

>EB141
TGCAAGTCGAGCGGTAGCACAGAGAGCTTGCTCTCGGGTGACGAGCGGCGGACGGGTGAGTAATGTCTGGGAAACTGCCTGATGGAGGGGGATAACTACT
GGAAACGGTAGCTAATACCGCATAACGTCGCAAGACCAAAGAGGGGGACCTTCGGGCCTCTTGCCATCAGATGTGCCCAGATGGGATTAGCTAGTAGGTG
GGGTAACGGCTCACCTAGGCGACGATCCCTAGCTGGTCTGAGAGGATGACCAGCCACACTGGAACTGAGACACGGTCCAGACTCCTACGGGAGGCAGCAG
TGGGGAATATTGCACAATGGGCGCAAGCCTGATGCAGCCATGCCGCGTGTATGAAGAAGGCCTTCGGGTTGTAAAGTACTTTCAGCGAGGAGGAAGGCAT
TGCGGTTAATAACCGCAGTGATTGACGTTACTCGCAGAAGAAGCACCGGCTAACTCCGTGCCAGCAGCCGCGGTAATACGGAGGGTGCAAGCGTTAATCG
GAATTACTGGGCGTAAAGCGCACGCAGGCGGTCTGTCAAGTCGGATGTGAAATCCCCGGGCTCAACCTGGGAACTGCATTCGAAACTGGCAGGCTAGAGT
CTTGTAGAGGGGGGTAGAATTCCAGGTGTAGCGGTGAAATGCGTAGAGATCTGGAGGAATACCGGTGGCGAAGGCGGCCCCCTGGACAAAGACTGACGCT
CAGGTGCGAAAGCGTGGGGAGCAAACAGGATTAGATACCCTGGTAGTCCACGCCGTAAACGATGTCGACTTGGAGGTTGTTCCCTTGAGGAGTGGCTTCC
GGAGCTAACGCGTTAAGTCGACCGCCTGGGGAGTACGGCCGCAAGGTTAAAACTCAAATGAATTGACGGGGGCCCGCACAAGCGGTGGAGCATGTGGTT
TAATTCGATGCAACGCGAAGAACCTTACCTACTCTTGACATCCAGAGAACTTAGCAGAGATGCTTTGGTGCCTTCGGGAACTCTGAGACAGGTGCTGCAT
GGCTGTCGTCAGCTCGTGTTGTGAAATGTTGGGTTAAGTCCCGCAACGAGCGCAACCCTTATCCTTTGTTGCCAGCGGTTCGGCCGGGAACTCAAAGGAG
ACTGCCAGTGATAAACTGGAGGAAGGTGGGGATGACGTCAAGTCATCATGGCCCTTACGAGTAGGGCTACACACGTGCTACAATGGCGCATACAAAGAGA
AGCGACCTCGCGAGAGCAAGCGGACCTCATAAAGTGCGTCGTAGTCCGGATTGGAGTCTGCAACTCGACTCCATGAAGTCGGAATCGCTAGTAATCGTAG
ATCAGAATGCTACGGTGAATACGTTCCCGGGCCTTGTACACACCGCCCGTCACACCATGGGAGTGGGTTGCAAAAGAAGTAGGTAGCTTAACCTTCGGGA
GGGCGCTA

>EB142
CGCCCTCCCGAAGGTTAAGCTACCTACTTCTTTTGCAACCCACTCCCATGGTGTGACGGGCGGTGTGTACAAGGCCCGGGAACGTATTCACCGTAGCATT
CTGATCTACGATTACTAGCGATTCCGACTTCATGGAGTCGAGTTGCAGACTCCAATCCGGACTACGACGCACTTTATGAGGTCCGCTTGCTCTCGCGAGG
TCGCTTCTCTTTGTATGCGCCATTGTAGCACGTGTGTAGCCCTACTCGTAAGGGCCATGATGACTTGACGTCATCCCCACCTTCCTCCAGTTTATCACTG
GCAGTCTCCTTTGAGTTCCCGGCCTAACCGCTGGCAACAAAGGATAAGGGTTGCGCTCGTTGCGGGACTTAACCCAACATTTCACAACACGAGCTGACGA
CAGCCATGCAGCACCTGTCTCAGAGTTCCCGAAGGCACCAATCCATCTCTGGAAAGTTCTCTGGATGTCAAGAGTAGGTAAGGTTCTTCGCGTTGCATCG
AATTAAACCACATGCTCCACCGCTTGTGCGGGCCCCCGTCAATTCATTTGAGTTTTAACCTTGCGGCCGTACTCCCCAGGCGGTCGACTTAACGCGTTAG
CTCCGGAAGCCACGCCTCAAGGGCACAACCTCCAAGTCGACATCGTTTACGGCGTGGACTACCAGGGTATCTAATCCTGTTTGCTCCCCACGCTTTCGCA
CCTGAGCGTCAGTCTTTGTCCAGGGGGCCGCCTTCGCCACCGGTATTCCTCCAGATCTCTACGCATTTCACCGCTACACCTGGAATTCTACCCCCCTCTA
CAAGACTCTAGCCTGCCAGTTTCGAATGCAGTTCCCAGGTTGAGCCCGGGGATTTCACATCCGACTTGACAGACCGCCTGCGTGCGCTTTACGCCCAGTA
ATTCCGATTAACGCTTGCACCCTCCGTATTACCGCGGCTGCTGGCACGGAGTTAGCCGGTGCTTCTTCTGCGGGTAACGTCAATTGGCTGAGGTTATTAA
CCTCAACACCCTTCCTCCCCCGCTGAAAAGTACTTTACAACCCGAAGGCCTTCTTCATACACGCGGCATGGCTGCATCAGGCTTGCGCCCACTGTGCAAT

ATTCCCCACTGCTGCCTCCCGTAGGAGTCTGGACCGTGTCTCAGTTCCAGTGTGGCTGGTCATCCTCTCAGACCAGCTAGGGATCGTCGCCTAGGTGAGC
CGTTACCCCACCTACTAGCTAATCCCATCTGGGCACATATGATGGCAAGAGGCCCGAAGGTCCCCCTCTTTGGTCTTGCGACGTTATGCGGTATTAGCTA
CCGTTTCCAGTAGTTATCCCCCTCCATCAGGCAGTTTCCCAGACATTACTCACCCGTCCGCCGCTCGCCGGCAAAGTAGCAAGCTACTTTCCGCTGCCGC
TCGACTGCATGTGTAGCC

>EB143
TGCAAGTCGAGCGGTAGCACAGGGAGCTTGCTCCTGGGTGACGAGCGGCGGACGGGTGAGTAATGTCTGGGAAACTGCCTGATGGAGGGGGATAACTACT
GGAAACGGTAGCTAATACCGCATAACGTCGCAAGACCAAAGAGGGGGACCTTCGGGCCTCTTGCCATCAGATGTGCCCAGATGGGATTAGCTAGTAGGTG
GGGTAACGGCTCACCTAGGCGACGATCCCTAGCTGGTCTGAGAGGATGACCAGCCACACTGGAACTGAGACACGGTCCAGACTCCTACGGGAGGCAGCAG
TGGGGAATATTGCACAATGGGCGCAAGCCTGATGCAGCCATGCCGCGTGTATGAAGAAGGCCTTCGGGGTTGTAAAGTACTTTCAGCGGGGAGGAAGGTG
TTGAGGTTAATAACCTCAGCCAATTGACGTTACCCGCAGAAAAAAGCACCGGCTAACTCCGTGCCCAGCAGCCGCGGTAATACGGAGGGTGCAAGCGTTA
ATCGGAATTACTGGGGCGTAAAGCGCACGCAGGCGGTCTGTCAAGTCGGATGTGAAATCCCCCGGGCTCAACCTGGGAACTGCATTCGAAACTGGCAGGC
TAGAGTCTTGTAGAGGGGGGTAGAATTCCAGGTGTAGCGGTGAAATGCGTAGAGATCTGGAGGAATACCGGTGGCGAAGGCGGCCCCCTGGACAAAGACT
GACGCTCAGGTGCGAAAGCGTGGGGAGCAAACAGGATTAGATACCCTGGTAGTCCACGCCGTAAACGATGTCGACTTGGAGGTTGTTCCCTTGAGGAGTG
GCTTCCGGAGCTAACGCGTTAAGTCGACCGCCTGGGGAGTACGGCCGCAAGGTTAAAACTCACATGAATTGACGGGGGCCCGCACAAGCGGTGGAGCATG
TGGTTTAATTCGATGCAACGCGAAGAACCTTACCTACTCTTGACATCCAGAGAACTTTTCCAGAGATGGCTTTGGTGCCTTCGGGAACTCTGAGACAGGT
GCTGCATGGCTGTCGTCAGCTCGTGTTGTGAAATGTTGGGTTAAGTCCCGCAACGAGCGCAACCCTTATCCTTTGTTGCCAGCGGTTCGGCCGGGAACTC
AAAGGAGACTGCCAGTGATAAACTGGAGGAAGGTGGGGATGACGTCAAGTCATCATGGCCCTTACGAGTAGGGCTACACACGTGCTACAATGGCGCATAC
AAAGAGAAGCGACCTCGCGAGAGCAAGCGGACCTCATAAAGTGCGTCGTAGTCCGGATTGGAGTCTGCAACTCGACTCCATGAAGTCGGAATCGCTAGTA
ATCGTAGATCAGAATGCTACGGTGAATACGTTCCCGGGCCTTGTACACACCGCCCGTCACACCATGGGAGTGGGTTGCAAAAGAAGTAGGTAGCTTAACC
TTCGGGAGGGCGCTACC

>EB144
GGTAGCGCCCTCCCGAAGGTTAAGCTACCTACTTCTTTTGCAACCCACTCCCATGGTGTGACGGGCGGTGTGTACAAGGCCCGGGAACGTATTCACCGTA
GCATTCTGATCTACGATTACTAGCGATTCCGACTTCATGGAGTCGAGTTGCAGACTCCAATCCGGACTACGACGCACTTTATGAGGTCCGCTTGCTCTCG
CGAGGTCGCTTCTCTTTGTATGCGCCATTGTAGCACGTGTGTAGCCCTACTCGTAAGGGCCATGATGACTTGACGTCATCCCCACCTTCCTCCAGTTTAT
CACTGGCAGTCTCCTTTGAGTTCCCGGCCGGACCGCTGGCAACAAAGGATAAGGGTTGCGCTCGTTGCGGGACTTAACCCAACATTTCACAACACGAGCT
GACGACAGCCATGCAGCACCTGTCTCAGAGTTCCCGAAGGCACCAATCCATCTCTGGAAAGTTCTCTGGATGTCAAGAGTAGGTAAGGTTCTTCGCGTTG
CATCGAATTAAACCACATGCTCCACCGCTTGTGCGGGCCCCCGTCAATTCATTTGAGTTTTAACCTTGCGGCCGTACTCCCCAGGCGGTCGACTTAACGC
GTTAGCTCCGGAAGCCACTCCTCAAGGGAACAACCTCCAAGTCGACATCGTTTACGGCGTGGACTACCAGGGTATCTAATCCTGTTTGCTCCCCACGCTT
TCGCACCTGAGCGTCAGTCTTTGTCCAGGGGGCCGCCTTCGCCACCGGTATTCCTCCAGATCTCTACGCATTTCACCGCTACACCTGGAATTCTACCCCC
CTCTACAAGACTCTAGCCTGCCAGTTTCGAATGCAGTTCCCAGGTTGAGCCCGGGGATTTCACATCCGACTTGACAGACCGCCTGCGTGCGCTTTACGCC
CAGTAATTCCGATTAACGCTTGCACCCTCCGTATTACCGCGGCTGCTGGCACGGAGTTAGCCGGTGCTTCTTCTGCGGGTAACGTCAATTGCTGAGGTTA
TTAACCTCAACACCTTCCTCCCCGCTGAAAGTACTTTACAACCCGAAGGCCTTCTTCATACACGCGGCATGGCTGCATCAGGCTTGCGCCCATTGTGCAA
TATTCCCCACTGCTGCCTCCCGTAGGAGTCTGGACCGTGTCTCAGTTCCAGTGTGGCTGGTCATCCTCTCAGACCAGCTAGGGATCGTCGCCTAGGTGAG
CCGTTACCCCACCTACTAGCTAATCCCATCTGGGCACATCTGATGGCAAGAGGCCCGAAGGTCCCCCTCTTTGGTCTTGCGACGTTATGCGGTATTAGCT
ACCGTTTCCAGTAGTTATCCCCCTCCATCAGGCAGTTTCCCAGACATTACTCACCCGTCCGCCGCTCGTCACCCAGGAGCAAGCTCCCTGTGCTACCGCT
CGAC

>EB145
CATGCAGTCGAACGGTAGCACAGAGGAGCTTGCTCCTTGGGTGACGAGTGGCGGACGGGTGAGTAATGTCTGGGAAACTGCCCGATGGAGGGGGATAACT
ACTGGAAACGGTAGCTAATACCGCATAACGTCGCAAGACCAAAGAGGGGGACCTTCGGGCCTCTTGCCATCGGATGTGCCCAGATGGGATTAGCTAGTAG
GTGGGGTAACGGCTCACCTAGGCGACGATCCCTAGCTGGTCTGAGAGGATGACCAGCCACACTGGAACTGAGACACGGTCCAGACTCCTACGGGAGGCAG
CAGTGGGGAATATTGCACAATGGGCGCAAGCCTGATGCAGCCATGCCGCGTGTATGAAGAAGGCCTTCGGGTTGTAAAGTACTTTCAGCGAGGAGGAAGG
TGTTGTGGTTAATAACCGCAGCAATTGACGTTACTCGCAGAAGAAGCACCGGCTAACTCCGTGCCAGCAGCCGCGGTAATACGGAGGGTGCAAGCGTTAA
TCGGAATTACTGGGCGTAAAGCGCACGCAGGCGGTCTGTCAAGTCGGATGTGAAATCCCCGGGCTCAACCTGGGAACTGCATCCGAAACTGGCAGGCTAG
AGTCTTGTAGAGGGGGGTAGAATTCCAGGTGTAGCGGTGAAATGCGTAGAGATCTGGAGGAATACCGGTGGCGAAGGCGGCCCCCTGGACAAAGACTGAC
GCTCAGGTGCGAAAGCGTGGGGAGCAAACAGGATTAGATACCCTGGTAGTCCACGCCGTAAACGATGTCGACTTGGAGGTTGTGCCCTTGAGGCGTGGCT
TCCGGAGCTAACGCGTTAAGTCGACCGCCTGGGGAGTACGGCCGCAAGGTTAAAACTCAAATGAATTGACGGGGGCCCGCACAAGCGGTGGAGCATGTG
GTTTAATTCGATGCAACGCGAAGAACCTTACCTACTCTTGACATCCAGAGAACTTAGCAGAGATGCTTTGGTGCCTTCGGGAACTCTGAGACAGGTGCTG
CATGGCTGTCGTCAGCTCGTGTTGTGAAATGTTGGGTTAAGTCCCGCAACGAGCGCAACCCTTATCCTTTGTTGCCAGCGGTTAGGCCGGGAACTCAAAG
GAGACTGCCAGTGATAAACTGGAGGAAGGTGGGGATGACGTCAAGTCATCATGGCCCTTACGAGTAGGGCTACACACGTGCTACAATGGCATATACAAAG
AGAAGCGACCTCGCGAGAGCAAGCGGACCTCATAAAGTATGTCGTAGTCCGGATTGGAGTCTGCAACTCGACTCCATGAAGTCGGAATCGCTAGTAATCG
TGGATCAGAATGCCACGGTGAATACGTTCCCGGGCCTTGTACACACCGCCCGTCACACCATGGGAGTGGGTTGCAAAAGAAGTAGGTAGCTTAACCTTCG
GGAGGGCGCTACC

>EB146
TCCTTACGGTTACTCCACCGACTTCGCTATGTTACAAACTCTCGTGGTGTGACGGGCGGTGTGTACAAGGCCCGGGAACGTATTCACCGCGGCATGCTGA
TCCGCGATTACTAACGATTCCAGCTTCATGTAGGCGAGTTGCAGCCTACAATCCGAACTGAGAATGGTTTTATGGGATTGGCTTGACCTCGCGGTCTTGC
AGCCCTTTGTACCATCCATTGTAGCACGTGTGTAGCCCAGGTCATAAGGGGCATGATGATTTGACGTCATCCCCACCTTCCTCCGGTTTGTCACCGGCAG
TCACCTTAGAGTGCCCAACTAAATGCTGGCAACTAAGATCAAGGGTTGCGCTCGTTGCGGGACTTAACCCAACATCTCACGACACGAGCTGACGACAACC
ATGCANCCACCTGTCACTCTGTCCCCCGAAGGGGAACGCTCTATCTCTAGAGTTGTCAGAGGATGTCAAGACCTGGTAAGGTTCTTCGCGTTGCTTCGAA
TTAAACCACATGCTCCACCGCTTGTGCGGGCCCCCGTCAATTCCTTTGAGTTTCAGTCTTGCGACCGTACTCCCCAGGCGGAGTGCTTAATGCGTTAGCT
GCAGCACTAAAGGGCGGAAACCCTCTAACACTTAGCACTCATCGTTTACGGCGTGGACTACCAGGGTATCTAATCCTGTTTGCTCCCCACGCTTTCGCGC
CTCAGCGTCAGTTACAGACCAAAAAGCCGCCTTCGCCACTGGTGTTCCTCCACATCTCTACGCATTTCACCGCTACACGTGGAATTCCGCTTTTCTCTTC
TGCACTCAAGTTCCCCAGTTTCCAATGACCCTCCACGGTTGAGCCGTGGGCTTTCACATCAGACTTAAGAAACCGCCTGCGCGCGCTTTACGCCCAATAA
TTCCGGATAACGCTTGCCACCTACGTATTACCGCGGCTGCTGGCACGTAGTTAGCCGTGGCTTTCTGGTTAGGTACCGTCAAGGTACGAGCAGTTACTCT
CGTACTTGTTCTTCCCTAACAACAGAGTTTTACGACCCGAAAGCCTTCATCACTCACGCGGCGTTGCTCCGTCAGACTTTCGTCCATTGCGGAAGATTCC
CTACTGCTGCCTCCCGTAGGAGTCTGGGCCGTGTCTCAGTCCCAGTGTGGCCGATCACCCTCTCAGGTCGGCTATGCATCGTTGCCTTGGTGAGCCGTTA
CCTCACCAACTAGCTAATGCACCGCGGGCCCATCTGTAAGTGATAGCCGAAACCATCTTTCAATCATCTCCCATGAAGGAGAAGATCCTATCCGGTATTA
GCTTCGGTTTCCCGAAGTTATCCCAGTCTTACAGGCAGGTTGCCCACGTGTTACTCACCCGTCCGCCGCTAACGTCATACGAAGCACAGCTTTTAATACA
GTTC

>EB147
CATGCAAGTCGAGCGGTAGCACAGAGAGCTTGCTCTCGGGTGACGAGCGGCGGACGGGTGAGTAATGTCTGGGAAACTGCCTGATGGAGGGGGATAACTA
CTGGAAACGGTAGCTAATACCGCATAACGTCGCAAGACCAAAGAGGGGGACCTTCGGGCCTCTTGCCATCAGATGTGCCCAGATGGGATTAGCTAGTAGG
TGGGGTAACGGCTCACCTAGGCGACGATCCCTAGCTGGTCTGAGAGGATGACCAGCCACACTGGAACTGAGACACGGTCCAGACTCCTACGGGAGGCAGC
AGTGGGGAATATTGCACAATGGGCGCAAGCCTGATGCAGCCATGCCGCGTGTATGAAGAAGGCCTTCGGGTTGTAAAGTACTTTCAGCGAGGAGGAAGGC

ATTGTGGTTAATAACCCCCAGTGATTGACGTTACTCGCAGAAGAAGCACCGGCTAACTCCGTGCCAGCAGCCGCGGTAATACGGAGGGTGCAAGCGTTAA
TCGGAATTACTGGGCGTAAAGCGCACGCAGGCGGTCTGTCAAGTCGGATGTGAAATCCCCGGGCTCAACCTGGGAACTGCATTCGAAACTGGCAGGCTAG
AGTCTTGTAGAGGGGGGTAGAATTCCAGGTGTAGCGGTGAAATGCGTAGAGATCTGGAGGAATACCGGTGGCGAAGGCGGCCCCCTGGACAAAGACTGAC
GCTCAGGTGCGAAAGCGTGGGGAGCAAACAGGATTAGATACCCTGGTAGTCCACGCCGTAAACGATGTCGACTTGGAGGTTGTTCCCTTGAGGAGTGGCT
TCCGGAGCTAACGCGTTAAGTCGACCGCCTGGGGAGTACGGCCGCAAGGTTAAAACTCAAATGAATTGACGGGGGCCCGCACAAGCGGTGGAGCATGTG
GTTTAATTCGATGCAACGCGAAGAACCTTACCTACTCTTGACATCCACAGAAATTTAGCAGAGATGCTTTGGTGCCTTCGGGAACTGTGAGACAGGTGCT
GCATGGCTGTCGTCAGCTCGTGTTGTGAAATGTTGGGTTAAGTCCCGCAACGAGCGCAACCCTTATCCTTTGTTGCCAGCGGTCCGGCCGGGAACTCAAA
GGAGACTGCCAGTGATAAACTGGAGGAAGGTGGGGATGACGTCAAGTCATCATGGCCCTTACGAGTAGGGCTACACACGTGCTACAATGGCGTATACAAA
GAGAAGCGACCTCGCGAGAGCAAGCGGACCTCATAAAGTACGTCGTAGTCCGGATTGGAGTCTGCAACTCGACTCCATGAAGTCGGAATCGCTAGTAATC
GTAGATCAGAATGCTACGGTGAATACGTTCCCGGGCCTTGTACACACCGCCCGTCACACCATGGGAGTGGGTTGCAAAAGAAGTAGGTAGCTTAACCTTC
GGGAGGGCGCT

>EB148
TGCAAGTCGGACGGTAGCACAGAGAGCTTGCTCTCGGGTGACGAGTGGCGGACGCGTGAGTAATGTCTGGGGATCTGCCCGATAGAGGGGGATAACCACT
GGAAACGGTGGCTAATACCGCATAACGTCGCAAGACCAAAGAGGGGGACCTTCGGGCCTCTCACTATCGGATGAACCCAGATGGGATTAGCTAGTAGGCG
GGGTAATGGCCCACCTAGGCGACAATCCCTAGCTGGTCTGAGAGGATGACCAGCCACACTGGAACTGAGACACGGTCCAGACTCCTACGGGAGGCAGCAG
TGGGGAATATTGCACAATGGGCGCAAGCCTGATGCACCCATGCCGCGTGTATGAAGAAGGCCTTCGGGTTGTAAAGTACTTTCAGCGGGGAGGAAGGCGA
TGCGGTTAATAACCGCGTCGATTGACGTTACCCGCAGAAAAAGCACCGGCTAACTCCGTGCCAGCAGCCGCGGTAATACGGAGGGTGCAAGCGTTAATCG
GAATTACTGGGCGTAAAGCGCACGCAGGCGGTCTGTTAAGTCAGATGTGAAATCCCCGGGCTTAACCTGGGAACTGCATTTGAAACTGGCAGGCTTGAGT
CTTGTAGAGGGGGGTAGAATTCCAGGTGTAGCGGTGAAATGCGTAGAGATCTGGAGGAATACCGGTGGCGAAGGCGGCCCCCTGGACAAAGACTGACGCT
CAGGTGCGAAAGCGTGGGGAGCAAACAGGATTAGATACCCTGGTAGTCCACGCCGTAAACGATGTCGACTTGGAGGTTGTTCCCCTGAGGAGTGGCTTCC
GGAGCTAACGCGTTAAGTCGACCGCCTGGGGAGTACGGCCGCAAGGTTAAAACTCAAATGAATTGACGGGGGCCCGCACAAGCGGTGGAGCATGTGGTTT
AATTCGATGCAACGCGAAGAACCTTACCTACTCTTGACATCCACGGAATTCGGCAGAGATGCCTTAGTGCCTTCGGGAACCGTGAGACAGGTGCTGCATG
GCTGTCGTCAGCTCGTGTTGTGAAATGTTGGGTTAAGTCCCGCAACGAGCGCAACCCTTATCCTTTGTTGCCAGCGATTCGGTCGGGAACTCAAAGGAGA
CTGCCGGTGATAAACCGGAGGAAGGTGGGGATGACGTCAAGTCATCATGGCCCTTACGAGTAGGGCTACACACGTGCTACAATGGCGCATACAAAGAGAA
GCGACCTCGCGAGAGCAAGCGGACCTCACAAAGTGCGTCGTAGTCCGGATCGGAGTCTGCAACTCGACTCCGTGAAGTCGGAATCGCTAGTAATCGTGGA
TCAGAATGCCACGGTGAATACGTTCCCGGGCCTTGTACACACCGCCCGTCACACCATGGGAGTGGGTTGCAAAAGAAGTAGGTAGCTTAACCTTCGGGAG
GGC

>EB149
TGCAAGTCGAACGGTAGCACAGAGAGCTTGCTCTCGGGTGACGAGTGGCGGACGGGTGAGTAATGTCTGGGAAACTGCCTGATGGAGGGGGATAACTACT
GGAAACGGTAGCTAATACCGCATAACGTCTTCGGACCAAAGTGGGGGACCTTCGGGCCTCACACCATCGGATGTGCCCAGATGGGATTAGCTAGTAGGTG
GGGTAATGGCTCACCTAGGCGACGATCCCTAGCTGGTCTGAGAGGATGACCAGCCACACTGGAACTGAGACACGGTCCAGACTCCTACGGGAGGCAGCAG
TGGGGAATATTGCACAATGGGCGCAAGCCTGATGCAGCCATGCCGCGTGTATGAAGAAGGCCTTCGGGTTGTAAAGTACTTTCAGCGGGGAGGAAGGCGA
CAGGGTTAATAACCCTGTCGATTGACGTTACCCGCAGAAGAAGCACCGGCTAACTCCGTGCCAGCAGCCGCGGTAATACGGAGGGTGCAAGCGTTAATCG
GAATTACTGGGCGTAAAGCGCACGCAGGCGGTCTGTCAAGTCAGATGTGAAATCCCCGGGCTTAACCTGGGAACTGCATTTGAAACTGGCAGGCTAGAGT
CTTGTAGAGGGGGGTAGAATTCCAGGTGTAGCGGTGAAATGCGTAGAGATCTGGAGGAATACCGGTGGCGAAGGCGGCCCCCTGGACAAAGACTGACGCT
CAGGTGCGAAAGCGTGGGGAGCAAACAGGATTAGATACCCTGGTAGTCCACGCCGTAAACGATGTCGACTTGGAGGTTGTGCCCTTGAGGCGTGGCTTCC
GGAGCTAACGCGTTAAGTCGACCGCCTGGGGAGTACGGCCGCAAGGTTAAAACTCAAATGAATTGACGGGGGCCCGCACAAGCGGTGGAGCATGTGGTT
TAATTCGATGCAACGCGAAGAACCTTACCTGGCCTTGACATCCACGGAATTCGGCAGAGATGCCTTAGTGCCTTCGGGAACCGTGAGACAGGTGCTGCAT
GGCTGTCGTCAGCTCGTGTTGTGAAATGTTGGGTTAAGTCCCGCAACGAGCGCAACCCTTATCCTTTGTTGCCAGCGAGTAATGTCGGGAACTCAAAGGA
GACTGCCGGTGACAAACCGGAGGAAGGTGGGGATGACGTCAAGTCATCATGGCCCTTACGGCCAGGGCTACACACGTGCTACAATGGCGCATACAAAGAG
AAGCGAACTCGCGAGAGCAAGCGGACCTCACAAAGTGCGTCGTAGTCCGGATCGGAGTCTGCAACTCGACTCCGTGAAGTCGGAATCGCTAGTAATCGTA
GATCAGAATGCTACGGTGAATACGTTCCCGGGCCTTGTACACACCGCCCGTCACACCATGGGAGTGGGTTGCAAAAGAAGTAGGTAGCTTAACCTTCGGG
AGGG

>EB150
TGCAGTCGAGCGGCAGCGGAAAGTAGCTTGCTACTTTGCCGGCGAGCGGCGGACGGGTGAGTAATGTCTGGGAAACTGCCTGATGGAGGGGGATAACTAC
TGGAAACGGTAGCTAATACCGCATGACCTCGAAAGAGCAAAGTGGGGGACCTTCGGGCCTCACGCCATCGGATGTGCCCAGATGGGATTAGCTAGTAGGT
GAGGTAATGGCTCACCTAGGCGACGATCCCTAGCTGGTCTGAGAGGATGACCAGCCACACTGGAACTGAGACACGGTCCAGACTCCTACGGGAGGCAGCN
AGTGGGGAATATTGCACAATGGGCGCAAGCCTGATGCAGCNCATGCCGCGTGTGTGAAGAAGGCCTTCGGGTTGTAAAGCACTTTCAGCGAGGAGGAAGG
CSTTTAAGGTTAATAACCTTAGTGATTGACGTTACTCGCAGAAGAAGCACCGGCTAACTCCGTGCCAGCAGCCGCGGTAATACGGAGGGTGCAAGCGTTA
ATCGGAATTACTGGGCGTAAAGCGCACGCAGGCGGTTTGTTAAGTCAGATGTGAAATCCCCGAGCTTAACTTGGGAACTGCATTTGAAACTGGCAAGCTA
GAGTCTTGTAGAGGGGGGTAGAATTCCAGGTGTAGCGGTGAAATGCGTAGAGATCTGGAGGAATACCGGTGGCGAAGGCGGCCCCCTGGACAAAGACTGA
CGCTCAGGTGCGAAAGCGTGGGGAGCAAACAGGATTAGATACCCTGGTAGTCCACGCTGTAAACGATGTCGATTTGGAGGTTGTGGCTTGACCCGTGGC
TTCCGGAGCTAACGCGTTAAATCGACCGCCTGGGGAGTACGGCCGCAAGGTTAAAACTCAAATGAATTGACGGGGGCCCGCACAAGCGGTGGAGCATGTG
GTTTAATTCGATGCAACGCGAAGAACCTTACCTACTCTTGACATCCAGAGAATTCGCTAGAGATAGCTTAGTGCCTTCGGGAACTCTGAGACAGGTGCTG
CATGGCTGTCGTCAGCTCGTGTTGTGAAATGTTGGGTTAAGTCCCGCAACGAGCGCAACCCTTATCCTTTGTTGCCAGCGCGTAATGGCGGGAACTCAAA
GGAGACTGCCGGTGATAAACCGGAGGAAGGTGGGGATGACGTCAAGTCATCATGGCCCTTACGAGTAGGGCTACACACGTGCTACAATGGCATATACAAA
GAGAAGCGAACTCGCGAGAGCAAGCGGACCTCATAAAGTATGTCGTAGTCCGGATTGGAGTCTGCAACTCGACTCCATGAAGTCGGAATCGCTAGTAATC
GTAGATCAGAATGCTACGGTGAATACGTTCCCGGGCCTTGTACACACCGCCCGTCACACCATGGGAGTGGGTTGCAAAAGAAGTAGGTAGCTTAACCTTC
GGGAGGGCGC

>EB151
GACGGAACTCTTCGGAGGGAAGGCAGTGGACTGAGCGGCGGACGGGTGAGTAACACGTAAGGAACCTGCCTCAAGGATTGGGATAACTCCGAGAAATCGG
AGCTAATACCGGATAGTTCATCGGACCGCATGGTCCGCTGATGAAAGGCGCTCCGGCGTCACCTTGAGATGGCCTTGCGGTGCATTAGCTAGTTGGTGGG
GTAACGGCCCACCAAGGCGACGATGCATAGCCGACCTGAGAGGGTGATCGGCCACACTGGGACTGAGACACGGCCCAGACTCCTACGGGAGGCAGCAGTA
GGGAATCTTCCACAATGGACGAAAGTCTGATGGAGCAACGCCGCGTGAGTGATGAAGGTTTTCGGATCGTAAAACTCTGTTGTAAGGGAAGAACACGTAC
GAGAGGAAATGCTCGTACCTTGACGGTACCTTACGAGAAAGCCACGGCTAACTACGTGCCAGCAGCCGCGGTAATACGTAGGTGGCAAGCGTTGTCCGGA
ATTATTGGGCGTAAAGCGCGCGCAGGCGGCCTTTTAAGTCTGATGTGAAAGCCCCCGGCTCAACCGGGGAGGGCCATTGGAAACTGGAAGGCTTGAGTAC
AGAAGAGAAGAGTGGAATTCCACGTGTAGCGGTGAAATGCGTAGAGATGTGGAGGAACACCAGTGGCGAAGGCGACTCTTTGGTCTGTAACTGACGCTGA
GGCGCGAAAGCGTGGGGAGCAAACAGGATTAGATACCCTGGTAGTCCACGCCGTAAAACGATGAGTGCTAGGTGTTGGGGGGTTTCCGCCCCTCAGTGCT
GAAGCTAACGCATTAAGCACTCCGCCTGGGGAGTACGGCCGCAAGGCTGAAACTCAAAGGAATTGACGGGGACCCGCACAAGCGGTGGAGCATGTGGTTT
AATTCGAAGCAACGCGAAGAACCTTACCAACTCTTGACATCCCATTGACCGCTTGAGAGATCAAGTTTTCCCTTCGGGGACAATGGTGACAGGTGGTGCA
TGGTTGTCGTCAGCTCGTGTCGTGAGATGTTGGGTTAAGTCCCGCAACGAGCGCAACCCCTATCCTTAGTTGCCAGCATTTAGTTGGGCACTCTAGGGAG
ACTGCCGGTGACAAACCGGAGGAAGGTGGGGATGACGTCAAATCATCATGCCCCTTATGAGTTGGGCTACACACGTGCTACAATGGACGGTACAAAGGGC
AGCGAGACCGCGAGGTGGAGCCAATCCCATAAAGCCGTTCCCAGTTCGGATTGCAGGCTGCAACTCGCCTGCATGAAGTCGGAATCGCTAGTAATCGCAG
GTCAGCATACTGCGGTGAATACGTTCCCGGGTCTTGTACACACCGCCCGTCACACCACGAGAGTTTGCAACACCCGAAGCCGGTGAGGTAACCGCAAGGA

>EB152
GGCTGGCTCCCTAAGGTTACCTCAACCGACTTCGGGTGTTACAAACTCTCGTGGTGTGACGGGCGGTGTGTACAAGACCCGGGAACGTATTCACCGCAGT
ATGCTGACCTGCGATTACTAGCGATTCCGACTTCATGCAGGCGAGTTGCAGCCTGCAATCCGAACTGAGAACGGCTTTCTGGGATTGGCTCCACCTCGCG
GCTTCGCTGCCCTTTGTACCGTCCATTGTAGCACGTGTGTAGCCCAACTCATAAGGGGCATGATGATTTGACGTCATCCCCACCTTCCTCCGGTTTGTCA
CCGGCAGTCTCCTTAGAGTGCCCAACTGAATGCTGGCAACTAAGGACAAGGGTTGCGCTCGTTGCGGGACTTAACCCAACATCTCACGACACGAGCTGAC
GACAACCATGCACCACCTGTCACCCCTGCCCCCGAAGGGGAAGGTACATCTCTGTACCGGTCAGGGGGATGTCAAGAGTTGGTAAGGTTCTTCGCGTTGC
TTCGAATTAAACCACATGCTCCACCGCTTGTGCGGGTCCCCGTCAATTCCTTTGAGTTTCAGCCTTGCGACCGTACTCCCCAGGCGGAGTGCTTAATGCG
TTAGCTTCAGCACTGAAGGGCGGAAACCCTCCAACACCTAGCACTCATCGTTTACGGCGTGGACTACCAGGGTATCTAATCCTGTTTGCTCCCCACGCTT
TCGCGCCTCAGCGTCAGTTATAGGCCAAAGAGTCGCCTTCGCCACTGGTGTTCCTCCACATCTCTACGCATTTCACCGCTACACGTGGAATTCCACTCTT
CTCTCCTATACTCAAGCCTCCCAGTTTCCAATGGCCCTCCCCGGTTGAGCCGGGGGCTTTCACATCAGACTTAAGAGGCCGCCTGCGCGCGCTTTACGCC
CAATAATTCCGGACAACGCTTGCCACCTACGTATTACCGCGGCTGCTGGCACGTAGTTAGCCGTGGCTTTCTCGCAAGGTACCGTCAAGGTGCCGCCATT
GCCTGCGGCACTTGTTCTTCCCTTACAACAGAACTTTACGACCCGAAGGCCTTCATCGTTCACGCGGCGTTGCTCCATCAGACTTTCGTCCATTGTGGAA
GATTCCCTACTGCTGCCTCCCGTAGGAGTCTGGGCCGTGTCTCAGTCCCAGTGTGGCCGATCACCCTCTCAGGTCGGCTATGCATCGTCGCCTTGGTGGG
CCATTACCCCACCAACTAGCTAATGCACCGCAAAGCCATCCCCAGGCGACGCCGAAGCGCCTTTCATCCTCGGACCATGCGGTCCGATGACCCATCCGGT
ATTAGCCCCGATTTCTCGTGGTTATCCCAGACCTGAGGGCAGGTTCTTTACGTGTTACTCACCCGTCCGCCGCTCATTCCGTCGACTTCCCCCCGAAGGG
TTCCGTCGATTCCTGCGCTCGAC

>EB153
CGGCTGGCTCCCTAAGGTTACCTCACCGACTTCGGGTGTTACAAACTCTCGTGGTGTGACGGGCGGTGTGTACAAGACCCGGGAACGTATTCACCGCAGT
ATGCTGACCTGCGATTACTAGCGATTCCGACTTCATGCAGGCGAGTTGCAGCCTGCAATCCGAACTGAGAACGGCTTTCTGGGATTGGCTCCACCTCGCG
GCTTCGCTGCCCTTTGTACCGTCCATTGTAGCACGTGTGTAGCCCAACTCATAAGGGGCATGATGATTTGACGTCATCCCCACCTTCCTCCGGTTTGTCA
CCGGCAGTCTCCTTAGAGTGCCCAACCAAATGCTGGCAACTAAGGACAAGGGTTGCGCTCGTTGCGGGACTTAACCCAACATCTCACGACACGAGCTGAC
GACAACCATGCACCACCTGTCACCCCTGCCCCCCGAAGGGGAAGGTACATCTCTGTACCGGTCAGGGGGATGTCAAGAGTTGGTAAGGTTCTTCGCGTTG
CTTCGAATTAAACCACATGCTCCACCGCTTGTGCGGGTCCCCCGTCAATTCCTTTGAGTTTCAGCCTTGCGACCGTACTCCCCAGGCGGAGTGCTTAATG
CGTTAGCTTCAGCACTGAAGGGCGGAAACCCTCCAACACCTAGCACTCATCGTTTACGGCGTGGACTACCAGGGTATCTAATCCTGTTTGCTCCCCACGC
TTTCGCGCCTCAGCGTCAGTTATAGGCCAAAGAGTCGCCTTCGCCACTGGTGTTCCTCCACATCTCTACGCATTTCACCGCTACACGTGGAATTCCACTC
TTCTCTCCTATACTCAAGCCTCCCAGTTTCCAATGGCCCTCCCCGGTTGAGCCGGGGGCTTTCACATCAGACTTAAGAGGCCGCCTGCGCGCGCTTTACG
CCCAATAATTCCGGACAACGCTTGCCACCTACGTATTACCGCGGCTGCTGGCACGTAGTTAGCCGTGGCTTTCTCGCAAGGTACCGTCAAGGTGCCGCCA
TTGCCTGCGGCACTTGTTCTTCCCTTACAACAGAACTTTACGACCCGAAGGCCTTCATCGTTCACGCGGCGTTGCTCCATCAGACTTTCGTCCATTGTGG
AAGATTCCCTACTGCTGCCTCCCGTAGGAGTCTGGGCCGTGTCTCAGTCCCAGTGTGGCCGATCACCCTCTCAGGTCGGCTATGCATCGTCGCCTTGGTG
GGCCATTACCCCACCAACTAGCTAATGCACCGCAAAGCCATCCCCAGGCGACGCCGAAGCGCCTTTCATCCTCGGACCATGCGGTCCGATGACCCATCCG
GTATTAGCCCCGATTTCTCGTGGTTATCCCAGACCTGAGGGCAGGTTCTTTACGTGTTACTCACCCGTCCGCCGCTCATTCCGTCGACTTCCCCCCGAAG
GGTTCCGTATGATTTCCTGCGCTCGACTGCA

>EB154
GTCGTAACAAGGTAACCGTAGAGTTTGATTATGGCTCAGTAAGTCGTAACAAGGTAACCGTGCAGTTTGATCAGGGATCCTTCGGCAAACCGAAGCTAAT
ACCGGATTTGATCTTCTCCTTCATGGGAGATGATTGAAAGATGGTTTCGGCTATCACTTACAGATGGGCCCGCGGTGCATTAACTAGTTGGTGAGGTAAC
GGCTCACCAAGGCAACGATGCATAGCCGACCTGAGAGGGTGATCGGCCACACTGGGACTGAGACACGGNCCCAGAYTCCTACGGGAGGCAGCAGTAGGGA
ATCTTCCGCAATGGACGAAAGTCTGACGGAGCAACGCCGCGTGAGTGATGAAGGCTTTCGGGTCGTAAAACTCTGTTGTTAGGGAAGAACAAGTACGAGA
GTAACTGCTCGTACCTTGACGGTACCTAACCAGAAAGCCACGGCTAACTACGTGCCAGCAGCCGCGGTAATACGTAGGTGGCAAGCGTTATCCGGAATTA
TTGGGCGTAAAGCGCGCGCAGGCGGTTTCTTAAGTCTGATGTGAAAGCCCACGGCTCAACCGTGGAGGGTCATTGGAAACTGGGGAACTTGAGTGCAGAA
GAGAAAAGCGGAATTCCACGTGTAGCGGTGAAATGCGTAGAGATGTGGAGGAACACCAGTGGCGAAGGCGGCTTTTTGGTCTGTAACTGACGCTGAGGCG
CGAAAGCGTGGGGAGCAAACAGGATTAGATACCCTGGTAGTCCACGCCGTAAACGATGAGTGCTAAGTGTTAGAGGGTTTCCGCCCTTTAGTGCTGCAGC
TAACGCATTAAGCACTCCGCCTGGGGAGTACGGTCGCAAGACTGAAACTCAAAGGAATTGACGGGGGCCCGCACAAGCGGTGGAGCATGTGGTTTAATTC
GAAGCAACGCGAAGAACCTTACCAGGTCTTGACATCCTCTGACAACTCTAGAGATAGAGCGTTCCCCTTCGGGGGACAGAGTGACAGGTGGTGCATGGTT
GTCGTCAGCTCGTGTCGTGAGATGTTGGGTTAAGTCCCGCAACGAGCGCAACCCTTGATCTTAGTTGCCAGCATTTAGTTGGGCACTCTAAGGTGACTGC
CGGTGACAAACCGGAGGAAGGTGGGGATGACGTCAAATCATCATGCCCCTTATGACCTGGGCTACACACGTGCTACAATGGATGGTACAAAGGGCTGCAA
GACCGCGAGGTCAAGCCAATCCCATAAAACCATTCTCAGTTCGGATTGTAGGCTGCAACTCGCCTACATGAAGCTGGAATCGTTAGTAATCGCGGATCAG
CATGACGCGGTGAATACGTTCCCGGGCCTTGTACACACCGCCCGTCACGTCG

>EB155
TGGCTCAGGATGAACGCTGGCGGCGTGCTTAACACATGCAAGTCGAACGGTGAAGCCAAGCTTGCTTGGTGGATCAGTGGCGAACGGGTGAGTAACACGT
GAGCAACCTGCCCTGGACTCTGGGATAAGCGCTGGAAACGGTGTCTAATACTGGATATGAGCCTCTATCGCATGGTGGGGGTTGGAAAGATTTTTCGGTC
TGGGATGGGCTCGCGGCCTATCAGCTTGTTGGTGAGGTAATGGCTCACCAAGGCGTCGACGGGTAGCCGGCCTGAGAGGGTGACCGGCCACACTGGGACT
GAGACACGGCCCAGACTCCTACGGGAGGCAGCAGTGGGGAATATTGCACAATGGGCGGAAGCCTGATGCAGCAACGCCGCGTGAGGGATGACGGCCTTCG
GGTTGTAAACCTCTTTTAGCAGGGAAGAAGCGAAAGTGACGGTACCTGCAGAAAAAGCGCCGGCTAACTACGTGCCAGCAGCCGCGGTAATACGTAGGGC
GCAAGCGTTATCCGGAATTATTGGGCGTAAAGAGCTCGTAGGCGGTTTGTCGCGTCTGCTGTGAAATCCCGAGGCTCAACCTCGGGTCTGCAGTGGGTAC
GGGCAGACTAGAGTGCGGTAGGGGAGATTGGAATTCCTGGTGTAGCNGGTGGAATGCGCAGATATCAGGAGGAACACCGATGGCGAAGGCAGATCTCTGG
GCCGTAACTGACGCTGAGGAGCGAAAGGGTGGGGAGCAAACAGGCTTAGATACCCTGGTAGTCCACCCCGTAAACGTTGGGAACTAGTTGTGGGGACCAT
TCCACGGTTTCCGTGACGCAGCTAACGCATTAAGTTCCCCGCCTGGGGAGTACGGCCGCAAGGCTAAAACTCAAAGGAATTGACGGGGACCCGCACAAGC
GGCGGAGCATGCGGATTAATTCGATGCAACGCGAAGAACCTTACCAAGGCTTGACATACACCAGAACACCGTAGAAATACGGGACTCTTTGGACACTGGT
GAACAGGTGGTGCATGGTTGTCGTCAGCTCGTGTCGTGAGATGTTGGGTTAAGTCCCGCAACGAGCGCAACCCTCGTTCTATGTTGCCAGCACGTAATGG
TGGGAACTCATGGGATACTGCCGGGGTCAACTCGGAGGAAGGTGGGGATGACGTCAAATCATCATGCCCCTTATGTCTTGGGCTTCACGCATGCTACAAT
GGCCGGTACAAAGGGCTGCAATACCGTGAGGTGGAGCGAATCCCAAAAAGCCGGTCCCAGTTCGGATTGAGGTCTGCAACTCGACCTCATGAAGTCGGAG
TCGCTAGTAATCGCAGATCAGCAACGCTGCGGTGAATACGTTCCCGGGTCTTGTACACACCGCCCGTCAAGTCATGAAAGTCGGTAACACCTGAAGCCGG
TGGCCCAACCCTTGTGGAGGGAGCCGTCCAAGGTGGGATCGGTAATTAGGACTAGTCGAAAAA

>EB156
GACGTTGGCGGCGGACGGGTGAGTAACACGTGGGCAACCTGCCTGTAAGACTGGGATAACTTCGGGAAACCGAAGCTAATACCGGATAGGATCTTCTCCT
TCATGGGAGATGATTGAAAGATGGTTTCGGCTATCACTTACAGATGGGCCCGCGGTGCATTAGCTAGTTGGTGAGGTAACGGCTCACCAAGGCAACGATG
CATAGCCGACCTGAGAGGGTGATCGGCCACACTGGGACTGAGACACGGCCCAGACTCCTACGGGAGGCAGCAGTAGGGAATCTTCCGCAATGGACGAAAG
TCTGACGGAGCAACGCCGCGTGAGTGATGAAGGCTTTCGGGTCGTAAAACTCTGTTGTTAGGGAAGAACAAGTACGAGAGTAACTGCTCGTACCTTGACG
GTACCTAACCAGAAAGCCACGGCTAACTACGTGCCAGCAGCCGCGGTAATACGTAGGTGGCAAGCGTTATCCGGAATTATTGGGCGTAAAGCGCGCGCAG
GCGGTTTCTTAAGTCTGATGTGAAAGCCCACGGCTCAACCGTGGAGGGTCATTGGAAACTGGGGAACTTGAGTGCAGAAGAGAAAAGCGGAATTCCACGT
GTAGCGGTGAAATGCGTAGAGATGTGGAGGAACACCAGTGGCGAAGGCGGCTTTTTGGTCTGTAACTGACGCTGAGGCGCGAAAGCGTGGGGAGCAAACA
GGATTAGATACCCTGGTAGTCCACGCCGTAAACGATGAGTGCTAAGTGTTAGAGGGTTTCCGCCCTTTAGTGCTGCAGCTAACGCATTAAGCACTCCGCC
TGGGGAGTACGGTCGCAAGACTGAAACTCAAAGGAATTGACGGGGGCCCGCACAAGCGGTGGAGCATGTGGTTTAATTCGAAGCAACGCGAAGAACCTTA

CCAGGTCTTGACATCCTCTGACAACTCTAGAGATAGAGCGTTCCCCTTCGGGGGACAGAGTGACAGGTGGTGCATGGTTGTCGTCAGCTCGTGTCGTGAG
ATGTTGGGTTAAGTCCCGCAACGAGCGCAACCCTTGATCTTAGTTGCCAGCATTTAGTTGGGCACTCTAAGGTGACTGCCGGTGACAAACCGGAGGAAGG
TGGGGATGACGTCAAATCATCATGCCCCTTATGACCTGGGCTACACACGTGCTACAATGGATGGTACAAAGGGCTGCAAGACCGCGAGGTCAAGCCAATC
CCATAAAACCATTCTCAGTTCGGATTGTAGGCTGCAACTCGCCTACATGAAGCTGGAATCGCTAGTAATCGCGGATCAGCATGCCGCGGTGAATACGTTC
CCGGGCCTTGTACACACCGCCCGTCACACCACGAGAGTTTGTAACATAGAGAAGTCGGTGGAGTAACCGTAAGG

>EB157
TTCTGCTCAGGATGAACGCTGGCGGCGTGCCTAATACATGCAAGTCGAGCGAACTGATTAGAAGCTTGCTTCTATGACGTTAGCGGCGGACGGGTGAGTA
ACACGTGGGCAACCTGCCTGTAAGACTGGGATAACTTCGGGAAACCGAAGCTAATACCGGATAGGATCTTCTCCTTCATGGGAGATGATTGAAAGATGGT
TTCGGCTATCACTTACAGATGGGCCCGCGGTGCATTAGCTAGTTGGTGAGGTAACGGCTCACCAAGGCAACGATGCATAGCCGACCTGAGAGGGTGATCG
GCCACACTGGGACTGAGACACGGCCCAGACTCCTACGGGAGGCAGCAGTAGGGAATCTTCCGCAATGGACGAAAGTCTGACGGAGCAACGCCGCGTGAGT
GATGAAGGCTTTCGGGTCGTAAAACTCTGTTGTTAGGGAAGAACAAGTACGAGAGTAACTGCTCGTACCTTGACGGTACCTAACCAGAAAGCCACGGCTA
ACTACGTGCCAGCAGCCGCGGTAATACGTAGGTGGCAAGCGTTATCCGGAATTATTGGGCGTAAAGCGCGCGCAGGCGGTTTCTTAAGTCTGATGTGAAA
GCCCACGGCTCAACCGTGGAGGGTCATTGGAAACTGGGGAACTTGAGTGCAGAAGAGAAAAGCGGAATTCCACGTGTAGCGGTGAAATGCGTAGAGATGT
GGAGGAACACCAGTGGCGAAGGCGGCTTTTTGGTCTGTAACTGACGCTGAGGCGCGAAAGCGTGGGGAGCAAACAGGATTAGATACCCTGGTAGTCCACG
CCGTAAACGATGAGTGCTAAGTGTTAGAGGGTTTCCGCCCTTTAGTGCTGCAGCTAACGCATTAAGCACTCCGCCTGGGGAGTACGGTCGCAAGACTGAA
ACTCAAAGGAATTGACGGGGGCCCGCACAAGCGGTGGAGCATGTGGTTTAATTCGAAGCAACGCGAAGAACCTTACCAGGTCTTGACATCCTCTGACAAC
TCTAGAGATAGAGCGTTCCCCTTCGGGGGACAGAGTGACAGGTGGTGCATGGTTGTCGTCAGCTCGTGTCGTGAGATGTTGGGTTAAGTCCCGCAACGAG
CGCAACCCTTGATCTTAGTTGCCAGCATTTAGTTGGGCACTCTAAGGTGACTGCCGGTGACAAACCGGAGGAAGGTGGGGATGACGTCAAATCATCATGC
CCCTTATGACCTGGGCTACACACGTGCTACAATGGATGGTACAAAGGGCTGCAAGACCGCGAGGTCAAGCCAATCCCATAAAACCATTCTCAGTTCGGAT
TGTAGGCTGCAACTCGCCTACATGAAGCTGGAATCGCTAGTAATCGCGGATCAGCATGCCGCGGTGAATACGTTCCCGGGCCTTGTACCAACCGCCCGTC
AAACCACGAGAATTTGTAACACCCGAAGTCGGTGGAATAACCGTAAGGAACTACCCCCCTAAGGGGGACAAATAATTGGGTGAA

>EB158
GGCAGCTCCTTGCGGTCACCGACTTCAGGTACCCCCAGCTTCCATGGCTTGACGGGCGGTGTGTACAAGGCCCGGGAACGTATTCACCGGATCATGGCTG
ATATCCGATTACTAGCGATTCCAGCTTCACGGAGTCGAGTTGCAGACTCCGATCCGAACTGAGAACGGTTTTATAGATTCGCATCCAGTCACCTGGTAGC
TGCTCTCTGTACCGTCCATTGTAGCACGTGTGTGGCCCAGGACGTAAGGGCCGTGATGATTTGACGTCATCCCCACCTTCCTCACAGTTTACACTGGCAG
TCTTGTTAGAGTTCCCGACATCACTCGCTGGCAACTAACAACAGGGGTTGCGCTCGTTATAGGACTTAANCCTGACACCTCACGGCACGAGCTGACGACA
ANCCATGCAGCACCTTGTAATTTTGTCTTGCGAAAGATCTGTTTCCAAATCGGTCAAACTACATTTAAGCCCTGGTAAGGTTCCTCGCGTATCATCGAAT
TAAACCACATGCTCCACCGCTTGTGCGGGCCCCCGTCAATTCCTTTGAGTTTCAGGCTTGCGCCCGTACTCCCCAGGTGGGATACTTATCACTTTCGCTT
AGCCACTCAAGCTTACGCCCAAACAGCTAGTATCCATCGTTTACGGCGTGGACTACCAGGGTATCTAATCCTGTTCGCTACCCACGCTTTCGTCCATCAG
CGTCAATCGTTTGTTAGTAACCTGCCTTCGCAATTGGTATTCCATGTAATATCTAAGCATTTCACCGCTACACTACATATTCTAGTTACTTCACAAAAAT
TCAAGCCCTACAGTATCAATGGCAATTTTTTGGTTAAGCCAAAAACTTTCACCGCTGACTTATAAGGCCGCCTACGGACCCTTTAAACCCAATGATTCCG
GATAACGCTTGGNATCCTCCGTATTACCGCGGNCTGCTGGCACGGAGTTAGCCGATCCTTATTCTTACGGTACCGTCAGTAAGCTACWCGTAGCTTTTTT
TNCTTCCCGTACAAAAGCAGTTTNACACACCATAGATGCTTCATCCTGCACGCGGCATGGCTGGATCAGGCTTCCGCCCATTGTCCAATATTCCTCACTG
CTGCCTCCCGTAGGAGTCTGGTCCGTGTCTCAGTACCAGTGTGGGGGATCTCCCTCTCAGGACCCCTACCCATCATCGTCTTGGTAAGCCGTTACCTTAC
CAACTAACTAATGGGACGCATGCTCATCTTATACCAATAAATCTTTATAATTAAAAAGATGCCGTTTTTATTAACCATAAGGTATTAATCCAAATTTCTC
TGGGCTATCCCTTAGTATAAGGTAGATTGCATACGCGTTACGCACCCGTGCGCCGGTCTCAAGCTCCGAAGACCATCTACCCCTCGACTTGCA

>EB159
GGCTCCCCCCACAAGGGTTGGGCCACCGGCTTCGGGTGTTACCGACTTTCGTGACTTGACGGGCGGTGTGTACAAGGCCCGGGAACGTATTCACCGCAGC
GTTGCTGATCTGCGATTACTAGCGACTCCAACTTCATGGGGTCGAGTTGCAGACCCCAATCCGAACTGAGACCAGTTTTAAGGGATTCGCTCCACCTCAC
GGTATCGCAACCCTCTGTACCAGCCATTGTAGCATGCGTGAAGCCCAAGACATAAGGGGCATGATGATTTGACGTCATCCCCACCTTCCTCCGAGTTGAC
CCCGGCAGTCTCCCATGAGTCCCCACCATTACGTGCTGGCAACATGGNAACGAGGGTTGCGCTCGTTGCGGGACTTAACCCAACATCTCACGACACGAGC
TGACGACAACCATGCACCACCTGTACACCAGCCCGAAGGCTGCACCATCTCTGGCACATTCCGGTGTATGTCAAGCCTTGGTAAGGTTCTTCGCGTTGCA
TCGAATTAATCCGCATGCTCCGCCGCTTGTGCGGGCCCCCGTCAATTCCTTTGAGTTTTAGCCTTGCGGCCGTACTCCCCAGGCGGGGAACTTAATGCGT
TAGCTACGGCACGGACCTCGTGGAATGAGGCCCACACCTAGTTCCCAACGTTTACGGCATGGACTACCAGGGTATCTAATCCTGTTCGCTCCCCATGCTT
TCGCTTCTCAGCGTCAGTAGTGGCCCAGAGACCTGCCTTCGCCATCGGTGTTCCTCCTGATATCTGCGCATTTCACCGCTACACCAGGAATTCCAGTCTC
CCCTACCACACTCTAGCCTGCCCGTACCCACCGCAGACCCAGGGTTAAGCCCTGGGCTTTCACGGCAGACGCGACAAACCGCCTACAAGCTCTTTACGCC
CAATAATTCCGGACAACGCTCGCACCCTACGTATTACCGCGGCTGCTGGCACGTAGTTAGCCGGTGCTTCTTCTGCAGGTACCGTCACAAAAAGCTTCGT
CCCTGCTGAAAGGAGTTTACAACCCGAAGGCCGTCATCCCCCACGCGGCGTCGCTGCATCAGGCTTCCGCCCATTGTGCAATATTCCCCACTGCTGCCTC
CCGTAGGAGTCTGGGCCGTGTCTCAGTCCCAGTGTGGCCGGTCACCCTCTCAGGCCGGCTACCCGTCAAAGCCATGGTAGGCCACTACCCCACCATCAAG
CTGATAGGCCGCGAGTCCATCCTCCACCACAAAAGCTTTCCACACACCACCATGCGACAATGTGTCATACCCGGTATTAGACCCCGTTTCCAAGGCTTAT
CCCAAAGTGAAGGGAAGGTTACTCACGTGTTACTCACCCGTTCGCCACTAATCCAAACCAGCAAGCTGGTCCTTCATCGTTCGAC

>EB160
TAGCTCCTTACGGTTACTCCACCGACTTCGCTAGTTACAAACTCTCGTGGTGTGACGGGCGGTGTGTACAAGGCCCGGGAACGTATTCACCGCGGCATGC
TGATCCGCGATTACTAGCGATTCCAGCTTCATGTAGGCGAGTTGCAGCCTACAATCCGAACTGAGAATGGTTTTATGGGATTGGCTTGACCTCGCGGTCT
TGCAGCCCTTTGTACCATCCATTGTAGCACGTGTGTAGCCCAGGTCATAAGGGGCATGATGATTTGACGTCATCCCCACCTTCCTCCGGTTTGTCACCGG
CAGTCACCTTAGAGTGCCCAACTAAATGCTGGCAACTAAGATCAAGGGTTGCGCTCGTTGCGGGACTTAACCCAACATCTCACGACACGAGCTGACGACA
ACCATGCACCACCTGTCACTCTGTCCCCCGAAGGGGAACGCTCTATCTCTAGAGTTGTCAGAGGATGTCAAGACCTGGTAAGGTTCTTCGCGTTGCTTCG
AATTAAACCACATGCTCCACCGCTTGTGCGGGCCCCCGTCAATTCCTTTGAGTTTCAGTCTTGCGACCGTACTCCCCAGGCGGAGTGCTTAATGCGTTAG
CTGCAGCACTAAAGGGCGGAAACCCTCTAACACTTAGCACTCATCGTTTACGGCGTGGACTACCAGGGTATCTAATCCTGTTTGCTCCCCACGCTTTCGC
GCCTCAGCGTCAGTTACAGACCAAAAAGCCGCCTTCGCCACTGGTGTTCCTCCACATCTCTACGCATTTCACCGCTACACGTGGAATTCCGCTTTTCTCT
TCTGCACTCAAGTTCCCCAGTTTCCAATGACCCTCCACGGTTGAGCCGTGGGCTTTCACATCAGACTTAAGAAACCGCCTGCGCGCGCTTTACGCCCAAT
AATTCCGGATAACGCTTGCCACCTACGTATTACCGCGGCTGCTGGCACGTAGTTAGCCGTGGCTTTCTGGTTAGGTACCGTCAAGGTACGAGCAGTTACT
CTCGTACTTGTTCTTCCCTAACAACAGAGTTTTACGACCCGAAAGCCTTCATCACTCACGCGGCGTTGCTCCGTCAGACTTTCGTCCATTGCGGAAGATT
CCCTACTGCTGCCTCCCGTAGGAGTCTGGGCCGTGTCTCAGTCCCAGTGTGGCCGATCACCCTCTCAGGTCGGCTATGCATCGTTGCCTTGGTGAGCCGT
TACCTCACCAACTAGCTAATGCACCGCGGGCCCATCTGTAAGTGATAGCCGAAACCATCTTTCAATCATCTCCCATGAAGGAGAAGATCCTATCCGGTAT
TAGCTTCGGTTTCCCGAAGTTATCCCAGTCTTACAGGCAGGTTGCCCACGTGTTACTCACCCGTCCGCCGCTAACGTCATAGAAGCAAGC

>EB161
CAAGGGGTTAGGCCACCGGCTTCGGGTGTTACCGACTTTCATGACGTGACGGGCGGTGTGTACAAGGCCCGGGAACGTATTCACCGCAGCGTTGCTGATC
TGCGATTACTAGCGACTCCGACTTCATGGGGTCGAGTTGCAGACCCCAATCCGAACTGAGACTGGCTTTAAGGGATTCGCTCCACCTCACGGTATCGCAG
CCCTCTGTACCAGCCATTGTAGCATGTGTGAAGCCCTGGACATAAGGGGCATGATGACTTGACGTCATCCCCACCTTCCTCCGAGTTGACCCCGGCAGTC
TCCTGCAAGTCCCCGGCATAACCCGCTGGCAATACAGGACAAGGGTTGCGCTCGTTGCGGGACTTAACCCAACATCTCACGACACGAGCTGACGACAGCC
ATGCACCACCTGTACACCAACCACAAGGGAACATGTATCTCTACATGCGTCTGGTGTATGTCAAACCCAGGTAAGGTTCTTCGCGTTGCATCGAATTAAT
CCCACATGCTCCGCCGCTTGTGCGGGCCCCCGTCAATTCCTTTGAGTTTTAGCCTTGCGGCCGTACTCCCCAGGCGGGGTACTTAATGCGTTAGCTACGG

CACGGAACTCGTGAAATGAGCCCCACACCTAGTACCCACCGTTTACGGCGTGGACTACCAGGGTATCTAATCCTGTTCGCTACCCACGCTTTCGCTCCTC
AGCGTCAGTTACTACCCAGAGACCCGCCTTCGCCACCGGTGTTCCTCCTGATATCTGCGCATTTCACCGCTACACCAGGAATTCCAGTCTCCCCTGTAGT
ACTCAAGTCTGCCCGTATCGCCTGCACGCCTACAATTGAGTTGCAGAATTTCACAGACGACGCGACAAACCGCCTACGAGCTCTTTACGCCCCAGTAATT
CCGGACAACGCTCGCACCCTACGTATTACCGCGGCTGCTGGCACGTAGTTGGCCGGTGCTTCTTCTCCAGGTACCGTCACTTGCGCTTCGTCCCTGGTGA
AAGAGGTTTACAACCCGAAGGCCGTCATCCCTCACGCGGCGTCGCTGCATCAGGCTTGCGCCCATTGTGCAATATTCCCCACTGCTGCCTCCCGTAGGAG
TCTGGGCCGTGTCTCAGTCCCAGTGTGGCCGATCACCCTCTCAGGTCGGCTACCCGTCGTCGCCTTGGTAGGCCATTACCCCACCAACAAGCTGATAGGC
CGCGGGCCCATCCTGAACCGCAAAAGCTTTCCACCCCAGAGCATGCACTCCAAGGTCATATCCGGTATTAGACCCAGTTTCCCAGGCTTATCCCAAAGTT
CAGGGCAGATCACCCACGTGTTACTCACCCGTTCGCCACTCGAGTACCCAGCAAGCTGGGCCTTTCCGTTCGACTGCA

>EB162
TGCAGTCGAACGGAAAGGCCCAGCTTGCTGGGTACTCGAGTGGCGAACGGGTGAGTAACACGTGGGTGATCTGCCCTGCACTCTGGGATAAGCCTGGGAA
ACTGGGTCTAATACCGGATATGACCAACTGTCGCATGGTGGTTGGTGGAAAGCTTTTGCGGTGTGGGATGGGCCCGCGGCCTATCAGCTTGTTGGTGGGG
TAATGGCCTACCAAGGCGACGACGGGTAGCCGACCTGAGAGGGTGATCGGCCACACTGGGACTGAGACACGGCCCAGACTCCTACGGGAGGCAGCAGTGG
GGAATATTGCACAATGGGCGCAAGCCTGATGCAGCGACGCCGCGTGAGGGATGACGGCCTTCGGGTTGTAAACCTCTTTCACCAGGGACGAAGCGTGAGT
GACGGTACCTGGAGAAGAAGCACCGGCCAACTACGTGCCAGCAGCCGCGGTAATACGTAGGGTGCGAGCGTTGTCCGGAATTTACTGGGCGTAAAGAGCT
CGTAGGCGGTTTGTCGCGTCGTCTGTGAAATTCTGCAACTCAATTGCAGGCGTGCAGGCGATACGGGCAGACTTGAGTACTACAGGGGAGACTGGAATTC
CTGGTGTAGCGGTGAAATGCGCAGATATCAGGAGGAACACCGGTGGCGAAGGCGGGTCTCTGGGTAGTAACTGACGCTGAGGAGCGAAAGCGTGGGTAGC
GAACAGGATTAGATACCCTGGTAGTCCACGCCGTAAACGGTGGGTACTAGGTGTGGGTTCCTTTTCACGGGATCCGTGCCGTAGCTAACGCATTAAGTAC
CCCGCCTGGGGAGTACGGCCGCAAGGCTAAAACTCAAAGGAATTGACGGGGGCCCGCACAAGCGGCGGAGCATGTGGATTAATTCGATGCAACGCGAAGA
ACCTTACCTGGGTTTGACATACACCAGACGCGGCTAGAGATAGTCGTTCCCTTGTGGTTGGTGTACAGGTGGTGCATGGCTGTCGTCAGCTCGTGTCGTG
AGATGTTGGGTTAAGTCCCGCAACGAGCGCAACCCTTGTCCTGTATTGCCAGCGGGTTATGCCGGGGACTTGCAGGAGACTGCCGGGGTCAACTCGGAGG
AAGGTGGGGATGACGTCAAGTCATCATGCCCCTTATGTCCAGGGCTTCACACATGCTACAATGGCTGGTACAGAGGGCTGCGATACCGTGAGGTGGAGCG
AATCCCTTAAAGCCAGTCTCAGTTCGGATTGGGGTCTGCAACTCGACCCCATGAAGTCGGAGTCGCTAGTAATCGCAGATCAGCAACGCTGCGGTGAATA
CGTTCCCGGGCCTTGTACACACCGCCCGTCACGTCATGAAAGTCGGTAACACCCGAAGCCGGTGGCCTAACCCC

>EB163
TGCAGTCGAACGGGCACTTCGGTGCTAGTGGCAGACGGGTGAGTAACACGTGGGAACGTACCTCTTTGTTCGGAATAATTCAGGGAAACTTGGACTAATA
CCGGATACGCCCTTCGGGGGAAAGATTTATCGCAGAGAGATCGGCCCGCGTCTGATTAGCTAGTTGGTGAGGTAATGGCTCACCAAGGCGACGATCAGTA
GCTGGTCTGAGAGGATGATCAGCCACACTGGGACTGAGACACGGCCCAGACTCCTACGGGAGGCAGCAGTGGGGAATATTGGACAATGGGCGCAAGCCTG
ATCCAGCCATGCCGCGTGAGTGATGAAGGCCTTAGGGTTGTAAAGCTCTTTTGTCCGGGAAGATAATGACTGTACCGGAAGAATAAGCCCCGGCTAACTT
CGTGCCAGCAGCCGCGGTAATACGAAGGGGGCTAGCGTTGCTCGGAATCACTGGGCGTAAAGGGCGCGTAGGCGGACTTTTAAGTCGGAGGTGAAAGCCC
AGGGCTCAACCCTGGAATTGCCTTCGATACTGGAAGTCTTGAGTTCGGAAGAGGTTGGTGGAACTGCGAGTGTAGAGGTGAAATTCGTAGATATTCGCAA
GAACACCAGTGGCGAAGGCGGCCAACTGGTCCGATACTGACGCTGAGGCGCGAAAGCGTGGGGAGCAAACAGGATTAGATACCCTGGTAGTCCACGCCGT
AAACGATGAATGCCAGCCGTTGGGGTGCATGCACTTCAGTGGCGCAGCTAACGCTTTAAGCATTCCGCCTGGGGAGTACGGTCGCAAGATTAAAACTCAA
AGGAATTGACGGGGGCCCGCACAAGCGGTGGAGCATGTGGTTTAATTCGAAGCAACGCGCAGAACCTTACCAGCCTTTGACATGTCCGGTTTGATCGACA
GAGATGTCTTTCTTCAGTTCGGCTGGCCGGAACACAGGTGCTGCATGGCTGTCGTCAGCTCGTGTCGTGAGATGTTGGGTTAAGTCCCGCAACGAGCGCA
ACCCTCGCCCCCTAGTTGCCATCATTCAGTTGGGAACTCTAGGGGGACTGCCGGTGATAAGCCGCGAGGAAGGTGGGGATGACGTCAAGTCCTCATGGCC
CTTACAGGCTGGGCTACACACGTGCTACAATGGCGGTGACAATGGGCAGCGAAAGGGTGACCTGGAGCTAATCCCAAAAAGCCGTCTCAGTTCGGATTGC
ACTCTGCAACTCGAGTGCATGAAGGTGGAATCGCTAGTAATCGTGGATCAGCATGCCACGGTGAATACGTTCCCGGGCCTTGTACACACCGCCCGTCACA
CCATGGGAGTTGGGTTTACCCGAAGGCGTCGCGCTAACCGCAAGGAGGCA

>EB164
TGCAGTCGAACGGTGAAGTCCCAGCTTGCTGGGATGGATCAGTGGCGAACGGGTGAGTAACACGTGAGCAACCTGCCCTCCACTTCGGGATAAGCCTTGG
AAACGGGGTCTAATACCGGATATGAGCCTGCATCGCATGGTGTGGGTTGGAAAGTTTTTCGGTGGGGGATGGGCTCGCGGCCTATCAGCTTGTTGGTGGG
GTGATGGCCTACCAAGGCGTCGACGGGTAGCCGGCCTGAGAGGGCGACCGGCCACACTGGGACTGAGACACGGCCCAGACTCCTACGGGAGGCAGCAGTG
GGGAATATTGCACAATGGGCGCAAGCCTGATGCAGCGACGCCGCGTGAGGGATGACGGCCTTCGGGTTGTAAACCTCTTTCAGCAGGGAAGAAGCGCAAG
TGACGGTACCTGCAGAAGAAGCGCCGGCTAACTACGTGCCAGCAGCCGCGGTAATACGTAGGGCGCAAGCGTTGTCCGGAATTATTGGGCGTAAAGAGCT
CGTAGGCGGTCTGTCGCGTCTGGTGTGAAATCCCGAGGCTCAACCTCGGGCTTGCATCGGGTACGGGCAGACTAGAGTGCGGTAGGGGAGACTGGAATTC
CTGGTGTAGCGGTGGAATGCGCAGATATCAGGAGGAACACCGATGGCGAAGGCAGGTCTCTGGGCCGCAACTGACGCTGAGGAGCGAAAGCATGGGGAGC
GAACAGGATTAGATACCCTGGTAGTCCATGCCGTAAACGTTGGGCACTAGGTGTGGGGCTCATTCCACGAGTTCCGTGCCGCAGCTAACGCATTAAGTGC
CCCGCCTGGGGAGTACGGCCGCAAGGCTAAAACTCAAAGGAATTGACGGGGGCCCGCACAAGCGGCGGAGCATGCGGATTAATTCGATGCAACGCGAAGA
ACCTTACCAAGGCTTGACATGCACCGGAAACACTCAGAGATGGGGTGCCCCGTAAGGTCGGTGCACAGGTGGTGCATGGTTGTCGTCAGCTCGTGTCGTG
AGATGTTGGGTTAAGTCCCGCAACGAGCGCAACCCTCGTCCTATGTTGCCAGCGGGTTATGCCGGGGACTCATGGGAGACTGCCGGGGTCAACTCGGAGG
AAGGTGGGGATGACGTCAAATCATCATGCCCCTTATGTCTTGGGCTTCACGCATGCTACAATGGCCGGTACAGAGGGCTGCGATACCGTAAGGTGGAGCG
AATCCCAAAAAGCCGGTCTCAGTTCGGATTGGGGTCTGCAACTCGACCCCATGAAGTCGGAGTCGCTAGTAATCGCAGATCAGCAACGCTGCGGTGAATA
CGTTCCCGGGCCTTGTACACACCGCCCGTCAAGTCACGAAAGTTGGTAACACCCGAAGCTCATGGCCCAACCGGTTTTCCGGGGGG

>EB165
GCCTCTATCGCATGGTGGGGGTTGGAAAGATTTTTCGGTCTGGGATGGGCTCGCGGCCTATCAGCTTGTTGGTGAGGTAATGGCTCACCAAGGCGTCGAC
GGGTAGCCGGCCTGAGAGGGTGACCGGCCACACTGGGACTGAGACACGGCCCAGACTCCTACGGGAGGCAGCAGTGGGGAATATTGCACAATGGGCGGAA
GCCTGATGCAGCAACGCCGCGTGAGGGATGACGGCCTTCGGGTTGTAAACCTCTTTTAGCAGGGAAGAAGCGAGAGTGACGGTACCTGCAGAAAAAGCGC
CGGCTAACTACGTGCCAGCAGCCGCGGTAATACGTAGGGCGCAAGCGTTATCCGGAATTATTGGGCGTAAAGAGCTCGTAGGCGGTTTGTCGCGTCTGCT
GTGAAATCCCGAGGCTCAACCTCGGGCCTGCAGTGGGTACGGGCAGACTAGAGTGCGGTAGGGGAGATTGGAATTCCTGGTGTAGCGGTGGAATGCGCAG
ATATCAGGAGGAACACCGATGGCGAAGGCAGATCTCTGGGCCGTAACTGACGCTGAGGAGCGAAAGGGTGGGGAGCAAACAGGCTTAGATACCCTGGTAG
TCCACCCCGTAAACGTTGGGAACTAGTTGTGGGGACCATTCCACGGTTTCCGTGACGCAGCTAACGCATTAAGTTCCCCGCCTGGGGAGTACGGCCGCAA
GGCTAAAACTCAAAGGAATTGACGGGGACCCGCACAAGCGGCGGAGCATGCGGATTAATTCGATGCAACGCGAAGAACCTTACCAAGGCTTGACATACAC
CAGAACACCGTAGAAATACGGGACTCTTTGGACACTGGTGAACAGGTGGTGCATGGTTGTCGTCAGCTCGTGTCGTGAGATGTTGGGTTAAGTCCCGCAA
CGAGCGCAACCCTCGTTCTATGTTGCCAGCACGTAATGGTGGGAACTCATGGGATACTGCCGGGGTCAACTCGGAGGAAGGTGGGGATGACGTCAAATCA
TCATGCCCCTTATGTCTTGGGCTTCACGCATGCTACAATGGCCGGTACAAAGGGCTGCAATACCGTGAGGTGGAGCGAATCCCAAAAAGCCGGTCCCAGT
TCGGATTGAGGTCTGCAACTCGACCTCATGAAGTAGGAGTCGCTTGTAATCGCAGATCAGCAACGTTGCGGTGAATACGTTCCCGGGTCTTGTACACTCC
GCCCCTCAAGTCCGGAACATCGTTACCA

>EB166
AGTTTGATTATGGATCAGGTAGATCGTACGTGGGCAACCTGCCTGTTGACTGGGATAACTTCGGGAAACCGAAGCTAATACCGGATAGGATCTTCTCCTT
CATGGGAGATGATTGAAAGATGGTTTCGGCTATCACTTACAGATGGGCCCGCGGTGCATTAGCTAGTTGGTGAGGTAACGGCTCACCAAGGCAACGATGC
ATAGCCGACCTGAGAGGGTGATCGGCCACACTGGGACTGAGACACGGCCCAGACTCCTACGGGAGGCAGCAGTAGGGAATCTTCCGCAATGGACGAAAGT
CTGACGGAGCAACGCCGCGTGAGTGATGAAGGCTTTCGGGTCGTAAAACTCTGTTGTTAGGGAAGAACAAGTACGAGAGTAACTGCTTGTACCTTGACGG
TACCTAACCAGAAAGCCACGGCTAAYTACGTGCCAGCAGCCGCGGTAATACGTAGGTGGCAAGCGTTATCCGGAATTATTGGGCGTAAAGCGCGCGCAGG

CGGTTTCTTAAGTCTGATGTGAAAGCCCACGGCTCAACCGTGGAGGGTCATTGGAAACTGGGGAACTTGAGTGCAGAAGAGAAAAGCGGAATTCCACGTG
TAGCGGTGAAATGCGTAGAGATGTGGAGGAACACCAGTGGCGAAGGCGGCTTTTTGGTCTGTAACTGACGCTGAGGCGCGAAAGCGTGGGGAGCAAACAG
GATTAGATACCCTGGTAGTCCACGCCGTAAACGATGAGTGCTAAGTGTTAGAGGGTTTCCGCCCTTTAGTGCTGCAGCTAACGCATTAAGCACTCCGCCT
GGGGAGTACGGTCGCAAGACTGAAACTCAAAGGAATTGACGGGGGCCCGCACAAGCGGTGGAGCATGTGGTTTAATTCGAAGCAACGCGAAGAACCTTAC
CAGGTCTTGACATCCTCTGACAACTCTAGAGATAGAGCGTTCCCCTTCGGGGGACAGAGTGACAGGTGGTGCATGGTTGTCGTCAGCTCGTGTCGTGAGA
TGTTGGGTTAAGTCCCGCAACGAGCGCAACCCTTGATCTTAGTTGCCAGCATTTAGTTGGGCACTCTAAGGTGACTGCCGGTGACAAACCGGAGGAAGGT
GGGGATGACGTCAAATCATCATGCCCCTTATGACCTGGGCTACACACGTGCTACAATGGATGGTACAAAGGGCTGCAAGACCGCGAGGTCAAGCCAATCC
CATAAAACCATTCTCAGTTCGGATTGTAGGCTGCAACTCGCCTACATGAAGCTGGAATCGCTAGTAATCGCGGATCAGCATGCCGCGGTGAATACGTTCC
CGGGCCTTGTACACACCGCCCGTCACACC

>EB167
TGCAGTCGAACGGTAGCACAGAGAGCTTGCTCTCGGGTGACGAGTGGCGGACGGGTGAGTAATGTCTGGGAAACTGCCCGATGGAGGGGGATAACTACTG
GAAACGGTAGCTAATACCGCATAACGTCGCAAGACCAAAGTGGGGGACCTTCGGGCCTCACACCATCGGATGTGCCCAGATGGGATTAGCTAGTAGGTGG
GGTAATGGCTCACCTAGGCGACGATCCCTAGCTGGTCTGAGAGGATGACCAGCCACACTGGAACTGAGACACGGTCCAGACTCCTACGGGAGGCAGCAGT
GGGGAATATTGCACAATGGGCGCAAGCCTGATGCAGCCATGCCGCGTGTATGAAGAAGGCCTTCGGGTTGTAAAGTACTTTCAGCGGGGAGGAAGGCGGT
GGAAGTTAATAACTTCACCGATTGACGTTACCCGCAGAAGAAGCACCGGCTAACTCCGTGCCAGCAGCCGCGGTAATACGGAGGGTGCAAGCGTTAATCG
GAATTACTGGGCGTAAAGCGCACGCAGGCGGTCTGTCAAGTCGGATGTGAAATCCCCGGGCTTAACCTGGGAACTGCATTCGAAACTGGCAGGCTAGAGT
CTTGTAGAGGGGGGTAGAATTCCAGGTGTAGCGGTGAAATGCGTAGAGATCTGGAGGAATACCGGTGGCGAAGGCGGCCCCCTGGACAAAGACTGACGCT
CAGGTGCGAAAGCGTGGGGAGCAAACAGGATTAGATACCCTGGTAGTCCACGCCGTAAACGATGTCGACTTGGAGGTTGTTCCCTTGAGGAGTGGCTTCC
GGAGCTAACGCGTTAAGTCGACCGCCTGGGGAGTACGGCCGCAAGGTTAAAACTCAAATGAATTGACGGGGGCCCGCACAAGCGGTGGAGCATGTGGTTT
AATTCGATGCAACGCGAAGAACCTTACCTACTCTTGACATCCAGAGAACTTAGCAGAGATGCTTTGGTGCCTTCGGGAACTCTGAGACAGGTGCTGCATG
GCTGTCGTCAGCTCGTGTTGTGAAATGTTGGGTTAAGTCCCGCAACGAGCGCAACCCTTATCCTTTGTTGCCAGCGGTTCGGCCGGGAACTCAAAGGAGA
CTGCCAGTGATAAACTGGAGGAAGGTGGGGATGACGTCAAGTCATCATGGCCCTTACGAGTAGGGCTACACACGTGCTACAATGGCGCATACAAAGAGAA
GCGACCTCGCGAGAGCAAGCGGACCTCATAAAGTGCGTCGTAGTCCGGATCGGAGTCTGCAACTCGACTCCGTGAAGTCGGAATCGCTAGTAATCGTAGA
TCAGAATGCTACGGTGAATACGTTCCCGGGCCTTGTACACACCGCCCGTCACACCATGGGAGTGGGTTGCAAAAGAAGTAGGTAGCTTAACCTTCGGGAG
GGCGC

>EB168
TGCAGTCGAACGGTGACCCTCGGAGCTTGCTTCGGGGTGAACAGTGGCGAACGGGTGAGTAACACGTGAGTAACCTGCCCCTGACTCTGGAATAAGCGCT
GGAAACGGCGTCTAATACCGGATACGACACGCCTCCGCATGGGGAGCGTGTGGAAAGTTTTTCGGTCTGGGATGGACTCGCGGCCTATCAGCTTGTTGGT
GAGGTAATGGCTCACCAAGGCGATGACGGGTAGCCGGCCTGAGAGGGCGACCGGCCACACTGGGACTGAGACACGGCCCAGACTCCTACGGGAGGCAGCA
GTGGGGAATATTGCACAATGGGCGAAAGCCTGATGCAGCGACGCCGCGTGAGGGATGACGGCCTTCGGGTTGTAAACCTCTTTCAGCAGGGAAGAAGCGA
AAGTGACGGTACCTGCAGAAGAAGCACCGGCTAACTACGTGCCAGCAGCCGCGGTAATACGTAGGGTGCGAGCGTTGTCCGGAATTATTGGGCGTAAAGA
GCTTGTAGGCGGTTTGTCGCGTCTGCCGTGAAAATCCGGGGCTCAACCCCGGACTTGCGGTGGGTACGGGCAGACTAGAGTGTGGTAGGGGAGACTGGAA
TTCCTGGTGTAGCGGTGAAATGCGCAGATATCAGGAGGAACACCGATGGCGAAGGCAGGTCTCTGGGCCACTACTGACGCTGAGAAGCGAAAGCATGGGG
AGCGAACAGGATTAGATACCCTGGTAGTCCATGCCGTAAACGTTGGGCGCTAGGTGTGGGACTCATTCCACGAGTTCCGTGCCGCAGCTAACGCATTAAG
CGCCCCGCCTGGGGAGTACGGCCGCAAGGCTAAAACTCAAAGGAATTGACGGGGGCCCGCACAAGCGGCGGAGCATGTGGATTAATTCGATGCAACGCGA
AGAACCTTACCAAGGCTTGACATATACCGGAAACATCTGGAGACAGGTGCCCCGCAAGGTCGGTATACAGGTGGTGCATGGTTGTCGTCAGCTCGTGTCG
TGAGATGTTGGGTTAAGTCCCGCAACGAGCGCAACCCTCGTTCTATGTTGCCAGCACGTAATGGTGGGGACTCATAGGAGACTGCCGGGGTCAACTCGGA
GGAAGGTGGGGATGAGGTCAAATCATCATGCCCCTTATGTCTTGGGCTTCACACATGCTACAATGGCCGGTACAAAGGGCTGCGAAACCGTGAGGTGGAG
CGAATCCCAAAAAACCGGTCTCAGTTCGGATTGGGGTCTGCAACTCGACCCCATGAAGTTGGAGTCGCTAGTAATCGCAGATCAGCAACGCTGCGGTGAA
TACGTTCCCGGGCCTTGTACACACCGCCCGTCAAGTCACGAAAGTCGGTAACACCCGAAGCCGGTGGCCCAACCCTTGTGGAGGGAGCCGTCG

>EB169
TGCCCTTAACTCTGGGATAAGCCTGGGAAACTGGGTCTAATACCGGATAGGAGCGTCCACCGCATGGTGGGTGTTGGAAAGATTTATCGGTTTTGGATGG
ACTCGCGGCCTATCAGCTTGTTGGTGAGGTAATGGCTCACCAAGGCGACGACGGGTAGCCGGCCTGAGAGGGTGACCGGCCACACTGGGACTGAGACACG
GCCCAGACTCCTACGGGAGGCAGCAGTGGGGAATATTGCACAATGGGCGCAAGCCTGATGCAGCGACGCCGCGTGAGGGATGACGGCCTTCGGGTTGTAA
ACCTCTTTCAGTAGGGAAGAAGCGAAAGTGACGGTACCTGCAGAAGAAGCACCGGCTAACTACGTGCCAGCAGCCGCGGTAATACGTAGGGTGCGAGCGT
TATCCGGAATTATTGGGCGTAAAGAGCTCGTAGGCGGTTTGTCGCGTCTGTCGTGAAAGTCCGGGGCTTAACCCCGGATCTGCGGTGGGTACGGGCAGAC
TAGAGTGCAGTAGGGGAGACTGGAATTCCTGGTGTAGCGGTGGAATGCGCAGATATCAGGAGGAACACCGATGGCGAAGGCAGGTCTCTGGGCTGTAACT
GACGCTGAGGAGCGAAAGCATGGGGAGCGAACAGGATTAGATACCCTGGTAGTCCATGCCGTAAACGTTGGGCACTAGGTGTGGGGACCATTCCACGGTT
TCCGCGCCGCAGCTAACGCATTAAGTGCCCCGCCTGGGGAGTACGGCCGCAAGGCTAAAACTCAAAGGAATTGACGGGGGCCCGCACAAGCGGCGGAGCA
TGCGGATTAATTCGATGCAACGCGAAGAACCTTACCAAGGCTTGACATGTTCTCGATCGCCGTAGAGATACGGTTTCCCCTTTGGGGCGGGTTCACAGGT
GGTGCATGGTTGTCGTCAGCTCGTGTCGTGAGATGTTGGGTTAAGTCCCGCAACGAGCGCAACCCTCGTTCCATGTTGCCAGCACGTCGTGGTGGGGACT
CATGGGAGACTGCCGGGGTCAACTCGGAGGAAGGTGAGGACGACGTCAAATCATCATGCCCCTTATGTCTTGGGCTTCACGCATGCTACAATGGCCGGTA
CAATGGGTTGCGATACTGTGAGGTGGAGCTAATCCCAAAAAGCCGGTNTCAGTTCGGATTGGGGTCTGCAACTCGACCCCATGAAGTCGGAGTCGCTAGT
AATCGCAGATCAGCAACGCTGCGGTGAATACGTTCCCGGGCCTTGTACACACCGCCCGTC

>EB170
GGCTCCCTCCCACAAGGGGTTAGGCCACCGGCTTCGGGTGTTACCAACTTTCGTGACTTGACGGGCGGTGTGTACAAGGCCCGGGAACGTATTCACCGCA
GCGTTGCTGATCTGCGATTACTAGCGACTCCGACTTCATGAGGTCGAGTTGCAGACCTCAATCCGAACTGAGACCGGCTTTTTGGGATTAGCTCCACCTC
ACAGTATCGCAACCCTTTGTACCGGCCATTGTAGCATGCGTGAAGCCCAAGACATAAGGGGCATGATGATTTGACGTCATCCCCACCTTCCTCCGAGTTG
ACCCCGGCAGTCTCCTATGAGTCCCCACCATCACGTGCTGGCAACATAGAACGAGGGTTGCGCTCGTTGCGGGACTTAACCCAACATCTCACGACACGAG
CTGACGACAACCATGCACCACCTGTCCACCAGCCCCGAAGGGAAACCCCATCTCTGGGGCGGTCCGGTGAATGTCAAGCCTTGGTAAGGTTCTTCGCGTT
GCATCGAATTAATCCGCATGCTCCGCCGCTTGTGCGGGCCCCCGTCAATTCCTTTGAGTTTTAGCCTTGCGGCCGTACTCCCCAGGCGGGGCACTTAATG
CGTTAGCTACGGCGCGGAAAACGTGGAATGTCCCCCACACCTAGTGCCCAACGTTTACGGCATGGACTACCAGGGTATCTAATCCTGTTCGCTCCCCATG
CTTTCGCTCCTCAGCGTCAGTAACAGCCCAGAGACCTGCCTTCGCCATCGGTGTTCCTCCTGATATCTGCGCATTTCACCGCTACACCAGGAATTCCAGT
CTCCCCTACTGCACTCTAGTCTGCCCGTACCCACTGCAGACCCGGGGTTGAGCCCCGGGCTTTCACAGCAGACGCGACAAACCGCCTACGAGCTCTTTAC
GCCCAATAATTCCGGACAACGCTTGCGCCCTACGTATTACCGCGGCTGCTGGCACGTAGTTAGCCGGCGCTTCTTCTGCAGGTACCGTCACTCTCGCTTC
TTCCCTACTGAAAGAGGTTTACAACCCGAAGGCCGTCATCCCTCACGCGGCGTCGCTGCATCAGGCTTCCGCCCATTGTGCAATATTCCCCACTGCTGCC
TCCCGTAGGAGTCTGGGCCGTGTCTCAGTCCCAGTGTGGCCGGTCACCCTCTCAGGCCGGCTACCCGTCGTCGCCTTGGTGAGCCATTACCCCACCAACA
AGCTGATAGGCCGTGAGCCCATCCAAAACCAGTAAAACCCTTTCCACCACCCACCATGCGGCAAGAGGTAGTATCCAGTATTAGACCCAGTTTCCCAGGC
TTATCCCAGAGTCAAGGGCAGGTTACTCACGTATTACTCACCCGTTCGCCACTAATCCGCCCAGCAAGCTGGGCATCATCGTTCGACTGCA

>EB171
GGGTTAGGCCACCGGCTTCGGGTGTTACCAACTTTCGTGACTTGACGGGCGGTGTGTACAAGGCCCGGGAACGTATTCACCGCAGCGTTGCTGATCTGCG
ATTACTAGCGACTCCGACTTCACGTGGTCGAGTTGCAGACCACGATCCGAACTGAGACCAGCTTTTTGGGATTAGCTCCACCTCACGGTATCGCAACCCA
TTGTACCGGCCATTGTAGCATGCGTGAAGCCCAAGACATAAGGGGCATGATGATTTGACGTCATCCCCACCTTCCTCCGAGTTGACCCCGGCAGTCTCCT

ATGAGTCCCCACCATCACGTGCTGGCAACATAGAACGAGGGTTGCGCTCGTTGCGGGACTTAACCCAACATCTCACGACACGAGCTGACGACAACCATGC
ACCACCTGTACACCAACCTCAAAGAGGAAAAACCCATCTCTGGGCCGGTCTGGTGTATGTCAAGCCTTGGTAAGGTTCTTCGCGTTGCATCGAATTAATC
CGCATGCTCCGCCGCTTGTGCGGGCCCCCCGTCAATTCCTTTGAGTTTTAGCCTTGCGGCCGTACTCCCCAGGCGGGGCACTTAATGCGTTAGCTACGGC
GCGGAAAACGTGGAATGTCCCCCACACCTAGTGCCCAACGTTTACGGCATGGACTACCAGGGTATCTAATCCTGTTCGCTCCCCATGCTTTCGCTCCTCA
GCGTCAGTAACAGCCCAGAGACCTGCCTTCGCCATCGGTGTTCCTCCTGATATCTGCGCATTTCACCGCTACACCAGGAATTCCAGTCTCCCCTACTGCA
CTCTAGTCTGCCCGTACCCACTGCACACCACGGGGTTAAGCCCCGGGCTTTCACAGCAGACGCGACAAACCGCCTACGAGCTCTTTACGCCCAATAATTC
CGGACAACGCTTGCGCCCTACGTATTACCGCGGCTGCTGGGCACGTAGTTAGCCGGCGCTTCTTCTGCACGTACCGTCACTTTCGCTTCTTCCGTGCTGA
AAGAGGTTTACAACCCGAAGGCCGTCATCCCTCACGCGGCGTCGCTGCATCAGGCTTTCGCCCATTGTGCAATATTCCCCACTGCTGCCTCCCGTAGGAG
TCTGGGCCGTGTCTCAGTCCCAGTGTGGCCGGTCACCCTCTCAGGCCGGCTACCCGTCGTCGCCTTGGTAGGCCATTACCCCACCAACAAGCTGATAGGC
CGTGAGCCCATCCAAAACCAGTAAAACCCTTTCCACACACCCCCATGCGAGAGAATGTAGTATCCAGTATTAGACCCAGTTTCCCAGGCTTATCCCAGAG
TCAAGGGCAGGTTACTCACGTATTACTCACCCGTTCGCCACTCATCCACCCAGATGCAAGCACCCAAGCTTCAGCGTTCGAC

>EB172
GTAAAGGATGAGTGGGGAACGGGTGAGTAATACGTGAGTAACCTGCCCTTGACTCTGGGATAAGCCTGGGAAACTGGGTCTAATACTGGATACGACATGT
CACCGCATGGTGGTGTGTGGAAAGGGTTTTACTGGTTTTGGATGGGCTCACGGCCTATCAGCTTGTTGGTGGGGTAATGGCTCACCAAGGCGACGACGGG
TAGCCGGCCTGAGAGGGTGACCGGCCACACTGGGACTGAGACACGGCCCAGACTCCTACGGGAGGCAGCAGTGGGGAATATTGCACAATGGGCGGAAGCC
TGATGCAGCGACGCCGCGTGAGGGATGACGGCCTTCGGGTTGTAAACCTCTTTCAGCACGGAAGAAGCGAAAGTGACGGTACGTGCAGAAGAAGCGCCGG
CTAACTACGTGCCAGCAGCCGCGGTAATACGTAGGGCGCAAGCGTTGTCCGGAATTATTGSGCGTAAAGAGCTCGTAGGCGGTTTGTCGCGTCTGCTGTG
AAAGCCCGGGGCTTAACCCCGGGTGTGCAGTGGGTACGGGCAGACTTGAGTGCAGTAGGGGAGACTGGAATTCCTGGTGTAGCGGTGAAATGCGCAGATA
TCAGGAGGAACACCGATGGCGAAGGCAGGTCTCTGGGCTGTTACTGACGCTGAGGAGCGAAAGCATGGGGAGCGAACAGGATTAGATACCCTGGTAGTCC
ATGCCGTAAACGTTGGGCACTAGGTGTGGGGAACATTCCACGTTTTCCGCGCCGTAGCTAACGCATTAAGTGCCCCGCCTGGGGAGTACGGCCGCAAGGC
TAAAACTCAAAGGAATTGACGGGGGCCCGCACAAGCGGCGGAGCATGCGGATTAATTCGATGCAACGCGAAGAACCTTACCAAGGCTTGACATACACCGG
ACCGGGCCAGAGATGGTCTTTCCCCCTTGTGGGGCTGGTGTACAGGTGGTGCATGGTTGTCGTCAGCTCGTGTCGTGAGATGTTGGGTTAAGTCCCGCAA
CGAGCGCAACCCTCGTTCTATGTTGCCAGCACGTGATGGTGGGGACTCATAGGAGACTGCCGGGGTCAACTCGGAGGAAGGTGGGGATGACGTCAAATCA
TCATGCCCCTTATGTCTTGGGCTTCACGCATGCTACAATGGCCAGTACAATGGGTTGCGATGCCGCGAGGTGGAGCTAATCCCAAAAAGCTGGTCTCAGT
TCGGATCGTGGTCTGCAACTCGACCACGTGAAGTCGGAGTCGCTTGTAATCGCAGATCAGCAACGCTGCGGTGAATACGTTCCCGGTCCTTGTACACACC
GCCCCTCAAGTCACGAAAGTTGGTAAC

>EB173
GGCTCCCTCCACAAGGGTTGGGCCACCGGCTTCGGGTGTTACCGACTTTCGTGACTTGACGGGCGGTGTGTACAAGGCCCGGGAACGTATTCACCGCAGC
GTTGCTGATCTGCGATTACTAGCGACTCCGACTTCATGAGGTCGAGTTGCAGACCTCAATCCGAACTGAGACCGGCTTTTTGGGATTAGCTCCACCTCAC
AGTATCGCAACCCTTTGTACCGGCCATTGTAGCATGCGTGAAGCCCAAGACATAAGGGGCATGATGATTTGACGTCATCCCCACCTTCCTCCGAGTTGAC
CCCGGCAGTCTCCCATGAGTCCCCACCATTACGTGCTGGCAACATGGAACGAGGGTTGCGCTCGTTGCGGGACTTAACCCAACATCTCACGACACGAGCT
GACGACAACCATGCACCACCTGTATACCGACCCCAAAGGGGAAGAACCATCTCTGGAACGATCCGGTATATGTCAAGCCTTGGTAAGGTTCTTCGCGTTG
CATCGAATTAATCCGCATGCTCCGCCGCTTGTGCGGGCCCCCGTCAATTCCTTTGAGTTTTAGCCTTGCGGCCGTACTCCCCAGGCGGGGCACTTAATGC
GTTAGCTACGGCGCGGAAAACGTGGAATGTCCCCCACACCTAGTGCCCAACGTTTACGGCATGGACTACCAGGGTATCTAATCCTGTTCGCTCCCCATGC
TTTCGCTCCTCAGCGTCAGTAACAGCCCAGAGACCTGCCTTCGCCATCGGTGTTCCTCCTGATATCTGCGCATTCCACCGCTACACCAGGAATTCCAGTC
TCCCCTACTGCACTCTAGTCTGCCCGTACCCACTGCACACCCGGGGTTAAGCCCCGGGCTTTCACAGCAGACGCGACAAACCGCCTACGAGCTCTTTACG
CCCAATAATTCCGGACAACGCTTGCGCCCTACGTATTACCGCGGCTGCTGGCACGTAGTTAGCCGGCGCTTCTTCTGCAGGTACCGTCACTTGTGGCTTC
TTCCCTGCTGAAAGAGGTTTACAACCCGAAGGCCGTCATCCCTCACGCGGCGTCGCTGCATCAGGCTTTCGCCCATTGTGCAATATTCCCCACTGCTGCC
TCCCGTAGGAGTCTGGGCCGTGTCTCAGTCCCAGTGTGGCCGGTCACCCTCTCAGGCCGGCTACCCGTCGTCGCCTTGGTGAGCCATTACCTCACCAACA
AGCTGATAGGCCGTGAGCCCATCCAAGACCAGTAAACCCTTTCCACACACCACCATGCGGTGACGTGTAGCATCCAGTATTAGACCCAGTTTCCCGGGCT
TATCCCAGAGTCAAGGGCAGGTTACTCACGTATTACTCACCCGTTCGCCACTCATCCACACCAGCAAGCTGGTGCTTCAGCGTTCGA

>EB174
TGTTACGACTTACTGAACCCCAACCAAACTCTACGGTTACCTTGCTCGAGGTGTGGAGGGAGGAGTGTACTCTACCCGGAAACGGATTCACCGCGGCATG
CTGATCCGCGATTACAAGCGATTCCAGCTTCATGTAGGCGAGTTGCAGCCTACAATCCGAACTGAGAATGGTTTTATGGGATTGGCTCGACCTCGCGGTT
TTGCTGCCCTTTGTACCATCCATTGTAGCACGTGTGTAGCCCAGGTCATAAGGGGCATGATGATTTGACGTCATCCCCACCTTCCTCCGGTTTGTCACCG
GCAGTCACCTTAGAGTGCCCAACTAAATGCTGGCAACTAAGATCAAGGGTTGCGCTCGTTGCGGGACTTAACCCAACATCTCACGACACGAGCTGACGAC
AACCATGCACCACCTGTCATCCTGTCCCCCGAAGGGGAACGTCCTATCTCTAGGATTGTCAGGAGATGTCAAGACCTGGTAAGGTTCTTCGCGTTGCTTC
GAATTAAACCACATGCTCCACCGCTTGTGCGGGCCCCCGTCAATTCCTTTGAGTTTCAGCCTTGCGGCCGTACTCCCCAGGCGGAGTGCTTAATGCGTTT
GCTGCAGCACTAAAGGGCGGAAACCCTCTAACACTTAGCACTCATCGTTTACGGCGTGGACTACCAGGGTATCTAATCCTGTTTGCTCCCCACGCTTTCG
CGCCTCAGCGTCAGTTACAGACCAAAGAGTCGCCTTCGCCACTGGTGTTCCTCCACATCTCTACGCATTTCACCGCTACACGTGGAATTCCACTCTTCTC
TTCTGCACTCAAGTCCCCCAGTTTCCAATGACCCTCCACGGTTGAGCCGTGGGCTTTCACATCAGACTTAAAGGACCGCCTGCGCGCGCTTTACGCCCAA
TAATTCCGGACAACGCTTGCCACCTACGTATTACCGCGGCTGCTGGCACGTAGTTAGCCGTGGCTTTCTGGTTAGGTACCGTCAAGGTACGAGCAGTTAC
TCTCGTACTTGTTCTTCCCTAACAACAGAGTTTTACGATCCGAAAACCTTCATCACTCACGCGGCGTTGCTCCGTCAGACTTTCGTCCATTGCGGAAGAT
TCCCTACTGCTGCCTCCCGTAGGAGTCTGGGCCGTGTCTCAGTCCCAGTGTGGCCGATCACCCTCTCAGGTCGGCTACGCATCGTCGCCTTGGTGAGCCG
TTACCTCACCAACTAGCTAATGCGCCGCGGGCCCATCTGTAAGTGACAGCGTAAACCGTCTTTCAGCTTTTCTACATGAGTAGAAAAGGATTATCCGGTA
TTAGCTCCGGTTTCCCGAAGTTATCCCAGTCTTACAGGCAGGTTGCCCACGAGTTACTCAGCC

>EB175
GTTACAAACTCTCGTGGTGTGACGGGCGGTGTGTACAAGGACCGGGAACGTATTCACCGCGGCATGCTGATCCGCGATTACTAGCGATTCCGGCTTCATG
CAGGCGAGTTGCAGCCTGCAATCCGAACTGAGAATGGCTTTATGGGATTCGCTTACCTTCGCAGGTTTGCAGCCCTTTGTACCATCCATTGTAGCACGTG
TGTAGCCCAGGTCATAAGGGGCATGATGATTTGACGTCATCCCCACCTTCCTCCGGTTTGTCACCGGCAGTCACCTTAGAGTGCCCAACTGAATGCTGGC
AACTAAGATCAAGGGTTGCGCTCGTTGCGGGACTTAACCCAACATCTCACGACACGAGCTGACGACAACCATGCACCACCTGTCACTCTGTCCCCCGAAG
GGGAAAGCCCTATCTCTAGGGTTGTCAGAGGATGTCAAGACCTGGTAAGGTTCTTCGCGTTGCTTCGAATTAAACCACATGCTCCACCGCTTGTGCGGGC
CCCCGTCAATTCCTTTGAGTTTCAGCCTTGCGGCCGTACTCCCCAGGCGGAGTGCTTAATGCGTTAGCTGCAGCACTAAAGGGCGGAAACCCTCTAACAC
TTAGCACTCATCGTTTACGGCGTGGACTACCAGGGTATCTAATCCTGTTTGCTCCCCACGCTTTCGCGCCTCAGTGTCAGTTACAGACCAGAAAGTCGCC
TTCGCCACTGGTGTTCCTCCAAATCTCTACGCATTTCACCGCTACACTTGGAATTCCACTTTCCTCTTCTGCACTCAAGTCCCCCAGTTTCCAATGACCC
TCCACGGTTGAGCCGTGGGCTTTCACATCAGACTTAAGGAACCACCTGCGCGCGCTTTACGCCCAATAATTCCGGACAACGCTTGCCACCTACGTATTAC
CGCGGCTGCTGGCACGTAGTTAGCCGTGGCTTTCTGGTTAGGTACCGTCAAGGTACCAGCAGTTACTCTGGTACTTGTTCTTCCCTAACAACAGAACTTT
ACGACCCGAAGGCCTTCTTCGTTCACGCGGCGTTGCTCCGTCAGACTTTCGTCCATTGCGGAAGATTCCCTACTGCTGCCTCCCGTAGGAGTCTGGGCCG
TGTCTCAGTCCCAGTGTGGCCGATCACCCTCTCAGGTCGGCTACGCATCGTCGCCTTGGTGAGCCATTACCTCACCAACTAGCTAATGCGCCGCGGGCCC
ATCTATAAGTGACAGCGTAAACCGTCTTTCCATCTTCTCTCATGCGAGAAAAGAACGTATCCGGTATTAGCTCCGGTTTCCCGAAGTTATCCCAGTCTTA
TAGGCAGGTTGCCCACGTGTTACTCACCCGTCCGCCGCTAATCTCAGGGAGCAAGCT

>EB176

CAAGGGGTTAGGCCACCGGCTTCGGGTGTTACCAACTTTCGTGACTTGACGGGCGGTGTGTACAAGGCCCGGGAACGTATTCACCGCAGCGTTGCTGATC
TGCGATTACTAGCGACTCCGACTTCATGAGGTCGAGTTGCAGACCTCAATCCGAACTGAGACCGGCTTTTTGGGATTAGCTCCACCTCACAGTATCGCAA
CCCTTTGTACCGGCCATTGTAGCATGCGTGAAGCCCAAGACATAAGGGGCATGATGATTTGACGTCATCCCCACCTTCCTCCGAGTTGACCCCGGCAGTC
TCCTATGAGTCCCCACCATCACGTGCTGGCAACATAGAACGAGGGTTGCGCTCGTTGCGGGACTTAACCCAACATCTCACGACACGAGCTGACGACAACC
ATGCACCACCTGTCCACCAGCCCCGAAGGGAAACCCCATCTCTGGGGCGGTCCGGTGAATGTCAAGCCTTGGTAAGGTTCTTCGCGTTGCATCGAATTAA
TCCGCATGCTCCGCCGCTTGTGCGGGCCCCCGTCAATTCCTTTGAGTTTTAGCCTTGCGGCCGTACTCCCCAGGCGGGGCACTTAATGCGTTAGCTACGG
CGCGGAAAACGTGGAATGTCCCCCACACCTAGTGCCCAACGTTTACGGCATGGACTACCAGGGTATCTAATCCTGTTCGCTCCCCATGCTTTCGCTCCTC
AGCGTCAGTAACAGCCCAGAGACCTGCCTTCGCCATCGGTGTTCCTCCTGATATCTGCGCATTTCACCGCTACACCAGGAATTCCAGTCTCCCCTACTGC
ACTCTAGTCTGCCCGTACCCACTGCAGACCCGGGGGTTGAGCCCCGGGCTTTCACAGCAGACGCGACAAACCGCCTACGAGCTCTTTACGCCCAATAATT
CCGGACAACGCTTGCGCCCTACGTATTACCGCGGCTGCTGGCACGTAGTTAGCCGGCGCTTCTTCTGCAGGTACCGTCACTCTCGCTTCTTCCCTACTGA
AAGAGGTTTACAACCCGAAGGCCGTCATCCCTCACGCGGCGTCGCTGCATCAGGCTTCCGCCCATTGTGCAATATTCCCCACTGCTGCCTCCCGTAGGAG
TCTGGGCCGTGTCTCAGTCCCAGTGTGGCCGGTCACCCTCTCAGGCCGGCTACCCGTCGTCGCCTTGGTGAGCCATTACCCCACCAACAAGCTGATAGGC
CGTGAGCCCATCCAAAACCAGTAAAACCCTTTCCACCACCCACCATGCGGTAAGAGGTAGTATCCAGTATTAGACCCAGTTTCCCAGGCTTATCCCAGAG
TCAAGGGCAGGTTACTCACGTATTACTCACCCGTTCGCCACTAATCCGCCCAGCAAGCTGGGCATCATCGTTCGA

>EB177
ATGATGCCCAGCTTGCTGGGCGGATTAGTGGCGAACGGGTGAGTAATACGTGAGTAACCTGCCCTTGACTCTGGGATAAGCCTGGGAAACTGGGTCTAAT
ACTGGATACTACCTCTTACCGCATGGTGGGTGGTGGAAAGGGTTTTACTGGTTTTGGATGGGCTCACGGCCTATCAGCTTGTTGGTGGGGTAATGGCTCA
CCAAGGCGACGACGGGTAGCCGGCCTGAGAGGGTGACCGGCCACACTGGGACTGAGACACGGCCCAGACTCCTACGGGAGGCAGCAGTGGGGAATATTGC
ACAATGGGCGGAAGCCTGATGCAGCGACGCCGCGTGAGGGATGACGGCCTTCGGGTTGTAAACCTCTTTCAGTAGGGAAGAAGCGAGAGTGACGGTACCT
GCAGAAGAAGCGCCGGCTAACTACGTGCCAGCAGCCGCGGTAATACGTAGGGCGCAAGCGTTGTCCGGAATTATTGGGCGTAAAGAGCTCGTAGGCGGTT
TGTCGCGTCTGCTGTGAAAGCCCGGGGCTCAACCCCGGGTCTGCAGTGGGTACGGGCAGACTAGAGTGCAGTAGGGGAGACTGGAATTCCTGGTGTAGCG
GTGAAATGCGCAGATATCAGGAGGAACACCGATGGCGAAGGCAGGTCTCTGGGCTGTTACTGACGCTGAGGAGCGAAAGCATGGGGAGCGAACAGGATTA
GATACCCTGGTAGTCCATGCCGTAAACGTTGGGCACTAGGTGTGGGGGACATTCCACGTTTTCCGCGCCGTAGCTAACGCATTAAGTGCCCCGCCTGGGG
AGTACGGCCGCAAGGCTAAAACTCAAAGGAATTGACGGGGGCCCGCACAAGCGGCGGAGCATGCGGATTAATTCGATGCAACGCGAAGAACCTTACCAAG
GCTTGACATTCACCGGACCGCCCCAGAGATGGGGTTTCCCTTCGGGGCTGGTGGACAGGTGGTGCATGGTTGTCGTCAGCTCGTGTCGTGAGATGTTGGG
TTAAGTCCCGCAACGAGCGCAACCCTCGTTCTATGTTGCCAGCACGTGATGGTGGGGACTCATAGGAGACTGCCGGGGTCAACTCGGAGGAAGGTGGGGA
TGACGTCAAATCATCATGCCCCTTATGTCTTGGGCTTCACGCATGCTACAATGGCCGGTACAAAGGGTTGCGATACTGTGAGGTGGAGCTAATCCCAAAA
AGCCGGTCTCAGTTCGGATTGAGGTCTGCAACTCGACCTCATGAAGTCGGAGTCGCTAGTAATCGCAGATCAGCAACGCTGCGGTGAATACGTTCCCGGG
CCTTGTACACACCGCCCGTCAAGTCACGAAAGTTGGTAACACCCGAAGCCGGTGGCCTAACCCCTTGTGGGAGGGAGCC

>EB178
AAGGGGTTAAGCCACTTCCTTCGGGTGTTACCCACTTTCGTCCCTTGACGGGCGGTGTGTACAACGCCCGAGAACGTATTCACCGCAGCGGTGCTGATCT
GCGATTACTATCGACTCCGACTTCATGAAGTCGAGTTGCACACCTCAATCTGAACTGAGACCTGCTTTTTGGGATTAGCTCCACCTCACAGTATCGCAAC
CCTTTGTACCGTCCATTGTAGCATGCGTCAAGCCCAAGACGTAAGGGGCATGATGATTTGACGTCCTCCCTACCTTCCTCCGAGTTGACCCCGGCAGTCT
CCTATGACTCCCCACCATCACGTGCTGGCAACATAAAACGAGGGTTGCGCTCGTTGCGGGACTTAACCCAACATCTCACGACACGAGCTGACGACAACCA
TGCACCACCTGTCCACCGACCCCGAAGGGAAACCCCATCTCTGGAGCGGTCCGGTGAATGTCAAGCCTTGGTAAGGTTCTTCGCGTTGCATCGAATTAAT
CCGTCATGCTCCGCCGCTTGTGCGGGACCCCCGTCAATTCCTTTGAGTTTTAGCCTTGCGGCCGTACTCCCCAGGCGGGGCACTTAATGCGTTAGCTACG
GCGCGGATAACGTGGAATGTCCCCCACACCTAGTGCCCAACGTTTACGGCATGGACTACCAGGGTATCTAATCCTGTTCGCTCCCCATGCTTTCGCTCCT
CAGCGTCAGTAACAGCCCAGAGACCTGCCTTCGCCATCGGTGTTCCTCCTGATATCTGCGCATTTCACCGCTACACCAGGAATTCCAGTCTCCCCTACTG
CACTCTAGTCTGTCCGTACCCACTGCAGACCCGGGGTTGAGCCCCGGGCTTTCACAGCAGACGCGACAAACCGCCTACGAGCTCTTTACGCCCAATAATT
CCGGACAACGCTTGCGCCCTACGTATTACCGCGGCTGCTGGCACGTAGTTAGCCGGCGCTTCTTCTGCAGGTACCGTCACTCTCGCTTCTTCCCTACTGA
AAGAGGTTTACAACCCGAAGGCCGTCATCCCTCACGCGGCGTCGCTGCATCAGGCTTCCGCCCATTGTGCAATATTCCCCACTGCTGCCTCCCGTAGGAG
TCTGGGCCGTGTCTCAGTCCCAGTGTGGCCGGTCACCCTCTCAGGCCGGCTACCCGTCGTCGCCTTGCTGAGCCATTACCCCACCAACAAGCTGATAGGC
CGTGAGCCCATCCAAAACCAGTAAAACCCTTTCCACCACCCACCATGCGGCAGGAAGTAGTATCCAGTATTAGACCCAGTTTCCCAGGCTTATCCCAGAG
TCAAGGGCAGGTTACTCACGTATTACTCACCCGTTCGCCACTAATCCACCCAGCAAGCTGGGCATCATCGCTCGACTGCA

>EB179
CGAGCGGTAGCACAGGGAGCTTGCTCCTGGGTGACGAGCGGCGGACGGGTGAGTAATGTCTGGGAAACTGCCTGATGGAGGGGGATAACTACTGGAAACG
GTAGCTAATACCGCATAACGTCGCAAGACCAAAGAGGGGGACCTTCGGGCCTCTTGCCATCAGATGTGCCCAGATGGGATTAGCTAGTAGGTGGGGTAAC
GGCTCACCTAGGCGACGATCCCTAGCTGGTCTGAGAGGATGACCAGCCACACTGGAACTGAGACACGGTCCAGACTCCTACGGGAGGCAGCAGTGGGGAA
TATTGCACAATGGGCGCAAGCCTGATGCAGCCATGCCGCGTGTATGAAGAAGGCCTTCGGGTTGTAAAGTACTTTCAGCGGGGAGGAAGGTGTTGAGGTT
AATAACCTCAGCAATTGACGTTACCCGCAGAAGGAAGACACCGGCTAACTCCGTGCCAGCAGCCGCGGTAATACGGAGGGTGCAAGCGTTAATCGGAATT
TACTGGGCGTAAAGCGCACGCAGGCGGTCTGTCAAGTCGGATGTGAAATCCCCGGGCTCAACCTGGGAACTGCATTCGAAACTGGCAGGCTAGAGTC
TTGTAGAGGGGGGTAGAATTCCAGGTGTAGCGGTGAAATGCGTAGAGATCTGGAGGAATACCGGTGGCGAAGGCGGCCCCCTGGACAAAGACTGACGCTC
AGGTGCGAAAGCGTGGGGAGCAAACAGGATTAGATACCCTGGTAGTCCACGCCGTAAACGATGTCGACTTGGAGGTTGTTCCCTTGAGGAGTGGCTTCCG
GAGCTAACGCGTTAAGTCGACCGCCTNGGGGAGTACGGCCGCAAGGTTAAAACTCAAATGAATTGACGGGGGCCCGCACAAGCGGTGGAGCATGTGGTTT
AATTCGATGCAACGCGAAGAACCTTACCTACTCTTGACATCCAGAGAACTTAGCAGAGATGGATTGGTGCCTTCGGGAACTCTGAGACAGGTGCTGCATG
GCTGTCGTCAGCTCGTGTTGTGAAATGTTGGGTTAAGTCCCGCAACGAGCGCAACCCTTATCCTTTGTTGCCAGCGGTTCGGCCGGGAACTCAAAGGAGA
CTGCCAGTGATAAACTGGAGGAAGGTGGGGATGACGTCAAGTCATCATGGCCCTTACGAGTAGGGCTACACACGTGCTACAATGGCGCATACAAAGAGAA
GCGACCTCGCGAGAGCAAGCGGACCTCATAAAGTGCGTCGTAGTCCGGATTGGAGTCTGCAACTCGACTCCATGAAGTCGGAATCGCTAGTAATCGTAGA
TCAGAATGCTACGGTGAATACGTTCCCGGGCCTTGTACACACCGCCCGTCACACCATGGGAGTGGGTTGCAAAAGAAGTAGGTAGCTTAACCTTCGGGAG
GGC

>EB180
GGTAGCGCCCTCCCGAAGGTTAAGCTACCTACTTCTTTTGCAACCCACTCCCATGGTGTGACGGGCGGTGTGTACAAGGCCCGGGAACGTATTCACCGTA
GCATTCTGATCTACGATTACTAGCGATTCCGACTTCACGGAGTCGAGTTGCAGACTCCGATCCGGACTACGACATACTTTGTGAGGTCCGCTTGCTCTCG
CGAGGTCGCTTCTCTTTGTATATGCCATTGTAGCACGTGTGTAGCCCTACTCGTAAGGGCCATGATGACTTGACGTCATCCCCACCTTCCTCCAGTTTAT
CACTGGCAGTCTCCTTTGAGTTCCCGGCCGAACCGCTGGCAACAAAGGATAAGGGTTGCGCTCGTTGCGGGACTTAACCCAACATTTCACAACACGAGCT
GACGACAGCCCATGCAGCACCTGTCTCAGAGTTCCCGAAGGCACCAAAGCATCTCTGCTAAGTTCTCTGGATGTCAAGAGTAGGTAAGGTTCTTCGCGTT
GCATCGAATTAAACCACATGCTCCACCGCTTGTGCGGGCCCCCGTCAATTCATTTGAGTTTTAACCTTGCGGCCGTACTCCCCAGGCGGTCGACTTAAC
GCGTTAGCTCCGGAAGCCACTCCTCAAGGGAACAACCTCCAAGTCGACATCGTTTACGGCGTGGACTACCAGGGTATCTAATCCTGTTTGCTCCCCACGC
TTTCGCACCTGAGCGTCAGTCTTTGTCCAGGGGGCCGCCTTCGCCACCGGTATTCCTCCAGATCTCTACGCATTTCACCGCTACACCTGGAATTCTACCC
CCCTCTACAAGACTCTAGCCTGCCAGTTTCGAATGCAGTTCCCAGGTTGAGCCCGGGGATTTCACATCCGACTTGACAGACCGCCTGCGTGCGCTTTACG
CCCAGTAATTCCGATTAACGCTTGCACCCTCCGTATTACCGCGGCTGCTGGCACGGAGTTAGCCGGTGCTTCTTCTGCGAGTAACGTCAATCACTGCGGT
TATTAACCACAATGCCTTCCTCCTCGCTGAAAGTACTTTACAACCCGAAGGCCTTCTTCATACACGCGGCATGGCTGCATCAGGCTTGCGCCCATTGTGC
AATATTCCCCACTGCTGCCTCCCGTAGGAGTCTGGACCGTGTCTCAGTTCCAGTGTGGCTGGTCATCCTCTCAGACCAGCTAGGGATCGTCGCCTAGGTG
AGCCATTACCCCACCTACTAGCTAATCCCATCTGGGCACATCTGATGGCAAGAGGCCCGAAGGTCCCCCTCTTTGGTCTTGCGACGTTATGCGGTATTAG

CTACCGTTTCCAGTAGTTATCCCCCTCCATCAGGCAGTTTCCCAGACATTACTCACCCGTCCGCCGCTCGTCACCCGAGAGCAAGCTCTCTGTGCTACCG
CTCGACTTGCA

>EB181
TGGGGTAACGGCTCACCTATGCGACAATACCTATCTGGTCTGAGAGGATAAGCCGCCACACTGTAACTGAGACACGGTCCAGACTCCTACAGGAGGCAGC
AGTGGGGAATATTGCGCAATGGGCGCAAGCCTGATGCGCCCATGTCGCGTGTGTGAAAAAAGCCTTCTGGTTGTAAAGTACTTTTCAGCGAGGGAGGAAG
GGCATTGCCGGTTAATAACCGCCAGTGATTGACGTTACTCTCAGAAAAAACACCGGCTAACTCCGTGCCCAGCAGCCGCGGTAATACGGAGGGTGCAAGC
GTTAATCGGAATTACTGGGCGTAAAGCGCACGCAGGCGGTCTGTCAAGTCGGATGTGAAATCCCCCGGGCTCAACCTGGGAACTGCATTCGAAACTGGCA
GGCTAGAGTCTTGTAGAGGGGGGTAGAATTCCAGGTGTAGCGGTGAAATGCGTAGAGATCTGGAGGAATACCGGTGGCGAAGGCGGCCCCCTGGACAAAG
ACTGACGCTCAGGTGCGAAAGCGTGGGGAGCAAACAGGATTAGATACCCTGGTAGTCCACGCCGTAAACGATGTCGACTTGGAGGTTGTTCCCTTGAGGA
GTGGCTTCCGGAGCTAACGCGTTAAGTCGACCGCCTGGGGAGTACGGCCGCAAGGTTAAAACTCAAATGAATTGACGGGGGCCCGCACAAGCGGTGGAGC
ATGTGGTTTAATTCGATGCAACGCGAAGAACCTTACCTACTCTTGACATCCACAGAATTTAGCAGAGATGCTTTAGTGCCTTCGGGAACTGTGAGACAGG
TGCTGCATGGCTGTCGTCAGCTCGTGTTGTGAAATGTTGGGTTAAGTCCCGCAACGAGCGCAACCCTTATCCTTTGTTGCCAGCGGTTCGGCCGGGAACT
CAAAGGAGACTGCCAGTGATAAACTGGAGGAAGGTGGGGATGACGTCAAGTCATCATGGCCCTTACGAGTAGGGCTACACACGTGCTACAATGGCGCATA
CAAAGAGAAGCGACCTCGCGAGAGCAAGCGGACCTCATAAAGTGCGTCGTAGTCCGGATTGGAGTCTGCAACTCGACTCCATGAAGTCGGAATCGCTAGT
AATCGTAGATCAGAATGCTACGGTGAATACGTTCCCGGGCCTTGTACACACCGCCCGTCACACCATGGGAGTGGGTTGCAAAAGAAGTAGGTAGCTTAAC
CTTCGGGAGGGCGC

>EB182
CGAACTGATTAAAAGCTTGCTTCTATGACGTTAGCGGCGGACGGGTGAGTAACACGTGGGCAACCTGCCTGTAAGACTGGGATAACTTCGGGAAACCGAA
GCTAATACCGGATAGGATCTTCTCCTTCATGGGAGATGATTGAAAGATGGTTTCGGCTATCACTTACAGATGGGCCCGCGGTGCATTAGCTAGTTGGTGA
GGTAACGGCTCACCAAGGCAACGATGCATAGCCGACCTGAGAGGGTGATCGGCCACACTGGGACTGAGACACGGCCCAGACTCCTACGGGAGGCAGCAGT
AGGGAATCTTCCGCAATGGACGAAAGTCTGACGGAGCAACGCCGCGTGAGTGATGAAGGCTTTCGGGTCGTAAAACTCTGTTGTTAGGGAAGAACAAGTA
CAAGAGTAACTGCTTGTACCTTGACGGTACCTAACCAGAAAGCCACGGCTAACTACGTGCCAGCAGCCGCGGTAATACGTAGGTGGCAAGCGTTATCCGG
AATTATTGGGCGTAAAGCGCGCGCAGGCGGTTTCTTAAGTCTGATGTGAAAGCCCACGGCTCAACCGTGGAGGGTCATTGGAAACTGGGGAACTTGAGTG
CAGAAGAGAAAAGCGGAATTCCACGTGTAGCGGTGAAATGCGTAGAGATGTGGAGGAACACCAGTGGCGAAGGCGGCTTTTTGGTCTGTAACTGACGCTG
AGGCGCGAAAGCGTGGGGAGCAAACAGGATTAGATACCCTGGTAGTCCACGCCGTAAACGATGAGTGCTAAGTGTTAGAGGGTTTCCGCCCTTTAGTGCT
GCAGCTAACGCATTAAGCACTCCGCCTGGGGAGTACGGTCGCAAGACTGAAACTCAAAGGAATTGACGGGGGCCCGCACAAGCGGTGGAGCATGTGGTTT
AATTCGAAGCAACGCGAAGAACCTTACCAGGTCTTGACATCCTCTGACAACTCTAGAGATAGAGCGTTCCCCTTCGGGGGACAGAGTGACAGGTGGTGCA
TGGTTGTCGTCAGCTCGTGTCGTGAGATGTTGGGTTAAGTCCCGCAACGAGCGCAACCCTTGATCTTAGTTGCCAGCATTTAGTTGGGCACTCTAAGGTG
ACTGCCGGTGACAAACCGGAGGAAGGTGGGGATGACGTCAAATCATCATGCCCCTTATGACCTGGGCTACACACGTGCTACAATGGATGGTACAAAGGGC
TGCAAGACCGCGAGGTCAAGCCAATCCCATAAAACCATTCTCAGTTCGGATTGTAGGCTGCAACTCGCCTACATGAAGCTGGAATCGCTAGTAATCGCGG
ATCAGCATGCCGCGGTGAATACGTTCCCGGGCCTTGTACACACCGCCCGTCACACCACGAGAGTTTGTAACATACCGAAGTCGGTGGAGTAACCGTAAGG
AGCTA

>EB183
CGGCTGGCTCCAAAAGGTTACCTCACCGACTTCGGGTGTTACAAACTCTCGTGGTGTGACGGGCGGTGTGTACAAGGCCCGGGAACGTATTCACCGCGGC
ATGCTGATCCGCGATTACTAGCGATTCCGGCTTCATGTAGGCGAGTTGCAGCCTACAATCCGAACTGAGAACGACTTTATCGGATTAGCTCCCTCTCGCG
AGTTGGCAACCGTTTGTATCGTCCATTGTAGCACGTGTGTAGCCCAGGTCATAAGGGGCATGATGATTTGACGTCATCCCCACCTTCCTCCGGTTTGTCA
CCGGCAGTCACCTTAGAGTGCCCAACTAAATGATGGCAACTAAGATCAAGGGTTGCGCTCGTTGCGGGACTTAACCCAACATCTCACGACACGAGCTGAC
GACAACCATGCACCACCTGTCACCGTTGCCCCCGAAGGGGAAACTATATCTCTACAGTGGTCAACGGGATGTCAAGACCTGGTAAGGTTCTTCGCGTTGC
TTCGAATTAAACCACATGCTCCACCGCTTGTGCGGGCCCCCGTCAATTCCTTTGAGTTTCAGTCTTGCGACCGTACTCCCCAGGCGGAGTGCTTAATGCG
TTAGCTGCAGCACTAAGGGGCGGAAACCCCCTAACACTTAGCACTCATCGTTTACGGCGTGGACTACCAGGGTATCTAATCCTGTTTGCTCCCCACGCTT
TCGCGCCTCAGCGTCAGTTACAGACCAGAAAGTCGCCTTCGCCACTGGTGTTCCTCCAAATCTCTACGCATTTCACCGCTACACTTGGAATTCCACTTTC
CTCTTCTGCACTCAAGTCCCCCAGTTTCCAATGACCCTCCACGGTTGAGCCGTGGGCTTTCACATCAGACTTAAAGGACCGCCTGCGCGCGCTTTACGCC
CAATAATTCCGGACAACGCTTGCCACCTACGTATTACCGCGGCTGCTGGCACGTAGTTAGCCGTGGCTTTCTAATAAGGTACCGTCAAGGTACAGCCAGT
TACTACTGTACTTGGTTCTTCCCTTACAACAGAGTTTTACGATCCGAAAACCTTCTTCACTCACGCGGCGTTGCTCCATCAGGCTTTCGCCCATTGTGGA
AGATTCCCTACTGCTGCCTCCCGTAGGAGTCTGGGCCGTGTCTCAGTCCCAGTGTGGCCGATCACCCTCTCAGGTCGGCTACGCATCGTCGCCTTGGTGA
GCCGTTACCTCACCAACTAGCTAATGCGCCGCGGGCCCATCCTATAGCGACAGCCGAAACCGTCTTTCAGTCTTTCACCATGAAGCAAAAGAGATTATTC
GGTATTAGCCCCGGTTTCCCGGAGTTATCCCAAACTATAGGGTAGGTTGCCCACGTGTTACTCACCCGTCCGCCGCTAACGTCAAAGGAGCAAGCTCCTT
TTCTGTTCGCTCGACTTGCA

>EB184
CGGCTGGCTCCATAAGGTTACCTCACCGACTTCGGGTGTTACAAACTCTCGTGGTGTGACGGGCGGTGTGTACAAGGCCCGGGAACGTATTCACCGCGGC
ATGCTGATCCGCGATTACTAGCGATTCCAGCTTCATGTAGGCGAGTTGCAGCCTACAATCCGAACTGAGAACGGTTTTATCAGATTAGCTCCATCTCGCG
ACTTCGCAACCGTTTGTACCGTCCATTGTAGCACGTGTGTAGCCCAGGTCATAAGGGGCATGATGATTTGACGTCATCCCCACCTTCCTCCGATTTGTCA
TCGGCAGTCTCCTTAGAGTGCCCAACTTAATGATGGCAACTAAGGACAAGGGTTGCGCTCGTTGCGGGACTTAACCCAACATCTCACGACACGAGCTGAC
GACAACCATGCACCACCTGTCACCACTGTCCCCGAAGGGAAAGCTATGTCTCCATAGCGGTCAGTGGGATGTCAAGACCTGGTAAGGTTCTTCGCGTTGC
TTCGAATTAAACCACATGCTCCACCGCTTGTGCGGGCCCCCGTCAATTCCTTTGAGTTTCAGTCTTGCGACCGTACTCCCCAGGCGGAGTGCTTAATGCG
TTAGCTGCAGCACTAAGGGGCGGAAACCCCCTAACACTTAGCACTCATCGTTTACGGCGTGGACTACCAGGGTATCTAATCCTGTTTGCTCCCCACGCTT
TCGCGCCTCAGCGTCAGTTACAGACCAGAAAGTCGCCTTCGCCACTGGTGTTCCTCCAAATCTCTACGCATTTCACCGCTACACTTGGAATTCCACTTTC
CTCTTCTGCACTCAAGTCCTCCAGTTTCCAATGACCCTCCTCGGTTGAGCCGAGGGCTTTCACATCAGACTTAAAGGACCGCCTGCGCGCGCTTTACGCC
CAATAATTCCGGACAACGCTTGCCACCTACGTATTACCGCGGCTGCTGGCACGTAGTTAGCCGTGGCTTTCTAATAAGGTACCGTCAAGGTACAGCCAGT
TACTACTGTACTTGTTCTTCCCTTACAACAGAGTTTTACGATCCGAAAACCTTCTTCACTCACGCGGCGTTGCTCCATCAGGCTTTCGCCCATTGTGGAA
GATTCCCTACTGCTGCCTCCCGTAGGAGTCTGGGCCGTGTCTCAGTCCCAGTGTGGCCGATCACCCTCTCAGGTCGGCTACGCATCGTCGCCTTGGTGAG
CCGTTACCTCACCAACTAGCTAATGCGCCGCGGGCCCATCCTATAGCGATAGCGTAAACCATCTTTCAACAATCTAGCAGGAGCTAAATTATATCATTCG
GTATTAGCCCCGGTTTCCCGGAGTTATCCCCAACTATAGGGCAGGTTGCCCACGTGTTACTCACCCGTCCGCCGCTAACTTCTGGGAGCAAGCTCCCATC
CGTCCGCTCGA

>EB185
GTGTGTACAAGGGCCGGGAACGTATTCACCGCGGCATGCTGATCCGCGATTACTAGCGATTCCGGCTTCATGTAGGCGAGTTGCAGCCTACAATCCGAAC
TGAGAACGACTTTATCGGATTAACTCCCTCTCGCGAGTTGGCAACCGTTTGTATCGTCCATTGTAGCACGTGTGTAGCCCAGGTCATAAGGGGCATGATG
ATTTGACGTCATCCCCACCTTCCTCCGGTTTGTCACCGGCAGTCACCTTAAAGTGCCCAACTAAATGATGGCAACTAAGATCAAGGGTTGCGCTCGTTGC
GGGACTTAACCCAACATCTCACGACACGAGCTGACGACAACCATGCACCACCTGTCACCGTTGTCCCCGAAGGGAAAACTGTATCTCTACAGTGGTCAAT
GGGATGTCAAGACCTGGTAAGGTTCTTTCGCGTTGCTTCCGAATTAAACCCACATGCTCCACCGCTTGTGCGGGCCCCCCGTCAATTCCTTTGAGTTTTC
AGTCTTGCGACCGTACTCCCCCAGGCGGAGTGCTTAATGCGTTAGCTGCAGCACTAAGGGGCGGAAACCCCCTAACACTTAGCACTCATCGTTTACGGCG
TGGACTACCAGGGTATCTAATCCTGTTTGCTCCCCACGCTTTCGCGCCTCAGTGTCAGTTACAGACCAGATAGTCGCCTTCGCCACTGGTGTTCCTCCAA
ATCTCTACGCATTTCACCGCTACACTTGGAATTCCACTATCCTCTTCTGCACTCAAGTCTCCCAGTTTCCAATGACCCTCCACGGTTGAGCCGTGGGCTT

TCACATCAGACTTAAGAAACCACCTGCGCGCGCTTTACGCCCAATAATTCCGGACAACGCTTGCCACCTACGTATTACCGCGGCTGCTGGCACGTAGTTA
GCCGTGGCTTTCTAATAAGGTACCGTCAAGGTACAGCCAGTTACTACTGTACTTGTTCTTCCCTTACAACAGAGTTTTACGAACCGAAATCCTTCTTCAC
TCACGCGGCGTTGCTCCATCAGGCTTTCGCCCATTGTGGAAGATTCCCTACTGCTGCCTCCCGTAGGAGTCTGGGCCGTGTCTCAGTCCCAGTGTGGCCG
ATCACCCTCTCAGGTCGGCTACGCATCGTCGCCTTGGTGAGCCGTTACCTCACCAACTAGCTAATGCGCCGCGGGCCCATCCTATAGCGACAGCCGAAAC
CGTCTTTTAGAATTGTCTCATGAGAGACAACAAGTTATTCGGTATTAGCCCCGGTTTCCCGGAGTTATCCCAAACTATAGGGTAGGTTGCCCACGTGTTA
CTCACCCGTCCGCCGCTAACGTCAAAGGAGCAAGCTCCTTATCTGTTCGCTCGAC

>EB186
TCGAGCGAACAGAGAAGGAGCTTGCTCCTTTGACGTTAGCGGCGGACGGGTGAGTAACACGTGGGCAACCTACCCTATAGTTTGGGATAACTCCGGGAAA
CCGGGGCTAATACCGAATAACTTGTTGTCTCTCATGAGACAATTCTAAAAGACGGTTTCTGCTGTCGCTATAGGATGGGCCCGCGGCGCATTAGCTAGTT
GGTGAGGTAACGGCTCACCAAGGGGACGATGCGTAGCCGAACTGAGAGGGTGATCGGCCACACTGGGACTGAGACACGGCCCAGACTCCTACGGGAGGCA
GCAGTAGGGAATCTTCCACAATGGGCGAAAGCCTGATGGAACAACGCCGCGTGAGTGAAGAAGGATTTCGGGTCGTAAAACTCTGGTGTAAGGGAAGAAC
AAGTACAGTAGTAACTGGCTGTACCTTGACGGTACCTTATTAGAAAGCCACGGCTAACTACGTGCCAGCAGCCGCGGTAATACGTAGGTGGCAAGCGTTG
TCCGGAATTATTGGGCGTAAAGCGCGCGCAGGTGGTTTCTTAAGTCTGATGTGAAAGCCCACGGCTCAACCGTGGAGGGTCATTGGAAACTGGGAGACTT
GAGTGCAGAAGAGGATAGTGGAATTCCAAGTGTAGCGGTGAAATGCGTAGAGATTTGGAGGAACACCAGTGGCGAAGGCGACTATCTGGTCTGTAACTGA
CACTGAGGCGCGAAAGCGTGGGGAGCAAACAGGATTAGATACCCTGGTAGTCCACGCCGTAAACGATGAGTGCTAAGTGTTAGGGGGTTTCCGCCCCCTT
AGTGCTGCAGCTAACGCATTAAGCACTCCGCCTGGGGAGTACGGTCGCAAGACTGAAACTCAAAGGAATTGACGGGGGCCCGCACAAGCGGTGGAGCATG
TGGTTTAATTCGAAGCAACGCGAAGAACCTTACCAGGTCTTGACATCCCATTGACCACTGTAGAGATACAGTTTTCCCTTCGGGGACAACGGTGACAGGT
GGTGCATGGTTGTCGTCAGCTCGTGTCGTGAGATGTTGGGTTAAGTCCCGCAACGAGCGCAACCCTTGATCTTAGTTGCCATCATTTAGTTGGGCACTCT
AAGGTGACTGCCGGTGACAAACCGGAGGAAGGTGGGGATGACGTCAAATCATCATGCCCCTTATGACCTGGGCTACACACGTGCTACAATGGACGATACA
AACGGTTGCCAACTCGCGAGAGGGAGCTAATCCGATAAAGTCGTTCTCAGTTCGGATTGTAGGCTGCAACTCGCCTACATGAAGCCGGAATCGCTAGTAA
TCGCGGATCAGCATGCCGCGGTGAATACGTTCCCGGGCCTTGTACACACCGCCCGTCACACCACGAGAGTTTGTAACACCCGAAGTCGGTGAGGTAACCT
TTTGGAGCC

>EB187
TGCTCAGGATGAACGCTGGCGGCGTGCCTAATACATGCAAGTCGAGCGAACTGATTAGAAGCTTGCTTCTATGACGTTAGCGGCGGACGGGTGAGTAACA
CGTGGGCAACCTGCCTGTAAGACTGGGATAACTTCGGGAAACCGAAGCTAATACCGGATAGGATCTTCTCCTTCATGGGAGATGATTGAAAGATGGTTTC
GGCTATCACTTACAGATGGGCCCGCGGTGCATTAGCTAGTTGGTGAGGTAACGGCTCACCAAGGCAACGATGCATAGCCGACCTGAGAGGGTGATCGGCC
ACACTGGGACTGAGACACGGCCCAGACTCCTACGGGAGGCAGCAGTAGGGAATCTTCCGCAATGGACGAAAGTCTGACGGAGCAACGCCGCGTGAGTGAT
GAAGGCTTTCGGGTCGTAAAACTCTGTTGTTAGGGAAGAACAAGTACGAGAGTAACTGCTCGTACCTTGACGGTACCTAACCAGAAAGCCACGGCTAACT
ACGTGCCAGCAGCCGCGGTAATACGTAGGTGGCAAGCGTTATCCGGAATTATTGGGCGTAAAGCGCGCGCAGGCGGTTTCTTAAGTCTGATGTGAAAGCC
CACGGCTCAACCGTGGAGGGTCATTGGAAACTGGGGAACTTGAGTGCAGAAGAGAAAAGCGGAATTCCACGTGTAGCGGTGAAATGCGTAGAGATGTGGA
GGAACACCAGTGGCGAAGGCGGCTTTTTGGTCTGTAACTGACGCTGAGGCGCGAAAGCGTGGGGAGCAAACAGGATTAGATACCCTGGTAGTCCACGCCG
TAAACGATGAGTGCTAAGTGTTAGAGGGTTTCCGCCCTTTAGTGCTGCAGCTAACGCATTAAGCACTCCGCCTGGGGAGTACGGTCGCAAGACTGAAACT
CAAAGGAATTGACGGGGGCCCGCACAAGCGGTGGAGCATGTGGTTTAATTCGAAGCAACGCGAAGAACCTTACCAGGTCTTGACATCCTCTGACAACTCT
AGAGATAGAGCGTTCCCCTTCGGGGGACAGAGTGACAGGTGGTGCATGGTTGTCGTCAGCTCGTGTCGTGAGATGTTGGGTTAAGTCCCGCAACGAGCGC
AACCCTTGATCTTAGTTGCCAGCATTTAGTTGGGCACTCTAAGGTGACTGCCGGTGACAAACCGGAGGAAGGTGGGGATGACGTCAAATCATCATGCCCC
TTATGACCTGGGCTACACACGTGCTACAATGGATGGTACAAAGGGCTGCAAGACCGCGAGGTCAAGCCAATCCCATAAAACCATTCTCAGTTCGGATTGT
AGGCTGCAACTCGCCTACATGAAGCTGGAATCGCTAGTAATCGCGGATCAGCATGCCGCGGTGAATACGTTCCCGGGCCTTGTACACACCGCCCGTCACA
CCACGAGAGTTTGTAACACCCGAAGTCGGTGGAGTAACCGTAAGGAGCTAGCCGCCTAAGGTGGGACAGATGATTGGGGTGA

>EB188
CTGGGATAACTTCGGGAAACCGAAGCTAATACCGGATAGGATCTTCTCCTTCATGGGAGATGATTGAAAGATGGTTTCGGCTATCACTTACAGATGGGCC
CGCGGTGCATTAGCTAGTTGGTGAGGTAACGGCTCACCAAGGCAACGATGCATAGCCGACCTGAGAGGGTGATCGGCCACACTGGGACTGAGACACGGCC
CAGACTCCTACGGGAGGCAGCAGTAGGGAATCTTCCGCAATGGACGAAAGTCTGACGGAGCAACGCCGCGTGAGTGATGAAGGCTTTCGGGTCGTAAAAC
TCTGTTGTTAGGGAAGAACAAGTACGAGAGTAACTGCTTGTACCTTGACGGTACCTAACCAGAAAGCCACGGCTAACTACGTGCCAGCAGCCGCGGTAAT
ACGTAGGTGGCAAGCGTTATCCGGAATTATTGGGCGTAAAGCGCGCGCAGGCGGTTTCTTAAGTCTGATGTGAAAGCCCACGGCTCAACCGTGGAGGGTC
ATTGGAAACTGGGGAACTTGAGTGCAGAAGAGAAAAGCGGAATTCCACGTGTAGCGGTGAAATGCGTAGAGATGTGGAGGAACACCAGTGGCGAAGGCGG
CTTTTTGGTCTGTAACTGACGCTGAGGCGCGAAAGCGTGGGGAGCAAACAGGATTAGATACCCTGGTAGTCCACGCCGTAAACGATGAGTGCTAAGTGTT
AGAGGGTTTCCGCCCTTTAGTGCTGCAGCTAACGCATTAAGCACTCCGCCTGGGGAGTACGGTCGCAAGACTGAAACTCAAAGGAATTGACGGGGGCCCG
CACAAGCGGTGGAGCATGTGGTTTAATTCGAAGCAACGCGAAGAACCTTACCAGGTCTTGACATCCTCTGACAACTCTAGAGATAGAGCGTTCCCCTTCG
GGGGACAGAGTGACAGGTGGTGCATGGTTGTCGTCAGCTCGTGTCGTGAGATGTTGGGTTAAGTCCCGCAACGAGCGCAACCCTTGATCTTAGTTGCCAG
CATTTAGTTGGGCACTCTAAGGTGACTGCCGGTGACAAACCGGAGGAAGGTGGGGATGACGTCAAATCATCATGCCCCTTATGACCTGGGCTACACACGT
GCTACAATGGATGGTACAAAGGGCTGCAAGACCGCGAGGTCAAGCCAATCCCATAAAACCATTCTCAGTTCGGATTGTAGGCTGCAACTCGCCTACATGA
AGCTGGAATCGCTAGTAATCGCGGATCAGCATGCCGCGGTGAATACGTTCCCGGGCCTTGTACACACCGCCCGTCACACCACGAGAGTTTGTAACACCCG
AAGTCGGTGGAGTAACCGT

>EB189
ACATGCAGTCGAGCGGGCACTTTCGGGTGTCAGCCGCGGACGGGTGAGTAACGCGTGAGAACGTGCCCTCTGGTTCGGAATAACTCAGGGAAACTTGATC
TAATACCGGATACGCCCTTTTGGGGAAAGGCTTGCTGCCGGAAGATCGGCCCGCGTCTGATTAGCTAGTTGGAGAGGTAACGGCTCACCAGGGCGACTAT
CTGTAGCTGGTCTGAGAAGATGATCAGACACACTGGGACTGAGACACGGCCCAGACTCCTACGGGAGGCAGCAGTGGGGAATATTGGACAATGGGCGCAA
GCCTGATCCAGCCATGCCGCGTGAGTGATGAAGGCCTTAGGGTTGTAAAGCTCTTTTATCCGGGACGATAATGACTGTACCGGAGGAATAAGCCCCGGCT
AACTTCGTGCCAGCAGCCGCGGTAATACGAAGGGGGCTAGCGTTGCTCGGAATCACTGGGCGTAAAGGGCGCGTAGGCGGCGTTTTAAGTCGGGGGTGAA
AGCCTGTGGCTCAACCACAGAATGGCCTTCGATACTGGGACGCTTGAGTATGGTAGAGGTTGGTGGAACTGCGAGTGTAGAGGTGAAATTCGTAGATATT
CGCAAGAACACCGGTGGCGAAGGCGGCCAACTGGACCATTACTGACGCTGAGGCGCGAAAGCGTGGGGAGCAAACAGGATTAGATACCCTGGTAGTCCAC
GCCGTAAACGATGAATGCCAGCTGTTGGGGTGCTTGCACCTCAGTAGCGCAGCTAACGCTTTGAGCATTCCGCCTGGGGAGTACGGTCGCAAGATTAAAA
CTCAAAGGAATTGACGGGGGCCCGCACAAGCGGTGGAGCATGTGGTTTAATTCGAAGCAACGCGCAGAACCTTACCATCCTTTGACATGGCGTGTTATGG
GGAGAGATTCCCAGTCCTCTTCGGAGGCGCGCACACAGGTGCTGCATGGGTGTCGTCAGCTCGTGTCGAGAGATGTTGGGTTAAGTCCCGCAACGAGCGC
AACCCACGTCCTTAGTTGCCATCATTCAGTTGGGCACTCTAGGGAGACTGCCGGTGATAAGCCGCGAGGAAGGTGTGGATGACGTCAAGTCCTCATGGCC
CTTACGGGATGGGCTACACACGTGCTACAATGGCGGTGACAGTGGGACGCGAAGGGGCGACCTGGAGCAAATCCCCAAAAGCCGTCTCAGTTCGGATTGC
ACTCTGCAACTCGGGTGCATGAAGGCGGAATCGCTAGTAATCGTGGATCAGCATGCCACGGTGAATACGTTCCCGGGCCTTGTACACCCCGCCCGTCACC
CCCTGGGAGTTGGTCTTCCCCGGAGGCGCTGCAGCCAACCGCAAGGAGGCAGGCG

>EB190
GATGTGACGAGCGGTGTGTACAATGTACGGGAACGTATTCACCGCGGCATGCTGATCCGCGATTACTAACGATTCCAGCTTCATGTAGGCGAGTTGCAGC
CTACAATCCGAACTGAGAATGGTTTTATGGGATTGGCTTGACCTCGCGGTCTTGCAGCCCTTTGTACCATCCATTGTAGCACGTGTGTAGCCCAGGTCAT
AAGGGGCATGATGATTTGACGTCATCCCCACCTTCCTCCGGTTTGTCACCGGCAGTCACCTTAGAGTGCCCAACTAAATGCTGGCAACTAAGATCAAGGG
TTGCGCTCGTTGCGGGACTTAACCCAACATCTCACGACACGAGCTGACGACAACCATGCACCACCTGTCACTCTGTCCCCCGAAGGGGAACGCTCTATCT

CTAGAGTTGTCAGAGGATGTCAAGACCTGGTAAGGTTCTTCGCGTTGCTTCGAATTAAACCACATGCTCCACCGCTTGTGCGGGCCCCCGTCAATTCCTT
TGAGTTTCAGTCTTGCGACCGTACTCCCCAGGCGGAGTGCTTAATGCGTTAGCTGCAGCACTAAAGGGCGGAAACCCTCTAACACTTAGCACTCATCGTT
TACGGCGTGGACTACCAGGGTATCTAATCCTGTTTGCTCCCCACGCTTTCGCGCCTCAGCGTCAGTTACAGACCAAAAAGCCGCCTTCGCCACTGGTGTT
CCTCCACATCTCTACGCATTTCACCGCTACACGTGGAATTCCGCTTTTCTCTTCTGCACTCAAGTTCCCCAGTTTCCAATGACCCTCCACGGTTGAGCCG
TGGGCTTTCACATCAGACTTAAGAAACCGCCTGCGCGCGCTTTACGCCCAATAATTCCGGATAACGCTTGCCACCTACGTATTACCGCGGCTGCTGGCAC
GTAGTTAGCCGTGGCTTTCTGGTTAGGTACCGTCAAGGTACAAGCAGTTACTCTTGTACTTGTTCTTCCCTAACAACAGAGTTTTACGACCCGAAAGCCT
TCATCACTCACGCGGCGTTGCTCCGTCAGACTTTCGTCCATTGCGGAAGATTCCCTACTGCTGCCTCCCGTAGGAGTCTGGGCCGTGTCTCAGTCCCAGT
GTGGCCGATCACCCTCTCAGGTCGGCTATGCATCGTTGCCTTGGTGAGCCGTTACCTCACCAACTAGCTAATGCACCGCGGGCCCATCTGTAAGTGATAG
CCGAAACCATCTTTCAATCATCTCCCATGAAGGAGAAGATCCTATCCGGTATTAGCTTCGGTTTCCCGAAGTTATCCCAGTCTTACAGGCAGGTTGCCCA
CGTGTTACTCACCCGTCCGCCGTCAAAGT

>EB191
AGAGTTTGATTATGGCTCAGGAGATCGTACGTGGTCAACCTGCCTGTTGACTGGGATAACTTCGGGAAACCGAAGCTAATACCGGATAGGATCTTCTCCT
TCATGGGAGATGATTGAAAGATGGTTTCGGCTATCACTTACAGATGGGCCCGCGGTGCATTAGCTAGTTGGTGAGGTAACGGCTCACCAAGGCAACGATG
CATAGCCGACCTGAGAGGGTGATCGGCCACACTGGGACTGAGACACGGCCCAGAYTCCTACGGGAGGCAGCAGTAGGGAATCTTCCGCAATGGACGAAAG
TCTGACGGAGCAACGCCGCGTGAGTGATGAAGGCTTTCGGGTCGTAAAACTCTGTTGTTAGGGAAGAACAAGTACAAGAGTAACTGCTTGTACCTTGACG
GTACCTAACCAGAAAGCCACGGCTAACTACGTGCCAGCAGCCGCGGTAATACGTAGGTGGCAAGCGTTATCCGGAATTATTGGGCGTAAAGCGCGCGCAG
GCGGTTTCTTAAGTCTGATGTGAAAGCCCACGGCTCAACCGTGGAGGGTCATTGGAAACTGGGGAACTTGAGTGCAGAAGAGAAAAGCGGAATTCCACGT
GTAGCGGTGAAATGCGTAGAGATGTGGAGGAACACCAGTGGCGAAGGCGGCTTTTTGGTCTGTAACTGACGCTGAGGCGCGAAAGCGTGGGGAGCAAACA
GGATTAGATACCCTGGTAGTCCACGCCGTAAACGATGAGTGCTAAGTGTTAGAGGGTTTCCGCCCTTTAGTGCTGCAGCTAACGCATTAAGCACTCCGCC
TGGGGAGTACGGTCGCAAGACTGAAACTCAAAGGAATTGACGGGGGCCCGCACAAGCGGTGGAGCATGTGGTTTAATTCGAAGCAACGCGAAGAACCTTA
CCAGGTCTTGACATCCTCTGACAACTCTAGAGATAGAGCGTTCCCCTTCGGGGGACAGAGTGACAGGTGGTGCATGGTTGTCGTCAGCTCGTGTCGTGAG
ATGTTGGGTTAAGTCCCGCAACGAGCGCAACCCTTGATCTTAGTTGCCAGCATTTAGTTGGGCACTCTAAGGTGACTGCCGGTGACAAACCGGAGGAAGG
TGGGGATGACGTCAAATCATCATGCCCCTTATGACCTGGGCTACACACGTGCTACAATGGATGGTACAAAGGGCTGCAAGACCGCGAGGTCAAGCCAATC
CCATAAAACCATTCTCAGTTCGGATTGTAGGCTGCAACTCGCCTACATGAAGCGGGAATCGTTAGTAATCGCGGATCAGCATGCCGCGGTGAATACGTTC
CCGGGCCTTGTACACACCGCCCGTCACATC

>EB192
ACGTTAGCGGCGGACGGGTGAGTAACACGTGGGCAACCTGCCTGTAAGACTGGGATAACTTCGGGAAACCGAAGCTAATACCGGATAGGATCTTCTCCTT
CATGGGAGATGATTGAAAGATGGTTTCGGCTATCACTTACAGATGGGCCCGCGGTGCATTAGCTAGTTGGTGAGGTAACGGCTCACCAAGGCAACGATGC
ATAGCCGACCTGAGAGGGTGATCGGCCACACTGGGACTGAGACACGGCCCAGACTCCTACGGGAGGCAGCAGTAGGGAATCTTCCGCAATGGACGAAAGT
CTGACGGAGCAACGCCGCGTGAGTGATGAAGGCTTTCGGGTCGTAAAACTCTGTTGTTAGGGAAGAACAAGTACAAGAGTAACTGCTTGTACCTTGACGG
TACCTAACCAGAAAGCCACGGCTAACTACGTGCCAGCAGCCGCGGTAATACGTAGGTGGCAAGCGTTATCCGGAATTATTGGGCGTAAAGCGCGCGCAGG
CGGTTTCTTAAGTCTGATGTGAAAGCCCACGGCTCAACCGTGGAGGGTCATTGGAAACTGGGGAACTTGAGTGCAGAAGAGAAAAGCGGAATTCCACGTG
TAGCGGTGAAATGCGTAGAGATGTGGAGGAACACCAGTGGCGAAGGCGGCTTTTTGGTCTGTAACTGACGCTGAGGCGCGAAAGCGTGGGGAGCAAACAG
GATTAGATACCCTGGTAGTCCACGCCGTAAACGATGAGTGCTAAGTGTTAGAGGGTTTCCGCCCTTTAGTGCTGCAGCTAACGCATTAAGCACTCCGCCT
GGGGAGTACGGTCGCAAGACTGAAACTCAAAGGAATTGACGGGGGCCCGCACAAGCGGTGGAGCATGTGGTTTAATTCGAAGCAACGCGAAGAACCTTAC
CAGGTCTTGACATCCTCTGACAACTCTAGAGATAGAGCGTTCCCCTTCGGGGGACAGAGTGACAGGTGGTGCATGGTTGTCGTCAGCTCGTGTCGTGAGA
TGTTGGGTTAAGTCCCGCAACGAGCGCAACCCTTGATCTTAGTTGCCAGCATTTAGTTGGGCACTCTAAGGTGACTGCCGGTGACAAACCGGAGGAAGGT
GGGGATGACGTCAAATCATCATGCCCCTTATGACCTGGGCTACACACGTGCTACAATGGATGGTACAAAGGGCTGCAAGACCGCGAGGTCAAGCCAATCC
CATAAAACCATTCTCAGTTCGGATTGTAGGCTGCAACTCGCCTACATGAAGCTGGAATCGTTAGTAATCGCGGATCAGCATGCCGCGGTGAATACGTTCC
CGTACCTTGTACACACCGCCCGTCACACCACGAGAGTTTGTAACATAGAG

>EB193
TTTTTTTTAGCTCAGGATGAACGCTGGCGGCGTGCTTAACACATGCAAGTCGAACGGTGAAGCAGAGCTTGCTCTGTGGATCAGTGGCGAACGGGTGAGT
AACACGTGAGCAACCTGCCCCTGACTCTGGGATAAGCGCTGGAAACGGCGTCTAATACTGGATATGAGCTGCGATCGCATGGTCAGCAGTTGGAAAGATT
TTTCGGTCTGGGATGGGCTCACGGCCTATCAGCTTGTTGGTGAGGTAATGGCTCACCAAGGCGTCGACGGGTAGCCGGCCTGAGAGGGTGACCGGCCACA
CTGGGACTGAGACACGGCCCAGACTCCTACGGGAGGCAGCAGTGGGGAATATTGCACAATGGGCGAAAGCCTGATGCAGCAACGCCGCGTGAGGGATGAC
GGCCTTCGGGTTGTAAACCTCTTTTAGCAGGGAAGAAGCGAAAGTGACGGTACCTGCAGAAAAAGCGCCGGCTAACTACGTGCCAGCAGCCGCGGTAATA
CGTAGGGCGCAAGCGTTATCCGGAATTATTGGGCGTAAAGAGCTCGTAGGCGGTTTGTCGCGTCTGCTGTGAAATCCCGAGGCTCAACCTCGGGTCTGCA
GTGGGTACGGGCAGACTAGAGTGCGGTAGGGGAGATTGGAATTCCTGGTGTAGCGGTGGAATGCGCAGATATCAGGAGGAACACCGATGGCGAAGGCAGA
TCTCTGGGCCGTTACTGACGCTGAGGAGCGAAAGGGTGGGGAGCAAACAGGCTTAGATACCCTGGTAGTCCACCCCGTAAACGTTGGGAACTAGTTGTGG
GGTCCATTCCACGGATTCCGTGACGCAGCTAACGCATTAAGTTCCCCGCCTGGGGAGTACGGCCGCAAGGCTAAAACTCAAAGGAATTGACGGGGACCCG
CACAAGCGGCGGAGCATGCGGATTAATTCGATGCAACGCGAAGAACCTTACCAAGGCTTGACATATACGAGAACGGGCCAGAAATGGTCAACTCTTTGGA
CACTCGTAAACAGGTGGTGCATGGTTGTCGTCAGCTCGTGTCGTGAGATGTTGGGTTAAGTCCCGCAACGAGCGCAACCCTCGTTCTATGTTGCCAGCAC
GTAATGGTGGGAACTCATGGGATACTGCCGGGGTCAACTCGGAGGAAGGTGGGGATGACGTCAAATCATCATGCCCCTTATGTCTTGGGCTTCACGCATG
CTACAATGGCCGGTACAAAGGGCTGCAATACCGCGAGGTGGAGCGAATCCCAAAAAGCCGGTCCCAGTTCGGATTGAGGTCTGCAACTCGACCTCATGAA
GTCGGAGTCGCTAGTAATCGCAGATCAGCAACGCTGCGGTGAATACGTTCCCGGGTCTTGTACACACCGCCCGTCAAGTCATGAAAGTCGGTAACACCTG
AAGCCGGTGGCCCAACCCTTGTGGAGGGAGCCGTCGAAGGTGGGATCGGTATTAGGATAATCGAC

>EB194
CGGTGAACACGGAGCTTGCTCTGTGGGATCAGTGGCGAACGGGTGAGTAACACGTGAGCAACCTACCCCTGACTCTGGGATAAGCGCTGGAAACGGCGTC
TAATACTGGATACGAGTGGCGACCGCATGGTCAGCTACTGGAAAGATTTATTGGTTGGGGATGGGCTCGCGGCCTATCAGCTTGTTGGTGAGGTAATGGC
TCACCAAGGCGTCGACGGGTAGCCGGCCTGAGAGGGTGACCGGCCACACTGGGACTGAGACACGGCCCAGACTCCTACGGGAGGCAGCAGTGGGGAATAT
TGCACAATGGGCGCAAGCCTGATGCAGCAACGCCGCGTGAGGGATGACGGCCTTCGGGTTGTAAACCTCTTTTAGCAGGGAAGAAGCGAAAGTGACGGTA
CCTGCAGAAAAAGCGCCGGCTAACTACGTGCCAGCAGCCGCGGTAATACGTAGGGCGCAAGCGTTATCCGGAATTATTGGGCGTAAAGAGCTCGTAGGCG
GTTTGTCGCGTCTGCTGTGAAATCCGGAGGCTCAACCTCCGGGCCTGCAGTGGGTACGGGCAGACTAGAGTGCGGTAGGGGAGATTGGAATTCCTGGTGT
AGCGGTGGAATGCGCAGATATCAGGAGGAACACCGATGGCGAAGGCAGATCTCTGGGCCGTAACTGACGCTGAGGAGCGAAAGGGTGGGGAGCAAACAGG
CTTAGATACCCTGGTAGTCCACCCCGTAAACGTTGGGAACTAGTTGTGGGGTCCATTCCACGGATTCCGTGACGCAGCTAACGCATTAAGTTCCCCGCCT
GGGGAGTACGGCCGCAAGGCTAAAACTCAAAGGAATTGACGGGGACCCGCACAAGCGGCGGAGCATGCGGATTAATTCGATGCAACGCGAAGAACCTTAC
CAAGGCTTGACATATACGAGAACGGGCCAGAAATGGTCAACTCTTTGGACACTCGTAAACAGGTGGTGCATGGTTGTCGTCAGCTCGTGTCGTGAGATGT
TGGGTTAAGTCCCGCAACGAGCGCAACCCTCGTTCTATGTTGCCAGCACGTAATGGTGGGAACTCATGGGATACTGCCGGGGTCAACTCGGAGGAAGGTG
GGGATGACGTCAAATCATCATGCCCCTTATGTCTTGGGCTTCACGCATGCTACAATGGCCGGTACAAAGGGCTGCAATACCGCGAGGTGGAGCGAATCCC
AAAAAGCCGGTCCCAGTTCGGATTGAGGTCTGCAACTCGACCTCATGAAGTCGGAGTCGCTAGTAATCGCAGATCAGCAACGCTGCGGTGAATACGTTCC
CGGGTCTTGTACACACCGCCCGTCAAGTCATGAAAGTCGGTAACACCTGAAGCCGGTGGCCTAACCC

>EB195

CCCGTGAATAGTTATGCAGGCGGACGGGTGAGTAACACGTGGGTAACCTGCCTGTAAGACTGGGATAACTCCGGGAAACCGGGGCTAATACCGGATGGTT
GTTTGAACCGCATGGTTCAAACATAAAAGGTGGCTTCGGCTACCACTTACAGATGGACCCGCGGCGCATTAGCTAGTTGGTGAGGTAACGGCTCACCAAG
GCAACGATGCGTAGCCGACCTGAGAGGGTGATCGGCCACACTGGGACTGAGACACGGCCCAGACTCCTACGGGAGGCAGCAGTAGGGAATCTTCCGCAAT
GGACGAAAGTCTGACGGAGCAACGCCGCGTGAGTGATGAAGGTTTTCGGATCGTAAAGCTCTGTTGTTAGGGAAGAACAAGTACCGTTCGAATAGGGCGG
TACCTTGACGGTACCTAACCAGAAAGCCACGGCTAACTACGTGCCAGCAGCCGCGGTAATACGTAGGTGGCAAGCGTTGTCCGGAATTATTGGGCGTAAA
GGGCTCGCAGGCGGTTTCTTAAGTCTGATGTGAAAGCCCCCGGCTCAACCGGGGAGGGTCATTGGAAACTGGGGAACTTGAGTGCAGAARAGGAGAGTGG
AATTCCACGTGTAGCGGTGAAATGCGTAKAGATGTGGAGGAACACCAGTGGCGAAGGCGACTCTCTGGTCTGTAACTGACGCTGAGGAGCGAAAGCGTGG
GGAGCGAACAGGATTAGATACCCTGGTAGTCCACGCCGTAAACGATGAGTGCTAAGTGTTAGGGGGTTTCCGCCCCTTAGTGCTGCAGCTAACGCATTAA
GCACTCCGCCTGGGGAGTACGGTCGCAAGACTGAAACTCAAAGGAATTGACGGGGGCCCGCACAAGCGGTGGAGCATGTGGTTTAATTCGAAGCAACGCG
AAGAACCTTACCAGGTCTTGACATCCTCTGACAATCCTAGAGATAGGACGTCCCCTTCGGGGGCAGAGTGACAGGTGGTGCATGGTTGTCGTCAGCTCGT
GTCGTGAGATGTTGGGTTAAGTCCCGCAACGAGCGCAACCCTTGATCTTAGTTGCCAGCATTCAGTTGGGCACTCTAAGGTGACTGCCGGTGACAAACCG
GAGGAAGGTGGGGATGACGTCAAATCATCATGCCCCTTATGACCTGGGCTACACACGTGCTACAATGGACAGAACAAAGGGCAGCGAAACCGCGAGGTTA
AGCCAATCCCACAAATCTGTTCTCAGTTCGGATCGCAGTCTGCAACTCGACTGCGTGAAGCTGGAATCGCTAGTAATCGCGGATCAGCATGCCGCGGTGA
ATACGTTCCCGGGCCTTGTACACACCGCCCGTCACACCACGAGAGTTTGTAACACCCGATGTCGGTGAGGTAACCTATTTGGAGCCAGCC

>EB196
GGCTCCCTCCACAAGGGTTAGGCCACCGGCTTCAGGTGTTACCGACTTTCATGACTTGACGGGCGGTGTGTACAAGACCCGGGAACGTATTCACCGCAGC
GTTGCTGATCTGCGATTACTAGCGACTCCGACTTCATGAGGTCGAGTTGCAGACCTCAATCCGAACTGGGACCGGCTTTTTGGGATTCGCTCCACCTCGC
GGTATTGCAGCCCTTTGTACCGGCCATTGTAGCATGCGTGAAGCCCAAGACATAAGGGGCATGATGATTTGACGTCATCCCCACCTTCCTCCGAGTTGAC
CCCGGCAGTATCCCATGAGTTCCCACCATTACGTGCTGGCAACATAGAACGAGGGTTGCGCTCGTTGCGGGACTTAACCCAACATCTCACGACACGAGCT
GACGACAAACCATGCACCACCCTGTTTACGAGTGTCCAAAGAGTTGACCATTTCTGGCCCGTTCTCGTATATGTCAAGCCTTGGTAAGGTTCTTCGCGTT
GCATCGAATTAATCCGCATGCTCCGCCGCTTGTGCGGGTCCCCGTCAATTCCTTTGAGTTTTAGCCTTGCGGCCGTACTCCCCAGGCGGGGAACTTAATG
CGTTAGCTGCGTCACGGAATCCGTGGAATGGACCCCACAACTAGTTCCCAACGTTTACGGGGTGGACTACCAGGGTATCTAAGCCTGTTTGCTCCCCACC
CTTTCGCTCCTCAGCGTCAGTTACGGCCCAGAGATCTGCCTTCGCCATCGGTGTTCCTCCTGATATCTGCGCATTCCACCGCTACACCAGGAATTCCAAT
CTCCCCTACCGCACTCTAGTCTGCCCGTACCCACTGCAGGCCGGAGGTTGAGCCCTCCGGATTTTCACAGCAGACGCGACAAACCGCCTACGAGCTCTTT
TACGCCCCAATAATTCCGGATAACGCTTGCGCCCTACGTATTACCGGCGGCTGCTGGGCACGTAGTTAGCCGGCGCTTTTTTCTGCAGGTACCGTCACTT
TTCGCTTCTTCCCTGCTAAAAGAGGTTTACAACCCGAAGGCCGTCATCCCTCACGCGGCGTTGCTGCATCAGGCTTTCGCCCATTGTGCAATATTCCCCA
CTGCTGCCTCCCGTAGGAGTCTGGGCCGTGTCTCAGTCCCAGTGTGGCCGGTCACCCTCTCAGGCCGGCTACCCGTCGACGCCTTGGTGAGCCATTACCT
CACCAACAAGCTGATAGGCCGCGAGCCCATCCCCAACCGAAATTCTTTCCAACCACTGAAGATGCCTTCGTGGTTCGTATCCAGTATTAGACGCCGTTTC
CAGCGCTTATCCCAGAGTCAGGGGCAGGTTGCTCACGTGTTACTCACCCGTTCGCCACTGATCCCACAGAGCAAGCTCCGTGTTCACCGTTCGACTT

>EB197
GGCTCCCTCCACAAGGGTTGGGCCACCGGCTTCAGGTGTTACCGACTTTCATGACTTGACGGGCGGTGTGTACAAGACCCGGGAACGTATTCACCGCAGC
GTTGCTGATCTGCGATTACTAGCGACTCCGACTTCATGAGGTCGAGTTGCAGACCTCAATCCGAACTGGGACCGGCTTTTTGGGATTCGCTCCACCTCAC
GGTATTGCAGCCCTTTGTACCGGCCATTGTAGCATGCGTGAAGCCCAAGACATAAGGGGCATGATGATTTGACGTCATCCCCACCTTCCTCCGAGTTGAC
CCCGGCAGTATCCCATGAGTTCCCACCATTACGTGCTGGCAACATAGAACGAGGGTTGCGCTCGTTGCGGGACTTAACCCAACATCTCACGACACGAGCT
GACGACAACCATGCACCACCTGTTCACCAGTGTCCAAAGAGTTGACCATTTCTGGCCCGNTTCTGGTGTATGTCAAGCCTTGGTAAGGTTCTTCGCGTTG
CATCGAATTAATCCGCATGCTCCGCCGCTTGTGCGGGTCCCCGTCAATTCCTTTGAGTTTTAGCCTTGCGGCCGTACTCCCCAGGCGGGGAACTTAATGC
GTTAGCTGCGTCACGGAAACCGTGGAATGGTCCCCACAACTAGTTCCCAACGTTTACGGGGTGGACTACCAGGGTATCTAAGCCTGTTTGCTCCCCACCC
TTTCGCTCCTCAGCGTCAGTTACGGCCCAGAGATCTGCCTTCGCCATCGGTGTTCCTCCTGATATCTGCGCATTCCACCGCTACACCAGGAATTCCAATC
TCCCCTACCGCACTCTAGTCTGCCCGTACCCACTGCAGGCCCGAGGTTGAGCCTCGGGATTTCACAGCAGACGCGACAAACCGCCTACGAGCTCTTTACG
CCCAATAATTCCGGATAACGCTTGCGCCCCTACGTATTACCGCGGCTGCTGGCACGTAGTTAGCCGGCGCTTTTTCTGCAGGTACCGTCACTTTCGCTTC
TTCCCTGCTAAAAGAGGTTTACAACCCGAAGGCCGTCATCCCTCACGCGGCGTTGCTGCATCAGGCTTTCGCCCATTGTGCAATATTCCCCACTGCTGCC
TCCCGTAGGAGTCTGGGCCGTGTCTCAGTCCCAGTGTGGCCGGTCACCCTCTCAGGCCGGCTACCCGTCGACGCCTTGGTGAGCCATTACCTCACCAACA
AGCTGATAGGCCGCGAGCCCATCCCAGACCAAAAAATCTTTCCAACCCCCACCATGCGGAAGAGGCTCATATCCAGTATTAGACGCCGTTTCCAGCGCTT
ATCCCAGAGTCCAGGGCAGGTTGCTCACGTGTTACTCACCCGTTCGCCACTGATCCACCAAGCAAGCTTGGCTTCACCGTTCGAC

>EB198
CCACAAGGGTTGGGCCAACCGGCTTCAGGTGTTACCGACTTTCATGACTTGACGGGCGGTGTGTACAAGACCCGGGAACGTATTCACCGCAGCGTTGCTG
ATCTGCGATTACTAGCGACTCCGACTTCATGAGGTCGAGTTGCAGACCTCAATCCGAACTGGGACCGGCTTTTTGGGATTCGCTCCACCTCACGGTATTG
CAGCCCTTTGTACCGGCCATTGTAGCATGCGTGAAGCCCAAGACATAAGGGGCATGATGATTTGACGTCATCCCCACCTTCCTCCGAGTTGACCCCGGCA
GTATCCCATGAGTTCCCACCATTACGTGCTGGCAACATAGAACGAGGGTTGCGCTCGTTGCGGGACTTAACCCAACATCTCACGACACGAGCTGACGACA
ACCATGCACCACCTGTATAGAGACCTTGCGGGGCGACTGTTTCCAGCCGTTTCCTCTATATGTCAAGCCTTGGTAAGGTTCTTCGCGTTGCATCGAATTA
ATCCGCATGCTCCGCCGCTTGTGCGGGTCCCCGTCAATTCCTTTGAGTTTTAGCCTTGCGGCCGTACTCCCCAGGCGGGGAACTTAATGCGTTAGCTGCG
TCACGGAATCCGTGGAATGGACCCCACAACTAGTTCCCAACGTTTACGGGGTGGACTACCAGGGTATCTAAGCCTGTTTGCTCCCCACCCTTTCGCTCCT
CAGCGTCAGTTACGGCCCAGAGATCTGCCTTCGCCATCGGTGTTCCTCCTGATATCTGCGCATTCCACCGCTACACCAGGAATTCCAATCTCCCCTACCG
CACTCTAGTCTGCCCGTACCCACTGCAGGCCCGAGGTTGAGCCTCGGGTTTTCACAGCAGACGCGACAGACCGCCTACGAGCTCTTTACGCCCAATAATT
CCGGATAACGCTTGCGCCCTACGTATTACCGCGGCTGCTGGCACGTAGTTAGCCGGCGCTTTTTCTGCAGGTACCGTCACTTTCGCTTCTTCCCTGCTAA
AAGAGGTTTACAACCCGAAGGCCGTCATCCCTCACGCGGCGTTGCTGCATCAGGCTTCCGCCCATTGTGCAATATTCCCCACTGCTGCCTCCCGTAGGAG
TCTGGGCCGTGTCTCAGTCCCAGTGTGGCCGGTCACCCTCTCAGGCCGGCTACCCGTCGACGCCTTGGTGAGCCATTACCTCACCAACAAGCTGATAGGC
CGCGAGCTCATCCCTGACCGAAATTCTTTCCAGCTACTGACCATGCGATCGCAGCTCGTATCCGGTATTAGACGCCGTTTCCAGCGCTTATCCCAGAGTC
AGGGGCAGATTGCTCACGTGTTACTCACCCGTTCGCCACTGATCCACAGAGCAAGCTCTGCTTCACCGTTCGACTTGCA

>EB199
GAGTTCGCGTGGGGTACCTGCCTATAAGACTGGGATAACTTCGGGAAACCGGAGCTAATACCGGATACGTTCTTTTCTCGCATGAGAGAAGATGGAAAGA
CGGTTTACGCTGTCACTTATAGATGGGCCCGCGGCGCATTAGCTAGTTGGTGAGGTAATGGCTCACCAAGGCGACGATGCGTAGCCGACCTGAGAGGGTG
ATCGGCCACACTGGGACTGAGACACGGCCCAGACTCCTACGGGAGGCAGCAGTAGGGAATCTTCCGCAATGGACGAAAGTCTGACGGAGCAACGCCGCGT
GAACGAAGAAGGCCTTCGGGTCGTAAAGTTCTGTTGTTAGGGAAGAACAAGTACCAGAGTAACTGCTGGTACCTTGACGGTACCTAACCAGAAAGCCACG
GCTAACTACGTGCCAGCAGCCGCGGTAATACGTAGGTGGCAAGCGTTGTCCGGAATTATTGGGCGTAAAGCGCGCGCAGGTGGTTCCTTAAGTCTGATGT
GAAAGCCCACGGCTCAACCGTGGAGGGTCATTGGAAACTGGGGAACTTGAGTGCAGAAGAGGAAAGTGGAATTCCAAGTGTAGCGGTGAAATGCGTAGAG
ATTTGGAGGAACACCAGTGGCGAAGGCGACTTTCTGGTCTGTAACTGACACTGAGGCGCGAAAGCGTGGGGAGCAAACAGGATTAGATACCCTGGTAGTC
CACGCCGTAAACGATGAGTGCTAAGTGTTAGAGGGTTTCCGCCCTTTAGTGCTGCAGCTAACGCATTAAGCACTCCGCCTGGGGAGTACGGCCGCAAGGC
TGAAACTCAAAGGAATTGACGGGGGCCCGCACAAGCGGTGGAGCATGTGGTTTAATTCGAAGCAACGCGAAGAACCTTACCAGGTCTTGACATCCTCTGA
CAACCCTAGAGATAGGGCTTTCCCCTTCGGGGGACAGAGTGACAGGTGGTGCATGGTTGTCGTCAGCTCGTGTCGTGAGATGTTGGGTTAAGTCCCGCAA
CGAGCGCAACCCTTGATCTTAGTTGCCAGCATTCAGTTGGGCACTCTAAGGTGACTGCCGGTGACAAACCGGAGGAAGGTGGGGATGACGTCAAATCATC
ATGCCCCTTATGACCTGGGCTACACACGTGCTACAATGGATGGTACAAAGGGCTGCAAACCTGCGAAGGTAAGCGAATCCCATAAAGCCATTCTCAGTTC
GGATTGCAGGCTGCAACTCGCCTGCATGAAGCCGGAATCGGTAGTAATCGCGGATCAGCATGCCGCGGGGAACACGTTCCCGGTCGAGGTACACCCCGGC
TCTCTCAGCCGTAGCAAGGTAACCGTAGAGTTTGATGTGTGGCTCAGTAAGTCGTAACAAGGT

>EB200
TGCAGTCGAGCGGACAGAAGGGAGCTTGCTCCCGGATGTTAGCGGCGGACGGGTGAGTAACACGTGGGTAACCTGCCTGTAAGACTGGGATAACTCCGGG
AAACCGGAGCTAATACCGGATAGTTCCTTGAACCGCATGGTTCAAGGATGAAAGACGGTTTCGGCTGTCACTTACAGATGGACCCGCGGCGCATTAGCTA
GTTGGTGGGGTAATGGCTCACCAAGGCGACGATGCGTAGCCGACCTGAGAGGGTGATCGGCCACACTGGGACTGAGACACGGCCCAGACTCCTACGGGAG
GCAGCAGTAGGGAATCTTCCGCAATGGACGAAAGTCTGACGGAGCAACGCCGCGTGAGTGATGAAGGTTTTCGGATCGTAAAGCTCTGTTGTTAGGGAAG
AACAAGTGCGAGAGTAACTGCTCGCACCTTGACGGTACCTAACCAGAAAGCCACGGCTAACTACGTGCCAGCAGCCGCGGTAATACGTAGGTGGCAAGCG
TTGTCCGGAATTATTGGGCGTAAAGGGCTCGCAGGCGGTTTCTTAAGTCTGATGTGAAAGCCCCCGGCTCAACCGGGGAGGGTCATTGGAAACTGGGAAA
CTTGAGTGCAGAAGAGGAGAGTGGAATTCCACGTGTAGCGGTGAAATGCGTAGAGATGTGGAGGAACACCAGTGGCGAAGGCGACTCTCTGGTCTGTAAC
TGACGCTGAGGAGCGAAAGCGTGGGGAGCGAACAGGATTAGATACCCTGGTAGTCCACGCCGTAAACGATGAGTGCTAAGTGTTAGGGGGTTTCCGCCCC
TTAGTGCTGCAGCTAACGCATTAAGCACTCCGCCTGGGGAGTACGGTCGCAAGACTGAAACTCAAAGGAATTGACGGGGGCCCGCACAAGCGGTGGAGCA
TGTGGTTTAATTCGAAGCAACGCGAAGAACCTTACCAGGTCTTGACATCCTCTGACAACCCTAGAGATAGGGCTTTCCCTTCGGGGACAGAGTGACAGGT
GGTGCATGGTTGTCGTCAGCTCGTGTCGTGAGATGTTGGGTTAAGTCCCGCAACGAGCGCAACCCTTGATCTTAGTTGCCAGCATTCAGTTGGGCACTCT
AAGGTGACTGCCGGTGACAAACCGGAGGAAGGTGGGGATGACGTCAAATCATCATGCCCCTTATGACCTGGGCTACACACGTGCTACAATGGACAGAACA
AAGGGCTGCAAGACCGCAAGGTTTAGCCAATCCCATAAATCTGTTCTCAGTTCGGATCGCAGTCTGCAACTCGACTGCGTGAAGCTGGAATCGCTAGTAA
TCGCGGATCAGCATGCCGCGGTGAATACGTTCCCGGGCCTTGTACACACCGCCCGTCACACCACGAGAGTTTGCAACACCCGAAGTCGGTGAGGTAACCT
TTATGGAGCCAGCCG

>EB201
GACTGGGATAACTCCGGGAAACCGGGGCTAATACCGGATAACATTTTGCACCGCATGGTGCGAAATTCAAAGGCGGCTTCGGCTGTCACTTATGGATGGA
CCCGCGTCGCATTAGCTAGTTGGTGAGGTAACGGCTCACCAAGGCAACGATGCGTAGCCGACCTGAGAGGGTGATCGGCCACACTGGGACTGAGACACGG
CCCAGACTCCTACGGGAGGCAGCAGTAGGGAATCTTCCGCAATGGACGAAAGTCTGACGGAGCAACGCCGCGTGAGTGATGAAGGCTTTCGGGTCGTAAA
ACTCTGTTGTTAGGGAAGAACAAGTGCTAGTTGAATAAGCTGGCACCTTGACGGTACCTAACCAGAAAGCCACGGCTAACTACGTGCCAGCAGCCGCGGT
AATACGTAGGTGGCAAGCGTTATCCGGAATTATTGGGCGTAAAGCGCGCGCAGGTGGTTTCTTAAGTCTGATGTGAAAGCCCACGGCTCAACCGTGGAGG
GTCATTGGAAACTGGGAGACTTGAGTGCAGAAGAGGAAAGTGGAATTCCATGTGTAGCGGTGAAATGCGTAGAGATATGGAGGAACACCAGTGGCGAAGG
CGACTTTCTGGTCTGTAACTGACACTGAGGCGCGAAAGCGTGGGGAGCAAACAGGATTAGATACCCTGGTAGTCCACGCCGTAAACGATGAGTGCTAAGT
GTTAGAGGGTTTNCCGCCCNTTTAGTGCTGAAGTTAACGCATTAAGCACTTCCGCCNTGGGGAGTACGGCCGCAAGGCTGAAACTCAAAGGAATTGACGG
GGGCCCGCACAAGCGGTGGAGCATGTGGTTTAATTCGAAGCAACGCGAAGAACCTTACCAGGTCTTGACATCCTCTGACAACCCTAGAGATAGGGCTTCC
CCTTCGGGGGCAGAGTGACAGGTGGTGCATGGTTGTCGTCAGCTCGTGTCGTGAGATGTTGGGTTAAGTCCCGCAACGAGCGCAACCCTTGATCTTAGTT
GCCATCATTAAGTTGGGCACTCTAAGGTGACTGCCGGTGACAAACCGGAGGAAGGTGGGGATGACGTCAAATCATCATGCCCCTTATGACCTGGGCTACA
CACGTGCTACAATGGACGGTACAAAGAGCTGCAAGACCGCGAGGTGGAGCTAATCTCATAAAACCGTTCTCAGTTCGGATTGTAGGCTGCAACTCGCCTA
CATGAAGCAGGAATCGCTAGTAATCGCGGATCAGCATGCCGCGGTGAATACGTTCCCGGTCCTTGTACACACCGCCCGTCTCACC

>EB202
CTGCAGTCGAGCGGACAGAAGGGAGCTTGCTCCCGGATGTTAGCGGCGGACGGGTGAGTAACACGTGGGTAACCTGCCTGTAAGACTGGGATAACTCCGG
GAAACCGGAGCTAATACCGGATAGTTCCTTGAACCGCATGGTTCAAGGATGAAAGACGGTTTCGGCTGTCACTTACAGATGGACCCGCGGCGCATTAGCT
AGTTGGTGAGGTAACGGCTCACCAAGGCGACGATGCGTAGCCGACCTGAGAGGGTGATCGGCCACACTGGGACTGAGACACGGCCCAGACTCCTACGGGA
GGCAGCAGTAGGGAATCTTCCGCAATGGACGAAAGTCTGACGGAGCAACGCCGCGTGAGTGATGAAGGTTTTCGGATCGTAAAGCTCTGTTGTTAGGGAA
GAACAAGTGCAAGAGTAACTGCTTGCACCTTGACGGTACCTAACCAGAAAGCCACGGCTAACTACGTGCCAGCAGCCGCGGTAATACGTAGGTGGCAAGC
GTTGTCCGGAATTATTGGGCGTAAAGGGCTCGCAGGCGGTTTCTTAAGTCTGATGTGAAAGCCCCCGGCTCAACCGGGGAGGGTCATTGGAAACTGGGAA
ACTTGAGTGCAGAAGAGGAGAGTGGAATTCCACGTGTAGCGGTGAAATGCGTAGAGATGTGGAGGAACACCAGTGGCGAAGGCGACTCTCTGGTCTGTAA
CTGACGCTGAGGAGCGAAAGCGTGGGGAGCGAACAGGATTAGATACCCTGGTAGTCCACGCCGTAAACGATGAGTGCTAAGTGTTAGGGGGTTTCCGCCC
CTTAGTGCTGCAGCTAACGCATTAAGCACTCCGCCTGGGGAGTACGGTCGCAAGACTGAAACTCAAAGGAATTGACGGGGGCCCGCACAAGCGGTGGAGC
ATGTGGTTTAATTCGAAGCAACGCGAAGAACCTTACCAGGTCTTGACATCCTCTGACAACCCTAGAGATAGGGCTTTCCCTTCGGGGACAGAGTGACAGG
TGGTGCATGGTTGTCGTCAGCTCGTGTCGTGAGATGTTGGGTTAAGTCCCGCAACGAGCGCAACCCTTGATCTTAGTTGCCAGCATTCAGTTGGGCACTC
TAAGGTGACTGCCGGTGACAAACCGGAGGAAGGTGGGGATGACGTCAAATCATCATGCCCCTTATGACCTGGGCTACACACGTGCTACAATGGACAGAAC
AAAGGGCTGCGAGACCGCAAGGTTTAGCCAATCCCACAAATCTGTTCTCAGTTCGGATCGCAGTCTGCAACTCGACTGCGTGAAGCTGGAATCGCTAGTA
ATCGCGGATCAGCATGCCGCGGTGAATACGTTCCCGGGCCTTGTACACACCGCCCGTCACACCACGAGAGTTTGCAACACCCGAAGTCGGTGAGGTAACC
TTTATGGAGCCAGCCGCCGAAGGTGGGGCAGATGATTGGGGTGAAGTCGTAACAA

>EB203
CGACGGCTCCCTCCACAAGGGTTAGGCCACCGGCTTCAGGTGTTACCGACTTTCATGACTTGACGGGCGGTGTGTACAAGACCCGGGAACGTATTCACCG
CAGCGTTGCTGATCTGCGATTACTAGCGACTCCGACTTCATGAGGTCGAGTTGCAGACCTCAATCCGAACTGGGACCGGCTTTTTGGGATTCGCTCCACC
TCACGGTATTGCAGCCCTTTGTACCGGCCATTGTAGCATGCGTGAAGCCCAAGACATAAGGGGCATGATGATTTGACGTCATCCCCACCTTCCTCCGAGT
TGACCCCGGCAGTATCCCATGAGTTCCCACCATTACGTGCTGGCAACATAGAACGAGGGTTGCGCTCGTTGCGGGACTTAACCCAACATCTCACGACACG
AGCTGACGACAACCATGCACCACCTGTTTACGAGTGTCCAAAGAGTTGACCCATTTCTGGCCCGTTCTCGTATATGTCAAGCCTTGGTAAGGTTCTTCGC
GTTGCATCGAATTAATCCGCATGCTCCGCCGCTTGTGCGGGTCCCCCGTCAATTCCTTTGAGTTTTAGCCTTGCGGCCGTACTCCCCAGGCGGGGAACTT
AATGCGTTAGCTGCGTCACGGAATCCGTGGAATGGACCCCACAACTAGTTCCCAACGTTTACGGGGTGGACTACCAGGGTATCTAAGCCTGTTTGCTCCC
CACCCTTTCGCTCCTCAGCGTCAGTTACGGCCCAGAGATCTGCCTTCGCCATCGGTGTTCCTCCTGATATCTGCGCATTCCACCGCTACACCAGGAATTC
CAATCTCCCCTACCGCACTCTAGTCTGCCCGTACCCACTGCAGGCCCGAGGTTGAGCCTCGGGATTTCACAGCAGACGCGACAAACCGCCTACGAGCTCT
TTACGCCCAATAATTCCGGATAACGCTTGCGCCCTACGTATTACCGCGGCTGCTGGCACGTAGTTAGCCGGCGCTTTTTCTGCAGGTACCGTCACTTTCG
CTTCTTCCCTGCTAAAAGAGGTTTACAACCCGAAGGCCGTCATCCCTCACGCGGCGTTGCTGCATCAGGCTTTCGCCCATTGTGCAATATTCCCCACTGC
TGCCTCCCGTAGGAGTCTGGGCCGTGTCTCAGTCCCAGTGTGGCCGGTCACCCTCTCAGGCCGGCTACCCGTCGACGCCTTGGTGAGCCATTACCTCACC
AACAAGCTGATAGGCCGCGAGCCCATCCCCAACCGAAAAATCTTTCCAAACGCAGACCATGCGGTCACGTCACATATCCAGTATTAGACGCCGTTTCCAG
CGCTTATCCCAGAGTCAGGGGCAGGTTGCTCACGTGTTACTCACCCGTTCGCCACTGATCCCACAGAGCAAGCTCCGTGTTCACCGTTCGAC

>EB204
TGCAAGTCGAACGGTGAACACGGAGCTTGCTCTGTGGGATCAGTGGCGAACGGGTGAGTAACACGTGAGCAACCTGCCCCTGACTCTGGGATAAGCGCTG
GAAACGGCGTCTAATACTGGATATGTGACGTGACCGCATGGTCTGCGTTTGGAAAGATTTTTCGGTTGGGGATGGGCTCGCGGCCTATCAGCTTGTTGGT
GAGGTAATGGCTCACCAAGGCGTCGACGGGTAGCCGGCCTGAGAGGGTGACCGGCCACACTGGGACTGAGACACGGCCCAGACTCCTACGGGAGGCAGCA
GTGGGGAATATTGCACAATGGGCGAAAGCCTGATGCAGCAACGCCGCGTGAGGGATGACGGCCTTCGGGTTGTAAACCTCTTTTAGCAGGGAAGAAGCGA
AAGTGACGGTACCTGCAGAAAAAGCGCCGGCTAACTACGTGCCAGCAGCCGCGGTAATACGTAGGGCGCAAGCGTTATCCGGAATTATTGGGCGTAAAGA
GCTCGTAGGCGGTTTGTCGCGTCTGCTGTGAAATCCCGAGGCTCAACCTCGGGCCTGCAGTGGGTACGGGCAGACTAGAGTGCGGTAGGGGAGATTGGAA
TTCCTGGTGTAGCGGTGGAATGCGCAGATATCAGGAGGAACACCGATGGCGAAGGCAGATCTCTGGGCCGTAACTGACGCTGAGGAGCGAAAGGGTGGGG
AGCAAACAGGCTTAGATACCCTGGTAGTCCACCCCGTAAACGTTGGGAACTAGTTGTGGGGTCCATTCCACGGATTCCGTGACGCAGCTAACGCATTAAG
TTCCCCGCCTGGGGAGTACGGCCGCAAGGCTAAAACTCAAAGGAATTGACGGGGACCCGCACAAGCGGCGGAGCATGCGGATTAATTCGATGCAACGCGA
AGAACCTTACCAAGGCTTGACATATACGAGAACGGGCCAGAAATGGTCAACTCTTTGGACACTCGTAAACAGGTGGGTGCATGGTTGTCGTCAGCTCGTG
TCGTGAGATGTTGGGTTAAGTCCCGCAACGAGCGCAACCCTCGTTCTATGTTGCCAGCACGTAATGGTGGGAACTCATGGGATACTGCCGGGGTCAACTC

GGAGGAAGGTGGGGATGACGTCAAATCATCATGCCCCTTATGTCTTGGGCTTCACGCATGCTACAATGGCCGGTACAAAGGGCTGCAATACCGTGAGGTG
GAGCGAATCCCAAAAAGCCGGTCCCAGTTCGGATTGAGGTCTGCAACTCGACCTCATGAAGTCGGAGTCGCTAGTAATCGCAGATCAGCAACGCTGCGGT
GAATACGTTCCCGGGTCTTGTACACACCGCCCGTCAAGTCATGAAAGTCGGTAACACCTGAAGCCGGTGGCCTAACCCTTGTGGAGGGAG

>EB205
CAAGGGTTAGGCCACCGGCTTCAGGTGTTACCGACTTTCATGACTTGACGGGCGGTGTGTACAAGACCCGGGAACGTATTCACCGCAGCGTTGCTGATCT
GCGATTACTAGCGACTCCGACTTCATGAGGTCGAGTTGCAGACCTCAATCCGAACTGGGACCGGCTTTTTGGGATTCGCTCCACCTCGCGGTATTGCAGC
CCTTTGTACCGGCCATTGTAGCATGCGTGAAGCCCAAGACATAAGGGGCATGATGATTTGACGTCATCCCCACCTTCCTCCGAGTTGACCCCGGCAGTAT
CCCATGAGTTCCCACCATTACGTGCTGGCAACATAGAACGAGGGTTGCGCTCGTTGCGGGACTTAACCCAACATCTCACGACACGAGCTGACGACAACCA
TGCACCACCTGTTTACGAGTGTCCAAAGAGTTGACCATTTTCTGGCCCGTTCTCGTATATGTCAAGCCTTGGTAAGGTTCTTCGCGTTGCATCGAATTAA
TCCGCATGCTCCGCCGCTTGTGCGGGTCCCCGTCAATTCCTTTGAGTTTTTAGCCTTGCGGCCGTACTCCCCAGGCGGGGAACTTAATGCGTTAGCTGCG
TCACGGAATCCGTGGAATGGACCCCACAACTAGTTCCCAACGTTTACGGGGTGGACTACCAGGGTATCTAAGCCTGTTTGCTCCCCACCCTTTCGCTCCT
CAGCGTCAGTTACGGCCCAGAGATCTGCCTTCGCCATCGGTGTTCCTCCTGATATCTGCGCATTCCACCGCTACACCAGGAATTCCAATCTCCCCTACCG
CACTCTAGTCTGCCCGTACCCACTGCAGGCCGGAGGTTGAGCCTCCGGATTTCACAGCAGACGCGACAAACCGCCTACGAGCTCTTTACGCCCAATAATT
CCGGATAACGCTTGCGCCCTACGTATTACCGCGGCTGCTGGCACGTAGTTAGCCGGCGCTTTTTCTGCAGGTACCGTCACTTTCGCTTCTTCCCTGCTAA
AAGAGGTTTACAACCCGAAGGCCGTCATCCCTCACGCGGCGTTGCTGCATCAGGCTTGCGCCCATTGTGCAATATTCCCCACTGCTGCCTCCCGTAGGAG
TCTGGGCCGTGTCTCAGTCCCAGTGTGGCCGGTCACCCTCTCAGGCCGGCTACCCGTCGACGCCTTGGTGAGCCATTACCTCACCAACAAGCTGATAGGC
CGCGAGCCCATCCCCAACCAATAAATCTTTCCAGTAGCTGACCATGCGGTCGCCACTCGTATCCAGTATTAGACGCCGTTTCCAGCGCTTATCCCAGAGT
CAGGGGTAGGTTGCTCACGTGTTACTCACCCGTTCGCCACTGATCCCACAGAGCAAGCTCCGTGTTCACCGTTCGACTGCA

>EB206
TCCACAAGGGTTGGGCCACCGGCTTCAGGTGTTACCGACTTTCATGACTTGACGGGCGGTGTGTACAAGACCCGGGAACGTATTCACCGCAGCGTTGCTG
ATCTGCGATTACTAGCGACTCCGACTTCATGAGGTCGAGTTGCAGACCTCAATCCGAACTGGGACCGGCTTTTTGGGATTCGCTCCACCTCACGGTATTG
CAGCCCTTTGTACCGGCCATTGTAGCATGCGTGAAGCCCAAGACATAAGGGGCATGATGATTTGACGTCATCCCCACCTTCCTCCGAGTTGACCCCGGCA
GTATCCCATGAGTTCCCACCATTACGTGCTGGCAACATAGAACGAGGGTTGCGCTCGTTGCGGGACTTAACCCAACATCTCACGACACGAGCTGACGACA
ACCATGCACCACCTGTATAGAGACCTTGCGGGGCGACTGTTTCCAGACGTTTCCTCTATATGTCAAGCCTTGGTAAGGTTCTTCGCGTTGCATCGAATTA
ATCCGCATGCTCCGCCGCTTGTGCGGGTCCCCGTCAATTCCTTTGAGTTTTAGCCTTGCGGCCGTACTCCCCAGGCGGGGAACTTAATGCGTTAGCTGCG
TCACGGAATCCGTGGAATGGACCCCACAACTAGTTCCCAACGTTTACGGGGTGGACTACCAGGGTATCTAAGCCTGTTTGCTCCCCACCCTTTCGCTCCT
CAGCGTCAGTTACGGCCCAGAGATCTGCCTTCGCCATCGGTGTTCCTCCTGATATCTGCGCATTCCACCGCTACACCAGGAATTCCAATCTCCCCTACCG
CACTCTAGTCTGCCCGTACCCACTGCAGACCCGAGGTTGAGCCTCGGGATTTCACAGCAGACGCGACAGACCGCCTACGAGCTCTTTACGCCCAATAATT
CCGGATAACGCTTGCGCCCTACGTATTACCGCGGCTGCTGGCACGTAGTTAGCCGGCGCTTTTTTCTGCAGGTACCGTCACTTTCGCTTCTTCCCTGCTA
AAAGAGGTTTACAACCCGAAGGCCGTCATCCCTCACGCGGCGTTGCTGCATCAGGCTTCCGCCCATTGTGCAATATTCCCCACTGCTGCCTCCCGTAGGA
GTCTGGGCCGTGTCTCAGTCCCAGTGTGGCCGGTCACCCTCTCAGGCCGGCTACCCGTCGACGCCTTGGTGAGCCATTACCTCACCAACAAGCTGATAGG
CCGCGAGCTCATCCCTGACCGAAAAATCTTTCCAACTGCCGACCATGCGGCCGCAGCTCGTATCCGGTATTAGACGCCGTTTCCAGCGCTTATCCCAGAG
TCAGGGGCAGATTGCTCACGTGTTACTCACCCGTTCGCCACTGATCCACCCAGCAAGCTGGGCTTCACCGTTCGACTGCA

>EB207
CCACAAGGGTTGGGCCACCGGCTTCAGGTGTTACCGACTTTCATGACTTGACGGGCGGTGTGTACAAGACCCGGGAACGTATTCACCGCAGCGTTGCTGA
TCTGCGATTACTAGCGACTCCGACTTCATGAGGTCGAGTTGCAGACCTCAATCCGAACTGGGACCGGCTTTTTGGGATTCGCTCCACCTTGCGGTATTGC
AGCCCTTTGTACCGGCCATTGTAGCATGCGTGAAGCCCAAGACATAAGGGGCATGATGATTTGACGTCATCCCCACCTTCCTCCGAGTTGACCCCGGCAG
TATCCCATGAGTTCCCACCATTACGTGCTGGCAACATAGAACGAGGGTTGCGCTCGTTGCGGGACTTAACCCAACATCTCACGACACGAGCTGACGACAA
ACCATGCACCACCTGTTTACGAGTGTCCAAAGAGTTCTACATTTCTGCAGCGTTCTCGTATATGTCAAGCCTTGGTAAGGTTCTTCGCGTTGCATCGAAT
TAATCCGCATGCTCCGCCGCTTGTGCGGGTCCCCGTCAATTCCTTTGAGTTTTAGCCTTGCGGCCGTACTCCCCCAGGCGGGGAACTTAATGCGTTAGCT
GCGTCACGGAATCCGTGGAATGGACCCCACAACTAGTTCCCAACGTTTACGGGGTGGACTACCAGGGTATCTAAGCCTGTTTGCTCCCCACCCTTTCGCT
CCTCAGCGTCAGTTACGGCCCAGAGATCTGCCTTCGCCATCGGTGTTCCTCCTGATATCTGCGCATTCCACCGCTACACCAGGAATTCCAATCTCCCCTA
CCGCACTCTAGTCTGCCCGTACCCACTGCAGGCCCGAGGTTGAGCCCTCGGGTTTTCACAGCAGACGCGACAAACCGCCTACGAGCTCTTTACGCCCAAT
AATTCCGGATAACGCTTGCGCCCTACGTATTACCGCGGCTGCTGGCACGTAGTTAGCCGGCGCTTTTTCTGCTGGTACGGTCACTTTCACTTCTTCCCTG
CTAAAAGAGGTTTACAACCCGAAGGCCGTCGTCCCTCACGCGGCGTTGCTGCATCAGGCTTGCGCCCATTGTGCAATATTCCCCACTGCTGCCTCCCGTA
GGAGTCTGGGCCGTGTCTCAGTCCCAGTGTGGCCGGTCACCCTCTCAGGCCGGCTACCCGTCGACGCCTTGGTGAGCCATTACCTCACCAACTAGCTGAT
AGGCCGCGAGCTCATCCCTGACCGAAATTCTTTCCAGCTGCTGAAGATGCCTTCGCAGCTCGTATCCGGTATTAGACGCCGTTTCCAGCGCTTATCCCAG
AGTCAGGGGCAGATTGCTCACGTGTTACTCACCCGTTCGCCACTGATCCAGAGAGCAAGCTCTCTTTCACCGTTCGAC

>EB208
CTCCCTCCACAAGGGTTAGGCCACCGGCTTCAGGTGTTACCGACTTTCATGACTTGACGGGCGGTGTGTACAAGACCCGGGAACGTATTCACCGCAGCGT
TGCTGATCTGCGATTACTAGCGACTCCGACTTCATGAGGTCGAGTTGCAGACCTCAATCCGAACTGGGACCGGCTTTTTGGGATTCGCTCCACCTCGCGG
TATTGCAGCCCTTTGTACCGGCCATTGTAGCATGCGTGAAGCCCAAGACATAAGGGGCATGATGATTTGACGTCATCCCCACCTTCCTCCGAGTTGACCC
CGGCAGTATCCCATGAGTTCCCACCATTACGTGCTGGCAACATAGAACGAGGGTTGCGCTCGTTGCGGGACTTAACCCAACATCTCACGACACGAGCTGA
CGACAACCATGCACCACCTGTTTACGAGTGTCCAAAGAGTTGACCATTTCTGGCCCGTTCTCGTATATGTCAAGCCTTGGTAAGGTTCTTCGCGTTGCAT
CGAATTAATCCGCATGCTCCGCCGCTTGTGCGGGTCCCCGTCAATTCCTTTGAGTTTTAGCCTTGCGGCCGTACTCCCCAGGCGGGGAACTTAATGCGTT
AGCTGCGTCACGGAATCCGTGGAATGGACCCCACAACTAGTTCCCAACGTTTACGGGGTGGACTACCAGGGTATCTAAGCCTGTTTGCTCCCCACCCTTT
CGCTCCTCAGCGTCAGTTACGGCCCAGAGATCTGCCTTCGCCATCGGTGTTCCTCCTGATATCTGCGCATTCCACCGCTACACCAGGAATTCCAATCTCC
CCTACCGCACTCTAGTCTGCCCGTACCCACTGCAGGCCGGAGGTTGAGCCTCCGGATTTCACAGCAGACGCGACAAACCGCCTACGAGCTCTTTACGCCC
AATAATTCCGGATAACGCTTGCGCCCTACGTATTACCGCGGCTGCTGGCACGTAGTTAGCCGGCGCTTTTTCTGCAGGTACCGTCACTTTCGCTTCTTCC
CTGCTAAAAGAGGTTTACAACCCGAAGGCCGTCATCCCTCACGCGGCGTTGCTGCATCAGGCTTTCGCCCATTGTGCAATATTCCCCACTGCTGCCTCCC
GTAGGAGTCTGGGCCGTGTCTCAGTCCCAGTGTGGCCGGTCACCCTCTCAGGCCGGCTACCCGTCGACGCCTTGGTGAGCCATTACCTCACCAACAAGCT
GATAGGCCGCGAGCCCATCCCCAACCGAAATTCTTTCCAGACGCAGACCATGCGGCCACGTCACATATCCAGTATTAGACGCCGTTTCCAGCGCTTATCC
CAGAGTCAGGGGCAGGTTGCTCACGTGTTACTGCGACCCGTTCGCCACTGATCCACAGAGCAAGCTCTGC

>EB209
TGCAGTCGAACGGTGAACACGGAGCTTGCTCTGTGGGATCAGTGGCGAACGGGTGAGTAACACGTGAGCAACCTGCCCCTGACTCTGGGATAAGCGCTGG
AAACGGCGTCTAATACTGGATATGTGACGTGACCGCATGGTCTGCGTTTGGAAAGATTTTTCGGTTGGGGATGGGCTCGCGGCCTATCAGCTTGTTGGTG
AGGTAATGGCTCACCAAGGCGTCGACGGGTAGCCGGCCTGAGAGGGTGACCGGCCACACTGGGACTGAGACACGGCCCAGACTCCTACGGGAGGCAGCAG
TGGGGAATATTGCACAATGGGCGAAAGCCTGATGCAGCAACGCCGCGTGAGGGATGACGGCCTTCGGGTTGTAAACCTCTTTTAGCAGGGAAGAAGCGAA
AGTGACGGTACCTGCAGAAAAAGCGCCGGCTAACTACGTGCCAGCAGCCGCGGTAATACGTAGGGCGCAAGCGTTATCCGGAATTATTGGGCGTAAAGAG
CTCGTAGGCGGTTTGTCGCGTCTGCTGTGAAATCCCGAGGCTCAACCTCGGGCCTGCAGTGGGTACGGGCAGACTAGAGTGCGGTAGGGGAGATTGGAAT
TCCTGGTGTAGCGGTGGAATGCGCAGATATCAGGAGGAACACCGATGGCGAAGGCAGATCTCTGGGCCGTAACTGACGCTGAGGAGCGAAAGGGTGGGGA
GCAAACAGGCTTAGATACCCTGGTAGTCCACCCCGTAAACGTTGGGAACTAGTTGTGGGGTCCATTCCACGGATTCCGTGACGCAGCTAACGCATTAAGT
TCCCCGCCTGGGGAGTACGGCCGCAAGGCTAAAACTCAAAGGAATTGACGGGGACCCGCACAAGCGGCGGAGCATGCGGATTAATTCGATGCAACGCGAA

GAACCTTACCAAGGCTTGACATATACGAGAACGGGCCAGAAATGGTCAACTCTTTGGGACACTCGTAAACAGGTGGTGCATGGTTGTCGTCAGCTCGTGT
CGTGAGATGTTGGGTTAAGTCCCGCAACGAGCGCAACCCTCGTTCTATGTTGCCAGCACGTAATGGTGGGAACTCATGGGATACTGCCGGGGTCAACTCG
GAGGAAGGTGGGGATGACGTCAAATCATCATGCCCCTTATGTCTTGGGCTTCACGCATGCTACAATGGCCGGTACAAAGGGCTGCAATACCGTGAGGTGG
AGCGAATCCCAAAAAGCCGGTCCCAGTTCGGATTGAGGTCTGCAACTCGACCTCATGAAGTCGGAGTCGCTAGTAATCGCAGATCAGCAACGCTGCGGTG
AATACGTTCCCGGGTCTTGTACACACCGCCCGTCAAGTCATGAAAGTCGGTAACACCTGAAGCCGGTGGCCTAACCCTTGTGGAGGGAG

>EB210
TGCAAGTCGAACGGTGAACACGGAGCTTGCTCTGTGGGATCAGTGGCGAACGGGTGAGTAACACGTGAGCAACCTGCCCCTGACTCTGGGATAAGCGCTG
GAAACGGCGTCTAATACTGGATATGTGACGTGACCGCATGGTCTGCGTTTGGAAAGATTTTTCGGTTGGGGATGGGCTCGCGGCCTATCAGCTTGTTGGT
GAGGTAATGGCTCACCAAGGCGTCGACGGGTAGCCGGCCTGAGAGGGTGACCGGCCACACTGGGACTGAGACACGGCCCAGACTCCTACGGGAGGCAGCA
GTGGGGAATATTGCACAATGGGCGAAAGCCTGATGCAGCAACGCCGCGTGAGGGATGACGGCCTTCGGGTTGTAAACCTCTTTTAGCAGGGAAGAAGCGA
AAGTGACGGTACCTGCAGAAAAAGCGCCGGCTAACTACGTGCCAGCAGCCGCGGTAATACGTAGGGCGCAAGCGTTATCCGGAATTATTGGGCGTAAAGA
GCTCGTAGGCGGTTTGTCGCGTCTGCTGTGAAATCCCGAGGCTCAACCTCGGGCCTGCAGTGGGTACGGGCAGACTAGAGTGCGGTAGGGGAGATTGGAA
TTCCTGGTGTAGCGGTGGAATGCGCAGATATCAGGAGGAACACCGATGGCGAAGGCAGATCTCTGGGCCGTAACTGACGCTGAGGAGCGAAAGGGTGGGG
AGCAAACAGGCTTAGATACCCTGGTAGTCCACCCCGTAAACGTTGGGAACTAGTTGTGGGGTCCATTCCACGGATTCCGTGACGCAGCTAACGCATTAAG
TTCCCCGCCTGGGGAGTACGGCCGCAAGGCTAAAACTCAAAGGAATTGACGGGGACCCGCACAAGCGGCGGAGCATGCGGATTAATTCGATGCAACGCGA
AGAACCTTACCAAGGCTTGACATATACGAAAACGGGCCAGAAATGGTCAACTCTTTGGACACTCGTAAACAGGTGGTGCATGGTTGTCGTCAGCTCGTGT
CGTGAGATGTTGGGTTAAGTCCCGCAACGAGCGCAACCCTCGTTCTATGTTGCCAGCACGTAATGGTGGGAACTCATGGGATACTGCCGGGGTCAACTCG
GAGGAAGGTGGGGATGACGTCAAATCATCATGCCCCTTATGTCTTGGGCTTCACGCATGCTACAATGGCCGGTACAAAGGGCTGCAATACCGTGAGGTGG
AGCGAATCCCAAAAAGCCGGTCCCAGTTCGGATTGAGGTCTGCAACTCGACCTCATGAAGTCGGAGTCGCTAGTAATCGCAGATCAGCAACGCTGCGGTG
AATACGTTCCCGGGTCTTGTACACACCGCCCGTCAAGTCATGAAAGTCGGTAACACCTGAAGCCGGTGGCCTAACCCTTGTGGAGGGAGCC

>EB211
TGCAAGTCGAACGGTGAACACGGAGCTTGCTCTGTGGGATCAGTGGCGAACGGGTGAGTAACACGTGAGCAACCTGCCCCTGACTCTGGGATAAGCGCTG
GAAACGGCGTCTAATACTGGATATGTGACGTGACCGCATGGTCTGCGTTTGGAAAGATTTTTCGGTTGGGGATGGGCTCGCGGCCTATCAGCTTGTTGGT
GAGGTAATGGCTCACCAAGGCGTCGACGGGTAGCCGGCCTGAGAGGGTGACCGGCCACACTGGGACTGAGACACGGCCCAGACTCCTACGGGAGGCAGCA
GTGGGGAATATTGCACAATGGGCGAAAGCCTGATGCAGCAACGCCGCGTGAGGGATGACGGCCTTCGGGTTGTAAACCTCTTTTAGCAGGGAAGAAGCGA
AAGTGACGGTACCTGCAGAAAAAGCGCCGGCTAACTACGTGCCAGCAGCCGCGGTAATACGTAGGGCGCAAGCGTTATCCGGAATTATTGGGCGTAAAGA
GCTCGTAGGCGGTTTGTCGCGTCTGCTGTGAAATCCCGAGGCTCAACCTCGGGCCTGCAGTGGGTACGGGCAGACTAGAGTGCGGTAGGGGAGATTGGAA
TTCCTGGTGTAGCGGTGGAATGCGCAGATATCAGGAGGAACACCGATGGCGAAGGCAGATCTCTGGGCCGTAACTGACGCTGAGGAGCGAAAGGGTGGGG
AGCAAACAGGCTTAGATACCCTGGTAGTCCACCCCGTAAACGTTGGGAACTAGTTGTGGGGTCCATTCCACGGATTCCGTGACGCAGCTAACGCATTAAG
TTCCCCGCCTGGGGAGTACGGCCGCAAGGCTAAAACTCAAAGGAATTGACGGGGACCCGCACAAGCGGCGGAGCATGCGGATTAATTCGATGCAACGCG
AAGAACCTTACCAAGGCTTGACATATACGAGAACGGGCCAGAAATGGTCAACTCTTTGGACACTCGTAAACAGGTGGTGCATGGTTGTCGTCAGCTCGTG
TCGTGAGATGTTGGGTTAAGTCCCGCAACGAGCGCAACCCTCGTTCTATGTTGCCAGCACGTAATGGTGGGAACTCATGGGATACTGCCGGGGTCAACTC
GGAGGAAGGTGGGGATGACGTCAAATCATCATGCCCCTTATGTCTTGGGCTTCACGCATGCTACAATGGCCGGTACAAAGGGCTGCAATACCGTGAGGTG
GAGCGAATCCCAAAAAGCCGGTCCCAGTTCGGATTGAGGTCTGCAACTCGACCTCATGAAGTCGGAGTCGCTAGTAATCGCAGATCAGCAACGCTGCGGT
GAATACGTTCCCGGGTCTTGTACACACCGCCCGTCAAGTCATGAAAGTCGGTAACACCTGAAGCCGGTGGCCTAACCCTTGTGGAGGGAGCCG

>EB212
GTCGAACGGTGAAGCCAAGCTTGCTTGGTGGATCAGTGGCGAACGGGTGAGTAACACGTGAGCAACCTGCCCTGGACTCTGGGATAAGCGCTGGAAACGG
CGTCTAATACTGGATATGAGCCCCTTCCGCATGGTGGGGGTTGGAAAGATTTTTTGGTCTGGGATGGGCTCGCGGCCTATCAGCTTGTTGGTGAGGTAAT
GGCTCACCAAGGCGTCGACGGGTAGCCGGCCTGAGAGGGTGACCGGCCACACTGGGACTGAGACACGGCCCAGACTCCTACGGGAGGCAGCAGTGGGGAA
TATTGCACAATGGGCGGAAGCCTGATGCAGCAACGCCGCGTGAGGGATGACGGCCTTCGGGTTGTAAACCTCTTTTAGCAGGGAAGAAGCGAGAGTGACG
GTACCTGCAGAAAAAGCGCCGGCTAACTACGTGCCAGCAGCCGCGGTAATACGTAGGGCGCAAGCGTTATCCGGAATTATTGGGCGTAAAGAGCTCGTAG
GCGGTTTGTCGCGTCTGCTGTGAAATCCCGAGGCTCAACCTCGGGCCTGCAGTGGGTACGGGCAGACTAGAGTGCGGTAGGGGAGATTGGAATTCCTGGT
GTAGCGGTGGAATGCGCAGATATCAGGAGGAACACCGATGGCGAAGGCAGATCTCTGGGCCGTAACTGACGCTGAGGAGCGAAAGGGTGGGGAGCAAACA
GGCTTAGATACCCTGGTAGTCCACCCCGTAAACGTTGGGAACTAGTTGTGGGGACCATTCCACGGTTTCCGTGACGCAGCTAACGCATTAAGTTCCCCGC
CTGGGGGAGTACGGCCGCAAGGCTAAAACTCAAAGGAATTGACGGGGACCCGCACAAGCGGCGGAGCATGCGGATTAATTCGATGCCAACGCGAAGAACC
TTACCAAGGCTTGACATACACCAGAACACCGTAGAAATACGGGACTCTTTGGACACTGGTGAACAGGTGGTGCATGGTTGTCGTCAGCTCGTGTCGTGAG
ATGTTGGGTTAAGTCCCGCAACGAGCGCAACCCTCGTTCTATGTTGCCAGCACGTAATGGTGGGAACTCATGGGATACTGCCGGGGTCAACTCGGAGGAA
GGTGGGGATGACGTCAAATCATCATGCCCCTTATGTCTTGGGCTTCACGCATGCTACAATGGCCGGTACAAAGGGCTGCAATACCGTGAGGTGGAGCGAA
TCCCAAAAAGCCGGTCCCAGTTCGGATTGAGGTCTGCAACTCGACCTCATGAAGTCGGAGTCGCTAGTAATCGCAGATCAGCAACGCTGCGGTGAATACG
TTCCCGGGTCTTGTACACACCGCCCGTCAAGTCATGAAAGTCGGTAACACCTGAAGCCGGTGGCCTAACCC

>EB213
TGCAGTCGAACGGTGAAGCCAAGCTTGCTTGGTGGATCAGTGGCGAACGGGTGAGTAACACGTGAGCAACCTGCCCTGGACTCTGGGATAAGCGCTGGAA
ACGGCGTCTAATACTGGATATGAGCCCCTATCGCATGGTGGGGGTTGGAAAGATTTTTTGGTCTGGGATGGGCTCGCGGCCTATCAGCTTGTTGGTGAGG
TAATGGCTCACCAAGGCGTCGACGGGTAGCCGGCCTGAGAGGGTGACCGGCCACACTGGGACTGAGACACGGCCCAGACTCCTACGGGAGGCAGCAGTGG
GGAATATTGCACAATGGGCGAAAGCCTGATGCAGCAACGCCGCGTGAGGGATGACGGCCTTCGGGTTGTAAACCTCTTTTAGCAGGGAAGAAGCGAAAAG
TGACGGTACCTGCAGAAAAAGCGCCGGCTAACTACGTGCCAGCAGCCGCGGTAATACGTAGGGCGCAAGCGTTATCCGGAATTATTGGGCGTAAAGAGCT
CGTAGGCGGTTTGTCGCGTCTGCTGTGAAATCCCGAGGCTCAACCTCGGGCCTGCAGTGGGTACGGGCAGACTAGAGTGCGGTAGGGGAGATTGGAATTC
CTGGTGTAGCGGTGGAATGCGCAGATATCAGGAGGAACACCGATGGCGAAGGCAGATCTCTGGGCCGTAACTGACGCTGAGGAGCGAAAGGGTGGGGAGC
AAACAGGCTTAGATACCCTGGTAGTCCACCCCGTAAACGTTGGGAACTAGTTGTGGGGACCATTCCACGGTTTCCGTGACGCAGCTAACGCATTAAGTTC
CCCGCCTGGGGAGTACGGCCGCAAGGCTAAAACTCAAAGGAATTGACGGGGACCCGCACAAGCGGCGGAGCATGCGGATTAATTCGATGCAACGCGAAGA
ACCTTACCAAGGCTTGACATATACGAAAACGGGCCAGAAATGGTCAACTCTTTGGACACTCGTAAACAGGTGGTGCATGGTTGTCGTCAGCTCGTGTCGT
GAGATGTTGGGTTAAGTCCCGCAACGAGCGCAACCCTCGTTCTATGTTGCCAGCACGTAATGGTGGGAACTCATGGGATACTGCCGGGGTCAACTCGGAG
GAAGGTGGGGATGACGTCAAATCATCATGCCCCTTATGTCTTGGGCTTCACGCATGCTACAATGGCCGGTACAAAGGGCTGCAATACCGTGAGGTGGAGC
GAATCCCAAAAAGCCGGTCCCAGTTCGGATTGAGGTCTGCAACTCGACCTCATGAAGTCGGAGTCGCTAGTAATCGCAGATCAGCAACGCTGCGGTGAAT
ACGTTCCCGGGTCTTGTACACACCGCCCGTCAAGTCATGAAAGTCGGTAACACCTGAAGCCGGTGGCCCAACCCTTGTGGAGGGAGCCGT

>EB214
GCAGTCGAACGGTGAAGAGAGCTTGCTCTCTGGATCAGTGGCGAACGGGTGAGTAACACGTGAGCAACCTGCCCCGGACTCTGGGATAACAGCTGGAAAC
AGCTGCTAATACCGGATACGAGCTGCGAAGGCATCTTCAGCAGCTGGAAAGAATTTCGGTCCGGGATGGGCTCGCGGCCTATCAGCTAGTTGGTGAGGTA
ATGGCTCACCAAGGCGTCGACGGGTAGCCGGCCTGAGAGGGTGACCGGCCACACTGGGACTGAGACACGGCCCAGACTCCTACGGGAGGCAGCAGTGGGG
AATATTGCACAATGGGCGAAAGCCTGATGCAGCAACGCCGCGTGAGGGATGACGGCCTTCGGGTTGTAAACCTCTTTTAGCAGGGAAGAAGCGAAAGTGA
CGGTACCTGCAGAAAAAGCGCCGGCTAACTACGTGCCAGCAGCCGCGGTAATACGTAGGGCGCAAGCGTTATCCGGAATTATTGGGCGTAAAGAGCTCGT
AGGCGGTTTGTCGCGTCTGCTGTGAAAACCCGAGGCTCAACCTCGGGCCTGCAGTGGGTACGGGCAGACTAGAGTGCGGTAGGGGAGATTGGAATTCCTG
GTGTAGCGGTGGAATGCGCAGATATCAGGAGGAACACCGATGGCGAAGGCAGATCTCTGGGCCGTAACTGACGCTGAGGAGCGAAAGGGTGGGGAGCAAA

CAGGCTTAGATACCCTGGTAGTCCACCCCGTAAACGTTGGGAACTAGTTGTGGGGTCCATTCCACGGATTCCGTGACGCAGCTAACGCATTAAGTTCCCC
GCCTGGGGAGTACGGCCGCAAGGNCTAAAACTCAAAGGAATTGACGGGGNACCCGCACAAGCGGCGGAGCATGCGGNATTAATTNCGATGCAACGCGAAG
AACCTTNACCAAGGGCTTGACATATACGAGAANCGGGCCNAGAAATGGTCAACTCTTTGGACACTCGTAAAACAGGTGGNTGCATGGGTTGTCGTCAGCN
TCGTGNTCGTGAGATGTTGGGTTAAGTCCCNGCAANCGANGCGCAACCCTCGTTCTATGTTGCCAGCACGTAATGGTGGGAACTCATGGGATACTGCCGG
GGTCAACTCGGAGGAAGGTGGGGATGACGTCAAATCATCATGCCCCTTATGTCTTGGGCTTCACGCATGCTACAATGGCCGGTACAAAGGGCTGCAATAC
CGCAAGGTGGAGCGAATCCCAAAAAGCCGGTCCCAGTTCGGATTGAGGTCTGCAACTCGACCTCATGAAGTCGGAGTCGCTAGTAATCGCAGATCAGCAA
CGCTGCGGTGAATACGTTCCCGGGTCTTGTACACACCGCCCGTCAAGTCATGAAAGTCGGTAACACCCGAAGCCGGTGGCCCAACCCTTGTGGAGGGAGC
C

>EB215
CCATGCAGTCGAACGGTGAACACGGAGCTTGCTCTGTGGGATCAGTGGCGAACGGGTGAGTAACACGTGAGCAACCTGCCCCTGACTCTGGGATAAGCGC
TGGAAACGGCGTCTAATACTGGATATGTGACGTGACCGCATGGTCTGCGTCTGGAAAGAATTTCGGTTGGGGATGGGCTCGCGGCCTATCAGCTTGTTGG
TGAGGTAATGGCTCACCAAGGCGTCGACGGGTAGCCGGCCTGAGAGGGTGACCGGCCACACTGGGACTGAGACACGGCCCAGACTCCTACGGGAGGCAGC
AGTGGGGAATATTGCACAATGGGCGCAAGCCTGATGCAGCAACGCCGCGTGAGGGACGACGGCCTTCGGGTTGTAAACCTCTTTTAGCAGGGAAGAAGCG
AAAAGTGACGGTACCTGCAGAAAAAAGCGCCGGCTAACTACGTGCCAGCAGCCGCGGTAATACGTAGGGCGCAAGCGTTATCCGGAATTATTGGGCGTAA
AGAGCTCGTAGGCGGTTTGTCGCGTCTGCTGTGAAATCCGGAGGCTCAACCTCCGGCCTGCAGTGGGTACGGGCAGACTAGAGTGCGGTAGGGGAGATTG
GAATTCCTGGTGTAGCGGTGGAATGCGCAGATATCAGGAGGAACACCGATGGCGAAGGCAGATCTCTGGGCCGTAACTGACGCTGAGGAGCGAAAGGGTG
GGGAGCAAACAGGCTTAGATACCCTGGTAGTCCACCCCGTAAACGTTGGGAACTAGTTGTGGGGTCCATTCCACGGATTCCGTGACGCAGCTAACGCATT
AAGTTCCCCGCCTGGGGAGTACGGCCGCAAGGCTAAAACTCAAAGGAATTGACGGGGACCCGCACAAGCGGCGGAGCATGCGGATTAATTCGATGCAACG
CGAAGAACCTTACCAAGGCTTGACATATACGAGAACGGGCCAGAAATGGTCAACTCTTTGGACACTCGTAAACAGGTGGTGCATGGTTGTCGTCAGCTCG
TGTCGTGAGATGTTGGGTTAAGTCCCGCAACGAGCGCAACCCTCGTTCTATGTTGCCAGCACGTAATGGTGGGAACTCATGGGATACTGCCGGGGTCAAC
TCGGAGGAAGGTGGGGATGACGTCAAATCATCATGCCCCTTATGTCTTGGGCTTCACGCATGCTACAATGGCCGGTACAAAGGGCTGCAATACCGCGAGG
TGGAGCGAATCCCAAAAAGCCGGTCCCAGTTCGGATTGAGGTCTGCAACTCGACCTCATGAAGTCGGAGTCGCTAGTAATCGCAGATCAGCAACGCTGCG
GTGAATACGTTCCCGGGTCTTGTACACACCGCCCGTCAAGTCATGAAAGTCGGTAACACCTGAAGCCGGTGGCCTAACCCTTGTGGAGGGAGCC

>EB216
TGCAGTCGAACGGTGAAGCAGAGCTTGCTCTGTGGATCAGTGGCGAACGGGTGAGTAACACGTGAGCAACCTGCCCTGGACTCTGGGATAAGCGCTGGAA
ACGGCGTCTAATACTGGATACGAGACGTGGCCGCATGGTCAACGTTTGGAAAGATTTTTTGGTTCAGGATGGGCTCGCGGCCTATCAGCTTGTTGGTGAG
GTAATGGCTCACCAAGGCGTCGACGGGTAGCCGGCCTGAGAGGGTGACCGGCCACACTGGGACTGAGACACGGCCCAGACTCCTACGGGAGGCAGCAGTG
GGGAATATTGCACAATGGGCGAAAGCCTGATGCAGCAACGCCGCGTGAGGGATGACGGCCTTCGGGTTGTAAACCTCTTTTAGCAAGGAAGAAGCGAAAG
TGACGGTACTTGCAGAAAAAGCGCCGGCTAACTACGTGCCAGCAGCCGCGGTAATACGTAGGGCGCAAGCGTTATCCGGAATTATTGGGCGTAAAGAGCT
CGTAGGCGGTTTGTCGCGTCTGCTGTGAAAACTGGAGGCTCAACCTCCAGCCTGCAGTGGGTACGGGCAGACTAGAGTGCGGTAGGGGAGATTGGAATTC
CTGGTGTAGCGGTGGAATGCGCAGATATCAGGAGGAACACCGATGGCGAAGGCAGATCTCTGGGCCGTAACTGACGCTGAGGAGCGAAAGGGTGGGGAGC
AAACAGGCTTAGATACCCTGGTAGTCCACCCCGTAAACGTTGGGAACTAGTTGTGGGGACCATTCCACGGTTTCCGTGACGCAGCTAACGCATTAAGTTC
CCCGCCTGGGGAGTACGGCCGCAAGGCTAAAACTCAAAGGAATTGACGGGGACCCGCACAAGCGGCGGAGCATGCGGATTAATTCGATGCAACGCGAAGA
ACCTTACCAAGGCTTGACATATACGAGAACGCTGCAGAAATGTAGAACTCTTTGGACACTCGTATACAGGTGGTGCATGGTTGTCGTCAGCTCGTGTCGT
GAGATGTTGGGTTAAGTCCCGCAACGAGCGCAACCCTCGTTCTATGTTGCCAGCACGTAATGGTGGGAACTCATGGGATACTGCCGGGGTCAACTCGGAG
GAAGGTGGGGATGACGTCAAATCATCATGCCCCTTATGTCTTGGGCTTCACGCATGCTACAATGGCCGGTACAAAGGGCTGCAATACCGTGAGGTGGAGC
GAATCCCAAAAAGCCGGTCCCAGTTCGGATTGAGGTCTGCAACTCGACCTCATGAAGTCGGAGTCGCTAGTAATCGCAGATCAGCAACGCTGCGGTGAAT
ACGTTCCCGGGTCTTGTACACACCGCCCGTCAAGTCATGAAAGTCGGTAACACCTGAAGCCGGTGGCCTAACCCTTGTGGAGGGAGCCGTCG

>EB217
GTTGCAAACTGCTCGTGGTGTGACGGGCGGTGTGTACAAGGCCCGGGAACGTATTCACCGCGGCATGCTGATCCGCGATTACTAGCGATTCCAGCTTCAC
GCAGTCGAGTTGCAGACTGCGATCCGAACTGAGAACAGATTTGTGGGATTGGCTAAACCTTGCGGTCTCGCAGCCCTTTGTTCTGTCCATTGTAGCACGT
GTGTAGCCCAGGTCATAAGGGGCATGATGATTTGACGTCATCCCCACCTTCCTCCGGTTTGTCACCGGCAGTCACCTTAGAGTGCCCAACTAAATGCTGG
CAACTAAGATCAAGGGTTGCGCTCGTTGCGGGACTTAACCCAACATCTCACGACACGAGCTGACGACAACCATGCACCACCTGTCACTCTGTCCCCGAAG
GGAAAGCCCTATCTCTAGGGTTGTCAGAGGATGTCAAGACCTGGTAAGGTTCTTCGCGTTGCTTCGAATTAAACCACATGCTCCACCGCTTGTGCGGGCC
CCCGTCAATTCCTTTGAGTTTCAGTCTTGCGACCGTACTCCCCAGGCGGAGTGCTTAATGCGTTAGCTGCAGCACTAAGGGGCGGAAACCCCCTAACACT
TAGCACTCATCGTTTACGGCGTGGACTACCAGGGTATCTAATCCTGTTCGCTCCCCACGCTTTCGCTCCTCAGCGTCAGTTACAGACCAGAGAGTCGCCT
TCGCCACTGGTGTTCCTCCACATCTCTACGCATTTCACCGCTACACGTGGAATTCCACTCTCCTCTTCTGCACTCAAGTTTCCCAGTTTCCAATGACCCT
CCCCGGTTGAGCCGGGGGCTTTCACATCAGACTTAAGAAACCGCCTGCGAGCCCTTTACGCCCAATAATTCCGGACAACGCTTGCCACCTACGTATTACC
GCGGCTGCTGGCACGTAGTTAGCCGTGGCTTTCTGGTTAGGTACCGTCAAGGTGCAAGCAGTTACTCTTGCACTTGTTCTTCCCTAACAACAGAGCTTTA
CGATCCGAAAACCTTCATCACTCACGCGGCGTTGCTCCGTCAGACTTTCGTCCATTGCGGAAGATTCCCTACTGCTGCCTCCCGTAGGAGTCTGGGCCGT
GTCTCAGTCCCAGTGTGGCCGATCACCCTCTCAGGTCGGCTACGCATCGTCGCCTTGGTGAGCCGTTACCTCACCAACTAGCTAATGCGCCGCGGGTCCA
TCTGTAAGTGACAGCCGAAACCGTCTTTCATCCTTGAACCATGCGGTTCAAGGAACTATCCGGTATTAGCTCCGGTTTCCCGGAGTTATCCCAGTCTTAC
AGGCAGGTTACCCACGTGTTACTCACCCGTCCGCCGCTAACATCACGGGAGCAAGCT

>EB218
CGACGGCTCCTCCACAAGGGTTAGGCCACCGGCTTCAGGTGTTACCGACTTTCATGACTTGACGGGCGGTGTGTACAAGACCCGGGAACGTATTCACCGC
AGCGTTGCTGATCTGCGATTACTAGCGACTCCGACTTCATGAGGTCGAGTTGCAGACCTCAATCCGAACTGGGACCGGCTTTTTGGGATTCGCTCCACCT
CGCGGTATTGCAGCCCTTTGTACCGGCCATTGTAGCATGCGTGAAGCCCAAGACATAAGGGGCATGATGATTTGACGTCATCCCCACCTTCCTCCGAGTT
GACCCCGGCAGTATCCCATGAGTTCCCACCATTACGTGCTGGCAACATAGAACGAGGGTTGCGCTCGTTGCGGGACTTAACCCAACATCTCACGACACGA
GCTGACGACAACCATGCACCACCTGTTTACGAGTGTCCAAAGAGTTGACCATTTCTGGCCCGTTCTCGTATATGTCAAGCCTTGGTAAGGTTCTTCGCGT
TGCATCGAATTAATCCGCATGCTCCGCCGCTTGTGCGGGTCCCCGTCAATTCCTTTGAGTTTTAGCCTTGCGGCCGTACTCCCCAGGCGGGGAACTTAAT
GCGTTAGCTGCGTCACGGAATCCGTGGAATGGACCCCACAACTAGTTCCCAACGTTTACGGGGTGGACTACCAGGGTATCTAAGCCTGTTTGCTCCCCAC
CCTTTCGCTCCTCAGCGTCAGTTACGGCCCAGAGATCTGCCTTCGCCATCGGTGTTCCTCCTGATATCTGCGCATTCCACCGCTACACCAGGAATTCCAA
TCTCCCCTACCGCACTCTAGTCTGCCCGTACCCACTGCAGGCCGGAGGTTGAGCCTCCGGATTTCACAGCAGACGCGACAAACCGCCTACGAGCTCTTTA
CGCCCAATAATTCCGGATAACGCTTGCGCCCTACGTATTACCGCGGCTGCTGGCACGTAGTTAGCCGGCGCTTTTTCTGCAGGTACCGTCACTTTCGCTT
CTTCCCTGCTAAAAGAGGTTTACAACCCGAAGGCCGTCGTCCCTCACGCGGCGTTGCTGCATCAGGCTTGCGCCCATTGTGCAATATTCCCCACTGCTGC
CTCCCGTAGGAGTCTGGGCCGTGTCTCAGTCCCAGTGTGGCCGGTCACCCTCTCAGGCCGGCTACCCGTCGACGCCTTGGTGAGCCATTACCTCACCAAC
AAGCTGATAGGCCGCGAGCCCATCCCCAACCGAAATTCTTTCCAGACGCAGACCATGCGGTCACGTCACATATCCAGTATTAGACGCCGTTTCCAGCGCT
TATCCCAGAGTCAGGGGCAGGTTGCTCACGTGTTACTCACCCGTTCGCCACTGATCCCACAGAGCAAGCTCCGTGTTCACCGTTCGACTGCATG

>EB219
TGCAGTCGAACGATGATGCCCAGCTTGCTGGGTGGATTAGTGGCGAACGGGTGAGTAACACGTGAGCAACCTGCCCCTGACTCTGGGATAAGCGCTGGAA
ACGGCGTCTAATACTGGATACGAGTAGCGATCGCATGGTCAGTTACTGGAAAGATTTTTTGGTTGGGGATGGGCTCGCGGCCTATCAGCTTGTTGGTGAG
GTAATGGCTCACCAAGGCGTCGACGGGTAGCCGGCCTGAGAGGGTGACCGGCCACACTGGGACTGAGACACGGCCCAGACTCCTACGGGAGGCAGCAGTG
GGGAATATTGCACAATGGGCGGAAGCCTGATGCAGCAACGCCGCGTGAGGGATGACGGCCTTCGGGTTGTAAACCTCTTTTAGCAGGGAAGAAGCGAGAG

TGACGGTACCTGCAGAAAAAGCGCCGGCTAACTACGTGCCAGCAGCCGCGGTAATACGTAGGGCGCAAGCGTTATCCGGAATTATTGGGCGTAAAGAGCT
CGTAGGCGGTTTGTCGCGTCTGCTGTGAAATCCCGAGGCTCAACCTCGGGCCTGCAGTGGGTACGGGCAGACTAGAGTGCGGTAGGGGAGATTGGAATTC
CTGGTGTAGCGGTGGAATGCGCAGATATCAGGAGGAACACCGATGGCGAAGGCAGATCTCTGGGCCGTAACTGACGCTGAGGAGCGAAAGGGTGGGGAGC
AAACAGGCTTAGATACCCTGGTAGTCCACCCCGTAAACGTTGGGAACTAGTTGTGGGGTCCATTCCACGGATTCCGTGACGCAGCTAACGCATTAAGTTC
CCCGCCTGGGGAGTACGGCCGCAAGGCTAAAACTCAAAGGAATTGACGGGGACCCGCACAAGCGGCGGAGCATGCGGATTAATTCGATGCAACGCGAAGA
ACCTTACCAAGGCTTGACATATACGAAAACGGGCCAGAAATGGTCAACTCTTTGGACACTCGTAAACAGGTGGTGCATGGTTGTCGTCAGCTCGTGTCGT
GAGATGTTGGGTTAAGTCCCGCAACGAGCGCAACCCTCGTTCTATGTTGCCAGCACGTAATGGTGGGAACTCATGGGATACTGCCGGGGTCAACTCGGAG
GAAGGTGGGGATGACGTCAAATCATCATGCCCCTTATGTCTTGGGCTTCACGCATGCTACAATGGCCGGTACAATGGGCAGCGATACCGTGAGGTGGAGC
GAATCCCAAAAAGCCGGTCCCAGTTCGGATTGAGGTCTGCAACTCGACCTCATGAAGTCGGAGTCGCTAGTAATCGCAGATCAGCAACGCTGCGGTGAAT
ACGTTCCCGGGTCTTGTACACACCGCCCGTCAAGTCATGAAAGTCGGTAACACCTGAAGCCGGTGGCCTAACCCCTTGTGGGAGGGAG

>EB220
ATGCAGTCGAACGGTGAAGCCAAGCTTGCTTGGTGGATCAGTGGCGAACGGGTGAGTAACACGTGAGCAACCTGCCCTGGACTCTGGGATAAGCGCTGGA
AACGGCGTCTAATACTGGATATGAGCCTCTTCCGCATGGTGGGGGTTGGAAAGATTTTTTGGTCTGGGATGGGCTCGCGGCCTATCAGCTTGTTGGTGAG
GTAATGGCTCACCAAGGCGTCGACGGGTAGCCGGCCTGAGAGGGTGACCGGCCACACTGGGACTGAGACACGGCCCAGACTCCTACGGGAGGCAGCAGTG
GGGAATATTGCACAATGGGCGAAAGCCTGATGCATCAACGCCGCGTGAGGGATGACGGCCTTCGGGTTGTAAACCTCTTTTAGCCAGGGAAGAAAGCGAA
AAGTGACCGGTACCTGCAGAAAAAAGCGCCGGGCTAACTACGTGCCCAGCAGCCGCGGTAATACGTAGGGCGCAAGCGTTATCCGGGAATTATTGGGCGT
AAAGAGCTCGTAGGCGGTTTGTCGCGTCTGCTGTGAAATCCCGAGGCTCAACCTCGGGCCTGCAGTGGGTACGGGCAGACTAGAGTGCGGTAGGGGAGAT
TGGAATTCCTGGTGTAGCGGTGGAATGCGCAGATATCAGGAGGAACACCGATGGCGAAGGCAGATCTCTGGGCCGTAACTGACGCTGAGGAGCGAAAGGG
TGGGGAGCAAACAGGCTTAGATACCCTGGTAGTCCACCCCGTAAACGTTGGGAACTAGTTGTGGGGACCATTCCACGGTTTCCGTGACGCAGCTAACGCA
TTAAGTTCCCCGCCTGGGGAGTACGGCCGCAAGGCTAAAACTCAAAGGAATTGACGGGGACCCGCACAAGCGGCGGAGCATGCGGATTAATTCGATGCAA
CGCGAAGAACCTTACCAAGGCTTGACATACACCAGAACGGGCCAGAAATGGTCAACTCTTTGGACACTGGTGAACAGGTGGTGCATGGTTGTCGTCAGCT
CGTGTCGTGAGATGTTGGGTTAAGTCCCGCAACGAGCGCAACCCTCGTTCTATGTTGCCAGCACGTAATGGTGGGAACTCATGGGATACTGCCGGGGTCA
ACTCGGAGGAAGGTGGGGATGACGTCAAATCATCATGCCCCTTATGTCTTGGGCTTCACGCATGCTACAATGGCCGGTACAAAGGGCTGCAATACCGTGA
GGTGGAGCGAATCCCAAAAAGCCGGTCCCAGTTCGGATTGAGGTCTGCAACTCGACCTCATGAAGTCGGAGTCGCTAGTAATCGCAGATCAGCAACGCTG
CGGTGAATACGTTCCCGGGTCTTGTACACACCGCCCGTCAAGTCATGAAAGTCGGTAACACCTGAAGCCGGTGGCCCAACCCTTGTGGAGGGAGCC

>EB221
GTCGAACGGTGAAGCCCAGCTTGCTGGGTGGATCAGTGGCGAACGGGTGAGTAACACGTGAGCAACCTGCCCTGGACTCTGGGATAAGCGCTGGAAACGG
CGTCTAATACTGGATATGAACCACGAAGGCATCTTCAGTGGTTGGAAAGATTTTTCGGTCTGGGATGGGCTCGCGGCCTATCAGCTTGTTGGTGAGGTAA
TGGCTCACCAAGGCGTCGACGGGTAGCCGGCCTGAGAGGGTGACCGGCCACACTGGGACTGAGACACGGCCCAGACTCCTACGGGAGGCAGCAGTGGGGA
ATATTGCACAATGGGCGGAAGCCTGATGCAGCAACGCCGCGTGAGGGATGACGGCCTTCGGGTTGTAAACCTCTTTTAGCAAGGAAGAAGCTTTTGTGAC
GGTACTTGCAGAAAAAGCGCCGGCTAACTACGTGCCAGCAGCCGCGGTAATACGTAGGGCGCAAGCGTTATCCGGAATTATTGGGCGTAAAGAGCTCGTA
GGCGGTTTGTCGCGTCTGCTGTGAAATCCCGAGGCTCAACCTCGGGCCTGCAGTGGGTACGGGCAGACTAGAGTGCGGTAGGGGAGATTGGAATTCCTGG
TGTAGCGGTGGAATGCGCAGATATCAGGAGGAACACCGATGGCGAAGGCAGATCTCTGGGCCGTAACTGACGCTGAGGAGCGAAAGGGTGGGGAGCAAAC
AGGCTTAGATACCCTGGTAGTCCACCCCGTAAACGTTGGGAACTAGTTGTGGGGTCCTTTCCACGGATTCCGTGACGCAGCTAACGCATTAAGTTCCCCG
CCTGGGGAGTACGGCCGCAAGGCTAAAACTCAAAGGAATTGACGGGGACCCGCACAAGCGGCGGAGCATGCGGATTAATTCGATGCAACGCGAAGAACCT
TACCAAGGCTTGACATACACGAAAACACCCTAGAAAATAGGGGACTCTTTGGACACTCGTGAACAGGTGGTGCATGGTTGTCGTCAGCTCGTGTCGTGAG
ATGTTGGGTTAAGTCCCGCAACGAGCGCAACCCTCGTTCTATGTTGCCAGCACGTAATGGTGGGAACTCATGGGATACTGCCGGGGTCAACTCGGAGGAA
GGTGGGGATGACGTCAAATCATCATGCCCCTTATGTCTTGGGCTTCACGCATGCTACAATGGCCGGTACAAAGGGCTGCAATACCGTGAGGTGGAGCGAA
TCCCAAAAAGCCGGTCCCAGTTCGGATTGAGGTCTGCAACTCGACCTCATGAAGTCGGAGTCGCTAGTAATCGCAGATCAGCAACGCTGCGGTGAATACG
TTCCCGGGTCTTGTACACACCGCCCGTCAAGTCATGAAAGTCGGTAACACCTGAAGCCGGTGGCCCAACCC

>EB222
TGCAGTCGACGGTGAAGCCAAGCTTGCTTGGTGGATCAGTGGCGAACGGGTGAGTAACACGTGAGCAACCTGCCCTGGACTCTGGGATAAGCGCTGGAAA
CGGCGTCTAATACTGGATATGAGCTCTCATCGCATGGTGGGGGTTGGAAAGATTTTTTGGTCTGGGATGGGCTCGCGGCCTATCAGCTTGTTGGTGAGGT
AATGGCTCACCAAGGCGTCGACGGGTAGCCGGCCTGAGAGGGTGACCGGCCACACTGGGACTGAGACACGGCCCAGACTCCTACGGGAGGCAGCAGTGGG
GAATATTGCACAATGGGCGAAAGCCTGATGCAGCAACGCCGCGTGAGGGATGACGGCCTTCGGGTTGTAAACCTCTTTTAGCAGGGAAGAAGCGAAAGTG
ACGGTACCTGCAGAAAAAGCGCCGGCTAACTACGTGCCAGCAGCCGCGGTAATACGTAGGGCGCAAGCGTTATCCGGAATTATTGGGCGTAAAGAGCTCG
TAGGCGGTTTGTCGCGTCTGCTGTGAAATCCCGAGGCTCAACCTCGGGCCTGCAGTGGGTACGGGCAGACTAGAGTGCGGTAGGGGAGATTGGAATTCCT
GGTGTAGCGGTGGAATGCGCAGATATCAGGAGGAACACCGATGGCGAAGGCAGATCTCTGGGCCGTAACTGACGCTGAGGAGCGAAAGGGTGGGGAGCAA
ACAGGCTTAGATACCCTGGTAGTCCACCCCGTAAACGTTGGGAACTAGTTGTGGGGACCATTCCACGGTTTCCGTGACGCAGCTAACGCATTAAGTTCCC
CGCCTGGGGGAGTACGGCCGCAAGGCTAAAACTCAAAGGAATTGACGGGGACCCGCACAAGCGGCGGAGCATGCGGATTAATTCGATGCAACGCGAAGAA
CCTTACCAAGGCTTGACATACACCAGAACGGGCCAGAAATGGTCAACTCTTTGGACACTGGTGAACAGGTGGTGCATGGTTGTCGTCAGCTCGTGTCGTG
AGATGTTGGGTTAAGTCCCGCAACGAGCGCAACCCTCGTTCTATGTTGCCAGCACGTAATGGTGGGAACTCATGGGATACTGCCGGGGTCAACTCGGAGG
AAGGTGGGGATGACGTCAAATCATCATGCCCCTTATGTCTTGGGCTTCACGCATGCTACAATGGCCGGTACAAAGGGCTGCAATACCGTGAGGTGGAGCG
AATCCCAAAAAGCCGGTCCCAGTTCGGATTGAGGTCTGCAACTCGACCTCATGAAGTCGGAGTCGCTAGTAATCGCAGATCAGCAACGCTGCGGTGAATA
CGTTCCCGGGTCTTGTACACACCGCCCGTCAAGTCATGAAAGTCGGTAACACCTGAAGCCGGTGGCCCAACCCTTGTGGAGGGAGCC

>EB223
CGGCTCCCTCCACAAGGGTTAGGCCACCGGCTTCAGGTGTTACCGACTTTCATGACTTGACGGGCGGTGTGTACAAGACCCGGGAACGTATTCACCGCAG
CGTTGCTGATCTGCGATTACTAGCGACTCCGACTTCATGAGGTCGAGTTGCAGACCTCAATCCGAACTGGGACCGGCTTTTTGGGATTCGCTCCACCTCG
CGGTATTGCAGCCCTTTGTACCGGCCATTGTAGCATGCGTGAAGCCCAAGACATAAGGGGCATGATGATTTGACGTCATCCCCACCTTCCTCCGAGTTGA
CCCCGGCAGTATCCCATGAGTTCCCACCATTACGTGCTGGCAACATAGAACGAGGGTTGCGCTCGTTGCGGGACTTAACCCAACATCTCACGACACGAGC
TGACGACAACCATGCACCACCTGTTTACGAGTGTCCAAAGAGTTGACCATTTCTGGCCCGTTCTCGTATATGTCAAGCCTTGGTAAGGTTCTTCGCGTTG
CATCGAATTAATCCGCATGCTCCGCCGCTTGTGCGGGTCCCCGTCAATTCCTTTGAGTTTTAGCCTTGCGGCCGTACTCCCCAGGCGGGGAACTTAATGC
GTTAGCTGCGTCACGGAATCCGTGGAATGGACCCCACAACTAGTTCCCAACGTTTACGGGGTGGACTACCAGGGTATCTAAGCCTGTTTGCTCCCCACCC
TTTCGCTCCTCAGCGTCAGTTACGGCCCAGAGATCTGCCTTCGCCATCGGTGTTCCTCCTGATATCTGCGCATTCCACCGCTACACCAGGAATTCCAATC
TCCCCTACCGCACTCTAGTCTGCCCGTACCCACTGCAGGCCGGAGGTTGAGCCTCCGGATTTCACAGCAGACGCGACAAACCGCCTACGAGCTCTTTACG
CCCAATAATTCCGGATAACGCTTGCGCCCTACGTATTACCGCGGCTGCTGGCACGTAGTTAGCCGGCGCTTTTTTCTGCAGGTACCGTCACTTTCGCTTC
TTCCCTGCTAAAAGAGGTTTACAACCCGAAGGCCGTCATCCCTCACGCGGCGTTGCTGCATCAGGCTTGCGCCCATTGTGCAATATTCCCCACTGCTGCC
TCCCGTAGGAGTCTGGGCCGTGTCTCAGTCCCAGTGTGGCCGGTCACCCTCTCAGGCCGGCTACCCGTCGACGCCTTGGTGAGCCATTACCTCACCAACA
AGCTGATAGGCCGCGAGCCCATCCCCAACCAATAAATCTTTCCAGTAACTGACCATGCGGTCGCTACTCGTATCCAGTATTAGACGCCGTTTCCAGCGCT
TATCCCAGAGTCAGGGGCAGGTTGCTCACGTGTTACTCACCCGTTCGCCACTGATCCCACAGAGCAAGCTCCGTGTTCACCGTTCGACTGCA

>EB224
GTCTGGGAAACTGCCTGATGGATGGGGATAACTACTGGAAACGGTAGCTAATACCGCAAAACGTCGCAGGACCCAAGTGGGGGACCTTCGGGCCTCATGC
CATCAGATGTGCCCAGATGGGATTAGCTAGTAGGTGGGGTAATGGCTCACCTAGGCGACGATCCCTAGCTGGTCTGAGAGGATGACCAGCCACACTGGAA

CTGAGACACGGTCCAGACTCCTACGGGAGGCAGCAGTGGGGAATATTGCACAATGGGCGCAAGCCTGATGCAGCCATGCCGCGTGTATGAAGAAGGCCTT
CGGGTTGTAAAGTACTTTCAGCGAGGAGGAAGGCGTTAAGGTTAATAACCTTRGCGATTGACGTTACTCGCAGAAGAAGCACCGGCTAACTCCGTGCCAG
CAGCCGCGGTAATACGGAGGGTGCAAGCGTTAATCGGAATTACTGGGCGTAAAGCGCACGCAGGCGGTCTGTTAAGTCAGATGTGAAATCCCCGGGCTCA
ACCTGGGAACTGCATTTGAAACTGGCAGGCYTGAGTCTTGTAGAGGGGGGTAGAATTCCAGGTGTAGCGGTGAAATGCGTAGAGATCTGGAGGAATACCG
GTGGCGAAGGCGGCCCCCTGGACAAAGACTGACGCTCAGGTGCGAAAGCGTGGGGAGCAAACAGGATTAGATACCCTGGTAGTCCACGCTGTAAACGATG
TCGACTTGGAGGTTGTTCCCTTGAGGAGTGGCTTCCGGAGCTAACGCGTTAAGTCGACCGCCTGGGGAGTACGGCCGCAAGGTTAAAACTCAAATGAATT
GACGGGGGCCCGCACAAGCGGTGGAGCATGTGGTTTAATTCGATGCAACGCGAAGAACCTTACCTACTCTTGACATCCAGAGAACTTAGCAGAGATGCTT
TGGTGCCTTCGGGAACTCTGAGACAGGTGCTGCATGGCTGTCGTCAGCTCGNNTGTTGTGAARTGTTGGGTTAAGTCCCGCAACGAGCGCAACCCTTATC
CTTTGTTGCCAGCGATTCGGTCGGGAACTCAAAGGAGACTGCCAGTGATAAACTGGAGGAAGGTGGGGATGACGTCAAGTCATCATGGCCCTTACGAGTA
GGGCTACACACGTGCTACAATGGCATATACAAAGAGAAGCGACCTCGCGAGAGCAAGCGGACCTCATAAAGTATGTCGTAGTCCGGATTGGAGTCTGCAA
CTCGACTCCATGAAGTCGGAATCGCTAGTAATCGTAGATCAGAATGCTACGGTGAATACGTTCCCGGGCCTTGTACACACCGCCCGTCACACCATGGGAG
TGGGCGCAAAAGAAGTAGGTAG

>EB225
TGCAGTCGAACGGTGAACACGGAGCTTGCTCTGTGGGATCAGTGGCGAACGGGTGAGTAACACGTGAGCAACCTGCCCCTGACTCTGGGATAAGCGCTGG
AAACGGCGTCTAATACTGGATATGTGACGTGATCGCATGGTCTGCGTCTGGAAAGAATTTCGGTTGGGGATGGGCTCGCGGCCTATCAGCTTGTTGGTGA
GGTAATGGCTCACCAAGGCGTCGACGGGTAGCCGGCCTGAGAGGGTGACCGGCCACACTGGGACTGAGACACGGCCCAGACTCCTACGGGAGGCAGCAGT
GGGGAATATTGCACAATGGGCGCAAGCCTGATGCAGCAACGCCGCGTGAGGGATGACGGCCTTCGGGTTGTAAACCTCTTTTAGCAGGGAAGAAGCGAAA
GTGACGGTACCTGCAGAAAAAAGCGCCGGCTAACTACGTGCCAGCAGCCGCGGTAATACGTAGGGCGCAAGCGTTATCCGGAATTATTGGGCGTAAAGAG
CTCGTAGGCGGTTTGTCGCGTCTGCTGTGAAATCCGGAGGCTCAACCTCCGGCCTGCAGTGGGTACGGGCAGACTAGAGTGCGGTAGGGGAGATTGGAAT
TCCTGGTGTAGCGGTGGAATGCGCAGATATCAGGAGGAACACCGATGGCGAAGGCAGATCTCTGGGCCGTAACTGACGCTGAGGAGCGAAAGGGTGGGGA
GCAAACAGGCTTAGATACCCTGGTAGTCCACCCCGTAAACGTTGGGAACTAGTTGTGGGGTCCATTCCACGGATTCCGTGACGCAGCTAACGCATTAAGT
TCCCCGCCTGGGGAGTACGGCCGCAAGGCTAAAACTCAAAGGAATTGACGGGGACCCGCACAAGCGGCGGAGCATGCGGATTAATTCGATGCAACGCGAA
GAACCTTACCAAGGCTTGACATATACGAGAACGGGCCAGAAATGGTCAACTCTTTGGACACTCGTAAACAGGTGGTGCATGGTTGTCGTCAGCTCGTGTC
GTGAGATGTTGGGTTAAGTCCCGCAACGAGCGCAACCCTCGTTCTATGTTGCCAGCACGTAATGGTGGGAACTCATGGGATACTGCCGGGGTCAACTCGG
AGGAAGGTGGGGATGACGTCAAATCATCATGCCCCTTATGTCTTGGGCTTCACGCATGCTACAATGGCCGGTACAAAGGGCTGCAATACCGCGAGGTGGA
GCGAATCCCAAAAAGCCGGTCCCAGTTCGGATTGAGGTCTGCAACTCGACCTCATGAAGTCGGAGTCGCTAGTAATCGCAGATCAGCAACGCTGCGGTGA
ATACGTTCCCGGGTCTTGTACACACCGCCCGTCAAGTCATGAAAGTCGGTAACACCTGAAGCCGGTGGCCTAACCCTTGTGGAGGGAGCCG

>EB226
GTTTGATCATGGCTCAGTAGTCGTAACAAGGTAACCGTAGAGTTTGATTATGGCTCAGAAGTCGTAACGAGGTAACCGTATAGTTTGATGATGGCTCACC
AGGACATAGCAGCGTATACGTAAAGTTTGATTGAACCGCAGGTTCAAGTTGAAAAGACGGTTTCGGCTGTCACTTACAGATGGGCCCGCGGCGCATTAAC
TAGTTGGTGAGGTAATGGCTCACCAAGGCAACGATGCGTAGCCGACCTGAGAGGGTGATCGGCCACACTGGGACTGRGACNNACGGCCCAGACTCCTACG
GGAGGCAGCAGTAGGGAATCTTCCGCAATGGACGAAAGTCTGACGGAGCAACGCCGCGTGAGTGATGAAGGTTTTCGGATCGTAAAACTCTGTTGTTAGG
GAAGANCAAGTGCGAGAGTAACTGCTCGCACCTTGACGGTACCTAACCAGAAAGCCACGGCTAACTACGTGCCAGCAGCCGCGGTAATACGTAGGTGGCA
AGCGTTGTCCGGAATTATTGGGCGTAAAGCGCGCGCAGGCGGTTTCTTAAGTCTGATGTGAAAGCCCCCGGCTCAACCGGGGAGGGTCATTGGAAACTGG
GAAACTTGAGTGCAGAAGAGGAGAGTGGAATTCCACGTGTAGCGGTGAAATGCGTAGAGATGTGGAGGAACACCAGTGGCGAAGGCGACTCTCTGGTCTG
TAACTGACGCTGAGGCGCGAAAGCGTGNGGAGCGAACAGGATTAGATACCCTGGTAGTCCACGCCGTAAACGATGAGTGCTAAGTTTAGAGGGTTTCCGC
CCTTTAGTGCTGCAGCTAACGCATTAAGCACTCCGCCTGGGGAGTACGGTCGCAAGACTGAAACTCAAAGGAATTGACGGGGGCCCGCACAAGCGGTGGA
GCATGTGGTTTAATTCGAAGCAACGCGAAGAACCTTACCAGGTCTTGACATCCTTTGCCACTTCTAGAGATAGAAGGTTCCCCTTCGGGGGACAAAGTGA
CAGGTGGTGCATGGTTGTCGTCAGCTCGTGTCGTGAGATGTTGGGTTAAGTCCCGCAACGAGCGCAACCCTTGATCTTAGTTGCCAGCATTCAGTTGGGC
ACTCTAAGGTGACTGCCGGTGACAAACCGGAGGAAGGTGGGGATGACGTCAAATCATCATGCCCCTTATGACCTGGGCTACACACGTGCTACAATGGATG
GTACAAAGGGCTGCGAGACCGCGAGGTTTAGCCAATCCCATAAAACCATTCTCAGTTCGGATTGCAGGCTGCAACTGGCCTGCATGAAGCGGGAATCGTT
GGTAATCGCGGATCAGCATGACGCGGTGA

>EB227
GGCTCCCTCCACAAGGGTTGGGCCACCGGCTTCAGGTGTTACCGACTTTCATGACTTGACGGGCGGTGTGTACAAGACCCGGGAACGTATTCACCGCAGC
GTTGCTGATCTGCGATTACTAGCGACTCCGACTTCATGAGGTCGAGTTGCAGACCTCAATCCGAACTGGGACCGGCTTTTTGGGATTCGCTCCACCTCAC
GGTATTGCAGCCCTTTGTACCGGCCATTGTAGCATGCGTGAAGCCCAAGACATAAGGGGCATGATGATTTGACGTCATCCCCACCTTCCTCCGAGTTGAC
CCCGGCAGTATCCCATGAGTTCCCACCATTACGTGCTGGCAACATAGAACGAGGGTTGCGCTCGTTGCGGGACTTAACCCAACATCTCACGACACGAGCT
GACGACAACCATGCACCACCTGTTCACCAGTGTCCAAAGAGTTGACCATTTCTGGCCCGTTCTGGTGTATGTCAAGCCTTGGTAAGGTTCTTCGCGTTGC
ATCGAATTAATCCGCATGCTCCGCCGCTTGTGCGGGTCCCCGTCAATTCCTTTGAGTTTTAGCCTTGCGGCCGTACTCCCCAGGCGGGGAACTTAATGCG
TTAGCTGCGTCACGGAAACCGTGGAATGGTCCCCACAACTAGTTCCCAACGTTTACGGGGTGGACTACCAGGGTATCTAAGCCTGTTTGCTCCCCACCCT
TTCGCTCCTCAGCGTCAGTTACGGCCCAGAGATCTGCCTTCGCCATCGGTGTTCCTCCTGATATCTGCGCATTCCACCGCTACACCAGGAATTCCAATCT
CCCCTACCGCACTCTAGTCTGCCCGTACCCACTGCAGGCCCGAGGTTGAGCCTCGGGATTTCACAGCAGACGCGACAAACCGCCTACGAGCTCTTTACGC
CCAATAATTCCGGATAACGCTTGCGCCCTACGTATTACCGCGGCTGCTGGCACGTAGTTAGCCGGCGCTTTTTCTGCAGGTACCGTCACTTTCGCTTCTT
CCCTGCTAAAAGAGGTTTACAACCCGAAGGCCGTCATCCCTCACGCGGCGTTGCTGCATCAGGCTTTCGCCCATTGTGCAATATTCCCCACTGCTGCCTC
CCGTAGGAGTCTGGGCCGTGTCTCAGTCCCAGTGTGGCCGGTCACCCTCTCAGGCCGGCTACCCGTCGACGCCTTGGTGAGCCATTACCTCACCAACAAG
CTGATAGGCCGCGAGCCCATCCCAGACCAAAAAATCTTTCCAACCCCCACCATGCGGAAGAGGCTCATATCCAGTATTAGACGCCGTTTCCAGCGCTTAT
CCCAGAGTCCAGGGCAGGTTGCTCACGTGTTACTCACCCGTTCGCCACTGATCCACCAAGCAAGCTTGGCTTCACCGTTCGACTGCA

>EB228
TACATGCAAGTCGAGCGGACAGAAGGGAGCTTGCTCCCGGATGTTAGCGGCGGACGGGTGAGTAACACGTGGGTAACCTGCCTGTAAGACTGGGATAACT
CCGGGAAACCGGAGCTAATACCGGATAGTTCCTTGAACCGCATGGTTCAAGGATGAAAGACGGTTTCGGCTGTCACTTACAGATGGACCCGCGGCGCATT
AGCTAGTTGGTGAGGTAACGGCTCACCAAGGCGACGATGCGTAGCCGACCTGAGAGGGTGATCGGCCACACTGGGACTGAGACACGGCCCAGACTCCTAC
GGGAGGCAGCAGTAGGGAATCTTCCGCAATGGACGAAAGTCTGACGGAGCAACGCCGCGTGAGTGATGAAGGTTTTCGGATCGTAAAGCTCTGTTGTTAG
GGAAGAACAAGTGCAAGAGTAACTGCTTGCACCTTGACGGTACCTAACCAGAAAGCCACGGCTAACTACGTGCCAGCAGCCGCGGTAATACGTAGGTGGC
AAGCGTTGTCCGGAATTATTGGGCGTAAAGGGCTCGCAGGCGGTTTCTTAAGTCTGATGTGAAAGCCCCCGGCTCAACCGGGGAGGGTCATTGGAAACTG
GGAAACTTGAGTGCAGAAGAGGAGAGTGGAATTCCACGTGTAGCGGTGAAATGCGTAGAGATGTGGAGGAACACCAGTGGCGAAGGCGACTCTCTGGTCT
GTAACTGACGCTGAGGAGCGAAAGCGTGGGGAGCGAACAGGATTAGATACCCTGGTAGTCCACGCCGTAAACGATGAGTGCTAAGTGTTAGGGGGTTTCC
GCCCCTTAGTGCTGCAGCTAACGCATTAAGCACTCCGCCTGGGGAGTACGGTCGCAAGACTGAAACTCAAAGGAATTGACGGGGGCCCGCACAAGCGGTG
GAGCATGTGGTTTAATTCGAAGCAACGCGAAGAACCTTACCAGGTCTTGACATCCTCTGACAACCCTAGAGATAGGGCTTTCCCTTCGGGGACAGAGTGA
CAGGTGGTGCATGGTTGTCGTCAGCTCGTGTCGTGAGATGTTGGGTTAAGTCCCGCAACGAGCGCAACCCTTGATCTTAGTTGCCAGCATTCAGTTGGGC
ACTCTAAGGTGACTGCCGGTGACAAACCGGAGGAAGGTGGGGATGACGTCAAATCATCATGCCCCTTATGACCTGGGCTACACACGTGCTACAATGGACA
GAACAAAGGGCTGCGAGACCGCAAGGTTTAGCCAATCCCACAAATCTGTTCTCAGTTCGGATCGCAGTCTGCAACTCGACTGCGTGAAGCTGGAATCGCT
AGTAATCGCGGATCAGCATGCCGCGGTGAATACGTTCCCGGGCCTTGTACACACCGCCCGTCACACCACGAGAGTTTGCAACACCCGAAGTCGGTGAGGT
AACCTTTATGGAGCCAGCCGCCGAAGGTGGGGCAGATGATTGGGGTGAAGTCG

>EB229
GTCGAACGGTGAAGGCGGAGCTTGCTCTGCTGGATCAGTGGCGAACGGGTGAGTAACACGTGAGCAATCTGCCCCTGACTCTGGGATAAGCGCTGGAAAC
GGCGTCTAATACCGGATACGAGCTGCGAAGGCATCTTCAGCAGCTGGAAAGAACTTCGGTCAGGGATGAGCTCGCGGCCTATCAGCTAGTTGGTGAGGTA
ACGGCTCACCAAGGCGTCGACGGGTAGCCGGCCTGAGAGGGTGACCGGCCACACTGGGACTGAGACACGGCCCAGACTCCTACGGGAGGCAGCAGTGGGG
AATATTGCACAATGGGCGCAAGCCTGATGCAGCAACGCCGCGTGAGGGACGACGGCCTTCGGGTTGTAAACCTCTTTTAGCAGGGAAGAAGCGAAAGTGA
CGGTACCTGCAGAAAAAGCGCCGGCTAACTACGTGCCAGCAGCCGCGGTAATACGTAGGGCGCAAGCGTTATCCGGAATTATTGGGCGTAAAGAGCTCGT
AGGCGGTTTGTCGCGTCTGCTGTGAAAACCCGAGGCTCAACCTCGGGCCTGCAGTGGGTACGGGCAGACTAGAGTGCGGTAGGGGAGATTGGAATTCCTG
GTGTAGCGGTGGAATGCGCAGATATCAGGAGGAACACCGATGGCGAAGGCAGATCTCTGGGCCGTAACTGACGCTGAGGAGCGAAAGGGTGGGGAGCAAA
CAGGCTTAGATACCCTGGTAGTCCACCCCGTAAACGTTGGGAACTAGTTGTGGGGACCATTCCACGGTCTCCGTGACGCAGCTAACGCATTAAGTTCCCC
GCCTGGGGAGTACGGCCGCAAGGCTAAAACTCAAAGGAATTGACGGGGACCCGCACAAGCGGCGGAGCATGCGGATTAATTCGATGCAACGCGAAGAACC
TTACCAAGGCTTGACATATACGAGAACGCTGCAGAAATGTAGAAACTCTTTGGACACTCGTAAACAGGTGGTGCATGGTTGTCGTCAGCTCGTGTCGTGA
GATGTTGGGTTAAGTCCCGCAACGAGCGCAACCCTCGTTCTATGTTGCCAGCACGTAATGGTGGGAACTCATGGGATACTGCCGGGGTCAACTCGGAGGA
AGGTGGGGATGACGTCAAATCATCATGCCCCTTATGTCTTGGGCTTCACGCATGCTACAATGGCCGGTACAAAGGGCTGCAATACCGCGAGGTGGAGCGA
ATCCCAAAAAGCCGGTCCCAGTTCGGATTGAGGTCTGCAACTCGACCTCATGAAGTCGGAGTCGCTAGTAATCGCAGATCAGCAACGCTGCGGTGAATAC
GTTCCCGGGTCTTGTACACACCGCCCGTCAAGTCATGAAAGTCGGTAACACCTGAAGCCGGTGGCCCAACCCTTGTGGAGGGAG

>EB230
ACGGCTCCCTCCACAAGGGTTGGGCCACCGGCTTCAGGTGTTACCGACTTTCATGACTTGACGGGCGGTGTGTACAAGACCCGGGAACGTATTCACCGCA
GCGTTGCTGATCTGCGATTACTAGCGACTCCGACTTCATGAGGTCGAGTTGCAGACCTCAATCCGAACTGGGACCGGCTTTTTGGGATTCGCTCCACCTC
ACGGTATTGCAGCCCTTTGTACCGGCCATTGTAGCATGCGTGAAGCCCAAGACATAAGGGGCATGATGATTTGACGTCATCCCCACCTTCCTCCGAGTTG
ACCCCGGCAGTATCCCATGAGTTCCCACCATTACGTGCTGGCAACATAGAACGAGGGTTGCGCTCGTTGCGGGACTTAACCCAACATCTCACGACACGAG
CTGACGACAACCATGCACCACCTGTTCACCAGTGTCCAAAGAGTTGACCATTTCTGGCCCGTTTCTGGTGTATGTCAAGCCTTGGTAAGGTTCTTCGCGT
TGCATCGAATTAATCCGCATGCTCCGCCGCTTGTGCGGGTCCCCGTCAATTCCTTTGAGTTTTAGCCTTGCGGCCGTACTCCCCCAGGCGGGGAACTTAA
TGCGTTAGCTGCGTCACGGAAACCGTGGAATGGTCCCCACAACTAGTTCCCAACGTTTACGGGGTGGACTACCAGGGTATCTAAGCCTGTTTGCTCCCCA
CCCTTTCGCTCCTCAGCGTCAGTTACGGCCCAGAGATCTGCCTTCGCCATCGGTGTTCCTCCTGATATCTGCGCATTCCACCGCTACACCAGGAATTCCA
ATCTCCCCTACCGCACTCTAGTCTGCCCGTACCCACTGCAGGCCCGAGGTTGAGCCTCGGGATTTCACAGCAGACGCGACAAACCGCCTACGAGCTCTTT
ACGCCCAATAATTCCGGATAACGCTTGCGCCCTACGTATTACCGCGGCTGCTGGCACGTAGTTAGCCGGCGCTTTTTCTGCAGGTACCGTCACTTTCGCT
TCTTCCCTGCTAAAAGAGGTTTACAACCCGAAGGCCGTCATCCCTCACGCGGCGTTGCTGCATCAGGCTTTCGCCCATTGTGCAATATTCCCCACTGCTG
CCTCCCGTAGGAGTCTGGGCCGTGTCTCAGTCCCAGTGTGGCCGGTCACCCTCTCAGGCCGGCTACCCGTCGACGCCTTGGTGAGCCATTACCTCACCAA
CAAGCTGATAGGCCGCGAGCCCATCCCAGACCAAAAAATCTTTCCAACCCCCACCATGCGATGAGAGCTCATATCCAGTATTAGACGCCGTTTCCAGCGC
TTATCCCAGAGTCCAGGGCAGGTTGCTCACGTGTTACTCACCCGTTCGCCACTGATCCACCAAGCAAGCTTGGCTTCACCGTTCGACTGCA

>EB231
TGCAGTCGAACGATGAAGCCCAGCTTGCTGGGTGGATTAGTGGCGAACGGGTGAGTAACACGTGAGTAACCTGCCCTTAACTCTGGGATAAGCCTGGGAA
ACTGGGTCTAATACCGGATAGGAGCGTCCACCGCATGGTGGGTGTTGGAAAGATTTATCGGTTTTGGATGGACTCGCGGCCTATCAGCTTGTTGGTGAGG
TAATGGCTCACCAAGGCGACGACGGGTAGCCGGCCTGAGAGGGTGACCGGCCACACTGGGACTGAGACACGGCCCAGACTCCTACGGGAGGCAGCAGTGG
GGAATATTGCACAATGGGCGAAAGCCTGATGCAGCGACGCCGCGTGAGGGATGACGGCCTTCGGGTTGTAAACCTCTTTCAGTAGGGAAGAAGCGAAAGT
GACGGTACCTGCAGAAGAAGCACCGGCTAACTACGTGCCAGCAGCCGCGGTAATACGTAGGGTGCGAGCGTTATCCGGAATTATTGGGCGTAAAGAGCTC
GTAGGCGGTTTGTCGCGTCTGTCGTGAAAGTCCGGGGCTTAACCCCGGATCTGCGGTGGGTACGGGCAGACTAGAGTGCAGTAGGGGAGACTGGAATTCC
TGGTGTAGCGGTGGAATGCGCAGATATCAGGAGGAACACCGATGGCGAAGGCAGGTCTCTGGGCTGTAACTGACGCTGAGGAGCGAAAGCATGGGGAGCG
AACAGGATTAGATACCCTGGTAGTCCATGCCGTAAACGTTGGGCACTAGGTGTGGGGGACCATTCCACGGTTTCCGCGCCGCAGCTAACGCATTAAGTGC
CCCGCCTGGGGGAGTACGGCCGCAAGGCTAAAACTCAAAGGAAATTGACGGGGGCCCGCACAAGCGGCGGAGCATGCGGATTAATTCGATGCAACGCGAA
GAACCTTACCAAGGCTTGACATGTTCTCGATCGCCGTGGAGATACGGTTTCCCCTTTGGGGCGGGTTCACAGGTGGTGCATGGTTGTCGTCAGCTCGTGT
CGTGAGATGTTGGGTTAAGTCCCGCAACGAGCGCAACCCTCGTTCCATGTTGCCAGCACGTAGTGGTGGGGACTCATGGGAGACTGCCGGGGTCAACTCG
GAGGAAGGTGAGGACGACGTCAAATCATCATGCCCCTTATGTCTTGGGCTTCACGCATGCTACAATGGCCGGTACAATGGGTTGCGATACTGTGAGGTGG
AGCTAATCCCAAAAAGCCGGTCTCAGTTCGGATTGGGGTCTGCAACTCGACCCCATGAAGTCGGAGTCGCTAGTAATCGCAGATCAGCAACGCTGCGGTG
AATACGTTCCCGGGCCTTGTACACACCGCCCGTCAAGTCACGAAAGTCGGTAACACCCGAAGCCGGGGCCTAACCC

>EB232
TGCAGTCGAGCGAATGATGAAAAAGCTTGCTTCTTCTGATTTACCGGCGGACGGGTGAGTAACACGTGGGCAACCTGCCCTGTAGATTGGGATAACTCCG
GGAAACCGGGGCTAATACCGAATAATCCATTTCCTCGCATGGAGGAATGTTAAAAGACGGTTTCGGCTGTCACTACTGGATGGGCCCGCGGCGCACTAGC
TAGTTGGTGAGGTAACGGCTCACCAAGGCGACCATGCGTAACCTACCTGAGAGGGTGATCGGCCACACTGGGACTGAGACACGGCCCASAYTCCTACGGG
AGGCAGCAGTAGGGAATCTTCCCCAATGGACGAAAGTCTGATGGAGCAACGCCGCGTGAGTGAAGAAGGTTTTCGGATCGTAAAACTCTGTTGTGAGGGA
AGAACAAGTACGAGAGTAACTGCTCGTACCTTGACGGTACCTCATTAGAAAGCCACGGCTAAYTACGTGCCAGCAGCCGCGGTAATACGTAGGTGGCAAG
CGTTGTCCGGAATTATTGGGCGTAAAGCGCGCGCAGGCGGTCCTTTAAGTCTGATGTGAAAGCCCACGGCTCAACCGTGGAGGGTCATTGGAAACTGGGG
GACTTGAGTGCAGAARAGGAAAGTGGAATTCCAAGTGTAGCGGTGAAATGCGTAKAGATTTGGAGGAACACCAGTGGCGAAGGCGACTTTCTGGTCTGTA
ACTGACGCTGAGGCGCGAAAGCGTGGGGAGCAAACAGGATTAGATACCCTGGTAGTCCACGCCGTAAACGATGAGTGCTAAGTGTTAGGGGGTTTCCGCC
CCTTAGTGCTGCAGCTAACGCATTAAGCACTCCGCCTGGGGAGTACGGTCGCAAGACTGAAACTCAAAGGAATTGACGGGGGCCCGCANCAAGCGGTGNG
AGCATKTGGKYWAATTCGAAGCAACGCGAAGAACCTTACCAGGTCTTGACATMCCGMTTWCCKTCCKAGAGATAGGATTTTCCCTTCGGGGACAGCGGTG
ACAGGTGGTGCATGGTTGTCGTCAGCTCGTGTCGTGAGATGTTGGGTTAAGTCCCGCAACGAGCGCAACCCTTGATCTTAGTTGCCAGCATTCAGTTGGG
CACTCTAAGGTGACTGCCGGTGATAAACCGGAGGAAGGTGGGGATGACGTCAAATCATCATGCCCCTTATGACCTGGGCTACACACGTGCTACAATGGAC
GGTACAGAGGGTCGCAACCCCGCGAGGGTGAGCTAATCCCATAAAACCGTTCTCAGTTCGGATTGTAGGCTGCAACTCGCCTACATGAAGCCGGAATCGC
TAGTAATCGTGGATCAGCATGCCACGGTGAATACGTTCCCGGGCCTTGTACACACCGCCCGTCACACCACGAGAGTTTGTAACACCCGAAGTCGGTGGGG
TAACCCTTTTGGGAGCCAGCCG

>EB233
GCAGTCGAACGATGAAGCCCAGCTTGCTGGGTGGATTAGTGGCGAACGGGTGAGTAACACGTGAGTAACCTGCCCTTAACTCTGGGATAAGCCTGGGAAA
CTGGGTCTAATACCGGATAGGAGCGCCGACCGCATGGTGGGTGTTGGAAAGATTTATCGGTTTTGGATGGACTCGCGGCCTATCAGCTTGTTGGTGAGGT
AATGGCTCACCAAGGCGACGACGGGTAGCCGGCCTGAGAGGGTGACCGGCCACACTGGGACTGAGACACGGCCCAGACTCCTACGGGAGGCAGCAGTGGG
GAATATTGCACAATGGGCGCAAGCCTGATGCAGCGACGCCGCGTGAGGGATGACGGCCTTCGGGTTGTAAACCTCTTTCAGTAGGGAAGAAGCGAAAGTG
ACGGTACCTGCAGAAGAAGCACCGGCTAACTACGTGCCAGCAGCCGCGGTAATACGTAGGGTGCGAGCGTTATCCGGAATTATTGGGCGTAAAGAGCTCG
TAGGCGGTTTGTCGCGTCTGTCGTGAAAGTCCGGGGCTTAACCCCGGATCTGCGGTGGGTACGGGCAGACTAGAGTGCAGTAGGGGAGACTGGAATTCCT
GGTGTAGCGGTGGAATGCGCAGATATCAGGAGGAACACCGATGGCGAAGGCAGGTCTCTGGGCTGTAACTGACGCTGAGGAGCGAAAGCATGGGGAGCGA
ACAGGATTAGATACCCTGGTAGTCCATGCCGTAAACGTTGGGCACTAGGTGTGGGGACCATTCCACGGTTTCCGCGCCGCAGCTAACGCATTAAGTGCCC
CGCCTGGGGAGTACGGCCGCAAGGCTAAAACTCAAAGGAATTGACGGGGGCCCGCACAAGCGGCGGAGCATGCGGATTAATTCGATGCAACGCGAAGAA
CCTTACCAAGGCTTGACATGTTCTCGATCGCCGTAGAGATACGGTTTCCCCTTTGGGGCGGGTTCACAGGTGGTGCATGGGTTGTCGTCAGCTCGTGTCG
TGAGATGTTGGGTTAAGTCCCGCAACGAGCGCAACCCTCGTTCCATGTTGCCAGCACGTAATGGTGGGGACTCATGGGAGACTGCCGGGGTCAACTCGGA
GGAAGGTGAGGACGACGTCAAATCATCATGCCCCTTATGTCTTGGGCTTCACGCATGCTACAATGGCCGGTACAATGGGTTGCGATACTGTGAGGTGGAG

CTAATCCCAAAAAGCCGGTCTCAGTTCGGATTGGGGTCTGCAACTCGACCCCATGAAGTCGGAGTCGCTAGTAATCGCAGATCAGCAACGCTGCGGTGAA
TACGTTCCCGGGCCTTGTACACACCGCCCGTCAAGTCACGAAAGTCGGTAACACCCGAAGCCGGTGGCCTAACCCTTG

>EB234
GGTTAGGCCACCGGCTTCGGAGTGTTACCAACTTTCGTGACTTGACGGGCGGTGTGTACAAGGCCCGGGAACGTATTCACCGCAGCGTTGCTGATCTGCG
ATTACTAGCGACTCCGACTTCATGGGGTCGAGTTGCAGACCCCAATCCGAACTGAGACCGGCTTTTTGGGATTAGCTCCACCTCACAGTATCGCAACCCA
TTGTACCGGCCATTGTAGCATGCGTGAAGCCCAAGACATAAGGGGCATGATGATTTGACGTCGTCCTCACCTTCCTCCGAGTTGACCCCGGCAGTCTCCC
ATGAGTCCCCACCATTACGTGCTGGCAACATGGAACGAGGGTTGCGCTCGTTGCGGGACTTAACCCAACATCTCACGACACGAGCTGACGACAACCATGC
ACCACCTGTGAACCCGCCCCAAAGGGGAAAACCGTATCTCTACGGCGATCGAGAACATGTCAAGCCTTGGTAAGGTTCTTCGCGTTGCATCGAAATTAAT
CCGCATGCTCCGCCGCTTGTGCGGGCCCCCGTCAATTCCTTTGAGTTTTAGCCTTGCGGCCGTACTCCCCAGGCGGGGCACTTAATGCGTTAGCTGCGGC
GCGGAAACCGTGGAATGGTCCCCACACCTAGTGCCCAACGTTTACGGCATGGACTACCAGGGTATCTAATCCTGTTCGCTCCCCATGCTTTCGCTCCTCA
GCGTCAGTTACAGCCCAGAGACCTGCCTTCGCCATCGGTGTTCCTCCTGATATCTGCGCATTCCACCGCTACACCAGGAATTCCAGTCTCCCCTACTGCA
CTCTAGTCTGCCCGTACCCACCGCAGATCCGGGGTTAAGCCCCGGACTTTCACGACAGACGCGACAAACCGCCTACGAGCTCTTTACGCCCAATAATTCC
GGATAACGCTCGCACCCTACGTATTACCGCGGCTGCTGGCACGTAGTTAGCCGGTGCTTCTTCTGCAGGTACCGTCACTTTCGCTTCTTCCCTACTGAAA
GAGGTTTACAACCCGAAGGCCGTCATCCCTCACGCGGCGTCGCTGCATCAGGCTTTCGCCCATTGTGCAATATTCCCCACTGCTGCCTCCCGTAGGAGTC
TGGGCCGTGTCTCAGTCCCAGTGTGGCCGGTCACCCTCTCAGGCCGGCTACCCGTCGTCGCCTTGGTGAGCCATTACCTCACCAACAAGCTGATAGGCCG
CGAGTCCATCCAAAACCGATAAATCTTTCCAACACCCACCATGCGGTGGACGCTCCTATCCGGTATTAGACCCAGTTTCCCAGGCTTATCCCAGAGTTAA
GGGCAGGTTACTCACGTGTTACTCACCCGTTCGCCACTAATCCACCCAGCAAGCTGGGCTTCATCGTTCGACTGC

>EB235
GGCTCCCCCACAAGGGTTAGGCCACCGGCTTCGGAGTAGATTACCGACTTTCGTGACTTGACGGGCGGTGTGTACAAGGCCCGGGAACGTATTCACCGCA
GCGTTGCTGATCTGCGATTACTAGCGACTCCGACTTCATGGGGTCGAGTTGCAGACCCCAATCCGAACTGAGACCGGCTTTTTGGGATTAGCTCCACCTC
ACAGTATCGCAACCCATTGTACCGGCCATTGTAGCATGCGTGAAGCCCAAGACATAAGGGGCATGATGATTTGACGTCGTCCTCACCTTCCTCCGAGTTG
ACCCCGGCAGTCTCCCATGAGTCCCCACCACGACGTGCTGGCAACATGGAACGAGGGTTGCGCTCGTTGCGGGACTTAACCCAACATCTCACGACACGAG
CTGACGACAACCATGCACCACCTGTGAACCCGCCCCAAAGGGGAAACCGTATCTCTACGGCGATCGAGAACATGTCAAGCCTTGGTAAGGTTCTTCGCGT
TGCATCGAATTAATCCGCATGCTCCGCCGCTTGTGCGGGCCCCCGTCAATTCCTTTGAGTTTTAGCCTTGCGGCCGTACTCCCCAGGCGGGGCACTTAAT
GCGTTAGCTGCGGCGCGGAAACCGTGGAATGGTCCCCACACCTAGTGCCCAACGTTTACGGCATGGACTACCAGGGTATCTAATCCTGTTCGCTCCCCAT
GCTTTCGCTCCTCAGCGTCAGTTACAGCCCAGAGACCTGCCTTCGCCATCGGTGTTCCTCCTGATATCTGCGCATTCCACCGCTACACCAGGAATTCCAG
TCTCCCCTACTGCACTCTAGTCTGCCCGTACCCACCGCAGATCCGGGGTTAAGCCCCGGACTTTCACGACAGACGCGACAAACCGCCTACGAGCTCTTTA
CGCCCAATAATTCCGGATAACGCTCGCACCCTACGTATTACCGCGGCTGCTGGCACGTAGTTAGCCGGTGCTTCTTCTGCAGGTACCGTCACTTTCGCTT
CTTCCCTACTGAAAGAGGTTTACAACCCGAAGGCCGTCATCCCTCACGCGGCGTCGCTGCATCAGGCTTGCGCCCATTGTGCAATATTCCCCACTGCTGC
CTCCCGTAGGAGTCTGGGCCGTGTCTCAGTCCCAGTGTGGCCGGTCACCCTCTCAGGCCGGCTACCCGTCGTCGCCTTGGTGAGCCATTACCTCACCAAC
AAGCTGATAGGCCGCGAGTCCATCCAAAACCGATAAATCTTTCCAACACCCACCATGCGGTGGACGCTCCTATCCGGTATTAGACCCAGTTTCCCAGGCT
TATCCCAGAGTTAAGGGCAGGTTACTCACGTGTTACTCACCCGTTCGCCACTAATCCACCCAGCAAGCTGGGCTTCATCGTTCGACTGCA

>EB236
ACGGCTCCCCCCACAAGGGTTAGGCCACCGGCTTCGGGTGTTACCAGAACTTTCGTGACTTGACGGGCGGTGTGTACAAGGCCCGGGAACGTATTCACCG
CAGCGTTGCTGATCTGCGATTACTAGCGACTCCGACTTCATGGGGTCGAGTTGCAGACCCCAATCCGAACTGAGACCGGCTTTTTGGGATTAGCTCCACC
TCACAGTATCGCAACCCATTGTACCGGCCATTGTAGCATGCGTGAAGCCCAAGACATAAGGGGCATGATGATTTGACGTCGTCCTCACCTTCCTCCGAGT
TGACCCCGGCAGTCTCCCATGAGTCCCCACCACTACGTGCTGGCAACATGGAACGAGGGTTGCGCTCGTTGCGGGACTTAACCCAACATCTCACGACACG
AGCTGACGACAACCATGCACCACCTGTGAACCCGCCCCAAAGGGGAAACCGTATCTCTACGGCGATCGAGAACATGTCAAGCCTTGGTAAGGTTCTTCGC
GTTGCATCGAATTAATCCGCATGCTCCGCCGCTTGTGCGGGCCCCCGTCAATTCCTTTGAGTTTTAGCCTTGCGGCCGTACTCCCCAGGCGGGGCACTTA
ATGCGTTAGCTGCGGCGCGGAAACCGTGGAATGGTCCCCACACCTAGTGCCCAACGTTTACGGCATGGACTACCAGGGTATCTAATCCTGTTCGCTCCCC
ATGCTTTCGCTCCTCAGCGTCAGTTACAGCCCAGAGACCTGCCTTCGCCATCGGTGTTCCTCCTGATATCTGCGCATTCCACCGCTACACCAGGAATTCC
AGTCTCCCCTACTGCACTCTAGTCTGCCCGTACCCACCGCAGATCCGGGGTTAAGCCCCGGACTTTCACGACAGACGCGACAAACCGCCTACGAGCTCTT
TACGCCCAATAATTCCGGATAACGCTCGCACCCTACGTATTACCGCGGCTGCTGGCACGTAGTTAGCCGGTGCTTCTTCTGCAGGTACCGTCACTTTCGC
TTCTTCCCTACTGAAAGAGGTTTACAACCCGAAGGCCGTCATCCCTCACGCGGCGTCGCTGCATCAGGCTTGCGCCCATTGTGCAATATTCCCCACTGCT
GCCTCCCGTAGGAGTCTGGGCCGTGTCTCAGTCCCAGTGTGGCCGGTCACCCTCTCAGGCCGGCTACCCGTCGTCGCCTTGGTGAGCCATTACCTCACCA
ACAAGCTGATAGGCCGCGAGTCCATCCAAAACCGATAAATCTTTCCAACACCCACCATGCGGTGGGCGCTCCTATCCGGTATTAGACCCAGTTTCCCAGG
CTTATCCCAGAGTTAAGGGCAGGTTACTCACGTGTTACTCACCCGTTCGCCACTAATCCACCCAGCAAGCTGGGCTTCATCGTTCG

>EB237
CAAGGGTTAGGCCACCGGCTTCGGGTGTTACCAACTTTCGTGACTTGACGGGCGGTGTGTACAAGGCCCGGGAACGTATTCACCGCAGCGTTGCTGATCT
GCGATTACTAGCGACTCCGACTTCATGGGGTCGAGTTGCAGACCCCAATCCGAACTGAGACCGGCTTTTTGGGATTAGCTCCACCTCACAGTATCGCAAC
CCATTGTACCGGCCATTGTAGCATGCGTGAAGCCCAAGACATAAGGGGCATGATGATTTGACGTCGTCCTCACCTTCCTCCGAGTTGACCCCGGCAGTCT
CCCATGAGTCCCCACCACGACGCGCCTGGCAACATGGAACGAGGGTTGCGCTCGTTGCGGGACTTAACCCAACATCTCACGACACGAGCTGACGACAACC
ATGCACCACCTGTGAACCCGCCCCCAAAGGGGAAACCGTATCTCTACGGCGATCGAGAACATGTCAAGCCTTGGTAAGGTTCTTCGCGTTGCATCGAATT
AATCCGCATGCTCCGCCGCTTGTGCGGGCCCCCGTCAATTCCTTTGAGTTTTAGCCTTGCGGCCGTACTCCCCAGGCGGGGCACTTAATGCGTTAGCTGC
GGCGCGGAAACCGTGGAATGGTCCCCACACCTAGTGCCCAACGTTTACGGCATGGACTACCAGGGTATCTAATCCTGTTCGCTCCCCATGCTTTCGCTCC
TCAGCGTCAGTTACAGCCCAGAGACCTGCCTTCGCCATCGGTGTTCCTCCTGATATCTGCGCATTCCACCGCTACACCAGGAATTCCAGTCTCCCCTACT
GCACTCTAGTCTGCCCGTACCCACCGCAGATCCGGGGTTAAGCCCCGGACTTTCACGACAGACGCGACAAACCGCCTACGAGCTCTTTACGCCCAATAAT
TCCGGATAACGCTCGCACCCTACGTATTACCGCGGCTGCTGGCACGTAGTTAGCCGGTGCTTCTTCTGCAGGTACCGTCACTTTCGCTTCTTCCCTACTG
AAAGAGGTTTACAACCCGAAGGCCGTCATCCCTCACGCGGCGTCGCTGCATCAGGCTTTCGCCCATTGTGCAATATTCCCCACTGCTGCCTCCCGTAGGA
GTCTGGGCCGTGTCTCAGTCCCAGTGTGGCCGGTCACCCTCTCAGGCCGGCTACCCGTCGTCGCCTTGGTGAGCCATTACCTCACCAACAAGCTGATAGG
CCGCGAGTCCATCCAAAACCGATAAATCTTTCCAACACCCACCATGCGGTGGACGCTCCTATCCGGTATTAGACCCAGTTTCCCAGGCTTATCCCAGAGT
TAAGGGCAGGTTACTCACGTGTTACTCACCCGTTCGCCACTAATCCACCCAGCAAGCTGGGCTTCATCGTTCGACTTGCA

>EB238
TGCAGTCGAACGATGAAGCCCAGCTTGCTGGGTGGATTAGTGGCGAACGGGTGAGTAACACGTGAGTAACCTGCCCTTAACTCTGGGATAAGCCTGGGAA
ACTGGGTCTAATACCGGATAGGAGCGCCCACCGCATGGTGGGTGTTGGAAAGATTTATCGGTTTTGGATGGACTCGCGGCCTATCAGCTTGTTGGTGAGG
TAATGGCTCACCAAGGCGACGACGGGTAGCCGGCCTGAGAGGGTGACCGGCCACACTGGGACTGAGACACGGCCCAGACTCCTACGGGAGGCAGCAGTGG
GGAATATTGCACAATGGGCGAAAGCCTGATGCAGCGACGCCGCGTGAGGGATGACGGCCTTCGGGTTGTAAACCTCTTTCAGTAGGGAAGAAGCGAAAGT
GACGGTACCTGCAGAAGAAGCACCGGCTAACTACGTGCCAGCAGCCGCGGTAATACGTAGGGTGCGAGCGTTATCCGGAATTATTGGGCGTAAAGAGCTC
GTAGGCGGTTTGTCGCGTCTGTCGTGAAAGTCCGGGGCTTAACCCCGGATCTGCGGTGGGTACGGGCAGACTAGAGTGCAGTAGGGGAGACTGGAATTCC
TGGTGTAGCGGTGGAATGCGCAGATATCAGGAGGAACACCGATGGCGAAGGCAGGTCTCTGGGCTGTAACTGACGCTGAGGAGCGAAAGCATGGGGAGCG
AACAGGATTAGATACCCTGGTAGTCCATGCCGTAAACGTTGGGCACTAGGTGTGGGGACCATTCCACGGTTTCCGCGCCGCAGCTAACGCATTAAGTGCC
CCGCCTGGGGAGTACGGCCGCAAGGCTAAAACTCAAAGGAATTGACGGGGGCCCGCACAAGCGGCGGAGCATGCGGATTAATTCGATGCAACGCGAAGAA
CCTTACCAAGGCTTGACATGTTCTCGATCGCCGTAGAGATACGGTTTCCCCTTTGGGGCGGGTTCACAGGTGGTGCATGGTTGTCGTCAGCTCGTGTCGT

GAGATGTTGGGTTAAGTCCCGCAACGAGCGCAACCCTCGTTCCATGTTGCCAGCACGTAATGGTGGGGACTCATGGGAGACTGCCGGGGTCAACTCGGAG
GAAGGTGAGGACGACGTCAAATCATCATGCCCCTTATGTCTTGGGCTTCACGCATGCTACAATGGCCGGTACAATGGGTTGCGATACTGTGAGGTGGAGC
TAATCCCAAAAAGCCGGTCTCAGTTCGGATTGGGGTCTGCAACTCGACCCCATGAAGTCGGAGTCGCTAGTAATCGCAGATCAGCAACGCTGCGGTGAAT
ACGTTCCCGGGCCTTGTACACACCGCCCGTCAAGTCACGAAAGTTGGTAACACCCGAAGCCGGTGGCCTAACCCTTG

>EB239
CAAGGGTTAGGCCACCGGCTTCGGGTGTTACCAACTTTCGTGACTTGACGGGCGGTGTGTACAAGGCCCGGGAACGTATTCACCGCAGCGTTGCTGATCT
GCGATTACTAGCGACTCCGACTTCATGGGGTCGAGTTGCAGACCCCAATCCGAACTGAGACCGGCTTTTTGGGATTAGCTCCACCTCACAGTATCGCAAC
CCATTGTACCGGCCATTGTAGCATGCGTGAAGCCCAAGACATAAGGGGCATGATGATTTGACGTCGTCCTCACCTTCCTCCGAGTTGACCCCGGCAGTCT
CCCATGAGTCCCCACCATTACGTGCTGGCAACATGGAACGAGGGTTGCGCTCGTTGCGGGACTTAACCCAACATCTCACGACACGAGCTGACGACAACCA
TGCACCACCTGTGAACCCGCCCCAAAGGGGAAACCGTATCTCTACGGCGATCGAGAACATGTCAAGCCTTGGTAAGGTTCTTCGCGTTGCATCGAATTAA
TCCGCATGCTCCGCCGCTTGTGCGGGCCCCCGTCAATTCCTTTGAGTTTTAGCCTTGCGGCCGTACTCCCCAGGCGGGGCACTTAATGCGTTAGCTGCGG
CGCGGAAACCGTGGAATGGTCCCCACACCTAGTGCCCAACGTTTACGGCATGGACTACCAGGGTATCTAATCCTGTTCGCTCCCCATGCTTTCGCTCCTC
AGCGTCAGTTACAGCCCAGAGACCTGCCTTCGCCATCGGTGTTCCTCCTGATATCTGCGCATTCCACCGCTACACCAGGAATTCCAGTCTCCCCTACTGC
ACTCTAGTCTGCCCGTACCCACCGCAGATCCGGGGTTAAGCCCCGGACTTTCACGACAGACGCGACAAACCGCCTACGAGCTCTTTACGCCCAATAATTC
CGGATAACGCTCGCACCCTACGTATTACCGCGGCTGCTGGCACGTAGTTAGCCGGTGCTTCTTCTGCAGGTACCGTCACTTTCGCTTCTTCCCTACTGAA
AGAGGTTTACAACCCGAAGGCCGTCATCCCTCACGCGGCGTCGCTGCATCAGGCTTTCGCCCATTGTGCAATATTCCCCACTGCTGCCTCCCGTAGGAGT
CTGGGCCGTGTCTCAGTCCCAGTGTGGCCGGTCACCCTCTCAGGCCGGCTACCCGTCGTCGCCTTGGTGAGCCATTACCTCACCAACAAGCTGATAGGCC
GCGAGTCCATCCAAAACCGATAAATCTTTCCAACACCCACCATGCGGTAGGCGCTCCTATCCGGTATTAGACCCAGTTTCCCAGGCTTATCCCAGAGTTA
AGGGCAGGTTACTCACGTGTTACTCACCCGTTCGCCACTAATCCACCCAGCAAGCTGGGCTTCATCGTTCGACTGCA

>EB240
TGCAAGTCGAACGATGAAGCCCAGCTTGCTGGGTGGATTAGTGGCGAACGGGTGAGTAACACGTGAGTAACCTGCCCTTAACTCTGGGATAAGCCTGGGA
AACTGGGTCTAATACCGGATAGGAGCGTCCACCGCATGGTGGGTGTTGGAAAGATTTATCGGTTTTGGATGGACTCGCGGCCTATCAGCTTGTTGGTGAG
GTAATGGCTCACCAAGGCGACGACGGGTAGCCGGCCTGAGAGGGTGACCGGCCACACTGGGACTGAGACACGGCCCAGACTCCTACGGGAGGCAGCAGTG
GGGAATATTGCACAATGGGCGCAAGCCTGATGCAGCGACGCCGCGTGAGGGATGACGGCCTTCGGGTTGTAAACCTCTTTCAGTAGGGAAGAAGCGAAAG
TGACGGTACCTGCAGAAGAAGCACCGGCTAACTACGTGCCCAGCAGCCGCGGTAATACGTAGGGTGCGAGCGTTATCCGGAATTATTGGGCGTAAAGAGC
TCGTAGGCGGTTTGTCGCGTCTGTCGTGAAAGTCCGGGGCTTAACCCCGGATCTGCGGTGGGTACGGGCAGACTAGAGTGCAGTAGGGGAGACTGGAATT
CCTGGTGTAGCGGTGGAATGCGCAGATATCAGGAGGAACACCGATGGCGAAGGCAGGTCTCTGGGCTGTAACTGACGCTGAGGAGCGAAAGCATGGGGAG
CGAACAGGATTAGATACCCTGGTAGTCCATGCCGTAAACGTTGGGCACTAGGTGTGGGGACCATTCCACGGTTTCCGCGCCGCAGCTAACGCATTAAGTG
CCCCGCCTGGGGAGTACGGCCGCAAGGCTAAAACTCAAAGGAATTGACGGGGGCCCGCACAAGCGGCGGAGCATGCGGATTAATTCGATGCAACGCGAA
GAACCTTACCAAGGCTTGACATGTTCTCGATCGCCGTAGAGATACGGTTTCCCCTTTGGGGCGGGTTCACAGGTGGTGCATGGTTGTCGTCAGCTCGTGT
CGTGAGATGTTGGGTTAAGTCCCGCAACGAGCGCAACCCTCGTTCCATGTTGCCAGCACGTCGTGGTGGGGACTCATGGGAGACTGCCGGGGTCAACTCG
GAGGAAGGTGAGGACGACGTCAAATCATCATGCCCCTTATGTCTTGGGCTTCACGCATGCTACAATGGCCGGTACAATGGGTTGCGATACTGTGAGGTGG
AGCTAATCCCAAAAAGCCGGTCTCAGTTCGGATTGGGGTCTGCAACTCGACCCCATGAAGTCGGAGTCGCTAGTAATCGCAGATCAGCAACGCTGCGGTG
AATACGTTCCCGGGCCTTGTACACACCGCCCGTCAAGTCACGAAAGTTTGGTAACACCCGAAGCCGGTGGCCTAACCCTTGTGGGGGGAGCC

>EB241
GCAGTCGAACGATGAAGCCCAGCTTGCTGGGTGGATTAGTGGCGAACGGGTGAGTAACACGTGAGTAACCTGCCCTTAACTCTGGGATAAGCCTGGGAAA
CTGGGTCTAATACCGGATAGGAGCGTCCACCGCATGGTGGGTGTTGGAAAGATTTATCGGTTTTGGATGGACTCGCGGCCTATCAGCTTGTTGGTGAGGT
AATGGCTCACCAAGGCGACGACGGGTAGCCGGCCTGAGAGGGTGACCGGCCACACTGGGACTGAGACACGGCCCAGACTCCTACGGGAGGCAGCAGTGGG
GAATATTGCACAATGGGCGAAAGCCTGATGCAGCGACGCCGCGTGAGGGATGACGGCCTTCGGGTTGTAAACCTCTTTCAGTAGGGAAGAAGCGAAAGTG
ACGGTACCTGCAGAAGAAGCACCGGCTAACTACGTGCCAGCAGCCGCGGTAATACGTAGGGTGCGAGCGTTATCCGGAATTATTGGGCGTAAAGAGCTCG
TAGGCGGTTTGTCGCGTCTGTCGTGAAAGTCCGGGGCTTAACCCCGGATCTGCGGTGGGTACGGGCAGACTAGAGTGCAGTAGGGGAGACTGGAATTCCT
GGTGTAGCGGTGGAATGCGCAGATATCAGGAGGAACACCGATGGCGAAGGCAGGTCTCTGGGCTGTAACTGACGCTGAGGAGCGAAAGCATGGGGAGCGA
ACAGGATTAGATACCCTGGTAGTCCATGCCGTAAACGTTGGGCACTAGGTGTGGGGACCATTCCACGGTTTCCGCGCCGCAGCTAACGCATTAAGTGCCC
CGCCTGGGGAGTACGGCCGCAAGGCTAAAACTCAAAGGAATTGACGGGGGCCCGCACAAGCGGCGGAGCATGCGGATTAATTCGATGCAACGCGAAGAAC
CTTACCAAGGCTTGACATGTTCTCGATCGCCGTAGAGATACGGTTTCCCCTTTGGGGCGGGTTCACAGGTGGTGCATGGTTGTCGTCAGCTCGTGTCGTG
AGATGTTGGGTTAAGTCCCGCAACGAGCGCAACCCTCGTTCCATGTTGCCAGCACGTAGTGGTGGGGACTCATGGGAGACTGCCGGGGTCAACTCGGAGG
AAGGTGAGGACGACGTCAAATCATCATGCCCCTTATGTCTTGGGCTTCACGCATGCTACAATGGCCGGTACAATGGGTTGCGATACTGTGAGGTGGAGCT
AATCCCAAAAAGCCGGTCTCAGTTCGGATTGGGGTCTGCAACTCGACCCCATGAAGTCGGAGTCGCTAGTAATCGCAGATCAGCAACGCTGCGGTGAATA
CGTTCCCGGGCCTTGTACACACCGCCCGTCAAGTCACGAAAGTCTGGTAACACTCCGAAGCCGGTGGCCTAA

>EB242
TCGCCTGCCTCCTTGCGGTTGGCGCAGCGCCGTCGGGTAAGACCAACTCCCATGGTGTGACGGGCGGTGTGTACAAGGCCCGGGAACGTATTCACCGTGG
CGTTCTGATCCACGATTACTAGCGATTCCGCCTTCATGCACTCGAGTTGCAGAGTGCAATCCGAACTGAGACGGCTTTTTAGGATTAGCTCCCCCTCGCG
GGTTCGCTGCCCATTGTCACCGCCATTGTAGCACGTGTGTAGCCCAGCCCGTAAGGGCCATGAGGACTTGACGTCATCCCCACCTTCCTCTCGGCTTATC
ACCGGCAGTCCCCCTAGAGTGCCCAACCAAATGATGGCAACTAGGGGCGAGGGTTGCGCTCGTTGCGGGACTTAACCCAACATCTCACGACACGAGCTGA
CGACAGCCATGCAGCACCTGTGTTCCGGCCAGCCGAACTGAAGGATCCCGTCTCCAGGATCCAAACCGGACATGTCAAAGGCTGGTAAGGTTCTGCGCGT
TGCTTCGAATTAAACCACATGCTCCACCGCTTGTGCGGGCCCCCGTCAATTCCTTTGAGTTTTAATCTTGCGACCGTACTCCCCAGGCGGAATGCTTAAA
GCGTTAGCTGCGCCACTGAGCAGCAAGCTGCCCAACGGCTGGCATTCATCGTTTACGGCGTGGACTACCAGGGTATCTAATCCTGTTTGCTCCCCACGCT
TTCGCGCCTCAGCGTCAGAACCGGACCAGTAAGCCGCCTTCGCCACTGGTGTTCTTGCGAATATCTACGAATTTCACCTCTACACTCGCAGTTCCACTTA
CCTCTTCCGGTCTCAAGCTCTACAGTATCGAAGGCAATTCTGTGGTTGAGCCACAGGCTTTCACCCCCGACTTATAAAGCCGCCTACGCGCCCTTTACGC
CCAGTGATTCCGAACAACGCTAGCCCCCTTCGTATTACCGCGGCTGCTGGCACGAAGTTAGCCGGGGCTTCTTCTTCCGGTACCGTCATTATCGTCCCGG
ACGAAAGAGCTTTACAACCCTAAGGCCTTCATCACTCACGCGGCATGGCTGGATCAGGCTTGCGCCCATTGTCCAATATTCCCCACTGCTGCCTCCCGTA
GGAGTTTGGGCCGTGTCTCAGTCCCAATGTGGCTGATCATCCTCTCAGACCAGCTACTGATCGTCGCCTTGGTGAGCCATTACCTCACCAACTAGCTAAT
CAGACGCGGGCCGATCCTTCAGCGATAAATCTTTCTGCTCTCGCACGTATCCGGTATTAGCCCAAGTTTCCCTGGGTTATCCCGAACTGAAGGGCACGTT
CCCACGCGTTACTCACCCGTCTGCCACTCACCCCGAAGGATGCGTTCGACTGCA

>EB243
GCTCAGGATGAACGCTGGCGGCGTGCCTAATACATGCAAGTCGAGCGAATGGATTGAGAGCTTGCTCTCAAGAAGTTAGCGGCGGACGGGTGAGTAACAC
GTGGGTAACCTGCCCATAAGACTGGGATAACTCCGGGAAACCGGGGCTAATACCGGATAATATTTTGAACTGCATGGTTCGAAATTGAAAGGCGGCTTCG
GCTGTCACTTATGGATGGACCCGCGTCGCATTAGCTAGTTGGTGAGGTAACGGCTCACCAAGGCAACGATGCGTAGCCGACCTGAGAGGGTGATCGGCCA
CACTGGGACTGAGACACGGCCCAGACTCCTACGGGAGGCAGCAGTAGGGAATCTTCCGCAATGGACGAAAGTCTGACGGAGCAACGCCGCGTGAGTGATG
AAGGCTTTCGGGTCGTAAAACTCTGTTGTTAGGGAAGAACAAGTGCTAGTTGAATAAGCTGGCACCTTGACGGTACCTAACCAGAAAGCCACGGCTAACT
ACGTGCCAGCAGCCGCGGTAATACGTAGGTGGCAAGCGTTATCCGGAATTATTGGGCGTAAAGCGCGCGCAGGTGGTTTCTTAAGTCTGATGTGAAAGCC
CACGGCTCAACCGTGGAGGGTCATTGGAAACTGGGAGACTTGAGTGCAGAAGAGGAAAGTGGAATTCCATGTGTAGCGGTGAAATGCGTAGAGATATGGA
GGAACACCAGTGGCGAAGGCGACTTTCTGGTCTGTAACTGACACTGAGGCGCGAAAGCGTGGGGAGCAAACAGGATTAGATACCCTGGTAGTCCACGCCG

TAAACGATGAGTGCTAAGTGTTAGAGGGTTTCCGCCCTTTAGTGCTGAAGTTAACGCATTAAGCACTCCGCCTGGGGAGTACGGCCGCAAGGCTGAAACT
CAAAGGAATTGACGGGGGCCCGCACAAGCGGTGGAGCATGTGGTTTAATTCGAAGCAACGCGAAGAACCTTACCAGGTCTTGACATCCTCTGAAAACCCT
AGAGATAGGGCTTCTCCTTCGGGAGCAGAGTGACAGGTGGTGCATGGTTGTCGTCAGCTCGTGTCGTGAGATGTTGGGTTAAGTCCCGCAACGAGCGCAA
CCCTTGATCTTAGTTGCCATCATTAAGTTGGGCACTCTAAGGTGACTGCCGGTGACAAACCGGAGGAAGGTGGGGATGACGTCAAATCATCATGCCCCTT
ATGACCTGGGCTACACACGTGCTACAATGGACGGTACAAAGAGCTGCAAGACCGCGAGGTGGAGCTAATCTCATAAAACCGTTCTCAGTTCGGATTGTAG
GCTGCAACTCGCCTACATGAAGCTGGAATCGCTAGTAATCGCGGATCAGCATGCCGCGGTGAATACGTTCCCGGGCCTTGTACACACCGCCCGTCACACC
ACGAGAGTTTGTAACACCCGAAGTCGGTGGGGTAACCTTTATGGAGCCAGCCGCCTAAGGTGGGACAAATAATTGGGGTGAATCTA

>EB244
ATGCGCGCCTATCATGCAGTCGAGCGAACAGACGAGGAGCTTGCTCCTTTGACGTTAGCGGCGGACGGGTGAGTAACACGTAGGTAACCTACCTATAAGA
CTGGGATAACTTCGGGAAACCGGAGCTAATACCGGATAATATTTCGAACCGCATGGTTCGATAGTGAAAGATGGCTTTGCTATCACTTATAGATGGACCT
GCGCCGTATTAGCTAGTTGGTAAGGTAACGGCTTACCAAGGCAACGATACGTAGCCGACCTGAGAGGGTGATCGGCCACACTGGAACTGAGACACGGTCC
AGACTCCTACGGGAGGCAGCAGTAGGGAATCTTCCGCAATGGGCGAAAGCCTGACGGAGCAACGCCGCGTGAGTGATGAAGGTCTTCGGATCGTAAAACT
CTGTTATTAGGGAAGAACAAACGTGTAAGTAACTGTGCACGTCTTGACGGTACCTAATCAGAAAGCCACGGCTAACTACGTGCCAGCAGCCGCGGTAATA
CGTAGGTGGCAAGCGTTATCCGGAATTATTGGGCGTAAAGCGCGCGTAGGCGGTTTTTTAAGTCTGATGTGAAAGCCCACGGCTCAACCGTGGAGGGTCA
TTGGAAACTGGAAAACTTGAGTGCAGAAGAGGAAAGTGGAATTCCATGTGTAGCGGTGAAATGCGCAGAGATATGGAGGAACACCAGTGGCGAAGGCGAC
TTTCTGGTCTGTAACTGACGCTGATGTGCGAAAGCGTGGGGATCAAACAGGATTAGATACCCTGGTAGTCCACGCCGTAAACGATGAGTGCTAAGTGTTA
GGGGGTTTCCGCCCCTTAGTGCTGCAGCTAACGCATTAAGCACTCCGCCTGGGGAGTACGACCGCAGGGTTGAAACTCAAAGGAATTGACGGGGACCCGC
ACAAGCGGTGGAGCATGTGGTTTATCGAAGCACCGCGAAGAACCTTACCAATCTTGACTCTTTGACCTTCTAAGATAAAATTTCCCCTTCGGGGACAAA
GTGACAGGTGGTGCAGGTGTCTCACTCGTCCTGAAATTTGGTTATCCCCACCAGC

>EB245
TGCAGTCGAACGGTAAGGCCCCTTCGGGGGTACACGAGTGGCGAACGGGTGAGTAACACGTGAGCAACCTGCCCTTGACACTGGGATAAGCGATTGAAAA
GTCGTCTAATACCGGATACGACCCACACAGGCATCTGAGTGGGTGGAAAGCTCCGGCGGTCAAGGATGGGCTCGCGGCCTATCAGCTTGTTGGTGAGGTA
ACGGCTCACCAAGGCTTCGACGGGTAGCCGGCCTGAGAGGGCGACCGGCCACACTGGGACTGAGATACGGCCCAGACTCCTACGGGAGGCAGCAGTGGGG
AATATTGCGCAATGGGCGAAAGCCTGACGCAGCAACGCCGCGTGCGGGATGACGGCCTTCGGGTTGTAAACCGCTTTCAGCTCCGACGAAGCTAACGTGA
CGGTAGGAGCAGAAGAAGCACCGGCCAACTACGTGCCAGCAGCCGCGGTAATACGTAGGGTGCGAGCGTTGTCCGGAATTATTGGGCGTAAAGAGCTTGT
AGGCGGTCTGTCGCGTCGGAAGTGAAAACTCAGGGCTTAACCCTGAGCCTGCTTCCGATACGGGCAGACTAGAGGAAGGTAGGGGAGAACGGAACTCCTG
GTGGAGCGGTGGAATGCGCAGATATCAGGAAGAACACCGGTGGCGAAGGCGGTTCTCTGGACCTTTCCTGACGCTGAGAAGCGAAAGCGTGGGGAGCAAA
CAGGCTTAGATACCCTGGTAGTCCACGCCGTAAACGGTGGGCACTAGGTGTGGGACTCATTCCACGAGTTCCGTGCCGTAGCTAACGCATTAAGTGCCCC
GCCTGGGGAGTACGGCCGCAAGGCTAAAACTCAAAGGAATTGACGGGGGCCCGCACAAGCGGCGGAGCATGTGGATTAATTCGATGCAACGCGAAGAAC
CTTACCTGGGTTTGACATATACCGGAAACATCTGGAGACAGGTGCCCCCGCAAGGTCGGTATACAGGTGGTGCATGGCTGTCGTCAGCTCGTGTCGTGAG
ATGTTGGGGTTAAGTCCCGCAACGAGCGCAACCCTCGTCCTATGTTGCCAGCGGATAATGCCGGGGACTCATAGGAGACTGCCGGGGTCAACTCGGAGGA
AGGTGGGGATGAGGTCAAGTCATCATGCCCCTTATGTCCAGGGCTTCACACATGCTACAATGGCTGGTACAAAGGGCTGCGAGACCGCAAGGTTTAGCGA
ATCCCAAAAAGCCAGTCTCAGTTCGGATTGGGGTCTGCAACTCGACCCCATGAAGTCGGAGTCGCTAGTAATCGCAGATCAGCAACGCTGCGGTGAATAC
GTTCCCGGGCCTTGTACACACCGCCCGTCAAGTCATGAAAGTCGGTAACACCCGAAGCCGGTGGCCCAACCCTTGTGGAGGGAG

>EB246
GGTACCGTAGAGCTTGATTATGGGTCAGAAGTCACGTGGGGAACCTGCCTGTAAGACTGGGATAACTCCGGGAAACCGGGGCTAATACCGGATAACTTCT
TTTCTCGCATGGGGAGAGGTTGAAAGACGGTTTTGCTGTCACTTACAGATGGGCCCGCGGCGCATTAGCTAGTTGGTGGGGTAACGGCCTACCAAGGCGA
CGATGCGTAGCCGACCTGAGAGGGTGATCGGCCACACTGGGACTGAGACACGGCCCAGACTCCTACGGGAGGCAGCAGTAGGGAATCTTCCGCAATGGAC
GAAAGTCTGACGGAGCAACGCCGCGTGAGTGATGAAGGCCTTCGGGTCGTAAAACTCTGTTATCAGGGAAGAACAAGTGCCGTTTAACTGACGGCGCCTT
GACGGTACCTGACCAGAAAGCCACGGCTAACTACGTGCCAGCAGCCGCGGTAATACGTAGGTGGCAAGCGTTGTCCGGAATTATTGGGCGTAAAGCGCGC
GCAGGCGGCTTCTTAAGTCTGATGTGAAAGCCCACGGCTCAACCGTGGAGGGTCATTGGAAACTGGGAGGCTTGAGTGCAGAAGAGAAGAGCGGAATTCC
ACGTGTAGCGGTGAAATGCGTAGAGATGTGGAGGAACACCAGTGGCGAAGGCGGCTCTTTGGTCTGTAACTGACGCTGAGGCGCGAAAGCGTGGGGAGAG
AACAGGATTAGATACCCTGGTATTCCACGCCGTAAACGATGAGTGCTAAGTGTTAGGGGGTTTCCGCCCCTTAGTGCTGCAGCAAACGCATTAAGCACTC
CGCCTGGGGAGTACGGCCGCAAGGCTGAAACTCAAAGGAATTGACGGGGGCCCGCACAAGCGGTGGAGCATGTGGTTTAATTCGAAGCAACGCGAAGAAC
CTTACCAGGTCTTGACATCCCGCTGATCACTCTGGAGACAGAGTTTTCCCTTCGGGGACAGCGGTGACAGGTGGTGCATGGTTGTCGTCAGCTCGTGTCG
TGAGATGTTGGGTTAAGTCCCGCAACGAGCGCAACCCTTGATCTTAGTTGCCAGCATTTAGTTGGGCACTCTAAGGTGACTGCCGGTGACAAACCGGAGG
AAGGTGGGGATGACGTCAAATCATCATGCCCCTTATGACCTGGGCTACACACGTGCTACAATGGATGGTACAGAGGGCAGCAAGACCGCGAGGTGGAGCA
AATCCCTTAAAACCATTCTCAGTTCGGATTGCAGGCTGCAACTCGCCTGCATGAAGCCGGAATCGTTGGTAATCGCGGATCATCATGACGCGGTGACTAC
GTTCCCGTAGATTTTGCTCATCGCTCATTACGTCGTAACAAGGTATCCGTAGAGTTTGATCATGGCTCAGTAAGTCG

>EB247
CTCCCTCCACAAGGGTTGGGCCACCGGCTTCGGGTGTTGCCGACTTTCGTGACGTGACGGGCGGTGTGTACAAGGCCCGGGAACGTATTCACCGCAGCGT
TGCTGATCTGCGATTACTAGCGACTCCGACTTCATGGGGTCGAGTTGCAGACCCCAATCCGAACTGAGACCGGCTTTTTGGGATTCGCTCACCCTTACAG
GATCGCAGCCCTTTGTACCGGCCATTGTAGCATGCGTGAAGCCCTGGACATAAGGGGCATGATGACTTGACGTCATCCCCACCTTCCTCCGAGTTGACCC
CGGCAGTCTCCTATGAGTCCCCACCATTAACGTGCTGGCAACATAGGACGAGGGTTGCGCTCGTTGCGGGACTTAACCCAACATCTCACGACACGAGCTG
ACGACAGCCATGCACCACCTGTACACCGACTAAAAGGGCACCTATCTCTAGGTGTTTCCGGTGTATGTCAAACCCAGGTAAGGTTCTTCGCGTTGCATCG
AATTAATCCGCATGCTCCGCCGCTTGTGCGGGCCCCCGTCAATTCCTTTGAGTTTTAGCCTTGCGGCCGTACTCCCCAGGCGGGGCGCTTAATGCGTTAG
CTGCGGCACGGAACCCATGGAATAGGCCCCACACCTAGCGCCCAACGTTTACGGTGTGGACTACCAGGGTATCTAATCCTGTTCGCTCCCCACACTTTCG
CTCCTCAGCGTCAGGTAATGCCCAGAGAACCGCCTTCGCCACCGGTGTTCCTCCTGATATCTGCGCATTTCACCGCTACACCAGGAATTCCGTTCTCCCC
TGCATACCTCTAGTCTGCCCGTATCGGAAGCAGGCACACAGTTAAGCTGTGTGTTTTCACTCCCGACGCGACAAACCGCCTACGAGCCCTTTACGCCCAA
TAATTCCGGACAACGCTCGCACCCTACGTATTACCGCGGCTGCTGGCACGTAGTTGGCCGGTGCTTCTTTAGTGGGTACCGTCACTTGCGCTTCGTCCCC
ACCGAAAGAGGTTTACAACCCGAAGGCCGTCATCCCTCACGCGGCGTTGCTGGATCAGGCTTCCGCCCATTGTCCAATATTCCCCACTGCTGCCTCCCGT
AGGAGTCTGGGCCGTGTCTCAGTCCCAGTGTGACCGGTCACCCTCTCAGGCCGGCTACCCGTCGAAGCCATGGTGAGCCGTTACCTCACCATCAAGCTGA
TAGGCCGCGAGCACATCCCGTACCGAAAAACTTTCCACCACACACCATGCAGCATGCGGTCATATCCGGTATTAATCACCGTTTCCGGTGGCTATCCCGA
AGTACGGGGCAGATTACTCACGTGTTACTCACCCGTTCGCCGCTCGAGTACCCCGAAGGGCCTTCCGCTCGACTGCA

>EB248
GGCGGCTGGCTCCTTACGGTTACCCCACCGACTTCGGGTGTTACAAACTCTCGTGGTGTGACGGGCGGTGTGTACAAGGCCCGGGAACGTATTCACCGCG
GCATGCTGATCCGCGATTACTAGCGATTCCGGCTTCATGCAGGCGAGTTGCAGCCTGCAATCCGAACTGAGAATGGTTTTATGGGATTGGCTTGACCTCG
CGGTCTTGCAGCCCTTTGTACCATCCATTGTAGCACGTGTGTAGCCCAGGTCATAAGGGGCATGATGATTTGACGTCATCCCCACCTTCCTCCGGTTTGT
CACCGGCAGTCACCTTAGAGTGCCCAACTGAATGCTGGCAACTAAGATCAAGGGTTGCGCTCGTTGCGGGACTTAACCCAACATCTCACGACACGAGCTG
ACGACAACCATGCACCACCTGTCACTTCGTCCCCCGAAGGGGAACCTTCTATCTCTAGAAGTAGCGAAGGATGTCAAGACCTGGTAAGGTTCTTCGCGTT
GCTTCGAATTAAACCACATGCTCCACCGCTTGTGCGGGCCCCCGTCAATTCCTTTGAGTTTCAGTCTTGCGACCGTACTCCCCAGGCGGAGTGCTTAATG
CGTTTGCTGCAGCACTAAAGGGCGGAAACCCTCTAMCACTTAGCACTCATCGTTTACGGCRTGGACTACCAGGGTATCTAATCCTGTTCGCTCCCCACGC
TTTCGCGCMTCAGCGTCAGTTACAGACCAGAGAGTCGCCTTCGCCACTGGTGTTCCTCCACATCTCTACGCATTTCACCGCTACACGTGGAATTCCACTC

TCCTCTTCTGCACTCAAGTTCCCCAGTTTCCAATGACCCTCCACGGTTGAGCCGTGGGCTTTCACATCAGACTTAAGAAACCGCCTGCGCGCGCTTTACG
CCCAATAATTCCGGACAACGCTTGCCACCTACGTATTACCGCGGCTGCTGGCACGTAGTTAGCCGTGGCTTTCTGGTTAGGTACCGTCAAGGTACCAGCA
GTTACTCTGGTACTTGTTCTTCCCTAACAACAGAACTTTACGACCCGAAGGCCTTCATCGTTCACGCGGCGTTGCTCCGTCAGACTTTCGTCCATTGCGG
AAGATTCCCTACTGCTGCCTCCCGTAGGAGTCTGGGCCGTGTCTCAGTCCCAGTGTGGCCGATCACCCTCTCAGGTCGGCTACGCATCGTCGCCTTGGTG
AGCCGTTACCTCACCAACTAGCTAATGCGCCGCGGGTCCATCTGTAAGTGATAGCCGAAACCATCTTTCAATTTTGAACCATGCGGTTCAAAATGTTATC
CGGTATTAGCTCCGGTTTCCCGGAGTTATCCCAATCTTACAGGCAGGTTACCCACGTGTTACTCACCCGTCCGCCGCTAACCTCAGGGAGCAAGCTCCCA
TTGGTTCGCTCGACTTGCA

>EB249
TTGATCATGGCTCAGTAAGTCGTAACCTGGTATCCGTAAATTTTGGGCGGAGGGCTGAGTAACACGTGGGCAACCTGCCTGTAAGACTGGGATAACTTCG
GGAAACCGGAGCTAATACCGGATAATTCTTATCCTCTCATGAGGATAAGCTGAAAGATGGTTTCGGCTATCACTTACAGATGGGCCCGCGGCGCAATAGC
TAGTTGGTGAGGTAACGGCTCACCAAGGCAACGATGCGTAGCCCACCTGAGAGGGTGATCGGCCACACTGGGACTGAGACACGGCCCAGACTCCTACGGG
AGGCAGCAGTAGGGAATCTTCCGCAATGGACGAAAGTCTGACGGAGCAACGCCNGCGTGAGTGATGAAGGTTTTCGGATCGTAAAACTCTGTTGTTAGGG
AAGAACAAGTACGAGAGTAACTGYTCGTACCTTGACGGTACCTAACCAGAAAGCCACGGCTAACTACGTGCCAGCAGCCGCGGTAATACGTAGGTGGCAA
GCGTTGTCCGGAATTATTGGGCGTAAAGCGCGCGCAGGCGGTTCCTTAAGTCTGATGTGAAAGCCCCCGGCTCAACCGGGGAGGGTCATTGGAAACTGGG
GAACTTGAGTGCAGAAGAGAAGAGCGGAATTCCACGTGTAGCGGTGAAATGCGTAGAGATGTGGAGGAACACCAGTGGCGAAGGCGGCTCTTTGGTCTGT
AACTGACGCTKARGCGCGAAAGCGTGGGGAGCAAACAGGATTAGATACCCTGGTAGTCCACGCCGTAAACGATGAGTGCTAAGTGTTAGAGGGTTTCCGC
CCTTTAGTGCTGCAGCAAACGCATTAAGCACTCCGCCTGGGGAGTACGGCCGCAAGGCTGAAACTCAAAGGAATTGACGGGGGCCCGCACAAGCGGTGGA
GCATGTGGTTTAATTCGAAGCAACGCGAAGAACCTTACCAGGTCTTGACATCCTCTGACACTCCTAGAGATAGGACGTTCCCCTTCGGGGGACAGAGTGA
CAGGTGGTGCATGGTTGTCGTCAGCTCGTGTCGTGAGATGTTGGGTTAAGTCCCGCAACGAGCGCAACCCTTGATCTTAGTTGCCAGCATTCAGTTGGGC
ACTCTAAGGTGACTGCCGGTGACAAACCGGAGGAAGGTGGGGATGACGTCAAATCATCATGCCCCTTATGACCTGGGCTACACACGTGCTACAATGGATG
GTACAAAGGGCTGCAAGACCGCGAGGTTAAGCGAATCCCATAAAACCATTCTCAGTTCGGATTGTAGGCTGCAACTCGCCTACATGAAGCTGGAATCGTT
AGTAATCGCGGATCAGCATGCCGCGGTGAATACGTTCCCGTACCTTGTACACACCGCCCATCACACCACGCGAGTT

>EB250
GCTCAGGATGAACGCTGGCGGCGTGCCTAATACATGCAAGTCGAGCGAACTGATTAGAAGCTTGCTTCTATGACGTTAGCGGCGGACGGGTGAGTAACAC
GTGGGCAACCTGCCTGTAAGACTGGGATAACTTCGGGAAACCGAAGCTAATACCGGATAGGATCTTCTCCTTCATGGGAGATGATTGAAAGATGGTTTCG
GCTATCACTTACAGATGGGCCCGCGGTGCATTAGCTAGTTGGTGAGGTAACGGCTCACCAAGGCAACGATGCATAGCCGACCTGAGAGGGTGATCGGCCA
CACTGGGACTGAGACACGGCCCAGACTCCTACGGGAGGCAGCAGTAGGGAATCTTCCGCAATGGACGAAAGTCTGACGGAGCAACGCCGCGTGAGTGATG
AAGGCTTTCGGGTCGTAAAACTCTGTTGTTAGGGAAGAACAAGTACGAGAGTAACTGCTCGTACCTTGACGGTACCTAACCAGAAAGCCACGGCTAACTA
CGTGCCAGCAGCCGCGGTAATACGTAGGTGGCAAGCGTTATCCGGAATTATTGGGCGTAAAGCGCGCGCAGGCGGTTTCTTAAGTCTGATGTGAAAGCCC
ACGGCTCAACCGTGGAGGGTCATTGGAAACTGGGGAACTTGAGTGCAGAAGAGAAAAGCGGAATTCCACGTGTAGCGGTGAAATGCGTAGAGATGTGGAG
GAACACCAGTGGCGAAGGCGGCTTTTTGGTCTGTAACTGACGCTGAGGCGCGAAAGCGTGGGGAGCAAACAGGATTAGATACCCTGGTAGTCCACGCCGT
AAACGATGAGTGCTAAGTGTTAGAGGGTTTCCGCCCTTTAGTGCTGCAGCTAACGCATTAAGCACTCCGCCTGGGGAGTACGGTCGCAAGACTGAAACTC
AAAGGAATTGACGGGGGCCCGCACAAGCGGTGGAGCATGTGGTTTAATTCGAAGCAACGCGAAGAACCTTACCAGGTCTTGACATCCTCTGACAACTCTA
GAGATAGAGCGTTCCCCTTCGGGGGACAGAGTGACAGGTGGTGCATGGTTGTCGTCAGCTCGTGTCGTGAGATGTTGGGTTAAGTCCCGCAACGAGCGCA
ACCCTTGATCTTAGTTGCCAGCATTTAGTTGGGCACTCTAAGGTGACTGCCGGTGACAAACCGGAGGAAGGTGGGGATGACGTCAAATCATCATGCCCCT
TATGACCTGGGCTACACACGTGCTACAATGGATGGTACAAAGGGCTGCAAGACCGCGAGGTCAAGCCAATCCCATAAAACCATTCTCAGTTCGGATTGTA
GGCTGCAACTCGCCTACATGAAGCTGGAATCGCTAGTAATCGCGGATCAGCATGCCGCGGTGAATACGTTCCCGGGCCTTGTACACACCGCCCGTCACAC
CACGAGAGTTTGTAACACCCGAAGTCGGTGGAGTAACCGTAAGGAGCTAGCCCCCTAAAGTGGGACAAATAATTGGGGTGA

>EB251
CGGCTGGCTCCTTGCGGTTACCCCACCGACTTCGGGTGTTATAAACTCTCGTGGTGTGACGGGCGGTGTGTACAAGACCCGGGAACGTATTCACCGCGGC
ATGCTGATCCGCGATTACTAGCAATTCCGACTTCATGCAGGCGAGTTGCAGCCTGCAATCCGAACTGAGACCGGCTTTGTTGGGATTGGCTCCATCTCGC
GATTTCGCAGCCCGTTGTACCGGCCATTGTAGTACGTGTGTAGCCCAGGTCATAAGGGGCATGATGATTTGACGTCATCCCCACCTTCCTCCGGTTTGTC
ACCGGCAGTCTATCTAGAGTGCCCACCCGAAGTGCTGGCAACTAAATATAAGGGTTGCGCTCGTTGCGGGACTTAACCCAACATCTCACGACACGAGCTG
ACGACAACCATGCACCACCTGTCTCAACTTTCCCCGAAGGGCACCTAACGCATCTCTGCCTCGTTAGTTGGATGTCAAGACCTGGTAAGGTTCTTCGCGT
TGCTTCGAATTAAACCACATACTCCACTGCTTGTGCGGGTCCCCGTCAATTCCTTTGAGTTTCAGTCTTGCGACCGTACTCCCCAGGCGGAGTGCTTAAT
GTGTTAACTTCGGCACCAAGGGTATCGAAACCCCTAACACCTAGCACTCATCGTTTACGGCGTGGACTACCAGGGTATCTAATCCTGTTTGCTCCCCACG
CTTTCGCGCCTCAGCGTCAGTTACAGCCCAGAGAGTCGCCTTCGCCACTGGTGTTCCTCCACATCTCTACGCATTTCACCGCTACACGTGGAATTCCACT
CTCCTCTTCTGCACTCAAGTCACACAGTTTCCAGTGCGATCCGGGGTTGAGCCCCGGGATTAAACACCAGACTTACATGACCGCCTGCGCGCGCTTTACG
CCCAATAATTCCGGACAACGCTTGCCCCCTACGTATTACCGCGGCTGCTGGCACGTAGTTAGCCGGGGCTTTCTTCTCAGGTACCGTCACCTTGAGAGCA
GTTACTCTCCCAAGCGTTCTTCCCTGGCAACAGAGCTTTACGATCCGAAAACCTTCATCACTCACGCGGCATTGCTCCGTCAGGCTTTCGCCCATTGCGG
AAGATTCCCTACTGCTGCCTCCCGTAGGAGTCTGGGCCGTGTCTCAGTCCCAGTGTGGCCGATCACCCTCTCAGGTCGGCTACGCATCGTCGCCTTGGTG
AGCCGTTACCTCACCAACTAGCTAATGCGCCGCAGGCCCATCCCCAAGTGACAGATTGCTCCGTCTTTCCAGTTCTCTTCAGGCGAAGAAAACAAGTATT
CGGTATTAGCTACCGTTTCCGGTAGTTGTCCCAAGCTTGAGGGCAGGTTGCCTACGTGTTACTCACCCGTCCGCCGCTAACCATCAGAGAAGCAAGCTTC
TCTTCAAGTCCGCTCGACTTGCA

>EB252
AGGTTACCTCACCGACTTCGGATGTTACAAACTCTCGTGGTGTGACGGGCGGTGTGTACAAGGCCCGGGAACGTATTCACCGCGGCATGCTGATCCGCGA
TTACTAGCGATTCCAGCTTCACGCAGTCGAGTTGCAGACTGCGATCCGAACTGAGAACAGATTTGTGGGATTGGCTTAACCTCGCGGTTTCGCTGCCCTT
TGTTCTGTCCATTGTAGCACGTGTGTAGCCCAGGTCATAAGGGGCATGATGATTTGACGTCATCCCCACCTTCCTCCGGTTTGTCACCGGCAGTCACCTT
AGAGTGCCCAACTGAATGCTGGCAACTAAGATCAAGGGTTGCGCTCGTTGCGGGACTTAACCCAACATCTCASGACACGAGCTGACGACAACCATGCACC
ACCTGTCACTCTGCCCCCGAAGGGGACGTCCTATCTCTAGGATTGTCAGAGGATGTCAAGACCTGGTAAGGTTCTTCGCGTTGCTTCGAATTAAACCACA
TGCTCCACCGCTTGTGCGGGCCCCCGTCAATTCCTTTGAGTTTCAGTCTTGCGACCGTACTCCCCAGGCGGAGTGCTTAATGCGTTAGCTGCAGCACTAA
GGGGCGGAAACCCCCTAACACTTAGCACTCATCGTTTACGGCGTGGACTACCAGGGTATCTAATCCTGTTCGCTCCCCACGCTTTCGCTCCTCAGCGTCA
GTTACAGACCAGAGAGTCGCCTTCGCCACTGGTGTTCCTCCACATCTCTACGCATTTCACCGCTACACGTGGAATTCCACTCTCCTCTTCTGCACTCAAG
TTCCCCAGTTTCCAATGACCCTCCCCGGTTGAGCCGGGGGCTTTCACATCAGACTTAAGAAACCGCCTGCGAGCCCTTTACGCCCAATAATTCCGGACAA
CGCTTGCCACCTACGTATTACCGCGGCTGCTGGCACGTAGTTAGCCGTGGCTTTCTGGTTAGGTACCGTCAAGGTGCCGCCCTATTTGAACGGCACTTGT
TCTTCCCTAACAACAGAGCTTTACGATCCGAAAACCTTCATCACTCACGCGGCGTTGCTCCGTCAGACTTTCGTCCATTGCGGAAGATTCCCTACTGCTG
CCTCCCGTAGGAGTCTGGGCCGTGTCTCAGTCCCAGTGTGGCCGATCACCCTCTCAGGTCGGCTACGCATCGTCGCCTTGGTGAGCCGTTACCTCACCAA
CTAGCTAATGCGCCGCGGGTCCATCTGTAAGTGGTAGCCGAAGCCACCTTTTATGTCTGAACCATGCGGTTCAAACAACCATCCGGTATTAGCCCCGGTT
TCCCGGAGTTATCCCAGTCTTACAGGCAGGTTACCCACGTGTTACTCACCCGTCCGCCGCTAACATCAGGGAGCAAGCTCTCCATCTGTACCGC

>EB253
TTAGCGGCGGACGGGTGAGTAACACGTGGGCAACCTACCTATAAGACTGGGATAACTTCGGGAAACCGGAGCTAATACCGGATGACATAAAGGAACTCCT
GTTCCTTTATTGAAAGATGGCTTCGGCTATCACTTACAGATGGGCCCGCGGCGCATTAGCTAGTTGGTGAGGTAACGGCTCACCAAGGCGACGATGCGTA
GCCGACCTGAGAGGGTGATCGGCCACACTGGGACTGAGACACGGCCCAGACTCCTACGGGAGGCAGCAGTAGGGAATCTTCCGCAATGGACGAAAGTCTG

ACGGAGCAACGCCGCGTGAACGATGAAGGCCTTCGGGTCGTAAAGTTCTGTTGTTAGGGAAGAACAAGTGCTAGTTAAATAAGCTGGCACCTTGACGGTA
CCTAACCAGAAAGCCACGGCTAACTACGTGCCAGCAGCCGCGGTAATACGTAGGTGGCAAGCGTTGTCCGGAATTATTGGGCGTAAAGCGCGCGCAGGCG
GTTTCTTAAGTCTGATGTGAAAGCCCCCGGCTCAACCGGGGAGGGTCATTGGAAACTGGGAAACTTGAGTGCAGAAGAGGAAAGTGGAATTCCAAGTGTA
GCGGTGAAATGCGTAGAGATTTGGAGGAACACCAGTGGCGAAGGCGACTTTCTGGTCTGTAACTGACGCTGAGGCGCGAAAGCGTGGGGAGCAAACAGGA
TTAGATACCCTGGTAGTCCACGCTGTAAACGATGAGTGCTAAGTGTTAGAGGGTTTCCGCCCTTTAGTGCTGAAGTTAACGCATTAAGCACTCCGCCTGG
GGAGTACGGTCGCAAGACTGAAACTCAAAGGAATTGACGGGGGCCCGCACAAGTGGTGGAGCATGTGGTTTAATTCGAAGCAACGCGAAGAACCTTACCA
GGTCTTGACATCCTCTGACAACCCTAGAGATAGGGCTTTCCCTTCGGGGACAGAGTGACAGGTGGTGCATGGTTGTCGTCAGCTCGTGTCGTGAGATGTT
GGGTTAAGTCCCGCAACGAGCGCAACCCTTGATCTTAGTTGCCAGCATTTAGTTGGGCACTCTAAGGTGACTGCCGGTGACAAACCGGAGGAAGGTGGGG
ATGACGTCAAATCATCATGCCCCTTATGACCTGGGCTACACACGTGCTACAATGGATAGTACAAAGGGTTGCAAGACCGCGAGGTGGAGCTAATCCCATA
AAACTATTCTCAGTTCGGATTGTAGGCTGCAACTCGCCTACATGAAGCCGGAATCACTAGTAATCGCGGATCAGCATGCCGCGGTGAATACGTTCCCGGT
CCTTGTACACACCGCCCGTCACACCACGAGAGTTTGTAACAT

>EB254
GGCGGCTGGCTCCTTGCGGTTACCTCACCGACTTCGGGTGTTGTAAACTCTCGTGGTGTGACGGGCGGTGTGTACAAGACCCGGGAACGTATTCACCGCG
GCATGCTGATCCGCGATTACTAGCAATTCCGACTTCATGCAGGCGAGTTGCAGCCTGCAATCCGAACTGAGACCAGCTTTGATAGGATTGGCTCCACCTC
GCGGCTTCGCTTCCCGTTGTACTGGCCATTGTAGTACGTGTGTAGCCCAGGTCATAAGGGGCATGATGATTTGACGTCATCCCCGCCTTCCTCCGGTTTG
TCACCGGCAGTCATTCTAGAGTGCCCACCCGAAGTGCTGGCAACTAAAATCAAGGGTTGCGCTCGTTGCGGGACTTAACCCAACATCTCACGACACGAGC
TGACGACAACCATGCACCACCTGTCTCCTCTGTCCCGAAGGAAAGGTCTATCTCTAGACCCGGTCAGAGGGATGTCAAGACCTGGTAAGGTTCTTCGCGT
TGCTTCGAATTAAACCACATACTCCACTGCTTGTGCGGGTCCCCGTCAATTCCTTTGAGTTTCAGTCTTGCGACCGTACTCCCCAGGCGGAATGCTTAAT
GTGTTAACTTCGGCACCAAGGGTATCGAAACCCCTAACACCTAGCATTCATCGTTTACGGCGTGGACTACCAGGGTATCTAATCCTGTTTGCTCCCCACG
CTTTCGCGCCTCAGCGTCAGTTACAGCCCAGAGAGTCGCCTTCGCCACTGGTGTTCCTCCACATATCTACGCATTTCACCGCTACACGTGGAATTCCACT
CTCCTCTTCTGCACTCAAGTCACCCAGTTTCCAGTGCGAACCAAGGTTGAGCCTTGGCCTTAAACACCAGACTTAAATGACCGCCTGCGCGCGCTTTACG
CCCAATAATTCCGGACAACGCTTGCCCCCTACGTATTACCGCGGCTGCTGGCACGTAGTTAGCCGGGGCTTTCTTCTCAGGTACCGTCACTCCGATAGCA
GTTACTCTATCGGACGTTCTTCCCTGGCAACAGAGCTTTACGATCCGAAAACCTTCATCACTCACGCGGCGTTGCTCCGTCAGGCTTTCGCCCATTGCGG
AAGATTCCCTACTGCTGCCTCCCGTAGGAGTCTGGGCCGTGTCTCAGTCCCAGTGTGGCCGTTCACCCTCTCAGGTCGGCTACGCATCGTCGCCTTGGTG
AGCCGTTACCTCACCAACTAGCTAATGCGCCGCAGGCCCATCCGCAAGTGACAGATTGCTCCGTCTTTCATCATCCCCTCAGGAGAGGAAATGAGATATC
CGGTATTAGCTCACGTTTCCGTGGGTTATCCCGGTCTTGCAGGCAGGTTGCCTACGTGTTACTCACCCGTCCGCCGCTAACCATCAGGAGAGCAAGCTCT
CCATCAAGTCCGCTCGACTGC

>EB255
TCGAGCGAATGGATTAAGAGCTTGCTCTTATGAAGTTAGCGGCGGACGGGTGAGTAACACGTGGGTAACCTGCCCATAAGACTGGGATAACTCCGGGAAA
CCGGGGCTAATACCGGATAACATTTTGAACCGCATGGTTCGAAATTGAAAGGCGGCTTCGGCTGTCACTTATGGATGGACCCGCGTCGCATTAGCTAGTT
GGTGAGGTAACGGCTCACCAAGGCAACGATGCGTAGCCGACCTGAGAGGGTGATCGGCCACACTGGGACTGAGACACGGCCCAGACTCCTACGGGAGGCA
GCAGTAGGGAATCTTCCGCAATGGACGAAAGTCTGACGGAGCAACGCCGCGTGAGTGATGAAGGCTTTCGGGTCGTAAAACTCTGTTGTTAGGGAAGAAC
AAGTGCTAGTTGAATAAGCTGGCACCTTGACGGTACCTAACCAGAAAGCCACGGCTAACTACGTGCCAGCAGCCGCGGTAATACGTAGGTGGCAAGCGTT
ATCCGGAATTATTGGGCGTAAAGCGCGCGCAGGTGGTTTCTTAAGTCTGATGTGAAAGCCCACGGCTCAACCGTGGAGGGTCATTGGAAACTGGGAGACT
TGAGTGCAGAAGAGGAAAGTGGAATTCCATGTGTAGCGGTGAAATGCGTAGAGATATGGAGGAACACCAGTGGCGAAGGCGACTTTCTGGTCTGTAACTG
ACACTGAGGCGCGAAAGCGTGGGGAGCAAACAGGATTAGATACCCTGGTAGTCCACGCCGTAAACGATGAGTGCTAAGTGTTAGAGGGTTTCCGCCCTTT
AGTGCTGAAGTTAACGCATTAAGCACTCCGCCTGGGGAGTACGGCCGCAAGGCTGAAACTCAAAGGAATTGACGGGGGCCCGCACAAGCGGTGGAGCATG
TGGTTTAATTCGAAGCAACGCGAAGAACCTTACCAGGTCTTGACATCCTCTGACAACCCTAGAGATAGGGCTTCTCCTTCGGGAGCAGAGTGACAGGTGG
TGCATGGTTGTCGTCAGCTCGTGTCGTGAGATGTTGGGTTAAGTCCCGCAACGAGCGCAACCCTTGATCTTAGTTGCCATCATTAAGTTGGGCACTCTAA
GGTGACTGCCGGTGACAAACCGGAGGAAGGTGGGGATGACGTCAAATCATCATGCCCCTTATGACCTGGGCTACACACGTGCTACAATGGACGGTACAAA
GAGCTGCAAGACCGCGAGGTGGAGCTAATCTCATAAAACCGTTCTCAGTTCGGATTGTAGGCTGCAACTCGCCTACATGAAGCTGGAATCGCTAGTAATC
GCGGATCAGCATGCCGCGGTGAATACGTTCCCGGGCCTTGTACACACCGCCCGTCACACCACGAGAGTTTGTAACACCCGAAGTCGGTGGGGTAACCTTT
TGGAGCCAGCCGCC

>EB256
CATGCAAGTCGAGCGGGGTTAGTTAGAAGCTTGCTTCTAACTAACCTAGCGGCGGACGGGTGAGTAACACGTAGGCAACCTGCCCACAAGACAGGGATAA
CTACCGGAAACGGTAGCTAATACCCGATACATCCTTTTCCTGCATGGGAGAAGGAGGAAAGGCGGAGCAATCTGTCACTTGTGGATGGGCCTGCGGCGCA
TTAGCTAGTTGGTGGGGTAAAGGCCTACCAAGGCGACGATGCGTAGCCGACCTGAGAGGGTGATCGGCCACACTGGGACTGAGACACGGCCCAGACTCCT
ACGGGAGGCAGCAGTAGGGAATCTTCCGCAATGGGCGAAAGCCTGACGGAGCAACGCCGCGTGAGTGATGAAGGTTTTCGGATCGTAAAGCTCTGTTGCC
AGGGAAGAACGTCTTATAGAGTAACTGCTATTAAAGTGACGGTACCTGAGAAGAAAGCCCCGGCTAACTACGTGCCAGCAGCCGCGGTAATACGTAGGGG
GCAAGCGTTGTCCGGAATTATTGGGCGTAAAGCGCGCGCAGGCGGCTCTTTAAGTCTGGTGTTTAATCCCGAGGCTCAACTTCGGGTCGCACTGGAAACT
GGGGAGCTTGAGTGCAGAAGAGGAGAGTGGAATTCCACGTGTAGCGGTGAAATGCGTAGAGATGTGGAGGAACACCAGTGGCGAAGGCGACTCTCTGGGC
TGTAACTGACGCTGAGGCGCGAAAGCGTGGGGAGCAAACAGGATTAGATACCCTGGTAGTCCACGCCGTAAACGATGAATGCTAGGTGTTAGGGGTTTCG
ATACCCTTGGTGCCGAAGTTAACACATTAAGCATTCCGCCTGGGGAGTACGGTCGCAAGACTGAAAACTCAAAGGAATTGACGGGGACCCGCACAAGCA
GTGGAGTATGTGGTTTAATTCGAAGCAACGCGAAGAACCTTACCAGGTCTTGACATCCCTTTGACCGGTCTAGAGATAGACCTTTCCTTCGGGACAGAGG
AGACAGGTGGTGCATGGTTGTCGTCAGCTCGTGTCGTGAGATGTTGGGTTAAGTCCCGCAACGAGCGCAACCCTTATGCTTAGTTGCCAGCAGGTCAAGC
TGGGCACTCTAAGCAGACTGCCGGTGACAAACCGGAGGAAGGTGGGGATGACGTCAAATCATCATGCCCCTTATGACCTGGGCTACACACGTACTACAAT
GGCCGGTACAACGGGAAGCGAAGGCGCGATGTGGAGCCAATCCTAGAAAAGCCGGTCTCAGTTCGGATTGTAGGCTGCAACTCGCCTACATGAAGTCGGA
ATTGCTAGTAATCGCGGATCAGCATGCCGCGGTGAATACGTTCCCGGGTCTTGTACACACCGCCCGTCACACCACGAGAGTTTACAACACCCGAAGTCGG
TGAGGTAACCGCAAGGAGCCAGCCGCC

>EB257
TCGAGCGAATGGATTAAGAGCTTGCTCTTATGAAGTTAGCGGCGGACGGGTGAGTAACACGTGGGTAACCTGCCCATAAGACTGGGATAACTCCGGGAAA
CCGGGGCTAATACCGGATAACATTTTGAACCGCATGGTTCGAAATTGAAAGGCGGCTTCGGCTGTCACTTATGGATGGACCCGCGTCGCATTAGCTAGTT
GGTGAGGTAACGGCTCACCAAGGCAACGATGCGTAGCCGACCTGAGAGGGTGATCGGCCACACTGGGACTGAGACACGGCCCAGACTCCTACGGGAGGCA
GCAGTAGGGAATCTTCCGCAATGGACGAAAGTCTGACGGAGCAACGCCGCGTGAGTGATGAAGGCTTTCGGGTCGTAAAACTCTGTTGTTAGGGAAGAAC
AAGTGCTAGTTGAATAAGCTGGCACCTTGACGGTACCTAACCAGAAAGCCACGGCTAACTACGTGCCAGCAGCCGCGGTAATACGTAGGTGGCAAGCGTT
ATCCGGAATTATTGGGCGTAAAGCGCGCGCAGGTGGTTTCTTAAGTCTGATGTGAAAGCCCACGGCTCAACCGTGGAGGGTCATTGGAAACTGGGAGACT
TGAGTGCAGAAGAGGAAAGTGGAATTCCATGTGTAGCGGTGAAATGCGTAGAGATATGGAGGAACACCAGTGGCGAAGGCGACTTTCTGGTCTGTAACTG
ACACTGAGGCGCGAAAGCGTGGGGAGCAAACAGGATTAGATACCCTGGTAGTCCACGCCGTAAACGATGAGTGCTAAGTGTTAGAGGGTTTCCGCCCTTT
AGTGCTGAAGTTAACGCATTAAGCACTCCGCCTGGGGAGTACGGCCGCAAGGCTGAAACTCAAAGGAATTGACGGGGGCCCGCACAAGCGGTGGAGCATG
TGGTTTAATTCGAAGCAACGCGAAGAACCTTACCAGGTCTTGACATCCTCTGAAAACCCTAGAGATAGGGCTTCTCCTTCGGGAGCAGAGTGACAGGTGG
TGCATGGTTGTCGTCAGCTCGTGTCGTGAGATGTTGGGTTAAGTCCCGCAACGAGCGCAACCCTTGATCTTAGTTGCCATCATTAAGTTGGGCACTCTAA
GGTGACTGCCGGTGACAAACCGGAGGAAGGTGGGGATGACGTCAAATCATCATGCCCCTTATGACCTGGGCTACACACGTGCTACAATGGACGGTACAAA
GAGCTGCAAGACCGCGAGGTGGAGCTAATCTCATAAAACCGTTCTCAGTTCGGATTGTAGGCTGCAACTCGCCTACATGAAGCTGGAATCGCTAGTAATC

GCGGATCAGCATGCCGCGGTGAATACGTTCCCGGGCCTTGTACACACCGCCCGTCACACCACGAGAGTTTGTAACATCCGAAGTCGGTGGGGTAACCTTT
TGGAGCCAGCCG

>EB258
GGTTCAAGGATGAAAGACGGTTTCGGCTGTCACTTACAGATGGACCCGCGGCGCATTAGCTAGTTGGTGAGGTAACGGCTCACCAAGGCGACGATGCGTA
GCCGACCTGAGAGGGTGATCGGCCACACTGGGACTNGAGACACGGCCCAGACTCCTACGGGANGGCAGCAGTAGGGAATCTTCCGCAATGGACGAAAGTC
TGACGGAGCAACGCCGCGTGAGTGATGAAGGTTTTCGGATCGTAAAGCTCTGTTGTTAGGGAAGAACAAGTGCAAGAGTAACTGCTTGCACCTTGACGGT
ACCTAACCAGAAAGCCACGGCTAACTACGTGCCAGCAGCCGCGGTAATACGTAGGTGGCAAGCGTTGTCCGGAATTATTGGGCGTAAAGGGCTCGCAGGC
GGTTTCTTAAGTCTGATGTGAAAGCCCCCGGCTCAACCGGGGAGGGTCATTGGAAACTGGGAAACTTGAGTGCAGAAGAGGAGAGTGGAATTCCACGTGT
AGCGGTGAAATGCGTAGAGATGTGGAGGAACACCAGTGGCGAAGGCGACTCTCTGGTCTGTAACTGACGCTGAGGAGCGAAAGCGTGGGGAGCGAACAGG
ATTAGATACCCTGGTAGTCCACGCCGTAAACGATGAGTGCTAAGTGTTAGGGGGTTTCCGCCCCTTAGTGCTGCAGCTAACGCATTAAGCACTCCGCCTG
GGGAGTACGGTCGCAAGACTGAAACTCAAAGGAATTGACGGGGGCCCGCACAAGCGGTGGAGCATGTGGTTTAATTCGAAGCAACGCGAAGAACCTTACC
AGGTCTTGACATCCTCTGACAACCCTAGAGATAGGGCTTTCCCTTCGGGGACAGAGTGACAGGTGGTGCATGGTTGTCGTCAGCTCGTGTCGTGAGATGT
TGGGTTAAGTCCCGCAACGAGCGCAACCCTTGATCTTAGTTGCCAGCATTCAGTTGGGCACTCTAAGGTGACTGCCGGTGACAAACCGGAGGAAGGTGGG
GATGACGTCAAATCATCATGCCCCTTATGACCTGGGCTACACACGTGCTACAATGGACAGAACAAAGGGCTGCGAGACCGCAAGGTTTAGCCAATCCCAC
AAATCTGTTCTCAGTTCGGATCGCAGTCTGCAACTCGAATGCGTGAAGCAGGAATCGGTAGTAATCGAGGATCAGCCTGCCGCGGGGAATACGTTCCCGG
TCGTGGTACACACCGCCCCTCAC

>EB259
TACATGCAAGTCGAGCGGACTTGAAGAGAAGCTTGCTTCTCTGATAGTTAGCGGCGGACGGGTGAGTAACACGTAGGCAACCTGCCCTCAAGCTTGGGAC
AACTACCGGAAACGGTAGCTAATACCGAATAGTTGTTTTCTTCTCCTGAAGAGAACTGGAAAGACGGAGCAATCTGTCACTTGGGGATGGGCCTGCGGCG
CATTAGCTAGTTGGTGGGGTAACGGCTCACCAAGGCGACGATGCGTAGCCGACCTGAGAGGGTGATCGGCCACACTGGGACTGAGACACGGCCCAGACTC
CTACGGGAGGCAGCAGTAGGGAATCTTCCGCAATGGGCGAAAGCCTGACGGAGCAATGCCGCGTGAGTGATGAAGGTTTTCGGATCGTAAAGCTCTGTTG
CCAGGGAAGAACGCTTGGGAGAGTAACTGCTCTCAAGGTGACGGTACCTGAGAAGAAAGCCCCGGCTAACTACGTGCCAGCAGCCGCGGTAATACGTAGG
GGGCAAGCGTTGTCCGGAATTATTGGGCGTAAAGCGCGCGCAGGCGGTCATTTAAGTCTGGTGTTTAATCCCGGGGCTCAACCCCGGATCGCACTGGAAA
CTGGGTGACTTGAGTGCAGAAGAGGAGAGTGGAATTCCACGTGTAGCGGTGAAATGCGTAGATATGTGGAGGAACACCAGTGGCGAAGGCGACTCTCTGG
GCTGTAACTGACGCTGAGGCGCGAAAGCGTGGGGAGCAAACAGGATTAGATACCCTGGTAGTCCACGCCGTAAACGATGAGTGCTAGGTGTTAGGGGTTT
CGATACCCTTGGTGCCGAAGTTAACACATTAAGCACTCCGCCTGGGGGAGTACGGTCGCAAGACTGAAAACTCAAAGGAATTGACGGGGACCCGCACAAG
CAGTGGAGTATGTGGTTTAATTCGAAGCAACGCGAAGAACCTTACCAGGTCTTGACATCCCCACTGAACCGGTGCAGAGATGTAACTTTCCTTCGGGAAC
AGACGAAGACAGGTGTGCATGGTTGTCGTCAGCTCGTGTCGTGAGATGTTGGGTTAAGTCCCGCAACGAGCGCAACCCTTGATCTTAGTTGCCAGCACTT
CGGGTGGGCACTCTAAGGTGACTGCCGGTGACAAACCGGAGGAAGGTGGGGATGACGTCAAATCATCATGCCCCTTATGACCTGGGCTACACACGTACTA
CAATGGCCGGTACAACGGGCTGTGAAGCCGCGAGGTGGAACGAATCCTAAAAAGCCGGTCTCAGTTCGGATTGCAGGCTGCAACTCGCCTGCATGAAGTC
GGAATTGCTAGTAATCGCGGATCAGCATGCCGCGGTGAATACGTTCCCGGGTCTTGTACACACCGCCCGTCACACCACGAGAGTTTATAACACCCGAAGT
CGGTGGGGTAACCGCAAGGAGCCAGCCG

>EB260
TGCAAGTCGAGCGGACTTGATGAGAAGCTTGCTTCTCTGATGGTTAGCGGCGGACGGGTGAGTAACACGTAGGCAACCTGCCCTCAAGCTTGGGACAACT
ACCGGAAACGGTAGCTAATACCGAATAGTTGTTTTCTTCTCCTGAAGAGAACTGGAAAGACGGAGCAATCTGTCACTTGGGGATGGGCCTGCGGCGCATT
AGCTAGTTGGTGGGGTAACGGCTCACCAAGGCGACGATGCGTAGCCGACCTGAGAGGGTGATCGGCCACACTGGGACTGAGACACGGCCCAGACTCCTAC
GGGAGGCAGCAGTAGGGAATCTTCCGCAATGGGCGAAAGCCTGACGGAGCAATGCCGCGTGAGTGATGAAGGTTTTCGGATCGTAAAGCTCTGTTGCCAG
GGAAGAACGCTTGGGAGAGTAACTGCTCTCAAGGTGACGGTACCTGAGAAGAAAGCCCCGGCTAACTACGTGCCAGCAGCCGCGGTAATACGTAGGGGGG
CAAGCGTTGTCCGGAATTATTGGGCGTAAAGCGCGCGCAGGCGGTCATTTAAGTCTGGTGTTTAATCCCGGGGCTCAACCCCGGATCGCACTGGAAACTG
GGTGACTTGAGTGCAGAAGAGGAGAGTGGAATTCCACGTGTAGCGGTGAAATGCGTAGATATGTGGAGGAACACCAGTGGCGAAGGCGACTCTCTGGGCT
GTAACTGACGCTGAGGCGCGAAAGCGTGGGGAGCAAACAGGATTAGATACCCTGGTAGTCCACGCCGTAAACGATGAGTGCTAGGTGTTAGGGGTTTCGA
TACCCTTGGTGCCGAAGTTAACACATTAAGCACTCCGCCTGGGGAGTACGGTCGCAAGACTGAAACTCAAAGGAATTGACGGGGACCCGCACAAGCAGTG
GAGTATGTGGTTTAATTCGAAGCAACGCGAAGAACCTTACCAGGTCTTGACATCCCTCTGACCGGTACAGAGATGTACCTTTTCCTTCGGGACAGAGGAG
ACAGGTGGTGCATGGTTGTCGTCAGCTCGTGTCGTGAGATGTTGGGTTAAGTCCCGCAACGAGCGCAACCCTTGATCTTAGTTGCCAGCACTTCGGGTGG
GCACTCTAAGGTGACTGCCGGTGACAAACCGGAGGAAGGTGGGGATGACGTCAAATCATCATGCCCCTTATGACCTGGGCTACACACGTACTACAATGGC
CGGTACAACGGGCTGTGAAGCCGCGAGGTGGAACGAATCCTAAAAAGCCGGTCTCAGTTCGGATTGCAGGCTGCAACTCGCCTGCATGAAGTCGGAATTG
CTAGTAATCGCGGATCAGCATGCCGCGGTGAATACGTTCCCGGGTCTTGTACACACCGCCCGTCACACCACGAGAGTTTATAACACCCGAAGTCGGTGGG
GTAACCGCAAGGAGCC

>EB261
GCAAGTCGAGCGGAGTTGATGGAGTGCTTGCACTCCTGATGCTTAGCGGCGGACGGGTGAGTAACACGTAGGTAACCTGCCTGTAAGACTGGGATAACAT
TCGGAAACGAATGCTAATACCGGATACGCGAATTTCTCGCATGAGGAATTCGGGAAAGACGGAGCAATCTGTCACTTACAGATGGACCTGCGGCGCATTA
GCTAGTTGGTGAGGTAACGGCTCACCAAGGCGACGATGCGTAGCCGACCTGAGAGGGTGATCGGCCACACTGGGACTGAGACACGGCCCAGACTCCTACG
GGAGGCAGCAGTAGGGAATCTTCCGCAATGGACGAAAGTCTGACGGAGCAACGCCGCGTGAGTGATGAAGGTTTTCGGATCGTAAAGCTCTGTTGCCAGG
GAAGAACGCTTGTGAGAGTAACTGCTCACAAGGTGACGGTACCTGAGAAGAAAGCCCCGGCTAACTACGTGCCAGCAGCCGCGGTAATACGTAGGGGGCA
AGCGTTGTCCGGAATTATTGGGCGTAAAGCGCGCGCAGGCGGTCAATTAAGTCTGGTGTTTAATCCCGGGGCTCAACCCCGGGTCGCACTGGAAACTGGT
TGACTTGAGTACAGAAGAGGAAAGTGGAATTCCACGTGTAGCGGTGAAATGCGTAGAGATGTGGAGGAACACCAGTGGCGAAGGCGACTTTCTGGGCTGT
AACTGACGCTGAGGCGCGAAAGCGTGGGGAGCAAACAGGATTAGATACCCTGGTAGTCCACGCCGTAAACGATGAATGCTAGGTGTTAGGGGTTTCGATA
CCCTTGGTGCCGAAGTTAACACATTAAGCATTCCGCCTGGGGAGTACGGTCGCAAGACTGAAACTCAAAGGAATTGACGGGGACCCGCACAAGCAGTGGA
GTATGTGGTTTAATTCGAAGCAACGCGAAGAACCTTACCAGGTCTTGACATCCAACTAACGAAGCAGAGATGCATTAGGTGCCCTTCGGGGAAAGTTGAG
ACAGGTGGTGCATGGTTGTCGTCAGCTCGTGTCGTGAGATGTTGGGTTAAGTCCCGCAACGAGCGCAACCCTTGATCTTAGTTGCCAGCACTTTGGGTGG
GCACTCTAGGATGACTGCCGGTGACAAACCGGAGGAAGGTGGGGATGACGTCAAATCATCATGCCCCTTATGACCTGGGCTACACACGTACTACAATGGC
CGATACAACGGGAAGCGAAACCGCGAGGTGGAGCCAATCCTATCAAAGTCGGTCTCAGTTCGGATTGCAGGCTGCAACTCGCCTGCATGAAGTCGGAATT
GCTAGTAATCGCGGATCAGCATGCCGCGGTGAATACGTTCCCGGGTCTTGTACACACCGCCCGTCACACCACGAGAGTTTACAACACCCGAAGTCGGTGG
GGTAACCCGCAAGGGAGCCAGCCG

>EB262
TCGGACGGTAGCACAGAGAGCTTGCTCTCGGGTGACGAGTGGCGGACGGGTGAGTAATGTCTGGGGATCTGCCCGATAGAGGGGGATAACCACTGGAAAC
GGTGGCTAATACCGCATAACGTCGCAAGACCAAAGAGGGGGACCTTCGGGCCTCTCACTATCGGATGAACCCAGATGGGATTAGCTAGTAGGCGGGGTAA
TGGCCCACCTAGGCGACGATCCCTAGCTGGTCTGAGAGGATGACCAGCCACACTGGAACTGAGACACGGTCCAGACTCCTACGGGAGGCAGCAGTGGGGA
ATATTGCACAATGGGCGCAAGCCTGATGCAGCCATGCCGCGTGTATGAAGAAGGCCTTCGGGTTGTAAAGTACTTTCAGCGGGGAGGAAGGCGACGGGGT
TAATAAGCCTGTCGATTGACGTTACCCGCAGAAGAAGCACCGGCTAACTCCGTGCCAGCAGCCGCGGTAATACGGAGGGTGCAAGCGTTAATCGGAATTA
CTGGGCGTAAAGCGCACGCAGGCGGTCTGTTAAGTCAGATGTGAAATCCCCGGGCTTAACCTGGGAACTGCATTTGAAACTGGCAGGCTTGAGTCTTGTA
GAGGGGGGTAGAATTCCAGGTGTAGCGGTGAAATGCGTAGAGATCTGGAGGAATACCGGTGGCGAAGGCGGCCCCCTGGACAAAGACTGACGCTCAGGTG
CGAAAGCGTGGGGAGCAAACAGGATTAGATACCCTGGTAGTCCACGCCGTAAACGATGTCGACTTGGAGGTTGTTCCCCTGAGGAGTGGCTTCCGGAGCT

AACGCGTTAAGTCGACCGCCTGGGGAGTACGGCCGCAAGGTTAAAACTCAAATGAATTGACGGGGGCCCGCACAAGCGGTGGAGCATGTGGTTTAATTCG
ATGCAACGCGAAGAACCTTACCTACTCTTGACATCCACGGAATTTGGCAGAGATGCCTTAGTGCCTTCGGGAACCGTGAGACAGGTGCTGCATGGCTGTC
GTCAGCTCGTGTTGTGAAATGTTGGGTTAAGTCCCGCAACGAGCGCAACCCTTATCCTTTGTTGCCAGCGATTCGGTCGGGAACTCAAAGGAGACTGCCG
GTGATAAACCGGAGGAANGGTGGGGATGACGTCAAGTCATCATGGCCCTTACGAGTAGGGCTACACACGTGCTACAATGGCGCATACAAAGAGAAGCGAC
CTCGCGAGAGCAAGCGGACCTCACAAAGTGCGTCGTAGTCCGGATCGGAGTCTGCAACTCGACTCCGTGAAGTCGGAATCGCTAGTAATCGTGGATCAGA
ATGCCACGGTGAATACGTTCCCGGGCCTTGTACACACCGCCCGTCACACCATGGGAGTGGGTTGCAAAAGAAGTAGGTAGCTTAACCTTCGGGAGGGCGC
TACC

>EB263
GTCGAGCGGTAGCACAGGGAGCTTGCTCCTGGGTGACGAGCGGCGGACGGGTGAGTAATGTCTGGGAAACTGCCTGATGGAGGGGGATAACTACTGGAAA
CGGTAGCTAATACCGCATAACGTCTTCGGACCAAAGAGGGGGACCTTCGGGCCTCTTGCCATCAGATGTGCCCAGATGGGATTAGCTAGTAGGTGAGGTA
ATGGCTCACCTAGGCGACGATCCCTAGCTGGTCTGAGAGGATGACCAGCCACACTGGAACTGAGACACGGTCCAGACTCCTACGGGAGGCAGCAGTGGGG
AATATTGCACAATGGGCGCAAGCCTGATGCAGCCATGCCGCGTGTATGAAGAAGGCCTTCGGGTTGTAAAGTACTTTCAGCGAGGAGGAAGGCATTGTGG
TTAATAACCGCAGTGATTGACGTTACTCGCAGAAGAAGCACCGGCTAACTCCGTGCCAGCAGCCGCGGTAATACGGAGGGTGCAAGCGTTAATCGGAATT
ACTGGGCGTAAAGCGCACGCAGGCGGTCTGTCAAGTCGGATGTGAAATCCCCGGGCTCAACCTGGGAACTGCATTCGAAACTGGCAGGCTAGAGTCTTGT
AGAGGGGGGTAGAATTCCAGGTGTAGCGGTGAAATGCGTAGAGATCTGGAGGAATACCGGTGGCGAAGGCGGCCCCCTGGACAAAGACTGACGCTCAGGT
GCGAAAGCGTGGGGAGCAAACAGGATTAGATACCCTGGTAGTCCACGCCGTAAACGATGTCGACTTGGAGGTTGTTCCCTTGAGGAGTGGCTTCCGGAGC
TAACGCGTTAAGTCGACCGCCTGGGGAGTACGGCCGCAAGGTTAAAACTCAAATGAATTGACGGGGGCCCGCACAAGCGGTGGAGCATGTGGTTTAATTC
GATGCAACGCGAAGAACCTTACCTACTCTTGACATCCAGAGAATTCGCCAGAGATGGCTTAGTGCCTTCGGGAACTCTGAGACAGGTGCTGCATGGCTGT
CGTCAGCTCGTGTTGTGAAATGTTGGGTTAAGTCCCGCAACGAGCGCAACCCTTATCCTTTGTTGCCAGCGGTTCGGCCGGGAACTCAAAGGAGACTGCC
AGTGATAAACTGGAGGAAGGTGGGGATGACGTCAAGTCATCATGGCCCTTACGAGTAGGGCTACACACGTGCTACAATGGCGCATACAAAGAGAAGCGAC
CTCGCGAGAGCAAGCGGACCTCATAAAGTGCGTCGTAGTCCGGATCGGAGTCTGCAACTCGACTCCGTGAAGTCGGAATCGCTAGTAATCGTAGATCAGA
ATGCTACGGTGAATACGTTCCCGGGCCTTGTACACACCTCCCGTCACACCATGGGAGTGGGTTGCAAAAGAAGTAGGTAGCTTAACCTTCGGGAGGGCG

>EB264
GTCGGACGGTAGCACAGAGAGCTTGCTCTCGGGTGACGAGTGGCGGACGGGTGAGTAATGTCTGGGGATCTGCCCGATAGAGGGGGATAACCACTGGAAA
CGGTGGCTAATACCGCATAACGTCGCAAGACCAAAGAGGGGGACCTTCGGGCCTCTCACTATCGGATGAACCCAGATGGGATTAGCTAGTAGGCGGGGTA
ATGGCCCACCTAGGCGACGATACCTAGCTGGTCTGAGAGGATGACCAGCCACACTGGAACTGAGACACGGTCCAGACTCCTACGGGAGGCAGCAGTGGGG
AATATTGCACAATGGGCGCAAGCCTGATGCAGCCATGCCGGCGTGTATGAAGAAGGCCTTTCGGGTTGTAAAGTACTTTCAGCGGGGAGGAAGGCGATGC
GGTTAATAACCCCGTCGATTGACGTTACCCGCAGAAGAAGCACCGGCTAACTCCGTGCCAGCAGCCGCGGTAATACGGAGGGTGCAAGCGTTAATCGGAA
TTACTGGGCGTAAAGCGCACGCAGGCGGTCTGTTAAGTCAGATGTGAAATCCCCGGGCTTAACCTGGGAACTGCATTTGAAACTGGCAGGCTTGAGTCTT
GTAGAGGGGGGTAGAATTCCAGGTGTAGCGGTGAAATGCGTAGAGATCTGGAGGAATACCGGTGGCGAAGGCGGCCCCCTGGACAAAGACTGACGCTCAG
GTGCGAAAGCGTGGGGAGCAAACAGGATTAGATACCCTGGTAGTCCACGCCGTAAACGATGTCGACTTGGAGGTTGTTCCCTTGAGGAGTGGCTTCCGGA
GCTAACGCGTTAAGTCGACCGCCTGGGGAGTACGGCCGCAAGGTTAAAACTCAAATGAATTGACGGGGGCCCGCACAAGCGGTGGAGCATGTGGTTTAAT
TCGATGCAACGCGAAGAACCTTACCTACTCTTGACATCCACGGAAATTTGTCAGAGATGCCTTATTGCCTTCGGGAACACTGAGACAGGTGCTGCATGGC
TGTCGTCAGCTCGTGTTGTGAAATGTTGGGTTAAGTCCCGCAACGAGCGCAACCCTTATCCTTTGTTGCCAGCGATTCGGTCGGGAACTCAAAGGAGACT
GCCGGTGATAAACCGGAGGAAGGTGGGGATGACGTCAAGTCATCATGGCCCTTACGAGTAGGGCTACACACGTGCTACAATGGCGCATACAAAGAGAAGC
GACCTCGCGAGAGCAAGCGGACCTCACAAAGTGCGTCGTAGTCCGGATCGGAGTCTGCAACTCGACTCCGTGAAGTCGGAATCGCTAGTAATCGTGGATC
AGAATGCCACGGTGAATACGTTCCCGGGCCTTGTACACACCGCCCGTCACACCATGGGAGTGGGTTGCAAAAGAAGTAGGTAGCTTAACCTTCGGGAGGG
CGCTA

>EB265
GACGTTGACGGCGGACGGGTGAGTAACACGTGGGCAACCTGCCTGTAAGACTGGGATAACTTCGGGAAACCGAAGCTAATACCGGATAGGATCTTCTCCT
TCATGGGAGATGATTGAAAGATGGTTTCGGCTATCACTTACAGATGGGCCCGCGGTGCATTAGCTAGTTGGTGAGGTAACGGCTCACCAAGGCAACGATG
CATAGCCGACCTGAGAGGGTGATCGGCCACACTGGGACTGAGACACGGCCCAGACTCCTACGGGAGGCAGCAGTAGGGAATCTTCCGCAATGGACGAAAG
TCTGACGGAGCAACGCCGCGTGAGTGATGAAGGCTTTCGGGTCGTAAAACTCTGTTGTTAGGGAAGAACAAGTACGAGAGTAACTGCTCGTACCTTGACG
GTACCTAACCAGAAAGCCACGGCTAACTACGTGCCAGCAGCCGCGGTAATACGTAGGTGGCAAGCGTTATCCGGAATTATTGGGCGTAAAGCGCGCGCAG
GCGGTTTCTTAAGTCTGATGTGAAAGCCCACGGCTCAACCGTGGAGGGTCATTGGAAACTGGGGAACTTGAGTGCAGAAGAGAAAAGCGGAATTCCACGT
GTAGCGGTGAAATGCGTAGAGATGTGGAGGAACACCAGTGGCGAAGGCGGCTTTTTGGTCTGTAACTGACGCTGAGGCGCGAAAGCGTGGGGAGCAAACA
GGATTAGATACCCTGGTAGTCCACGCCGTAAACGATGAGTGCTAAGTGTTAGAGGGTTTCCGCCCTTTAGTGCTGCAGCTAACGCATTAAGCACTCCGCC
TGGGGAGTACGGTCGCAAGACTGAAACTCAAAGGAATTGACGGGGGCCCGCACAAGCGGTGGAGCATGTGGTTTAATTCGAAGCAACGCGAAGAACCTTA
CCAGGTCTTGACATCCTCTGACAACTCTAGAGATAGAGCGTTCCCCTTCGGGGGACAGAGTGACAGGTGGTGCATGGTTGTCGTCAGCTCGTGTCGTGAG
ATGTTGGGTTAAGTCCCGCAACGAGCGCAACCCTTGATCTTAGTTGCCAGCATTTAGTTGGGCACTCTAAGGTGACTGCCGGTGACAAACCGGAGGAANG
GTGGGGATGACGTCAAATCATCATGCCCCTTATGACCTGGGCTACACACGTGCTACAATGGATGGTACAAAGGGCTGCAAGACCGCGAGGTCAAGCCAAT
CCCATAAAACCATTCTCAGTTCGGATTGTAGGCTGCAACTCGCCTACATGAAGCTGGAATCGTTAGTAATCGCGGATCAGCATGCCGCGGTGAATACGTT
CCCGGGCCTTGTACACACCGCCCGTCACACCACGAGAGTTTGTAACTAGAGAAGTCGGTGGAGTCAC

>EB266
CCCTCCCGAAGGTTAAGCTACCTACTTCTTTTGCAACCCACTCCCATGGTGTGACGGGCGGTGTGTACAAGGCCCGGGAACGTATTCACCGTGGCATTCT
GATCCACGATTACTAGCGATTCCGACTTCACGGAGTCGAGTTGCAGACTCCGATCCGGACTACGACGCACTTTGTGAGGTCCGCTTGCTCTCGCGAGGTC
GCTTCTCTTTGTATGCGCCATTGTAGCACGTGTGTAGCCCTACTCGTAAGGGCCATGATGACTTGACGTCATCCCCACCTTCCTCCGGTTTATCACCGGC
AGTCTCCTTTGAGTTCCCGACCGAATCGCTGGCAACAAAGGATAAGGGTTGCGCTCGTTGCGGGACTTAACCCAACATTTCACAACACGAGCTGACGACA
GCCATGCAGCACCTGTCTCAGAGTTCCCGAAGGCACCAAAGCATCTCTGCTAAGTTCTCTGGATGTCAAGAGTAGGTAAGGTTCTTCGCGTTGCATCGAA
TTAAACCACATGCTCCACCGCTTGTGCGGGCCCCCGTCAATTCATTTGAGTTTTAACCTTGCGGCCGTACTCCCCAGGCGGTCGACTTAACGCGTTAGCT
CCGGAAGCCACTCCTCAAGGGAACAACCTCCAAGTCGACATCGTTTACGGCGTGGACTACCAGGGTATCTAATCCTGTTTGCTCCCCACGCTTTCGCACC
TGAGCGTCAGTCTTTGTCCAGGGGGCCGCCTTCGCCACCGGTATTCCTCCAGATCTCTACGCATTTCACCGCTACACCTGGAATTCTACCCCCCTCTACA
AGACTCAAGCCTGCCAGTTTCAAATGCAGTTCCCAGGTTAAGCCCGGGGATTTCACATCTGACTTAACAGACCGCCTGCGTGCGCTTTACGCCCAGTAAT
TCCGATTAACGCTTGCACCCTCCGTATTACCGCGGCTGCTGGCACGGAGTTAGCCGGTGCTTCTTCTGCGGGTAACGTCAATCGGAGGCGGTTATTAACC
GCATCGCCTTCCTCCCCCGCTGAAAGTACTTTACAACCCGAAGGCCTTCTTCATACACGCGGCATGGCTGCATCAGGCTTGCGCCCATTGTGCAATATTC
CCCACTGCTGCCTCCCGTAGGAGTCTGGACCGTGTCTCAGTTCCAGTGTGGCTGGTCATCCTCTCAGACCAGCTAGGGATCGTCGCCTAGGTGGGCCATT
ACCCCGCCTACTAGCTAATCCCATCTGGGTTCATCCGATAGTGAGAGGCCCGAAGGTCCCCCTCTTTGGTCTTGCGACGTTATGCGGTATTAGCCACCGT
TTCCAGTGGTTATCCCCCTCTATCGGGCAGATCCCCAGACATTACTCACCCGTCCGCCACTCGTCACCCAAGGAGCAAGCTCCTCTGTGCTACCGTCCGA
CTTGCA

>EB267
TCGAGCGAGGACTTCGGTTCTAGCGGCGGACGGGTGAGTAACGCGTGGGAACGTGCCCTTTGCTACGGAATAGCCCCGGGAAACTGGGAGTAATACCGTA
TGTGCCCATTGCCCTTGGCGGGTATTGGGGAAAGATTTATCGGCAAAGGATCGGCCCGCGTTGGATTAGGTAGTTGGTGGGGTAATGGCCTACCAAGCCG
ACGATCCATAGCTGGTTTGAGAGGATGATCAGCCACACTGGGACTGAGACACGGCCCAGACTCCTACGGGAGGCAGCAGTGGGGAATCTTAGACAATGGG

GGCAACCCTGATCTAGCCATGCCGCGTGAGTGATGAAGGCCTTAGGGTTGTAAAGCTCTTTCAGCTGGGAAGATAATGACGGTACCAGCAGAAGAAGCCC
CGGCTAACTCCGTGCCAGCAGCCGCGGTAATACGGAGGGGGCTAGCGTTGTTCGGAATTACTGGGCGTAAAGCGCACGTAGGCGGACTGGAAAGTTGGGG
GTGAAATCCCGGGGCTCAACCTCGGAACTGCCTTCAAAACTATCGGTCTGGAGTTCGAGAGAGGTGAGTGGAATTCCGAGTGTAGAGGTGAAATTCGTAG
ATATTCGGAGGAACACCAGTGGCGAAGGCGGCTCACTGGCTCGATACTGACGCTGAGGTGCGAAAGCGTGGGGAGCAAACAGGATTAGATACCCTGGTAG
TCCACGCCGTAAACGATGAATGCCAGACGTCGGCAAGCATGCTTGTCGGTGTCACACCTAACGGATTAAGCATTCCGCCTGGGGAGTACGGTCGCAAGAT
TAAAACTCAAAGGAATTGACGGGGGCCCGCACAAGCGGTGGAGCATGTGGTTTAATTCGAAGCAACGCGCAGAACCTTACCAACCCTTGACATCCCTGGA
CCGGTCCGGAGACGGATCTTTCACTTCGGTGACCAGGAGACAGGTGCTGCATGGCTGTCGTCAGCTCGTGTCGTGAGATGTTCGGTTAAGTCCGGCAACG
AGCGCAACCCACGTCCCCAGTTGCCAGCATTCAGTTGGGCACTCTGTGGAAACTGCCGGTGATAAGCCGGAGGAAGGTGTGGATGACGTCAAGTCCTCAT
GGCCCTTACGGGTTGGGCTACACACGTGCTACAATGGTGGTGACAGTGGGTTAATCCCCAAAAGCCATCTCAGTTCGGATTGTCCTCTGCAACTCGAGGG
CATGAAGTTGGAATCGCTAGTAATCGCGGAACAGCATGCCGCGGTGAATACGTTCCCGGGCCTTGTACACACCGCCCGTCACACCATGGGAGTTGGTTCT
ACCCGACGACGCTGCGCTAACCCGCAAGGGAGGCAGGC

>EB268
TAGCGCACGGCCGTCGGGTAGACCCAACTCCCATGGTGTGACGGGCGGTGTGTACAAGGCCCGGGAACGTATTCACCGCGGCATGCTGTTCCGCGATTAC
TAGCGATTCCAACTTCATGGGGTCGAGTTGCAGACCCCAATCCGAACTGAGATGGCTTTTGGGGATTAACCCACTGTCACCACCATTGTAGCACGTGTGT
AGCCCAACCCGTAAGGGCCATGAGGACTTGACGTCATCCACACCTTCCTCCGGCTTATCACCGGCAGTTTCCACAGAGTGCCCCAACTGAATGCTGGCAA
CTGGGGACGTGGGTTGCGCTCGTTGCCGGACTTAACCGAACATCTCACGACACGAGCTGACGACAGCCCATGCAGCACCTGTCTTCCAGGCCACCGAAGT
GGAAAGCCCCATCTCTGGGGATGTCCTGGGATGTCAAGGGTTGGTAAGGTTCTGCGCGTTGCTTCGAATTAAACCACATGCTCCACCGCTTGTGCGGGCC
CCCGTCAATTCCTTTGAGTTTTAATCTTGCGACCGTACTCCCCAGGCGGAATGCTTAATCCGTTAGGTGTGTCACCGAATTGCATGCAACCCGACGACTG
GCATTCATCGTTTACGGCGTGGACTACCAGGGTATCTAATCCTGTTTGCTCCCCACGCTTTCGCACCTCAGCGTCAGTATCGAGCCAGTGAGCCGCCTTC
GCCACTGGTGTTCCTCCGAATATCTACGAATTTCACCTCTACACTCGGAATTCCACTCACCTCTCTCGAACTCCAGACCGATAGTTTTGAAGGCAGTTCC
GAGGTTGAGCCCCGGGATTTCACCCCCAACTTTCCGGTCCGCCTACGTGCGCTTTACGCCCAGTAATTCCGAACAACGCTAGCCCCCTCCGTATTACCGC
GGCTGCTGGCACGGAGTTAGCCGGGGCTTCTTCTGCTGGTACCGTCATTATCTTCCCAGCTGAAAGAGCTTTACAACCCTAAGGCCTTCATCACTCACGC
GGCATGGCTAGATCAGGGTTGCCCCCATTGTCTAAGATTCCCCACTGCTGCCTCCCGTAGGAGTCTGGGCCGTGTCTCAGTCCCAGTGTGGCTGATCATC
CTCTCAAACCAGCTATGGATCGTCGGCTTGGTAGGCCATTACCCCACCAACTACCTAATCCAACGCGGGCTAATCCTTCCCCGATAAATCTTTCCCCCAA
AGGGCGTATGCGGTATTACTCTCAGTTTCCCGAGGCTATTCCGCAGAGAAGGGCATATTCCCACGCGTTACTCACCCGTCCGCCGCTAACCCCGAAGGGT
CCGCTCGACTGCATGTGTAG

>EB269
CGGGAACGTATTCACCGCGGCATGCTGATCCGCGATTACTAACGATTCCAGCTTCATGTAGGCGAGTTGCAGCCTACAATCCGAACTGAGAATGGTTTTA
TGGGATTGGCTTGACCTCGCGGTCTTGCAGCCCTTTGTACCATCCATTGTAGCACGTGTGTAGCCCAGGTCATAAGGGGCATGATGATTTGACGTCATCC
CCACCTTCCTCCGGTTTGTCACCGGCAGTCACCTTAGAGTGCCCAACTAAATGCTGGCAACTAAGATCAAGGGTTGCGCTCGTTGCGGGACTTAACCCAA
CATCTCACGACACGAGCTGACGACAACCATGCACCACCTGTCACTCTGTCCCCCGAAGGGGAACGCTCTATCTCTAGAGTTGTCAGAGGATGTCAAGACC
TGGTAAGGTTCNTTCGCGTTGCTTCGAATTAAACCACATGCTCCACCGCTTGTGCGGGCCCCCGTCAATTCCTTTGAGTNTTCAGTCTTGCGACCGTACT
NCCCCAGNGCGGAGTGCTTAATGCGTTAGCTGCAGCACTNAAANGGGCGGAAACCCTCTAACACTNTAGCACTNCATCGTNTTACNGGCGTGNGACTACC
NANGGGTATCTAATCNCTGTTTNGCTCCNCCACNGCTTTNCGCGCCTNCAGCGTCAGTTACAGACCAAAAAGCCGCCTTCGCCACTGGTGTTCCTCCACA
TCTCTACGCATTTCACCGCTACACGTGGAATTCCGCTTTTCTCTTCTGCACTCAAGTTCCCCAGTTTCCAATGACCCTCCACGGTTGAGCCGTGGGCTTT
CACATCAGACTTAAGAAACCGCCTGCGCGCGCTTTACGCCCAATAATTCCGGATAACGCTTGCCACCTACGTATTACCGCGGCTGCTGGCACGTAGTTAG
CCGTGGCTTTCTGGTTAGGTACCGTCAAGGTACGAGCAGTTACTCTCGTACTTGTTCTTCCCTAACAACAGAGTTTTACGACCCGAAAGCCTTCATCACT
CACGCGGCGTTGCTCCGTCAGACTTTCGTCCATTGCGGAAGATTCCCTACTGCTGCCTCCCGTAGGAATCTGGGCCGTGTCTCAGTCCCAGTGTGGCCGA
TCACCCTCTCAGGTCGGCTATGCATCCTTGCCTTGGTGAACCGTTACCT

>EB270
GTCGAGCGGATTGATGGGAGCTTGCTCCCTGATATCAGCGGCGGACGGGTGAGTAACACGTGGGCAACCTGCCCTGCAGATGGGGATAACTCCGGGAAAC
CGGGGCTAATACCGAATAATCGGTTCTTCCGCATGGAAGAACTCTGAAAGACGGTTTCGGCTGTCACTGCAGGATGGGCCCGCGGCGCATTAGCTAGTTG
GTGGGGTAATGGCCTACCAAGGCGACGATGCGTAGCCGACCTGAGAGGGTGATCGGCCACACTGGGACTGAGACACGGCCCAGACTCCTACGGGAGGCAG
CAGTAGGGAATCTTCCACAATGGACGAAAGTCTGATGGAGCAACGCCGCGTGAGCGAAGAAGGTTTTCGGATCGTAAAGCTCTGTTGCGAGGGAAGAACA
AGTACGGGAGTAACTGCCCGTACCATGACGGTACCTCGTCAGAAAGCCACGGCTAACTACGTGCCAGCAGCCGCGGTAATACGTAGGTGGCAAGCGTTGT
CCGGAATTATTGGGCGTAAAGCGCGCGCAGGCGGTCCTTTAAGTCTGATGTGAAAGCCCACGGCTCAACCGTGGAGGGTCATTGGAAACTGGAGGACTTG
AGTACAGAAGAGGAAAGCGGAATTCCACGTGTAGCGGTGAAATGCGTAGAGATGTGGAGGAACACCAGTGGCGAAGGCGGCTTTCTGGTCTGTAACTGAC
GCTGAGGCGCGAAAGCGTGGGGAGCAAACAGGATTAGATACCCTGGTAGTCCACGCCGTAAACGATGAGTGCTAAGTGTTAGGGGGTTTCCGCCCCTTAG
TGCTGCAGCTAACGCATTAAGCACTCCGCCTGGGGAGTACGGCCGCAAGGCTGAAACTCAAAGGAATTGACGGGGACCCGCACAAGCGGTGGAGCATGTG
GTTTAATTCGAAGCAACGCGAAGAACCTTACCAGGTCTTGACATCCCGCTGACCGGCATGGAGACATGTCTTTCCCTTCGGGGACAGCGGTGACAGGTGG
TGCATGGTTGTCGTCAGCTCGTGTCGTGAGATGTTGGGTTAAGTCCCGCAACGAGCGCAACCCTTGATCTTAGTTGCCAGCATTCAGTTGGGCACTCTAA
GGTGACTGCCGGTGACAAACCGGAGGAAGGTGGGGATGACGTCAAATCATCATGCCCCTTATGACCTGGGCTACACACGTGCTACAATGGACGATACAAA
GGGCTGCAAACCCGCGAGGGGGAGCCAATCCCATAAAATCGTTCCCAGTTCGGATTGCAGGCTGCAACTCGCCTGCATGAAGCCGGAATCGCTAGTAATC
GTGGATCAGCATGCCACGGTGAATACGTTCCCGGGTCTTGTACACACCGCCCGTCACACCACGAGAGTTTGTAACACCTCGAAGTCGGTGGGGTAACCCT
TACGGGAGCCAGCCGCCG

>EB271
CCGTGAATAGTTTGATTATGACTCATGAAGTCGCGTGGGGAACCTGCCTGTTTTACTGGTATAACTCCGGGAAACCGGGGCTAATACCGGATGGTTGTTT
GAACCGCATGGTTCAGACATAAAAGGTGGCTTCGGCTACCACTTACAGATGGACCCGCGGCGCATTAGCTAGTTGGTGAGGTAACGGCTCACCAAGGCAA
CGATGCGTAGCCGACCTGAGAGGGTGATCGGCCACACTGGGACTGAGACACGGCCCAGAYTCCTACGGGAGGCAGCAGTAGGGAATCTTCCGCAATGGAC
GAAAGTCTGACGGAGCAACGCCGCGTGAGTGATGAAGGTTTTCGGATCGTAAAGCTCTGTTGTTAGGGAAGAACAAGTGCCGTTCAAATAGGGCGGCACC
TTGACGGTACCTAACCAGAAAGCCACGGCTAACTACGTGCCAGCAGCCGCGGTAATACGTAGGTGGCAAGCGTTGTCCGGAATTATTGGGCGTAAAGGGC
TCGCAGGCGGTTTCTTAAGTCTGATGTGAAAGCCCCCGGCTCAACCGGGGAGGGTCATTGGAAACTGGGGAACTTGAGTGCAGAAGAGGAGAGTGGAATT
CCACGTGTAGCGGTGAAATGCGTAGAGATGTGGAGGAACACCAGTGGCGAAGGCGACTCTCTGGTCTGTAACTGACGCTGAGGAGCGAAAGCGTGGGGAG
CGAACAGGATTAGATACCCTGGTAGTCCACGCCGTAAACGATGAGTGCTAAGTGTTAGGGGGTTTCCGCCCCTTAGTGCTGCAGCTAACGCATTAAGCAC
TCCGCCTGGGGAGTACGGTCGCAAGACTGAAACTCAAAGGAATTGACGGGGGCCCGCACAAGCGGTGGAGCATGTGGTTTAATTCGAAGCAACGCGAAGA
ACCTTACCAGGTCTTGACATCCTCTGACAATCCTAGAGATAGGACGTCCCCTTCGGGGGCAGAGTGACAGGTGGTGCATGGTTGTCGTCAGCTCGTGTCG
TGAGATGTTGGGNTTAAGTCCCGCAACGAGCGCAACCCTTGATCTTAGTTGCCAGCATTCAGTTGGGCACTCTAAGGTGACTGCCGGTGACAAACCGGAG
GAAGGTGGGGATGACGTCAAATCATCATGCCCCTTATGACCTGGGCTACACACGTGCTACAATGGGCAGAACAAAGGGCAGCGAAACCGCGAGGTTAAGC
CAATCCCACAAATCTGTTCTCAGTTCGGATCGCAGTCTGCAACTCGACTGCGTGAAGCAGGAATCGCTAGTAATCGCGGATCAGCATGCCGCGGTGAATA
CGTTCCCGGGCCTGGTACACACCGCCCCTCTCACCA

>EB272
TCAGGACGAACGCTGGCGGCGTGCTAATACATGCAAGTCGAGCGGACAGATGGGAGCTTGCTCCCTGATGTTAGCGGCGGACGGGTGAGTAACACGTGGG
TAACCTGCCTGTAAGACTGGGATAACTCCGGGAAACCGGGGCTAATACCGGATGGTTGTTTGAACCGCATGGTTCAGACATAAAAGGTGGCTTCGGCTAC

CACTTACAGATGGACCCGCGGCGCATTAGCTAGTTGGTGAGGTAACGGCTCACCAAGGCAACGATGCGTAGCCGACCTGAGAGGGTGATCGGCCACACTG
GGACTGAGACACGGCCCAGACTCCTACGGGAGGCAGCAGTAGGGAATCTTCCGCAATGGACGAAAGTCTGACGGAGCAACGCCGCGTGAGTGATGAAGGT
TTTCGGATCGTAAAGCTCTGTTGTTAGGGAAGAACAAGTGCCGTTCAAATAGGGCGGCACCTTGACGGTACCTAACCAGAAAGCCACGGCTAACTACGTG
CCAGCAGCCGCGGTAATACGTAGGTGGCAAGCGTTGTCCGGAATTATTGGGCGTAAAGGGCTCGCAGGCGGTTTCTTAAGTCTGATGTGAAAGCCCCCGG
CTCAACCGGGGAGGGTCATTGGAAACTGGGGAACTTGAGTGCAGAAGAGGAGAGTGGAATTCCACGTGTAGCGGTGAAATGCGTAGAGATGTGGAGGAAC
ACCAGTGGCGAAGGCGACTCTCTGGTCTGTAACTGACGCTGAGGAGCGAAAGCGTGGGGAGCGAACAGGATTAGATACCCTGGTAGTCCACGCCGTAAAC
GATGAGTGCTAAGTGTTAGGGGGTTTCCGCCCCTTAGTGCTGCAGCTAACGCATTAAGCACTCCGCCTGGGGAGTACGGTCGCAAGACTGAAACTCAAAG
GAATTGACGGGGGCCCGCACAAGCGGTGGAGCATGTGGTTTAATTCGAAGCAACGCGAAGAACCTTACCAGGTCTTGACATCCTCTGACAATCCTAGAGA
TAGGACGTCCCCTTCGGGGGCAGAGTGACAGGTGGTGCATGGTTGTCGTCAGCTCGTGTCGTGAGATGTTGGGTTAAGTCCCGCAACGAGCGCAACCCTT
GATCTTAGTTGCCAGCATTCAGTTGGGCACTCTAAGGTGACTGCCGGTGACAAACCGGAGGAAGGTGGGGATGACGTCAAATCATCATGCCCCTTATGA
CCTGGGCTACACACGTGCTACAATGGACAGAACAAAGGGCAGCGAAACCGCGAGGTTAAGCCAATCCCACAAATCTGTTCTCAGTTCGGATCGCAGTCTG
CAACTCGACTGCGTGAAGCTGGAATCGCTAGTAATCGCGGATCAGCATGCCGCGGTGAATACGTTCCCGGGCCTTGTACACACCGCCCGTCACACCACGA
GAGTTTGTAACACCCGAAGTCGGTGAGGTAACCTTTTAGGAACCAGCCCCCCAAAGTGGGACAGATGATTGGGGTGAAT

>EB273
GTTTCGGCGGACGGGTGAGTAACACGTGGGTAACCTGCCTGTAAGACTGGGATAACTCCGGGAAACCGGGGCTAATACCGGATGGTTGTTTGAACCGCAT
GGTTCAGACATAAAAGGTGGCTTCGGCTACCACTTACAGATGGACCCGCGGCGCATTAGCTAGTTGGTGAGGTAACGGCTCACCAAGGCAACGATGCGTA
GCCGACCTGAGAGGGTGATCGGCCACACTGGGACTGAGACACGGCCCAGACTCCTACGGGAGGCAGCAGTAGGGAATCTTCCGCAATGGACGAAAGTCTG
ACGGAGCAACGCCGCGTGAGTGATGAAGGTTTTCGGATCGTAAAGCTCTGTTGTTAGGGAAGAACAAGTGCCGTTCAAATAGGGCGGCACCTTGACGGTA
CCTAACCAGAAAGCCACGGCTAACTACGTGCCAGCAGCCGCGGTAATACGTAGGTGGCAAGCGTTGTCCGGAATTATTGGGCGTAAAGGGCTCGCAGGCG
GTTTCTTAAGTCTGATGTGAAAGCCCCCGGCTCAACCGGGGAGGGTCATTGGAAACTGGGGAACTTGAGTGCAGAAGAGGAGAGTGGAATTCCACGTGTA
GCGGTGAAATGCGTAGAGATGTGGAGGAACACCAGTGGCGAAGGCGACTCTCTGGTCTGTAACTGACGCTGAGGAGCGAAAGCGTGGGGAGCGAACAGGA
TTAGATACCCTGGTAGTCCACGCCGTAAACGATGAGTGCTAAGTGTTAGGGGGTTTCCGCCCCTTAGTGCTGCAGCTAACGCATTAAGCACTCCGCCTGG
GGAGTACGGTCGCAAGACTGAAACTCAAAGGAATTGACGGGGGCCCGCACAAGCGGTGGAGCATGTGGTTTAATTCGAAGCAACGCGAAGAACCTTACCA
GGTCTTGACATCCTCTGACAATCCTAGAGATAGGACGTCCCCTTCGGGGGCAGAGTGACAGGTGGTGCATGGTTGTCGTCAGCTCGTGTCGTGAGATGTT
GGGTTAAGTCCCGCAACGAGCGCAACCCTTGATCTTAGTTGCCAGCATTCAGTTGGGCACTCTAAGGTGACTGCCGGTGACAAACCGGAGGAAGGTGGGG
NATGACGTCAAATCATCATGCCCCTTATGACCTGGGCTACACACGTGCTACAATGGGCAGAACAAAGGGCAGCGAAACCGCGAGGTTAAGCCAATCCCAC
AAATCTGTTCTCAGTTCGGATCGCAGTCTGCAACTCGACTGCGTGAAGCTGGAATCGCTAGTAATCGCGGATCAGCATGCCGCGGTGAATACGTTCCCGG
TCCTTGTACACACCGCCCCTCACACCA

>EB274
ACCGTCCCCCCGAAGGTTAGACTAGCTACTTCTGGAGCAACCCACTCCCATGGTGTGACGGGCGGTGTGTACAAGGCCCGGGAACGTATTCACCGTGACA
TTCTGATTCACGATTACTAGCGATTCCGACTTCACGCAGTCGAGTTGCAGACTGCGATCCGGACTACGATCGGTTTTATGGGATTAGCTCCACCTCGCGG
CTTGGCAACCCTTTGTACCGACCATTGTAGCACGTGTGTAGCCCTGGCCGTAAGGGCCATGATGACTTGACGTCATCCCCACCTTCCTCCGGTTTGTCAC
CGGCAGTCTCCTTAGAGTGCCCACCATTACGTGCTGGTAACTAAGGACAAGGGTTGCGCTCGTTACGGGACTTAACCCAACATCTCACGACACGAGCTGA
CGACAGCCATGCAGCACCTGTGTCTGAGTTCCCGAAGGCACCAATCCATCTCTGGAAAGTTCTCAGCATGTCAAGGCCAGGTAAGGTTCTTCGCGTTGCT
TCGAATTAAACCACATGCTCCACCGCTTGTGCGGGCCCCCGTCAATTCATTTGAGTTTTAACCTTGCGGCCGTACTCCCCAGGCGGTCAACTTAATGCGT
TAGCTGCGCCACTAAGTTCTCAAGGAACCCAACGGCTAGTTGACATCGTTTACGGCGTGGACTACCAGGGTATCTAATCCTGTTTGCTCCCCACGCTTTC
GCACCTCAGTGTCAGTATCAGTCCAGGTGGTCGCCTTCGCCACTGGTGTTCCTTCCTATATCTACGCATTTCACCGCTACACAGGAAATTCCACCACCCT
CTACCGTACTCTAGCTCGCCAGTTTTGGATGCAGTTCCCAGGTTGAGCCCGGGGCTTTCACATCCAACTTAACGAACCACCTACGCGCGCTTTACGCCCA
GTAATTCCGATTAACGCTTGCACCCTTCGTATTACCGCGGCTGCTGGCACGAAGTTAGCCGGTGCTTATTCTGTCGGTAACGTCAAAACACTAACGTATT
AGGTTAATGCCCTTCCTCCCAACTTAAAGTGCTTTACAATCCGAAGACCTTCTTCACACACGCGGCATGGCTGGATCAGGCTTTCGCCCATTGTCCAATA
TTCCCCACTGCTGCCTCCCGTAGGAGTCTGGACCGTGTCTCAGTTCCAGTGTGACTGATCATCCTCTCAGACCAGTTACGGATCGTCGCCTTGGTGAGCC
ATTACCTCACCAACTAGCTAATCCGACCTAGGCTCATCTGATAGCGCAAGGCCCGAAGGTCCCCTGCTTTCTCCCGTAGGACGTATGCGGTATTAGCGTT
CCTTTCGGAACGTTATCCCCCACTACCAGGCAGATTCCTAGGCATTACTCACCCGTCCGCCGCTAAATCAAGGAGCAAGCTCCTCTCATCCGCTCGACTT
GCA

>EB275
TGCAGTCGAGCGGTAGAGAGAAGCTTGCTTCTCTTGAGAGCGGCGGACGGGTGAGTAATGCCTAGGAATCTGCCTGGTAGTGGGGGATAACGTTCGGAAA
CGAACGCTAATACCGCATACGTCCTACGGGAGAAAGCAGGGGACCTTCGGGCCTTGCGCTATCAGATGAGCCTAGGTCGGATTAGCTAGTTGGTGAGGTA
ATGGCTCACCAAGGCGACGATCCGTAACTGGTCTGAGAGGATGATCAGTCACACTGGAACTGAGACACGGTCCAGACTCCTACGGGAGGCAGCAGTGGGG
AATATTGGACAATGGGCGAAAGCCTGATCCAGCCATGCCGCGTGTGTGAAGAAGGTCTTCGGATTGTAAAGCACTTTAAGTTGGGAGGAAGGGCAATTAC
CTAATACGTGATTGTTTTGACGTTACCGACAGAATAAGCACCGGCTAACTCTGTGCCAGCAGCCGCGGTAATACAGAGGGTGCAAGCGTTAATCGGAATT
ACTGGGCGTAAAGCGCGCGTAGGTGGTTTGTTAAGTTGGATGTGAAATCCCCGGGCTCAACCTGGGAACTGCATTCAAAACTGACTGACTAGAGTATGGT
AGAGGGTGGTGGAATTTCCTGTGTAGCGGTGAAATGCGTAGATATAGGAAGGAACACCAGTGGCGAAGGCGACCACCTGGACTAATACTGACACTGAGGT
GCGAAAGCGTGGGGAGCAAACAGGATTAGATACCCTGGTAGTCCACGCCGTAAACGATGTCAACTAGCCGTTGGAAGCCTTGAGCTTTTAGTGGCGCAGC
TAACGCATTAAGTTGACCGCCTGGGGAGTACGGCCGCAAGGTTAAAACTCAAATGAATTGACGGGGGCCCGCACAAGCGGTGGAGCATGTGGTTTAATTC
GAAGCAACGCGAAGAACCTTACCAGGCCTTGACATCCAATGAACTTTCCAGAGATGGATTGGTGCCTTCGGGAACATTGAGACAGGTGCTGCATGGCTGT
CGTCAGCTCGTGTCGTGAGATGTTGGGTTAAGTCCCGTAACGAGCGCAACCCTTGTCCTTAGTTACCAGCACGTAATGGTGGGCACTCTAAGGAGACTGC
CGGTGACAAACCGGAGGAAGGTGGGGATGACGTCAAGTCATCATGGCCCTTACGGCCTGGGCTACACACGTGCTACAATGGTCGGTACAGAGGGTTGCCA
AACCGCGAGGTGGAGCTAATCCCATAAAACCGATCGTAGTCCGGATCGCAGTCTGCAACTCGACTGCGTGAAGTCGGAATCGCTAGTAATCGCGAATCAG
AATGTCGCGGTGAATACGTTCCCGGGCCTTGTACACACCGCCCGTCACACCATGGGAGTGGGTTGCACCAGAAGTAGCTAGTCTAACCTTCGGGAGGACG
GT

>EB276
TGCAAGTCGAGCGGATGAAAGGAGCTTGCTCCTGGATTCAGCGGCGGACGGGTGAGTAATGCCTAGGAATCTGCCTGGTAGTGGGGGACAACGTTTCGAA
AGGAACGCTAATACCGCATACGTCCTACGGGAGAAAGCAGGGGACCTTCGGGCCTTGCGCTATCAGATGAGCCTAGGTCGGATTAGCTAGTTGGTGAGGT
AATGGCTCACCAAGGCGACGATCCGTAACTGGTCTGAGAGGATGATCAGTCACACTGGAACTGAGACACGGTCCAGACTCCTACGGGAGGCAGCAGTGGG
GAATATTGGACAATGGGCGAAAGCCTGATCCAGCCATGCCGCGTGTGTGAAGAAGGTCTTCGGATTGTAAAGCACTTTAAGTTGGGAGGAAGGGTTGTAG
ATTAATACTCTGCAATTTTGACGTTACCGACAGAATAAGCACCGGCTAACTCTGTGCCAGCAGCCGCGGTAATACAGAGGGTGCAAGCGTTAATCGGAAT
TACTGGGCGTAAAGCGCGCGTAGGTGGTTTGTTAAGTTGGATGTGAAATCCCCGGGCTCAACCTGGGAACTGCATCCAAAACTGGCAAGCTAGAGTATGG
TAGAGGGTGGTGGAATTTCCTGTGTAGCGGTGAAATGCGTAGATATAGGAAGGAACACCAGTGGCGAAGGCGACCACCTGGACTGATACTGACACTGAGG
TGCGAAAGCGTGGGGAGCAAACAGGATTAGATACCCTGGTAGTCCACGCCGTAAACGATGTCAACTAGCCGTTGGGAGCCTTGAGCTCTTAGTGGCGCAG
CTAACGCATTAAGTTGACCGCCTGGGGAGTACGGCCGCAAGGTTAAAACTCAAATGAATTGACGGGGGCCCGCACAAGCGGTGGAGCATGTGGTTTAATT
CGAAGCAACGCGAAGAACCTTACCAGGCCTTGACATCCAATGAACTTTCCAGAGATGGATTGGTGCCTTCGGGAACATTGAGACAGGTGCTGCATGGCTG
TCGTCAGCTCGTGTCGTGAGATGTTGGGTTAAGTCCCGTAACGAGCGCAACCCTTGTCCTTAGTTACCAGCACGTTATGGTGGGCACTCTAAGGAGACTG
CCGGTGACAAACCGGAGGAAGGTGGGGATGACGTCAAGTCATCATGGCCCTTACGGCCTGGGCTACACACGTGCTACAATGGTCGGTACAGAGGGTTGCC
AAGCCGCGAGGTGGAGCTAATCCCACAAAACCGATCGTAGTCCGGATCGCAGTCTGCAACTCGACTGCGTGAAGTCGGAATCGCTAGTAATCGCGAATCA

GAATGTCGCGGTGAATACGTTCCCGGGCCTTGTACACACCGCCCGTCACACCATGGGAGTGGGTTGCACCAGAAGTAGCTAGTCTAACCTTCGGGAGGAC
GGT

>EB277
ACCGTCCTCCCGAAGGTTAGACTAGCTACTTCTGGTGCAACCCACTCCCATGGTGTGACGGGCGGTGTGTACAAGGCCCGGGAACGTATTCACCGCGACA
TTCTGATTCGCGATTACTAGCGATTCCGACTTCACGCAGTCGAGTTGCAGACTGCGATCCGGACTACGATCGGTTTTGTGAGATTAGCTCCACCTCGCGG
CTTGGCAACCCTCTGTACCGACCATTGTAGCACGTGTGTAGCCCAGGCCGTAAGGGCCATGATGACTTGACGTCATCCCCACCTTCCTCCGGTTTGTCAC
CGGCAGTCTCCTTAGAGTGCCCACCATAACGTGCTGGTAACTAAGGACAAGGGTTGCGCTCGTTACGGGACTTAACCCAACATCTCACGACACGAGCTGA
CGACAGCCATGCAGCACCTGTGTCAGAGTTCCCGAAGGCACCAATCCATCTCTGGAAAGTTCTCTGCATGTCAAGGCCTGGTAAGGTTCTTCGCGTTGCT
TCGAATTAAACCACATGCTCCACCGCTTGTGCGGGCCCCCGTCAATTCATTTGAGTTTTAACCTTGCGGCCGTACTCCCCAGGCGGTCAACTTAATGCGT
TAGCTGCGCCACTAAAATCTCAAGGATTCCAACGGCTAGTTGACATCGTTTACGGCGTGGACTACCAGGGTATCTAATCCTGTTTGCTCCCCACGCTTTC
GCACCTCAGTGTCAGTATCAGTCCAGGTGGTCGCCTTCGCCACTGGTGTTCCTTCCTATATCTACGCATTTCACCGCTACACAGGAAATTCCACCACCCT
CTACCGTACTCTAGCTTGCCAGTTTTGGATGCAGTTCCCAGGTTGAGCCCGGGGCTTTCACATCCAACTTAACAAACCACCTACGCGCGCTTTACGCCCA
GTAATTCCGATTAACGCTTGCACCCTCTGTATTACCGCGGCTGCTGGCACAGAGTTAGCCGGTGCTTATTCTGTCGGTAACGTCAAAATTGCAGAGTATT
AATCTACAACCCTTCCTCCCAACTTAAAGTGCTTTACAATCCGAAGACCTTCTTCACACACGCGGCATGGCTGGATCAGGCTTTCGCCCATTGTCCAATA
TTCCCCACTGCTGCCTCCCGTAGGAGTCTGGACCGTGTCTCAGTTCCAGTGTGACTGATCATCCTCTCAGACCAGTTACGGATCGTCGCCTTGGTGAGCC
ATTACCTCACCAACAAGCTAATCCGACCTAGGCTCATCTGATAGCGCAAGGCCCGAAGGTCCCCTGCTTTCTCCCGTAGGACGTATGCGGTATTAGCGTT
CCTTTCGAAACGTTGTCCCCCACTACCAGGCAGATTCCTAGGCATTACTCACCCGTCCGCCGCTGAATCAAGGAGCAAGCTCCCTGTCATCCGCTCGACT
GC

>EB278
TGCAAGTCGAGCGAATTGATGGGAGCTTGCTCCCTGATATTAGCGGCGGACGGGTGAGTAACACGTGGGCAACCTGCCCTGCAGATGGGGATAACTCCGG
GAAACCGGGGCTAATACCGAATAATCAGTTCCTCCGCATGGAGGAACTCTGAAAGACGGTTTCGGCTGTCACTGCAGGATGGGCCCGCGGCGCATTAGCT
AGTTGGTGGGGTAACGGCCTACCAAGGCGACGATGCGTAGCCGACCTGAGAGGGTGATCGGCCACACTGGGACTGAGACACGGCCCAGACTCCTACGGGA
GGCAGCAGTAGGGAATCTTCCACAATGGACGAAAGTCTGATGGAGCAACGCCGCGTGAGCGAAGAAGGTTTTCGGATCGTAAAGCTCTGTTGCGAGGGAA
GAACAAGTACGGGAGTAACTGCCCGTACCTTGACGGTACCTCGTCAGAAAGCCACGGCTAACTACGTGCCAGCAGCCGCGGTAATACGTAGGTGGCAAGC
GTTGTCCGGAATTATTGGGCGTAAAGCGCGCGCAGGCGGTCCTTTAAGTCTGATGTGAAAGCCCACGGCTCAACCGTGGAGGGTCATTGGAAACTGGAGG
ACTTGAGTACAGAAGAGGAAAGCGGAATTCCACGTGTAGCGGTGAAATGCGTAGAGATGTGGAGGAACACCAGTGGCGAAGGCGGCTTTCTGGTCTGTAA
CTGACGCTGAGGCGCGAAAGCGTGGGGAGCAAACAGGATTAGATACCCTGGTAGTCCACGCCGTAAACGATGAGTGCTAAGTGTTAGGGGGTTTCCGCCC
CTTAGTGCTGCAGCTAACGCATTAAGCACTCCGCCTGGGGAGTACGGCCGCAAGGCTGAAACTCAAAGGAATTGACGGGGACCCGCACAAGCGGTGGAGC
ATGTGGTTTAATTCGAAGCAACGCGAAGAACCTTACCAGGTCTTGACATCCCGCTGACCGGTGTAGAGATACGCCTTTCCCTTCGGGGACAGCGGTGACA
GGTGGTGCATGGTTGTCGTCAGCTCGTGTCGTGAGATGTTGGGTTAAGTCCCGCAACGAGCGCAACCCTTGATCTTAGTTGCCAGCATTCAGTTGGGCAC
TCTAAGGTGACTGCCGGTGACAAACCGGAGGAAGGTGGGGATGACGTCAAATCATCATGCCCCTTATGACCTGGGCTACACACGTGCTACAATGGACGGT
ACAAAGGGCTGCGAACCCGCGAGGGGGAGCCAATCCCATAAAACCGTTCCCAGTTCGGATTGCAGGCTGCAACTCGCCTGCATGAAGCCGGAATCGCTAG
TAATCGTGGATCAGCATGCCACGGTGAATACGTTCCCGGGTCTTGTACACACCGCCCGTCACACCACGAGAGTTTGTAACACCCGAAGTCGGTGGGGTAA
CCCTTACGGGAGCCAGCCGCCGAAG

>EB279
ACCGTCCTCCCGAAGGTTAGACTAGCTACTTCTGGTGCAACCCACTCCCATGGTGTGACGGGCGGTGTGTACAAGGCCCGGGAACGTATTCACCGCGACA
TTCTGATTCGCGATTACTAGCGATTCCGACTTCACGCAGTCGAGTTGCAGACTGCGATCCGGACTACGATCGGTTTTGTGAGATTAGCTCCACCTCGCGG
CTTGGCGACCCTCTGTACCGACCATTGTAGCACGTGTGTAGCCCAGGCCGTAAGGGCCATGATGACTTGACGTCATCCCCACCTTCCTCCGGTTTGTCAC
CGGCAGTCTCCTTAGAGTGCCCACCATAACGTGCTGGTAACTAAGGACAAGGGTTGCGCTCGTTACGGGACTTAACCAACATCTCACGACACGAGCTGAC
GACAGCCATGCAGCACCTGTCTCAATGTTCCCGAAGGCACCAATCCATCTCTGGAAAGTTCATTGGATGTCAAGGCCTGGTAAGGTTCTTCGCGTTGCTT
CGAATTAAACCACATGCTCCACCGCTTGTGCGGGCCCCCGTCAATTCATTTGAGTTTTAACCTTGCGGCCGTACTCCCCAGGCGGTCAACTTAATGCGTT
AGCTGCGCCACTAAAATCTCAAGGATTCCAACGGCTAGTTGACATCGTTTACGGCGTGGACTACCAGGGTATCTAATCCTGTTTGCTCCCCACGCTTTCG
CACCTCAGTGTCAGTATGAGCCCAGGTGGTCGCCTTCGCCACTGGTGTTCCTTCCTATATCTACGCATTTCACCGCTACACAGGAAATTCCACCACCCTC
TGCCCTACTCTAGCTCGCCAGTTTTGGATGCAGTTCCCAGGTTGAGCCCGGGGATTTCACATCCAACTTAACGAACCACCTACGCGCGCTTTACGCCCAG
TAATTCCGATTAACGCTTGCACCCTCTGTATTACCGCGGCTGCTGGCACAGAGTTAGCCGGTGCTTATTCTGTCGGTAACGTCAAAACAGCAAGGTATTC
GCTTACTGCCCTTCCTCCCAACTTAAAGTGCTTTACAATCCGAAGACCTTCTTCACACACGCGGCATGGCTGGATCAGGCTTTCGCCCATTGTCCAATAT
TCCCCACTGCTGCCTCCCGTAGGAGTCTGGACCGTGTCTCAGTTCCAGTGTGACTGATCATCCTCTCAGACCAGTTACGGATCGTAGCCTTGGTGAGCCA
TTACCTCACCAACTAGCTAATCCGACCTAGGCTCATCTGATAGCGCAAGGCCCGAAGGTCCCCTGCTTTCTCCCGTAGGACGTATGCGGTATTAGCGTTC
CTTTCGAAACGTTGTCCCCCACTACCAGGCAGATTCCTAGGTATTACTCACCCGTCCGCCGCTGAATCGAAGAGCAAGCTCTTCTCATCCGCTCGACTGC
A

>EB280
CCGTCCTCCCGAAGGTTAGACTAGCTACTTCTGGTGCAACCCACTCCCATGGTGTGACGGGCGGTGTGTACAAGGCCCGGGAACGTATTCACCGCGACAT
TCTGATTCGCGATTACTAGCGATTCCGACTTCACGCAGTCGAGTTGCAGACTGCGATCCGGACTACGATCGGTTTTATGGGATTAGCTCCACCTCGCGGC
TTGGCAACCCTTTGTACCGACCATTGTAGCACGTGTGTAGCCCAGGCCGTAAGGGCCATGATGACTTGACGTCATCCCCACCTTCCTCCGGTTTGTCACC
GGCAGTCTCCTTAGAGTGCCCACCATAACGTGCTGGTAACTAAGGACAAGGGTTGCGCTCGTTACGGGACTTAACCCAACATCTCACGACACGAGCTGAC
GACAGCCATGCAGCACCTGTCTCAATGTTCCCGAAGGCACCAATCCATCTCTGGAAAGTTCATTGGATGTCAAGGCCTGGTAAGGTTCTTCGCGTTGCTT
CGAATTAAACCACATGCTCCACCGCTTGTGCGGGCCCCCGTCAATTCATTTGAGTTTTAACCTTGCGGCCGTACTCCCCAGGCGGTCAACTTAATGCGTT
AGCTGCGCCACTAAGAGCTCAAGGCTCCCAACGGCTAGTTGACATCGTTTACGGCGTGGACTACCAGGGTATCTAATCCTGTTTGCTCCCCACGCTTTCG
CACCTCAGTGTCAGTATCAGTCCAGGTGGTCGCCTTCGCCACTGGTGTTCCTTCCTATATCTACGCATTTCACCGCTACACAGGAAATTCCACCACCCTC
TACCATACTCTAGCTCGCCAGTTTTGGATGCAGTTCCCAGGTTGAGCCCGGGGGATTTCACATCCAACTTAACGAACCACCTACGCGCGCTTTACGCCCA
GTAATTCCGATTAACGCTTGCACCCTCTGTATTACCGCGGCTGCTGGCACAGAGTTAGCCGGTGCTTATTCTGTCGGTAACGTCAAAATTGCAGAGTATT
AATCTACAACCCTTCCTCCCAACTTAAAGTGCTTTACAATCCGAAGACCTTCTTCACACACGCGGCATGGCTGGATCAGGCTTTCGCCCATTGTCCAATA
TTCCCCACTGCTGCCTCCCGTAGGAGTCTGGACCGTGTCTCAGTTCCAGTGTGACTGATCATCCTCTCAGACCAGTTACGGATCGTCGCCTTGGTGAGCC
ATTACCTCACCAACTAGCTAATCCGACCTAGGCTCATCTGATAGCGCAAGGCCCGAAGGTCCCCTGCTTTCTCCCGTAGGACGTATGCGGTATTAGCGTT
CCTTTCGAAACGTTGTCCCCCACTACCAGGCAGATTCCTAGGCATTACTCACCCGTCCGCCGCTGAATCCAGGAGCAAGCTCCCTTCATCCGCTCGACTT
GCA

>EB281
CGAGCGGTAGAGAGAAGCTTGCTTCTCTTGAGAGCGGCGGACGGGTGAGTAATGCCTAGGAATCTGCCTGGTAGTGGGGGATAACGTTCGGAAACGGACG
CTAATACCGCATACGTCCTACGGGAGAAAGCAGGGGACCTTCGGGCCTTGCGCTATCAGATGAGCCTAGGTCGGATTAGCTAGTTGGTGGGGTAATGGCT
CACCAAGGCGACGATCCGTAACTGGTCTGAGAGGATGATCAGTCACACTGGAACTGAGACACGGTCCAGACTCCTACGGGAGGCAGCAGTGGGGAATATT
GGACAATGGGCGAAAGCCTGATCCAGCCATGCCGCGTGTGTGAAGAAGGTCTTCGGATTGTAAAGCACTTTAAGTTGGGAGGAAGGGCAGTTGCCTAATA
CGTAACTGTTTTGACGTTACCGACAGAATAAGCACCGGCTAACTCTGTGCCAGCAGCCGCGGTAATACAGAGGGTGCAAGCGTTAATCGGAATTACTGGG
CGTAAAGCGCGCGTAGGTGGTTTGTTAAGTTGGATGTGAAATCCCCGGGCTCAACCTGGGAACTGCATTCAAAACTGACTGACTAGAGTATGGTAGAGGG

TGGTGGAATTTCCTGTGTAGCGGTGAAATGCGTAGATATAGGAAGGAACACCAGTGGCGAAGGCGACCACCTGGACTGATACTGACACTGAGGTGCGAAA
GCGTGGGGAGCAAACAGGATTAGATACCCTGGTAGTCCACGCCGTAAACGATGTCAACTAGCCGTTGGAAGCCTTGAGCTTTTAGTGGCGCAGCTAACGC
ATTAAGTTGACCGCCTGGGGAGTACGGCCGCAAGGTTAAAACTCAAATGAATTGACGGGGGCCCGCACAAGCGGTGGAGCATGTGGTTTAATTCGAAGC
AACGCGAAGAACCTTACCAGGCCTTGACATCCAATGAACTTTCCAGAGATGGATTGGTGCCTTCGGGAGCATTGAGACAGGTGCTGCATGGCTGTCGTCA
GCTCGTGTCGTGAGATGTTGGGTTAAGTCCCGTAACGAGCGCAACCCTTGTCCTTAGTTACCAGCACATAATGGTGGGCACTCTAAGGAGACTGCCGGTG
ACAAACCGGAGGAAGGTGGGGATGACGTCAAGTCATCATGGCCCTTACGGCCTGGGCTACACACGTGCTACAATGGTCGGTACAGAGGGTTGCCAAGCCG
CGAGGTGGAGCTAATCCCATAAAACCGATCGTAGTCCGGATCGCAGTCTGCAACTCGACTGCGTGAAGTCGGAATCGCTAGTAATCGCGAATCAGAATGT
CGCGGTGAATACGTTCCCGGGCCTTGTACACACCGCCCGTCACACCATGGGAGTGGGTTGCACCAGAAGTAGCTAGTCTAACCTTCGGGAGGACGGT

>EB282
GTCGAGCGGATGAAGGGGGCTTGCTCCCTGATTTAGCGGCGGACGGGTGAGTAATGCCTAGGAATCTGCCTGGTAGTGGGGGATAACGTTCCGAAAGGAA
CGCTAATACCGCATACGTCCTACGGGAGAAAGCAGGGGACCTTCGGGCCTTGCGCTATCAGATGAGCCTAGGTCGGATTAGCTAGTTGGTGAGGTAATGG
CTCACCAAGGCGACGATCCGTAACTGGTCTGAGAGGATGATCAGTCACACTGGAACTGAGACACGGTCCAGACTCCTACGGGAGGCAGCAGTGGGGAATA
TTGGACAATGGGCGAAAGCCTGATCCAGCCATGCCGCGTGTGTGAAGAAGGTCTTCGGATTGTAAAGCACTTTAAGTTGGGAGGAAGGGCATTAACCTAA
TACGTTAGTGTTTTGACGTTACCAACAGAATAAGCACCGGCTAACTTCGTGCCAGCAGCCGCGGTAATACGAAGGGTGCAAGCGTTAATCGGAATTACTG
GGCGTAAAGCGCGCGTAGGTGGTTCGTTAAGTTGGATGTGAAAGCCCCGGGCTCAACCTGGGAACTGCATCCAAAACTGGCGAGCTAGAGTACGGTAGAG
GGTGGTGGAATTTCCTGTGTAGCGGTGAAATGCGTAGATATAGGAAGGAACACCAGTGGCGAAGGCGACCACCTGGACTGATACTGACACTGAGGTGCGA
AAGCGTGGGGAGCAAACAGGATTAGATACCCTGGTAGTCCACGCCGTAAACGATGTCAACTAGCCGTTGGAATCCTTGAGATTTTAGTGGCGCAGCTAAC
GCATTAAGTTGACCGCCTGGGGGAGTACGGCCGCAAGGTTAAAACTCAAATGAATTGACGGGGGCCCGCACAAGCGGTGGAGCATGTGGTTTAATTCGAA
GCAACGCGAAGAACCTTACCTGGCCTTGACATGCTGAGAACTTTCCAGAGATGGATTGGTGCCTTCGGGAACTCAGACACAGGTGCTGCATGGCTGTCGT
CAGCTCGTGTCGTGAGATGTTGGGTTAAGTCCCGTAACGAGCGCAANCCCTTGTCCTTAGTTACCAGCACCTCGGGTGGGCACTCTAAGGAGACTGCCGG
TGACAAACCGGAGGAAGGTGGGGATGACGTCAAGTCATCATGGCCCTTACGGCCAGGGCTACACACGTGCTACAATGGTCGGTACAAAGGGTTGCCAAGC
CGCGAGGTGGAGCTAATCCCATAAAACCGATCGTAGTCCGGATCGCAGTCTGCAACTCGACTGCGTGAAGTCGGAATCGCTAGTAATCGTGAATCAGAAT
GTCACGGTGAATACGTTCCCGGGCCTTGTACACACCGCCCGTCACACCATGGGAGTGGGTTGCTCCAGAAGTAGCTAGTCTAACCTTCGGGGGGAC

>EB283
CTTGCTCTGTTCAAGTTAGCGGCGGACGGGTGAGTAACACGTGGGTAACCTGCCTGTAAGACTGGGATAACTCCGGGAAACCGGGGCTAATACCGGATAT
TCTTTCTTCTCCGCATGGAGAAGGATGGAAAGGCGGCTTCGGCTGTCACTTACAGATGGACCCGCGGCGCATTAGCTAGTTGGTGGGGTAACGGCCCACC
AAGGCGACGATGCGTAGCCGACCTGAGAGGGTGATCGGCCACACTGGGACTGAGACACGGCCCAGACTCCTACGGGAGGCAGCAGTAGGGAATCTTCCGC
AATGGACGAAAGTCTGACGGAGCAACGCCGCGTGAGTGAAGAAGGTTTTCGGATCGTAAAGCTCTGTTGTTAGGGAAGAACAAGTACGGGAGTAACTGTC
CGTACCTTGACGGTACCTAACCAGAAAGCCACGGCTAACTACGTGCCAGCAGCCGCGGTAATACGTAGGTGGCAAGCGTTGTCCGGAATTATTGGGCGTA
AAGCGCGCGCAGGCGGCTTCTTAAGTCTGATGTGAAAGCCCACGGCTCAACCGTGGAGGGTCATTGGAAACTGGGAGGCTTGAGTGCAGAAGAGGAGAGC
GGAATTCCACGTGTAGCGGTGAAATGCGTAGAGATGTGGAGGAACACCAGTGGCGAAGGCGGCTCTCTGGTCTGTAACTGACGCTGAGGCGCGAAAGCGT
GGGGAGCGAACAGGATTAGATACCCTGGTAGTCCACGCCGTAAACGATGAGTGCTAAGTGTTGGAGGGTTTCCGCCCTTCAGTGCTGCAGCTAACGCATT
AAGCACTCCGCCTGGGGAGTACGGTCGCAAGACTGAAACTCAAAGGAATTGACGGGGGCCCGCACAAGCGGTGGAGCATGTGGTTTAATTCGAAGCAACG
CGAAGAACCTTACCAGGTCTTGACATCCCGCTGACCGGTCTGGAGACAGATCTTTCCCTTCGGGGACAGCGGTGACAGGTGGTGCATGGTTGTCGTCAGC
TCGTGTCGTGAGATGTTGGGTTAAGTCCCGCAACGAGCGCAACCCTTGATCTTAGTTGCCAGCATTCAGTTGGGCACTCTAAGGTGACTGCCGGTGACAA
ACCGGAGGAAGGTGGGGATGACGTCAAATCATCATGCCCCTTATGACCTGGGCTACACACGTGCTACAATGGATGGTACAAAGGGCTGCAAGACCGCAAG
GTTTAGCCAATCCCATAAAACCATTCTCAGTTCGGATTGCAGGCTGCAACTCGCCTGCATGAAGCCGGAATCGCTAGTAATCGCGGATCAGCATGCCGCG
GTGAATACGTTCCCGGGCCTTGTACACACCGCCCGTCACACCACGAGAGTTTGCAACACCTCGAAGTCGGTGGGGTAACCCTTACGGGAGCCAGCCGCCT

>EB284
TATGGCTCAGATTGAACGCTGGCGGCAGGCCTAACACATGCAAGTCGAGCGGATGAAAGGAGCTTGCTCCTGGATTCAGCGGCGGACGGGTGAGTAATGC
CTAGGAATCTGCCTGGTAGTGGGGGACAACGTTTCGAAAGGAACGCTAATACCGCATACGTCCTACGGGAGAAAGCAGGGGACCTTCGGGCCTTGCGCTA
TCAGATGAGCCTAGGTCGGATTAGCTAGTTGGTGAGGTAATGGCTCACCAAGGCGACGATCCGTAACTGGTCTGAGAGGATGATCAGTCACACTGGAACT
GAGACACGGTCCAGACTCCTACGGGAGGCAGCAGTGGGGAATATTGGACAATGGGCGAAAGCCTGATCCAGCCATGCCGCGTGTGTGAAGAAGGTCTTCG
GATTGTAAAGCACTTTAAGTTGGGAGGAAGGGTTGTAGATTAATACTCTGCAATTTTGACGTTACCGACAGAATAAGCACCGGCTAACTCTGTGCCAGCA
GCCGCGGTAATACAGAGGGTGCAAGCGTTAATCGGAATTACTGGGCGTAAAGCGCGCGTAGGTGGTTCGTTAAGTTGGATGTGAAATCCCCGGGCTCAAC
CTGGGAACTGCATCCAAAACTGGCGAGCTAGAGTATGGTAGAGGGTGGTGGAATTTCCTGTGTAGCGGTGAAATGCGTAGATATAGGAAGGAACACCAGT
GGCGAAGGCGACCACCTGGACTGATACTGACACTGAGGTGCGAAAGCGTGGGGAGCAAACAGGATTAGATACCCTGGTAGTCCACGCCGTAAACGATGTC
AACTAGCCGTTGGGAGCCTTGAGCTCTTAGTGGCGCAGCTAACGCATTAAGTTGACCGCCTGGGGAGTACGGCCGCAAGGTTAAAACTCAAATGAATTGA
CGGGGGCCCGCACAAGCGGTGGAGCATGTGGTTTAATTCGAAGCAACGCGAAGAACCTTACCAGGCCTTGACATCCAATGAACTTTCCAGAGATGGATTG
GTGCCTTCGGGAACATTGAGACAGGTGCTGCATGGCTGTCGTCAGCTCGTGTCGTGAGATGTTGGGTTAAGTCCCGTAACGAGCGCAACCCTTGTCCTTA
GTTACCAGCACGTTATGGTGGGCACTCTAAGGAGACTGCCGGTGACAAACCGGAGGAAGGTGGGGATGACGTCAAGTCATCATGGCCCTTACGGCCTGGG
CTACACACGTGCTACAATGGTCGGTACAAAGGGTTGCCAAGCCGCGAGGTGGAGCTAATCCCATAAAACCGATCGTAGTCCGGATCGCAGTCTGCAACTC
GACTGCGTGAAGTCGGAATCGCTAGTAATCGCGAATCAGAATGTCGCGGTGAATACGTTCCCGGGCCTTGTACACACCGCCCGTCACACCATGGGAGTGG
GTTGCACCAGAAGTAGCTAGTCTAACCTTCGGGAGGACGGTACCACG

>EB285
CGAGCGGCAGCACGGGTACTTGTACCTGGTGGCGAGCGGCGGACGGGTGAGTAATGCCTAGGAATCTGCCTAGTAGTGGGGGATAACGTCCGGAAACGGG
CGCTAATACCGCATACGTCCTACGGGAGAAAGTGGGGGATCTTCGGACCTCACGCTATTAGATGAGCCTAGGTCGGATTAGCTAGTTGGTGAGGTAATGG
CTCACCAAGGCGACGATCCGTAACTGGTCTGAGAGGATGATCAGTCACACTGGAACTGAGACACGGTCCAGACTCCTACGGGAGGCAGCAGTGGGGAATA
TTGGACAATGGGCGAAAGCCTGATCCAGCCATGCCGCGTGTGTGAAGAAGGTCTTCGGATTGTAAAGCACTTTAAGTTGGGAGGAAGGGCAGTTACCTAA
TACGTGATTGTTTTGACGTTACCGACAGAATAAGCACCGGCTAACTCTGTGCCAGCAGCCGCGGTAATACAGAGGGTGCAAGCGTTAATCGGAATTACTG
GGCGTAAAGCGCGCGTAGGTGGTTTGTTAAGTTGGATGTGAAAGCCCCGGGCTCAACCTGGGAACTGCATCCAAAACTGGCAAGCTAGAGTATGGTAGAG
GGTGGTGGAATTTCCTGTGTAGCGGTGAAATGCGTAGATATAGGAAGGAACACCAGTGGCGAAGGCGACCACCTGGACTGATACTGACACTGAGGTGCGA
AAGCGTGGGGAGCAAACAGGATTAGATACCCTGGTAGTCCACGCCGTAAACGATGTCAACTAGCCGTTGGGAGCCTTGAGCTCTTAGTGGCGCAGCTAAC
GCATTAAGTTGACCGCCTGGGGAGTACGGCCGCAAGGTTAAAACTCAAATGAATTGACGGGGGCCCGCACAAGCGGTGGAGCATGTGGTTTAATTCGAAG
CAACGCGAAGAACCTTACCAGGCCTTGACATCCAATGAACTTTCTAGAGATAGATTGGTGCCTTCGGGAACATTGAGACAGGTGCTGCATGGCTGTCGTC
AGCTCGTGTCGTGAGATGTTGGGTTAAGTCCCGTAACGAGCGCAACCCTTGTCCTTAGTTACCAGCACGTTATGGTGGGCACTCTAAGGAGACTGCCGGT
GACAAACCGGAGGAAGGTGGGGATGACGTCAAGTCATCATGGCCCTTACGGCCTGGGCTACACACGTGCTACAATGGTCGGTACAAAGGGTTGCCAAGCC
GCGAGGTGGAGCTAATCCCATAAAACCGATCGTAGTCCGGATCGCAGTCTGCAACTCGACTGCGTGAAGTCGGAATCGCTAGTAATCGCGAATCAGAATG
TCGCGGTGAATACGTTCCCGGGCCTTGTACACACCGCCCGTCACACCATGGGAGTGGGTTGCACCAGAAGTAGCTAGTCTAACCTTCGGGAGGACGGTA

>EB286
GTCCTCCCGAAGGTTAGACTAGCTACTTCTGGTGCAACCCACTCCCATGGTGTGACGGGCGGTGTGTACAAGGCCCGGGAACGTATTCACCGCGACATTC
TGATTCGCGATTACTAGCGATTCCGACTTCACGCAGTCGAGTTGCAGACTGCGATCCGGACTACGATCGGTTTTGTGAGATTAGCTCCACCTCGCGGCTT
GGCAACCCTCTGTACCGACCATTGTAGCACGTGTGTAGCCCAGGCCGTAAGGGCCATGATGACTTGACGTCATCCCCACCTTCCTCCGGTTTGTCACCGG

CAGTCTCCTTAGAGTGCCCACCATAACGTGCTGGTAACTAAGGACAAGGGTTGCGCTCGTTACGGGACTTAACCCAACATCTCACGACACGAGCTGACGA
CAGCCATGCAGCACCTGTGTCAGAGTTCCCGAAGGCACCCAATCTATCTCTGGAAAGTTCTCTGCATGTCAAGGCCTGGTAAGGTTCTTCGCGTTGCTTC
GAATTAAACCACATGCTCCACCGCTTGTGCGGGCCCCCGTCAATTCATTTGAGTTTTAACCTTGCGGCCGTACTCCCCAGGCGGTCAACTTAATGCGTTA
GCTGCGCCACTAAAATCTCAAGGATTCCAACGGCTAGTTGACATCGTTTACGGCGTGGACTACCAGGGTATCTAATCCTGTTTGCTCCCCACGCTTTCGC
ACCTCAGTGTCAGTATCAGTCCAGGTGGTCGCCTTCGCCACTGGTGTTCCTTCCTATATCTACGCATTTCACCGCTACACAGGAAATTCCACCACCCTCT
ACCGTACTCTAGCTTGCCAGTTTTGGATGCAGTTCCCAGGTTGAGCCCGGGGCTTTCACATTCAACTTAACAAACCACCTACGCGCGCTTTACGCCCAGT
AATTCCGATTAACGCTTGCACCCTCTGTATTACCGCGGCTGCTGGCACAGAGTTAGCCGGTGCTTATTCTGTCGGTAACGTCAAAACAGCAAGGTATTCG
CTTACTGCCCTTCCTCCCAACTTAAAGTGCTTTACAATCCGAAGACCTTCTTCACACACGCGGCATGGCTGGATCAGGCTTTCGCCCATTGTCCAATATT
CCCCACTGCTGCCTCCCGTAGGAGTCTGGACCGTGTCTCAGTTCCAGTGTGACTGATCATCCTCTCAGACCAGTTACGGATCGTCGCCTTGGTGAGCCAT
TACCCCACCAACTAGCTAATCCGACCTAGGCTCATCTGATAGCGCAAGGCCCGAAGGTCCCCTGCTTTCTCCCGTAGGACGTATGCGGTATTAGCGTTCC
TTTCGAAACGTTGTCCCCCACTACCAGGCAGATTCCTAGGCATTACTCACCCGTCCGCCGCTGAATCGAAGAGCAAGCTCTTCTCATCCGCTCGACTGCA

>EB287
AGTTTGATTATGGCTCAGTAAGTCGTAACAAGGTAACCGTAAAGTTTGATTATGGCTCATTAAGTCGTAACAAGGTAACCGTATAGTTTGATGATGGCTC
ACCAGGACATAGGGGCGAATACGGAAAGGTTGATTGAACCGCAGGTTCCGACATAAGAGGTGGCTTCGGCTACCACTTACAGATGGACCCGCGGCGCATA
GCTAGTTGGTGAGGTAACGGCTCACCAAGGCGACGATGCGTAGCCGACCTGAGAGGGTGATCGGCCACACTGGGACTGAGACACGGCCCAGACTCCTACG
GGAGGCAGCAGTAGGGAATCTTCCGCAATGGACGAAAGTCTGACGGAGCAACGCCGCGTGAGTGATGAAGGTTTTCGGATCGTAAAGCTCTGTTGTTAGG
GAAGAACAAGTGCCGTTCAANTAGGGCGGCACCTTGACGGTACCTAACCAGAANGCCACGGCTAACTACGTGCCAGCAGCCGCGGTAATACGTAGGTGGC
AAGCGTTGTCCGGAATTATTGGNCGTAAAGGGCTCGCAGGCGGTTTCTTAAGTCTGATGTGAAAGCCCCCGGCTCAACCGGGGAGGGTCATTGGAAACTG
GGGAACTTGAGTGCAGAAGAGGAGAGTGGAATTCCACGTGTAGCGGTGAAATGCGTAGAGATGTGGAGGAACACCAGTGGCGAAGGCGACTCTCTGGTCT
GTAACTGACGCTGAGGAGCGAAAGCGTGGGGAGCGAACAGGATTAGATACCCTGGTAGTCCACGCCGTAAACGATGAGTGCTAAGTGTTAGGGGGTTTCC
GCCCCTTAGTGCTGCAGCTAACGCATTAAGCACTCCGCCTGGGGAGTACGGTCGCAAGACTGAAACTCAAAGGAATTGACGGGGGCCCGCACAAGCGGTG
GAGCATGTGGTTTAATTCGAAGCAACGCGAAGAACCTTACCAGGTCTTGACATCCTCTGACAATCCTAGAGATAGGACGTCCCCTTCGGGGNGCAGAGTG
ACAGGTGGTGCATGGTTGTCGTCAGCTCGTGTCGTGAGATGTTGGGTTAAGTCCCGCAACGAGCGCAACCCTTGATCTTAGTTGCCAGCATTCAGTTGGG
CACTCTAAGGTGACTGCCGGTGACAAACCGGAGGAAGGTGGGGATGACGTCAAATCATCATGCCCCTTATGACCTGGGCTACACACGTGCTACAATGGAC
AGAACAAAGGGCAGCGAAACCGCGAGGTTAAGCCAATCCCACAAATCTGTTCTCAGTTCGGATCGCAGTCTGCAACTCGAATGCGTGAAGCAGGAATCGG
TAGTAATCGCGGATCAGCCTGTCGCGGGGAACACGTTCCCGGTCGTGGTTCACCCCGGCTCTC

>EB288
CCTACGGGAGAAAGCAGGGGACCTTCGGGCCTTGCGCTAATAGATGAGCCTAAGTCGGATTAGCTAGTTGGTGGGGTAAAGGCCTACCAAGGCGACTATC
TGTAGCGGGTCTGAGAGGATGATCCGCCACACTGGGACTGAGACACGGCCCACACTCCTACGGGAGGCAGCAGTGGGGAATATTGGACGATGGGGGGAAC
CCTGATCCAGCCATGCCGCGTGTGTGAAGAAGGCCTTTTGGTTGTAAAGCACTTTANGCGAGGAGGAGGCTACCGAGATTANTACTCTTGGATAGTGGAC
GTTACTCGCAGAATAAGCACCGGCTAACTCTGTGCCAGCAGCCGCGGTAATACAGAGGGTGCAAGCGTTAATCGGATTTACTGGGCGTAAAGCGCGCGTA
GGTGGCCAATTAAGTCAAATGTGAAATCCCCGAGCTTAACTTGGGAATTGCATTCGATACTGGTTGGCTAGAGTATGGGAGAGGATGGTAGAATTCCAGG
TGTAGCGGTGAAATGCGTAGAGATCTGGAGGAATACCGATGGCGAAGGCAGCCATCTGGCCTAATACTGACACTGAGGTGCGAAAGCATGGGGAGCAAAC
AGGATTAGATACCCTGGTAGTCCATGCCGTAAACGATGTCTACTAGCCGTTGGGGCCTTTGAGGCTTTAGTGGCGCAGCTAACGCGATAAGTAGACCGCC
TGGGGAGTACGGTCGCAAGACTAAAACTCAAATGAATTGACGGGGGCCCGCACAAGCGGTGGAGCATGTGGTTTAATTCGATGCAACGCGAAGAACCTTA
CCTGGTCTTGACATAGTAAGAACTTTCCAGAGATGGATTGGTGCCTTCGGGAACTTACATACAGGTGCTGCATGGCTGTCGTCAGCTCGTGTCGTGAGAT
GTTGGGTTAAGTCCCGCAACGAGCGCAACCCTTTTCCTTATTTGCCAGCGGGTTAAGCCGGGAACTTTAAGGATACTGCCAGTGACAAACTGGAGGAAGG
CGGGGACGACGTCAAGTCATCATGGCCCTTACGACCAGGGCTACACACGTGCTACAATGGTCGGTACAAAGGGTTGCTACCTCGCGAGAGGATGCTAATC
TCAAAAAGCCGATCGTAGTCCGGATTGGAGTCTGCAACTCGACTCCATGAAGTGGGAATCGCTGGTAATCGCGGATCAGAATGCCGCGGTGAATACGTTC
CCGGGCCTTGTACACACCGCCCGTCACACCATGGGAGTTTGCGCACCAGAAGTAGGTGGT

>EB289
TGCAGTCGAGCGGATGAGGAGAGGCTTGCTCTCTGATTCAGCGGCGGACGGGTGAGTAATGCCTAGGAATCTGCCTGATAGTGGGGGACAACGTTTCGAA
AGGAACGCTAATACCGCATACGTCCTACGGGAGAAAGCAGGGGACCTTCGGGCCTTGCGCTATCAGATGAGCCTAGGTCGGATTAGCTAGTTGGTGAGGT
AACGGCTCACCAAGGCGACGATCCGTAACTGGTCTGAGAGGATGATCAGTCACACTGGAACTGAGACACGGTCCAGACTCCTACGGGAGGCAGCAGTGGG
GAATATTGGACAATGGGCGAAAGCCTGATCCAGCCATGCCGCGTGTGTGAAGAAGGTCTTCGGATTGTAAAGCACTTTAAGTTGGGAGGAAGGGCATTAA
CCTAATACGTTAGTGTTTTGACGTTACCGACAGAATAAGCACCGGCTAACTTCGTGCCAGCAGCCGCGGTAATACGAAGGGTGCAAGCGTTAATCGGAAT
TACTGGGCGTAAAGCGCGCGTAGGTGGTTTGTTAAGTTGAATGTGAAAGCCCCGGGCTCAACCTGGGAACTGCATCCAAAACTGGCAAGCTAGAGTATGG
CAGAGGGTGGTGGAATTTCCTGTGTAGCGGTGAAATGCGTAGATATAGGAAGGAACACCAGTGGCGAAGGCGACCACCTGGGCTAATACTGACACTGAGG
TGCGAAAGCGTGGGGAGCAAACAGGATTAGATACCCTGGTAGTCCACGCCGTAAACGATGTCGACTAGCCGTTGGGATCCTTGAGATCTTAGTGGCGCAG
CTAACGCATTAAGTCGACCGCCTGGGGAGTACGGCCGCAAGGTTAAAACTCAAATGAATTGACGGGGGCCCGCACAAGCGGTGGAGCATGTGGTTTAATT
CGAAGCAACGCGAAGAACCTTACCAGGCCTTGACATGCAGAGAACTTTCCAGAGATGGATTGGTGCCTTCGGGAACTCTGACACAGGTGCTGCATGGCTG
TCGTCAGCTCGTGTCGTGAGATGTTGGGTTAAGTCCCGTAACGAGCGCAACCCTTGTCCTTAGTTACCAGCACGTTAAGGTGGGCACTCTAAGGAGACTG
CCGGTGACAAACCGGAGGAAGGTGGGGATGACGTCAAGTCATCATGGCCCTTACGGCCTGGGCTACACACGTGCTACAATGGTCGGTACAAAGGGTTGCC
AAGCCGCGAGGTGGAGCTAATCCCATAAAACCGATCGTAGTCCGGATCGCAGTCTGCAACTCGACTGCGTGAAGTCGGAATCGCTAGTAATCGTGAATCA
GAATGTCACGGTGAATACGTTCCCGGGCCTTGTACACACCGCCCGTCACACCATGGGAGTGGGTTGCTCCAGAAGTAGCTAGTCTAACCTTCGGGGGGAC
GGTACCAC

>EB290
CGAGCGGATGACGGGAGCTTGCTCCTGGATTCAGCGGCGGACGGGTGAGTAATGCCTAGGAATCTGCCTGGTAGTGGGGGACAACGTTTCGAAAGGAACG
CTAATACCGCATACGTCCTACGGGAGAAAGCAGGGGACCTTCGGGCCTTGCGCTATCAGATGAGCCTAGGTCGGATTAGCTTGTTGGTGAGGTAATGGCT
CACCAAGGCGACGATCCGTAACTGGTCTGAGAGGATGATCAGTCACACTGGAACTGAGACACGGTCCAGACTCCTACGGGAGGCAGCAGTGGGGAATATT
GGACAATGGGCGAAAGCCTGATCCAGCCATGCCGCGTGTGTGAAGAAGGTCTTCGGATTGTAAAGCACTTTAAGTTGGGAGGAAGGGTTGTAGATTAATA
CTCTGCAATTTTGACGTTACCGACAGAATAAGCACCGGCTAACTCTGTGCCAGCAGCCGCGGTAATACAGAGGGTGCAAGCGTTAATCGGAATTACTGGG
CGTAAAGCGCGCGTAGGTGGTTTGTTAAGTTGGATGTGAAAGCCCCGGGCTCAACCTGGGAACTGCATCCAAAACTGGCAAGCTAGAGTACGGTAGAGGG
TGGTGGAATTTCCTGTGTAGCGGTGAAATGCGTAGATATAGGAAGGAACACCAGTGGCGAAGGCGACCACCTGGACTGATACTGACACTGAGGTGCGAAA
GCGTGGGGAGCAAACAGGATTAGATACCCTGGTAGTCCACGCCGTAAACGATGTCAACTAGCCGTTGGAATCCTTGAGATTTTAGTGGCGCAGCTAACGC
ATTAAGTTGACCGCCTGGGGAGTACGGCCGCAAGGTTAAAACTCAAATGAATTGACGGGGGCCCGCACAAGCGGTGGAGCATGTGGTTTAATTCGAAGCA
ACGCGAAGAACCTTACCAGGCCTTGACATGCAGAGAACTTTCCAGAGATGGATTGGTGCCTTCGGGAACTCTGACACAGGTGCTGCATGGCTGTCGTCAG
CTCGTGTCGTGAGATGTTGGGTTAAGTCCCGTAACGAGCGCAACCCTTGTCCTTAGTTACCAGCACGTTATGGTGGGCACTCTAAGGAGACTGCCGGTGA
CAAACCGGAGGAAGGTGGGGATGACGTCAAGTCATCATGGCCCTTACGGCCTGGGCTACACACGTGCTACAATGGTCGGTACAGAGGGTTGCCAAGCCGC
GAGGTGGAGCTAATCTCACAAAACCGATCGTAGTCCGGATCGCAGTCTGCAACTCGACTGCGTGAAGTCGGAATCGCTAGTAATCGCGAATCAGAATGTC
GCGGTGAATACGTTCCCGGGCCTTGTACACACCGCCCGTCACACCATGGGAGTGGGTTGCACACAAGAAGTAGCTAGTCTAACCTTCGGG

>EB291

TGCAGTCGAGCGGATGAAGGGAGCTTGCTCCTGGATTCAGCGGCGGACGGGTGAGTAATGCCTAGGAATCTGCCTGGTAGTGGGGGACAACGTTTCGAAA
GGAACGCTAATACCGCATACGTCCTACGGGAGAAAGCAGGGGACCTTCGGGCCTTGCGCTATCAGATGAGCCTAGGTCGGATTAGCTAGTTGGTGAGGTA
ATGGCTCACCAAGGCGACGATCCGTAACTGGTCTGAGAGGATGATCAGTCACACTGGAACTGAGACACGGTCCAGACTCCTACGGGAGGCAGCAGTGGGG
AATATTGGACAATGGGCGAAAGCCTGATCCAGCCATGCCGCGTGTGTGAAGAAGGTCTTCGGATTGTAAAGCACTTTAAGTTGGGAGGAAGGGTTGTAGA
TTAATACTTTGCTGTTTTGACGTTACCGACAGAATAAGCACCGGCTAACTCTGTGCCAGCAGCCGCGGTAATACAGAGGGTGCAAGCGTTAATCGGAATT
ACTGGGCGTAAAGCGCGCGTAGGTGGTTCGTTAAGTTGGATGTGAAATCCCCGGGCTCAACCTGGGAACTGCATCCAAAACTGGCGAGCTAGAGTATGGT
AGAGGGTGGTGGAATTTCCTGTGTAGCGGTGAAATGCGTAGATATAGGAAGGAACACCAGTGGCGAAGGCGACCACCTGGACTGATACTGACACTGAGGT
GCGAAAGCGTGGGGAGCAAACAGGATTAGATACCCTGGTAGTCCACGCCGTAAACGATGTCAACTAGCCGTTGGGAGCCTTGAGCTCTTAGTGGCGCAGC
TAACGCATTAAGTTGACCGCCTGGGGAGTACGGCCGCAAGGTTAAAACTCAAATGAATTGACGGGGGCCCGCACAAGCGGTGGAGCATGTGGTTTAATTC
GAAGCAACGCGAAGAACCTTACCAGGCCTTGACATCCAATGAACTTTCCAGAGATGGATTGGTGCCTTCGGGAACATTGAGACAGGTGCTGCATGGCTGT
CGTCAGCTCGTGTCGTGAGATGTTGGGTTAAGTCCCGTAACGAGCGCAACCCTTGTCCTTAGTTACCAGCACGTAATGGTGGGCACTCTAAGGAGACTGC
CGGTGACAAACCGGAGGAAGGTGGGGATGACGTCAAGTCATCATGGCCCTTACGGCCTGGGCTACACACGTGCTACAATGGTCGGTACAAAGGGTTGCCA
AGCCGCGAGGTGGAGCTAATCCCATAAAACCGATCGTAGTCCGGATCGCAGTCTGCAACTCGACTGCGTGAAGTCGGAATCGCTAGTAATCGCGAATCAG
AATGTCGCGGTGAATACGTTCCCGGGCCTTGTACACACCGCCCGTCACACCATGGGAGTGGGTTGCACCAGAAGTAGCTAGTCTAACCTTCGGGAGGACG
G

>EB292
TCGTAACAAGGTAACCGTAGAGTTTGATTATGGCTCATGAAGTCGTATGAGGTACCCGCCTAGTTTGAGGTATAACTTCGGGAAACCGGAGCTAATACCG
GAACGTTCTTTTCTCGCATGAGAGAAGATGGAAAGACGGTTTACGCTGTCACTTATAGATGGGCCCGCGGCGCATTAACTAGTTGGTGAGGTAATGGCTC
ACCAAGGCGACGATGCGTAGCCGACCTGAGAGGGTGATCGGCCACACTGGGACTGAGACACGGCCCAGACTCCTACGGGAGGCAGCAGTAGGGAATCTTC
CGCAATGGACGAAAGTCTGACGGAGCAACGCCGCGTGAACGAAGAAGGCCTTCGGGTCGTAAAGTTCTGTTGTTAGGGAAGAACAAGTACCAGAGTAACT
GCTGGTACCTTGACGGTACCTAACCAGAAAGCCACGGCTAACTACGTGCCAGCAGCCGCGGTAATACGTAGGTGGCAAGCGTTGTCCGGAATTATTGGGC
GTAAAGCGCGCGCAGGTGGTTCCTTAAGTCTGATGTGAAAGCCCACGGCTCAACCGTGGAGGGTCATTGGAAACTGGGGAACTTGAGTGCAGAAGAGGAA
AGTGGAATTCCAAGTGTAGCGGTGAAATGCGTAGAGATTTGGAGGAACACCAGTGGCGAAGGCGACTTTCTGGTCTGTAACTGACACTGAGGCGCGAAAG
CGTGGGGAGCAAACAGGATTAGATACCCTGGTAGTCCACGCCGTAAACGATGAGTGCTAAGTGTTAGAGGGTTTCCGCCCTTTAGTGCTGCAGCTAACGC
ATTAAGCACTCCGCCTGGGGAGTACGGCCGCAAGGCTTGAAACTCAAAGGAATTGACGGGGGCCCGCACAAGCGGTGGAGCATGTGGTTTAATTCGAAGC
AACGCGAAGAACCTTACCAGGTCTTGACATCCTCTGACAACCCTAGAGATAGGGCTTTCCCCTTCGGGGGACAGAGTGACAGGTGGTGCATGGTTGTCGT
CAGCTCGTGTCGTGAGATGTTGGGTTAAGTCCCGCAACGAGCGCAACCCTTGATTTTAGTTGCCAGCATTCAGTTGGGCACTCTAAGGTGACTGCCGGTG
ACAAACCGGAGGAAGGTGGGGATGACGTCAAATCATCATGCCCCTTATGACCTGGGCTACACACGTGCTACAATGGATGGTACAAAGGGCTGCAAACCTG
CGAAGGTAAGCGAATCCCATAAAGCCATTCTCAGTTCGGATTGCAGGCTGCAACTCGCCTGCATGAAGCCGGAATCGGTAGTAATCGCGGATCAGCATGC
CGCGGTGAATACGTTCCCGGGCCTTGTACACCCCGCCCCTCACACCCC

>EB293
TGCAGTCGAGCGGATGACAAGAGCTTGCTCTTCCATTCGGCGGCGGACGGGTGAGTAATGCCTAGGAATCTGCCTGGCAGTGGGGGACAACGTTTCAAAA
GGAACGCTAATACCGCATACGTCCTACGGGAGAAAGCAGGGGACCTTCGGGCCTTGCGCTATCAGATGAGCCTAGGTCGGATTAGCTAGTTGGTGGGGTA
ATGGCTCACCAAGGCGACGATCCGTAACTGGTCTGAGAGGATGATCAGTCACACTGGAACTGAGACACGGTCCAGACTCCTACGGGAGGCAGCAGTGGGG
AATATTGGACAATGGGCGAAAGCCTGATCCAGCCATGCCGCGTGTGTGAAGAAGGTCTTCGGATTGTAAAGCACTTTAAGTTGGGAGGAAGGGCATTAAC
CTAATACGTTAGTGTTTTGACGTTACCCGACAGAATAAGCACCGGCTAACTCTGTGCCAGCAGCCGCGGTAATACAGAGGGTGCAAGCGTTAATCGGAAT
TACTGGGCGTAAAGCGCGCGTAGGTGGTTTGTTAAGTTGGATGTGAAAGCCCCGGGCTCAACCTGGGAACTGCATCCAAAACTGGCAAGCTAGAGTACGG
TAGAGGGTGGTGGAATTTCCTGTGTAGCGGTGAAATGCGTAGATATAGGAAGGAACACCAGTGGCGAAGGCGACCACCTGGACTGATACTGACACTGAGG
TGCTAAAGCGTGGGGAGCAAACAAGATTAGATACCCTGGTAGTCCACGCCGTAAACGATGTCAACTAGCCGTTGGAATCCTTGAGATTTTAGTGGCGCAG
CTAACGCATTAAGTTGACCGCCTGGGGAGTACGGCCGCAAGGTTAAAACTCAAATGAATTGAACGGGGGCCCGCACAACCGGTGGAGCATGTGGATTAAT
TCGAAGCAACGCGAAGAACCTTACCAGGCCTTGACATGCAGAGAACTTTCCAGAGATGGATTGGTGCCTTCGGGAACTCTGACACAGGTGCTGCATGGCT
GTCGTCAGCTCGTGTCGTGAGATGTTGGGTTAAGTCCCGTAACGAGCGCAACCCTTGTCCTTAGTTACCACCACGTTATGGTGGGCACTCTAAGGAGACT
GCCGGTGACAAACCGGAGGAAGGTGGGGATGACGTCAAGTCATCATGGCCCTTACGGCATGGGCTACCCACGTGCTCCAATGGTCGGTACAGAGGGTTGC
CAAGCCGCGAGGCGGAGCTAATCTCACAAAACCGATCGTAGTCCGGATCGCAGTCTGCAACTCGATTGCGTGAAGTCGGAATCGCTAGTAATCGCGAATC
AGAATGTCGCGGTGAATACGTTCCCGGGCCTTGTACACACCGCCCGTCACACCATGGGAGTGGGTTGCACCAGAAGTAGGTAGTTTAACCTTCGGGAGGA
CGGT

>EB294
CGAGCGGTAGAGAGAAGCTTGCTTCTCTTGAGAGCGGCGGACGGGTGAGTAATGCCTAGGAATCTGCCTGGTAGTGGGGGATAACGTTCGGAAACGAACG
CTAATACCGCATACGTCCTACGGGAGAAAGCAGGGGACCTTCGGGCCTTGCGCTATCAGATGAGCCTAGGTCGGATTAGCTAGTTGGTGAGGTAATGGCT
CACCAAGGCGACGATCCGTAACTGGTCTGAGAGGATGATCAGTCACACTGGAACTGAGACACGGTCCAGACTCCTACGGGAGGCAGCAGTGGGGAATATT
GGACAATGGGCGAAAGCCTGATCCAGCCATGCCGCGTGTGTGAAGAAGGTCTTCGGATTGTAAAGCACTTTAAGTTGGGAGGAAGGGCATTAACCTAATA
CGTTGGTGTCTTGACGTTACCGACAGAATAAGCACCGGCTAACTCTGTGCCAGCAGCCGCGGTAATACAGAGGGTGCAAGCGTTAATCGGAATTACTGGG
CGTAAAGCGCGCGTAGGTGGTTTGTTAAGTTGGATGTGAAATCCCCGGGCTCAACCTGGGAACTGCATTCAAAACTGACAAGCTAGAGTATGGTAGAGGG
TGGTGGAATTTCCTGTGTAGCGGTGAAATGCGTAGATATAGGAAGGAACACCAGTGGCGAAGGCGACCACCTGGACTGATACTGACACTGAGGTGCGAAA
GCGTGGGGAGCAAACAGGATTAGATACCCTGGTAGTCCACGCCGTAAACGATGTCAACTAGCCGTTGGGAGCCTTGAGCTCTTAGTGGCGCAGCTAACGC
ATTAAGTTGACCGCCTGGGGAGTACGGCCGCAAGGTTAAAACTCAAATGAATTGACGGGGGCCCGCACAAGCGGTGGAGCATGTGGTTTAATTCGAAGCA
ACGCGAAGAACCTTACCAGGCCTTGACATCCAATGAACTTTCCAGAGATGGATGGGTGCCTTCGGGAACATTGAGACAGGTGCTGCATGGCTGTCGTCAG
CTCGTGTCGTGAGATGTTGGGTTAAGTCCCGTAACGAGCGCAACCCTTGTCATTAGTTACCAGCACGTTATGGTGGGCACTCTAAGGAGACTGCCGGTGA
CAAACCGGAGGAAGGTGGGGATGACGTCAAGTCATCATGGCCCTTACGGCCTGGGCTACACACGTGCTACAATGGTCGGTACAGAGGGTTGCCAAGCCGC
GAGGTGGAGCTAATCCCAGAAAACCGATCGTAGTCCGGATCGCAGTCTGCAACTCGACTGCGTGAAGTCGGAATCGCTAGTAATCGCGAATCAGAATGTC
GCGGTGAATACGTTCCCGGGCCTTGTACACACCGCCCGTCACACCATGGGAGTGGGTTGCACCAGAAGTAGCTAGTCTAACCTTCGGGAGGA

>EB295
CAGCTTGCTCCCTGAGATTAACGGCGGACGGGTGAGTACACGTGGGTAACCTGCCTGTAAGACTGGGATAACTCCGGGAAACCGGGGCTAATACCGGATA
ACTTTTTCCTTCGCATGAAGGAGAATTGAAAGATGGCTTTTAGCTATCACTTACGGATGGACCCGCGGCGCATTAGCTAGTTGGTGAGGTAACGGCTCAC
CAAGGSGACRRTCGGTAGCCRACTTGAGAGGGTGATCGGCCACACTGGGACTGAGACACGGCCCAGATTCCTACGGGAGGCAGCAGTAGGGAATCTTCCG
CAATGGACGAAAGTCTGACGGAGCAACGCCGCSTGAGTGATGAAGGTTTTCGGATCGTAAAACTCTGTTGTTAGGGAAGAACAAGTATCGTTCGAATAGG
GCGGTACCTTGACGGTACCTAACCAGAAAGCCACGGCTAACTACGTGCCAGCAGCCGCGGTAATACGTAGGTGGCAAGCGTTGTCCGGAATTATTGGGCG
TAAAGCGCGCGCAGGCGGTTTCTTAAGTCTGATGTGAAAGCCCACGGCTCAACCGTGGAGGGTCATTGGAAACTGGGAGACTTGAGTGCAGAAGAGAAGA
GCGGAATTCCACGTGTASCGGTGAAATGCGTAGAGATGTGGAGGAACACCAGTGGCGAAGGCGGCTCTTTGGTCTGTAACTGACGCTGAGGCGCGAAAGC
GTGGGGAGCGAACAGGATTAGATACCCTGGTAGTCCACGCCGTAAACGATGAGTGCTAAGTGTTAGAGGGTTTCCGCCCTTTAGTGCTGCAGCTAACGCA
TTAAGCACTCCGCCTGGGGAGTACGGCCGCAAGGCTGAAACTCAAAGGAATTGACGGGGGCCCGCACAAGCGGTGGAGCATGTGGTTTAATTCGAAGCAA
CGCGAAGAACCTTACCAGGTCTTGACATCCTCTTGACCTCCCTAGAGATAGGGATTTCCCTTCGGGGACAAGAGTGACAGGTGGTGCATGGTTGTCGTCA
GCTCGTGTCGTGAGATGTTGGGTTAAGTCCCGCAACGAGCGCAACCCTTGACCTTAGTTGCCAGCATTCAGTTGGGCACTCTAAGGTGACTGCCGGTGAC
AAACCGGAGGAAGGTGGGGATGACGTCAAATCATCATGCCCCTTATGACCTGGGCTACACACGTGCTACAATGGATGGTACAAAGGGTTGCTAGACCGCG

AGGTTACGCTAATCCCATAAAACCATTCTCAGTTCGGATTGTAGGCTGCAACTCGCCTACATGAAGCCGGAATCGCTAGTAATCGCGGATCAGCATGCCG
CGGTGAATACGTTCCCGGGCCTTGTACACACCGCCCGTCACACCACGAGAGTTTGTAACACCCGAAGTCGGTGAGGTAACCTTTGGAGCCAGCCG

>EB296
AAACTCGCGTGGTGTGACGGGCGGTGTGTACAAGGCCCGGGAACGTATTCACCGCGGCATGCTGATCCGCGATTACTAACGATTCCGGCTTCATGCAGGC
GAGTTGCAGCCTGCAATCCGAACTGAGAATGGTTTTATGGGATTGGCTTCACCTCGCGGCTTCGCTGCCCTTTGTTCCATCCATTGTAGCACGTGTGTAG
CCCAGGTCATAAGGGGCATGATGATTTGACGTCATCCCCACCTTCCTCCGGTTTGTCACCGGCAGTCACCTTAGAGTGCCCAACTTAATGCTGGCAACTA
AGATCAAGGGTTGCGCTCGTTGCGGGACTTAACCCAACATCTCACGACACGAGCTGACGACAACCATGCACCACCTGTCACTCTGTCCCCCGAAGGGGAA
AGCCCTATCTCTAGGGTTGTCAGAGGATGTCAAGACCTGGTAAGGTTCTTCGCGTTGCTTCGAATTAAACCACATGCTCCACCGCTTGTGCGGGCCCCCG
TCAATTCCTTTGAGTTTCAGCCTTGCGGCCGTACTCCCCAGGCGGAGTGCTTAATGCGTTTGCTGCAGCACTAAAGGGCGGAAACCCTCTAACACTTAGC
ACTCATCGTTTACGGCGTGGACTACCAGGGTATCTAATCCTGTTCGCTCCCCACGCTTTCGCGCCTCAGCGTCAGTTACAGACCAGAGAGCCGCCTTCGC
CACTGGTGTTCCTCCACATCTCTACGCATTTCACCGCTACACGTGGAATTCCGCTCTCCTCTTCTGCACTCAAGTTCCCCAGTTTCCAATGACCCTCCCC
GGTTGAGCCGGGGGCTTTCACATCAGACTTAAGGAACCGCCTGCGCGCGCTTTACGCCCAATAATTCCGGACAACGCTTGCCACCTACGTATTACCGCGG
CTGCTGGCACGTAGTTAGCCGTGGCTTTCTGGTCAGGTACCGTCAAGGTACCGGCAGTTACTCCGATACTTGTTCTTCCCTGACAACAGAGCTTTACGAC
CCGAAGGCCTTCATCGCTCACGCGGCGTTGCTCCGTCAGACTTTCGTCCATTGCGGAAGATTCCCTACTGCTGCCTCCCGTAGGAGTCTGGGCCGTGTCT
CAGTCCCAGTGTGGCCGATCACCCTCTCAGGTCGGCTACGCATCGTTGCCTTGGTGAGCCGTTACCTCACCAACTAGCTAATGCGCCGCGGGCCCATCTG
TAAGTGACAGCCGAAACCGTCTTTCAGCTTTCCCTCATGTGAGGGAAAGGATTATCCGGTATTAGCTCCGGTTTCCCGAAGTTATCCCAGTCTTACAGGC
AGGTTGCCCACGTGTTACTCACCCGTCCGCCGAAGAT

>EB297
GCGGGGATCGTAGCTTGCTACTTGACCTAGCGGCGGACGGGTGAGTAATGCTTAGGAATCTGCCTATTAGTGGGGGACAACATCTCGAAAGGGATGCTAA
TACCGCATACGTCCTACGGGAGAAAGCAGGGGACCTTCGGGCCTTGCGCTAATAGATGAGCCTAAGTCGGATTAGCTAGTTGGTGGGGTAAAGGCCTACC
AAGGCGACGATCTGTAGCGGGTCTGAGAGGATGATCCGCCACACTGGGACTGAGACACGGCCCAGACTCCTACGGGAGGCAGCAGTGGGGAATATTGGAC
AATGGGGGGAACCCTGATCCAGCCATGCCGCGTGTGTGAAGAAGGCCTTTTGGTTGTAAAGCACTTTAAGCGAGGAGGAGGCTACCGAGATTAATACTCT
TGGATAGTGGACGTTACTCGCAGAATAAGCACCGGCTAACTCTGTGCCAGCAGCCGCGGTAATACAGAGGGTGCAAGCGTTAATCGGATTTACTGGGCGT
AAAGCGCGCGTAGGTGGCCAATTAAGTCAAATGTGAAATCCCCGAGCTTAACTTGGGAATTGCATTCGATACTGGTTGGCTAGAGTATGGGAGAGGATGG
TAGAATTCCAGGTGTAGCGGTGAAATGCGTAGAGATCTGGAGGAATACCGATGGCGAAGGCAGCCATCTGGCCTAATACTGACACTGAGGTGCGAAAGCA
TGGGGAGCAAACAGGATTAGATACCCTGGTAGTCCATGCCGTAAACGATGTCTACTAGCCGTTGGGGCCTTTGAGGCTTTAGTGGCGCAGCTAACGCGAT
AAGTAGACCGCCTGGGGAGTACGGTCGCAAGACTAAAACTCAAATGAATTGACGGGGGCCCGCACAAGCGGTGGAGCATGTGGTTTAATTCGATGCAACG
CGAAGAACCTTACCTGGTCTTGACATAGTAAGAACTTTCCAGAGATGGATTGGTGCCTTCGGGAACTTACATACAGGTGCTGCATGGCTGTCGTCAGCTC
GTGTCGTGAGATGTTGGGTTAAGTCCCGCAACGAGCGCAACCCTTTTCCTTATTTGCCAGCGGGTTAAGCCGGGAACTTTAAGGATACTGCCAGTGACAA
ACTGGAGGAAGGCGGGGACGACGTCAAGTCATCATGGCCCTTACGACCAGGGCTACACACGTGCTACAATGGTCGGTACAAAGGGTTGCTACCTCGCGAG
AGGATGCTAATCTCAAAAAGCCGATCGTAGTCCGGATTGGAGTCTGCAACTCGACTCCATGAAGTCGGAATCGCTAGTAATCGCGGATCAGAATGCCGCG
GTGAATACGTTCCCGGGCCTTGTACACACCGCCCGTCACACCATGGGAGTTTGTTGCTACAGAAGTAGGT

>EB298
CCGTCCTCCGAAGGTTAGACTAGCTACTTCTGGTGCAACCCACTCCCATGGTGTGACGGGCGGTGTGTACAAGGCCCGGGAACGTATTCACCGCGACATT
CTGATTCGCGATTACTAGCGATTCCGACTTCACGCAGTCGAGTTGCAGACTGCGATCCGGACTACGATCGGTTTTATGGGATTAGCTCCACCTCGCGGCT
TGGCAACCCTTTGTACCGACCATTGTAGCACGTGTGTAGCCCAGGCCGTAAGGGCCATGATGACTTGACGTCATCCCCACCTTCCTCCGGTTTGTCACCG
GCAGTCTCCTTAGAGTGCCCACCATAACGTGCTGGTAACTAAGGACAAGGGTTGCGCTCGTTACGGGACTTAACCCAACATCTCACGACACGAGCTGACG
ACAGCCATGCAGCACCTGTCTCAATGTTCCCGAAGGCACCAATCTATCTCTAGAAAGTTCATTGGATGTCAAGGCCTGGTAAGGTTCTTCGCGTTGCTTC
GAATTAAACCACATGCTCCACCGCTTGTGCGGGCCCCCGTCAATTCATTTGAGTTTTAACCTTGCGGCCGTACTCCCCAGGCGGTCAACTTAATGCGTT
AGCTGCGCCACTAAGAGCTCAAGGCTCCCAACGGCTAGTTGACATCGTTTACGGCGTGGACTACCAGGGTATCTAATCCTGTTTGCTCCCCACGCTTTCG
CACCTCAGTGTCAGTATCAGTCCAGGTGGTCGCCTTCGCCACTGGTGTTCCTTCCTATATCTACGCATTTCACCGCTACACAGGAAATTCCACCACCCTC
TACCATACTCTAGCTTGCCAGTTTTGGATGCAGTTCCCAGGTTGAGCCCGGGGCTTTCACATCCAACTTAACAAACCACCTACGCGCGCTTTACGCCCAG
TAATTCCGATTAACGCTTGCACCCTCTGTATTACCGCGGCTGCTGGCACAGAGTTAGCCGGTGCTTATTCTGTCGGTAACGTCAAAACAATCACGTATTA
GGTAACTGCCCTTCCTCCCAACTTAAAGTGCTTTACAATCCGAAGACCTTCTTCACACACGCGGCATGGCTGGATCAGGCTTTCGCCCATTGTCCAATAT
TCCCCACTGCTGCCTCCCGTAGGAGTCTGGACCGTGTCTCAGTTCCAGTGTGACTGATCATCCTCTCAGACCAGTTACGGATCGTCGCCTTGGTGAGCCA
TTACCTCACCAACTAGCTAATCCGACCTAGGCTCATCTAATAGCGTGAGGTCCGAAGATCCCCCACTTTCTCCCGTAGGACGTATGCGGTATTAGCGCCC
GTTTCCGGACGTTATCCCCCACTACTAGGCAGATTCCTAGGCATTACTCACCCGTCCGCCGCTCGCCACCAGGTACAAGTACCCGTGCTGCCGCTCGACT
GCATGTGTAGC

>EB299
GTCGAGCGAATGGATGGGAGCTTGCTCCCTGAAGTTAGCGGCGGACGGGTGAGTAACACGTGGGCAACCTACCTACAAGATTGGGATAACTTCGGGAAAC
CGGAGCTAATACCGGGTAATACATCGGATTGCATGATTCGATGTTGAAAGATGGCTTCGGCTATCACTTGTAGATGGGCCCGCGGCGCATTAGCTAGTTG
GTGAGGTAACGGCTCACCAAGGCAACGATGCGTAGCCGACCTGAGAGGGTGATCGGCCACACTGGGACTGAGACACGGCCCAGACTCCTACGGGAGGCAG
CAGTAGGGAATCTTCCGCAATGGACGAAAGTCTGACGGAGCAACGCCGCGTGAGTGATGAAGGCCTTCGGGTCGTAAAGCTCTGTTGTTAGGGAAGAACA
AGTACCGTTTGAATAAGGCGGTACCTTGACGGTACCTAACCAGAAAGCCACGGCTAACTACGTGCCAGCAGCCGCGGTAATACGTAGGTGGCAAGCGTTA
TCCGGAATTATTGGGCGTAAAGCGCGCGCAGGCGGTTCCTTAAGTCTGATGTGAAAGCCCACGGCTCAACCGTGGAGGGTCATTGGAAACTGGGGAACTT
GAGTGCAGAAGAGAAGAGCGGAATTCCACGTGTAGCGGTGAAATGCGTAGAGATGTGGAGGAACACCAGTGGCGAAGGCGGCTCTTTGGTCTGTAACTGA
CGCTGAGGCGCGAAAGCGTGGGGAGCAAACAGGATTAGATACCCTGGTAGTCCACGCCGTAAACGATGAGTGCTAAGTGTTAGAGGGTTTCCGCCCTTTA
GTGCTGAAGTTAACGCATTAAGCACTCCGCCTGGGGAGTACGGTCGCAAGACTGAAACTCAAAGGAATTGACGGGGGCCCGCACAAGTGGTGGAGCATGT
GGTTTAATTCGAAGCAACGCGAAGAACCTTACCAGGTCTTGACATCCTCTGACAACTCTAGAGATAGAGCTTTCCCTTCGGGGACAGAGTGACAGGTGGT
GCATGGTTGTCGTCAGCTCGTGTCGTGAGATGTTGGGTTAAGTCCCGCAACGAGCGCAACCCTTGATCTTAGTTGCCAGCATTAAGTTGGGCACTCTAAG
GTGACTGCCGGTGACAAACCGGAGGAANGGTGGGGATGACGTCAAATCATCATGCCCCTTATGACCTGGGNCTACACACGTGCTACAATGGATGGTACAA
AGGGCTGCGAGACCGCGAGGTTAAGCGAATCCCATAAAACCATTCTCAGTTCGGATTGTAGGCTGCAACTCGCCTACATGAAGCCGGAATCACTAGTAAT
CGCGGATCAGCATGCCGCGGTGAATACGTTCCCGGGCCTTGTACACACCGCCCGTCACACCACGAGAGTTTGTAACACCCGAAGTCGGTGGGGTAACCCT
TTTGGGAGCCAGCCGCC

>EB300
TACCGTCCTCCGAGGGTTAGACTAGCTACTTCTGGTGCAACCCACTCCCATGGTGTGACGGGCGGTGTGTACAAGGCCCGGGAACGTATTCACCGCGACA
TTCTGATTCGCGATTACTAGCGATTCCGACTTCACGCAGTCGAGTTGCAGACTGCGATCCGGACTACGATCGGTTTTCTGGGATTAGCTCCACCTCGCGG
CTTGGCAACCCTCTGTACCGACCATTGTAGCACGTGTGTAGCCCAGGCCGTAAGGGCCATGATGACTTGACGTCATCCCCACCTTCCTCCGGTTTGTCAC
CGGCAGTCTCCTTAGAGTGCCCACCATAACGTGCTGGTAACTAAGGACAAGGGTTGCGCTCGTTACGGGACTTAACCCAACATCTCACGACACGAGCTGA
CGACAGCCATGCAGCACCTGTCTCAATGTTCCCGAAGGCACCGATTCCATCTCTGGAAAGTTCATTGGATGTCAAGGCCTGGTAAGGTTCTTCGCGTTGC
TTCGAATTAAACCACATGCTCCACCGCTTGTGCGGGCCCCCGTCAATTCATTTGAGTTTTAACCTTGCGGCCGTACTCCCCAGGCGGTCAACTTAATGCG
TTAGCTGCGCCACTAAGAGCTCAAGGCTCCCAACGGCTAGTTGACATCGTTTACGGCGTGGACTACCAGGGTATCTAATCCTGTTTGCTCCCCACGCTTT
CGCACCTCAGTGTCAGTATCAGTCCAGGTGGTCGCCTTCGCCACTGGTGTTCCTTCCTATATCTACGCATTTCACCGCTACACAGGAAATTCCACCACCC

TCTACCATACTCTAGCTTGTCAGTTTTGAATGCAGTTCCCAGGTTGAGCCCGGGGATTTCACATCCAACTTAACAAACCACCTACGCGCGCTTTACGCCC
AGTAATTCCGATTAACGCTTGCACCCTCTGTATTACCGCGGCTGCTGGCACAGAGTTAGCCGGTGCTTATTCTGTCGGTAACGTCAAGACACCAACGTAT
TAGGTTAATGCCCTTCCTCCCAACTTAAAGTGCTTTACAATCCGAAGACCTTCTTCACACACGCGGCATGGCTGGATCAGGCTTTCGCCCATTGTCCAAT
ATTCCCCACTGCTGCCTCCCGTAGGAGTCTGGACCGTGTCTCAGTTCCAGTGTGACTGATCATCCTCTCAGACCAGTTACGGATCGTCGCCTTGGTGAGC
CATTACCTCACCAACTAGCTAATCCGACCTAGGCTCATCTGATAGCGCAAGGCCCGAAGGTCCCCTGCTTTCTCCCGTAGGACGTATGCGGTATTAGCGT
TCGTTTCCGAACGTTATCCCCCACTACCAGGCAGATTCCTAGGCATTACTCACCCGTCCGCCGCTCTCAAGAGAAGCAAGCTTCTCTCTACCGCTCGACT
TGC

>EB301
GTCGAGCGGATGACAGGGAGCTTGCTCCTGGATTCAGCGGCGGACGGGTGAGTAATGCCTAGGAATCTGCCTGGTAGTGGGGGACAACGTTTCGAAAGGA
ACGCTAATACCGCATACGTCCTACGGGAGAAAGCAGGGGACCTTCGGGCCTTGCGCTATCAGATGAGCCTAGGTCGGATTAGCTAGTTGGTGAGGTAATG
GCTCACCAAGGCGACGATCCGTAACTGGTCTGAGAGGATGATCAGTCACACTGGAACTGAGACACGGTCCAGACTCCTACGGGAGGCAGCAGTGGGGAAT
ATTGGACAATGGGCGAAAGCCTGATCCAGCCATGCCGCGTGTGTGAAGAAGGTCTTCGGATTGTAAAGCACTTTAAGTTGGGAGGAAGGGCAGTAAATTA
ATACTTTGCTGTTTTGACGTTACCGACAGAATAAGCACCGGCTAACTCTGTGCCAGCAGCCGCGGTAATACAGAGGGTGCAAGCGTTAATCGGAATTACT
GGGCGTAAAGCGCGCGTAGGTGGTTTGTTAAGTTGGATGTGAAAGCCCCGGGCTCAACCTGGGAACTGCATTCAAAACTGACAAGCTAGAGTATGGTAGA
GGGTGGTGGAATTTCCTGTGTAGCGGTGAAATGCGTAGATATAGGAAGGAACACCAGTGGCGAAGGCGACCACCTGGACTGATACTGACACTGAGGTGCG
AAAGCGTGGGGAGCAAACAGGATTAGATACCCTGGTAGTCCACGCCGTAAACGATGTCAACTAGCCGTTGGGAGCCTTGAGCTCTTAGTGGCGCAGCTAA
CGCATTAAGTTGACCGCCTGGGGGAGTACGGCCGCAAGGTTAAAACTCAAATGAATTGACGGGGGCCCGCACAAGCGGTGGAGCATGTGGTTTAATTTC
GAAGCAACGCGAAGAACCTTACCAGGCCTTGACATCCAATGAACTTTCCAGAGATGGATTGGTGCCTTCGGGAACATTGAGACAGGTGCTGCATGGCTGT
CGTCAGCTCGTGTCGTGAGATGTTGGGTTAAGTCCCGTAACGAGCGCAACCCTTGTCCTTAGTTACCAGCACGTAATGGTGGGCACTCTAAGGAGACTGC
CGGTGACAAACCGGAGGAAGGTGGGGATGACGTCAAGTCATCATGGCCCTTACGGCCTGGGCTACACACGTGCTACAATGGTCGGTACAGAGGGTTGCCA
AGCCGCGAGGTGGAGCTAATCCCATAAAACCGATCGTAGTCCGGATCGCAGTCTGCAACTCGACTGCGTGAAGTCGGAATCGCTAGTAATCGCGAATCAG
AATGTCGCGGTGAATACGTTCCCGGGCCTTGTACACACCGCCCGTCACACCATGGGAGTGGGTTGCACCAGAAGTAGCTAGTCTAACCTTCGGGGGGACG
GT

>EB302
CATGAGTAACACGTGGGCAACCTGCCTGTAAGACTGGGATAACTTCGGGAAACCGGAGCTAATACCGGATAATCCTTTTCCTCTCATGAGGAAAAGCTGA
AAGACGGTTTCGGCTGTCACTTACAGATGGGCCCGCGGCGCATTAGCTAGTTGGTGAGGTAACGGCTCACCAAGGCGACGATGCGTAGCCGACCTGAGAG
GGTGATCGGCCACACTGGGACTGAGACACGGCCCAGACTCCTACGGGAGGCAGCAGTAGGGAATCTTCCGCAATGGACGAAAGTCTGACGGAGCAACGCC
GCGTGAGCGATGAAGGCCTTCGGGTCGTAAAGCTCTGTTGTCAGGGAAGAACAAGTACCGGAGTAACTGCCGGTACCTTGACGGTACCTGACCAGAAAGC
CACGGCTAACTACGTGCCAGCAGCCGCGGTAATACGTAGGTGGCAAGCGTTGTCCGGAANTTATTGGGCGTNAAAGCGCGCGCAGGCGGTTCCTTAAGTC
TGATGTGAAAGCCCCCGGCTCAACCGGGGAGGGTCATTGGAAACTGGGGAACTTGAGTGCAGAAGAGGAGAGCGGAATTCCACGTGTAGCGGTGAAATGC
GTAGAGATGTGGAGGAACACCAGTGGCGAAGGCGGCTCTCTGGTCTGTAACTGACGCTGAGGCGCGAAAGCGTGGGGAGCGAACAGGATTAGATACCCTG
GTAGTCCACGCCGTAAACGATGAGTGCTAAGTGTTAGAGGGTTTCCGCCCTTTAGTGCTGCAGCAAACGCATTAAGCACTCCGCCTGGGGAGTACGGCCG
CAAGGCTGAAACTCAAAGGAATTGACGGGGGCCCGCACAAGCGGTGGAGCATGTGGTTTAATTCGAAGCAACGCGAAGAACCTTACCAGGTCTTGACATC
CTCTGACAACCCTAGAGATAGGGCGTTCCCCTTCGGGGGACAGAGTGACAGGTGGTGCATGGTTGTCGTCAGCTCGTGTCGTGAGATGTTGGGTTAAGTC
CCGCAACGAGCGCAACCCTTGATCTTAGTTGCCAGCATTCAGTTGGGCACTCTAAGGTGACTGCCGGTGACAAACCGGAGGAAGGTGGGGATGACGTCAA
ATCATCATGCCCCTTATGACCTGGGCTACACACGTGCTACAATGGATGGAACAAAGGGCAGCGAAGCCGCGAGGTGAAGCCAATCCCATAAAACCATTCT
CAGTTCGGATTGCAGGCTGCAACTCGCCTGCATGAAGCCGGAATCGTTGGTAATCGCGGATCAGCATGCCGCGGTGACTACGTTCCCGTAGATTGTGCAC

>EB303
CTCAGGATGAACGCTGGCGGCGTGCTTAACACATGCAAGTCGAACGGTGAAGCCAAGCTTGCTTGGTGGATCAGTGGCGAACGGGTGAGTAACACGTGAG
CAACCTGCCCTGGACTCTGGGATAAGCGCTGGAAACGGCGTCTAATACTGGATATGAGCTCCTTCCGCATGGTGGGGGTTGGAAAGATTTTTCGGTCTGG
GATGGGCTCGCGGCCTATCAGCTTGTTGGTGAGGTAATGGCTCACCAAGGCGTCGACGGGTAGCCGGCCTGAGAGGGTGACCGGCCACACTGGGACTGAG
ACACGGCCCAGACTCCTACGGGAGGCAGCAGTGGGGAATATTGCACAATGGGCGGAAGCCTGATGCAGCAACGCCGCGTGAGGGATGACGGCCTTCGGGT
TGTAAACCTCTTTTAGCAGGGAAGAAGCGAAAGTGACGGTACCTGCAGAAAAAGCGCCGGCTAACTACGTGCCAGCAGCCGCGGTAATACGTAGGGCGCA
AGCGTTATCCGGAATTATTGGGCGTAAAGAGCTCGTAGGCGGTTTGTCGCGTCTGCTGTGAAATCCCGAGGCTCAACCTCGGGCCTGCAGTGGGTACGGG
CAGACTAGAGTGCGGTAGGGGAGATTGGAATTCCTGGTGTAGCGGTGGAATGCGCAGATATCAGGAGGAACACCGATGGCGAAGGCAGATCTCTGGGCCG
TAACTGACGCTGAGGAGCGAAAGGGTGGGGAGCAAACAGGCTTAGATACCCTGGTAGTCCACCCCGTAAACGTTGGGAACTAGTTGTGGGGACCATTCCA
CGGTTTCCGTGACGCAGCTAACGCATTAAGTTCCCCGCCTGGGGAGTACGGCCGCAAGGCTAAAACTCAAAGGAATTGACGGGGACCCGCACAAGCGGCG
GAGCATGCGGATTAATTCGATGCAACGCGAAGAACCTTACCAAGGCTTGACATATACGAGAACGGGCCAGAAATGGTCAACTCTTTGGACACTCGTAAAC
AGGTGGTGCATGGTTGTCGTCAGCTCGTGTCGTGAGATGTTGGGTTAAGTCCCGCAACGAGCGCAACCCTCGTTCTATGTTGCCAGCACGTAATGGTGGG
AACTCATGGGATACTGCCGGGGTCAACTCGGAGGAAGGTGGGGATGACGTCAAATCATCATGCCCCTTATGTCTTGGGCTTCACGCATGCTACAATGGCC
GGTACAAAGGGCTGCAATACCGTAAGGTGGAGCGAATCCCAAAAAGCCGGTCCCAGTTCGGATTGAGGTCTGCAACTCGACCTCATGAAGTCGGAGTCGC
TAGTAATCGCAGATCAGCAACGCTGCGGTGAATACGTTCCCGGGTCTTGTACACACCGCCCGTCAAGTCATGAAAGTCGGTAACACCTGAAGCCGGTGGC
CCAACCCTTGTGGAAGGAGCCGTCCAAAGTGGGATCGGTATTAGGATAATCGACA

>EB304
ACCGTCCTCCCGAAGGTTAGACTAGCTACTTCTGGTGCAACCCACTCCCATGGTGTGACGGGCGGTGTGTACAAGGCCCGGGAACGTATTCACCGCGACA
TTCTGATTCGCGATTACTAGCGATTCCGACTTCACGCAGTCGAGTTGCAGACTGCGATCCGGACTACGATCGGTTTTATGGGATTAGCTCCACCTCGCGG
CTTGGCAACCCTTTGTACCGACCATTGTAGCACGTGTGTAGCCCAGGCCGTAAGGGCCATGATGACTTGACGTCATCCCCACCTTCCTCCGGTTTGTCAC
CGGCAGTCTCCTTAGAGTGCCCACCATAACGTGCTGGTAACTAAGGACAAGGGTTGCGCTCGTTACGGGACTTAACCCAACATCTCACGACACGAGCTGA
CGACAGCCATGCAGCACCTGTCTCAATGTTCCCGAAGGCACCAATCTATCTCTAGAAAGTTCATTGGATGTCAAGGCCTGGTAAGGTTCTTCGCGTTGCT
TCGAATTAAACCACATGCTCCACCGCTTGTGCGGGCCCCCGTCAATTCATTTGAGTTTTAACCTTGCGGCCGTACTCCCCAGGCGGTCAACTTAATGCGT
TAGCTGCGCCACTAAGAGCTCAAGGCTCCCAACGGCTAGTTGACATCGTTTACGGCGTGGACTACCAGGGTATCTAATCCTGTTTGCTCCCCACGCTTTC
GCACCTCAGTGTCAGTATCAGTCCAGGTGGTCGCCTTCGCCACTGGTGTTCCTTCCTATATCTACGCATTTCACCGCTACACAGGAAATTCCACCACCCT
CTACCATACTCTAGCTTGCCAGTTTTGGATGCAGTTCCCAGGTTGAGCCCGGGGCTTTCACATCCAACTTAACAAACCACCTACGCGCGCTTTACGCCCA
GTAATTCCGATTAACGCTTGCACCCTCTGTATTACCGCGGCTGCTGGCACAGAGTTAGCCGGTGCTTATTCTGTCGGTAACGTCAAAACAATCACGTATT
AGGTAACTGCCCTTCCTCCCAACTTAAAGTGCTTTACAATCCGAAGACCTTCTTCACACACGCGGCATGGCTGGATCAGGCTTTCGCCCATTGTCCAATA
TTCCCCACTGCTGCCTCCCGTAGGAGTCTGGACCGTGTCTCAGTTCCAGTGTGACTGATCATCCTCTCAGACCAGTTACGGATCGTCGCCTTGGTGAGCC
ATTACCTCACCAACTAGCTAATCCGACCTAGGCTCATCTAATAGCGTGAGGTCCGAAGATCCCCCACTTTCTCCCGTAGGACGTATGCGGTATTAGCGCC
CGTTTCCGGACGTTATCCCCCACTACTAGGCAGATTCCTAGGCATTACTCACCCGTCCGCCGCTCGCCACCAGGTACAAGTACCCGTGCTGCCGCTCGAC
TGCA

>EB305
GTCGAGCGGATGAAAGGAGCTTGCTCCTGGATTCAGCGGCGGACGGGTGAGTAATGCCTAGGAATCTGCCTGGTAGTGGGGGACAACGTTTCGAAAGGAA
CGCTAATACCGCATACGTCCTACGGGAGAAAGCAGGGGACCTTCGGGCCTTGCGCTATCAGATGAGCCTAGGTCGGATTAGCTAGTTGGTGAGGTAATGG
CTCACCAAGGCGACGATCCGTAACTGGTCTGAGAGGATGATCAGTCACACTGGAACTGAGACACGGTCCAGACTCCTACGGGAGGCAGCAGTGGGGAATA

TTGGACAATGGGCGAAAGCCTGATCCAGCCATGCCGCGTGTGTGAAGAAGGTCTTCGGATTGTAAAGCACTTTAAGTTGGGAGGAAGGGCAGTAAATTAA
TACTTTGCTGTTTTGACGTTACCGACAGAATAAGCACCGGCTAACTCTGTGCCAGCAGCCGCGGTAATACAGAGGGTGCAAGCGTTAATCGGAATTACTG
GGCGTAAAGCGCGCGTAGGTGGTTCGTTAAGTTGGATGTGAAATCCCCGGGCTCAACCTGGGAACTGCATCCAAAACTGGCGAGCTAGAGTATGGTAGAG
GGTGGTGGAATTTCCTGTGTAGCGGTGAAATGCGTAGATATAGGAAGGAACACCAGTGGCGAAGGCGACCACCTGGACTGATACTGACACTGAGGTGCGA
AAGCGTGGGGAGCAAACAGGATTAGATACCCTGGTAGTCCACGCCGTAAACGATGTCAACTAGCCGTTGGGAGCCTTGAGCTCTTAGTGGCGCAGCTAAC
GCATTAAGTTGACCGCCTGGGGAGTACGGCCGCAAGGTTAAAACTCAAATGAATTGACGGGGGCCCGCACAAGCGGTGGAGCATGTGGTTTAATTCGAAG
CAACGCGAAGAACCTTACCAGGCCTTGACATCCAATGAACTTTCCAGAGATGGATTGGTGCCTTCGGGAACATTGAGACAGGTGCTGCATGGCTGTCGTC
AGCTCGTGTCGTGAGATGTTGGGTTAAGTCCCGTAACGAGCGCAACCCTTGTCCTTAGTTACCAGCACGTAATGGTGGGCACTCTAAGGAGACTGCCGGT
GACAAACCGGAGGAAGGTGGGGATGACGTCAAGTCATCATGGCCCTTACGGCCTGGGCTACACACGTGCTACAATGGTCGGTACAAAGGGTTGCCAAGCC
GCGAGGTGGAGCTAATCCCATAAAACCGATCGTAGTCCGGATCGCAGTCTGCAACTCGACTGCGTGAAGTCGGAATCGCTAGTAATCGCGAATCAGAATG
TCGCGGTGAATACGTTCCCGGGCCTTGTACACACCGCCCGTCACACCATGGGAGTGGGTTGCACCAGAAGTAGCTAGTCTAACCTTCGGGGG

>EB306
TGCAAGTCGAGCGGTAGAGAGAAGCTTGCTTCTCTTGAGAGCGGCGGACGGGTGAGTAATGCCTAGGAATCTGCCTGGTAGTGGGGGATAACGTCCGGAA
ACGGACGCTAATACCGCATACGTCCTACGGGAGAAAGCAGGGGACCTTCGGGCCTTGCGCTATCAGATGAGCCTAGGTCGGATTAGCTAGTTGGTGGGGT
AATGGCTCACCAAGGCGACGATCCGTAACTGGTCTGAGAGGATGATCAGTCACACTGGAACTGAGACACGGTCCAGACTCCTACGGGAGGCAGCAGTGGG
GAATATTGGACAATGGGCGAAAGCCTGATCCAGCCATGCCGCGTGTGTGAAGAAGGTCTTCGGATTGTAAAGCACTTTAAGTTGGGAGGAAGGGCCATTA
CCTAATACGTGATGGTTTTGACGTTACCGACAGAATAAGCACCGGCTAACTCTGTGCCAGCAGCCGCGGTAATACAGAGGGTGCAAGCGTTAATCGGAAT
TACTGGGCGTAAAGCGCGCGTAGGTGGTTTGTTAAGTTGGATGTGAAATCCCCGGGCTCAACCTGGGAACTGCATTCAAAACTGACTGACTAGAGTATGG
TAGAGGGTGGTGGAATTTCCTGTGTAGCGGTGAAATGCGTAGATATAGGAAGGAACACCAGTGGCGAAGGCGACCACCTGGACTGATACTGACACTGAGG
TGCGAAAGCGTGGGGAGCAAACAGGATTAGATACCCTGGTAGTCCACGCCGTAAACGATGTCAACTAGCCGTTGGGAGCCTTGAGCTCTTAGTGGCGCAG
CTAACGCATTAAGTTGACCGCCTGGGGAGTACGGCCGCAAGGTTAAAACTCAAATGAATTGACGGGGGCCCGCACAAGCGGTGGAGCATGTGGTTTAATT
CGAAGCAACGCGAAGAACCTTACCAGGCCTTGACATCCAATGAACTTTCTAGAGATAGATTGGTGCCTTCGGGAACATTGAGACAGGTGCTGCATGGCTG
TCGTCAGCTCGTGTCGTGAGATGTTGGGTTAAGTCCCGTAACGAGCGCAACCCTTGTCCTTAGTTACCAGCACGTTATGGTGGGCACTCTAAGGAGACTG
CCGGTGACAAACCGGAGGAAGGTGGGGATGACGTCAAGTCATCATGGCCCTTACGGCCTGGGCTACACACGTGCTACAATGGTCGGTACAAAGGGTTGCC
AAGCCGCGAGGTGGAGCTAATCCCATAAAACCGATCGTAGTCCGGATCGCAGTCTGCAACTCGACTGCGTGAAGTCGGAATCGCTAGTAATCGCGAATCA
GAATGTCGCGGTGAATACGTTCCCGGGCCTTGTACACACCGCCCGTCACACCATGGGAGTGGGTTGCACCAGAAGTAGCTAGTCTAACCTTCGGGGGGAC
GGTA

>EB307
ACCGTCCTCCCGAAGGTTAGACTAGCTACTTCTGGTGCAACCCACTCCCATGGTGTGACGGGCGGTGTGTACAAGGCCCGGGAACGTATTCACCGCGACA
TTCTGATTCGCGATTACTAGCGATTCCGACTTCACGCAGTCGAGTTGCAGACTGCGATCCGGACTACGATCGGTTTTGTGAGATTAGCTCCACCTCGCGG
CTTGGCAACCCTCTGTACCGACCATTGTAGCACGTGTGTAGCCCAGGCCGTAAGGGCCATGATGACTTGACGTCATCCCCACCTTCCTCCGGTTTGTCAC
CGGCAGTCTCCTTAGAGTGCCCACCATAACGTGCTGGTAACTAAGGACAAGGGTTGCGCTCGTTACGGGACTTAACCCAACATCTCACGACACGAGCTGA
CGACAGCCATGCAGCACCTGTCTCAGTGTTCCCGAAGGCACCAAACCATCTCTGGTAAGTTCACTGGATGTCAAGGCCTGGTAAGGTTCTTCGCGTTGCT
TCGAATTAAACCACATGCTCCACCGCTTGTGCGGGCCCCCGTCAATTCATTTGAGTTTTAACCTTGCGGCCGTACTCCCCAGGCGGTCAACTTAATGCGT
TAGCTGCGCCACTAAAATCTCAAGGATTCCAACGGCTAGTTGACATCGTTTACGGCGTGGACTACCAGGGTATCTAATCCTGTTTGCTCCCCACGCTTTC
GCACCTCAGTGTCAGTATGAGCCCAGGTGGTCGCCTTCGCCACTGGTGTTCCTTCCTATATCTACGCATTTCACCGCTACACAGGAAATTCCACCACCCT
CTGCCCTACTCTAGCTTGCCAGTTTTGGATGCAGTTCCCAGGTTGAGCCCGGGGATTTCACATCCAACTTAACAAACCACCTACGCGCGCTTTACGCCCA
GTAATTCCGATTAACGCTTGCACCCTCTGTATTACCGCGGCTGCTGGCACAGAGTTAGCCGGTGCTTATTCTGTCGGTAACGTCAAAACTGCTTCGTATT
AGGAAACAACCCTTCCTCCCAACTTAAAGTGCTTTACAATCCGAAGACCTTCTTCACACACGCGGCATGGCTGGATCAGGCTTTCGCCCATTGTCCAATA
TTCCCCACTGCTGCCTCCCGTAGGAGTCTGGACCGTGTCTCAGTTCCAGTGTGACTGATCATCCTCTCAGACCAGTTACGGATCGTCGCCTTGGTGAGCC
ATTACCTCACCAACTAGCTAATCCGACCTAGGCTCATCTGATAGCGCAAGGCCCGAAGGTCCCCTGCTTTCTCCCGTAGGACGTATGCGGTATTAGCGTT
CCTTTCGAAACGTTGTCCCCCACTACCAGGCAGATTCCTAGGCATTACTCACCCGTCCGCCGCTGAATCGAAGAGCAAGCTCTTCTCATCCGCTCGACTT
GCA

>EB308
TGCAGTCGAGCGGATGAAGGGAGCTTGCTCCTGGATTCAGCGGCGGACGGGTGAGTAATGCCTAGGAATCTGCCTGGTAGTGGGGGACAACGTTTCGAAA
GGAACGCTAATACCGCATACGTCCTACGGGAGAAAGCAGGGGACCTTCGGGCCTTGCGCTATCAGATGAGCCTAGGTCGGATTAGCTAGTTGGTGAGGTA
ATGGCTCACCAAGGCGACGATCCGTAACTGGTCTGAGAGGATGATCAGTCACACTGGAACTGAGACACGGTCCAGACTCCTACGGGAGGCAGCAGTGGGG
AATATTGGACAATGGGCGAAAGCCTGATCCAGCCATGCCGCGTGTGTGAAGAAGGTCTTCGGATTGTAAAGCACTTTAAGTTGGGAGGAAGGGTTGTAGA
TTAATACTCTGCAATTTTGACGTTACCGACAGAATAAGCACCGGCTAACTCTGTGCCAGCAGCCGCGGTAATACAGAGGGTGCAAGCGTTAATCGGAATT
ACTGGGCGTAAAGCGCGCGTAGGTGGTTCGTTAAGTTGGATGTGAAATCCCCGGGCTCAACCTGGGAACTGCATCCAAAACTGGCGAGCTAGAGTATGGT
AGAGGGTGGTGGAATTTCCTGTGTAGCGGTGAAATGCGTAGATATAGGAAGGAACACCAGTGGCGAAGGCGACCACCTGGACTGATACTGACACTGAGGT
GCGAAAGCGTGGGGAGCAAACAGGATTAGATACCCTGGTAGTCCACGCCGTAAACGATGTCAACTAGCCGTTGGGAGCCTTGAGCTCTTAGTGGCGCAGC
TAACGCATTAAGTTGACCGCCTGGGGAGTACGGCCGCAAGGTTAAAACTCAAATGAATTGACGGGGGCCCGCACAAGCGGTGGAGCATGTGGTTTAATT
CGAAGCAACGCGAAGAACCTTACCAGGCCTTGACATCCAATGAACTTTCCAGAGATGGATTGGTGCCTTCGGGAACATTGAGACAGGTGCTGCATGGCTG
TCGTCAGCTCGTGTCGTGAGATGTTGGGTTAAGTCCCGTAACGAGCGCAACCCTTGTCCTTAGTTACCAGCACGTTATGGTGGGCACTCTAAGGAGACTG
CCGGTGACAAACCGGAGGAAGGTGGGGATGACGTCAAGTCATCATGGCCCTTACGGCCTGGGCTACACACGTGCTACAATGGTCGGTACAAAGGGTTGCC
AAGCCGCGAGGTGGAGCTAATCCCATAAAACCGATCGTAGTCCGGATCGCAGTCTGCAACTCGACTGCGTGAAGTCGGAATCGCTAGTAATCGCGAATCA
GAATGTCGCGGTGAATACGTTCCCGGGCCTTGTACACACCGCCCGTCACACCATGGGAGTGGGTTGCACCAGAAGTAGCTAGTCTAACCTTCGGGAGGAC

>EB309
TCGAGCGGATGAGAAGAGCTTGCTCTTCGATTCAGCGGCGGACGGGTGAGTAATGCCTAGGAATCTGCCTGGTAGTGGGGGACAACGTTTCGAAAGGAAC
GCTAATACCGCATACGTCCTACGGGAGAAAGCAGGGGACCTTCGGGCCTTGCGCTATCAGATGAGCCTAGGTCGGATTAGCTAGTTGGTGAGGTAATGGC
TCACCAAGGCGACGATCCGTAACTGGTCTGAGAGGATGATCAGTCACACTGGAACTGAGACACGGTCCAGACTCCTACGGGAGGCAGCAGTGGGGAATAT
TGGACAATGGGCGAAAGCCTGATCCAGCCATGCCGCGTGTGTGAAGAAGGTCTTCGGATTGTAAAGCACTTTAAGTTGGGAGGAAGGGCTGTTTCCTAAT
ACGAAGCAGTTTTGACGTTACCGACAGAATAAGCACCGGCTAACTCTGTGCCAGCAGCCGCGGTAATACAGAGGGTGCAAGCGTTAATCGGAATTACTGG
GCGTAAAGCGCGCGTAGGTGGTTTGTTAAGTTGGATGTGAAATCCCCGGGCTCAACCTGGGAACTGCATCCAAAACTGGCAAGCTAGAGTAGGGCAGAGG
GTGGTGGAATTTCCTGTGTAGCGGTGAAATGCGTAGATATAGGAAGGAACACCAGTGGCGAAGGCGACCACCTGGGCTCATACTGACACTGAGGTGCGAA
AGCGTGGGGAGCAAACAGGATTAGATACCCTGGTAGTCCACGCCGTAAACGATGTCAACTAGCCGTTGGAATCCTTGAGATTTTAGTGGCGCAGCTAACG
CATTAAGTTGACCGCCTGGGGAGTACGGCCGCAAGGTTAAAACTCAAATGAATTGACGGGGGCCCGCACAAGCGGTGGAGCATGTGGTTTAATTCGAAGC
AACGCGAAGAACCTTACCAGGCCTTGACATCCAGTGAACTTACCAGAGATGGTTTGGTGCCTTCGGGAACACTGAGACAGGTGCTGCATGGCTGTCGTCA
GCTCGTGTCGTGAGATGTTGGGTTAAGTCCCGTAACGAGCGCAACCCTTGTCCTTAGTTACCAGCACGTTATGGTGGGCACTCTAAGGAGACTGCCGGTG
ACAAACCGGAGGAAGGTGGGGATGACGTCAAGTCATCATGGCCCTTACGGCCTGGGCTACACACGTGCTACAATGGTCGGTACAGAGGGTTGCCAAGCCG
CGAGGTGGAGCTAATCTCACAAAACCGATCGTAGTCCGGATCGCAGTCTGCAACTCGACTGCGTGAAGTCGGAATCGCTAGTAATCGCGAATCAGAATGT
CGCGGTGAATACGTTCCCGGGCCTTGTACACACCGCCCGTCACACCATGGGAGTGGGTTGCACCAGAAGTAGCTAGTCTAACCTTCGGGAGG

>EB310
GATGACGCTGGCGGCGTGCCTAATACATGCAAGTCGAGCGAATGGATTAAGAGCTTGCTCTTATGAAGTTAGCGGCGGACGGGTGAGTAACACGTGGGTA
ACCTGCCCATAAGACTGGGATAACTCCGGGAAACCGGGGCTAATACCGGATAACATTTTGAACCGCATGGTTCGAAATTGAAAGGCGGCTTCGGCTGTCA
CTTATGGATGGACCCGCGTCGCATTAGCTAGTTGGTGAGGTAACGGCTCACCAAGGCAACGATGCGTAGCCGACCTGAGAGGGTGATCGGCCACACTGGG
ACTGAGACACGGCCCAGACTCCTACGGGAGGCAGCAGTAGGGAATCTTCCGCAATGGACGAAAGTCTGACGGAGCAACGCCGCGTGAGTGATGAAGGCTT
TCGGGTCGTAAAACTCTGTTGTTAGGGAAGAACAAGTGCTAGTTGAATAAGCTGGCACCTTGACGGTACCTAACCAGAAAGCCACGGCTAACTACGTGCC
AGCAGCCGCGGTAATACGTAGGTGGCAAGCGTTATCCGGAATTATTGGGCGTAAAGCGCGCGCAGGTGGTTTCTTAAGTCTGATGTGAAAGCCCACGGCT
CAACCGTGGAGGGTCATTGGAAACTGGGAGACTTGAGTGCAGAAGAGGAAAGTGGAATTCCATGTGTAGCGGTGAAATGCGTAGAGATATGGAGGAACAC
CAGTGGCGAAGGCGACTTTCTGGTCTGTAACTGACACTGAGGCGCGAAAGCGTGGGGAGCAAACAGGATTAGATACCCTGGTAGTCCACGCCGTAAACGA
TGAGTGCTAAGTGTTAGAGGGTTTCCGCCCTTTAGTGCTGAAGTTAACGCATTAAGCACTCCGCCTGGGGAGTACGGCCGCAAGGCTGAAACTCAAAGGA
ATTGACGGGGGCCCGCACAAGCGGTGGAGCATGTGGTTTAATTCGAAGCACGCGAAGAACCTTACCAGTCGTGACATCCTCTGACACCCTAGAGATAGGG
CTTCTCCTTCGGGAGCAGAGTGA

>EB311
ACCGTCCCCCCGAAGGTTAGACTAGCTACTTCTGGTGCAACCCACTCCCATGGTGTGACGGGCGGTGTGTACAAGGCCCGGGAACGTATTCACCGCGACA
TTCTGATTCGCGATTACTAGCGATTCCGACTTCACGCAGTCGAGTTGCAGACTGCGATCCGGACTACGATCGGTTTTGTGGGATTAGCTCCACCTCGCGG
CTTGGCAACCCTCTGTACCGACCATTGTAGCACGTGTGTAGCCCAGGCCGTAAGGGCCATGATGACTTGACGTCATCCCCACCTTCCTCCGGTTTGTCAC
CGGCAGTCTCCTTAGAGTGCCCACCATAACGTGCTGGTAACTAAGGACAAGGGTTGCGCTCGTTACGGGACTTAACCCAACATCTCACGACACGAGCTGA
CGACAGCCATGCAGCACCTGTCTCAATGTTCCCGAAGGCACCAATCCATCTCTGGAAAGTTCATTGGATGTCAAGGCCTGGTAAGGTTCTTCGCGTTGCT
TCGAATTAAACCACATGCTCCACCGCTTGTGCGGGCCCCCGTCAATTCATTTGAGTTTTAACCTTGCGGCCGTACTCCCCAGGCGGTCAACTTAATGCGT
TAGCTGCGCCACTAAGAGCTCAAGGCTCCCAACGGCTAGTTGACATCGTTTACGGCGTGGACTACCAGGGTATCTAATCCTGTTTGCTCCCCACGCTTTC
GCACCTCAGTGTCAGTATCAGTCCAGGTGGTCGCCTTCGCCACTGGTGTTCCTTCCTATATCTACGCATTTCACCGCTACACAGGAAATTCCACCACCCT
CTACCATACTCTAGCTTGTCAGTTTTGAATGCAGTTCCCAGGTTGAGCCCGGGGCTTTCACATCCAACTTAACAAACCACCTACGCGCGCTTTACGCCCA
GTAATTCCGATTAACGCTTGCACCCTCTGTATTACCGCGGCTGCTGGCACAGAGTTAGCCGGTGCTTATTCTGTCGGTAACGTCAAAATTGCAGAGTATT
AATCTACAACCCTTCCTCCCAACTTAAAGTGCTTTACAATCCGAAGACCTTCTTCACACACGCGGCATGGCTGGATCAGGCTTTCGCCCATTGTCCAATA
TTCCCCACTGCTGCCTCCCGTAGGAGTCTGGACCGTGTCTCAGTTCCAGTGTGACTGATCATCCTCTCAGACCAGTTACGGATCGTCGCCTTGGTGAGCC
ATTACCTCACCAACTAGCTAATCCGACCTAGGCTCATCTGATAGCGCAAGGCCCGAAGGTCCCCTGCTTTCTCCCGTAGGACGTATGCGGTATTAGCGTT
CCTTTCGAAACGTTGTCCCCCACTACCAGGCAGATTCCTAGGCATTACTCACCCGTCCGCCGCTGAATCAGAGAGCAAGCTCTCTTCATCCGCTCGACTT
GCATG

>EB312
GGCAGCACAGGAGAGCTTGCTCTCTGGGTGGCGAGTGGCGGACGGGTGAGGAATACATCGGAATCTACCTTTTCGTGGGGGATAACGTAGGGAAACTTAC
GCTAATACCGCATACGACCTACGGGTGAAAGTGGGGGACCGCAAGGCCTCACGCGATTAGATGAGCCGATGTCCGATTAGCTAGTTGGCGGGGTAATGGC
CCACCAAGGCGACGATCGGTAGCTGGTCTGAGAGGATGATCAGCCACACTGGAACTGAGACACGGTCCAGACTCCTACGGGAGGCAGCAGTGGGGAATAT
TGGACAATGGGCGCAAGCCTGATCCAGCCATACCGCGTGGGTGAAGAAGGCCTTCGGGTTGTAAAGCCCTTTTGTTGGGAAAGAAATCCTATCGGTTAAT
AACCGGTGGGGATGACGGTACCCAAAGAATAAGCACCGGCTAACTTCGTGCCAGCAGCCGCGGTAATACGAAGGGTGCAAGCGTTACTCGGAATTACTGG
GCGTAAAGCGTGCGTAGGTGGTGGTTTAAGTCTGCTGTGAAAGCCCTGGGCTCAACCTGGGAATTGCAGTGGATACTGGATCACTAGAGTGTGGTAGAGG
GATGCGGAATTTCTGGTGTAGCAGTGAAATGCGTAGAGATCAGAAGGAACATCCGTGGCGAAGGCGGCATCCTGGGCCAACACTGACACTGAGGCACGAA
AGCGTGGGGAGCAAACAGGATTAGATACCCTGGTAGTCCACGCCCTAAACGATGCGAACTGGATGTTGGGTGCAACTTGGCACCCAGTATCGAAGCTAAC
GCGTTAAGTTCGCCGCCTGGGGAGTACGGTCGCAAGACTGAAACTCAAAGGAATTGACGGGGGCCCGCACAAGCGGTGGAGTATGTGGTTTAATTCGATG
CAACGCGAAGAACCTTACCTGGTCTTGACATCCACGGAACTTTCCAGAGATGGATTGGTGCCTTCGGGAACCGTGAGACAGGTGCTGCATGGCTGTCGTC
AGCTCGTGTCGTGAGATGTTGGGTTAAGTCCCGCAACGAGCGCAACCCTTGTCCTTAGTTGCCAGCACGTAATGGTGGGAACTCTAAGGAGACCGCCGGT
GACAAACCGGAGGAAGGTGGGGATGACGTCAAGTCATCATGGCCCTTACGACCAGGGCTACACACGTACTACAATGGTAGGGACAGAGGGCTGCAAACCC
GCGAGGGTGAGCCAATCCCAGAAACCCTATCTCAGTCCGGATTGGAGTCTGCAACTCGACTCCATGAAGTCGGAATCGCTAGTAATCGCAGATCAGCATT
GCTGCGGTGAATACGTTCCCGGGCCTTGTACACACCGCCCGTCACACCATGGGAGTTTGTTGCACCAGAAGCAGGTAGCTTAACCTTCGGGAGGGCGCT

>EB313
CGAGCGAATGACGAAGAAGCTTGCTTCTTCTGATTTAGCGGCGGACGGGTGAGTAACACGTGGGCAACCTGCCCTGTAGATTGGGATAACTCCGGGAAAC
CGGGGCTAATACCGAATAATCCATTTCCTCACATGGGGAGATGTTAAAAGACGGTTTCGGCTGTCACTACAGGATGGGCCCGCGGCGCATTAGCTAGTTG
GTGAGGTAACGGCTCACCAAGGCGACGATGCGTAGCCGACCTGAGAGGGTGATCGGCCACACTGGGACTGAGACACGGCCCAGACTCCTACGGGAGGCAG
CAGTAGGGAATCTTCCACAATGGACGAAAGTCTGATGGAGCAATGCCGCGTGAGTGAAGAAGGTTTTCGGATCGTAAAACTCTGTTGTGAGGGAAGAACA
AGTACGAGAGTAACTGCTCGTACCTTGACGGTACCTCATTAGAAAGCCACGGCTAACTACGTGCCAGCAGCCGCGGTAATACGTAGGTGGCAAGCGTTGT
CCGGAATTATTGGGCGTAAAGCGCGCGCAGGCGGTCCTTTAAGTCTGATGTGAAATCCCACGGCTCAACCGTGGAAGGTCATTGGAAACTGGGGGACTTG
AGTACAGAAGAGGAAAGCGGAATTCCAAGTGTAGCGGTGAAATGCGTAGAGATTTGGAGGAACACCAGTGGCGAAGGCGGCTTTCTGGTCTGTAACTGAC
GCTGAGGCGCGAAAGCGTGGGGAGCAAACAGGATTAGATACCCTGGTAGTCCACGCCGTAAACGATGAGTGCTAAGTGTTAGGGGGTTTCCGCCCCTTAG
TGCTGCAGCTAACGCATTAAGCACTCCGCCTGGGGAGTACGGTCGCAAGACTGAAACTCAAAGGAATTGACGGGGGCCCGCACAAGCGGTGGAGCATGT
GGTTTAATTCGAAGCAACGCGAAGAACCTTACCAGGTCTTGGACATCCCGCTGACCGCTCTAGAGATAGAGTTTTTCCCTTCGGGGACAGCGGTGACAGG
TGGTGCATGGTTGTCGTCAGCTCGTGTCGTGAGATGTTGGGTTAAGTCCCGCAACGAGCGCAACCCTTGATCTTAGTTGCCAGCATTCAGTTGGGCACTC
TAAGGTGACTGCCGGTGATAAACCGGAGGAAGGTGGGGATGACGTCAAATCATCATGCCCCTTATGACCTGGGCTACACACGTGCTACAATGGACGGTAC
AGAGGGTCGCAACCCCGCGAGGGTGAGCAAATCCCATAAAACCGTTCTCAGTTCGGATTGTAGGCTGCAACTCGCCTACATGAAGCCGGAATCGCTAGTA
ATCGTGGATCAGCATGCCACGGTGAATACGTTCCCGGGCCTTGTACACACCGCCCGTCACACCACGAGAGTTTGTAACACCCGAAGTCGGTGGGGTAACC
CTTACGGGAGCCAGCCG

>EB314
CTGGCTCCCGTAAGGGTTACCCCACCGACTTCGGGTGTTACAAACTCTCGTGGTGTGACGGGCGGTGTGTACAAGGCCCGGGAACGTATTCACCGTGGCA
TGCTGATCCACGATTACTAGCGATTCCGGCTTCATGTAGGCGAGTTGCAGCCTACAATCCGAACTGAGAACGGTTTTATGGGATTAGCTCACCCTCGCGG
GGTTGCGACCCTCTGTACCGTCCATTGTAGCACGTGTGTAGCCCAGGTCATAAGGGGCATGATGATTTGACGTCATCCCCACCTTCCTCCGGTTTATCAC
CGGCAGTCACCTTAGAGTGCCCAACTGAATGCTGGCAACTAAGATCAAGGGTTGCGCTCGTTGCGGGACTTAACCCAACATCTCACGACACGAGCTGACG
ACAACCATGCACCACCTGTCACCGCTGTCCCCGAAGGGAAAAAATTCTATCTCTAGAACGGTCAGCGGGATGTCAAGACCTGGTAAGGTTCTTCGCGTTG
CTTCGAATTAAACCACATGCTCCACCGCTTGTGCGGGCCCCCGTCAATTCCTTTGAGTTTCAGTCTTGCGACCGTACTCCCCAGGCGGAGTGCTTAATGC
GTTAGCTGCAGCACTAAGGGGCGGAAACCCCCTAACACTTAGCACTCATCGTTTACGGCGTGGACTACCAGGGTATCTAATCCTGTTTGCTCCCCACGCT
TTCGCGCCTCAGCGTCAGTTACAGACCAGAAAGTCGCCTTCGCCACTGGTGTTCCTCCAAATCTCTACGCATTTCACCGCTACACTTGGAATTCCACTTT
CCTCTTCTGCACTCAAGTCCCCCAGTTTCCAATGACCTTCCACGGTTGAGCCGTGGGCTTTCACATCAGACTTAAAGGACCGCCTGCGCGCGCTTTACGC
CCAATAATTCCGGACAACGCTTGCCACCTACGTATTACCGCGGCTGCTGGCACGTAGTTAGCCGTGGCTTTCTAATGAGGTACCGTCAAGGTACGAGCAG
TTACTCTCGTACTTGTTCTTCCCTCACAACAGAGTTTTACGATCCGAAAACCTTCTTCACTCACGCGGCGTTGCTCCATCAGACTTTCGTCCATTGTGGA
AGATTCCCTACTGCTGCCTCCCGTAGGAGTCTGGGCCGTGTCTCAGTCCCAGTGTGGCCGATCACCCTCTCAGGTCGGCTACGCATCGTCGCCTTGGTGA
GCCGTTACCTCACCAACTAGCTAATGCGCCGCGGGCCCATCCTGTAGTGACAGCCGAGACCGTCTTTTAACATTCCCACATGTGAGGGAATGGATTATTC

GGTATTAGCCCCGGTTTCCCGGAGTTATCCCAATCTACAGGGCAGGTTGCCCACGTGTTACTCACCCGTCCGCCGCTAAATCAGAAGAAGCAAGCTTCTT
CGTCATTCGCTCGACTTGCA

>EB315
TGCAAGTCGAGCGGTAGCACAGGAGAGCTTGCTCTCTGGGTGACGAGCGGCGGACGGGTGAGTAATGTCTGGGAAACTGCCTGATGGAGGGGGATAACTA
CTGGAAACGGTAGCTAATACCGCATGACCTCGCAAGAGCAAAGTGGGGGACCTTCGGGCCTCACGCCATCGGATGTGCCCAGATGGGATTAGCTAGTAGG
TGGGGTAATGGCTCACCTAGGCGACGATCCCTAGCTGGTCTGAGAGGATGACCAGCCACACTGGAACTGAGACACGGTCCAGACTCCTACGGGAGGCAGC
AGTGGGGAATATTGCACAATGGGCGCAAGCCTGATGCAGCCATGCCGCGTGTGTGAAGAAGGCCTTAGGGTTGTAAAGCACTTTCAGCGAGGAGGAAGGG
TTCAGTGTTAATAGCACTGTACATTGACGTTACTCGCAGAAGAAGCACCGGCTAACTCCGTGCCAGCAGCCGCGGTAATACGGAGGGTGCAAGCGTTAAT
CGGAATTACTGGGCGTAAAGCGCACGCAGGCGGTTTGTTAAGTCAGATGTGAAATCCCCGAGCTTAACTTGGGAACTGCATTTGAAACTGGCAAGCTAGA
GTCTTGTAGAGGGGGGTAGAATTCCAGGTGTAGCGGTGAAATGCGTAGAGATCTGGAGGAATACCGGTGGCGAAGGCGGCCCCCTGGACAAAGACTGACG
CTCAGGTGCGAAAGCGTGGGGAGCAAACAGGATTAGATACCCTGGTAGTCCACGCTGTAAACGATGTCGACTTGGAGGTTGTGCCCTTGAGGCGTGGCTT
CCGGAGCTAACGCGTTAAGTCGACCGCCTGGGGAGTACGGCCGCAAGGTTAAAACTCAAATGAATTGACGGGGGCCCGCACAAGCGGTGGAGCATGTGGT
TTAATTCGATGCAACGCGAAGAACCTTACCTACTCTTGACATCCAGAGAATTCGCTAGAGATAGCTTAGTGCCTTCGGGAACTCTGAGACAGGTGCTGCA
TGGCTGTCGTCAGCTCGTGTTGTGAAATGTTGGGTTAAGTCCCGCAACGAGCGCAACCCTTATCCTTTGTTGCCAGCGAGTAATGTCGGGAACTCAAAGG
AGACTGCCGGTGATAAACCGGAGGAAGGTGGGGATGACGTCAAGTCATCATGGCCCTTACGAGTAGGGCTACACACGTGCTACAATGGCATATACAAAGA
GAAGCAAACTCGCGAGAGCAAGCGGACCTCATAAAGTATGTCGTAGTCCGGATTGGAGTCTGCAACTCGACTCCATGAAGTCGGAATCGCTAGTAATCGT
AGATCAGAATGCTACGGTGAATACGTTCCCGGGCCTTGTACACACCGCCCGTCACACCATGGGAGTGGGTTGCAAAAGAAGTAGGTAGCTTAACCTTC

>EB316
GGTAACGACGTTCATGACTTGAGGGGCGGTGTGTACAAGACCCGGGAACGTATTCACCGCAGCGTTGCTGATCTGCGATTACTAGCGACTCCGACTTCAT
GAGGTCGAGTTGCAGACCTCAATCCGAACTGGGACCGGCTTTTTGGGATTCGCTCCACCTCGCGGTATTGCAGCCCTTTGTACCGGCCATTGTAGCATGC
GTGAAGCCCAAGACATAAGGGGCATGATGATTTGACGTCATCCCCACCTTCCTCCGAGTTGACCCCGGCAGTATCCCATGAGTTCCCACCATTACGTGCT
GGCAACATAGAACGAGGGTTGCGCTCGTTGCGGGACTTAACCCAACATCTCACGACACGAGCTGACGACAACCATGCACCACCTGTTTACGAGTGTCCAA
AGAGTTGACCATTTCTGGCCCGTTCTCGTATATGTCAAGCCTTGGTAAGGTTCTTCGCGTTGCATCGAATTAATCCGCATGCTCCGCCGCTTGTGCGGGT
CCCCGTCAATTCCTTTGAGTTTTAGCCTTGCGGCCGTACTCCCCAGGCGGGGAACTTAATGCGTTAGCTGCGTCACGGAATCCGTGGAATGGACCCCACA
ACTAGTTCCCAACGTTTACGGGGTGGACTACCAGGGTATCTAAGCCTGTTTGCTCCCCACCCTTTCGCTCCTCAGCGTCAGTAACGGCCCAGAGATCTGC
CTTCGCCATCGGTGTTCCTCCTGATATCTGCGCATTCCACCGCTACACCAGGAATTCCAATCTCCCCTACCGCACTCTAGTCTGCCCGTACCCACTGCAG
ACCCGAGGTTGAGCCTCGGGATTTCACAGCAGACGCGACAAACCGCCTACGAGCTCTTTACGCCCAATAATTCCGGATAACGCTTGCGCCCTACGTATTA
CCGCGGCTGCTGGCACGTAGTTAGCCGGCGCTTTTTCTGCAGGTACCGTCACTTTCGCTTCTTCCCTGCTAAAAGAGGTTTACAACCCGAAGGCCGTCAT
CCCTCACGCGGCGTTGCTGCATCAGGCTTTCGCCCATTGTGCAATATTCCCCACTGCTGCCTCCCGTAGGAGTCTGGGCCGTGTCTCAGTCCCAGTGTGG
CCGGTCACCCTCTCAGGCCGGCTACCCGTCGACGCCTTGGTGAGCCATTACCTCACCAACAAGCTGATAGGCCGTGAGCCCATCCCAGACCGAAAAATCT
TTCCAACTGCTGACCATGCGATCGCAGCTCATATCCAGTATTAGACGCCGTTTCCAGCGCTTATCCCAGAGTCAGGGGCAGGTTGCTCACGTGTTACTCA
CCCGTTCGCCAA

>EB317
TTAAGCTACCTACTTCTTTTGCAACCCACTCCCATGGTGTGACGGGCGGTGTGTACAAGGCCCGGGAACGTATTCACCGTAGCATTCTGATCTACGATTA
CTAGCGATTCCGACTTCATGGAGTCGAGTTGCAGACTCCAATCCGGACTACGACATACTTTATGAGGTCCGCTTGCTCTCGCGAGTTCGCTTCTCTTTGT
ATATGCCATTGTAGCACGTGTGTAGCCCTACTCGTAAGGGCCATGATGACTTGACGTCATCCCCACCTTCCTCCGGTTTATCACCGGCAGTCTCCTTTGA
GTTCCCGACATGACTCGCTGGCAACAAAGGATAAGGGTTGCGCTCGTTGCGGGACTTAACCCAACATTTCACAACACGAGCTGACGACAGCCATGCAGCA
CCTGTCTCAGAGTTCCCGAAGGCACTAAGCTATCTCTAGCGAATTCTCTGGATGTCAAGAGTAGGTAAGGTTCTTCGCGTTGCATCGAATTAAACCACAT
GCTCCACCGCTTGTGCGGGCCCCCGTCAATTCATTTGAGTTTTAACCTTGCGGCCGTACTCCCCAGGCGGTCGATTTAACGCGTTAGCTCCGGAAGCCAC
GGGTCAAGCCCACAACCTCCAAATCGACATCGTTTACAGCGTGGACTACCAGGGTATCTAATCCTGTTTGCTCCCCACGCTTTCGCACCTGAGCGTCAGT
CTTTGTCCAGGGGGCCGCCTTCGCCACCGGTATTCCTCCAGATCTCTACGCATTTCACCGCTACACCTGGAATTCTACCCCCCTCTACAAGACTCTAGCT
TGCCAGTTTCAAATGCAGTTCCCAAGTTAAGCTCGGGGATTTCACATCTGACTTAACAAACCGCCTGCGTGCGCTTTACGCCCAGTAATTCCGATTAACG
CTTGCACCCTCCGTATTACCGCGGCTGCTGGCACGGAGTTAGCCGGTGCTTCTTCTGCGAGTAACGTCAATGCACAGTGCTATTAACACTGAACCCTTCC
TCCTCGCTGAAAGTGCTTTACAACCCGAAGGCCTTCTTCACACACGCGGCATGGCTGCATCAGGCTTGCGCCCATTGTGCAATATTCCCCACTGCTGCCT
CCCGTAGGAGTCTGGACCGTGTCTCAGTTCCAGTGTGGCTGGTCATCCTCTCAGACCAGCTAGGGATCGTCGCCTAGGTGAGCCATTACCTCACCTACTA
GCTAATCCCATCTGGGCACATCCGATGGCGTGAGGCCCGAAGGTCCCCCACTTTGCTCTTTCGAGGTCATGCGGTATTAGCTACCGTTTCCAGTAGTTAT
CCCCCTCCATCAGGCAGTTTCCCAGACATTACTCACCCGTCCGCCGCTCGCCGGCAAAGTAGCAAGCTACTTCCCGCTGCCGCTCGACTTGCA

>EB318
ACACATGCAAGTCGAGCGGCAGCGGGAAGTAGCTTGCTACTTTGCCGGCGAGCGGCGGACGGGTGAGTAATGTCTGGGAAACTGCCTGATGGAGGGGGAT
AACTACTGGAAACGGTAGCTAATACCGCATGACCTCGCAAGAGCAAAGTGGGGGACCTTCGGGCCTCACGCCATCGGATGTGCCCAGATGGGATTAGCTA
GTAGGTGAGGTAATGGCTCACCTAGGCGACGATCCCTAGCTGGTCTGAGAGGATGACCAGCCACACTGGAACTGAGACACGGTCCAGACTCCTACGGGAG
GCAGCAGTGGGGAATATTGCACAATGGGCGCAAGCCTGATGCAGCCATGCCGCGTGTGTGAAGAAGGCCCTTTAGGGTTGTAAAGCACTTTCAGGCGAGG
AGGAAGGGTTCAGTGTTAATAGCACTGTTCATTGACGTTACTCGCAGAAGAAGCACCGGCTAACTCCGTGCCAGCAGCCGCGGTAATACGGAGGGTGCAA
GCGTTAATCGGAATTACTGGGCGTAAAGCGCACGCAGGCGGTTTGTTAAGTCAGATGTGAAATCCCCCGAGCTTAACTTGGGAACTGCATTTGAAACTGG
CAAGCTAGAGTCTTGTAGAGGGGGGTAGAATTCCAGGTGTAGCGGTGAAATGCGTAGAGATCTGGAGGAATACCGGTGGCGAAGGCGGCCCCCTGGACAA
AGACTGACGCTCAGGTGCGAAAGCGTGGGGAGCAAACAGGATTAGATACCCTGGTAGTCCACGCTGTAAACGATGTCGACTTGGAGGTTGTGCCCTTGAG
GCGTGGCTTCCGGAGCTAACGCGTTAAGTCGACCGCCTGGGGAGTACGGCCGCAAGGTTAAAACTCAAATGAATTGACGGGGGCCCGCACAAGCGGTGGA
GCATGTGGTTTAATTCGATGCCAACGCGAAGAACCTTACCTACTCTTGACATCCAGAGAATTCGCTAGAGATAGCTTAGTGCCTTCGGGAACTCTGAGAC
AGGTGCTGCATGGCTGTCGTCAGCTCGTGTTGTGAAATGTTGGGTTAAGTCCCGCAACGAGCGCAACCCTTATCCTTTGTTGCCAGCACGTAATGGTGGG
AACTCAAAGGAGACTGCCGGTGACAAACCGGAGGAAGGTGGGGATGACGTCAAGTCATCATGGCCCTTACGAGTAGGGCTACACACGTGCTACAATGGCA
TATACAAAGAGAAGCAAACTCGCGAGAGCAAGCGGACCTCATAAAGTATGTCGTAGTCCGGATTGGAGTCTGCAACTCGACTCCATGAAGTCGGAATCGC
TAGTAATCGTAGATCAGAATGCTACGGTGAATACGTTCCCGGGCCTTGTACACACCGCCCGTCACACCATGGGAGTGGGTTGCAAAAGAAGTAGGTAGCT
TAACCTTCGGGAGGGCGCTACCAC

>EB319
AGCGCCCTCCCGAAGGTTAAGCTACCTACTTCTTTTGCAACCCACTCCCATGGTGTGACGGGCGGTGTGTACAAGGCCCGGGAACGTATTCACCGTAGCA
TTCTGATCTACGATTACTAGCGATTCCGACTTCATGGAGTCGAGTTGCAGACTCCAATCCGGACTACGACATACTTTATGAGGTCCGCTTGCTCTCGCGA
GGTCGCTTCTCTTTGTATATGCCATTGTAGCACGTGTGTAGCCCTACTCGTAAGGGCCATGATGACTTGACGTCATCCCCACCTTCCTCCAGTTTATCAC
TGGCAGTCTCCTTTGAGTTCCCGACCGAATCGCTGGCAACAAAGGATAAGGGTTGCGCTCGTTGCGGGACTTAACCCAACATTTCACAACACGAGCTGAC
GACAGCCATGCAGCACCTGTCTCAGAGTTCCCGAAGGCACCAAAGCATCTCTGCTAAGTTCTCTGGATGTCAAGAGTAGGTAAGGTTCTTCGCGTTGCAT
CGAATTAAACCACATGCTCCACCGCTTGTGCGGGCCCCCGTCAATTCATTTGAGTTTTAACCTTGCGGCCGTACTCCCCAGGCGGTCGACTTAACGCGTT
AGCTCCGGAAGCCACTCCTCAAGGGAACAACCTCCAAGTCGACATCGTTTACAGCGTGGACTACCAGGGTATCTAATCCTGTTTGCTCCCCACGCTTTCG
CACCTGAGCGTCAGTCTTTGTCCAGGGGGCCGCCTTCGCCACCGGTATTCCTCCAGATCTCTACGCATTTCACCGCTACACCTGGAATTCTACCCCCCTC
TACAAGACTCAAGCCTGCCAGTTTCAAATGCAGTTCCCAGGTTGAGCCCGGGGATTTCACATCTGACTTAACAGACCGCCTGCGTGCGCTTTACGCCCAG

TAATTCCGATTAACGCTTGCACCCTCCGTATTACCGCGGCTGCTGGCACGGAGTTAGCCGGTGCTTCTTCTGCGAGTAACGTCAATCGCTAAGGTTATTA
ACCTTAACGCCTTCCTCCTCGCTGAAAGTACTTTACAACCCGAAGGCCTTCTTCATACACGCGGCATGGCTGCATCAGGCTTGCGCCCATTGTGCAATAT
TCCCCACTGCTGCCTCCCGTAGGAGTCTGGACCGTGTCTCAGTTCCAGTGTGGCTGGTCATCCTCTCAGACCAGCTAGGGATCGTCGCCTAGGTGAGCCA
TTACCCCACCTACTAGCTAATCCCATCTGGGCACATCTGATGGCATGAGGCCCGAAGGTCCCCCACTTTGGTCTTGCGACGTTATGCGGTATTAGCTACC
GTTTCCAGTAGTTATCCCCCTCCATCAGGCAGTTTCCCAGACATTACTCACCCGTCCGCCGCTCGTCACCCGAGAGCAAGCTCTCTGTGCTACCGCTCGA
CTTGCA

>EB320
CGCCCTCCCGAAGGTTAAGCTACCTACTTCTTTTGCAACCCACTCCCATGGTGTGACGGGCGGTGTGTACAAGGCCCGGGAACGTATTCACCGTAGCATT
CTGATCTACGATTACTAGCGATTCCGACTTCATGGAGTCGAGTTGCAGACTCCAATCCGGACTACGACATACTTTATGAGGTCCGCTTGCTCTCGCGAGG
TCGCTTCTCTTTGTATATGCCATTGTAGCACGTGTGTAGCCCTACTCGTAAGGGCCATGATGACTTGACGTCATCCCCACCTTCCTCCAGTTTATCACTG
GCAGTCTCCTTTGAGTTCCCGACCGAATCGCTGGCAACAAAGGATAAGGGTTGCGCTCGTTGCGGGACTTAACCCAACATTTCACAACACGAGCTGACGA
CAGCCATGCAGCACCTGTCTCAGAGTTCCCGAAGGCACCAAAGCATCTCTGCTAAGTTCTCTGGATGTCAAGAGTAGGTAAGGTTCTTCGCGTTGCATCG
AATTAAACCACATGCTCCACCGCTTGTGCGGGCCCCCGTCAATTCATTTGAGTTTTAACCTTGCGGCCGTACTCCCCAGGCGGTCGACTTAACGCGTTAG
CTCCGGAAGCCACTCCTCAAGGGAACAACCTCCAAGTCGACATCGTTTACAGCGTGGACTACCAGGGTATCTAATCCTGTTTGCTCCCCACGCTTTCGCA
CCTGAGCGTCAGTCTTTGTCCAGGGGGCCGCCTTCGCCACCGGTATTCCTCCAGATCTCTACGCATTTCACCGCTACACCTGGAATTCTACCCCCCTCTA
CAAGACTCAAGCCTGCCAGTTTCAAATGCAGTTCCCAGGTTGAGCCCGGGGGATTTCACATCTGACTTAACAGACCGCCTGCGTGCGCTTTACGCCCAGT
AATTCCGATTAACGCTTGCACCCTCCGTATTACCGCGGCTGCTGGCACGGAGTTAGCCGGTGCTTCTTCTGCGAGTAACGTCAATCACTAAGGTTATTAA
CCTTAATGCCTTCCTCCTCGCTGAAAGTACTTTACAACCCGAAGGCCTTCTTCATACACGCGGCATGGCTGCATCAGGCTTGCGCCCATTGTGCAATATT
CCCCACTGCTGCCTCCCGTAGGAGTCTGGACCGTGTCTCAGTTCCAGTGTGGCTGGTCATCCTCTCAGACCAGCTAGGGATCGTCGCCTAGGTGAGCCAT
TACCCCACCTACTAGCTAATCCCATCTGGGCACATCTGATGGCATGAGGCCCGAAGGTCCCCCACTTTGGTCTTGCGACGTTATGCGGTATTAGCTACCG
TTTCCAGTAGTTATCCCCCTCCATCAGGCAGTTTCCCAGACATTACTCACCCGTCCGCCGCTCGTCACCCGAGAGCAAGCTCTCTGTGCTACCGCTCGAC
TGCA

>EB321
GAGTAACACGTGGGTAACCTGCCTGTAAGACTGGGATAACTCCGGGAAACCGGAGCTAATACCGGATAGTTCCTTGAACCGCATGGTTCAAGGATGAAAG
ACGGTTTCGGCTGTCACTTACAGATGGACCCGCGGCGCATTAGCTAGTTGGTGAGGTAACGGCTCACCAAGGCGACGATGCGTAGCCGACCTGAGAGGGT
GATCGGCCACACTGGGACTGAGACACGGCCCAGACTCCTACGGGAGGCAGCAGTAGGGAATCTTCCGCAATGGACGAAAGTCTGACGGAGCAACGCCGCG
TGAGTGATGAAGGTTTTCGGATCGTAAAGCTCTGTTGTTAGGGAAGAACAAGTGCAAGAGTAACTGCTTGCACCTTGACGGTACCTAACCAGAAAGCCAC
GGCTAACTACGTGCCAGCAGCCGCGGTAATACGTAGGTGGCAAGCGTTGTCCGGAATTATTGGGCGTAAAGGGCTCGCAGGCGGTTTCTTAAGTCTGATG
TGAAAGCCCCCGGCTCAACCGGGGAGGGTCATTGGAAACTGGGAAACTTGAGTGCAGAAGAGGAGAGTGGAATTCCACGTGTAGCGGTGAAATGCGTAGA
GATGTGGAGGAACACCAGTGGCGAAGGCGACTCTCTGGTCTGTAACTGACGCTGAGGAGCGAAAGCGTGGGGAGCGAACAGGATTAGATACCCTGGTAGT
CCACGCCGTAAACGATGAGTGCTAAGTGTTAGGGGGTTTCCGCCCCTTAGTGCTGCAGCTAACGCATTAAGCACTCCGCCTGGGGAGTACGGTCGCAAGA
CTGAAACTCAAAGGAATTGACGGGGGCCCGCACAAGCGGTGGAGCATGTGGTTTAATTCGAAGCAACGCGAAGAACCTTACCAGGTCTTGACATCCTCTG
ACAACCCTAGAGATAGGGCTTTCCCTTCGGGGACAGAGTGACAGGTGGTGCATGGTTGTCGTCAGCTCGTGTCGTGAGATGTTGGGTTAAGTCCCGCAAC
GAGCGCAACCCTTGATCTTAGTTGCCAGCATTCAGTTGGGCACTCTAAGGTGACTGCCGGTGACAAACCGGAGGAAGGTGGGGATGACGTCAAATCATCA
TGCCCCTTATGACCTGGGCTACACACGTGCTACAATGGACAGAACAAAGGGCTGCGAGACCGCAAGGTTTAGCCAATCCCACAAATCTGTTCTCAGTTCG
GATCGCAGTCTGCAACTCGACTGCGTGAAGCTGGAATCGCTAGTAATCGCGGATCAGCATGCCGCGGTGAATACGTTCCCGGGCCTTGTACACACCGCCC
GTCACACCACGAGAGTTTGCAACA

>EB322
GGGGGGCTTCGCATGGGGGCGGAGCCTGACGGAGCACGCCGCGTGAGTGATGAAGGCCTTCGGGTCGTAAAACTCTGTTATTAGGGAAGAACAAATGTGT
AAGTAACTATGCACGTCTTGACGGTACCTAATCAGAAAGCCACGGCTAACTACGTGCCAGCAGCCGCGGTAATACGTAGGTGGCAAGCGTTATCCGGAAT
TATTGGGCGTAAAGCGCGCGTAGGCGGTTTCTTAAGTCTGATGTGAAAGCCCACGGCTCAACCGTGGAGGGTCATTGGAAACTGGGAAACTTGAGTGCAG
GAGAGGAAAGTGGAATTCCATGTGTAGCGGTGAAATGCGCAGAGATATGGAGGAACACCAGTGGCGAAGGCGACTTTCTGGTCTGCAACTGACGCTGATG
TGCGAAAGCGTGGGGATCAAACAGGATTAGATACCCTGGTAGTCCACGCCGTAAACGATGAGTGCTAAGTGTTAGGGGGTTTCCGCCCCTTAGTGCTGCA
GCTAACGCATTAAGCACTCCGCCTGGGGAGTACGACCGCAAGGTTGAAACTCAAAGGAATTGACGGGGACCCGCACAAGCGGTGGAGCATGTGGTTTAAT
TCGAAGCAACGCGAAGAACCTTACCAAATCTTGACATCCTTTGCCCCCTCTAGAGATAGAGGTTTCCCCTTCGGGGGACAAAGTGACAGGTGGTGCATGG
TTGTCGTCAGCTCGTGTCGTGAGATGTTGGGTTAAGTCCCGCAACGAGCGCAACCCTTAAGCTTAGTTGCCATCATTCAGTTGGGCACTCTAAGTTGACT
GCCGGTGACAAACCGGAGGAAGGTGGGGATGACGTCAAATCATCATGCCCCTTATGATTTGGGCTACACACGTGCTACAATGGACAATACAAAGGGCAG
CTAAACCGCGAGGTCAAGCAAATCCCATAAAGTTGTTCTCAGTTCGGATTGTAGTCTGCAACTCGACTACATGAAGCTGGAATCGCTAGTAATCGTAGAT
CAGCATGCTACGGTGAATACGTTCCCGGGTCTTGTACACACCGCCCGTCACACCACGAGAGTTTGTAACACCCGAAGCCGGTGGAGCAACCACTTTGTGG
AGCTAGCCCTCCAAAGTGGGACAAATGATTGGGGTGAATCTAAAAAAAGGGGAGAACGCAGATAAGGGGGGGGGCGGGGCTGTGTTGGGC

>EB323
ACGTGGGTAACCTACCTATAAGACTGGAATAACTCCGGGAAACCGGGGCTAATGCCGGATAACATGTTGGACCGCATGGTCCTACAGTGAAAGACGGTCT
TGCTGTCACTTATAGATGGACCCGCGCCGTATTAGCTAGTTGGTAAGGTAACGGCTTACCAAGGCAACGATACGTAGCCGACCTGAGAGGGTGATCGGCC
ACACTGGAACTGAGACACGGTCCAGACTCCTACGGGAGGCAGCAGTAGGGAATCTTCCGCAATGGGCGAAAGCCTGACGGAGCAACGCCGCGTGAGTGAT
GAAGGCCTTCGGGTCGTAAAACTCTGTTATTAGGGAAGAACAAATGTGTAAGTAACTATGCACGTCTTGACGGTACCTAATCAGAAGCCACGGCTAACTA
CGTGCCAGCAGCCGCGGTAATACGTAGGTGGCAAGCGTTATCCGGAATTATTGGGCGTAAAGCGCGCGTAGGCGGTTTCTTAAGTCTGATGTGAAAGCCC
ACGGCTCAACCGTGGAGGGTCATTGGAAACTGGGAAACTTGAGTGCAGGAGAGGAAAGTGGAATTCCATGTGTAGCGGTGAAATGCGCAGAGATATGGAG
GAACACCAGTGGCGAAGGCGACTTTCTGGTCTGCAACTGACGCTGATGTGCGAAAGCGTGGGGATCAAACAGGATTAGATACCCTGGTAGTCCACGCCGT
AAACGATGAGTGCTAAGTGTTAGGGGGTTTCCGCCCCTTAGTGCTGCAGCTAACGCATTAAGCACTCCGCCTGGGGAGTACGACCGCAAGGTTGAAACTC
AAAGGAATTGACGGGGACCCGCACAAGCGGTGGAGCATGTGGTTTAATTCGAAGCAACGCGAAGAACCTTACCAAATCTTGACATCCTTTGCCCCCTCTA
GAGATAGAGGTTTCCCCTTCGGGGGACAAAGTGACAGGTGGTGCATGGTTGTCGTCAGCTCGTGTCGTGAGATGTTGGGTTAAGTCCCGCAACGAGCGCA
ACCCTTAAGCTTAGTTGCCATCATTCAGTTGGGCACTCTAAGTTGACTGCCGGTGACAAACCGGAGGAAGGTGGGGATGACGTCAAATCATCATGCCCCT
TATGATTTGGGCTACACACGTGCTACAATGGACAATACAAAGGGCAGCTAAACCGCGAGGTCAAGCAAATCCCATAAAGTTGTTCTCAGTTCGGATTGTA
GTCTGCAACTCGACTACATGAAGCTGGAATCGCTAGTAATCGTAGATCAGCATGCTACGGTGAATACGTTCCCGGGTCTGGTACACACCGCCCGTCACAC
CACGAGACTTTGTAACCCCTGAAGCCGGTGGAGCATCC

>EB324
GTGGGGAACCTGCCTGTAAGACTGGGATAACTCCGGGAAACCGGAGCTAATACCGGATAGTTCCTTGAACCGCATGGTTCAAGGATGAAAGACGGTTTCG
GCTGTCACTTACAGATGGACCCGCGGCGCATTAGCTAGTTGGTGAGGTAACGGCTCACCAAGGCGACGATGCGTAGCCGACCTGAGAGGGTGATCGGCCA
CACTGGGACTGAGACACGGCCCAGACTCCTACGGGAGGCAGCAGTAGGGAATCTTCCGCAATGGACGAAAGTCTGACGGAGCAACGCCGCGTGAGTGATG
AAGGTTTTCGGATCGTAAAGCTCTGTTGTTAGGGAAGAACAAGTGCAAGAGTAACTGCTTGCACCTTGACGGTACCTAACCAGAAAGCCACGGCTAACTA
CGTGCCAGCAGCCGCGGTAATACGTAGGTGGCAAGCGTTGTCCGGAATTATTGGGCGTAAAGGGCTCGCAGGNCGGTTTCTTAAGTCTGATGTGAAAGCC
CCCGGCTCAACCGGGGAGGGTCATTGGAAACTGGGAAACTTGAGTGCAGAAGAGGAGAGTGGAATTCCACGTGTAGCGGTGAAATGCGTAGAGATGTGGA
GGAACACCAGTGGCGAAGGCGACTCTCTGGTCTGTAACTGACGCTGAGGAGCGAAAGCGTGGGGAGYGAACAGGATTAGATACCCTGGTAGTCCCCNCCG

TAAACGATGAGTGCTAAGTGTTAGGGGGTTYCCGCCCTTTAGTGCTGCAGCTAACGCATTAAGCACTCCGCCTGGGGAGTACGGTCGCAAGACTGAAACT
CAAAGGAATTGACGGGGGCCCGCACAAGCGGTGGAGCATGTGGTTTAATTCGAAGCAACGCGAAGAACCTTACCAGGTCTTGACATCCTCTGACAACCCT
AGAGATAGGGCTTTCCCTTCGGGGACAGAGTGACAGGTGGTGCATGGTTGTCGTCAGCTCGTGTCGTGAGATGTTGGGTTAAGTCCCGCAACGAGCGCAA
CCCTTGATCTTAGTTGCCAGCATTCAGTTGGGCACTCTAAGGTGACTGCCGGTGACAAACCGGAGGAAGGTGGGGATGACGTCAAATCATCATGCCCCTT
ATGACCTGGGCTACACACGTGCTACAATGGACAGAACAAAGGGCTGCGAGACCGCAAGGTTTAGCCAATCCCACAAATCTGTTCTCAGTTCGGATCGCAG
TCTGCAACTCGACTGCGTGAAGCAGGAATCGCTAGTAATCGCGGATCAGCATGCCGCGGTGAATACGTTCCCGGGCCTGGTACACACCGCCCCTCAC

>EB325
GTTACCGACTTTCATGACTTGACGGGCGGTGTGTACAAGGCCCGGGAACGTATTCACCGCAGCGTTGCTGATCTGCGATTACTAGCGACTCCGACTTCAT
GAGGTCGAGTTGCAGACCTCAATCCGAACTGGGACCGACTTTTTGGGATTCGCTCCACCTTGCGGTATTGCAGCCCTTTGTATCGGCCATTGTAGCATGC
GTGAAGCCCAAGACATAAGGGGCATGATGATTTGACGTCGTCCCCACCTNTCCTCCGAGTTGACCCCGGCAGTATCCCATGAGTTCCCACCATTACGTGC
TGGCAACATAGAACGAGGGTTGCGCTCGTTGCGGGACTTAACCCAACATCTCACGACACGAGCTGACGACAACCATGCACCACCTGTATACGAGTGTCCA
AAGAGTTGACCATTTCTGGCCCGTTCTCGTATATGTCAAGCCTTGGTAAGGTTCTTCGCGTTGCATCGAATTAATCCGCATGCTCCGCCGCTTGTGCGGG
CCCCCGTCAATTCCTTTGAGTTTTAGCCTTGCGGCCGTACTCCCCAGGCGGGGAACTTAATGCGTTAGCTGCGACACGGAGACCGTGGAATGGTCCCCAC
ATCTAGTTCCCAACGTTTACGGGGTGGACTACCAGGGTATCTAAGCCTGTTTGCTCCCCACCCTTTCGCTCCTCAGCGTCAGTTACGGCCCAGAGATCTG
CCTTCGCCATCGGTGTTCCTCCTGATATCTGCGCATTCCACCGCTACACCAGGAATTCCAATCTCCCCTACCGCACTCTAGTCTGCCCGTACCCACTGCA
GACCCGAGGTTGAGCCTCGGGATTTCACAGCAGACGCGACAAACCGCCTACGAGCTCTTTACGCCCAATAATTCCGGACAACGCTTGCACCCTACGTATT
ACCGCGGCTGCTGGCACGTAGTTAGCCGGTGCTTTTTCTGCAGGTACCGTCACTTTCGCTTCTTCCCTGCTAAAAGAGGTTTACAACCCGAAGGCCGTCG
TCCCTCACGCGGCGTTGCTGCATCAGGCTTTCGCCCATTGTGCAATATTCCCCACTGCTGCCTCCCGTAGGAGTCTGGGCCGTGTCTCAGTCCCAGTGTG
GCCGGTCACCCTCTCAGGCCGGCTACCCGTCGTCGCCTTGGTGAGCCATTACCTCACCAACAAGCTGATAGGCCGCGAGTCCATCCTTGACCAAAATTCT
TTCCACAACCAGACGATGCCGTCGGTTGTCATATCCGGTATTAGACGCCGTTTCCAGCGCTTATCCCAGAGTCAAGGGCAGGTTACTCACGTGTTACTCA
CCCGTTCGCCAAT

>EB326
AAGTAGCTTGCTACTTTGCCGGCGAGCGGCGGACGGGTGAGTAATGTCTGGGAAACTGCCTGATGGAGGGGGATAACTACTGGAAACGGTAGCTAATACC
GCATGACCTCGAAAGAGCAAAGTGGGGGATCTTCGGACCTCACGCCATCGGATGTGCCCAGATGGGATTAGCTAGTAGGTGAGGTAATGGCTCACCTAGG
CGACGATCCCTAGCTGGTCTGAGAGGATGACCAGCCACACTGGAACTGAGACACGGTCCAGACTCCTACGGGAGGCAGCAGTGGGGAATATTGCACAATG
GGCGCAAGCCTGATGCAGCCATGCCGCGTGTGTGAAGAAGGCCTTAGGGTTGTAAAGCACTTTCAGCGAGGAGGAAGGCATCATACTTAATACGTGTGGT
GATTGACGTTACTCGCAGAAGAAGCACCGGCTAACTCCGTGCCAGCAGCCGCGGTAATACGGAGGGTGCAAGCGTTAATCGGAATTACTGGGCGTAAAGC
GCACGCAGGCGGTTTGTTAAGTCAGATGTGAAATCCCCGCGCTTAACGTGGGAACTGCATTTGAAACTGGCAAGCTAGAGTCTTGTAGAGGGGGGTAGAA
TTCCAGGTGTAGCGGTGAAATGCGTAGAGATCTGGAGGAATACCGGTGGCGAAGGCGGCCCCCTGGACAAAGACTGACGCTCAGGTGCGAAAGCGTGGGG
AGCAAACAGGATTAGATACCCTGGTAGTCCACGCTGTAAACGATGTCGACTTGGAGGTTGTGCCCTTGAGGCGTGGCTTCCGGAGCTAACGCGTTAAGTC
GACCGCCTGGGGAGTACGGCCGCAAGGTTAAAACTCAAATGAATTGACGGGGGCCCGCACAAGCGGTGGAGCATGTGGTTTAATTCGATGCAACGCGAAG
AACCTTACCTACTCTTGACATCCACGGAATTCGCCAGAGATGGCTTAGTGCCTTCGGGAACCGTGAGACAGGTGCTGCATGGCTGTCGTCAGCTCGTGTT
GTGAAATGTTGGGTTAAGTCCCGCAACGAGCGCAACCCTTATCCTTTGTTGCCAGCACGTAATGGTGGGAACTCAAAGGAGACTGCCGGTGATAAACCGG
AGGAAGGTGGGGATGACGTCAAGTCATCATGGCCCTTACGAGTAGGGCTACACACGTGCTACAATGGCATATACAAAGAGAAGCGAACTCGCGAGAGCAA
GCGGACCTCATAAAGTATGTCGTAGTCCGGATTGGAGTCTGCAACTCGACTCCA

>EB327
CGAGGGGTTAGGCCACCGGCTTCGGAGTGTTACCGACTTTCATGACGTGACGGGCGGTGTGTACAAGGCCCGGGAACGTATTCACCGCAGCGTTGCTGAT
CTGCGATTACTAGCGACTCCGACTTCACGGGGTCGAGTTGCAGACCCCGATCCGAACTGAGACCGGCTTTAAGGGATTCGCTCCACCTCGCGGTATCGCA
GCCCTCTGTACCGGCCATTGTAGCATGTGTGAAGCCCTGGACATAAGGGGCATGATGACTTGACGTCGTCCCCACCTTCCTCCGAGTTGACCCCGGCAGT
CTCCTGCGAGTCCCCACCATTACGTGCTGGCAACACAGGACAAGGGTTGCGCTCGTTGCGGGACTTAACCCAACATCTCACGACACGAGCTGACGACAGC
CATGCACCACCTGTACACCGACCACAAGGGAAACCCCATCTCTGGGGCGGTCCCGGTGTATGTCAAACCCAGGTAAGGTTCTTCGCGTTGCATCGAATTA
ATCCACATGCTCCGCCGCTTGTGCGGGCCCCCGTCAATTCCTTTGAGTTTTAGCCTTGCGGCCGTACTCCCCAGGCGGGGCGCTTAATGCGTTAGCTACG
GCACGGATCCCGTGGAAGGAAACCCACACCTAGCGCCCACCGTTTACGGCGTGGACTACCAGGGTATCTAATCCTGTTCGCTACCCACGCTTTCGCTCCT
CAGCGTCAGTTACTGCCCAGAGACCCGCCTTCGCCACCGGTGTTCCTCCTGATATCTGCGCATTTCACCGCTACACCAGGAATTCCAGTCTCCCCTGCAG
TACTCAAGTCTGCCCGTATCGCCTGCAAGCCCGCAGTTGAGCTGCGGGTTTTCACAGACGACGCGACAAACCGCCTACGAGCTCTTTACGCCCAGTAATT
CCGGACAACGCTCGCACCCTACGTATTACCGCGGCTGCTGGCACGTAGTTGGCCGGTGCTTCTTCTGTACCTACCGTCACTTGCGCTTCGTCGGTACTGA
AAGAGGTTTACAACCCGAAGGCCGTCATCCCTCACGCGGCGTCGCTGCATCAGGCTTGCGCCCATTGTGCAATATTCCCCACTGCTGCCTCCCGTAGGAG
TCTGGGCCGTGTCTCAGTCCCAGTGTGGCCGGTCGCCCTCTCAGGCCGGCTACCCGTCGTCGCCTTGGTGGGCCGTTACCCCACCAACAAGCTGATAGGC
CGCGGGCCCATCCTGCACCGGAAAACCTTTCCACCCCGGAACATGCATCCCGAGGTCCTATCCGGTATTAGACCCAGTTTCCCAGGCTTATCCCGAAGTG
CAGGGCAGATCACCCACGTGTTACTCACCCGTTCGCCACTAATCCACCCAGCAAGCTGGGCTTCATCGTTCGACTGC

>EB328
CGGAAAGGCCTCACCTTGCTGGGGTACTCGAGTGGCGAACGGGTGAGTAACACGTGGGTGATCTGCCTTGCACTTCGGGATAAGCTTGGGAAACTGGGTC
TAATACCGGATAGGACAGCTGTTTAGTGTCAGTTGTGGAAAGTTTTTTCGGTGCAAGATGAGCTCGCGGCCTATCAGCTTGTTGGTGGGGTAATGGCCTA
CCAAGGCGTCGACGGGTAGCCGGCCTGAGAGGGTGGACGGCCACATTGGGACTGAGATACGGCCCAGACTCCTACGGGAGGCAGCAGTGGGGAATATTGC
ACAATGGGCGGAAGCCTGATGCAGCGACGCCGCGTGGGGGATGAAGGCCTTCGGGTTGTAAACTCCTTTCGACAGGGACGAAGCTTTTGTGACGGTACCT
GTATAAGAAGCACCGGCTAACTACGTGCCAGCAGCCGCGGTAATACGTAGGGTGCGAGCGTTGTCCGGAATTACTGGGCGTAAAGAGCTCGTAGGTGGTT
TGTCGCGTCGTCTGTGAAATTCCGGGGCTTAACTCCGGGCGTGCAGGCGATACGGGCTGACTTGAGTGCTGTAGGGGAGACTGGAATTCCTGGTGTAGCG
GTGAAATGCGCAGATATCAGGAGGAACACCGATGGCGAAGGCAGGTCTCTGGGCAGTAACTGACGCTGAGGAGCGAAAGCATGGGTAGCGAACAGGATTA
GATACCCTGGTAGTCCATGCCGTAAACGGTGGGCGCTAGGTGTAGGGGGCTTCCACGTCTTCTGTGCCGTAGCTAACGCATTAAGCGCCCCGCCTGGGGA
GTACGGCCGCAAGGCTAAAACTCAAAGGAATTGACGGGGGCCCGCACAAGCGGCGGAGCATGTGGATTAATTCGATGCAACGCGAAGAACCTTACCTGGG
CTTGACATATAGCGGATCGCTGCAGAGATGTAGTTTCCCTTTGTGGTCGTTATACAGGTGGTGCATGGTTGTCGTCAGCTCGTGTCGTGAGATGTTGGGT
TAAGTCCCGCAACGAGCGCAACCCTTGTCTTATGTTGCCAGCATTTGGTTGGGGACTCATGAGAGACTGCCGGGGTCAACTCGGAGGAAGGTGGGGATGA
CGTCAAATCATCATGCCCCTTATGTCCAGGGCTTCACACATGCTACAATGGTCGGTACAACGCGCAGCGACACTGTGAGGTGGAGCGAATCGCTGAAAGC
CGGCCTTAGTTCGGATTGGGGTCTGCAACTCGACCCCATGAAGTCGGAGTCGCTAGTAATCGCAGATCAGCAACGCTGCGGTGAATACGTTCCCGGGCCT
TGTACACACCGCCCGTCACGTCATGAAAGTTGGTAACTACGCACGAAGCTCAGTGGCTCAA

>EB329
TGCAAGTCGAACGGTAACAGGAAGCAGCTTGCTGCTTCGCTGACGAGTGGCGGACGGGTGAGTAATGTCTGGGAAACTGCCTGATGGCGGGGGATAACTA
CTGGAAACGGTAGCTAATACCGCATAACGTCGCAAGACCAAAGAGGGGGACCTTCGGGCCTCTTGCCATCGGATGTGCCCAGATGGGATTAGCTAGTAGG
TGGGGTAACGGCTCACCTAGGCGACGATCCCTAGCTGGTCTGAGAGGATGACCAGCCACACTGGAACTGAGACACGGTCCAGACTCCTACGGGAGGCAGC
AGTGGGGAATATTGCACAATGGGCGCAAGCCTGATGCAGCCATGCCGCGTGTATGAAGAAGGCCTTCGGGTTGTAAAGTACTTTCAGCGGGGAGGAAGGC
GATACGGGTTAATAACCGTGTCGATTGACGTTACCCGCAGAAGAAGCACCGGCTAACTCCGTGCCAGCAGCCGCGGTAATACGGAGGGTGCAAGCGTTAA
TCGGAATTACTGGGGCGTAAAGCGCACGCAGGCGGTCTGTCAAGTCGGATGTGAAATCCCCGGGCTCAACCCGGGAACTGCATTCGAAACTGGCAGGCTA
GAGTCTTGTAGAGGGGGGGTAGAATTCCAGGTGTAGCGGTGAAATGCGTAGAGATCTGGAGGAATACCGGTGGCGAAGGCGGCCCCCTGGACAAAGACTG

ACGCTCAGGTGCGAAAGCGTGGGGAGCAAACAGGATTAGATACCCTGGTAGTCCACGCCGTAAACGATGTCGACTTGGAGGTTGTGCCCTTGAGGCGTGG
CTTCCGGAGCTAACGCGTTAAGTCGACCGCCTGGGGGAGTACGGCCGCAAGGTTAAAACTCAAATGAATTGACGGGGGCCCGCACAAGCGGTGGAGCA
TGTGGTTTAATTCGATGCAACGCGAAGAACCTTACCTGGGTCTTGACATCCACAGAATCCTGCAGAGATGCGGGAGTGCCTTCGGGAACTGTGAGACAGG
TGCTGCATGGCTGTCGTCAGCTCGTGTTGTGAAATGTTGGGTTAAGTCCCGCAACGAGCGCAACCCTTATCCTTTGTTGCCAGCGGTCCGGCCGGGAACT
CAAAGGAGACTGCCAGTGATAAACTGGAGGAAGGTGGGGATGACGTCAAGTCATCATGGCCCTTACGACCAGGGCTACACACGTGCTACAATGGCGCATA
CAAAGAGAAGCGAACTCGCGAGAGCAAGCGGACCTCATAAAGTGCGTCGTAGTCCGGATTGGAGTCTGCAACTCGACTCCATGAAGTCGGAATCGCTAGT
AATCGTGGATCAGAATGCCACGGTGAATACGTTCCCGGGCCTTGTACACACCGCCCGTCACACCATGGGAGTGGGTTGCAAAAGAAGTAGGTAGCTTAAC
CTTCGGGAGGGCGC

>EB330
TTCTGGCTCAGGACGAACGCTGGCGGCGTGCTTAACACATGCAAGTCGAACGGAAAGGGCCTTTGCTTGCAGGGGTGCTCGAGTGGCGAACGGGTGAGTA
ACACGTGGGTGATCTGCCTTGTACTCTGGGATAAGCTTGGGAAACTGGGTCTAATACCGGATAGGAACTGCCTTTAGTGTGGTGGTTGGAAAGTTTTTC
GGTGCAAGATGAGCTCGCGGCCTATCAGCTTGTTGGTGGGGTAATGGCCTACCAAGGCGTCGACGGGTAGCCGGCCTGAGAGGGTGTACGGCCACATTGG
GACTGAGATACGGCCCAGACTCCTACGGGAGGCAGCAGTGGGGAATATTGCACAATGGGCGGAAGCCTGATGCAGCGACGCCGCGTGGGGGATGACGGCC
TTCGGGTTGTAAACTCCTTTCGACAGGGACGAAGCTTTTTGTGACGGTACCTGTATAAGAAGCACCGGCTAACTACGTGCCAGCAGCCGCGGTAATACGT
AGGGTGCGAGCGTTGTCCGGAATTACTGGGCGTAAAGAGCTCGTAGGTGGTTTGTCGCGTCGTCTGTGAAATTCCGGGGCTTAACTCCGGGCGTGCAGGC
GATACGGGCATAACTTGAGTGCTGTAGGGGAGACTGGAATTCCTGGTGTAGCGGTGAAATGCGCAGATATCAGGAGGAACACCGATGGCGAAGGCAGGTC
TCTGGGCAGTTACTGACGCTGAGGAGCGAAAGCATGGGTAGCGAACAGGATTAGATACCCTGGTAGTCCATGCCGTAAACGGTGGGCGCTAGGTGTAGGG
GGCTTCCACGTCTTCTGTGCCGTAGCTAACGCATTAAGCGCCCCGCCTGGGGAGTACGGCCGCAAGGCTAAAACTCAAAGGAATTGACGGGGGCCCGCAC
AAGCGGCGAAGCATGTGGATTAATTCGATGCAACGCGAAGAACCTTACCTGGGCTTGACATACACCAGATCGCTGCAAAGATGTAGTTTCCCTTGTGGTT
GGTGTACAGGTGGTGCATGGTTGTCGTCAGCTCGTGTCGTGAGATGTTGGGTTAAGTCCCGCAACGAGCGCAACCCTTGTCTTATGTTGCCAGCATTTGG
TTGGGGACTCATGAGAGACTGCCGGGGTTAACTCGGAAGAAGGTGGGGATGACGTCAAATCATCATGCCCCTTATGTCCAGGGCTTCACACATGCTACAA
TGGTCGGTACAACGCGCAGCGACACTGTGAGGTGGAGCGAATCGCTGAAAGCCGGTCTTAGTTCGGATTGGGGTCTGCAACTCGACCCCATGAAGTCGGA
ATCGCTAGTAATCGTAGATCAGCAATGCTGCGGTGAATACGTTCCCGGGCCTTGTAACCACCGCCCGTCACGTCATGAAAGTTGGTAACCCCCGAAGCCA
GTGGCTCAAACTTGTTAAGGGAGCTGT

>EB331
GCCCTCCCGAAGGTTAAGCTACCTACTTCTTTTGCAACCCACTCCCATGGTGTGACGGGCGGTGTGTACAAGGCCCGGGAACGTATTCACCGTAGCATTC
TGATCTACGATTACTAGCGATTCCGACTTCACGGAGTCGAGTTGCAGACTCCGATCCGGACTACGACATACTTTATGAGGTCCGCTTGCTCTCGCGAGTT
CGCTTCTCTTTGTATATGCCATTGTAGCACGTGTGTAGCCCTACTCGTAAGGGCCATGATGACTTGACGTCATCCCCACCTTCCTCCGGTTTATCACCGG
CAGTCTCCTTTGAGTTCCCGCCATTACGCGCTGGCAACAAAGGATAAGGGTTGCGCTCGTTGCGGGACTTAACCCAACATTTCACAACACGAGCTGACGA
CAGCCATGCAGCACCTGTCTCACAGTTCCCGAAGGCACCAATCCATCTCTGGAAAGTTCTGTGGATGTCAAGAGTAGGTAAGGTTCTTCGCGTTGCATCG
AATTAAACCACATGCTCCACCGCTTGTGCGGGCCCCCGTCAATTCATTTGAGTTTTAACCTTGCGGCCGTACTCCCCAGGCGGTCGACTTAACGCGTTAG
CTCCGGAAGCCACGCCTCAAGGGCACAACCTCCAAGTCGACATCGTTTACAGCGTGGACTACCAGGGTATCTAATCCTGTTTGCTCCCCACGCTTTCGCA
CCTGAGCGTCAGTCTTTGTCCAGGGGGCCGCCTTCGCCACCGGTATTCCTCCAGATCTCTACGCATTTCACCGCTACACCTGGAATTCTACCCCCCTCTA
CAAGACTCTAGCTTGCCAGTTTCAAATGCAGTTCCCAAGTTAAGCTCGGGGATTTCACATCTGACTTAACAAACCGCCTGCGTGCGCTTTACGCCCAGTA
ATTCCGATTAACGCTTGCACCCTCCGTATTACCGCGGCTGCTGGCACGGAGTTAGCCGGTGCTTCTTCTGCGAGTAACGTCAATGAACGGTGCTATTAAC
ACCGAACCCTTCCTCCTCGCTGAAAGTGCTTTACAACCCGAAGGCCTTCTTCACACACGCGGCATGGCTGCATCAGGCTTGCGCCCATTGTGCAATATTC
CCCACTGCTGCCTCCCGTAGGAGTCTGGACCGTGTCTCAGTTCCAGTGTGGCTGGTCATCCTCTCAGACCAGCTAGGGATCGTCGCCTAGGTGAGCCATT
ACCTCACCTACTAGCTAATCCCATCTGGGCACATCCGATGGTGTGAGGCCCGAAGGTCCCCCACTTTGGTCCGAAGACGTTATGCGGTATTAGCTACCGT
TTCCAGTAGTTATCCCCCTCCATCGGGCAGTTTCCCAGACATTACTCACCCGTCCGCCGCTCGTCACCCAGGAGCAAGCTCCCTGTGCTACCGCTCGACT
TGCATG

>EB332
TCCTTTGACGTTGGCGGCGGACGGGTGAGTAACACGTAGGTAACCTACCTATAAGACTGGGATAACTTCGGGAAACCGGAGCTAATACCGGATAATATTT
CGAACCGCATGGTTCGATAGTGAAAGATGGCTTTGCTATCACTTATAGATGGACCTGCGCCGTATTAGCTAGTTGGTAAGGTAACGGCTTACCAAGGCAA
CGATACGTAGCCGACCTGAGAGGGTGATCGGCCACACTGGAACTGAGACACGGTCCAGACTCCTACGGGAGGCAGCAGTAGGGAATCTTCCGCAATGGGC
GAAAGCCTGACGGAGCAACGCCGCGTGAGTGATGAAGGTCTTCGGATCGTAAAACTCTGTTATTAGGGAAGAACAAACGTGTAAGTAACTGTGCACGTCT
TGACGGTACCTAATCAGAAAGCCACGGCTAACTACGTGCCAGCAGCCGCGGTAATACGTAGGTGGCAAGCGTTATCCGGAATTATTGGGCGTAAAGCGCG
CGTAGGCGGTTTTTTAAGTCTGATGTGAAAGCCCACGGCTCAACCGTGGAGGGTCATTGGAAACTGGAAAACTTGAGTGCAGAAGAGGAAAGTGGAATTC
CATGTGTAGCGGTGAAATGCGCAGAGATATGGAGGAACACCAGTGGCGAAGGCGACTTTCTGGTCTGTAACTGACGCTGATGTGCGAAAGCGTGGGGATC
AAACAGGATTAGATACCCTGGTAGTCCACGCCGTAAACGATGAGTGCTAAGTGTTAGGGGGTTTCCGCCCCTTAGTGCTGCAGCTAACGCATTAAGCACT
CCGCCTGGGGAGTACGACCGCAAGGTTGAAACTCAAAGGAATTGACGGGGACCCGCACAAGCGGTGGAGCATGTGGTTTAATTCGAAGCAACGCGAAGAA
CCTTACCAAATCTTGACATCCTTTGACCCTTCTAGANGATAGAAGTTTCCCCTTCGGGGGACAAAGTGACAGGTGGTGCATGGTTGTCGTCAGCTCGTGT
CGTGAGATGTTGGGTTAAGTCCCGCAACGAGCGCAACCCTTAAGCTTAGTTGCCATCATTAAGTTGGGCACTCTAAGTTGACTGCCGGTGACAAACCGGA
GGAAGGTGGGGATGACGTCAAATCATCATGCCCCTTATGATTTGGGCTACACACGTGCTACAATGGACAATACAAAGGGCAGCGAAACCGCGAGGTCAAG
CAAATCCCATAAAGTTGTTCTCAGTTCGGATTGTAGTCTGCAACTCGACTACATGAAGCTGGAATCGCTAGTAATCGTAGATCAGCATGCTACGGTGAAT
ACGTTCCCGGGTCTTGTACACACCGCCCGTCACACCACGAGAGTTTGTAACTAGAGAAGCCGGTGG

>EB333
TGCAAGTCGAGCGGTAGCACAAGGGGAGCTTGCTCCCTGGGTGACGAGCGGCGGACGGGTGAGTAATGTCTGGGAAACTGCCTGATGGAGGGGGATAACT
ACTGGAAACGGTAGCTAATACCGCATAACGTCGCAAGACCAAAGAGGGGGACCTTCGGGCCTCTTGCCATCAGATGTGCCCAGATGGGATTAGCTAGTAG
GTGGGGTAATGGCTCACCTAGGCGACGATCCCTAGCTGGTCTGAGAGGATGACCAGCCACACTGGAACTGAGACACGGTCCAGACTCCTACGGGAGGCAG
CAGTGGGGAATATTGCACAATGGGCGCAAGCCTGATGCAGCCATGCCGCGTGTGTGAAGAAGGCCTTCGGGTTGTAAAGCACTTTCAGCGAGGAGGAAGG
TGGTGAGCTTAATACGCTCATCAATTGACGTTACTCGCAGAAGAAGCACCGGCTAACTCCGTGCCAGCAGCCGCGGTAATACGGAGGGTGCAAGCGTTAA
TCGGAATTACTGGGCGTAAAGCGCACGCAGGCGGTTTGTTAAGTCAGATGTGAAATCCCCGGGCTCAACCTGGGAACTGCATTTGAAACTGGCAAGCTAG
AGTCTCGTAGAGGGGGGTAGAATTCCAGGTGTAGCGGTGAAATGCGTAGAGATCTGGAGGAATACCGGTGGCGAAGGCGGCCCCCTGGACGAAGACTGAC
GCTCAGGTGCGAAAGCGTGGGGAGCAAACAGGATTAGATACCCTGGTAGTCCACGCTGTAAACGATGTCGATTTGGAGGTTGTGCCCTTGAGGCGTGGCT
TCCGGAGCTAACGCGTTAAATCGACCGCCTGGGGAGTACGGCCGCAAGGTTAAAACTCAAATGAATTGACGGGGGCCCGCACAAGCGGTGGAGCATGTGG
TTTAATTCGATGCAACGCGAAGAACCTTACCTACTCTTGACATCCAGAGAACTTAGCAGAGATGCTTTGGTGCCTTCGGGAACTCTGAGACAGGTGCTGC
ATGGCTGTCGTCAGCTCGTGTTGTGAAATGTTGGGTTAAGTCCCGCAACGAGCGCAACCCTTATCCTTTGTTGCCAGCGGTTCGGCCGGGAACTCAAAGG
AGACTGCCAGTGATAAACTGGAGGAAGGTGGGGATGACGTCAAGTCATCATGGCCCTTACGAGTAGGGCTACACACGTGCTACAATGGCATATACAAAGA
GAAGCGACCTCGCGAGAGCAAGCGGACCTCATAAAGTATGTCGTAGTCCGGATTGGAGTCTGCAACTCGACTCCATGAAGTCGGAATCGCTAGTAATCGT
AGATCAGAATGCTACGGTGAATACGTTCCCGGGCCTTGTACACACCGCCCGTCACACCATGGGAGTGGGTTGCAAAAGAAGTAGGTAGCTTAACCTTCGG
GAGGGCGCTTA

>EB334

TGCAAGTCGAGCGGTAGCACAGGAGAGCTTGCTCTCTGGGTGACGAGCGGCGGACGGGTGAGTAATGTCTGGGAAACTGCCTGATGGAGGGGGATAACTA
CTGGAAACGGTAGCTAATACCGCATAACGTCTACGGACCAAAGTGGGGGACCTTCGGGCCTCACGCCATCAGATGTGCCCAGATGGGATTAGCTAGTAGG
TGGGGTAATGGCTCACCTAGGCGACGATCCCTAGCTGGTCTGAGAGGATGACCAGCCACACTGGAACTGAGACACGGTCCAGACTCCTACGGGAGGCAGC
AGTGGGGAATATTGCACAATGGGCGCAAGCCTGATGCAGCCATGCCGCGTGTGTGAAGAAGGCCTTAGGGTTGTAAAGCACTTTCAGCGAGGAGGAAGGG
TTCAGTGTTAATAGCACTGTGCATTGACGTTACTCGCAGAAGAAGCACCGGCTAACTCCGTGCCAGCAGCCGCGGTAATACGGAGGGTGCAAGCGTTAAT
CGGAATTACTGGGCGTAAAGCGCACGCAGGCGGTTTGTTAAGTCAGATGTGAAATCCCCGCGCTTAACGTGGGAACTGCATTTGAAACTGGCAAGCTAGA
GTCTTGTAGAGGGGGGTAGAATTCCAGGTGTAGCGGTGAAATGCGTAGAGATCTGGAGGAATACCGGTGGCGAAGGCGGCCCCCTGGACAAAGACTGACG
CTCAGGTGCGAAAGCGTGGGGAGCAAACAGGATTAGATACCCTGGTAGTCCACGCTGTAAACGATGTCGATTTGGAGGTTGTGCCCTTGAGGCGTGGCTT
CCGGAGCTAACGCGTTAAATCGACCGCCTGGGGAGTACGGCCGCAAGGTTAAAACTCAAATGAATTGACGGGGGCCCGCACAAGCGGTGGAGCATGTGGT
TTAATTCGATGCAACGCGAAGAACCTTACCTACTCTTGACATCCAGAGAACTTTCCAGAGATGGATTGGTGCCTTCGGGAACTCTGAGACAGGTGCTGCA
TGGCTGTCGTCAGCTCGTGTTGTGAAATGTTGGGTTAAGTCCCGCAACGAGCGCAACCCTTATCCTTTGTTGCCAGCGATTCGGTCGGGAACTCAAAGGA
GACTGCCGGTGATAAACCGGAGGAAGGTGGGGATGACGTCAAGTCATCATGGCCCTTACGAGTAGGGCTACACACGTGCTACAATGGCGTATACAAAGAG
AAGCGAACTCGCGAGAGCAAGCGGACCTCATAAAGTACGTCGTAGTCCGGATTGGAGTCTGCAACTCGACTCCATGAAGTCGGAATCGCTAGTAATCGTA
GATCAGAATGCTACGGTGAATACGTTCCCGGGCCTTGTACACACCGCCCGTCACACCATGGGAGTGGGTTGCAAAAGAAGTAGGTAGCTTAACCTTCGGG
AGGGCGC

>EB335
TGCTCAGGATGAACGCTGGCGGCGTGCCTAATACATGCAAGTCGAGCGAACAGACGGGGAGCTTGCTCCCCTGACGTTAGCGGCGGACGGGTGAGTAACA
CGTGGGTAACCTACCTATAAGACTGGAATAACTCCGGGAAACCGGGGCTAATGCCGGATAACATGTTGGACCGCATGGTCCTACAGTGAAAGACGGTCTT
GCTGTCACTTATAGATGGACCCGCGCCGTATTAGCTAGTTGGTAAGGTAACGGCTTACCAAGGCAACGATACGTAGCCGACCTGAGAGGGTGATCGGCCA
CACTGGAACTGAGACACGGTCCAGACTCCTACGGGAGGCAGCAGTAGGGAATCTTCCGCAATGGGCGAAAGCCTGACGGAGCAACGCCGCGTGAGTGATG
AAGGCCTTCGGGTCGTAAAACTCTGTTATTAGGGAAGAACAAATGTGTAAGTAACTATGCACGTCTTGACGGTACCTAATCAGAAAGCCACGGCTAACTA
CGTGCCAGCAGCCGCGGTAATACGTAGGTGGCAAGCGTTATCCGGAATTATTGGGCGTAAAGCGCGCGTAGGCGGTTTCTTAAGTCTGATGTGAAAGCCC
ACGGCTCAACCGTGGAGGGTCATTGGAAACTGGGAAACTTGAGTGCAGGAGAGGAAAGTGGAATTCCATGTGTAGCGGTGAAATGCGCAGAGATATGGAG
GAACACCAGTGGCGAAGGCGACTTTCTGGTCTGCAACTGACGCTGATGTGCGAAAGCGTGGGGATCAAACAGGATTAGATACCCTGGTAGTCCACGCCGT
AAACGATGAGTGCTAAGTGTTAGGGGGTTTCCGCCCCTTAGTGCTGCAGCTAACGCATTAAGCACTCCGCCTGGGGAGTACGACCGCAAGGTTGAAACTC
AAAGGAATTGACGGGGACCCGCACAAGCGGTGGAGCATGTGGTTTAATTCGAAGCAACGCGAAGAACCTTACCAAATCTTGACATCCTTTGCCCCCTCTA
GAGATAGAGGTTTCCCCTTCGGGGGACAAAGTGACAGGTGGTGCATGGTTGTCGTCAGCTCGTGTCGTGAGATGTTGGGTTAAGTCCCGCAACGAGCGCA
ACCCTTAAGCTTAGTTGCCATCATTCAGTTGGGCACTCTAAGTTGACTGCCGGTGACAAACCGGAGGAAGGTGGGGATGACGTCAAATCATCATGCCCCT
TATGATTTGGGCTACACACGTGCTACAATGGACAATACAAAGGGCAGCTAAACCGCGAGGTCAAGCAAATCCCATAAAGTTGTTCTCAGTTCGGATTGTA
GTCTGCAACTCGACTACATGAAGCTGGAATCGCTAGTAATCGTAGATCAGCATGCTACGGTGAATACGTTCCCGGGTCTTGTACACACCGCCCGTCACAC
CACGAGAGTTTGTAACACCCGAAGCCGGTGGAGCAACCACTTTGTGGAGCTAGCCGTCCAA

>EB336
CTTGCTTCTCTTAGAAAGCGGCGGACGGGTGAGTAATGCCTAGGAATCTGCCTGGTAGTGGGGGATAACGTTCGGAAACGGACGCTAATACCGCATACGT
CCTACGGGAGAAAGCAGGGGACCTTCGGGCCTTGCGCTATCAGATGAGCCTAGGTCGGATTAGCTAGTTGGTGAGGTAATGGCTCACCAAGGCGACGATC
CGTAACTGGTCTGAGAGGATGATCAGTCACACTGGAACTGAGACACGGTCCAGACTCCTACGGGAGGCAGCAGTGGGGAATATTGGACAATGGGCGAAAG
CCTGATCCAGCCATGCCGCGTGTGTGAAGAAGGTCTTCGGATTGTAAAGCACTTTAAGTTGGGAGGAAGGGTTGTAGATTAATACTCTGCAATTTTGACG
TTACCGACAGAATAAGCACCGGCTAACTCTGTGCCAGCAGCCGCGGTAATACAGAGGGTGCAAGCGTTAATCGGAATTACTGGGCGTAAAGCGCGCGTAG
GTGGTTTGTTAAGTTGGATGTGAAATCCCCGGGCTCAACCTGGGAACTGCATTCAAAACTGACTGACTAGAGTATGGTAGAGGGTGGTGGAATTTCCTGT
GTAGCGGTGAAATGCGTAGATATAGGAAGGAACACCAGTGGCGAAGGCGACCACCTGGACTAATACTGACACTGAGGTGCGAAAGCGTGGGGAGCAAACA
GGATTAGATACCCTGGTAGTCCACGCCGTAAACGATGTCAACTAGCCGTTGGAAGCCTTGAGCTTTTAGTGGCGCAGCTAACGCATTAAGTTGACCGCCT
GGGGAGTACGGCCGCAAGGTTAAAACTCAAATGAATTGACGGGGGCCCGCACAAGCGGTGGAGCATGTGGTTTAATTCGAAGCAACGCGAAGAACCTTAC
CAGGCCTTGACATCCAATGAACTTTCTAGAGATAGATTGGTGCCTTCGGGAACATTGAGACAGGTGCTGCATGGCTGTCGTCAGCTCGTGTCGTGAGATG
TTGGGTTAAGTCCCGTAACGAGCGCAACCCTTGTCCTTAGTTACCAGCACGTTATGGTGGGCACTCTAAGGAGACTGCCGGTGACAAACCGGAGGAAGGT
GGGGATGACGTCAAGTCATCATGGCCCTTACGGCCTGGGCTACACACGTGCTACAATGGTCGGTACAGAGGGTTGCCAAGCCGCGAGGTGGAGCTAATCC
CATAAAACCGATCGTAGTCCGGATCGCAGTCTGCAACTCGACTGCGTGAAGTCGGAATCGTTAGTAATCGCGAATCAGAATGTCGCGGTGAATACGTTCC
CGTACATTGTACACACCGCTCATCACACC

>EB337
TGCAAGTCGAACGATGATGCCCAGCTTGCTGGGTGGATTAGTGGCGAACGGGTGAGTAACACGTGAGTAACCTGCCCCTGACTCTGGGATAAGCGTTGGA
AACGACGTCTAATACTGGATATGATCACTGGCCGCATGGTCTGGTGGTGGAAAGATTTTTTGGTTGGGGATGGACTCGCGGCCTATCAGCTTGTTGGTGA
GGTAATGGCTCACCAAGGCGACGACGGGTAGCCGGCCTGAGAGGGTGACCGGCCACACTGGGACTGAGACACGGCCCAGACTCCTACGGGAGGCAGCAGT
GGGGAATATTGCACAATGGGCGAAAGCCTGATGCAGCAACGCCGCGTGAGGGATGACGGCCTTCGGGTTGTAAACCTCTTTTAGTAGGGAAGAAGCGAAA
GTGACGGTACCTGCAGAAAAAGCACCGGCTAACTACGTGCCAGCAGCCGCGGTAATACGTAGGGTGCAAGCGTTGTCCGGAATTATTGGGCGTAAAGAGC
TCGTAGGCGGTTTGTCGCGTCTGCTGTGAAATCCCGAGGCTCAACCTCGGGCTTGCAGTGGGTACGGGCAGACTAGAGTGCGGTAGGGGAGATTGGAATT
CCTGGTGTAGCGGTGGAATGCGCAGATATCAGGAGGAACACCGATGGCGAAGGCAGATCTCTGGGCCGTAACTGACGCTGAGGAGCGAAAGCATGGGGAG
CGAACAGGATTAGATACCCTGGTAGTCCATGCCGTAAACGTTGGGCGCTAGATGTAGGGACCTTTCCACGGTTTCTGTGTCGTAGCTAACGCATTAAGCG
CCCCGCCTGGGGAGTACGGCCGCAAGGCTAAAACTCAAAGGAATTGACGGGGGCCCGCACAAGCGGCGGAGCATGCGGATTAATTCGATGCAACGCGAAG
AACCTTACCAAGGCTTGACATACACCGGAAACGGCCAGAGATGGTCGCCCCCTTGTGGTCGGTGTACAGGTGGTGCATGGTTGTCGTCAGCTCGTGTCGT
GAGATGTTGGGTTAAGTCCCGCAACGAGCGCAACCCTCGTTCTATGTTGCCAGCGGGTTATGCCGGGGACTCATAGGAGACTGCCGGGGTCAACTCGGAG
GAAGGTGGGGATGACGTCAAATCATCATGCCCCTTATGTCTTGGGCTTCACGCATGCTACAATGGCCGGTACAAAGGGCTGCGATACCGTAAGGTGGAGC
GAATCCCAAAAAGCCGGTCTCAGTTCGGATTGAGGTCTGCAACTCGACCTCATGAAGTCGGAGTCGCTAGTAATCGCAGATCAGCAACGCTGCGGTGAAT
ACGTTCCCGGGCCTTGTACACACCGCCCGT
CAAGTCATGAAAGTCGGTAACACCCGAAGCCGGTGGCCTAACCCTTGTGGAAGGAGCCGTCGAAGGTGGG
ATCGGTGATTAGGACTAA

>EB338
GCGCCCTCCCGAAGGTTAAGCTACCTACTTCTTTTGCAACCCACTCCCATGGTGTGACGGGCGGTGTGTACAAGGCCCGGGAACGTATTCACCGTAGCAT
TCTGATCTACGATTACTAGCGATTCCGACTTCATGGAGTCGAGTTGCAGACTCCAATCCGGACTACGACGTACTTTATGAGGTCCGCTTACTCTCGCGAG
CTCGCTTCTCTTTGTATACGCCATTGTAGCACGTGTGTAGCCCTACTCGTAAGGGCCATGATGACTTGACGTCATCCCCACCTTCCTCCGGTTTATCACC
GGCAGTCTCCTTTGAGTTCCCGACCGAATCGCTGGCAACAAAGGATAAGGGTTGCGCTCGTTGCGGGACTTAACCCAACATTTCACAACACGAGCTGACG
ACAGCCATGCAGCACCTGTCTCAGAGTTCCCGAAGGCACCAATCCATCTCTGGAAAGTTCTCTGGATGTCAAGAGTAGGTAAGGTTCTTCGCGTTGCATC
GAATTAAACCACATGCTCCACCGCTTGTGCGGGCCCCCGTCAATTCATTTGAGTTTTAACCTTGCGGCCGTACTCCCCAGGCGGTCGATTTAACGCGTTA
GCTCCGGAAGCCACGCCTCAAGGGCACAACCTCCAAATCGACATCGTTTACAGCGTGGACTACCAGGGTATCTAATCCTGTTTGCTCCCCACGCTTTCGC
ACCTGAGCGTCAGTCTTCGTCCAGGGGGCCGCCTTCGCCACCGGTATTCCTCCAGATCTCTACGCATTTCACCGCTACACCTGGAATTCTACCCCCCTCT
ACGAGACTCTAGCTTGCCAGTTTCAAATGCAGTTCCCACGTTAAGCGCGGGGATTTCACATCTGACTTAACAAACCGCCTGCGTGCGCTTTACGCCCAGT
AATTCCGATTAACGCTTGCACCCTCCGTATTACCGCGGCTGCTGGCACGGAGTTAGCCGGTGCTTCTTCTGCGAGTAACGTCAATGTAATGCCGTATTAA

GACACTACCCTTCCTCCTCGCTGAAAGTGCTTTACAACCCGAAGGCCTTCTTCACACACGCGGCATGGCTGCATCAGGCTTGCGCCCATTGTGCAATATT
CCCCACTGCTGCCTCCCGTAGGAGTCTGGACCGTGTCTCAGTTCCAGTGTGGCTGGTCATCCTCTCAGACCAGCTAGGGATCGTCGCCTAGGTGAGCCAT
TACCCCACCTACTAGCTAATCCCATCTGGGCACATCTGATGGCGTGAGGCCCGAAGGTCCCCCACTTTGGTCCGAAGACGTTATGCGGTATTAGCTACCG
TTTCCAGTAGTTATCCCCCTCCATCAGGCAGTTTCCCAGACATTACTCACCCGTCCGCCGCTCGTCACCCAGGGAGCAAGCTCCCCCGTGCTACCGCTCG
AC

>EB339
GCGCCCTCCCGAAGGTTAAGCTACCTACTTCTTTTGCAACCCACTCCCATGGTGTGACGGGCGGTGTGTACAAGGCCCGGGAACGTATTCACCGTAGCAT
TCTGATCTACGATTACTAGCGATTCCGACTTCACGGAGTCGAGTTGCAGACTCCGATCCGGACTACGACATACTTTATGAGGTCCGCTTGCTCTCGCGAG
TTCGCTTCTCTTTGTATATGCCATTGTAGCACGTGTGTAGCCCTACTCGTAAGGGCCATGATGACTTGACGTCATCCCCACCTTCCTCCGGTTTATCACC
GGCAGTCTCCTTTGAGTTCCCGCCCATTACGCGCTGGCAACAAAGGATAAGGGTTGCGCTCGTTGCGGGACTTAACCCAACATTTCACAACACGAGCTGA
CGACAGCCATGCAGCACCTGTCTCACAGTTCCCGAAGGCACCAATCCATCTCTGGAAAGTTCTGTGGATGTCAAGAGTAGGTAAGGTTCTTCGCGTTGCA
TCGAATTAAACCACATGCTCCACCGCTTGTGCGGGCCCCCGTCAATTCATTTGAGTTTTAACCTTGCGGCCGTACTCCCCAGGCGGTCGACTTAACGCGT
TAGCTCCGGAAGCCACGCCTCAAGGGCACAACCTCCAAGTCGACATCGTTTACAGCGTGGACTACCAGGGTATCTAATCCTGTTTGCTCCCCACGCTTTC
GCACCTGAGCGTCAGTCTTTGTCCAGGGGGCCGCCTTCGCCACCGGTATTCCTCCAGATCTCTACGCATTTCACCGCTACACCTGGAATTCTACCCCCCT
CTACAAGACTCTAGCTTGCCAGTTTCAAATGCAGTTCCCAAGTTAAGCTCGGGGATTTCACATCTGACTTAACAAACCGCCTGCGTGCGCTTTACGCCCA
GTAATTCCGATTAACGCTTGCACCCTCCGTATTACCGCGGCTGCTGGCACGGAGTTAGCCGGTGCTTCTTCTGCGAGTAACGTCAATGAACGGTGCTATT
AACACCGAACCCTTCCTCCTCGCTGAAAGTGCTTTACAACCCGAAGGCCTTCTTCACACACGCGGCATGGCTGCATCAGGCTTGCGCCCATTGTGCAATA
TTCCCCACTGCTGCCTCCCGTAGGAGTCTGGACCGTGTCTCAGTTCCAGTGTGGCTGGTCATCCTCTCAGACCAGCTAGGGATCGTCGCCTAGGTGAGCC
ATTACCTCACCTACTAGCTAATCCCATCTGGGCACATCCGATGGTGTGAGGCCCGAAGGTCCCCCACTTTGGTCCGAAGACGTTATGCGGTATTAGCTAC
CGTTTCCAGTAGTTATCCCCCTCCATCGGGCAGTTTCCCAGACATTACTCACCCGTCCGCCGCTCGTCACCCAGGAGCAAGCTCCCTGTGCTACCGCTCG
ACTGCATGTGTAGC

>EB340
CCCTCCCGAAGGTTAAGCTACCTACTTCTTTTGCAACCCACTCCCATGGTGTGACGGGCGGTGTGTACAAGGCCCGGGAACGTATTCACCGTAGCATTCT
GATCTACGATTACTAGCGATTCCGACTTCATGGAGTCGAGTTGCAGACTCCAATCCGGACTACGACATACTTTATGAGGTCCGCTTGCTCTCGCGAGGTC
GCTTCTCTTTGTATATGCCATTGTAGCACGTGTGTAGCCCTACTCGTAAGGGCCATGATGACTTGACGTCATCCCCACCTTCCTCCAGTTTATCACTGGC
AGTCTCCTTTGAGTTCCCGGCCGAACCGCTGGCAACAAAGGATAAGGGTTGCGCTCGTTGCGGGACTTAACCCAACATTTCACAACACGAGCTGACGACA
GCCATGCAGCACCTGTCTCAGAGTTCCCGAAGGCACCAATCCATCTCTGGAAAGTTCTCTGGATGTCAAGAGTAGGTAAGGTTCTTCGCGTTGCATCGAA
TTAAACCACATGCTCCACCGCTTGTGCGGGCCCCCGTCAATTCATTTGAGTTTTAACCTTGCGGCCGTACTCCCCAGGCGGTCGATTTAACGCGTTAGCT
CCGGAAGCCACGCCTCAAGGGCACAACCTCCAAATCGACATCGTTTACAGCGTGGACTACCAGGGTATCTAATCCTGTTTGCTCCCCACGCTTTCGCACC
TGAGCGTCAGTCTTCGTCCAGGGGGCCGCCTTCGCCACCGGTATTCCTCCAGATCTCTACGCATTTCACCGCTACACCTGGAATTCTACCCCCCTCTACG
AGACTCTAGCTTGCCAGTTTCAAATGCAGTTCCCAGGTTGAGCCCGGGGATTTCACATCTGACTTAACAAACCGCCTGCGTGCGCTTTACGCCCAGTAAT
TCCGATTAACGCTTGCACCCTCCGTATTACCGCGGCTGCTGGCACGGAGTTAGCCGGTGCTTCTTCTGCGAGTAACGTCAATTGATGAACGTATTAAGTT
CACCACCTTCCTCCTCGCTGAAAGTGCTTTACAACCCGAAGGCCTTCTTCACACACGCGGCATGGCTGCATCAGGCTTGCGCCCATTGTGCAATATTCCC
CACTGCTGCCTCCCGTAGGAGTCTGGACCGTGTCTCAGTTCCAGTGTGGCTGGTCATCCTCTCAGACCAGCTAGGGATCGTCGCCTAGGTGAGCCATTAC
CCCACCTACTAGCTAATCCCATCTGGGCACATCTGATGGCAAGAGGCCCGAAGGTCCCCCTCTTTGGTCTTGCGACGTTATGCGGTATTAGCTACCGTTT
CCAGTAGTTATCCCCCTCCATCAGGCAGTTTCCCAGACATTACTCACCCGTCCGCCGCTCGTCACCCGGGGAGCAAGCTCCCCTGTGCTACCGCTCGACT
GCA

>EB341
TGCAGTCGAGCGGTAGCACAGGGGAGCTTGCTCCCCGGGTGACGAGCGGCGGACGGGTGAGTAATGTCTGGGAAACTGCCTGATGGAGGGGGATAACTAC
TGGAAACGGTAGCTAATACCGCATAACGTCGCAAGACCAAAGAGGGGGACCTTCGGGCCTCTTGCCATCAGATGTGCCCAGATGGGATTAGCTAGTAGGT
GGGGTAATGGCTCACCTAGGCGACGATCCCTAGCTGGTCTGAGAGGATGACCAGCCACACTGGAACTGAGACACGGTCCAGACTCCTACGGGAGGCAGCA
GTGGGGAATATTGCACAATGGGCGCAAGCCTGATGCAGCCATGCCGCGTGTGTGAAGAAGGCCTTCGGGTTGTAAAGCACTTTCAGCGAGGAGGAAGGTG
GTGAACTTAATACGTTCATCAATTGACGTTACTCGCAGAAGAAGCACCGGCTAACTCCGTGCCAGCAGCCGCGGTAATACGGAGGGTGCAAGCGTTAATC
GGAATTACTGGGCGTAAAGCGCACGCAGGCGGTTTGTTAAGTCAGATGTGAAATCCCCGGGCTCAACCTGGGAACTGCATTTGAAACTGGCAAGCTAGAG
TCTCGTAGAGGGGGGTAGAATTCCAGGTGTAGCGGTGAAATGCGTAGAGATCTGGAGGAATACCGGTGGCGAAGGCGGCCCCCTGGACGAAGACTGACGC
TCAGGTGCGAAAGCGTGGGGAGCAAACAGGATTAGATACCCTGGTAGTCCACGCTGTAAACGATGTCGATTTGGAGGTTGTGCCCTTGAGGCGTGGCTTC
CGGAGCTAACGCGTTAAATCGACCGCCTGGGGAGTACGGCCGCAAGGTTAAAACTCAAATGAATTGACGGGGGCCCGCACAAGCGGTGGAGCATGTGGTT
TAATTCGATGCAACGCGAAGAACCTTACCTACTCTTGACATCCAGAGAACTTTTCCAGAGATGGATTGGTGCCTTCGGGAACTCTGAGACAGGTGCTGCA
TGGCTGTCGTCAGCTCGTGTTGTGAAATGTTGGGTTAAGTCCCGCAACGAGCGCAACCCTTATCCTTTGTTGCCAGCGGTTCGGCCGGGAACTCAAAGGA
GACTGCCAGTGATAAACTGGAGGAAGGTGGGGATGACGTCAAGTCATCATGGCCCTTACGAGTAGGGCTACACACGTGCTACAATGGCATATACAAAGAG
AAGCGACCTCGCGAGAGCAAGCGGACCTCATAAAGTATGTCGTAGTCCGGATTGGAGTCTGCAACTCGACTCCATGAAGTCGGAATCGCTAGTAATCGTA
GATCAGAATGCTACGGTGAATACGTTCCCGGGCCTTGTACACACCGCCCGTCACACCATGGGAGTGGGTTGCAAAAGAAGTAGGTAGCTTAACCTTCGGG
AGGGCGCTTA

>EB342
TAACACGTGAGCAACCTGCCCCTGACTCTGGGATAAGCGCTGGAAACGGCGTCTAATACTGGATACGAGATGTGACCGCATGGTCGATGTCTGGAAAGAT
TTTTCGGTTGGGGATGGGCTCGCGGCCTATCAGCTTGTTGGTGAGGTAATGGCTCACCAAGGCGTCGACGGGTAGCCGGCCTGAGAGGGTGACCGGCCAC
ACTGGGACTGAGACACGGCCCAGACTCCTACGGGAGGCAGCAGTGGGGAATATTGCACAATGGGCGGAAGCCTGATGCAGCAACGCCGCGTGAGGGATGA
CGGCCTTCGGGTTGTAAACCTCTTTTAGCAAGGAAGAAGCGAAAGTGACGGTACTTGCAGAAAAAGCGCCGGCTAACTACGTGCCAGCAGCCGCGGTAAT
ACGTAGGGCGCAAGCGTTATCCGGAATTATTGGGCGTAAAGAGCTCGTAGGCGGTTTGTCGCGTCTGCTGTGAAATCCCGAGGCTCAACCTCGGGCCTGC
AGTGGGTACGGGCAGACTAGAGTGCGGTAGGGGAGATTGGAATTCCTGGTGTAGCGGTGGAATGCGCAGATATCAGGAGGAACACCGATGGCGAAGGCAG
ATCTCTGGGCCGTAACTGACGCTGAGGAGCGAAAGGGTGGGGAGCAAACAGGCTTAGATACCCTGGTAGTCCACCCCGTAAACGTTGGGAACTAGTTGTG
GGGTCCTTTCCACGGATTCCGTGACGCAGCTAACGCATTAAGTTCCCCGCCTGGGGAGTACGGCCGCAAGGCTAAAACTCAAAGGAATTGACGGGGACCC
GCACAAGCGGCGGAGCATGCGGATTAATTCGATGCAACGCGAAGAACCTTACCAAGGCTTGACATACACGAGAACACCCTAGAAATAGGGGACTCTTTGG
ACACTCGTGAACAGGTGGTGCATGGTTGTCGTCAGCTCGTGTCGTGAGATGTTGGGTTAAGTCCCGCAACGAGCGCAACCCTCGTTCTATGTTGCCAGCA
CGTAATGGTGGGAACTCATGGGATACTGCCGGGGTCAACTCGGAGGAAGGTGGGGATGACGTCAAATCATCATGCCCCTTATGTCTTGGGCTTCACGCAT
GCTACAATGGCCGGTACAAAGGGCTGCAATACCGTGAGGTGGAGCGAATCCCAAAAAGCCGGTCCCAGTTCGGATTGAGGTCTGCAACTCGACCTCATGA
AGTCGGAGTCGCTTGTAATCGCAGATCAGCAACGCTGCGGTGAATACGTTCCCGGTTCTTGTACACACCGCCCCTCAAGTCATGAACATCGTTAAC

>EB343
AACGGGTGAGTAACACGTGAGTAACCTGCCCCTGACTCTGGGATAAGCGTTGGAAACGACGTCTAATACTGGATATGATCACTGGCCGCATGGTCTGGTG
GTGGAAAGATTTTTTGGTTGGGGATGGACTCGCGGCCTATCAGCTTGTTGGTGAGGTAATGGCTCACCAAGGCGACGACGGGTAGCCGGCCTGAGAGGGT
GACCGGCCACACTGGGACTGAGACACGGCCCAGACTCCTACGGGAGGCAGCAGTGGGGAATATTGCACAATGGGCGAAAGCCTGATGCAGCAACGCCGCG
TGAGGGATGACGGCCTTCGGGTTGTAAACCTCTTTTAGTAGGGAAGAAGCGAAAGTGACGGTACCTGCAGAAAAAGCACCGGCTAACTACGTGCCAGCAG
CCGCGGTAATACGTAGGGTGCAAGCGTTGTCCGGAATTATTGGGCGTAAAGAGCTCGTAGGCGGTTTGTCGCGTCTGCTGTGAAATCCCGANGGCTCAAC

CTCGGGCTTGCAGTGGGTANNCGGGCAGACTANGANGTGCGGTANGGGGAGATTNGGAATTCCTGGTGTAGCGGTGGAATGCNGCAGATATCAGGANGGA
ACACCGATGGCGAAGGCANGATCTNCTGGGCCGTAACTGANCGCTGAGGAGCGAAANGCATGGGGNAGCGNAACAGGATTAGATACCCCTGGNTAGTCCA
TGCCNGTAAACGTTGGGCGCTAGATGTAGGGNACCTTTCCACGGTTTNCTGTGTCGTAGCTAACGCATTAAGCGCCCCGCCTGGGGNAGTACGGCCGCAA
GGCTAAAACTCAAAGGAATTGACGGGGGNCCCGCACAAGCGGCGGAGCATGCGGATTAATTCGATGCAACGCGAAGAACCTTACCAAGGCTTGACATACA
CCGGAAACGGCCAGAGATGGTCGCCCCCTTGTGGTCGGTGTACAGGTGGTGCATGGTTGTCGTCAGCTCGTGTCGTGAGATGTTGGGTTAAGTCCCGCAA
CGAGCGCAACCCTCGTTCTATGTTGCCAGCGGGTTATGCCGGGGACTCATAGGAGACTGCCGGGGTCAACTCGGAGGAAGGTGGGGATGACGTCAAATCA
TCATGCCCCTTATGTCTTGGGCTTCACGCATGCTACAATGGCCGGTACAAAGGGCTGCGATACCGTAAGGTGGAGCGAATCCCAAAAAGCCGGTCTCAGT
TCGGATTGAGGTCTGCAACTCGACCTCATGAAGTCGGAGTCGCTTGTAATCGCAGATCAGCAACGCTGCGGTGAATACGTTCCCGGTCGTTGTACACACC
GCCTCTCAAGTCCGGAACATCG

>EB344
CCGGTTGGGCCATGAGCTTCGGGTAGTTACCAACTTTCGTGACTTGACGGGCGGTGTGTACAAGGCCCGGGAACGTATTCACCGCAGCGTTGCTGATCTG
CGATTACTAGCGACTCCGACTTCATGGGGTCGAGTTGCAGACCCCAATCCGAACTGAGACCGGCTTTTTGGGATTCGCTCCACCTTACGGTATCGCAGCC
CTCTGTACCGGCCATTGTAGCATGCGTGAAGCCCAAGACATAAGGGGCATGATGATTTGACGTCATCCCCACCTTCCTCCGAGTTGACCCCGGCAGTCTC
CCATGAGTCCCCGGCATAACCCGCTGGCAACATAGGACGAGGGTTGCGCTCGTTGCGGGACTTAACCCAACATCTCACGACACGAGCTGACGACAACCA
TGCACCACCTGTGCACCGACCTTGCGGGGCACCCATCTCTGAGTGTTTCCGGTGCATGTCAAGCCTTGGTAAGGTTCTTCGCGTTGCATCGAATTAATCC
GCATGCTCCGCCGCTTGTGCGGGCCCCCGTCAATTCCTTTGAGTTTTAGCCTTGCGGCCGTACTCCCCAGGCGGGGCACTTAATGCGTTAGCTGCGGCAC
GGAACTCGTGGAATGAGCCCCACACCTAGTGCCCAACGTTTACGGCATGGACTACCAGGGTATCTAATCCTGTTCGCTCCCCATGCTTTCGCTCCTCAGC
GTCAGTTGCGGCCCAGAGACCTGCCTTCGCCATCGGTGTTCCTCCTGATATCTGCGCATTCCACCGCTACACCAGGAATTCCAGTCTCCCCTACCGCACT
CTAGTCTGCCCGTACCCGATGCAAGCCCGAGGTTGAGCCTCGGGATTTCACACCAGACGCGACAGACCGCCTACGAGCTCTTTACGCCCAATAATTCCGG
ACAACGCTTGCGCCCTACGTATTACCGCGGCTGCTGGCACGTAGTTAGCCGGCGCTTCTTCTGCAGGTACCGTCACTTGCGCTTCTTCCCTGCTGAAAGA
GGTTTACAACCCGAAGGCCGTCATCCCTCACGCGGCGTCGCTGCATCAGGCTTTCGCCCATTGTGCAATATTCCCCACTGCTGCCTCCCGTAGGAGTCTG
GGCCGTGTCTCAGTCCCAGTGTGGCCGGTCGCCCTCTCAGGCCGGCTACCCGTCGACGCCTTGGTAGGCCATCACCCCACCAACAAGCTGATAGGCCGCG
AGCCCATCCCCCACCGAAAAACTTTCCAACACCCACCATGCGATGAGTGCTCATATCCGGTATTAGACCCCGTTTCCAAGGCTTATCCCGAAGTGGAGGG
CAGGTTGCTCACGTGTTACTCACCCGTTCGCCACTGATCCACCCAGCAAGCTGGGCTTCACCGTTCGACTTGCA

>EB345
TGGCGAACGGGTGAGTAACACGTGAGTAACCTGCCCCTGACTCTGGGATAAGCGTTGGAAACGACGTCTAATACTGGATATGATCACTGGTCGCATGGCC
TGGTGGTGGAAAGATTTTTTGGTTGGGGATGGACTCGCGGCCTATCAGCTTGTTGGTGAGGTAATGGCTCACCAAGGCGACGACGGGTAGCCGGCCTGAG
AGGGTGACCGGCCACACTGGGACTGAGACACGGCCCAGACTCCTACGGGAGGCAGCAGTGGGGAATATTGCACAATGGGCGAAAGCCTGATGCAGCAACG
CCGCGTGAGGGATGACGGCCTTCGGGTTGTAAACCTCTTTTAGTAGGGAAGAAGCGAAAGTGACGGTACCTGCAGAAAAAGCACCGGCTAACTACGTGCC
AGCAGCCGCGGTAATACGTAGGGTGCAAGCGTTGTCCGGAATTATTGGGCGTAAAGAGCTCGTAGGCGGTTTGTCGCGTCTGCTGTGAAATCCCGAGGCT
CAACCTCGGGCTTGCAGTGGGTACGGGCAGACTAGAGTGCGGTAGGGGAGATTGGAATTCCTGGTGTAGCGGTGGAATGCGCAGATATCAGGAGGAACAC
CGATGGCGAAGGCAGATCTCTGGGCCGTAACTGACGCTGAGGAGCGAAAGCATGGGGAGCGAACAGGATTAGATACCCTGGTAGTCCATGCCGTAAACGT
TGGGCGCTAGATGTAGGGACCTTTCCACGGTTTCTGTGTCGTAGCTAACGCATTAAGCGCCCCGCCTGGGGAGTACGGCCGCAAGGCTAAAACTCAAAGG
AATTGACGGGGGCCCGCACAAGCGGCGGAGCATGCGGATTAATTCGATGCAACGCGAAGAACCTTACCAAGGCTTGACATACACCGGAAACGGCCAGAGA
TGGTCGCCCCCTTGTGGTCGGTGTACAGGTGGTGCATGGTTGTCGTCAGCTCGTGTCGTGAGATGTTGGGTTAAGTCCCGCAACGAGCGCAACCCTCGTT
CTATGTTGCCAGCGGGTTATGCCGGGGACTCATAGGAGACTGCCGGGGTCAACTCGGAGGAAGGTGGGGATGACGTCAAATCATCATGCCCCTTATGTCT
TGGGCTTCACGCATGCTACAATGGCCGGTACAAAGGGCTGCGATACCGTAAGGTGGAGCGAATCCCAAAAAGCCGGTCTCAGTTCGGATTGAGGTCTGCA
ACTCGACCTCATGAAGTCGGAGTCGCTTGTAATCGCAGATCAGCAACGCTGCGGTGAATACGTTCCCGGTCCTTGTACACACCGCCCGTCAAGTCATGAA
AGTCGGTAA

>EB346
CGATGATCCGGAGCTTGCTCCTCGTGATTAGTGGCGAACGGGTGAGTAACACGTGAGTAACCTGCCCCTGACTCTGGGATAAGCGTTGGAAACGACGTCT
AATACTGGATACGATCACCGGCCGCATGGTCTGGTGGTGGAAAGATTTTTTGGTTGGGGATGGACTCGCGGCCTATCAGCTTGTTGGTGAGGTAATGGCT
CACCAAGGCGACGACGGGTAGCCGGCCTGAGAGGGTGACCGGCCACACTGGGACTGAGACACGGCCCAGACTCCTACGGGAGGCAGCAGTGGGGAATATT
GCACAATGGGCGAAAGCCTGATGCAGCAACGCCGCGTGAGGGATGACGGCCTTCGGGTTGTAAACCTCTTTTAGTAGGGAAGAAGCGAAAGTGACGGTAC
CTGCAGAAAAAGCACCGGCTAACTACGTGCCAGCAGCCGCGGTAATACGTAGGGTGCAAGCGTTGTCCGGAATTATTGGGCGTAAAGAGCTCGTAGGCGG
TTTGTCGCGTCTGCTGTGAAATCCCGAGGCTCAACCTCGGGCTTGCAGTGGGTACGGGCAGACTAGAGTGCGGTAGGGGAGATTGGAATTCCTGGTGTAG
CGGTGGAATGCGCAGATATCAGGAGGAACACCGATGGCGAAGGCAGATCTCTGGGCCGTAACTGACGCTGAGGAGCGAAAGCATGGGGAGCGAACAGGAT
TAGATACCCTGGTAGTCCATGCCGTAAACGTTGGGCGCTAGATGTAGGGACCTTTCCACGGTTTCTGTGTCGTAGCTAACGCATTAAGCGCCCCGCCTGG
GGAGTACGGCCGCAAGGCTAAAACTCAAAGGAATTGACGGGGGCCCGCACAAGCGGCGGAGCATGCGGATTAATTCGATGCAACGCGAAGAACCTTACCA
AGGCTTGACATACACCGGAAACGGCCAGAGATGGTCGCCCCCTTGTGGTCGGTGTACAGGTGGTGCATGGTTGTCGTCAGCTCGTGTCGTGAGATGTTGG
GTTAAGTCCCGCAACGAGCGCAACCCTCGTTCTATGTTGCCAGCGGGTTATGCCGGGGACTCATAGGAGACTGCCGGGGTCAACTCGGAGGAANGGTGGG
GATGACGTCAAATCATCATGCCCCTTATGTCTTGGGCTTCACGCATGCTACAATGGCCGGTACAAAGGGCTGCGATACCGTAAGGTGGAGCGAATCCCAA
AAAGCCGGTCTCAGTTCGGATTGAGGTCTGCAACTCGACCTCATGAAGTCGGAGTCGCTAGTAATCGCAGATCAGCAACGCTGCGGTGAATACGTTCCCG
GGCCTTGTACACACCGCCCGTCAAGTCATGAAAGTCGGTAACACTACGAAGCCGGTGGCCTAA

>EB347
GGCTACTCTTGAGTTTGATCCTGGCTCAGTGCGGCTGGATCACCTCCTTAGAGTTTGATCCCGGATCGGTGAGTAATGCCTACGAATCTGCCTGGTAGTG
GGGGACAACGTTTCGAAAGGAACGCTAATACCGCATACGTCCTACGGGAGAAAGCAGGGGACCTTCGGGCCTTGCGCTATCAGATGAGCCTAGGTCGGAT
TAGCTAGTTGGTGAGGTAATGGCTCACCAAGGCGACGATCCGTAACTGGTCTGAGAGGATGATCAGTCACACTGGAACTGAGACACGGTCCAGACTCCTA
CGGGAGGCAGCAGTGGGGAATATTGGACAATGGGCGAAAGCCTGATCCAGCCATGCCGCGTGTGTGAAGAAGGTCTTCGGATTGTAAAGCACTTTAAGTT
GGGAGGAAGGGCAGTAAGTTAATACCTTGCTGTTTTGACGTTACCGACAGAATAAGCACCGGCTAACTCTGTGCCAGCAGCCGCGGTAATACAGAGGGTG
CAAGCGTTAATCGGGAATTAACTGGGCGTAAAGCGCGCGTAGGTGGGTTCGTTAAGTTGGATGTGAAAGCCCCCGGGCTCAACCTGGGAACTGCATCCA
AAACTGGCGAGCTAGAGTACGGTAGAGGGTGGTGGAATTTCCTGTGTAGCGGTGAAATGCGTAGATATAGGAAGGAACACCAGTGGCGAAGGCGACCACC
TGGACTGATACTGACACTGAGGTGCGAAAGCGTGGGGAGCAAACAGGATTAGATACCCTGGTAGTCCACGCCGTAAACGATGTCAACTAGCCGTTGGAAT
CCTTGAGATTTTAGTGGCGCAGCTAACGCATTAAGTTGACCGCCTGGGGAGTACGGCCGCAAGGTTAAAACTCAAATGAATTGACGGGGGCCCGCACAAG
CGGTGGAGCATGTGGTTTAATTCGAAGCAACGCGAAGAACCTTACCAGGCCTTGACATGCAGAGAACTTTCCAGAGATGGATTGGTGCCTTCGGGAACT
CTGACACAGGTGCTGCATGGCTGTCGTCAGCTCGTGTCGTGAGATGTTGGGTTAAGTCCCGTAACGAGCGCAACCCTTGTCCTTAGTTACCAGCACGTTA
TGGTGGGCACTCTAAGGAGACTGCCGGTGACAAACCGGAGGAAGGTGGGGATGACGTCAAGTCATCATGGCCCTTACGGCCTGGGCTACACACGTGCTAC
AATGGTCGGTACAGAGGGTTGCCAAGCCGCGAGGTGGAGCTAATCTCACAAAACCGATCGTAGTCCGGATCGCAGTCTGCAACTCGACTGCGTGAAGTCG
GAATCGCTAGTAATCGCGAATCAGAATGTCGCGGTGAATACGTTCCCGGGCCTTGTACACACCGCCCGTCACACCATGGGAGTGGGTTGCACCAGAAGTA
GCTAGTCTAACGGACGGCCTCCTTAGAGTTTGATCCTGGCTCAGTGCGGCTGGAACACCTCCTAGAGCACTGGAGAGATACTAGC

>EB348
GACGAACGCTGGCGGCGTGCTTAACACATGCAAGTCGAACGATGATCAGGAGCTTGCTCCTGTGATTAGTGGCGAACGGGTGAGTAACACGTGAGTAACC
TGCCCCTGACTCTGGGATAAGCGTTGGAAACGACGTCTAATACTGGATATGATCACTGGTCGCATGGCCTGGTGGTGGAAAGATTTTTTGGTTGGGGATG

GACTCGCGGCCTATCAGCTTGTTGGTGAGGTAATGGCTCACCAAGGCGACGACGGGTAGCCGGCCTGAGAGGGTGACCGGCCACACTGGGACTGAGACAC
GGCCCAGACTCCTACGGGAGGCAGCAGTGGGGAATATTGCACAATGGGCGAAAGCCTGATGCAGCAACGCCGCGTGAGGGATGACGGCCTTCGGGTTGTA
AACCTCTTTTAGTAGGGAAGAAGGGGGCTTCGGTTCTTGACGGTACCTGCAGAAAAAGCACCGGCTAACTACGTGCCAGCAGCCGCGGTAATACGTAGGG
TGCAAGCGTTGTCCGGAATTATTGGGCGTAAAGAGCTCGTAGGCGGTTTGTCGCGTCTGCTGTGAAATCCCGAGGCTCAACCTCGGGCTTGCAGTGGGTA
CGGGCAGACTAGAGTGCGGTAGGGGAGATTGGAATTCCTGGTGTAGCGGTGGAATGCGCAGATATCAGGAGGAACACCGATGGCGAAGGCAGATCTCTGG
GCCGTAACTGACGCTGAGGAGCGAAAGCATGGGGAGCGAACAGGATTAGATACCCTGGTAGTCCATGCCGTAAACGTTGGGCGCTAGATGTAGGGACCTT
TCCACGGTTTCTGTGTCGTAGCTAACGCATTAAGCGCCCCGCCTGGGGAGTACGGCCGCAAGGCTAAAACTCAAAGGAATTGACGGGGGCCGCACAAGCG
GCGGAGCATGCGGATTAATTCGATGCAACGCGAAGAACCTTACCAAGGCTTGACATACACCGGAAACGGCCAGAGATGGTCGCCCCCTTGTGGTCGGTGT
ACAGGTGGTGCATGGTTGTCGTCAGCTCGTGTCGTGAGATGTTGGGTTAAGTCCCGCAACGAGCGCAACCCTCGTTCTATGTTGCCAGCGGGTTATGCCG
GGGACTCATAGGAGACTGCCGGGGTCAACTCGGAGGAAGGTGGGGATGACGTCAAATCATCATGCCCCTTATGTCTTGGGCTTCACGCATGCTACAATGG
CCGGTACAAAGGGCTGCGATACCGTAAGGTGGAGCGAATCCCAAAAAGCCGGTCTCAGTTCGGATTGAGGTCTGCAACTCGACCTCATGAAGTCGGAGTC
GCTAGTAATCGCAGATCAGCAACGCTGCGGTGAATACGTTCCCGGGCCTTGTACACACCGCCCGTCAAGTCATGAAAGTCGGTAACACCCGAAGCCGGTG
GCCTAACCCTTGTGGGAGGAGCCGTCGAAGGTGGGATCGGTGATTAGGACTAAGTCGTAACAAGGTAGCGTACCGGAAGGTGC

>EB349
GGCTGGCTCCCGTAAGGGTTACCCCACCGACTTCGGGTGTTACAAACTCTCGTGGTGTGACGGGCGGTGTGTACAAGACCCGGGAACGTATTCACCGTGG
CATGCTGATCCACGATTACTAGCGATTCCGGCTTCATGCAGGCGAGTTGCAGCCTACAATCCGAACTGGGAACGGTTTTCTGGGATTGGCTCCCCCTCGC
GGGTTTGCAGCCCTCTGTACCGTCCATTGTAGCACGTGTGTAGCCCAGGTCATAAGGGGCATGATGATTTGACGTCATCCCCACCTTCCTCCGGTTTGTC
ACCGGCAGTCACCTTAGAGTGCCCAACTAAATGATGGCAACTAAGATTAAGGGTTGCGCTCGTTGCGGGACTTAACCCAACATCTCACGACACGAGCTGA
CGACAACCATGCACCACCTGTCACCACTGTCCCCGAAGGGAAAGACATGTCTCCATGCCGGTCAGTGGGATGTCAAGACCTGGTAAGGTTCTTCGCGTTG
CTTCGAATTAAACCACATGCTCCACCGCTTGTGCGGGTCCCCGTCAATTCCTTTGAGTTTCAGCCTTGCGGCCGTACTCCCCAGGCGGAGTGCTTAATGC
GTTAGCTGCAGCACTAAGGGGCGGAAACCCCCTAACACTTAGCACTCATCGTTTACGGCGTGGACTACCAGGGTATCTAATCCTGTTTGCTCCCCACGCT
TTCGCGCCTCAGCGTCAGTTACAGACCAGAAAGCCGCCTTCGCCACTGGTGTTCCTCCACATCTCTACGCATTTCACCGCTACACGTGGAATTCCGCTTT
CCTCTTCTGTACTCAAGTCCCCCAGTTTCCAATGACCCTCCACGGTTGAGCCGTGGGCTTTCACATCAGACTTAAAGGACCGCCTGCGCGCGCTTTACGC
CCAATAATTCCGGACAACGCTTGCCACCTACGTATTACCGCGGCTGCTGGCACGTAGTTAGCCGTGGCTTTCTGACGAGGTACCGTCAAGGTACGGGCAG
TTACTCCCGTACGTGTTCTTCCCTCGCAACAGAGCTTTACGATCCGAAAACCTTCTTCACTCACGCGGCATTGCTCCATCAGACTTTCGTCCATTGTGGA
AGATTCCCTACTGCTGCCTCCCGTAGGAGTCTGGGCCGTGTCTCAGTCCCAGTGTGGCCGATCACCCTCTCAGGTCGGCTACGCATCGTTGCCTTGGTAG
GCCATTACCCCACCAACTAGCTAATGCGCCGCGGGCCCATCCTGCAGTGACAGCCGAAACCGTCTTTCAGAATTCCTTCATGCGAAGGAATTGATTATTC
GGTATTAGCCCCGGTTTCCCGGAGTTATCCCCATCTGCAGGGCAGGTTGCCCACGTGTTACTCACCCGTCCGCCGCTAATCTCAAGGAGCAAGCTCCTTA
TTTGATTCGCTCGACTTGCA

>EB350
CGGCGGACGGGTGAGTAATGCCTAGGAATCTGCCTAGTAGTGGGGGACAACGTTTCGAAAGGAACGCTAATACCGCATACGTCCTACGGGAGAAAGTGGG
GGATCTTCGGACCTCACGCTATTAGATGAGCCTAGGTCGGATTAGCTAGTTGKTAGGGTAAAGGCCTACCAAGGCGACGATCCGTAACTGGTCTGAGAGG
ATGATCAGTCACACTGGAACTGAGACACGGTCCAGACTCCTACGGGAGGCAGCAGTGGGGAATATTGGACAATGGGCGAAAGCCTGATCCAGCCATGCCG
CGTGTGTGAAGAAGGCCTTCGGGTCGTAAAGCACTTTAAGTTGGGAGGAAGGGCTCATAGCGAATACCTGTGAGTTTTGACGTTACCAACAGAATAAGCA
CCGGCTAACTTCGTGCCAGCAGCCGCGGTAATACGAAGGGTGCAAGCGTTAATCGGAATTACTGGGCGTAAAGCGCGCGTAGGTGGCTTGATAAGTTGGA
TGTGAAATCCCCGGGCTCAACCTGGGAACTGCATCCAAAACTGTCTGGCTAGAGTGCGGTAGAGGGTAGTGGAATTTCCAGTGTAGCGGTGAAATGCGTA
GATATTGGAAGGAACACCAGTGGCGAAGGCGACTACCTGGACTGACACTGACACTGAGGTGCGAAAGCGTGGGGAGCAAACAGGATTAGATACCCTGGTA
GTCCACGCCGTAAACGATGTCAACTAGCCGTTGGGATCCTTGAGATCTTAGTGGCGCAGCTAACGCATTAAGTTGACCGCCTGGGGAGTACGGCCGCAAG
GTTAAAACTCAAATGAATTGACGGGGGCCCGCACAAGCGGTGGAGCATGTGGTTTAATTCGAAGCAACGCGAAGAACCTTACCTGGCCTTGACATGCTGA
GAACTTTCCAGAGATGGATTGGTGCCTTCGGGAACTCAGACACAGGTGCTGCATGGCTGTCGTCAGCTCGTGTCGTGAGATGTTGGGTTAAGTCCCGTAA
CGAGCGCAACCCTTGTCCTTAGTTACCAGCACGTTATGGTGGGCACTCTAAGGAGACTGCCGGTGACAAACCGGAGGAAGGTGGGGATGACGTCAAGTCA
TCATGGCCCTTACGGCCAGGGCTACACACGTGCTACAATGGTCGGTACAAAGGGTTGCCAAACCGCGAGGTGGAGCTAATCCCATAAAACCGATCGTAGT
CCGGATCGCAGTCTGCAACTCGACTGCGTGAAGTCGGAATCGCTGGTAATCGTGAATCAGAACGTCACGGTGAATACGTTCCCGGGCCTTGTACACACCG
CCCGTCACACC

>EB351
CGGAGCATTGCTACCTCGTGAATTAGTGGCGAACGGGTGAGTAACACGTGAGTAACCTGCCCCTGACTCTGGGATAAGCGTTGGAAACGACGTCTAATAC
TGGATATGATCACTGGCCGCATGGTCTGGTGGTGGAAAGATTTTTTGGTTGGGGATGGACTCGCGGCCTATCAGCTTGTTGGTGAGGTAATGGCTCACCA
AGGCGACGACGGGTAGCCGGCCTGAGAGGGTGACCGGCCACACTGGGACTGAGACACGGCCCAGACTCCTACGGGAGGCAGCAGTGGGGAATATTGCACA
ATGGGCGAAAGCCTGATGCAGCAACGCCGCGTGAGGGATGACGGCCTTCGGGTTGTAAACCTCTTTTAGTAGGGAAGAAGCGAGAGTGACGGTACCTGCA
GAAAAAGCACCGGCTAACTACGTGCCAGCAGCCGCGGTAATACGTAGGGTGCAAGCGTTGTCCGGAATTATTGGGCGTAAAGAGCTCGTAGGCGGTTTGT
CGCGTCTGCTGTGAAATCCCGAGGCTCAACCTCGGGCTTGCAGTGGGTACGGGCAGACTAGAGTGCGGTAGGGGAGATTGGAATTCCTGGTGTAGCGGTG
GAATGCGCAGATATCAGGAGGAACACCGATGGCGAAGGCAGATCTCTGGGCCGTAACTGACGCTGAGGAGCGAAAGCGTGGGGAGCGAACAGGATTAGAT
ACCCTGGTAGTCCACGCCGTAAACGTTGGGCGCTAGATGTAGGGACCTTTCCACGGTTTCTGTGTCGTAGCTAACGCATTAAGCGCCCCGCCTGGGGAGT
ACGGCCGCAAGGCTAAAACTCAAAGGAATTGACGGGGGCCCGCACAAGCGGCGGAGCATGCGGATTAATTCGATGCAACGCGAAGAACCTTACCAAGGCT
TGACATACACCGGAAACGGCCAGAGATGGTCGCCCCCTTGTGGTCGGTGTACAGGTGGTGCATGGTTGTCGTCAGCTCGTGTCGTGAGATGTTGGGTTAA
GTCCCGCAACGAGCGCAACCCTCGTTCTATGTTGCCAGCGCGTTATGGCGGGGACTCATAGGAGACTGCCGGGGTCAACTCGGAGGAAGGTGGGGATGAC
GTCAAATCATCATGCCCCTTATGTCTTGGGCTTCACGCATGCTACAATGGCCGGTACAAAGGGCTGCGATACCGTAAGGTGGAGCGAATCCCAAAAAGCC
GGTCTCAGTTCGGATTGAGGTCTGCAACTCGACCTCATGAAGTCGGAGTCGCTAGTAATCGCAGATCAGCAACGCTGCGGTGAATACGTTCCCGGTCCTT
GTACACACCGCCCGTCAAGTCATGAAAGTCGGTAACA

>EB352
GTCGAGCGAACAGACGAGGAGCTTGCTCCTCTGACGTTAGCGGCGGACGGGTGAGTAACACGTGGATAACCTACCTATAAGACTGGGATAACTTCGGGAA
ACCGGAGCTAATACCGGATAACATGTTGAACCGCATGGTTCAACAGTGAAAGACGGTCTTGCTGTCACTTATAGATGGATCCGCGCCGCATTAGCTAGTT
GGTAAGGTAACGGCTTACCAAGGCAACGATGCGTAGCCGACCTGAGAGGGTGATCGGCCACACTGGAACTGAGACACGGTCCAGACTCCTACGGGAGGCA
GCAGTAGGGAATCTTCCGCAATGGGCGAAAGCCTGACGGAGCAACGCCGCGTGAGTGAAGAAGGTCTTCGGATCGTAAAACTCTGTTATTAGGGAAGAAC
AAATGTGTAAGTAACTATGCACGTCTTGACGGTACCTAATCAGAAAGCCACGGCTAACTACGTGCCAGCAGCCGCGGTAATACGTAGGTGGCAAGCGTTA
TCCGGAATTATTGGGCGTAAAGCGCGCGTAGGCGGTTTTTTAAGTCTGATGTGAAAGCCCACGGCTCAACCGTGGAGGGTCATTGGAAACTGGAAAACTT
GAGTGCAGAAGAGGAAAGTGGAATTCCATGTGTAGCGGTGAAATGCGCAGAGATATGGAGGAACACCAGTGGCGAAGGCGACTTTCTGGTCTGTAACTGA
CGCTGATGTGCGAAAGCGTGGGGATCAAACAGGATTAGATACCCTGGTAGTCCACGCCGTAAACGATGAGTGCTAAGTGTTAGGGGGTTTCCGCCCCTTA
GTGCTGCAGCTAACGCATTAAGCACTCCGCCTGGGGAGTACGACCGCAAGGTTGAAACTCAAAGGAATTGACGGGGACCCGCACAAGCGGTGGAGCATGT
GGTTTAATTCGAAGCAACGCGAAGAACCTTACCAAATCTTGACATCCTCTGATCCCTCTAGAGATAGAGTTTTCCCCTTCGGGGGACAGAGTGACAGGTG
GTGCATGGTTGTCGTCAGCTCGTGTCGTGAGATGTTGGGTTAAGTCCCGCAACGAGCGCAACCCTTAAGCTTAGTTGCCATCATTAAGTTGGGCACTCTA
AGTTGACTGCCGGTGACAAACCGGAGGAAGGTGGGGATGACGTCAAATCATCATGCCCCTTATGATTTGGGCTACACACGTGCTACAATGGACAATACAA
AGGGTAGCGAAACCGCGAGGTCAAGCAAATCCCATAAAGTTGTTCTCAGTTCGGATTGTAGTCTGCAACTCGACTACATGAAGCTGGAATCGCTAGTAAT

CGTAGATCAGCATGCTACGGTGAATACGTTCCCGGGTCTTGTACACACCGCCCGTCACACCACGAGAGTTTGTAACACCCGAAGCCGGTGGAGTAACCTT
TTGGAGCTAG

>EB353
GTCGAGCGAACAGATAAGGAGCTTGCTCCTTTGACGTTAGCGGCGGACGGGTGAGTAACACGTGGGTAACCTACCTATAAGACTGGAATAACTCCGGGAA
ACCGGGGCTAATGCCGGATAACATTTAGAACCGCATGGTTCTAAAGTGAAAGATGGTTTTGCTATCACTTATAGATGGACCCGCGCCGTATTAGCTAGTT
GGTAAGGTAACGGCTTACCAAGGCAACGATACGTAGCCGACCTGAGAGGGTGATCGGCCACACTGGAACTGAGACACGGTCCAGACTCCTACGGGAGGCA
GCAGTAGGGAATCTTCCGCAATGGGCGAAAGCCTGACGGAGCAACGCCGCGTGAGTGATGAAGGTCTTCGGATCGTAAAACTCTGTTATTAGGGAAGAAC
CAAATGTGTAAGTAACTATGCACGTCTTGACGGTACCTAATCAGAAAGCCACGGCTAACTACGTGCCAGCAGCCGCGGTAATACGTAGGTGGCAAGCGTT
ATCCGGAATTATTGGGCGTAAAGCGCGCGTAGGCGGTTTCTTAAGTCTGATGTGAAAGCCCACGGCTCAACCGTGGAGGGTCATTGGAAACTGGGAAACT
TGAGTGCAGAAGAGGAAAGTGGAATTCCATGTGTAGCGGTGAAATGCGCAGAGATATGGAGGAACACCAGTGGCGAAGGCGACTTTCTGGTCTGTAACTG
ACGCTGATGTGCGAAAGCGTGGGGATCAAACAGGATTAGATACCCTGGTAGTCCACGCCGTAAACGATGAGTGCTAAGTGTTAGGGGGTTTCCGCCCCTT
AGTGCTGCAGCTAACGCATTAAGCACTCCGCCTGGGGAGTACGACCGCAAGGTTGAAACTCAAAGGAATTGACGGGGACCCGCACAAGCGGTGGAGCATG
TGGTTTAATTCGAAGCAACGCGAAGAACCTTACCAAATCTTGACATCCTTTGACAACTCTAGAGATAGAGCCTTCCCCTTCGGGGGACAAAGTGACAGGT
GGTGCATGGTTGTCGTCAGCTCGTGTCGTGAGATGTTGGGTTAAGTCCCGCAACGAGCGCAACCCTTAAACTTAGTTGCCAGCATTTAGTTGGGCACTCT
AAGTTGACTGCCGGTGACAAACCGGAGGAAGGTGGGGATGACGTCAAATCATCATGCCCCTTATGATTTGGGCTACACACGTGCTACAATGGACAATACA
AAGGGCAGCTAAACCGCGAGGTCATGCAAATCCCATAAAGTTGTTCTCAGTTCGGATTGTAGTCTGCAACTCGACTACATGAAGCTGGAATCGCTAGTAA
TCGTAGATCAGCATGCTACGGTGAATACGTTCCCGGGTCTTGTACACACCGCCCGTCACACCACGAGAGTTTGTAACACCCGAAGCCGGTGGAGTAACCA
TTTATGGAGCTAGCCGTCGAA

>EB354
CTATACATGCAGTCGAGCGAACAGATAAGGAGCTTGCTCCTTTGACGTTAGCGGCGGACGGGTGAGTAACACGTGGGTAACCTACCTATAAGACTGGGAT
AACTTCGGGAAACCGGAGCTAATACCGGATAATATTTCGAACCGCATGGTTCGATAGTGAAAGATGGTTTTGCTATCACTTATAGATGGACCCGCGCCGT
ATTAGCTAGTTGGTAAGGTAACGGCTTACCAAGGCAACGATACGTAGCCGACCTGAGAGGGTGATCGGCCACACTGGAACTGAGACACGGTCCAGACTCC
TACGGGAGGCAGCAGTAGGGAATCTTCCGCAATGGGCGAAAGCCTGACGGAGCAACGCCGCGTGAGTGATGAAGGTCTTCGGATCGTAAAACTCTGTTAT
TAGGGAAGAACATACGTGTAAGTAACTATGCACGTCTTGACGGTACCTAATCAGAAAGCCACGGCTAACTACGTGCCAGCAGCCGCGGTAATACGTAGGT
GGCAAGCGTTATCCGGAATTATTGGGCGTAAAGCGCGCGTAGGCGGTTTTTTAAGTCTGATGTGAAAGCCCACGGCTCAACCGTGGAGGGTCATTGGAAA
CTGGAAAACTTGAGTGCAGAAGAGGAAAGTGGAATTCCATGTGTAGCGGTGAAATGCGCAGAGATATGGAGGAACACCAGTGGCGAAGGCGACTTTCTGG
TCTGTAACTGACGCTGATGTGCGAAAGCGTGGGGATCAAACAGGATTAGATACCCTGGTAGTCCACGCCGTAAACGATGAGTGCTAAGTGTTAGGGGGTT
TCCGCCCCTTAGTGCTGCAGCTAACGCATTAAGCACTCCGCCTGGGGAGTACGACCGCAAGGTTGAAACTCAAAGGAATTGACGGGGACCCGCACAAGCG
GTGGAGCATGTGGTTTAATTCGAAGCAACGCGAAGAACCTTACCAAATCTTGACATCCTTTGACCGCTCTAGAGATAGAGTTTTCCCCTTCGGGGGACAA
AGTGACAGGTGGTGCATGGTTGTCGTCAGCTCGTGTCGTGAGATGTTGGGTTAAGTCCCGCAACGAGCGCAACCCTTAAACTTAGTTGCCAGCATTGAGT
TGGGCACTCTAAGTTGACTGCCGGTGACAAACCGGAGGAAGGTGGGGATGACGTCAAATCATCATGCCCCTTATGATTTGGGCTACACACGTGCTACAAT
GGACAATACAAAGGGCAGCGAAACCGCGAGGTCAAGCAAATCCCATAAAGTTGTTCTCAGTTCGGATTGTAGTCTGCAACTCGACTACATGAAGCTGGAA
TCGCTAGTAATCGTAGATCAGCATGCTACGGTGAATACGTTCCCGGGTCTTGTACACACCGCCCGTCACACCACGAGAGTTTGTAACACCCGAAGCCGGT
GGAGTAACCATTTTGGAGCTAGCCG

>EB355
GCTTACCATGCAGTCGAACGATGATGCCCAGCTTGCTGGGTGGATTAGTGGCGAACGGGTGAGTAACACGTGAGTAACCTGCCCCTGACTCTGGGATAAG
CGTTGGAAACGACGTCTAATACTGGATATGATCACTGGCCGCATGGTCTGGTGGTGGAAAGATTTTTTGGTTGGGGATGGACTCGCGGCCTATCAGCTTG
TTGGTGAGGTAATGGCTCACCAAGGCGACGACGGGTAGCCGGCCTGAGAGGGTGACCGGCCACACTGGGACTGAGACACGGCCCAGACTCCTACGGGAGG
CAGCAGTGGGGAATATTGCACAATGGGCGAAAGCCTGATGCAGCAACGCCGCGTGAGGGATGACGGCCTTCGGGTTGTAAACCTCTTTTAGTAGGGAAGA
AGCGAAAGTGACGGTACCTGCAGAAAAAGCACCGGCTAACTACGTGCCAGCAGCCGCGGTAATACGTAGGGTGCAAGCGTTGTCCGGAATTATTGGGCGT
AAAGAGCTCGTAGGCGGTTTGTCGCGTCTGCTGTGAAATCCCGAGGCTCAACCTCGGGCTTGCAGTGGGTACGGGCAGACTAGAGTGCGGTAGGGGAGAT
TGGAATTCCTGGTGTAGCGGTGGAATGCGCAGATATCAGGAGGAACACCGATGGCGAAGGCAGATCTCTGGGCCGTAACTGACGCTGAGGAGCGAAAGCA
TGGGGGAGCGAACAGGATTAGATACCCTGGTAGTCCATGCCGTAAACGTTGGGCGCTAGATGTAGGGACCTTTCCACGGTTTCTGTGTCGTAGCTAACGC
ATTAAGCGCCCCGCCTGGGGAGTACGGCCGCAAGGCTAAAACTCAAAGGAATTGACGGGGGCCCGCACAAGCGGCGGAGCATGCGGATTAATTCGATGCA
ACGCGAAGAACCTTACCAAGGCTTGACATACACCGGAAACGGCCAGAGATGGTCGCCCCCTTGTGGTCGGTGTACAGGTGGTGCATGGTTGTCGTCAGCT
CGTGTCGTGAGATGTTGGGTTAAGTCCCGCAACGAGCGCAACCCTCGTTCTATGTTGCCAGCGGGTTATGCCGGGGACTCATAGGAGACTGCCGGGGTCA
ACTCGGAGGAAGGTGGGGATGACGTCAAATCATCATGCCCCTTATGTCTTGGGCTTCACGCATGCTACAATGGCCGGTACAAAGGGCTGCGATACCGTAA
GGTGGAGCGAATCCCAAAAAGCCGGTCTCAGTTCGGATTGAGGTCTGCAACTCGACCTCATGAAGTCGGAGTCGCTAGTAATCGCAGATCAGCAACGCTG
CGGTGAATACGTTCCCGGGCCTTGTACACACCGCCCGTCAAGTCATGAAAGTCGGTAACACCCGAAGCCGGTGGCCTAACCCTTGTGGTAA

>EB356
TGCTCAGGATGAACGCTGGCGGCGTGCCTAATACATGCAAGTCGAGCGAACAGACGAGGAGCTTGCTCCTTTGACGTTAGCGGCGGACGGGTGAGTAACA
CGTAGGTAACCTACCTATAAGACTGGGATAACTTCGGGAAACCGGAGCTAATACCGGATAATATTTCGAACCGCATGGTTCGATAGTGAAAGATGGCTTT
GCTATCACTTATAGATGGACCTGCGCCGTATTAGCTAGTTGGTAAGGTAACGGCTTACCAAGGCAACGATACGTAGCCGACCTGAGAGGGTGATCGGCCA
CACTGGAACTGAGACACGGTCCAGACTCCTACGGGAGGCAGCAGTAGGGAATCTTCCGCAATGGGCGAAAGCCTGACGGAGCAACGCCGCGTGAGTGATG
AAGGTCTTCGGATCGTAAAACTCTGTTATTAGGGAAGAACAAACGTGTAAGTAACTGTGCACGTCTTGACGGTACCTAATCAGAAAGCCACGGCTAACTA
CGTGCCAGCAGCCGCGGTAATACGTAGGTGGCAAGCGTTATCCGGAATTATTGGGCGTAAAGCGCGCGTAGGCGGTTTTTTAAGTCTGATGTGAAAGCCC
ACGGCTCAACCGTGGAGGGTCATTGGAAACTGGAAAACTTGAGTGCAGAAGAGGAAAGTGGAATTCCATGTGTAGCGGTGAAATGCGCAGAGATATGGAG
GAACACCAGTGGCGAAGGCGACTTTCTGGTCTGTAACTGACGCTGATGTGCGAAAGCGTGGGGATCAAACAGGATTAGATACCCTGGTAGTCCACGCCGT
AAACGATGAGTGCTAAGTGTTAGGGGGTTTCCGCCCCTTAGTGCTGCAGCTAACGCATTAAGCACTCCGCCTGGGGAGTACGACCGCAAGGTTGAAACTC
AAAGGAATTGACGGGGACCCGCACAAGCGGTGGAGCATGTGGTTTAATTCGAAGCAACGCGAAGAACCTTACCAAATCTTGACATCCTTTGACCCTTCTA
GAGATAGAAGTTTCCCCTTCGGGGGACAAAGTGACAGGTGGTGCATGGTTGTCGTCAGCTCGTGTCGTGAGATGTTGGGTTAAGTCCCGCAACGAGCGCA
ACCCTTAAGCTTAGTTGCCATCATTAAGTTGGGCACTCTAAGTTGACTGCCGGTGACAAACCGGAGGAAGGTGGGGGATGACGTCAAATCATCATGCCCC
TTATGATTTGGGCTACACACGTGCTACAATGGACAATACAAAGGGCAGCGAAACCGCGAGGTCAAGCAAATCCCATAAAGTTGTTCTCAGTTCGGATTGT
AGTCTGCAACTCGACTACATGAAGCTGGAATCGCTAGTAATCGTAGATCAGCATGCTACGGTGAATACGTTCCCGGGTCTTGTACACACCGCCCGTCACA
CCACGAGAGTTTGTAACACCCGAAGCCGGTGGAGTAACCATTTGGAGCTAGCCGTCGAAGGTGGGAAAATGATTGGGGTGAAT

>EB357
GCTTGCTTGGTGGATCAGTGGCGAACGGGTGAGTAACACGTGAGCAACCTGCCCTGGACTCTGGGATAAGCGCTGGAAACGGCGTCTAATACTGGATATG
AGCTCTCATCGCATGGTGGGGGTTGGAAAGATTTTTCGGTCTGGGATGGGCTCGCGGCCTATCAGCTTGTTGGTGAGGTAATGGCTCACCAAGGCGTCGA
CGGGTAGCCGGCCTGAGAGGGTGACCGGCCACACTGGGACTGAGACACGGCCCAGACTCCTACGGGAGGCAGCAGTGGGGAATATTGCACAATGGGCGGA
AGCCTGATGCAGCAACGCCGCGTGAGGGATGACGGCCTTCGGGTTGTAAACCTCTTTTAGCAGGGAAGAAGCGAGAGTGACGGTACCTGCAGAAAAAGCG
CCGGCTAACTACGTGCCAGCAGCCGCGGTAATACGTAGGGCGCAAGCGTTATCCGGAATTATTGGGCGTAAAGAGCTCGTAGGCGGTTTGTCGCGTCTGC
TGTGAAATCCCGAGGCTCAACCTCGGGCCTGCAGTGGGTACGGGCAGACTAGAGTGCGGTAGGGGAGATTGGAATTCCTGGTGTAGCGGTGGAATGCGCA
GATATCAGGAGGAACACCGATGGCGAAGGCAGATCTCTGGGCCGTAACTGACGCTGAGGAGCGAAAGGGTGGGGAGCAAACAGGCTTAGATACCCTGGTA

GTCCACCCCGTAAACGTTGGGAACTAGTTGTGGGGACCATTCCACGGTTTCCGTGACGCAGCTAACGCATTAAGTTCCCCGCCTGGGGAGTACGGCCGCA
AGGCTAAAACTCAAAGGAATTGACGGGGACCCGCACAAGCGGCGGAGCATGCGGATTAATTCGATGCAACGCGAAGAACCTTACCAAGGCTTGACATACA
CCAGAACACCGTAGAAATACGGGACTCTTTGGACACTGGTGAACAGGTGGTGCATGGTTGTCGTCAGCTCGTGTCGTGAGATGTTGGGTTAAGTCCCGCA
ACGAGCGCAACCCTCGTTCTATGTTGCCAGCACGTAATGGTGGGAACTCATGGGATACTGCCGGGGTCAACTCGGAGGAAGGTGGGGATGACGTCAAATC
ATCATGCCCCTTATGTCTTGGGCTTCACGCATGCTACAATGGCCGGTACAAAGGGCTGCAATACCGTGAGGTGGAGCGAATCCCAAAAAGCCGGTCCCAG
TTCGGATTGAGGTCTGCAACTCGACCTCATGAAGTCGGAGTCGCTAGTAATCGCAGATCAGCAACGCTGCGGTGAATACGTTCCCGGGTCTTGTACACAC
CGCCCGTCAAGTCATGAAAGTCGGTAACACCTGAAGCCGGTGGCCCAA

>EB358
GCTCAGGATGAACGCTGGCGGCGTGCTTAACACATGCAAGTCGAACGATGAAGCCCAGCTTGCTGGGTGGATTAGTGGCGAACGGGTGAGTAACACGTGA
GTAACCTGCCCTTAACTCTGGGATAAGCCTGGGAAACTGGGTCTAATACCGGATAGGAGCGCCGACCGCATGGTGGGTGTTGGAAAGATTTATCGGTTTT
GGATGGACTCGCGGCCTATCAGCTTGTTGGTGAGGTAATGGCTCACCAAGGCGACGACGGGTAGCCGGCCTGAGAGGGTGACCGGCCACACTGGGACTGA
GACACGGCCCAGACTCCTACGGGAGGCAGCAGTGGGGAATATTGCACAATGGGCGCAAGCCTGATGCAGCGACGCCGCGTGAGGGATGACGGCCTTCGGG
TTGTAAACCTCTTTCAGTAGGGAAGAAGCGAAAGTGACGGTACCTGCAGAAGAAGCACCGGCTAACTACGTGCCAGCAGCCGCGGTAATACGTAGGGTGC
GAGCGTTATCCGGAATTATTGGGCGTAAAGAGCTCGTAGGCGGTTTGTCGCGTCTGTCGTGAAAGTCCGGGGCTTAACCCCGGATCTGCGGTGGGTACGG
GCAGACTAGAGTGCAGTAGGGGAGACTGGAATTCCTGGTGTAGCGGTGGAATGCGCAGATATCAGGAGGAACACCGATGGCGAAGGCAGGTCTCTGGGCT
GTAACTGACGCTGAGGAGCGAAAGCATGGGGAGCGAACAGGATTAGATACCCTGGTAGTCCATGCCGTAAACGTTGGGCACTAGGTGTGGGGACCATTCC
ACGGTTTCCGCGCCGCAGCTAACGCATTAAGTGCCCCGCCTGGGGAGTACGGCCGCAAGGCTAAAACTCAAAGGAATTGACGGGGGCCCGCACAAGCGGC
GGAGCATGCGGATTAATTCGATGCAACGCGAAGAACCTTACCAAGGCTTGACATGTTCTCGATCGCCGTAGAGATACGGTTTCCCCTTTGGGGCGGGTTC
ACAGGTGGTGCATGGTTGTCGTCAGCTCGTGTCGTGAGATGTTGGGTTAAGTCCCGCAACGAGCGCAACCCTCGTTCCATGTTGCCAGCACGTAATGGTG
GGGACTCATGGGAGACTGCCGGGGTCAACTCGGAGGAAGGTGAGGACGACGTCAAATCATCATGCCCCTTATGTCTTGGGCTTCACGCATGCTACAATGG
CCGGTACAATGGGTCGCGATACTGTGAGGTGGAGCTAATCCCAAAAAGCCGGTCTCAGTTCGGATTGGGGTCTGCAACTCGACCCCATGAAGTCGGAGTC
GCTAGTAATCGCAGATCAGCAACGCTGCGGTGAATACGTTCCCGGGCCTTGTACACACCGCCCGTCAAGTCACGAAAGTCGGTAACACCCGAAGCCGGTG
GCCTAACCCTTGTGGGGGGGAGCCGTCCAAAGTGGGACCAGCGATTGGGACTAT

>EB359
TTCGACGGCTAGCTCCAAATGGTTACTCCACCGGCTTCGGGTGTTACAAACTCTCGTGGTGTGACGGGCGGTGTGTACAAGACCCGGGAACGTATTCACC
GTAGCATGCTGATCTACGATTACTAGCGATTCCAGCTTCATGTAGTCGAGTTGCAGACTACAATCCGAACTGAGAACAACTTTATGGGATTTGCTTGACC
TCGCGGTTTCGCTGCCCTTTGTATTGTCCATTGTAGCACGTGTGTAGCCCAAATCATAAGGGGCATGATGATTTGACGTCATCCCCACCTTCCTCCGGTT
TGTCACCGGCAGTCAACTTAGAGTGCCCAACTTAATGATGGCAACTAAGCTTAAGGGTTGCGCTCGTTGCGGGACTTAACCCAACATCTCACGACACGAG
CTGACGACAACCATGCACCACCTGTCACTTTGTCCCCCGAAGGGGAAACTTCTATCTCTAGAAGGGTCAAAGGATGTCAAGATTTGGTAAGGTTCTTCGC
GTTGCTTCGAATTAAACCACATGCTCCACCGCTTGTGCGGGTCCCCGTCAATTCCTTTGAGTTTCAACCTTGCGGTCGTACTCCCCAGGCGGAGTGCTTA
ATGCGTTAGCTGCAGCACTAAGGGGCGGAAACCCCCTAACACTTAGCACTCATCGTTTACGGCGTGGACTACCAGGGTATCTAATCCTGTTTGATCCCCA
CGCTTTCGCACATCAGCGTCAGTTACAGACCAGAAAGTCGCCTTCGCCACTGGTGTTCCTCCATATCTCTGCGCATTTCACCGCTACACATGGAATTCCA
CTTTCCTCTTCTGCACTCAAGTTTTCCAGTTTCCAATGACCCTCCACGGTTGAGCCGTGGGCTTTCACATCAGACTTAAAAAACCGCCTACGCGCGCTTT
ACGCCCAATAATTCCGGATAACGCTTGCCACCTACGTATTACCGCGGCTGCTGGCACGTAGTTAGCCGTGGCTTTCTGATTAGGTACCGTCAAGACGTGC
ACAGTTACTTACACGTTTGTTCTTCCCTAATAACAGAGTTTTACGATCCGAAGACCTTCATCACTCACGCGGCGTTGCTCCGTCAGGCTTTCGCCCATTG
CGGAAGATTCCCTACTGCTGCCTCCCGTAGGAGTCTGGACCGTGTCTCAGTTCCAGTGTGGCCGATCACCCTCTCAGGTCGGCTACGTATCGTTGCCTTG
GTAAGCCGTTACCTTACCAACTAGCTAATACGGCGCAGGTCCATCTATAAGTGATAGCAAAGCCATCTTTCACTATCGAACCATGCGGTTCGAAATATTA
TCCGGTATTAGCTCCGGTTTCCCGAAGTTATCCCAGTCTTATAGGTAGGTTACCTACGTGTTACTCACCCGTCCGCCGCTAACGTCAAAGGAGCAAGCTC
CTCGTCTGTTCGCTCGACTGCA

>EB360
GGCTAGCTCCATAAATGGTTACTCCACCGGCTTCGGGTGTTACAAACTCTCGTGGTGTGACGGGCGGTGTGTACAAGACCCGGGAACGTATTCACCGTAG
CATGCTGATCTACGATTACTAGCGATTCCAGCTTCATGTAGTCGAGTTGCAGACTACAATCCGAACTGAGAACAACTTTATGGGATTTGCATGACCTCGC
GGTTTAGCTGCCCTTTGTATTGTCCATTGTAGCACGTGTGTAGCCCAAATCATAAGGGGCATGATGATTTGACGTCATCCCCACCTTCCTCCGGTTTGTC
ACCGGCAGTCAACCTAGAGTGCCCAACTTAATGATGGCAACTAAGCTTAAGGGTTGCGCTCGTTGCGGGACTTAACCCAACATCTCACGACACGAGCTGA
CGACAACCATGCACCACCTGTCACTTTGTCCCCCGAAGGGGAAGGCTCTATCTCTAGAGTTTTCAAAGGATGTCAAGATTTGGTAAGGTTCTTCGCGTTG
CTTCGAATTAAACCACATGCTCCACCGCTTGTGCGGGTCCCCGTCAATTCCTTTGAGTTTCAACCTTGCGGTCGTACTCCCCAGGCGGAGTGCTTAATGC
GTTAGCTGCAGCACTAAGGGGCGGAAACCCCCTAACACTTAGCACTCATCGTTTACGGCGTGGACTACCAGGGTATCTAATCCTGTTTGATCCCCACGCT
TTCGCACATCAGCGTCAGTTACAGACCAGAAAGTCGCCTTCGCCACTGGTGTTCCTCCATATCTCTGCGCATTTCACCGCTACACATGGAATTCCACTTT
CCTCTTCTGCACTCAAGTTTCCCAGTTTCCAATGACCCTCCACGGTTGAGCCGTGGGCTTTCACATCAGACTTAAGAAACCGCCTACGCGCGCTTTACGC
CCAATAATTCCGGATAACGCTTGCCACCTACGTATTACCGCGGCTGCTGGCACGTAGTTAGCCGTGGCTTTCTGATTAGGTACCGTCAAGACATGCACAG
TTACTTACACGTTTGTTCTTCCCTAATAACAGAGTTTTACGAGCCGAAACCCTTCATCACTCACGCGGCGTTGCTCCGTCAGGCTTTCGCCCATTGCGGA
AGATTCCCTACTGCTGCCTCCCGTAGGAGTCTGGACCGTGTCTCAGTTCCAGTGTGGCCGATCACCCTCTCAGGTCGGCTACGTATCGTCGCCTTGGTAA
GCCGTTACCTTACCAACTAGCTAATACGGCGCGGGTCCATCTATAAGTGATAGCAAAACCATCTTTCACTTTAGAACCATGCGGTTCCAAATGTTATCCG
GTATTAGCTCCGGTTTCCCGAAGTTATCCCAGTCTTATAGGTAGGTTACCCACGTGTTACTCACCCGTCCGCCGCTAACGTCAAAGGAGCAAGCTCCTTA
TCTGTTCGCTCGACTGC

>EB361
TGCAGTCGAACGGCAGCACAGTAAGAGCTTGCTCTTATGGGTGGCGAGTGGCGGACGGGTGAGGAATACATCGGAATCTACTTTTTCGTGGGGGATAACG
TAGGGAAACTTACGCTAATACCGCATACGACCTACGGGTGAAAGCAGGGGATCTTCGGACCTTGCGCGATTGAATGAGCCGATGTCGGATTAGCTAGTTG
GCGGGGTAAAGGCCCACCAAGGCGACGATCCGTAGCTGGTCTGAGAGGATGATCAGCCACACTGGAACTGAGACACGGTCCAGACTCCTACGGGAGGCAG
CAGTGGGGAATATTGGACAATGGGCGCAAGCCTGATCCAGCCATACCGCGTGGGTGAAGAAGGCCTTCGGGTTGTAAAGCCCTTTTGTTGGGAAAGAAAT
CCAGCCGGCTAATACCTGGTTGGGATGACGGTACCCAAAGAATAAGCACCGGCTAACTTCGTGCCAGCAGCCGCGGTAATACGAAGGGTGCAAGCGTTAC
TCGGAATTACTGGGCGTAAAGCGTGCGTAGGTGGTTGTTTAAGTCTGTTGTGAAAGCCCTGGGCTCAACCTGGGAACTGCAGTGGAAACTGGACAACTAG
AGTGTGGTAGAGGGTAGCGGAATTCCCGGTGTAGCAGTGAAATGCGTAGAGATCGGGAGGAACATCCATGGCGAAGGCAGCTACCTGGACCAACACTGAC
ACTGAGGCACGAAAGCGTGGGGAGCAAACAGGATTAGATACCCTGGTAGTCCACGCCCTAAACGATGCGAACTGGATGTTGGGTGCAATTTGGCACGCAG
TATCGAAGCTAACGCGTTAAGTTCGCCGCCTGGGGAGTACGGTCGCAAGACTGAAACTCAAAGGAATTGACGGGGGCCCGCACAAGCGGTGGAGTATGTG
GTTTAATTCGATGCAACGCGAAGAACCTTACCTGGGCCTTGACATGTCGAGAACTTTCCAGAGATGGATTGGTGCCTTCGGGAACTCGAACACAGGTGCT
GCATGGCTGTCGTCAGCTCGTGTCGTGAGATGTTGGGTTAAGTCCCGCAACGAGCGCAACCCTTGTCCTTAGTTGCCAGCACGTAATGGTGGGAACTCTA
AGGAGACCGCCGGTGACAAACCGGAGGAAGGTGGGGATGACGTCAAGTCATCATGGCCCTTACGGCCAGGGCTACACACGTACTACAATGGTAGGGACAG
AGGGCTGCAAGCCGGCGACGGTAAGCCAATCCCAGAAACCCTATCTCAGTCCGGATTGGAGTCTGCAACTCGACTCCATGAAGTCGGAATCGCTAGTAAT
CGCAGATCAGCATTGCTGCGGTGAATACGTTCCCGGGCCTTGTACACACCGCCCGTCACACCATGGGAGTTTGTTGCACCAGAAGCAGGTAGCTTAACCT
TCGGGAGGGCGCT

>EB362

CGCCCTCCCGAAGGTTAAGCTACCTGCTTCTGGTGCAACAAACTCCCATGGTGTGACGGGCGGTGTGTACAAGGCCCGGGAACGTATTCACCGCAGCAAT
GCTGATCTGCGATTACTAGCGATTCCGACTTCATGGAGTCGAGTTGCAGACTCCAATCCGGACTGAGATAGGGTTTCTGGGATTGGCTTGCCCTCGCGGG
TTTGCAGCCCTCTGTCCCTACCATTGTAGTACGTGTGTAGCCCTGGTCGTAAGGGCCATGATGACTTGACGTCATCCCCACCTTCCTCCGGTTTGTCACC
GGCGGTCTCCTTAGAGTTCCCACCATTACGTGCTGGCAACTAAGGACAAGGGTTGCGCTCGTTGCGGGACTTAACCCAACATCTCACGACACGAGCTGAC
GACAGCCATGCAGCACCTGTGTTCGAGTTCCCGAAGGCACCAATCCATCTCTGGAAAGTTCTCGACATGTCAAGACCAGGTAAGGTTCTTCGCGTTGCAT
CGAATTAAACCACATACTCCACCGCTTGTGCGGGCCCCCGTCAATTCCTTTGAGTTTCAGTCTTGCGACCGTACTCCCCAGGCGGCGAACTTAACGCGTT
AGCTTCGATACTGCGTGCCAAATTGCACCCAACATCCAGTTCGCATCGTTTAGGGCGTGGACTACCAGGGTATCTAATCCTGTTTGCTCCCCACGCTTTC
GTGCCTCAGTGTCAGTGTTGGTCCAGGTAGCTGCCTTCGCCATGGATGTTCCTCCCGATCTCTACGCATTTCACTGCTACACCGGGAATTCCACTACCCT
CTACCACACTCTAGTCGCCCAGTATCCACTGCAATTCCCAGGTTGAGCCCAGGGCTTTCACAACAGACTTAAACAACCACCTACGCACGCTTTACGCCCA
GTAATTCCGAGTAACGCTTGCACCCTTCGTATTACCGCGGCTGCTGGCACGAAGTTAGCCGGTGCTTATTCTTTGGGTACCGTCAGAACAACCGGGTATT
AGCCGACTGCTTTTCTTTCCCAACAAAAGGGCTTTACAACCCGAAGGCCTTCTTCACCCACGCGGTATGGCTGGATCAGGCTTGCGCCCATTGTCCAATA
TTCCCCACTGCTGCCTCCCGTAGGAGTCTGGACCGTGTCTCAGTTCCAGTGTGGCTGATCATCCTCTCAGACCAGCTACGGATCGTCGCCTTGGTGGGCC
TTTACCCCGCCAACTAGCTAATCCGACATCGGCTCATCTATCCGCGCAAGGCCCGAAGGTCCCCTGCTTTCACCCGAAGGTCGTATGCGGTATTAGCGTA
AGTTTCCCTACGTTATCCCCCACGAAAAGGTAGATTCCGATGTATTCCTCACCCGTCCGCCACTCGCCACCCATAAGAGCAAGCTCTTACTGTGCTGCCG
TTCGACTGCATG

>EB363
GAAGGTTAAGCTACCTGCTTCTGGTGCAACAAACTCCCATGGTGTGACGGGCGGTGTGTACAAGGCCCGGGAACGTATTCACCGCAGCAATGCTGATCTG
CGATTACTAGCGATTCCGACTTCATGGAGTCGAGTTGCAGACTCCAATCCGGACTGAGATAGGGTTTCTGGGATTGGCTTGCCCTCGCGGGTTTGCAGCC
CTCTGTCCCTACCATTGTAGTACGTGTGTAGCCCTGGTCGTAAGGGCCATGATGACTTGACGTCATCCCCACCTTCCTCCGGTTTGTCACCGGCGGTCTC
CTTAGAGTTCCCACCATTACGTGCTGGCAACTAAGGACAAGGGTTGCGCTCGTTGCGGGACTTAACCCAACATCTCACGACACGAGCTGACGACAGCCAT
GCAGCACCTGTGTTCGAGTTCCCGAAGGCACCAATCCATCTCTGGAAAGTTCTCGACATGTCAAGACCAGGTAAGGTTCTTCGCGTTGCATCGAATTAAA
CCACATACTCCACCGCTTGTGCGGGCCCCCGTCAATTCCTTTGAGTTTCAGTCTTGCGACCGTACTCCCCAGGCGGCGAACTTAACGCGTTAGCTTCGAT
ACTGCGTGCCAAATTGCACCCAACATCCAGTTCGCATCGTTTAGGGCGTGGACTACCAGGGTATCTAATCCTGTTTGCTCCCCACGCTTTCGTGCCTCAG
TGTCAGTGTTGGTCCAGGTAGCTGCCTTCGCCATGGATGTTCCTCCCGATCTCTACGCATTTCACTGCTACACCGGGAATTCCACTACCCTCTACCACAC
TCTAGTCGCCCAGTATCCACTGCAATTCCCAGGTTGAGCCCAGGGCTTTCACAACAGACTTAAACAACCACCTACGCACGCTTTACGCCCAGTAATTCCG
AGTAACGCTTGCACCCTTCGTATTACCGCGGCTGCTGGCACGAAGTTAGCCGGTGCTTATTCTTTGGGTACCGTCAGAACAACCGAGTATTAATCGATTG
CTTTTCTTTCCCAACAAAAGGGCTTTACAACCCGAAGGCCTTCTTCACCCACGCGGTATGGCTGGATCAGGCTTGCGCCCATTGTCCAATATTCCCCACT
GCTGCCTCCCGTAGGAGTCTGGACCGTGTCTCAGTTCCAGTGTGGCTGATCATCCTCTCAGACCAGCTACGGATCGTCGCCTTGGTGGGCCTTTACCCCG
CCAACTAGCTAATCCGACATCGGCTCATCTATCCGCGCAAGGCCCGAAGGTCCCCTGCTTTCACCCGAAGGTCGTATGCGGTATTAGCGTAAGTTTCCCT
ACGTTATCCCCCACGAAAAGGTAGATTCCGATGTATTCCTCACCCGTCCGCCACTCGCCACCCATAAGAGCAAGCTCTTATTGTGCTGCCGTTCG

>EB364
TTAAGCTACCTGCTTCTGGTGCAACAAACTCCCATGGTGTGACGGGCGGTGTGTACAAGGCCCGGGAACGTATTCACCGCAGCAATGCTGATCTGCGATT
ACTAGCGATTCCGACTTCATGGAGTCGAGTTGCAGACTCCAATCCGGACTGAGATAGGGTTTCTGGGATTGGCTTGCCCTCGCGGGTTTGCAGCCCTCTG
TCCCTACCATTGTAGTACGTGTGTAGCCCTGGTCGTAAGGGCCATGATGACTTGACGTCATCCCCACCTTCCTCCGGTTTGTCACCGGCGGTCTCCTTAG
AGTTCCCACCATTACGTGCTGGCAACTAAGGACAAGGGTTGCGCTCGTTGCGGGACTTAACCCAACATCTCACGACACGAGCTGACGACAGCNCATGCAG
CACCTGTGNTTCGAGTTCCCGAAGGCACCAATCCATCTNCTGGAAAGTTNCTCGACATGTCAAGACNCAGGTAAGGTTCTTNCGCGTTGCATCGAATTAA
ACCACATACTCCACCGCTTGTGCGGGCCCCCCGTCAATTCCTTTGAGTTTTCAGTCTTGCGACCGTACTCCCCAGGCGGCGAACTTAACGCGTTAGCTTC
GATACTGCGTGCCAAATTGCACCCAACATCCAGTTCGCATCGTTTAGGGCGTGGACTACCAGGGTATCTAATCCTGTTTGCTCCCCACGCTTTCGTGCCT
CAGTGTCAGTGTTGGTCCAGGTAGCTGCCTTCGCCATGGATGTTCCTCCCGATCTCTACGCATTTCACTGCTACACCGGGAATTCCACTACCCTCTACCA
CACTCTAGTCGCCCAGTATCCACTGCAATTCCCAGGTTGAGCCCAGGGCTTTCACAACAGACTTAAACAACCACCTACGCACGCTTTACGCCCAGTAATT
CCGAGTAACGCTTGCACCCTTCGTATTACCGCGGCTGCTGGCACGAAGTTAGCCGGTGCTTATTCTTTGGGTACCGTCAGAACAACCGGGTATTAGCCAG
CTGCTTTTCTTTCCCAACAAAAGGGCTTTACAACCCGAAGGCCTTCTTCACCCACGCGGTATGGCTGGATCAGGCTTGCGCCCATTGTCCAATATTCCCC
ACTGCTGCCTCCCGTAGGAGTCTGGACCGTGTCTCAGTTCCAGTGTGGCTGATCATCCTCTCAGACCAGCTACGGATCGTCGCCTTGGTGGGCCTTTACC
CCGCCAACTAGCTAATCCGACATCGGCTCATCTATCCGCGCAAGGCCCGAAGGTCCCCTGCTTTCACCCGAAGGTCGTATGCGGTATTAGCGTAAGTTTC
CCTACGTTATCCCCCACGAAAAGGTAGATTCCGATGTATTCCTCACCCGTCCGCCACTCGCCACCCATAAGAGCAAGCTCTTACTGTGCTGCCGTTCGAC
TGCA

>EB365
TTTTTTTAGCTCAGGACGAACGCTGGCGGCGTGCTTAACACATGCAAGTCGAACGATGATCAGGAGCTTGCTCCTGTGATTAGTGGCGAACGGGTGAGTA
ACACGTGAGTAACCTGCCCCTGACTCTGGGATAAGCGTTGGAAACGACGTCTAATACTGGATATGACTTCCGGCCGCATGGTCTGGTTGTGGAAAGATTT
TTTGGTTGGGGATGGACTCGCGGCCTATCAGCTTGTTGGTGAGGTAATGGCTCACCAAGGCGACGACGGGTAGCCGGCCTGAGAGGGTGACCGGCCACAC
TGGGACTGAGACACGGCCCAGACTCCTACGGGAGGCAGCAGTGGGGAATATTGCACAATGGGCGAAAGCCTGATGCAGCAACGCCGCGTGAGGGATGACG
GCCTTCGGGTTGTAAACCTCTTTTAGTAGGGAAGAAGCGAAAGTGACGGTACCTGCAGAAAAAGCACCGGCTAACTACGTGCCAGCAGCCGCGGTAATAC
GTAGGGTGCAAGCGTTGTCCGGAATTATTGGGCGTAAAGAGCTCGTAGGCGGTTTGTCGCGTCTGCTGTGAAATCCCGAGGCTCAACCTCGGGCTTGCAG
TGGGTACGGGCAGACTAGAGTGCGGTAGGGGAGATTGGAATTCCTGGTGTAGCGGTGGAATGCGCAGATATCAGGAGGAACACCGATGGCGAAGGCAGAT
CTCTGGGCCGTAACTGACGCTGAGGAGCGAAAGCATGGGGAGCGAACAGGATTAGATACCCTGGTAGTCCATGCCGTAAACGTTGGGCGCTAGATGTAGG
GACCTTTCCACGGTTTCTGTGTCGTAGCTAACGCATTAAGCGCCCCGCCTGGGGAGTACGGCCGCAAGGCTAAAACTCAAAGGAATTGACGGGGGCCCGC
ACAAGCGGCGGAGCATGCGGATTAATTCGATGCAACGCGAAGAACCTTACCAAGGCTTGACATACACCGGAAACGGCCAGAGATGGTCGCCCCCTTGTGG
TCGGTGTACAGGTGGTGCATGGTTGTCGTCAGCTCGTGTCGTGAGATGTTGGGTTAAGTCCCGCAACGAGCGCAACCCTCGTTCTATGTTGCCAGCGGGT
TATGCCGGGGACTCATAGGAGACTGCCGGGGTCAACTCGGAGGAAGGTGGGGATGACGTCAAATCATCATGCCCCTTATGTCTTGGGCTTCACGCATGCT
ACAATGGCCGGTACAAAGGGCTGCGATACCGTAAGGTGGAGCGAATCCCAAAAAGCCGGTCTCAGTTCGGATTGAGGTCTGCAACTCGACCTCATGAAGT
CGGAGTCGCTAGTAATCGCAGATCAGCAACGCTGCGGTGAATACGTTCCCGGGCCTTGTACACACCGCCCGTCAAGTCATGAAAGTCGGTAACACCCGAA
GCCGGTGGCCTAACCCTTGTGGAAGGAGCCGTCGAAGGTGGGATCGGTGATTAGAATAATCGAC

>EB366
GGGTAACCTACCAATTTGACTGGGATAACTTCGGGAAACCGGAGCTAATACCGGATAACATTTGGAACCGCATGGTTCTAAAGTGAAAGATGGTTTTGCT
ATCACTTATAGATGGACCCGCGCCGTATTAGCTAGTTGGTAAGGTAACGGCTTACCAAGGCGACGATACGTAGCCGACCTGAGAGGGTGATCGGCCACAC
TGGAACTGAGACACGGTCCAGACTCCTACGGGAGGCAGCAGTAGGGAATCTTCCGCAATGGGCGAAAGCCTGACGGAGCAACGCCGCGTGAGTGATGAAG
GGTTTCGGCTCGTAAAACTCTGTTATTAGGGAAGAACAAYGTGTAAGTAACTGTGCACGTCTTGACGGTACCTAATCAGAAAGCCACGGCTAACTACGT
GCCAGCAGCCGCGGTAATACGTAGGTGGCAAGCGTTATCCGGAATTATTGGGCGTAAAGCGCGCGTAGGCGGTTTCTTAAGTCTGATGTGAAAGCCCACG
GCTCAACCGTGGAGGGTCATTGGAAACTGGGAAACTTGAGTGCAGAAGAGGAAAGTGGAATTCCATGTGTAGCGGTGAAATGCGCAGAGATATGGAGGAA
CACCAGTGGCGAAGGCGACTTTCTGGTCTGTAACTGACGCTGATGTGCGAAAGCGTGGGGATCAAACAGGATTAGATACCCTGGTAGTCCACGCCGTAAA
CGATGAGTGCTAAGTGTTAGGGGGTTTCCGCCCCTTAGTGCTGCAGCTAACGCATTAAGCACTCCGCCTGGGGAGTACGACCGCAAGGTTGAAACTCAAA
GGAATTGACGGGGACCCGCACAAGCGGTGGAGCATGTGGTTTAATTCGAAGCAACGCGAAGAACCTTACCAAATCTTGACATCCTTTGAAAACTCTAGAG
ATAGAGCCTTCCCCTTCGGGGGACAAAGTGACAGGTGGTGCATGGTTGTCGTCAGCTCGTGTCGTGAGATGTTGGGTTAAGTCCCGCAACGAGCGCAACC
CTTAAGCTTAGTTGCCATCATTAAGTTGGGCACTCTAGGTTGACTGCCGGTGACAAACCGGAGGAAGGTGGGGATGACGTCAAATCATCATGCCCCTTAT

GATTTGGGCTACACACGTGCTACAATGGACAATACAAAGGGCAGCTAAACCGCGAGGTCATGCAAATCCCATAAAGTTGTTCTCAGTTCGGATTGTAGTC
TGCAACTCGACTACATGAAGCAGGAATCGCTTGTAATCGTAGATCAGCATGGTACGGTGAATACGTTCCCGGGTCTAGTACACACCGCCCGTCACACCTC
GAGAGTTTGTAAC

>EB367
CCCACTCCCATGGTGTGACGGGCGGTGTGTACAAGGTACGGGAACGTATTCACCGCGACATTCTGATTCGCGATTACTAACGATTCCGACTTCACGCAGT
CGAGTTGCAGACTGCGATCCGGACTACGATCGGTTTTATGGGATTAGCTCCACCTCGCGGCTTGGCAACCCTCTGTACCGACCATTGTAGCACGTGTGTA
GCCCAGGCCGTAAGGGCCATGATGACTTGACGTCATCCCCACCTTCCTCCGGTTTGTCACCGGCAGTCTCCTTAGAGTGCCCACCATTACGTGCTGGTAA
CTAAGGACAAGGGTTGCGCTCGTTACGGGACTTAACCCAACATCTCACGACACGAGCTGACGACAGCCATGCAGCACCTGTCTCAATGTTCCCGAAGGCA
CCAATCTATCTCTAGAAAGTTCATTGGATGTCAAGGCCTGGTAAGGTTCTTCGCGTTGCTTCGAATTAAACCACATGCTCCACCGCTTGTGCGGGCCCCC
GTCAATTCATTTGAGTTTTAACCTTGCGGCCGTACTCCCCAGGCGGTCAACTTAATGCGTTAGCTGCGCCACTAAAAGCTCAAGGCTTCCAACGGCTAGT
TGACATCGTTTACGGCGTGGACTACCAGGGTATCTAATCCTGTTTGCTCCCCACGCTTTCGCACCTCAGTGTCAGTATTAGTCCAGGTGGTCGCCTTCGC
CACTGGTGTTCCTTCCTATATCTACGCATTTCACCGCTACACAGGAAATTCCACCACCCTCTACCATACTCTAGTCAGTCAGTTTTGAATGCAGTTCCCA
GGTTGAGCCCGGGGATTTCACATCCAACTTAACAAACCACCTACGCGCGCTTTACGCCCAGTAATTCCGATTAACGCTTGCACCCTCTGTATTACCGCGG
CTGCTGGCACAGAGTTAGCCGGTGCTTATTCTGTCGGTAACGTCAAAATTGCAGAGTATTAATCTACAACCCTTCCTCCCAACTTAAAGTGCTTTACAAT
CCGAAGACCTTCTTCACACACGCGGCATGGCTGGATCAGGCTTTCGCCCATTGTCCAATATTCCCCACTGCTGCCTCCCGTAGGAGTCTGGACCGTGTCT
CAGTTCCAGTGTGACTGATCATCCTCTCAGACCAGTTACGGATCGTCGCCTTGGTGAGCCATTACCTCACCAACTAGCTAATCCGACCTAGGCTCATCTG
ATAGCGCAAGGCCCGAAGGTCCCCTGCTTTCTCCCGTAGGACGTATGCGGTATTAGCGTCCGTTTCCGAACGTTATCCCCCACTACCAGGCAGATTCCTA
GGCATTACTCACCCGTCCGCCGCTTTCTAACGAGA

>EB368
GGTTCGGAAACGGAAGCTAATACCGCAGACGTCCTACGGGAGAAAGCAGGGGACCTTCGGGCCTTGCGCTATCAGATGAGCCTAGGTCGGATTAGCTAGT
TGGTGAGGTAATGGCTCACCAAGGCGACGATCCGTAACTGGTCTGAGAGGATGATCAGTCACACTGGAACTGAGACACGGTCCAGACTCYTACGGGAGGC
AGCAGTGGGGAATATTGGACAATGGGCGAAAGCCTGATCCAGCCATGCCGCGTGTGTGAAGAAGGTCTTCGGATTGTAAAGCACTTTAAGTTGGGAGGAA
GGGTTGTAGATTAATACTCTGCAATTTTGACGTTACCGACAGAATAAGCACCGGCTAACTCTGTGCCAGCAGCCGCGGTAATACAGAGGGTGCAAGCGTT
AATCGGAATTACTGGGCGTAAAGCGCGCGTAGGTGGTTTGTTAAGTTGGATGTGAAATCCCCGGGCTCAACCTGGGAACTGCATTCAAAACTGACTGACT
AGAGTATGGTAGAGGGTGGTGGAATTTCCTGTGTAGCGGTGAAATGCGTAGATATAGGAAGGAACACCAGTGGCGAAGGCGACCACCTGGACTAATACTG
ACACTGAGGTGCGAAAGCGTGGGGAGCAAACAGGATTAGATACCCTGGTAGTCCACGCCGTAAACGATGTCAACTAGCCGTTGGAAGCCTTGAGCTTTTA
GTGGCGCAGCTAACGCATTAAGTTGACCGCCTGGGGAGTACGGCCGCAAGGTTAAAACTCAAATGAATTGACGGGGGCCCGCACAAGCGGTGGAGCATGT
GGTTTAATTCGAAGCAACGCGAAGAACCTTACCAGGCCTTGACATCCAATGAACTTTCTAGAGATAGATTGGTGCCTTCGGGAACATTGAGACAGGTGCT
GCATGGCTGTCGTCAGCTCGTGTCGTGAGATGTTGGGTTAAGTCCCGTAACGAGCGCAACCCTTGTCCTTAGTTACCAGCACGTTATGGTGGGCACTCTA
AGGAGACTGCCGGTGACAAACCGGAGGAAGGTGGGGATGACGTCAAGTCATCATGGCCCTTACGGCCTGGGCTACACACGTGCTACAATGGTCGGTACAG
AGGGTTGCCAAGCCGCGAGGTGGAGCTAATCCCATAAAACCGATCGTAGTCCGGATCGCAGTCTGCAACTCGACTGCGTGAAGTCGGAATCGCTGGTAAT
CGCGAATCAGAATGTCGCGGTGAATACGTTCCCGGGCCTTGTACACACCGCCCGTCAC

>EB369
AACTCTACTGTTACCTTGCTAGAGGTGTGACGGGAGGAGTGAACTCGGCCCGGGAACGGATTCACCGCGGCATGCTGATCCGCGATTACAAGCAATTCCG
GCTTCATGTAGGCGAGTTGCAGCCTACAATCCGAACTGAGAATGACTTTTTGGGATTGGCTTGGCCTCGCGGCTTCGCAACCCTTTGTATCATCCATTGT
AGCACGTGTGTAGCCCAGGTCATAAGGGGCATGATGATTTGACGTCATCCCCACCTTCCTCCGGTTTGTCACCGGCAGTCACCTTAGAGTGCCCAACTGA
ATGCTGGCAACTAAGGTCAAGGGTTGCGCTCGTTGCGGGACTTAACCCAACATCTCACGACACGAGCTGACGACAACCATGCACCACCTGTCACTTTGTC
CCCCGAAGGGGAAAGCTCTATCTCTAGAGTGGTCAAAGGATGTCAAGACCTGGTAAGGTTCTTCGCGTTGCTTCGAATTAAACCACATGCTCCACTGCTT
GTGCGGGCCCCCGTCAATTCCTTTGAGTTTCAACCTTGCGGTCGTACTCCCCAGGCGGAGTGCTTAATGTGTTAACTTCAGCACTGAGGGTGGAACCCCC
CAACACCTAGCACTCATCGTTTACGGCGTGGACTACCAGGGTATCTAATCCTGTTTGCTCCCCACGCTTTCGCGCCTCAGCGTCAGTTACAGGCCAAAAA
GCCGCCTTCGCCACTGGTGTTCCTCCACATCTCTACGCATTTCACCGCTACACGTGGAATTCCACTTTTCTCTCCTGCACTCAAGTCTCCCAGTTTCCAA
TGACCCTCCACGGTTGAGCCGTGGGCTTTCACATCAGACTTAAGAGACCGCCTGCGCGCGCTTTACGCCCAATAATTCCGGATAACGCTTGCCACCTACG
TATTACCGCGGCTGCTGGCACGTAGTTAGCCGTGGCTTTCTGGTTAGGTACCGTCAAGGTACGAGCAGTTACTCTCGTACTTGTTCTTCTCTAACAACAG
AGCTTTACGACCCGAAGGCCTTCATCGCTCACGCGGCGTTGCTCGGTCAGGCTTTCGCCCATTGCCGAAGATTCCCTACTGCTGCCTCCCGTAGGAGTCT
GGGCCGTGTCTCAGTCCCAGTGTGGCCGATCACCCTCTCAGGTCGGCTACGCATCGTCGCCTTGGTGAGCCGTTACCTCACCAACTAGCTAATGCGCCGC
GGGCTCATCTGTAAGTGTCAGCCGAAACCGACTTTCAAAAAGAAGAAATGCTTCTTCTTCAATTTTCCGGTATTCGCCCCGGTGTCCCGGAGTTATATCA
TCAAACCATACGGTTACCTTGTTACGACTTAATGAGCCATAATCAAACTCTACGGTTACCTTGTTACGACTTACTGAGCCATGAT

>EB370
TCAGATTGAACGCTGGCGGCAGGCCTAACACATGCAAGTCGGACGGTAGCACAGAGGAGCTTGCTCCTCGGGTGACGAGTGGCGGACGGGTGAGTAATGT
CTGGGGATCTGCCCGATAGAGGGGGATAACCACTGGAAACGGTGGCTAATACCGCATAACGTCGCAAGACCAAAGAGGGGGACCTTCGGGCCTCTCACTA
TCGGATGAACCCAGATGGGATTAGCTAGTAGGCGGGGTAACGGCCCACCTAGGCGACGATCCCTAGCTGGTCTGAGAGGATGACCAGCCACACTGGAACT
GAGACACGGTCCAGACTCCTACGGGAGGCAGCAGTGGGGAATATTGCACAATGGGCGCAAGCCTGATGCAGCCATGCCGCGTGTATGAAGAAGGCCTTCG
GGTTGTAAAGTACTTTCAGCGGGGAGGAAGGCGATGTGGTTAATAACCGCGTCGATTGACGTTACCCGCAGAAGAAGCACCGGCTAACTCCGTGCCAGCA
GCCGCGGTAATACGGAGGGTGCAAGCGTTAATCGGAATTACTGGGCGTAAAGCGCACGCAGGCGGTCTGTTAAGTCAGATGTGAAATCCCCGGGCTTAAC
CTGGGAACTGCATTTGAAACTGGCAGGCTTGAGTCTCGTAGAGGGGGGTAGAATTCCAGGTGTAGCGGTGAAATGCGTAGAGATCTGGAGGAATACCGGT
GGCGAAGGCGGCCCCCTGGACGAAGACTGACGCTCAGGTGCGAAAGCGTGGGGAGCAAACAGGATTAGATACCCTGGTAGTCCACGCCGTAAACGATGTC
GACTTGGAGGTTGTTCCCTTGAGGAGTGGCTTCCGGAGCTAACGCGTTAAGTCGACCGCCTGGGGAGTACGGCCGCAAGGTTAAAACTCAAATGAATTGA
CGGGGGCCCGCACAAGCGGTGGAGCATGTGGTTTAATTCGATGCAACGCGAAGAACCTTACCTACTCTTGACATCCACAGAATTTGGCAGAGATGCCTTA
GTGCCTTCGGGAACTCTGAGACAGGTGCTGCATGGCTGTCGTCAGCTCGTGTTGTGAAATGTTGGGTTAAGTCCCGCAACGAGCGCAACCCTTATCCTTT
GTTGCCAGCGATTCGGTCGGGAACTCAAAGGAGACTGCCGGTGATAAACCGGAGGAAGGTGGGGATGACGTCAAGTCATCATGGCCCTTACGAGTAGGGC
TACACACGTGCTACAATGGCGCATACAAAGAGAAGCGACCTCGCGAGAGCAAGCGGACCTCATAAAGTGCGTCGTAGTCCGGATCGGAGTCTGCAACTCG
ACTCCGTGAAGTCGGAATCGCTAGTAATCGTGGATCAGAATGCCACGGTGAATACGTTCCCGGGCCTTGTACACACCGCCCGTCACACCATGGGGAGTGG
GTTGCAAAAGAAGTAGGTAGCTTAACCTTCGGGAGGGCGCTTACCACTTTGTGATTCATGACTGGGGTGAATCTA

>EB371
TGATTATGGCTCAGTAAGTCGTAACAAGGTAGCCGTATAGTTTGATTATGGCTCAGTAAGTCGTAACAAGGTAGCCGCTGCCTTGATTACTGGGATAACT
ACCGGAAACGGTAGCTAATACCGGATAATCGTTTTCTTCTCCTGAAGAGACCGGGAAAGACGGAGCAATCTGTCACTGAGGGATGGGCCTGCGGCGCATT
AGCTAGTTGGTGGGGTAACGGCTCACCAAGGCGACGATGCGTAGCCGACCTGAGAGGGTGATCGGCCACACTGGGACTGAGACACGGCCCAGAYTCCTAC
GGGAGGCAGCAGTAGGGAATCTTCCGCAATGGACGAAAGTCTGACGGAGCAACGCCGCGTGAGTGATGAAGGTTTTCGGATCGTAAAGCTCTGTTGCCAG
GGAAGAACGTCGGGTAGAGTAACTGCTATCCGAGTGACGGTACCTGAGAAGAAAGCCCCGGCTAACTACGTGCCAGCAGCCGCGGTAATACGTAGGGGGC
AAGCGTTGTCCGGAATTATTGGGCGTAAAGCGCGCGCAGGCGGTCATTTAAGTCTGGTGTTTAATCCCGAAGCTCAACTTCGGGTCGCATCGGAAACTGG
ATGACTTGAGTGCAGAAGAGGAGAGTGGAATTCCACGTGTAGCGGTGAAATGCGTAGAGATGTGGAGGAACACCAGTGGCGAAGGCGACTCTCTGGGCTG
TAACTGACGCTGAGGCGCGAAAGCGTGGGGAGCAAACAGGATTAGATACCCTGGTAGTCCACGCCGTAAACGATGAATGCTAGGTGTTAGGGGTTTCGAT
ACCCTTGGTGCCGAAGTTAACACATTAAGCATTCCGCCTGGGGAGTACGGTCGCAAGACTGAAACTCAAAGGAATTGACGGGGACCCGCACAAGCAGTGG

AGTATGTGGTTTAATTCGAAGCAACGCGAAGAACCTTACCAGGTCTTGACATCTGAATGACCGGTGCAGAGATGTACCTTTTCTTCGGAACATTCAAGAC
AGGTGGTGCATGGTTGTCGTCAGCTCGTGTCGTGAGATGTTGGGTTAAGTCCCGCAACGAGCGCAACCCTTATGCTTAGTTGCCAGCACATCATGGTGGG
CACTCTAAGCAGACTGCCGGTGACAAACCGGAGGAAGGTGGGGATGACGTCAAATCATCATGCCCCTTATGACCTGGGCTACACACGTACTACAATGGTC
GGTACAACGGGAAGCGAAGCCGCGAGGTGGAGCGAATCCTAAAAAGCCGATCTCAGTTCGGATTGCAGGCTGCAACTCGCCTGCATGAAGTCGGAATTGC
TTGTAATCGCGGATCAGCATGCCGCGGTGAATACGTTCCCGGGTCTAGTACACTCCGCCCTTCACACCTCGAGAC

>EB372
CAAGGTAGCCGTAGAGTTTGATTATGGCTCATGAAGTCGTAACGGGGTACCCGTATGGTTTGATGGTGTAACTCCGGGACACCGGGGCTAATACCGGAAG
GTTGTTTGAACCGCATGGTTCAAACATAAAAGGTGGCTTCGGCTACCACTTACAGATGGACCCGCGGCGCATTAGCTAGTTGGTGAGGTAACGGCTCACC
AAGGCAACGATGCGTAGCCGACCTGAGAGGGTGATCGGCCACACTGGGACTGAGACACGGCCCAGACTCCTACGGGAGGCAGCAGTAGGGAATCTTCCGC
AATGGACGAAAGTCTGACGGAGCAACGCCGCGTGAGTGATGAAGGTTTTCGGATCGTAAAGCTCTGTTGTTAGGGAAGAACAAGTACCGTTCGAATAGGG
CGGTACCTTGACGGTACCTAACCAGAAAGCCACGGCTAACTACGTGCCAGCAGCCGCGGTAATACGTAGGTGGCAAGCGTTGTCCGGAATTATTGGGCGT
AAAGGGCTCGCAGGCGGTTTCTTAAGTCTGATGTGAAAGCCCCCGGCTCAACCGGGGAGGGTCATTGGAAACTGGGGAACTTGAGTGCAGAAGAGGAGAG
TGGAATTCCACGTGTAGCGGTGAAATGCGTAGAGATGTGGAGGAACACCAGTGGCGAAGGCGACTCTCTGGTCTGTAACTGACGCTGAGGAGCGAAAGCG
TGGGGAGCGAACAGGATTAGATACCCTGGTAGTCCACGCCGTAAACGATGAGTGCTAAGTGTTAGGGGGTTTCCGCCCCTTAGTGCTGCAGCTAACGCAT
TAAGCACTCCGCCTGGGGAGTACGGTCGCAAGACTGAAACTCAAAGGAATTGACGGGGGCCCGCACAAGCGGTGGAGCATGTGGTTTAATTCGAAGCAAC
GCGAAGAACCTTACCAGGTCTTGACATCCTCTGACAATCCTAGAGATAGGACGTCCCCTTCGGGGGCAGAGTGACAGGTGGTGCATGGTTGTCGTCAGCT
CGTGTCGTGAGATGTTGGGTTAAGTCCCGCAACGAGCGCAACCCTTGATCTTAGTTGCCAGCATTCAGTTGGGCACTCTAAGGTGACTGCCGGTGACAAA
CCGGAGGAAGGTGGGGATGACGTCAAATCATCATGCCCCTTATGACCTGGGCTACACACGTGCTACAATGGACAGAACAAAGGGCAGCGAAACCGCGAGG
TTAAGCCAATCCCACAAATCTGTTCTCAGTTCGGATCGCGGTCTGCAACTCGAATGCGTGAAGCAGGAATCGGTAGTAATCGCGGATCAGCCTGCCGCGG
GGAATACGTTCCCGGTCCTTGTACACCCCGCCTCTCTC

>EB373
CGAGCGGTACTTGCATGAGAGAGCTTGCTCTCCTAGATGGTTAGCGGCGGACGGGTGAGTAACACGTAGGCAACCTGCCCTCTGGACTGGGATAACTACC
GGAAACGGTAGCTAATACCAGATAATTCACTTCTTCGCATGGAGAAGTGAGGAAAGACGGAGCAATCTGTCACCGGAGGATGGGCCTGCGGCGCATTAGC
TAGTTGGAGAGGTAACGGCTCCCCAAGGCGACGATGCGTAGCCGACCTGAGAGGGTGATCGGCCACACTGGGACTGAGACACGGCCCAGACTCCTACGGG
AGGCAGCAGTAGGGAATCTTCCGCAATGGGCGAAAGCCTGACGGAGCAACGCCGCGTGAGTGATGAAGGTTTTCGGATCGTAAAGCTCTGTTGCCAAGGA
AGAACGTCCTTAAGAGTAACTGCTTAAGGAGTGACGGTACTTGAGAAGAAAGCCCCGGCTAACTACGTGCCAGCAGCCGCGGTAATACGTAGGGGGCAAG
CGTTGTCCGGAATTATTGGGCGTAAAGCGCGCGCAGGCGGTCATTTAAGTCTGGTGTTTAAGCCCGGGGCTCAACCCCGGATCGCACGGGAAACTGGATG
ACTTGAGTGCAGAAGAGGAGAGTGGAATTCCACGTGTAGCGGTGAAATGCGTAGAGATGTGGAGGAACACCAGTGGCGAAGGCGACTCTCTGGGCTGTAA
CTGACGCTGAGGCGCGAAAGCGTGGGGAGCAAACAGGATTAGATACCCTGGTAGTCCACGCCGTAAACGATGAGTGCTAGGTGTTAGGGGTTTCGATACC
CTTGGTGCCGAAGTTAACACATTAAGCACTCCGCCTGGGGAGTACGGTCGCAAGACTGAAACTCAAAGGAATTGACGGGGACCCGCACAAGCAGTGGAGT
ATGTGGTTTAATTCGAAGCAACGCGAAGAACCTTACCAGGTCTTGACATCTGAATGACCGGTACAGAGATGTACCTTTCCTTCGGGACATTCAAGACAGG
TGGTGCATGGTTGTCGTCAGCTCGTGTCGTGAGATGTTGGGTTAAGTCCCGCAACGAGCGCAACCCTTGACCTTAGTTGCCAGCACTTCGGATGGGCACT
CTAGGGTGACTGCCGGTGACAAACCGGAGGAAGGTGGGGATGACGTCAAATCATCATGCCCCTTATGACCTGGGCTACACACGTACTACAATGGCTGGTA
CAACGGGAAGCGAAGCCGCGAGGTGGAGCCAATCCTAAAAAGCCAGTCTCAGTTCGGATTGCAGGCTGCAACTCGCCTGCATGAAGTCGGAATTGCTAGT
AATCGCGGATCAGCATGCCGCGGTGAATACGTTCCCGGGTCTTGTACACACCGCCCGTCACACCACGAGAGTTTACAACATCCCGAAGTCGGTGGGGTAA
CCGCAAGGAGCCAGCCGCCG

>EB374
AGTTTGACTGGGATACCTTCGGGATCCGGAGCTAATACCGGATAACATTTGGAACCGCATGGTTCTAAAGTGAAAGATGGTTTTGCTATCACTTATAGAT
GGACCCGCGCCGTATTAGCTAGTTGGTAAGGTAACGGCTTACCAAGGCGACGATACGTAGCCGACCTGAGAGGGTGATCGGCCACACTGGAACTGAGACA
CGGTCCAGACTCCTACGGGAGGCAGCAGTAGGGAATCTTCCGCAATGGGCGAAAGCCTGACGGAGCAACGCCGCGTGAGTGATGAAGGGTTTCGGCTCGT
AAAACTCTGTTATTAGGGAAGAACAAATGTGTAAGTAACTATGCACATCTTGACGGTACCTAATCAGAAAGCCACGGCTAACTACGTGCCAGCAGCCGCG
GTAATACGTAGGTGGCAAGCGTTATCCGGAATTATTGGGCGTAAAGCGCGCGTAGGCGGTTTCTTAAGTCTGATGTGAAAGCCCACGGCTCAACCGTGGA
GGGTCATTGGAAACTGGGAAACTTGAGTGCAGAAGAGGAAAGTGGAATTCCATGTGTAGCGGTGAAATGCGCAGAGATATGGAGGAACACCAGTGGCGAA
GGCGACTTTCTGGTCTGTAACTGACGCTGATGTGCGAAAGCGTGGGGNATCAAACAGGATTAGATACCCTGGTAGTCCACGCCGTAAACGATGAGTGCTA
AGTGTTAGGGGGTTTCCGCCCCTTAGTGCTGCAGCTAACGCATTAAGCACTCCGCCTGGGGAGTACGACCGCAAGGTTGAAACTCAAAGGAATTGACGGG
GACCCGCACAAGCGGTGGAGCATGTGGTTTAATTCGAAGCAACGCGAAGAACCTTACCAAATCTTGACATCCTTTGAAAACTCTAGAGATAGAGCCTTCC
CCTTCGGGGGACAAAGTGACAGGTGGTGCATGGTTGTCGTCAGCTCGTGTCGTGAGATGTTGGGTTAAGTCCCGCAACGAGCGCAACCCTTAAGCTTAGT
TGCCATCATTAAGTTGGGCACTCTAGGTTGACTGCCGGTGACAAACCGGAGGAAGGTGGGGATGACGTCAAATCATCATGCCCCTTATGATTTGGGCTAC
ACACGTGCTACAATGGACAATACAAAGGGCAGCTAAACCGCGAGGTCATGCAAATCCCATAAAGTTGTTCTCAGTTCGGATTGTAGTCTGCAACTCGACT
ACATGAAGCAGGAATCGCTTGTAATCGTAGATCAGCATGGTACGGTGAATACGTTCCCGGGTCTAGTACACACCGCCCTTCACACCTCG

>EB375
TGCAGTCGAACGGCAGCACAGAGGAGCTTGCTCCTTGGGTGGCGAGTGGCGGACGGGTGAGGAATACATCGGAATCTGCCTATTTGTGGGGGATAACGTA
GGGAAACTTACGCTAATACCGCATACGACCTACGGGTGAAAGCGGAGGACCTTCGGGCTTCGCGCAGATAGATGAGCCGATGTCGGATTAGCTAGTTGGC
GGGGTAAAGGCCCACCAAGGCGACGATCCGTAGCTGGTCTGAGAGGATGATCAGCCACACTGGAACTGAGACACGGTCCAGACTCCTACGGGAGGCAGCA
GTGGGGAATATTGGACAATGGGCGCAAGCCTGATCCAGCCATGCCGCGTGGGTGAAGAAGGCCTTCGGGTTGTAAAGCCCTTTTGTTGGGAAAGAAAAGC
AGTCGGTTAATACCCGATTGTTCTGACGGTACCCCAAAGAATAAGCACCGGCTAACTTCGTGCCAGCAGCCGCGGTAATACGAAGGGTGCAAGCGTTACT
CGGAATTACTGGGCGTAAAGCGTGCGTAGGTGGTTTGTTAAGTCTGATGTGAAAGCCCTGGGCTCAACCTGGGAATTGCATTGGATACTGGCAGGCTAGA
GTGCGGTAGAGGGTAGTGGAATTCCCGGTGTAGCAGTGAAATGCGTAGAGATCGGGAGGAACATCCGTGGCGAAGGCGACTACCTGGACCAGCACTGACA
CTGAGGCACGAAAGCGTGGGGAGCAAACAGGATTAGATACCCTGGTAGTCCACGCCCTAAACGATGCGAACTGGATGTTGGGTTCAATTAGGAACTCAGT
ATCGAAGCTAACGCGTTAAGTTCGCCGCCTGGGGAGTACGGTCGCAAGACTGAAACTCAAAGGAATTGACGGGGGCCCGCACAAGCGGTGGAGTATGTGG
TTTAATTCGATGCAACGCGAAGAACCTTACCTGGCCTTGACATGTCGAGAACTTTCCAGAGATGGATTGGTGCCTTCGGGAACTCGAACACAGGTGCTGC
ATGGCTGTCGTCAGCTCGTGTCGTGAGATGTTGGGTTAAGTCCCGCAACGAGCGCAACCCTTGTCCTTAGTTGCCAGCACGTAATGGTGGGAACTCTAA
GGAGACCGCCGGTGACAAACCGGAGGAAGGTGGGGATGACGTCAAGTCATCATGGCCCTTACGGCCAGGGCTACACACGTACTACAATGGGAAGGACAGA
GGGCTGCAAACCCGCGAGGGCAAGCCAATCCCAGAAACCCTTCTCTCAGTCCGGATCGGAGTCTGCAACTCGACTCCGTGAAGTCGGAATCGCTAGTAATC
GCAGATCAGCATTGCTGCGGTGAATACGTTCCCGGGCCTTGTACACACCGCCCGTCACACCATGGGAGTTTGTTGCACCAGAAGCAGGTAGCTTAACCTT
CGGGAGGGCGCT

>EB376
GTAGGAGCTTGCTCCTTGGGTGGCGAGTGGCGGACGGGTGAGGAATACATCGGAATCTGCCTATTTGTGGGGGATAACGTAGGGAAACTTACGCTAATAC
CGCATACGACCTACGGGTGAAAGCGGAGGACCTTCGGGCTTCGCGCAGATAGATGAGCCGATGTCGGATTAGCTAGTTGGCGGGGTAAAGGCCCACCAAG
GCGACGATCCGTAGCTGGTCTGAGAGGATGATCAGCCACACTGGAACTGAGACACGGTCCAGACTCCTACGGGAGGCAGCAGTGGGGAATATTGGACAAT
GGGCGCAAGCCTGATCCAGCCATGCCGCGTGGGTGAAGAAGGCCTTCGGGTTGTAAAGCCCTTTTGTTGGGAAAGAAAAGCAGTCGGTTAATACCCGATT
GTTCTGACGGTACCCAAAGAATAAGCACCGGCTAACTTCGTGCCAGCAGCCGCGGTAATACGAAGGGTGCAAGCGTTACTCGGAATTACTGGGCGTAAAG
CGTGCGTAGGTGGTTTGTTAAGTCTGATGTGAAAGCCCTGGGCTCAACCTGGGAATTGCATTGGATACTGGCAGGCTAGAGTGCGGTAGAGGGTAGTGGA

ATTCCCGGTGTAGCAGTGAAATGCGTAGAGATCGGGAGGAACATCCGTGGCGAAGGCGACTACCTGGACCAGCACTGACACTGAGGCACGAAAGCGTGGG
GAGCAAACAGGATTAGATACCCTGGTAGTCCACGCCCTAAACGATGCGAACTGGATGTTGGGTTCAACTAGGAACTCAGTATCGAAGCTAACGCGTTAAG
TTCGCCGCCTGGGGAGTACGGTCGCAAGACTGAAACTCAAAGGAATTGACGGGGGCCCGCACAAGCGGTGGAGTATGTGGTTTAATTCGATGCAACGCGA
AGAACCTTACCTGGCCTTGACATGCACGGAACTTTCCAGAGATGGATTGGTGCCTTCGGGAACCGTGACACAGGTGCTGCATGGCTGTCGTCAGCTCGTG
TCGTGAGATGTTGGGTTAAGTCCCGCAACGAGCGCNAACCCTTGTCCTTAGTTGCCAGCACGTAATGGTGGGAACTCTAAGGAGACCGCCGGTGACAAAC
CGGAGGAAGGTGGGGATGACGTCAAGTCATCATGGCCCTTACGGCCAGGGCTACACACGTACTACAATGGGAAGGACAGAGGGCTGCAAACCCGCGAGGG
CAAGCCAATCCCAGAAACCTTCTCTCAGTCCGGATCGGAGTCTGCAACTCGACTCCGTGAAGTCGGAATCGCTAGTAATCGCAGATCAGCATTGCTGCGG
TGAATACGTTCCCGGGCCTTGTACACACCGCCCGTCACACCATGGGAGTTTGTTGCTCCAGAAGCAGGTAGCTTAA

>EB377
TCCTTGCGGTTAGCTACCTACTTCTGGAAAAACCCGCTCCCATGGTGTGACGGGCGGTGTGTACAAGACCCGGGAACGTATTCACCGCGGCATGCTGATC
CGCGATTACTAGCGATTCCAACTTCACGTAGTCGAGTTGCAGACTACGATCCGGACTACGATACACTTTCTGGGATTAGCTCCCCCTCGCGGGTTGGCGG
CCCTCTGTATGTACCATTGTATGACGTGTGAAGCCCTACCCATAAGGGCCATGAGGACTTGACGTCATCCCCACCTTCCTCCGGTTTGTCACCGGCAGTC
TCATTAGAGTGCTCTTGCGTAGCAACTAATGACAAGGGTTGCGCTCGTTGCGGGACTTAACCCAACATCTCACGACACGAGCTGACGACAGCCATGCAGC
ACCTGTGTTCAGGTTCCCTTTCGGGCACWRCCAAATNCTCTYCGGTCTTCCTGACATGTCAAGGGTAGGTAAGGTTTTTCGCGTTGCATCGAATTAATCC
ACATCATCCACCGCTTGTGCGGGTCCCCGTCAATTCCTTTGAGTTTTAATCTTGCGACCGTACTNCCCCAGGCGGTCTACTTCACGCGTTAGCTGCGTTA
CCAAGTTAATTAAAACCCGACAACTAGTAGACATCGTTTAGGGCGTGGACTACCAGGGTATCTAATCCTGTTTGCTCCCCACGCTTTCGTGCATGAGCGT
CAATCTTGACCCAGGGGGCTGCCTTCGCCATCGGTGTTCCTCCACATCTCTACGCATTTCACTGCTACACGTGGAATTCTACCCCCCTCTGCCAGATTCT
AGCCTTGCAGTCTCCATCGCAATTCCCAGGTTGAGCCCGGGGATTTCACGACAGACTTACAAAACCGCCTGCGCACGCTTTACGCCCAGTAATTCCGATT
AACGCTTGCACCCTACGTATTACCGCGGCTGCTGGCACGTAGTTAGCCGGTGCTTATTCTTCAGGTACCGTCATTAGCCGTGGATATTAGCCACAACCGT
TTCTTCCCTGACAAAAGAGCTTTACAACCCGAAGGCCTTCTTCACTCACGCGGCATTGCTGGATCAGGCTTGCGCCCATTGTCCAAAATTCCCCACTGCT
GCCTCCCGTAGGAGTCTGGACCGTGTCTCAGTTCCAGTGTGGCTGGTCGTCCTCTCAGACCAGCTACTGATCGTCGCCTTGGTGAGCCTTTACCTCACCA
ACTAGCTAATCAGATATCGGCCGCTCCAGGAGCACAAGGCCCGAAGGTCCCCTGCTTTCATCCATAGATCGTATGCGGTATTAGCGTAACTTTCGCTACG
TTATCCCCCACTCTTGGGTACGTTCCGATATATTACTCACCCGTTCGCCACTCGTCACGCGGTAGCAAGCTCCGCATGTTACCG

>EB378
ATGCAAGTCGAACGGTGAAGCCCAGCTTGCTGGGTGGATCAGTGGCGAACGGGTGAGTAACACGTGAGCAATCTGCCCCTGACTCTGGGATAAGCGCTGG
AAACGGCGTCTAATACTGGATATGAACCGTGGAGGCATCTTCAACGGTTGGAAAGATTTTTTGGTCAGGGATGAGCTCGCGGCCTATCAGCTTGTTGGTG
AGGTAATGGCTCACCAAGGCGTCGACGGGTAGCCGGCCTGAGAGGGTGACCGGCCACACTGGGACTGAGACACGGCCCAGACTCCTACGGGAGGCAGCAG
TGGGGAATATTGCACAATGGGCGCAAGCCTGATGCAGCAACGCCGCGTGAGGGATGACGGCCTTCGGGTTGTAAACCTCTTTTAGCAGGGAAGAAGCGAA
AGTGACGGTACCTGCAGAAAAAGCGCCGGCTAACTACGTGCCAGCAGCCGCGGTAATACGTAGGGCGCAAGCGTTATCCGGAATTATTGGGCGTAAAGAG
CTCGTAGGCGGTTTGTCGCGTCTGCTGTGAAATCCCGAGGCAACCTCGGGTCTGCAGTGGGTACGGGCAGACTAGAGTGCGGTAGGGGAGATTGGAATTC
CTGGTGTAGCGGTGGAATGCGCAGATATCAGGAGGAACACCGATGGCGAAGGCAGATCTCTGGGCCGTAACTGACGCTGAGGAGCGAAAGGGTGGGGAGC
AAACAGGCTTAGATACCCTGGTAGTCCACCCCGTAAACGTTGGGAACTAGTTGTGGGGTCCATTCCACGGATTCCGTGACGCAGCTAACGCATTAAGTTC
CCCGCCTGGGGAGTACGGCCGCAAGGCTAAAACTCAAAGGAATTGACGGGGACCCGCACAAGCGGCGGAGCATGCGGATTAATTCGATGCAACGCGAAGA
ACCTTACCAAGGCTTGACATATACGAGAACGCTGCAGAAATGTAGAACTCTTTGGACACTCGTAAACAGGTGGTGCATGGTTGTCGTCAGCTCGTGTCGT
GAGATGTTGGGTTAAGTCCCGCAACGAGCGCAACCCTCGTTCTATGTTGCCAGCACGTAATGGTGGGAACTCATGGGATACTGCCGGGGTCAACTCGGAG
GAAGGTGGGGATGACGTCAAATCATCATGCCCCTTATGTCTTGGGCTTCACGCATGCTACAATGGCCGGTACAAAGGGCTGCAATACCGTAAGGTGGAGC
GAATCCCAAAAAGCCGGTCCCAGTTCGGATTGAGGTCTGCAACTCGACCTCATGAAGTCGGAGTCGCTAGTAATCGCAGATCAGCAACGCTGCGGTGAAT
ACGTTCCCGGGTCTTGTACACACCGCCCGTCAAGTCATGAAAGTCGGTAACACCTGAAGCCGGTGGCCCAACCCTTGTGGAGGGAGCC

>EB379
ATGCAAGTCGAACGGTGAAGCCCAGCTTGCTGGGTGGATCAGTGGCGAACGGGTGAGTAACACGTGAGCAATCTGCCCCTGACTCTGGGATAAGCGCTGG
AAACGGCGTCTAATACTGGATATGAACCGTGGAGGCATCTTCAACGGTTGGAAAGATTTTTTGGTCAGGGATGAGCTCGCGGCCTATCAGCTTGTTGGTG
AGGTAATGGCTCACCAAGGCGTCGACGGGTAGCCGGCCTGAGAGGGTGACCGGCCACACTGGGACTGAGACACGGCCCAGACTCCTACGGGAGGCAGCAG
TGGGGAATATTGCACAATGGGCGCAAGCCTGATGCAGCAACGCCGCGTGAGGGATGACGGCCTTCGGGTTGTAAACCTCTTTTAGCAGGGAAGAAGCGAA
AGTGACGGTACCTGCAGAAAAAGCGCCGGCTAACTACGTGCCAGCAGCCGCGGTAATACGTAGGGCGCAAGCGTTATCCGGAATTATTGGGCGTAAAGAG
CTCGTAGGCGGTTTGTCGCGTCTGCTGTGAAATCCCGAGGCTCAACCTCGGGTCTGCAGTGGGTACGGGCAGACTAGAGTGCGGTAGGGGAGATTGGAAT
TCCTGGTGTAGCGGTGGAATGCGCAGATATCAGGAGGAACACCGATGGCGAAGGCAGATCTCTGGGCCGTAACTGACGCTGAGGAGCGAAAGGGTGGGGA
GCAAACAGGCTTAGATACCCTGGTAGTCCACCCCGTAAACGTTGGGAACTAGTTGTGGGGTCCATTCCACGGATTCCGTGACGCAGCTAACGCATTAAGT
TCCCCGCCTGGGGAGTACGGCCGCAAGGCTAAAACTCAAAGGAATTGACGGGGACCCGCACAAGCGGCGGAGCATGCGGATTAATTCGATGCAACGCGAA
GAACCTTACCAAGGCTTGACATATACGAGAACGCTGCAGAAATGTAGAACTCTTTGGACACTCGTAAACAGGTGGTGCATGGTTGTCGTCAGCTCGTGTC
GTGAGATGTTGGGTTAAGTCCCGCAACGAGCGCAACCCTCGTTCTATGTTGCCAGCACGTAATGGTGGGAACTCATGGGATACTGCCGGGGTCAACTCGG
AGGAAGGTGGGGATGACGTCAAATCATCATGCCCCTTATGTCTTGGGCTTCACGCATGCTACAATGGCCGGTACAAAGGGCTGCAATACCGTAAGGTGGA
GCGAATCCCAAAAAGCCGGTCCCAGTTCGGATTGAGGTCTGCAACTCGACCTCATGAAGTCGGAGTCGCTAGTAATCGCAGATCAGCAACGCTGCGGTGA
ATACGTTCCCGGGTCTTGTACACACCGCCCGTCAAGTCATGAAAGTCGGTAACACCTGAAGCCGGTGGCCCAACCCTTGTGGAGGGAGCC

>EB380
TATTTGCGGCGTGCTTACCATGCAGTCGAACGGTGAAGCCCAGCTTGCTGGGTGGATCAGTGGCGAACGGGTGAGTAACACGTGAGCAACCTGCCCCGGA
CTCTGGGATAAGCGCTGGAAACGGCGTCTAATACTGGATATGAGCTGCAACCGCATGGTTAGCAGTTGGAAAGATTTTTCGGTCTGGGATGGGCTCGCGG
CCTATCAGCTTGTTGGTGAGGTAATGGCTCACCAAGGCGTCGACGGGTAGCCGGCCTGAGAGGGTGACCGGCCACACTGGGACTGAGACACGGCCCAGAC
TCCTACGGGAGGCAGCAGTGGGGAATATTGCACAATGGGCGGAAGCCTGATGCAGCAACGCCGCGTGAGGGACGACGGCCTTCGGGTTGTAAACCTCTTT
TAGCAAGGAAGAAGCGAAAGTGACGGTACTTGCAGAAAAAGCGCCGGCTAACTACGTGCCAGCAGCCGCGGTAATACGTAGGGCGCAAGCGTTATCCGGA
ATTATTGGGCGTAAAGAGCTCGTAGGCGGTTTGTCGCGTCTGCTGTGAAATCCCGAGGCTCAACCTCGGGTCTGCAGTGGGTACGGGCAGACTAGAGTGC
GGTAGGGGAGATTGGAATTCCTGGTGTAGCGGTGGAATGCGCAGATATCAGGAGGAACACCGATGGCGAAGGCAGATCTCTGGGCCGTAACTGACGCTGA
GGAGCGAAAGGGTGGGGAGCAAACAGGCTTAGATACCCTGGTAGTCCACCCCGTAAACGTTGGGAACTAGTTGTGGGGTCCATTCCACGGATTCCGTGAC
GCAGCTAACGCATTAAGTTCCCCGCCTGGGGAGTACGGCCGCAAGGCTAAAACTCAAAGGAATTGACGGGGACCCGCACAAGCGGCGGAGCATGCGGATT
AATTCGATGCAACGCGAAGAACCTTACCAAGGCTTGACATACACGAGAACGCTGCAGAAATGTAGAACTCTTTGGACACTCGTGAACAGGTGGTGCATGG
TTGTCGTCAGCTCGTGTCGTGAGATGTTGGGTTAAGTCCCGCAACGAGCGCAACCCTCGTTCTATGTTGCCAGCACGTAATGGTGGGAACTCATGGGATA
CTGCCGGGGTCAACTCGGAGGAAGGTGGGGATGACGTCAAATCATCATGCCCCTTATGTCTTGGGCTTCACGCATGCTACAATGGCCGGTACAAAGGGCT
GCAATACCGTAAGGTGGAGCGAATCCCAAAAAGCCGGTCCCAGTTCGGATTGAGGTCTGCAACTCGACCTCATGAAGTCGGAGTCGCTAGTAATCGCAGA
TCAGCAACGCTGCGGTGAATACGTTCCCGGGTCTTGTACACACCGCCCGTCAAGTCATGAAAGTCGGTAACACCTGAAGCCGGTGGCCCAACCCTTGTGG
AGGGAGCCGTCGAAGGTGATCCT

>EB381
GCTCAGGATGAACGCTGGCGGCGTGCTTAACACATGCAAGTCGAACGGTGAAGGAGAGCTTGCTCTTTGGATCAGTGGCGAACGGGTGAGTAACACGTGA
GCAATCTGCCCCTGACTCTGGGATAACAGTTGGAAACAGCTGCTAATACCGGATACGAGACGTGACCGCATGGTCGACGTTTGGAAAGAATTTCGGTCAG
GGATGAGCTCGCGGCCTATCAGCTTGTTGGTGAGGTAATGGCTCACCAAGGCGTCGACGGGTAGCCGGCCTGAGAGGGTGACCGGCCACACTGGGACTGA

GACACGGCCCAGACTCCTACGGGAGGCAGCAGTGGGGAATATTGCACAATGGGCGAAAGCCTGATGCAGCAACGCCGCGTGAGGGACGACGGCCTTCGGG
TTGTAAACCTCTTTTAGCAGGGAAGAAGCGAAAGTGACGGTACCTGCAGAAAAAGCGCCGGCTAACTACGTGCCAGCAGCCGCGGTAATACGTAGGGCGC
AAGCGTTATCCGGAATTATTGGGCGTAAAGAGCTCGTAGGCGGTTTGTCGCGTCTGCTGTGAAAACTGGAGGCTCAACCTCCAGCCTGCAGTGGGTACGG
GCAGACTAGAGTGCGGTAGGGGAGATTGGAATTCCTGGTGTAGCGGTGGAATGCGCAGATATCAGGAGGAACACCGATGGCGAAGGCAGATCTCTGGGCC
GTAACTGACGCTGAGGAGCGAAAGGGTGGGGAGCAAACAGGCTTAGATACCCTGGTAGTCCACCCCGTAAACGTTGGGAACTAGTTGTGGGGTCCATTCC
ACGGATTCCGTGACGCAGCTAACGCATTAAGTTCCCCGCCTGGGGAGTACGGCCGCAAGGCTAAAACTCAAAGGAATTGACGGGGACCCGCACAAGCGGC
GGAGCATGCGGATTAATTCGATGCAACGCGAAGAACCTTACCAAGGCTTGACATATACGAGAACGGGCTAGAAATAGTCAACTCTTTGGACACTCGTAAA
CAGGTGGTGCATGGTTGTCGTCAGCTCGTGTCGTGAGATGTTGGGTTAAGTCCCGCAACGAGCGCAACCCTCGTTCTATGTTGCCAGCACGTAATGGTGG
GAACTCATGGGATACTGCCGGGGTCAACTCGGAGGAAGGTGGGGATGACGTCAAATCATCATGCCCCTTATGTCTTGGGCTTCACGCATGCTACAATGGC
CGGTACAAAGGGCTGCAATACCGTAAGGTGGAGCGAATCCCAAAAAGCCGGTCCCAGTTCGGATTGAGGTCTGCAACTCGACCTCATGAAGTCGGAGTCG
CTAGTAATCGCAGATCAGCAACGCTGCGGTGAATACGTTCCCGGGTCTTGTACACACCGCCCGTCAAGTCATGAAAGTCGGTAACACCTGAAGCCGGTGG
CCCAACCCTTGTGGAGGGAGCCGTCGAAAGTGGGATCGGTATTAGGACTAATCGAAAAAAGGGGGG

>EB382
GCTCAGGATGAACGCTGGCGGCGTGCTTAACACATGCAAGTCGAACGGTGAAGCCCAGCTTGCTGGGTGGATCAGTGGCGAACGGGTGAGTAACACGTGA
GCAATCTGCCCCTGACTCTGGGATAAGCGCTGGAAACGGCGTCTAATACTGGATACGAACCGTGGAGGCATCTTCAACGGTTGGAAAGATTTTTTGGTCA
GGGATGAGCTCGCGGCCTATCAGCTTGTTGGTGAGGTAATGGCTCACCAAGGCGTCGACGGGTAGCCGGCCTGAGAGGGTGACCGGCCACACTGGGACTG
AGACACGGCCCAGACTCCTACGGGAGGCAGCAGTGGGGAATATTGCACAATGGGCGCAAGCCTGATGCAGCAACGCCGCGTGAGGGATGACGGCCTTCGG
GTTGTAAACCTCTTTTAGCAGGGAAGAAGCGAAAGTGACGGTACCTGCAGAAAAAGCGCCGGCTAACTACGTGCCAGCAGCCGCGGTAATACGTAGGGCG
CAAGCGTTATCCGGAATTATTGGGCGTAAAGAGCTCGTAGGCGGTTTGTCGCGTCTGCTGTGAAATCCCGAGGCTCAACCTCGGGTCTGCAGTGGGTACG
GGCAGACTAGAGTGCGGTAGGGGAGATTGGAATTCCTGGTGTAGCGGTGGAATGCGCAGATATCAGGAGGAACACCGATGGCGAAGGCAGATCTCTGGGC
CGTAACTGACGCTGAGGAGCGAAAGGGTGGGGAGCAAACAGGCTTAGATACCCTGGTAGTCCACCCCGTAAACGTTGGGAACTAGTTGTGGGGTCCATTC
CACGGATTCCGTGACGCAGCTAACGCATTAAGTTCCCCGCCTGGGGAGTACGGCCGCAAGGCTAAAACTCAAAGGAATTGACGGGGACCCGCACAAGCGG
CGGAGCATGCGGATTAATTCGATGCAACGCGAAGAACCTTACCAAGGCTTGACATATACGAGAACGCTGCAGAAATGTAGAACTCTTTGGACACTCGTAA
ACAGGTGGTGCATGGTTGTCGTCAGCTCGTGTCGTGAGATGTTGGGTTAAGTCCCGCAACGAGCGCAACCCTCGTTCTATGTTGCCAGCACGTAATGGTG
GGAACTCATGGGATACTGCCGGGGTCAACTCGGAGGAAGGTGGGGATGACGTCAAATCATCATGCCCCTTATGTCTTGGGCTTCACGCATGCTACAATGG
CCGGTACAAAGGGCTGCAATACCGTAAGGTGGAGCGAATCCCAAAAAGCCGGTCCCAGTTCGGATTGAGGTCTGCAACTCGACCTCATGAAGTCGGAGTC
GCTAGTAATCGCAGATCAGCAACGCTGCGGTGAATACGTTCCCGGGTCTTGTACACACCGCCCGTCAAGTCATGAAAGTCGGTAACACCTGAAGCCGGTG
GCCCAACCCTTGTGGAGGGAGCCGTCGAAGGTGGGATCGGTATTAGGATAATCGAC

>EB383
CTGGGATAAGCGCTGGAAACGGCGTCTAATACTGGATACAAACCGTGGAGGCATCTTCAACGGTTGGAAAGATTTTTTGGTCAGGGATGAGCTCGCGGCC
TATCAGCTTGTTGGTGAGGTAATGGCTCACCAAGGCGTCGACGGGTAGCCGGCCTGAGAGGGTGACCGGCCACACTGGGACTGAGACACGGCCCAGACTC
CTACGGGAGGCAGCAGTGGGGAATATTGCACAATGGGCGCAAGCCTGATGCAGCAACGCCGCGTGAGGGATGACGGCCTTCGGGTTGTAAACCTCTTTTA
GCAGGGAAGAAGCGAAAGTGACGGTACCTGCAGAAAAAGCGCCGGCTAACTACGTGCCAGCAGCCGCGGTAATACGTAGGGCGCAAGCGTTATCCGGAAT
TATTGGGCGTAAAGAGCTCGTAGGCGGTTTGTCGCGTCTGCTGTGAAATCCCGAGGCTCAACCTCGGGTCTGCAGTGGGTACGGGCAGACTAGAGTGCGG
TAGGGGAGATTGGAATTCCTGGTGTAGCGGTGGAATGCGCAGATATCAGGAGGAACACCGATGGCGAAGGCAGATCTCTGGGCCGTAACTGACGCTGAGG
AGCGAAAGGGTGGGGAGCAAACAGGCTTAGATACCCTGGTAGTCCACCCCGTAAACGTTGGGAACTAGTTGTGGGGTCCATTCCACGGATTCCGTGACGC
AGCTAACGCATTAAGTTCCCCGCCTGGGGAGTACGGCCGCAAGGCTAAAACTCAAAGGAATTGACGGGGACCCGCACAAGCGGCGGAGCATGCGGATTAA
TTCGATGCAACGCGAAGAACCTTACCAAGGCTTGACATATACGAGAACGCTGCAGAAATGTAGAACTCTTTGGACACTCGTAAACAGGTGGTGCATGGTT
GTCGTCAGCTCGTGTCGTGAGATGTTGGGTTAAGTCCCGCAACGAGCGCAACCCTCGTTCTATGTTGCCAGCACGTAATGGTGGGAACTCATGGGATACT
GCCGGGGTCAACTCGGAGGAAGGTGGGGATGACGTCAAATCATCATGCCCCTTATGTCTTGGGCTTCACGCATGCTACAATGGCCGGTACAAAGGGCTGC
AATACCGTAAGGTGGAGCGAATCCCAAAAAGCCGGTCCCAGTTCGGATTGAGGTCTGCAACTCGACCTCATGAAGTAGGAGTCGCTAGTAATCGCAGATC
AGCAACGTTGCGGTGAATACGTTCCCGGGTCTTGTACACACCGCCCGTCAAGTCATGAACGTCGGTAACAC

>EB384
GAGTAACACGTGAGCAATCTGCCCCTGACTCTGGGATAAGCGCTGGAAACGGCGTCTAATACTGGATACGAACCGTGGAGGCATCTTCAACGGTTGGAAA
GATTTTTTGGTCAGGGATGAGCTCGCGGCCTATCAGCTTGTTGGTGAGGTAATGGCTCACCAAGGCGTCGACGGGTAGCCGGCCTGAGAGGGTGACCGGC
CACACTGGGACTGAGACACGGCCCAGACTCCTACGGGAGGCAGCAGTGGGGAATATTGCACAATGGGCGAAAGCCTGATGCAGCAACGCCGCGTGAGGGA
TGACGGCCTTCGGGTTGTAAACCTCTTTTAGCAGGGAAGAAGCGAAAGTGACGGTACCTGCAGAAAAAGCGCCGGCTAACTACGTGCCAGCAGCCGCGGT
AATACGTAGGGCGCAAGCGTTATCCGGAATTATTGGGCGTAAAGAGCTCGTAGGCGGTTTGTCGCGTCTGCTGTGAAATCCCGAGGCTCAACTTCGGGTC
TGCAGTGGGTACGGGCAGACTAGAGTGCGGTAGGGGAGATTGGAATTCCTGGTGTAGCGGTGGAATGCGCAGATATCAGGAGGAACACCGATGGCGAAGG
CAGATCTCTGGGCCGTAACTGACGCTGAGGAGCGAAAGGGTGGGGAGCAAACAGGCTTAGATACCCTGGTAGTCCACCCCGTAAACGTTGGGAACTAGTT
GTGGGGTCCATTCCACGGATTCCGTGACGCAGCTAACGCATTAAGTTCCCCGCCTGGGGAGTACGGCCGCAAGGCTAAAACTCAAAGGAATTGACGGGGA
CCCGCACAAGCGGCGGAGCATGCGGATTAATTCGATGCAACGCGAAGAACCTTACCAAGGCTTGACATATACGAGAACGCTGCAGAAATGTAGAACTCTT
TGGACACTCGTAAACAGGTGGTGCATGGTTGTCGTCAGCTCGTGTCGTGAGATGTTGGGTTAAGTCCCGCAACGAGCGCAACCCTCGTTCTATGTTGCCA
GCACGTAATGGTGGGAACTCATGGGATACTGCCGGGGTCAACTCGGAGGAAGGTGGGGATGACGTCAAATCATCATGCCCCTTATGTCTTGGGCTTCACG
CATGCTACAATGGCCGGTACAAAGGGCTGCAATACCGTGAGGTGGAGCGAATCCCAAAAAGCCGGTCCCAGTTCGGATTGAGGTCTGCAACTCGACCTCA
TGAAGTCGGAGTCGCTAGTAATCGCAGATCAGCAACGCTGCGGTGAATACGTTCCCGGTTGTTGTACACACCGCCCCTCAAGTCAGGAAC

>EB385
CCACCGGCTTCAGGTGTTACCGACTTTCATGACTTGACGGGCGGTGTGTACAAGACCCGGGAACGTATTCACCGCAGCGTTGCTGATCTGCGATTACTAG
CGACTCCGACTTCATGAGGTCGAGTTGCAGACCTCAATCCGAACTGGGACCGGCTTTTTGGGATTCGCTCCACCTCACGGTATTGCAGCCCTTTGTACCG
GCCATTGTAGCATGCGTGAAGCCCAAGACATAAGGGGCATGATGATTTGACGTCATCCCCACCTTCCTCCGAGTTGACCCCGGCAGTATCCCATGAGTTC
CCACCATTACGTGCTGGCAACATAGAACGAGGGTTGCGCTCGTTGCGGGACTTAACCCAACATCTCACGACACGAGCTGACGACAACCATGCACCACCTG
TATAGAGACCTTGCGGGGCGACTGTTTCCAGACGTTTCCTCTATATGTCAAGCCTTGGTAAGGTTCTTCGCGTTGCATCGAATTAATCCGCATGCTCCGC
CGCTTGTGCGGGTCCCCGTCAATTCCTTTGAGTTTTAGCCTTGCGGCCGTACTCCCCAGGCGGGGAACTTAATGCGTTAGCTGCGTCACGGAATCCGTGG
AATGGACCCCACAACTAGTTCCCAACGTTTACGGGGTGGACTACCAGGGTATCTAAGCCTGTTTGCTCCCCACCCTTTCGCTCCTCAGCGTCAGTTACGG
CCCAGAGATCTGCCTTCGCCATCGGTGTTCCTCCTGATATCTGCGCATTCCACCGCTACACCAGGAATTCCAATCTCCCCTACCGCACTCTAGTCTGCCC
GTACCCACTGCAGGCCCGAGGTTGAGCCTCGGGTTTTCACAGCAGACGCGACAGACCGCCTACGAGCTCTTTACGCCCAATAATTCCGGATAACGCTTGC
GCCCTACGTATTACCGCGGCTGCTGGCACGTAGTTAGCCGGCGCTTTTTCTGCAGGTACCGTCACTTTCGCTTCTTCCCTGCTAAAAGAGGTTTACAACC
CGAAGGCCGTCATCCCTCACGCGGCGTTGCTGCATCAGGCTTGCGCCCATTGTGCAATATTCCCCACTGCTGCCTCCCGTAGGAGTCTGGGCCGTGTCTC
AGTCCCAGTGTGGCCGGTCACCCTCTCAGGCCGGCTACCCGTCGACGCCTTGGTGAGCCATTACCTCACCAACAAGCTGATAGGCCGCGAGCTCATCCCT
GACCGAAATTCTTTCCAGCTACTGACCATGCGGTCGCAGCTCGTATCCGGTATTAGACGCCGTTTCCAGCGCTTATCCCAGAGTCAGGGGCAGATTGCTC
ACGTGTTACTCACCCGTTCGCCAATGATCACACAGAGCAAGCTATGATTCA

>EB386

TAGAGTACGGGTTCGGAAAGATTTTTCGTCTGGGATGGGCTCGCGGCCTATCAGCTTGTTGGTGAGGTAATGGCTCACCAAGGCGTCGACGGGTAGCCGG
CCTGAGAGGGTGACCGGCCACACTGGGACTGAGACACGGCCCAGACTCCTACGGGAGGCAGCAGTGGGGAATATTGCACAATGGGCGCAAGCCTGATGCA
GCAACGCCGCGTGAGGGATGACGGCCTTCGGGTTGTAAACCTCTTTTAGCAGGGAAGAAGCGTAAGTGACGGTACCTGCAGAAAAAGCGCCGGCTAACTA
CGTGCCAGCAGCCGCGGTAATACGTAGGGCGCAGCGTTATCCGGAATTATTGGGCGTAAAGAGCTCGTAGGCGGTTTGTCGCGTCTGCTGTGAAATCCCG
AGGCTCAACCTCGGGCCTGCAGTGGGTACGGGCAGACTAGAGTGCGGTAGGGGAGATTGGAATTCCTGGTGTAGCGGTGGAATGCGCAGATATCAGGAGG
AACACCGATGGCGAAGGCAGATCTCTGGGCCGTAACTGACGCTGAGGAGCGAAAGGGTGGGGAGCAAACAGGCTTAGATACCCTGGTAGTCCACCCCGTA
AACGTTGGGAACTAGTTGTGGGGACCATTCCACGGTTTCCGTGACGCAGCTAACGCATTAAGTTCCCCGCCTGGGGAGTACGGCCGCAAGGCTAAAACTC
AAAGGAATTGACGGGGACCCGCACAAGCGGCGGAGCATGCGGATTAATTCGATGCAACGCGAAGAACCTTACCAAGGCTTGACATACACCAGAACACCGT
AGAAATACGGGACTCTTTGGACACTGGTGAACAGGTGGTGCATGGTTGTCGTCAGCTCGTGTCGTGAGATGTTGGGTTAAGTCCCGCAACGAGCGCAACC
CTCGTTCTATGTTGCCAGCACGTAATGGTGGGAACTCATGGGATACTGCCGGGGTCAACTCGGAGGAAGGTGGGGATGACGTCAAATCATCATGCCCCTT
ATGTCTTGGGCTTCACGCATGCTACAATGGCCGGTACAAAGGCTGCAATACCGTGAGGTGGAGCGAATCCCAAAAAGCCGGTCCCAGTTCGGATTGAGGT
CTGCAACTCGACCTCATGAAGTCGGAGTCGCTAGTAATCGCAGATCAGCAACGCTGCGGTGAATACGTTCCCGGGTCTTGTACACACCGCCCGTCAAGTC
ATGAAAGTCGGTAACACCTGAAGCCGGTGGCCCAACCCCCTTTG

>EB387
GGTAACGATGTTCCGGACTTGAGAGGCGGGGTGTACCACACCCGGGAACGTGTTCACCGCAACGTTGCTGATCTGCGATTACAAGCGACTCCTACTTCAT
GAGGTCGAGTTGCAGACCTCAATCCGAACTGGGACCGGCTTTTTGGGATTCGCTCCACCTCGCGGTATTGCAGCCCTTTGTACCGGCCATTGTAGCATGC
GTGAAGCCCAAGACATAAGGGGCATGATGATTTGACGTCATCCCCACCTTCCTCCGAGTTGACCCCGGCAGTATCCCATGAGTTCCCACCATTACGTGCT
GGCAACATAGAACGAGGGTTGCGCTCGTTGCGGGACTTAACCCAACATCTCACGACACGAGCTGACGACAACCATGCACCACCTGTTTACGAGTGTCCAA
AGAGTTGACCATTTCTGGCCCGTTCTCGTATATGTCAAGCCTTGGTAAGGTTCTTCGCGTTGCATCGAATTAATCCGCATGCTCCGCCGCTTGTGCGGGT
CCCCGTCAATTCCTTTGAGTTTTAGCCTTGCGGCCGTACTCCCCAGGCGGGGAACTTAATGCGTTAGCTGCGTCACGGAATCCGTGGAATGGACCCCACA
ACTAGTTCCCAACGTTTACGGGGTGGACTACCAGGGTATCTAAGCCTGTTTGCTCCCCACCCTTTCGCTCCTCAGCGTCAGTTACGGCCCAGAGATCTGC
CTTCGCCATCGGTGTTCCTCCTGATATCTGCGCATTCCACCGCTACACCAGGAATTCCAATCTCCCCTACCGCACTCTAGTCTGCCCGTACCCACTGCAG
GCCCGAGGTTGAGCCTCGGGATTTCACAGCAGACGCGACAAACCGCCTACGAGCTCTTTACGCCCAATAATTCCGGATAACGCTTGCGCCCNTACGTATT
ACCGCGGCTGCTGGCACGTAGTTAGCCGGCGCTTTTTCTGCAGGTACCGTCACTTTCGCTTCTTCCCTGCTAAAAGAGGTTTACAACCCGAAGGCCGTCA
TCCCTCACGCGGCGTTGCTGCATCAGGCGTGCGCCCATTGTGCAATATTCCCCACTGCTGCCTCCCGTAGGAGTCTGGGCCGTGTCTCAGTCCCAGTGTG
GCCGGTCACCCTCTCAGGCCGGCTACCCGTCGACGCCTTGGTGAGCCATTACCTCACCAACAAGCTGATAGGCCGCGAGCCCATCCCCAACCGAAATTCT
TTCCAGTAACTGACCATGCGGTCGCTACAAAGTATCCAATATTCGACGCCGTGTCCAGCGAGCCACCATCAAACAGGGGCGGTTACCTTGTTACGACTTA
CTGAGCCATAATCAAACTCTACGGTTACCTTGTTACGACTTACTGAGCCATGATCAAACTCT

>EB388
GATCATGGCTCAGTAAGTCGTAGCAAGGTACCCGTATAGTTTGATCATGGCTCAAGTAGTCGTGAGCGGCTGCCCTGGACTCTGGGATAAGCGCTGGAAA
CGGTGTCTAATACCGGATATGAGCCACGGCCGCATGGTCGGTGGTTGGAAAGATTTTTTGGTCTGGGATGGGCTCGCGGCCTATCAGCTTGTTGGTGAGG
TAATGGCTCACCAAGGCGTCGACGGGTAGCCGGCCTGAGAGGGTGACCGGCCACACTGGGACTGAGACACGGCCCAGACTCCTACGGGAGGCAGCAGTGG
GGAATATTGCACAATGGGCGGAAGCCTGATGCAGCAACGCCGCGTGAGGGATGACGGCCTTCGGGTTGTAAACCTCTTTTAGCAGGGAAGAAGCGTGAGT
GACGGTACCTGCAGAAAAAGCGCCGGCTAACTACGTGCCAGCAGCCGCGGTAATACGTAGGGCGCAAGCGTTATCCGGAATTATTGGGCGTAAAGAGCTC
GTAGGCGGTTTGTCGCGTCTGCTGTGAAATCCCGAGGCTCAACTTCGGGCTTGCAGTGGGTACGGGCAGACTAGAGTGCGGTAGGGGAGATTGGAATTCC
TGGTGTAGCGGTGGAATGCGCAGATATCAGGAGGAACACCGATGGCGAAGGCAGATCTCTGGGCCGTAACTGACGCTGAGGAGCGAAAGGGTGGGGAGCA
AACAGGCTTAGATACCCTGGTAGTCCACCCCGTAAACGTTGGGAACTAGTTGTGGGGGCCATTCCACGGTCTCCGTGACGCAGCTAACGCATTAAGTTCC
CCGCCTGGGGAGTACGGCCGCAAGGCTAAAACTCAAAGGAATTGACGGGGACCCGCACAAGCGGCGGAGCATGCGGATTAATTCGATGCAACGCGAAGAA
CCTTACCAAGGCTTGACATACACCAGAACACCGTAGAAATACGGGACTCTTTGGACACTGGTGAACAGGTGGTGCATGGTTGTCGTCAGCTCGTGTCGTG
AGATGTTGGGTTAAGTCCCGCAACGAGCGCAACCCTCGTTCTATGTTGCCAGCACGTTATGGTGGGAACTCATGGGATACTGCCGGGGTCAACTCGGAGG
AAGGTGGGGATGACGTCAAATCATCATGCCCCTTATGTCTTGGGCTTCACGCATGCTACAATGGCCGGTACAAAGGGCTGCAATACCGTGAGGTGGAGCG
AATCCCAAAAAGCCGGTCCCAGTTCGGAATGAGGTCTGCAACTCGACCTCATGAAGTAGGAGTCGCTTATAATCGCAGATCAGCACCGTAGCTGGGAACA
CGTTACCGGTTGAGTACACCACGGCTCACTAATTCGTAACAAGGTAACCGTAGAG

>EB389
CTCAGGACGAACGTGGCGGCATGCCTATACATGCAAGTCGAGCGGAGTTTTGAGAAGCTTGCTTCTCTGATGCTTAGCGGCGGACGGGTGAGTAACACGT
AGGCAAGATGCCCTCAATCTTGGGACAACTACCGGAAACGGTATCTAATATCGAATACTTGCTTTGTTTGTATGAAGGAAGCTGGAAAGACGGAGCAATC
TGTCACTTGAGGATGGGCTTGCGGCGCATTAGCTAGTTGGTGAGGTAACGGCTCACCAAGGCGTCGATGCGTAGCCGACCTGAGAGGGTGATCGGCCACA
CTGGGATTGAGACACGCCCCAGAATCGTGCGGGAGGCAGCAGTAGTGAATGTTGCGCAATGGGCGAANNCCTGACGGAGCAATGCCGCGTGAGTGATGAA
GGTYTTCGGATCGTAAAGCTCTGTTGCCAGGGAAGAACGTCCTTGMGAGTAACTGCTCAAGGAGTGACGGTACCTGAGAAGAAAGCCCCGGCTAACTACG
TGCCAGCAGCCGCGGTAATACGTARGGGGCAAGCGTTGTCCGGAATTATTGGGCGTAAAGCGCGYGCAGGCRGTCATTTAAGTCTGGTGTTTAATCCCGG
GGCTCAACCCCGGATCGCACTGGAAACTGGATGACTTGAGTGCAGAAGAGGAGAGTGGAATTCCACGTGTAGCGGTGAAATGCGTAGAGATGTGGAGGAA
CACCAGTGGCGAARGCGACTCTCTGGRCTGTAACTGACGCTRAGGCGCGAAAGCGTGGGGAGCAAACAGGATTAGATACCCTGGTAGTCCACGCCKTARA
CGATGAATGCTAGGTGTTAGGGGTTTCGATACCCTTGKTGCCGMAAGTAACGCATTAAACATTCCGCCTGGGGAGTACCGTCGCAAGACTGAAACTCACA
GGAATTGACGGGAACCCGCACAATCAGTGGAGTATGTGGTTTAAATCTTAGCAACGCTTAAAATCTTACAAAGTCTTGACATCCTTCTGACCGCTCTAGA
CATACGTCTTTCCTTCGGGACAGATTATACACGTGTCGCATGGTTGTCGTCAGCTCGTGTCGTGAGATGTTGGGTTAACTCCCGCAACGATCGCAACCCT
TGATCTTAGTTGCCACCACTTCTGGTGGGCACTCTAACGTGAATGACGGTGACAAACCGGACGAAGGTGGGGATGACGTCGAATCAGTCATGCCCCTTAT
GACCTGTGCTACGCACGTACTACAATGGCCGGTACAACGTGCAGTGAACCCGCGAGGTGGAACGAATCTCAGAAAGCCGGTCTCAATTCGGATTGCAGGC
TGCAAATCCCCTGCATGAACTCGAAATGCTAGTAATCGCGGAT
